# Comparative genomic analysis and epigenetic profiling of the *Drosophila melanogaster* B chromosome

**DOI:** 10.1093/genetics/iyag113

**Published:** 2026-05-05

**Authors:** Ana B S M Ferretti, Diogo Milani, Emiliano Martí, Rhavenna T Alves-Gomes, Maria D Vibranovski, Stacey L Hanlon, Diogo C Cabral-de-Mello

**Affiliations:** Departamento de Biologia Geral e Aplicada, UNESP—Univ Estadual Paulista, Instituto de Biociências/IB, Rio Claro, São Paulo 13606-900, Brazil; School of Mathematical and Natural Sciences, New College of Interdisciplinary Arts and Sciences, Arizona State University, Glendale, AZ 85306, United States; Departamento de Biologia Geral e Aplicada, UNESP—Univ Estadual Paulista, Instituto de Biociências/IB, Rio Claro, São Paulo 13606-900, Brazil; Department of Biology, University of Rochester, Rochester, NY 14627, United States; Departamento de Biologia Geral e Aplicada, UNESP—Univ Estadual Paulista, Instituto de Biociências/IB, Rio Claro, São Paulo 13606-900, Brazil; School of Mathematical and Natural Sciences, New College of Interdisciplinary Arts and Sciences, Arizona State University, Glendale, AZ 85306, United States; Department of Genetics and Evolutionary Biology, University of São Paulo, São Paulo, São Paulo 05508-090, Brazil; Department of Molecular and Cell Biology, Genetics and Genomics, University of Connecticut, Storrs, CT 06269, United States; Institute for Systems Genomics, University of Connecticut, Storrs, CT 06269, United States; Departamento de Biologia Geral e Aplicada, UNESP—Univ Estadual Paulista, Instituto de Biociências/IB, Rio Claro, São Paulo 13606-900, Brazil

**Keywords:** B chromosome, epigenetic silencing, repetitive DNA, satellite DNAs, transposable elements, cytogenetics, karyotype evolution

## Abstract

Supernumerary B chromosomes have been identified in hundreds of species across many different taxa, each with their own evolutionary history, unique sequence composition, and epigenetic profile. In this study, we generated a detailed repetitive sequence and epigenetic landscape of the B chromosomes in *Drosophila melanogaster*. By comparing the repetitive DNA content of stocks with (+B) and without (0B) B chromosomes, we found 9 satellite DNA (satDNA) sequences that were enriched in the +B genome, 3 of which were cytogenetically confirmed to be on the B chromosomes. Our satDNA analysis also led to the discovery of 3 novel satDNA repeats that are not on the B chromosomes and have not been described previously (Sat-307, Sat-597, and Sat-228). Analysis of transposable elements (TEs) revealed that although TEs comprise similar proportions of the 0B and +B genomes, the centromeric transposon *G2/Jockey* is modestly enriched in the +B genome, consistent with the high number of centromeres present in the +B genome. We also immunostained both male somatic and germline tissues to gain insight into the epigenetic state of the B chromosomes and found the presence of heterochromatin-associated histone modifications (H3K9me1, H3K9me2), a lack of acetylation (H4K5ac), and the absence of RNA polymerase II in the B chromosome nuclear territory, suggesting the B chromosomes have a reduced level of transcription. Together, our work further defines the genetic composition of the *D. melanogaster* B chromosome and establishes its epigenetic profile, which will provide a foundation for future work investigating changes to repetitive DNA over time and how a transcriptionally repressed chromosome influences chromatin maintenance in the genome.

## Introduction

B chromosomes are nonessential chromosomes that appear in addition to the regular chromosome complement (A chromosomes) in a wide range of eukaryotic groups (Jones 2017; Oliveira et al. 2024). Most B chromosomes are thought to have originated from the standard set after a catastrophic event, such as an error during cell division that promotes chromosome lagging and micronucleation, which then may lead to extensive breakage and rearrangements (Houben et al. 2025). Although in the past B chromosomes were mainly known as inert elements, recent advances in genomics have changed this view. The sequence composition and functional analyses of several B chromosomes have provided examples of their effect at cellular and organismal levels across different species, thus B chromosomes are considered to be active “genomic players” (Ahmad and Martins 2019; Dalla Benetta et al. 2019; Johnson Pokorná and Reifová 2021; Rajpal et al. 2023; Ferree et al. 2024; Oliveira et al. 2024). B chromosomes are often primarily composed of repetitive DNA, such as satellite DNAs (satDNAs) and transposable elements (TEs), although some protein-coding genes have been identified (Valente et al. 2014; Huang et al. 2016; Navarro-Domínguez et al. 2017). Multigene families, including rDNA and histones, have also been reported on these chromosomes (Camacho 2005; Ahmad and Martins 2019; Camacho et al. 2021; Oliveira et al. 2024). Since B chromosomes do not typically recombine with the A chromosomes, and they are subject to a different set of selective pressures, the evolutionary trajectories of the B chromosomes are distinct from those of A chromosomes (Camacho et al. 2000, 2021; Ahmad and Martins 2019; Rajpal et al. 2023).

The presence, composition, and origin of B chromosomes in insects have been predominantly studied in grasshoppers (Muñoz-Pajares et al. 2011; Bernardino et al. 2017; Jetybayev et al. 2018; Milani et al. 2018, 2021), bees and wasps (Perfectti and Werren 2001; Werren and Stouthamer 2003; Novaes et al. 2021), fungus gnats (Hodson et al. 2022), and beetles (Cabral-de-Mello et al. 2010; Amorim et al. 2016). However, a recent study identified B chromosomes in *Drosophila melanogaster* (Bauerly et al. 2014), a powerhouse genetic and genomic system (Jenkins et al. 2022; Öztürk-Çolak et al. 2024), offering a unique opportunity to address fundamental questions of B chromosome biology including their origin, composition, regulation, and modes of inheritance. These B chromosomes are thought to originate from chromosome 4, where centromeric fragments from the short left arm fused and formed a stable chromosome (Hanlon et al. 2018). This model is consistent with the findings that the B chromosomes have functional centromeres and telomeres and are enriched for several simple satellite repeats that are found on chromosome 4, including *AATAT, AAGAG* and *AAGAT* (Bauerly et al. 2014; Hanlon et al. 2018). The presence of these repeats on the B chromosomes has been confirmed cytogenetically by fluorescence *in situ* hybridization (FISH), as well as genomically through the analysis of sequencing data generated from isolated B chromosomes that were separated from the rest of the A chromosomes using pulsed field gel electrophoresis (PFGE) (Hanlon et al 2018). Similar to the left arm of chromosome 4, the B chromosomes are likely entirely heterochromatic and can induce position effect variegation (PEV), suggesting that they influence chromatin organization that can have consequences on gene expression (Bauerly et al. 2014).

The rapid turnover of repetitive sequences in *Drosophila* (Petrov et al. 2011; Wei et al. 2014) and their role in the evolution of B chromosomes prompted us to further investigate the sequence composition and epigenetic profile of the *D. melanogaster* B chromosome. This study aims to provide a deeper molecular characterization of the *D. melanogaster* B chromosome, with a particular focus on satDNA, TEs, and multigene families. Using cutting-edge bioinformatic approaches for repetitive DNA identification and characterization, we performed a comprehensive analysis of the B chromosome sequence by comparing genomic samples from stocks that carry (+B) or do not carry (0B) B chromosomes. We found that the +B genome was enriched (>5× over the 0B samples) for several satDNA sequences, including the *AAGAGAAGAT* 10-mer repeat. Our analysis also revealed 3 previously unidentified satDNA repeats that are not carried by the B chromosomes but are present on the essential chromosomes. The +B genome did not have an overall enrichment for specific TE families, but we did find a modest enrichment (>2× over the 0B samples) for *G2/Jockey*, a retrotransposon associated with *Drosophila* centromeres (Chang et al. 2019; Courret et al. 2024). We also investigated the transcriptional activity of the B chromosome at the cytological level by immunostaining for RNA polymerase and specific histone marks. We found that the B chromosomes form an transcriptionally repressed domain that is free of total RNA polymerase II (total Pol-II) and its activated form (RNA pol II phosphorylated on serine 2 on C-Terminal Domain, RNA pol II S2p-CTD). The chromatin in this domain is hypermethylated and hypoacetylated in both somatic and germline cells of males, indicating that the B chromosomes have a reduced level of transcription. Overall, our findings provide a more comprehensive profile of the *D. melanogaster* B chromosomes at a genomic and cytological level, which will serve as a basis of future studies that investigate the genomic consequences of harboring these supernumerary chromosomes.

## Materials and methods

### Drosophila stocks

Stocks of *D. melanogaster* were maintained on standard cornmeal media with tegosept at 24 °C. Three stocks were used to identify new B chromosome sequences: the original B chromosome stock “+B” (*y w/y* + *Y; mtrm^126^/TM3, Sb Ser; sv^spa-pol^*), a stock without B chromosomes and no *mtrm^126^* allele, here referred to as “0B-TM3” (*y w/y* + *Y; Pr Dr/TM3, Sb Ser; sv^spa-pol^*), and a laboratory wild-type (WT) stock that does not have B chromosomes and will be referred to as “0B-WT” (*y w/y* + *Y; sv^spa-pol^*). An additional laboratory wild-type Oregon-R (*Wolbachia* free) from Arizona State University stock was used for validation of new satDNAs.

### Genome sequencing

Ten healthy males from each of the 3 stocks (+B, 0B-WT, and 0B-TM3) were collected and quick-frozen in liquid nitrogen, then used for genomic DNA (gDNA) extraction with the Qiagen DNeasy Blood and Tissue Kit following the supplementary protocol for isolation of total DNA from insects. 500 ng of each sample was sonicated via Covaris to 600 bp, then size selected from 500 to 700 bp on a Pippin Prep prior to library construction with the KAPA High Throughput Library Prep kit. The libraries were sequenced on the Illumina HiSeq (+B) or NextSeq (0B-WT and 0B-TM3) platforms at the Stowers Institute for Medical Research in Kansas City, MO. For further analysis and validation of the repeats on the B chromosome, we leveraged a library produced from the original stock where the B chromosomes were isolated from the essential chromosomes by pulsed field gel electrophoresis (PFGE-B) (Hanlon et al. 2018). Raw sequencing data were subjected to quality assessment using FastQC to evaluate base quality, sequence composition, and GC content. All libraries exhibited comparable quality profiles and an identical GC content (41%), indicating the absence of major platform- or batch-specific compositional biases. To further assess the potential effects of sequencing platform on repetitive DNA quantification, we performed principal component analyses (PCA) based on satellite DNA abundance, including analyses using all satellite families and analyses restricted to satellites previously classified as A-linked for *D. melanogaster*. PCA analyses were conducted in R, and sample relationships were further summarized by calculating Euclidean distances between genotype centroids. These analyses were used to assess clustering patterns with respect to biological genotype and sequencing platform. In both cases, libraries clustered according to genotype rather than sequencing platform, supporting that downstream differences in satellite composition reflect biological variation rather than technical artifacts (Supplementary Fig. 1).

### Identification of satellite DNAs

For satDNA identification, we used the satMiner protocol (Ruiz-Ruano et al. 2016) available at GitHub (https://github.com/fjruizruano/satminer). SatMiner allows the identification of satDNAs by several rounds of clustering in RepeatExplorer (Novák et al. 2013), and filtering out cluster of sequences previously identified with DeconSeq (Schmieder and Edwards 2011). We characterize previously unidentified putative satDNA in the *Drosophila* genome, with satMiner, performing 3 rounds of clustering and filtering. We started with 2 × 900,000 and for the second and third round we added 300,000 reads. RepeatExplorer was run using default options as suggested in Ruiz-Ruano et al. (2016). We selected the putative satDNA clusters according to Novák et al. (2013). To determine clusters as satDNAs we reviewed the internal structure of contigs in each cluster using the dotmach in Geneious v4.8 software (Kearse et al. 2012) and Tandem Repeat Finder (TRF; Benson 1999). TRF searches for patterns of tandem repetitions in DNA sequences. To search for homology between satDNAs we performed an all-vs-all comparison of dimers of satDNA consensi with RepeatMasker (Smit et al. 2013) using the “rm_homology.py” script (https://github.com/fjruizruano/ngs-protocols).

### Validation of in-tandem organization of the newly identified satDNAs in *Drosophila melanogaster*

To validate the tandem structure of novel satDNAs, we used the program Repeat Profiler (Negm et al. 2021) with trimers from each tandem repeat to analyze the coverage at the junctions of the monomers. To validate the broad occurrence of the new satDNAs in *D. melanogaster*, we performed this analysis in one genome from each fly stock from this work and in 3 wild-type genomes from NCBI (accession number: SRR1104305, SRR1538755, and SRR23524159). We generated a coverage plot from our analysis that has even coverage across the monomers and their junctions, indicating the new satDNA monomers are arranged in tandem rather than in single copies that are surrounded by intergenic sequence. To validate this in-tandem arrangement, we searched for the satDNAs in PacBio reads (accession number SRR30623885) and checked their arrangement. For this we did a local Blast search using trimer sequences and recovered the fifteen reads with highest similarity. Then, we selected the read presenting the highest similarity and annotated the monomers using the annotation tool from Geneious. Additionally, we determined the chromosomal locations of each tandem repeat through the CHRomosome In Silico MAPPing (CHRISMAPP) pipeline (Rico-Porras et al. 2024) using the FlyBase release 6.58 of the assembled genome. An annotation of each satDNA monomer was performed in Geneious using 80% of similarity and posteriorly was mapped using CHRISMAPP v.2 R script to map directly the annotated satDNA in the chromosome assembly.

### Comparative repetitive DNA sequence analysis for genomic abundance and divergence in +B and 0B genomes

We estimated sequence genomic abundance and divergence of satDNAs, TEs, and multigene families in all our libraries using RepeatMasker (Smit et al. 2013). For this analysis we used 13 satDNA families, including the 3 new satDNAs discovered here (see results) and 11 satDNAs previously described (Ganetzky 1977; Abad et al. 1992, 2000; Lohe et al. 1993; Wei et al. 2014; Bao et al. 2015; Talbert et al. 2018; Bao 2020; de Lima et al. 2020; de Lima and Ruiz-Ruano 2022). A total of 184 TE consensus of *D. melanogaster* from RepBase library (Bao et al. 2015) and 12 multigene families downloaded from NCBI were also used. The multigene families included repeats frequently found on B chromosomes (Ahmad and Martins 2019; Oliveira et al. 2024), i.e. U small nucleolar DNA (snDNA), histone genes, and ribosomal DNAs (rDNA). The consensus sequences for all the elements used in the analysis are included in Supplementary File 1.

Prior to RepeatMasker analyses, all libraries were preprocessed using 2 scripts from the satMiner workflow (Ruiz-Ruano et al. 2016). FASTQ files were first processed with *fastq_edit_ids.py* to standardize read identifiers and remove platform-specific metadata, ensuring uniform paired-end formatting across datasets. Reads were subsequently processed with *rexp_prepare_normaltag.py*, which performs adapter trimming and quality filtering using Trimmomatic (Phred + 33 encoding; sliding-window of 4 bp with a minimum Phred score of 30; minimum read length of 100 bp), followed by random subsampling to 1.5 million paired-end reads per library using *seqtk* (https://github.com/lh3/seqtk), read shuffling, and conversion to FASTA format.

RepeatMasker was then run using normalized datasets, aligning reads against the full library of repetitive sequence consensus sequences. We aligned the reads against all consensus of repetitive sequences and estimated the average Kimura 2-parameter distance (K2P) for each sequence using the script “calcDivergenceFromAlign.pl” from RepeatMasker utilities. The genomic abundance was estimated as the proportion of nucleotides that aligned to the consensus sequences, normalized by the total of nucleotides in each library. For satDNAs with monomer lengths exceeding 100 bp, dimers were generated using their consensus sequences, and for satDNAs with shorter monomers, the consensus sequences were concatenated into fragments of approximately 200 bp. In addition to the satDNA abundance estimation using RepeatMasker, low-complexity satDNAs (*AAGAG*, *AAGAT*, *AATAT*, *AAGAGAAGAT*, *Prodsat*, *dodeca-11*, and *dodeca-12*) were subject to k-mer-based analysis, as it has a better performance for this type of repeat (Wei et al. 2014, 2018). Finally, the genomic abundance and divergence are presented as the average across libraries of each genotype, including 4 genomes 0B-WT, 4 genomes 0B-TM3, 2 genomes +B and the PFGE-B library. We then calculated the enrichment of each sequence in the +B genomes by computing the fold change relative to 0B-WT and 0B-TM3. A sequence was considered not enriched (<2×), modestly enriched (≥2× but <5×), or enriched (≥5×). We selected some repetitive elements from our list as targets for fluorescence in situ hybridization to (1) confirm the presence of these elements on the B chromosome, and (2) determine which of the chromosomes from complement A also carry the sequence and its approximated location (e.g. proximal to the centromere, at the distal tip, etc.).

### Slide mounting and fluorescence in situ hybridization (FISH)

For assessing the cytogenetic location of the 3 novel satDNA repeats, the following protocol was applied to male third-instar larvae. Brains were dissected in saline solution (physiological solution) and transferred for 0.75 M potassium chloride (KCl) for hypotonic treatment for 10 min, then fixed in modified Carnoy's solution (3:1, absolute ethanol: glacial acetic acid). A single brain was placed on a slide with a droplet (∼20 µL) of 50% glacial acetic acid. The material was disaggregated and spread on the slide on top of a heating plate at 45 °C until the solution evaporated.

Cytological mapping of selected sequences was done following Cabral-de-Mello and Marec (2021) with probes directly labeled with Biotin, Cy3, ATTO 590, or AlexaFluor488 at the 3′ (Supplementary Table 1). Probes labeled with Biotin were detected with Streptavidin Alexa Fluor 488-conjugated (Invitrogen, San Diego, CA, USA). After the hybridization, the preparations were counterstained with 4′,6-diamidino-2′-phenylindole (DAPI) and mounted in VECTASHIELD (Vector, Burlingame, CA, USA). The slides were observed using an Olympus microscope BX61 equipped with a fluorescence lamp and appropriate filters. The images were documented with a DP70-cooled digital camera in grayscale and then pseudo-colored. Images were merged and optimized for brightness and contrast with Adobe Photoshop CS6.

### Competitive inhibitor FISH

A probe targeting the *AAGAGAAGAT* satDNA repeat may also bind to stretches of *AAGAG* or *AAGAT* and provide a false positive signal for the location of *AAGAGAAGAT* within the genome. To counter this, FISH with fluorescently labeled probes for *AAGAT* and *AAGAGAAAGAT* was conducted in the presence of unlabeled probes targeting *AAGAG* or *AAGAT* (see Supplementary Table 1). These unlabeled probes are in excess (>20×) of the labeled probes; any competitive binding by these probes will result in the reduction or elimination of FISH signal. Indeed, we saw a significant reduction in normal *AAGAT* signal in the presence of an unlabeled *AAGAT* probe, as well as the complete absence of *AAGAGAAGAT* signal when an unlabeled *AAGAGAAGAT* probe was also present, indicating the success of our competitive inhibitor approach (Supplementary Fig. 2).

The protocol for FISH from a previous study was applied to brains obtained from male third instar larvae (Hanlon et al. 2018). Briefly, brains were removed third instar larvae in 0.7% sodium chloride, and then placed in a hypotonic treatment of 0.5% sodium citrate for 5 min. Brains were then moved to a fixative solution (2.5% paraformaldehyde, 45% acetic acid) for 4 min, and then squashed with a hand clamp between a slide and a siliconized coverslip for 2 min. Following freezing in liquid nitrogen for 5 min, slides were dehydrated in cold (−20 °C) 70% ethanol for 10 min followed by cold 100% ethanol for 10 min. Slides were then air dried before FISH.

For FISH, 21 µL of hybridization solution (50% formamide, 10% dextransulfate, 2× SSC, 100 ng of each fluorescence-labeled probe, 2 µg of each unlabeled probe, see Supplementary Table 1) was applied to the dried sample. A coverslip was placed on top and the slide was heated to 95 °C for 5 min, followed by an overnight incubation at 30 °C to ensure hybridization equilibrium was reached. Slides were then washed 3 times with 0.1× SSC, blown dry using compressed air, and mounted with Vectashield + DAPI to be imaged immediately. To minimize variability between samples, slides were imaged on a DeltaVision Ultra widefield microscope using identical imaging parameters, including exposure time, light transmission percentage, stack settings (3 µm thickness with 0.2 µm *z*-steps), filter sets, and deconvolution post-processing settings. For each sample, the cytogenetic location of FISH signal was assessed by going through each *z*-stack. For figure images, a single representative slice from each experiment was adjusted for brightness and contrast by setting the minimum window intensity value to the equivalent of the background (∼100 to 200 for the red and green channels, ∼1,700 for the blue channel) and the maximum intensity window value to 2,141 (red channel), 2,308 (green channel), and 25,547 (blue channel).

### Immunofluorescence-FISH (IF-FISH)

We used 6 different antibodies to investigate the patterns of transcriptional activation and inactivation in the B chromosome nuclear territory. We explored transcriptional activation using a total Pol-II antibody associated with active chromatin, along with markers for transcription initiation—Serine 5 phosphorylation of the C-Terminal Domain (CTD) of Pol-II (pSer5-CTD Pol-II)—and transcriptional elongation—Serine 2 phosphorylation (pSer2-CTD Pol-II) (Phatnani and Greenleaf 2006; Mahadevaraju et al. 2021). Additionally, we examined epigenetic histone modifications, specifically acetylation (H4K5ac) and mono (H3K9me1) and di-methylation (H3K9me2) (Cowell et al. 2002; Sims et al. 2003; Hennig and Weyrich 2013). The full list of antibodies is detailed in Supplementary Table 2. To precisely locate the B chromosome territory, we performed FISH mapping using the *AAGAT* sequence, which is enriched on both the B and the 4th chromosomes (Hanlon et al. 2018), followed by immunofluorescence. The signal of the probe was validated in both +B and Oregon-R (0B) male cells, including brain somatic cells and spermatocytes, which were also analyzed by IF (Supplementary Fig. 3).

For the analysis of somatic cells, we dissected brains from third instar male larvae, and for germline analysis we dissected testes from virgin adult males. After dissections in cold PBS, brains were transferred to 4% paraformaldehyde in PBS and fixed for 30 min, followed by three 5 min washes in 1 × PBS. We macerated 5 brains with 20 µL 1 × PBS directly on a poly-lysine coated slide. After dissecting the testes (10 per slide), we removed the pre and postmeiotic phases to retain primarily spermatocytes (Vibranovski et al. 2009) that were fixed as previously described for 15 min. We gently squashed spermatocytes between the poly-lysine slide and a coverslip, immersed them in liquid nitrogen, and then placed them in ice-cold 1 × PBS until the blocking step (maximum of 15 min). The slides were then incubated in a humid chamber for 10 min with 50 µL of blocking buffer (BSA 3%, 0.1% Triton-X in 1 × PBS).

For the immunofluorescence (IF), we incubated the slides with the primary antibody diluted in blocking solution overnight at 4 °C. The dilution was done according to the antibody used (for RNApol and acetylation it was 1:300; for histone methylation it was 1:100). The slides were washed 3 times for 5 min in PBST (0.1% Triton-X in PBS 1×) and incubated the specific secondary antibody (anti-rabbit or anti-mouse diluted 1:100) at 37 °C for 1 h. The slides were washed 2 × 5 min in 1 × PBST. To proceed with the FISH after IF experiment, the slides were post-fixed in 100 μL of 3.7% formaldehyde in 1 × PBS for 15 min at room temperature, washed 2 × 5 min in 1 × PBST, and dehydrated in 70% ethanol for 2 min, followed by 100% ethanol for 2 min. The slides were incubated with the same hybridization mix used for FISH (see above) at 75 °C for 1 min to denature DNA, then incubated at 37 °C overnight in a humid chamber. After the incubation, the slides were washed 3 times for 5 min with 4×SSCT (0.1% Triton-X in 4×SSC). The chromosomes were counterstained with DAPI and slides were mounted like in the FISH protocol (Cabral-de-Mello and Marec 2021). The imaging was performed in a Nikon AX R Confocal microscope at the Advanced Light Microscopy Facility in the Biodesign Institute of Arizona State University.

## Results

### Satellite DNA composition of *D. melanogaster* 0B and +B genomes

For hundreds of generations, the B chromosome stock (+B) has been a closed population. On average, each fly in this stock carries 10 to 12 B chromosomes, which equates to roughly 20 Mbp of additional repetitive, heterochromatic sequence in its genome. To understand the impact of maintaining these B chromosomes on the abundance and diversity of satDNA and TEs, we first used RepeatExplorer/SatMiner to identify satDNAs in the 0B and +B genomes. We found several previously identified satDNAs, including *1.688* (Abad et al. 2000; de Lima et al. 2020), *Prodsat* (Abad et al. 2000), *Responder* (Ganetzky 1977*)*, *dodeca* (Abad et al. 1992), *AAGAT* (Wei et al. 2014; Talbert et al. 2018), *AAGAG* and *AATAT* (Lohe et al. 1993; De Lima and Ruiz-Ruano 2022), and SAT-2_Dmel (Bao 2020) (Table 1). We found both the 360 and 190 bp subfamilies of the *1.688* satDNA (de Lima et al. 2020), as well as the 11- and 12-mer variants of *dodeca,* respectively: *CCCGTACTGGT* and *CCCGTACTCGGT* (Abad et al. 1992). We designated these sequences as *dodeca-11* (*CCCGTACTGGT*) and *dodeca-12* (*CCCGTACTCGGT*). We also found an arrangement of 2 satDNAs, *AAGAG* and *AAGAT*, forming a tandem array (i.e. *AAGAGAAGAT*).

**Table 1.** General genomic characteristics for the satDNAs identified in the *D. melanogaster* genomes.

SatDNA family	Length (bp)	A + T (%)	0B-WT Genomic abundance (%)	Divergence (%)	0B-TM3 Genomic abundance (%)	Divergence (%)	B + Genomic abundance (%)	Divergence (%)	PFGE-B Genomic abundance (%)	Divergence (%)	B+/0B-WT	B+/0B-TM3
1.688 subfamily 357	357	69.8	1.4664	9.6	1.8431	9.7	1.4156	9.7	0.4873	9.9	1.0	0.8
subfamily 179	179	70.9	0.5402	6.4	0.7019	6.0	0.5279	6.2	0.1843	6.5	1.0	0.8
Prodsat	10	80	0.4913	5.1	0.5993	4.4	0.9331	3.8	0.3213	4.2	1.9	1.6
AAGAT	5	80	0.0027	6.0	0.0022	5.8	0.1678	3.5	7.0073	4.0	62.5	76.7
Responder	117	68.4	0.0301	10.0	0.0321	10.1	0.1256	9.3	0.0241	9.4	4.2	3.9
Dodeca-11	11	36.7	0.0098	6.1	0.0069	6.2	0.0799	5.6	0.0220	5.4	8.2	11.6
Dodeca-12	12	33.3	0.0072	6.1	0.0052	6.6	0.0495	5.6	0.0328	5.7	6.9	9.5
Sat-307	307	63.2	0.0178	6.3	0.0178	8.8	0.0451	6.0	0.0286	5.3	2.5	2.5
AAGAG	5	60	0.0028	7.8	0.0026	7.5	0.0408	4.4	0.3497	4.7	14.7	15.9
AATAT	5	100	0.0146	5.5	0.0186	5.3	0.0408	4.2	7.3140	4.7	2.8	2.2
Sat-597	597	69	0.0258	6.5	0.0277	6.6	0.0281	6.6	0.0090	7.2	1.1	1.0
Sat-228	228	55.7	0.0070	4.8	0.0093	4.9	0.0149	4.8	0.0032	4.8	2.1	1.6
AAGAGAAGAT	10	70	0.0004	9.1	0.0004	9.4	0.0223	6.3	0.8982	6.2	55.8	55.8
SAT-2_Dmel	246	63.8	0.0113	8.2	0.0104	8.2	0.0128	8.6	0.0043	7.9	1.1	1.2
**Total abundance**			**2.6275**		**3.2774**		**3.5042**		**16.6861**		**1.3**	**1.1**
**Divergence average**				**7.0**		**7.1**		**6.0**		**6.1**		

The table includes the name of each satDNA family, monomer length in base pairs, A + T content percentage. For the 3 genomes analyzed (0B-WT, 0B-TM3, and +B) and the sequenced pulsed field electrophoresis B (PFGE-B) isolated it is shown the genomic abundance (%) and divergence (K2P %) average, the last 2 lines shows the total genomic abundance and the average divergence for all satDNAs. The last 2 columns show the genomic proportion between B+ genome and 0B-WT and 0B-TM3 genome, respectively.

**Fig. 1.**
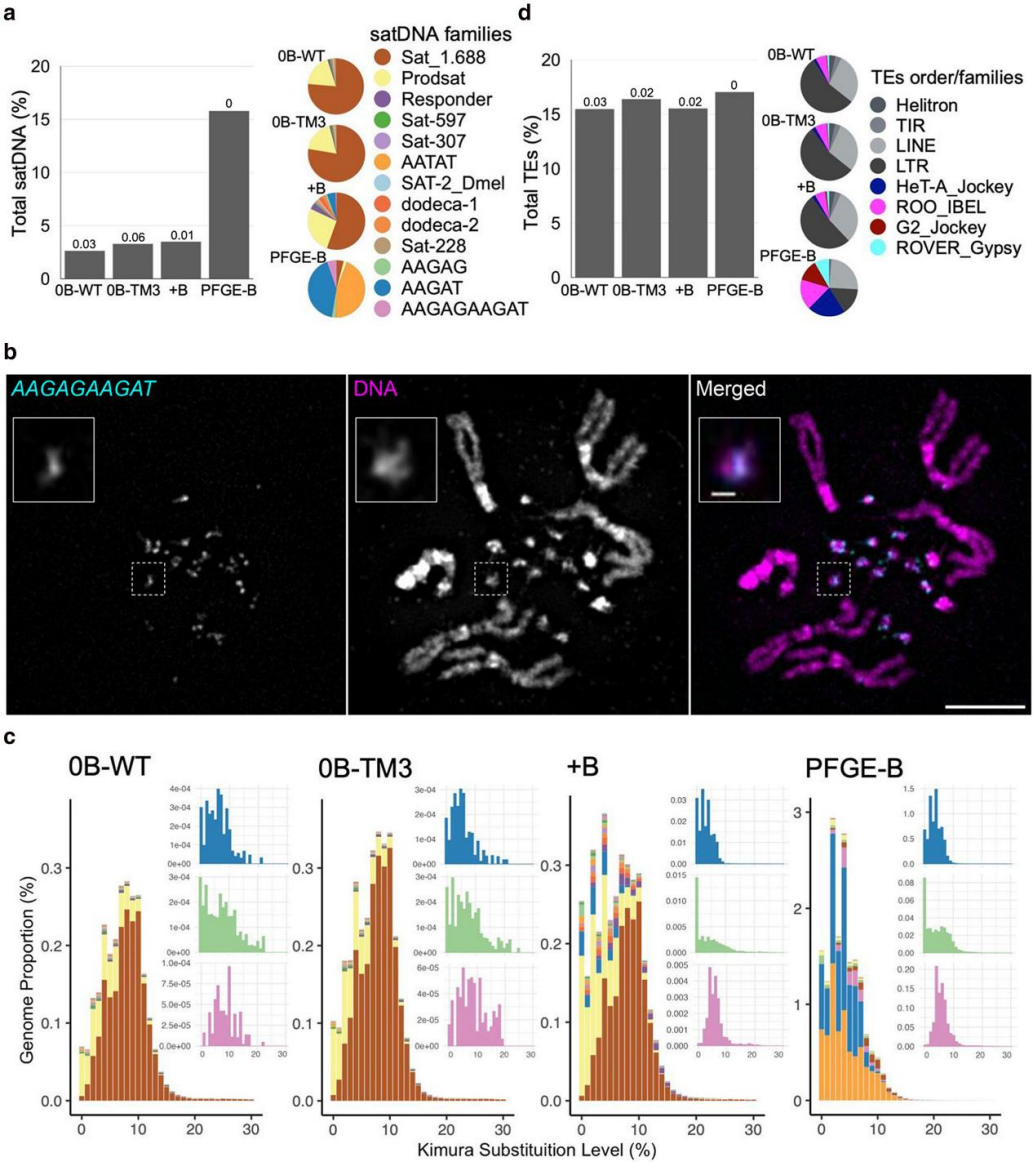
Satellite DNAs and TEs in the genome of *Drosophila melanogaster* a, b, d) genomic characteristics for the 3 genomes (WT, TM3, +B) and the sequenced pulsed field electrophoresis B (PFGE-B) and b) chromosomal mapping in mitotic chromosomes of +B stock. a) Bar plots showing the mean total genomic abundance of satDNA, expressed as percentages; standard deviation is shown above bars, indicating minimal variability among replicates. Circular plots representing the genomic abundance of each satDNA family (represented by different colors). b) FISH mapping of *AAGAGAAGAT* satDNA in a mitotic metaphase from a larval brain showing enrichment on the B chromosomes and chromosome 4. Inset: magnification of a single B chromosome. Scale bar = 5 μm (image), 0.5 μm (inset). c) SatDNA landscape (genomic abundance vs K2P divergence, both in percentage). Colors correspond to those in (a), with individual landscapes shown for *AAGAT*, *Prodsat*, and *AAGAG*. These 3 satDNAs were highlighted due to lower K2P in general divergence for +B and PFGE-B genomes when comparing with WT and TM3 genomes, that is noticed with graphical abundance peaks shifted toward lower K2P divergence for the satDNAs. d) Bar plots displaying the mean total genomic abundance of TEs, expressed in as percentages; standard deviation is shown above bars, indicating minimal variability among replicates of TEs, with circular plots depicting the proportion of TEs, either by order (in gray tones) or specific families (colored). In (a) and (d) the numbers above the bars refer to standard deviation values.

To determine the abundance of a specific genetic element relative to the rest of the genome, we quantified the number of reads that aligned to the consensus sequence using RepeatMasker and then normalized it against the total number of nucleotides in each library to obtain its relative genomic abundance. We observed differences in total satDNA abundance between our 3 stocks: 2.63% for 0B-WT, 3.28% for 0B-TM3, and 3.50% for +B (Fig. 1a). For low-complexity satDNAs (*AAGAG, AAGAT, AATAT, AAGAGAAGAT, Prodsat, dodeca-11*, and *dodeca-12*), these differences were more pronounced, with values of 0.53% for 0B-WT, 0.64% for 0B-TM3, and 1.33% for +B. A similar pattern was observed using k-seek, with abundances of 0.97% for 0B-WT, 0.64% for 0B-TM3, and 1.68% for +B. Such variation of abundance between stocks could be attributed to differences in genetic backgrounds or the prolonged inbreeding of these stocks, which may also be the cause of differences in total satDNA estimation in comparison to previous analysis (Wei et al. 2014; de Lima and Ruiz-Ruano 2022). In our study, the increased abundance of simple satDNAs appear to correlate with the presence of B chromosomes and not by technical bias in sequencing platform (Supplementary Fig. 1) or method of estimation.

To further define the abundance of these simple satDNAs between stocks with and without B chromosomes, we used RepeatMasker to estimate their enrichment: *AAGAT* (62.5× that of 0B-WT stock and 76.7× that of 0B-TM3 stock), *AAGAGAAGAT* (55.75× for both 0B-WT and 0B-TM3 stocks), *AAGAG* (14.7× for 0B-WT stock and 15.9× for 0B-TM3 stock), *dodeca-11* (8.2× for 0B-WT stock and 11.6× for 0B-TM3 stock), and *dodeca-12* (6.9× for 0B-WT stock and 9.5× for 0B-TM3 stock) (Table 1). To ensure this enrichment was independent of our method of quantification, we applied a K-mer-based analysis that was consistent with our initial analysis (Supplementary Table 3), indicating that these sequences are enriched (>5×) in the +B stock Our findings of relative genomic abundance using comparative genome analysis between stocks with and without B chromosomes is consistent with satDNA abundance trends in the PFGE-B library, especially for *AAGAT* and *AAGAG* and its tandem array *AAGAGAAGAT* (Fig. 1a, Table 1, Supplementary Table 3).

Based on our analysis of satDNA enrichment in the +B genome, we next performed FISH to determine which of these sequences are present on the B chromosome. Several of our +B-enriched satDNA sequences were only present at their known cytogenetic location, and we found 2 +B-enriched satDNAs that were already described elsewhere (*AAGAT* and *AAGAG*). The remaining satDNA repeat, *AAGAGAAGAT*, had yet to be cytogenetically characterized. Since a FISH probe for *AAGAGAAGAT* may promiscuously bind to arrays of *AAGAG* or *AAGAT*, we performed competitive inhibitor FISH using a fluorescently labeled probe recognizing *AAGAGAAGAT* combined with an excess of 2 unlabeled probes that target *AAGAG* and *AAGAT* (see Materials and methods). The unlabeled probes bind to their targets and prevent off-target binding by the labeled *AAGAGAAGAT* probe, ensuring the signal observed is from fully hybridized *AAGAGAAGAT* probe. We found that the *AAGAGAAGAT* satDNA is on both chromosome 4 and the B chromosomes at low but appreciable levels, consistent with our genomic analysis and the current model for chromosome 4 as the progenitor of the B chromosomes (Fig. 1b). When a third unlabeled probe that targets *AAGAGAAGAT* was included in our FISH, the signal for chromosome 4 and the B chromosomes was abolished (Supplementary Fig. 2).

To provide a more detailed analysis of the evolutionary dynamics of satDNA sequences, we examined their abundances and K2P divergence patterns through the generation of divergence landscapes. Repeats with peaks of abundance in lower K2P divergence are more similar and likely to have undergone more recent amplification or homogenization, whereas higher K2P divergence levels suggest older sequences that accumulated more mutations over time and are less similar (Plohl et al. 2012; Ruiz-Ruano et al. 2016). Overall, the mean divergence considering all satDNAs was slightly lower in the +B genome (6.0%) compared with 0B-WT (7.0%) and 0B-TM3 (7.1%) (Table 1). The general landscape profiles were similar across all 3 genomes, but several satDNA families showed higher abundance in lower K2P specifically in the +B genome. This pattern was particularly evident for the K2P values for 3 satDNAs: *AAGAG* (4.4% in the +B genome and 7.5 to 7.8% in the 0B genomes), *AAGAT* (3.5% in the +B genome and 5.8 to 6.0% in the 0B genomes), and the tandem arrangement *AAGAGAAGAT* (6.3% in the +B genome and 9.1 to 9.4% in 0B genomes). *Prodsat* and *AATAT* also exhibited lower divergence in the +B genome, although the differences were less pronounced. The higher abundance and lower K2P divergence values observed in the +B genome compared with both 0B genomes for certain satellite DNAs suggest that these differences are driven by the presence of the B chromosome. Divergence landscape analysis of the PFGE-B library supports this interpretation, revealing consistently low divergence values for these sequences on the B chromosome (Fig. 1c). Together, these patterns support the model that the B chromosomes arose recently and increased in copy number from a single origin since they carry relatively homogeneous copies of specific satellite DNAs that have not yet undergone substantial differentiation.

### Validation of new satDNAs in the *Drosophila melanogaster* genome

**Fig. 2.**
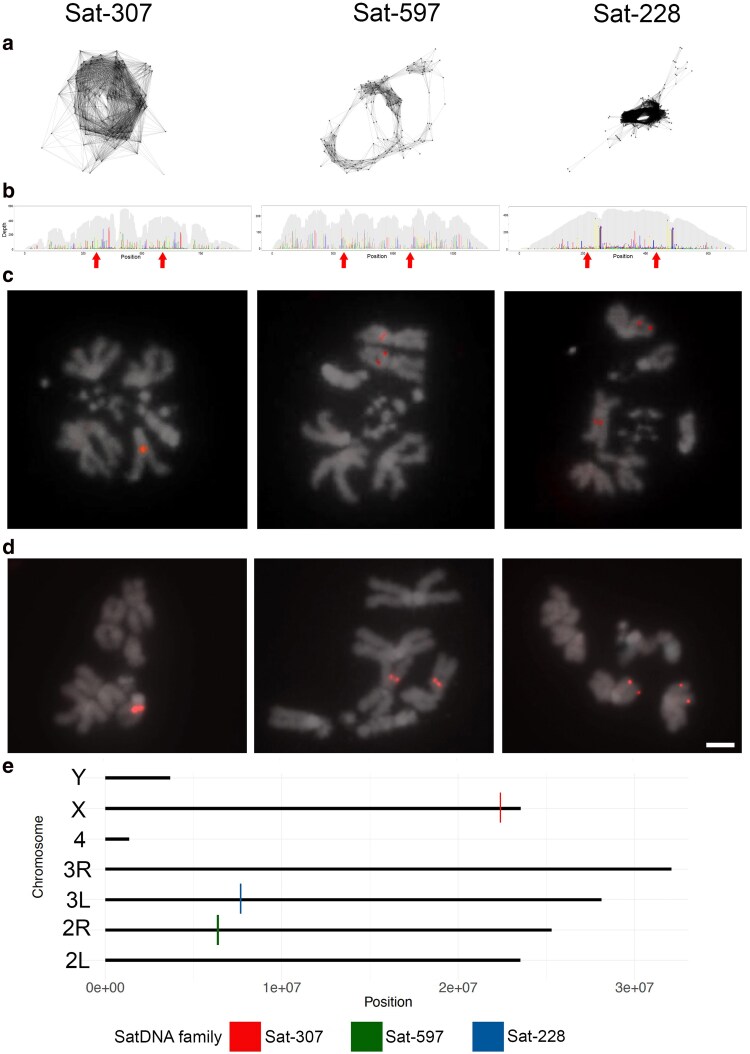
Novel satDNAs for identified from RepetExplorer analysis in the *D. melanogaster*. a) Repeat Explorer logo graph for each satDNA cluster showing circular star structure, typical for satDNAs. b) RepeatProfiler chart generated by mapping of Illumina reads from an Oregon genome in trimmer of each satDNA family. Note the presence of similar coverage in the connection of monomers (shown by the arrows) with other regions of satDNA sequences, evidencing occurrence of hybrid reads and in tandem arrangement. c) FISH mapping of the 3 satDNAs on mitotic brain cells from the +B stock and d) Oregon-R stock. The names of mapped satDNAs are labeled at the top of the panel. Scale bar = 2.5 μm e) Mapping of the 3 satDNA families obtained through the CHRISMAPP approach in genome the release 6.

Interestingly, in addition to the several known satDNA sequences, we revealed 3 previously undescribed satDNAs and named them according to their monomer length: Sat-228, Sat-307, and Sat-597 (Table 1). Several of our observations support that these sequences are true satDNAs. First, the in silico characterization using RepeatExplorer of these 3 sequences revealed a graph with a characteristic circular shape of satDNA clusters (Novák et al. 2020), indicating that the reads are connected in a way that forms tandem repeats (Fig. 2a). Second, we validated sequence coverage across monomer-monomer junctions using RepeatProfiler. The continuous coverage across the junctions is consistent with a tandem organization on these repeats (Fig. 2b). We performed this analysis using satDNAs trimmers (i.e. sequences composed of 3 tandemly repeated monomers for each satDNA) in 3 different Illumina libraries as well as the 0B libraries used in our analyses. The analyses of RepeatProfiler on these additional genomes also revealed a coverage characteristic of satDNAs, including reads spanning the junctions between monomers that further validate their tandem array organization (Supplementary Fig. 4). Additionally, we validated the presence and tandem organization of these satDNAs on multiple PacBio long reads revealing arrays with at least 3 in-tandem copies each satDNA (Supplementary Fig. 5).

Third, our chromosomal mapping with FISH revealed discrete hybridization signals, which are characteristic of clustered or tandemly repeated sequences. We performed FISH on mitotic chromosomes from the +B stock and on Oregon-R as it represents a distinct genetic background compared with the +B stock and serves to independently confirm the presence of these satDNAs (Fig. 2, c and d). This analysis revealed that Sat-307 is located near the pericentromeric region of the X chromosome, Sat-597 is in the interstitial region of chromosome 2, and Sat-228 is in the interstitial region of chromosome 3. For further validation, we mapped these new satDNAs to the assembled genome (Release 6.58) using CHRISMAPP analysis (for details, see Materials and methods), which showed consistent localization patterns with our FISH results (Fig. 2e).

Interestingly, for Sat-228, the BLAST analysis identified sequence similarity to the protein-coding gene *Ankyrin* 2 (*Ank2*—FlyBase ID: FBgn0261788). To further investigate the presence of this satellite within *Ank2*, we mapped Sat-228 to the *Ank2* open reading frame and identified 94 monomeric repetitions of the satDNA spanning approximately 22 Kbp. Among the 22 annotated *Ank2* transcript isoforms, 18 include this stretch of Sat-228 in their pre-mRNA, however only 2—*Ank2*-RU (FlyBase ID: FBtr0303125) and *Ank2*-RAA (FlyBase ID: FBtr0344224)—retain Sat-228 after splicing in their processed mRNA (Supplementary Fig. 6).

At the protein level, *Ank2* is characterized by a conserved tandem repeat motif of approximately 33 amino acids across taxa (Bork 1993; Goto et al. 2008; Jegla et al. 2016). The Sat-228 did not show homology in BLAST searches outside *Drosophila*, nor did it align with the conserved tandem repeat motifs within the *Drosophila Ank2* polypeptide sequence. Specifically, in the polypeptides derived from the satellite-containing transcripts (*Ank2*-PAA: FBpp0310630, FBtr0344224; and *Ank2*-PU: FBpp0292244, FBtr0303125), the characteristic tandem amino acid motifs were present as observed in all other 20 isoforms (Supplementary Fig. 7). It is important to note that although the 2 satellite-containing isoforms of *Ank2* were identified through mRNA prediction, currently they still lack in vivo validation (Matthews et al. 2015). Given that satDNA is traditionally classified as a noncoding tandem repeat (Garrido-Ramos 2017), we assume that Sat-228 represents a satDNA sequence integrated within a protein-coding region.

### The abundance of transposable elements and multigene families is similar between the 0B and +B genomes

The comparative TE landscapes of the 0B and +B genomes present a different scenario in comparison to satDNAs. The overall TE genomic abundance of 0B-WT, 0B-TM3, and +B were 15.46%, 16.38%, and 15.52%, respectively (Table 2, Supplementary Table 4, Fig. 1d). In line with this, differences in genome abundance for specific TEs were also minor, varying only from 0.29× to 2.1× the B+/0B-TM3 comparison, and from 0.43× to 2.6× in the B+/0B-WT comparison. The only TE modestly enriched (>2× over the 0B samples) in both comparisons was *G2/Jockey*. This result implies that besides the slight differences in total TE content across libraries, no specific families showed enrichment in the +B genome. The quantification of the specific TE repeats in the PFGE-B revealed a slight increase in genomic abundance of a few elements, mainly the *HeT-A* (3.6%), the *Bel_Roo_I* (2.9%), the *G2/Jockey* (2.04%), and the *Rover_Gypsy* (1.43%). These specific elements are highlighted in Fig. 1d. Other TEs represented 1% or less of the PFGE-B (Supplementary Table 4). Finally, we estimated the abundance of a handful of multigene families as they are frequently observed in the B chromosome of distinct species. Analysis of 45S and 5S rDNA subunits, 6 spliceosomal genes, and 5 histone genes (Ahmad and Martins 2019; Oliveira et al. 2024) revealed similar small differences in the genomic abundance between +B and 0B stocks. We confirmed these observations with the PFGE-B library, in which multigene families represent only 0.25% of its content (Supplementary Table 5). Together, the abundance of TE and multigene families do not appear to be significantly different in the stock that has harbored B chromosomes for nearly 25 yr.

**Table 2.** Genomic characteristics for the TEs analyzed in the distinct genomes and the sequenced pulsed field electrophoresis B (PFGE -B).

	0B-WT		0B-TM3		B+		PFGE-B		B+/0B-WT	B+/0B-TM3
TE class	Genome proportion (%)	Divergence (%)	Genome proportion (%)	Divergence (%)	Genome proportion (%)	Divergence (%)	Genome proportion (%)	Divergence (%)		
*Helitron*	0.5536	8.6	0.5486	8.5	0.5138	8.5	0.1113	8.3	0.9	0.9
TIR	0.5372	5.5	0.5492	5.4	0.5448	5.0	0.1227	4.8	1.0	1.0
LINE	4.7138	5.8	5.1521	5.8	5.2551	5.8	9.8555	5.8	1.1	1.0
LTR	9.6608	6.1	10.1354	5.9	9.2068	5.9	6.9528	5.6	1.0	0.9
**Total abundance**	**15.4655**		**16.3853**		**15.5206**		**17.0422**		1.0	0.9
**Divergence average**		**6.5**		**6.4**		**6.3**		**6.2**		

It is shown for each of the 4 TE classes the genome abundance (%) and divergence (K2P %). The last 2 lines show the total abundance and the average divergence for all the TEs analyzed.

### Epigenetic features of the B chromosome

**Fig. 3.**
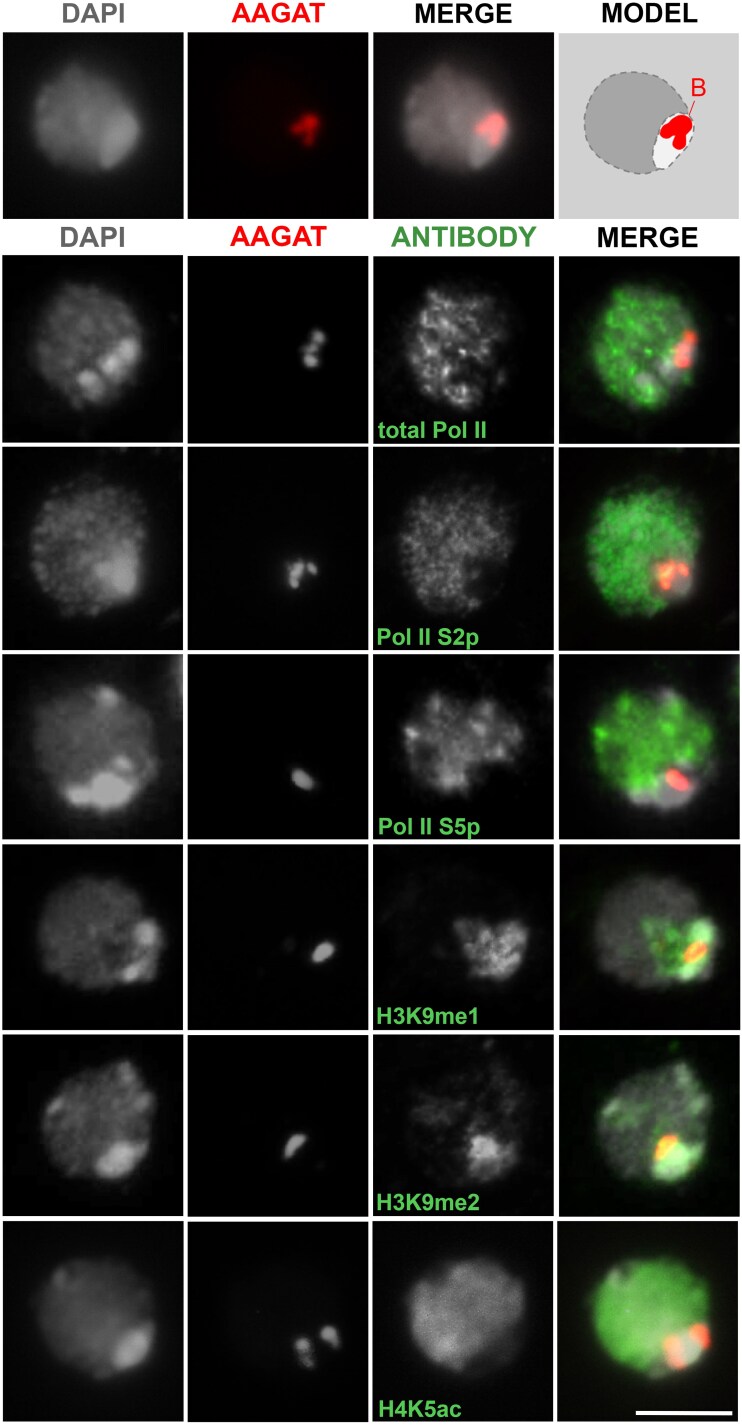
Transcriptional activity and histone epigenetic modifications together with B chromosome territory identification in male brain somatic interphase cell of *D. melanogaster* + B stock. The first panel shows the FISH mapping of the probe *AAGAT*, which allows the identification mainly of the B chromosome territory depicted as a cartoon in the top right corner. Subsequent panels show the immunostaining with specific antibodies, each directly indicated in the images. In the merged images, the antibody signal is colored in green and the B chromosome territory is shown in red. Scale bar = 2.5 μm.

**Fig. 4.**
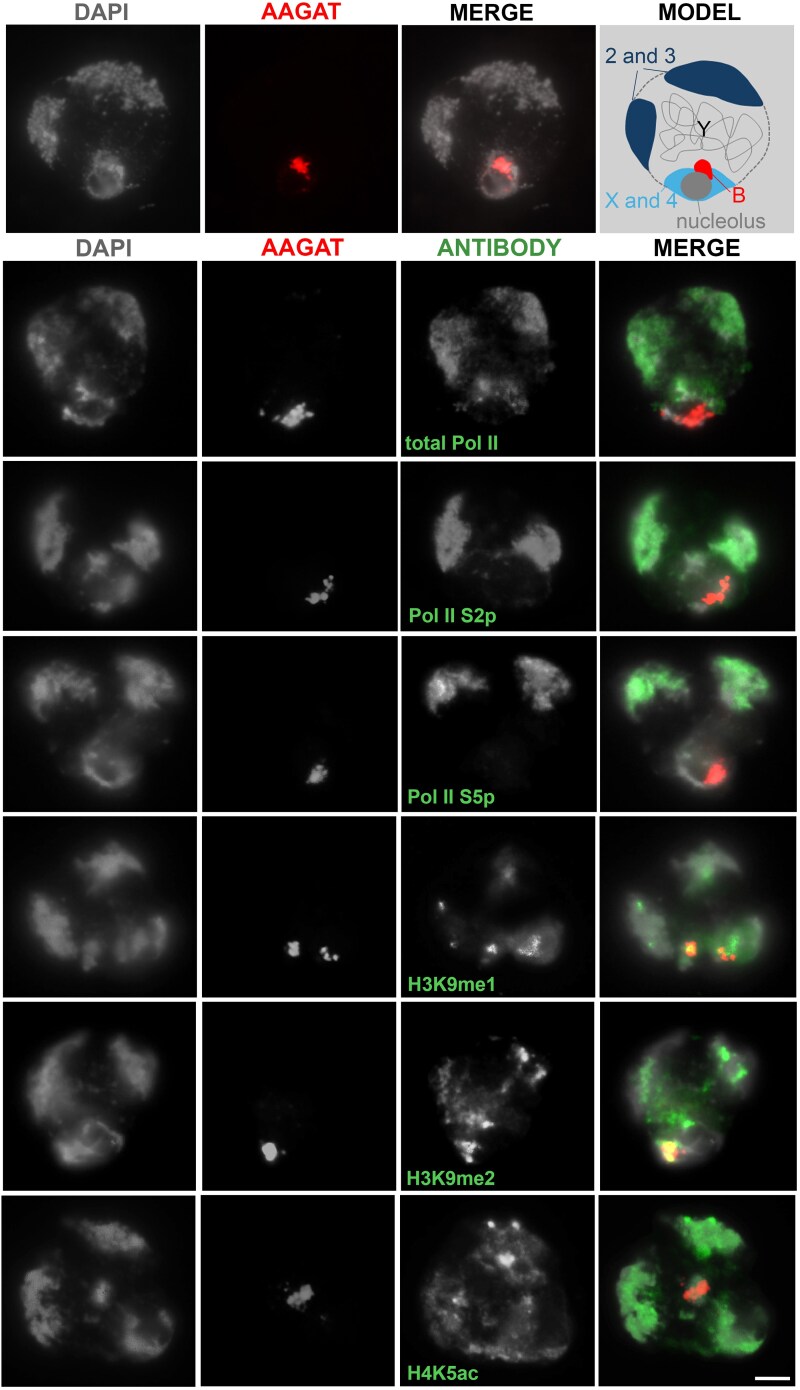
Transcriptional activity and histone epigenetic modifications together with B chromosome territory identification in late primary spermatocytes male germline cell of *D. melanogaster* +B stock. The first panel shows the FISH mapping of the probe *AAGAT*, which allows the identification mainly of the B chromosome territory depicted as a cartoon in the top right corner. The territories of other chromosomes were identified from Mahadevaraju et al. (2021). Subsequent panels show the immunostaining with specific antibodies, each directly indicated in the images. In the merged images, the antibody signal is shown in green, while the B chromosome territory is in red. Scale bar = 5 μm.

Our bioinformatic analysis indicates that B chromosomes have yet to deviate significantly in sequence from each other and from their chromosome of origin, chromosome 4. As expected from this observation, the B chromosomes consistently localize alongside the chromosome 4 near the DAPI-bright chromocenter in somatic interphase cells, as well as the chromosome 4 territories in primary spermatocytes within the male germline (Supplementary Fig. 3). We were next curious if the epigenetic features of the B chromosomes are similar to chromosome 4, specifically the heterochromatic left arm of chromosome 4 that gave rise to the initial B chromosome. To assess overall transcriptional activity of the B chromosome and the heterochromatic portion of chromosome 4, we performed immunolocalization of RNA polymerase II (Pol-II), including both total Pol-II and its phosphorylated active forms, and compared it to chromosome 4 and B chromosome localization in brain somatic cells and male germ cells. Specifically, we analyzed Serine 5 phosphorylation of Pol-II (associated with transcription initiation) and Serine 2 phosphorylation (associated with elongation) and used *AAGAT* satDNA FISH to identify the territory with both the B chromosomes and chromosome 4 heterochromatin (B+ 4het). In both somatic (interphase) and germ cells (primary spermatocytes), the B chromosomes and chromosome 4 heterochromatin consistently occupied a territory devoid of RNA polymerase II, irrespective of its activation state (Figs. 3 and 4). 3D imaging analysis further confirmed this observation: the green fluorescence signal from Pol-II antibodies did not overlap with the red FISH signal from the B+ 4het chromosome territory but was present throughout the DAPI-dim regions that are associated with euchromatin (Supplementary Videos 1–12).

To investigate the association between histone modifications and the reduced presence of RNA polymerase II in the B+ 4het chromosome territory, we conducted immunolocalization analyses of methylated histones using H3K9me1 and H3K9me2 antibodies. The results showed that the B+ 4het chromosome territory is hypermethylated relative to other chromosomal regions that displayed weaker signals. Conversely, immunolocalization with an antibody for acetylation that is associated with chromatin transcription, the H4K5ac antibody, revealed no labeling of the B+ 4het chromosome territory, while other regions of the nucleus were prominently labeled. These patterns were consistent in both somatic and germline cells (Figs. 3 and 4) and were also observed in the 3D imaging analysis. In the 3D imaging, the green signals for methylation were associated with the B+ 4het chromosome's red FISH signal, whereas for acetylation, the overlap is predominantly seen in the DAPI-dim regions (Supplementary Videos 1–12). Our analysis indicates that the B chromosomes still retain the nuclear localization, transcriptional activity, and epigenetic signatures of the heterochromatic location on chromosome 4 they likely arose from.

## Discussion

### B chromosomes as repositories for specific satDNA sequences

Our study provides novel insights into the composition, differential abundance of distinct repetitive DNA classes, and epigenetic landscape of the B chromosome in *Drosophila melanogaster*. Moreover, the identification of 3 previously uncharacterized sequences (Sat-307, Sat-597, and Sat-228) found exclusively on the A chromosomes provides a more complete picture of the repetitive DNA landscape in *D. melanogaster*. The enrichment of specific satDNAs (*AAGAT*, *AAGAG*, and *AAGAGAAGAT*) in +B genomes, further validated by FISH in this work or previously (Hanlon et al. 2018) suggests that the satDNAs are the main drivers of B chromosome composition of *D. melanogaster*. This observation aligns with previous findings in other species where B chromosomes preferentially accumulate repetitive DNA, potentially influencing its genome organization and function (Ahmad and Martins 2019; Camacho et al. 2021; Oliveira et al. 2024). In the case of *D. melanogaster*, satDNAs enriched in the B chromosome are also found in other chromosomes, such as 2, 3, 4, and Y. The fact that chromosome 4 harbors all of them, with some repeats being exclusively shared between chromosomes 4 and B, most parsimoniously supports previous hypothesis about the origin of the B chromosome from chromosome 4 (Hanlon et al. 2018). Notably, the +B genome exhibits reduced sequence divergence for satDNAs enriched on the B chromosome, as evidenced by lower K2P values. This reduced divergence suggests that these sequences are relatively homogeneous, which is consistent with the idea that these satDNAs have not yet undergone substantial differentiation since the emergence of the B chromosomes (<25 yr). SatDNAs are known for playing a role in *Drosophila* centromere architecture and maintaining proper chromosome segregation during cell division (Jagannathan et al. 2018; Talbert et al. 2018; Chang et al. 2019; Courret et al. 2024). Misregulation of their expression is found to lead to various defects in the maintenance of genomic architecture, chromosome segregation and gametogenesis (Jagannathan et al. 2018; Mills et al. 2019; Wei et al. 2021). In this way, the presence and maintenance of satDNAs in the B chromosome of *D. melanogaster* could favor the stabilization of the centromere structure.

The overall genomic abundance of TEs and multigene families remained largely unchanged between 0B and +B genomes, implying that the presence of the B chromosome does not broadly affect genome-wide repeat dynamics. It reinforces the evidence that the satDNAs—but not the TEs or multigene families—are the main component of these B chromosomes. However, the specific enrichment of *G2/Jockey* in the PFGE-B library suggests that the B chromosome may selectively retain or amplify certain elements contributing to its maintenance, especially for centromeric sequences, favoring its own maintenance. The fact that satellites are enriched but not TEs might also imply that these elements have different dynamics on B chromosome stock of *D. melanogaster*: due to its high mutation rate (fueled by gene conversion, nonhomologous and sister chromatid recombination), satDNAs may be able to experience changes in genomic abundance in shorter timescales than TEs. Indeed, the *de-novo* formation of these B chromosomes represents an instance of experimental evolution.

### Novel satDNAs for *D. melanogaster*

The discovery of Sat-307, Sat-597, and Sat-228 provides an important addition to the catalog of satDNAs in *D. melanogaster*. The validation of these sequences across distinct genetic backgrounds suggests their conservation, although it would be interesting to study their evolution in a broader sample and also at a different evolutionary timescale by studies in sister species. The confirmation of their tandem repeat structure through RepeatProfiler, CHRISMAPP, Pacbio reads along with cytological validation via FISH, strongly supports their classification as authentic satDNAs. Although the genomic abundance is low for the 3 satDNAs, it is important to notice that they are more abundant than other satDNAs previously described. The genome of *D. melanogaster* is enriched with satDNAs that are 7 bp or fewer in length (Lohe et al. 1993; reviewed in Lauria Sneideman and Meller 2021), which contrasts with the 3 repeats discovered here. These 3 are among the top in monomer length, with Sat-597 being the largest described in the *D. melanogaster* genome. Moreover, our FISH analysis demonstrates that these elements occupy discrete areas in only one chromosome. Their localization either outside of or adjacent to the prominent heterochromatin blocks, with 1 instance (Sat-228) located within a gene, aligns with previous reports of other euchromatic satDNAs in *Drosophila* (Kuhn et al. 2012; Gallach 2014; Sproul et al. 2020). Given the potential for tandem repeats to influence the expression of nearby genes (Rockman and Wray 2002; Gemayel et al. 2010; Feliciello et al. 2015; Deshpande and Meller 2018; Kim et al. 2019), these 3 novel satDNAs could serve as valuable models for studying how tandem repeats modulate gene expression. More functional analyses will be essential to determine whether these sequences contribute to transcriptional regulation of nearby genes.

### Evidence for the reduced B chromosome transcriptional activity

Our results provide compelling evidence that the B chromosome in *D. melanogaster* has reduced transcriptional activity as compared to regions of euchromatin in both somatic and germline male cells. The absence of total and active forms of RNA polymerase II in the B chromosome nuclear territory is likely due to its heterochromatic state that is enriched for repressive epigenetic marks like H3K9 methylation and deficient for marks that indicate active transcription like H4K5 acetylation. Three-dimensional imaging further confirms the unique spatial organization of the B chromosome within the nucleus. The strong colocalization of methylation signals with the B chromosome, combined with the confinement of acetylation marks to other DAPI-stained regions, indicates that the B chromosome is embedded within a repressive chromatin environment. Having this initial nuclear localization as a newly formed B chromosome may make it easier for it to be maintained for longer since a transcriptionally quiescent B chromosome may minimize its impact on host genome function. (Camacho et al. 2000; Santos et al. 2024).

In both somatic and germline cells, we found that the B chromosomes of *D. melanogaster* display a stable epigenetic pattern. Similar to what we observed here, the B1 variant of the repeat-enriched B chromosome in the grasshopper *E. plorans* (Cabrero et al. 1999) displayed the absence of RNA polymerase II and the presence of epigenetic marks associated with transcriptional repression during spermatogenesis (Cabrero et al. 2007; Santos et al. 2024). Not all B chromosomes remain in a single epigenetic state; some B chromosomes have been found to shift their transcriptional and epigenetic states (or modulate those of the host genome) to enhance their transmission during sexual reproduction. In the pseudococcid *Pseudococcus viburni* Vea et al. (2025) showed that the B chromosome is highly heterochromatic, exhibiting heterochromatin-associated histone modifications (H3K9me3, H3K27me3, and H4K20me3). However, as meiosis progresses, the B chromosome transitions to a less compact state and acquires euchromatin-associated modifications such as H4K16ac, H3K27ac and H3K18ac. This shift remodels its chromatin landscape to resemble the maternal complement and ultimately enables to segregate with the maternal chromosomes, ensuring its transmission to progeny since chromosomes with paternal epigenetic marks are eliminated (Herbette and Ross 2025; Vea et al. 2025). In the jewel wasp *Nasonia vitripennis*, the PSR B chromosome, which is ∼89.8% repetitive (Benetta et al. 2020), has H3K9me2/3 but lacks the 2 marks present on the paternally derived genome (H3K27me1 and H4K20me1). During the first embryonic mitosis, the paternal genome is eliminated and the haploid embryo develops into a male, enabling PSR to reliably achieve 100% transmission (Aldrich and Ferree 2017). Interestingly, the transcriptional repression of the B chromosome mirrors the epigenetic landscape of chromosome 4 heterochromatin from which it is derived. The heterochromatic left arm of chromosome 4 is enriched in repetitive DNA and exhibits hallmark repressive marks such as H3K9me2 and H3K9me3 and a lack of RNA polymerase (Yasuhara and Wakimoto 2008; Riddle et al. 2011; Mahadevaraju et al. 2021). Notably, chromosome 4 represents the ancestral X chromosome in *Drosophila* (Vicoso and Bachtrog 2013) and occupies the same nuclear territory as the X chromosome in spermatocytes. In this territory, it is also found to be depleted of active RNA polymerase, which correlates with markedly reduced transcriptional activity as a consequence of meiotic X chromosome inactivation (Mahadevaraju et al. 2021). This suggests that both chromosomal elements are subject to strong epigenetic regulation, reinforcing the idea that the B chromosome has largely retained the heterochromatic nature of its ancestral genomic region.

Our study has provided new insights into the composition, evolution, and epigenetic regulation of the B chromosome of *D. melanogaster*, along with discovering 3 new satDNAs. The enrichment and low divergence of specific satDNAs found on the B chromosomes suggest that the composition of the B chromosome is governed by few families of satDNAs. Additionally, the lack of significant active transcription and its repressive heterochromatic state may be a mechanism for how new B chromosomes minimize disruption to essential genomic functions to allow them to persist in a population for several generations. Future research into the functional impact of presence of B chromosomes on the transcription of sequences from standard chromosomes would begin to elucidate the broader significance of B chromosomes in genome biology and lead to a comprehensive understanding of the interplay between repetitive DNA, epigenetics, and chromosomal evolution.

## Supplementary Material

iyag113_Supplementary_Data

iyag113_Peer_Review_History

## Data Availability

Illumina sequencing reads generated in this report are available at the National Center for Biotechnology Information SRA (http://www.ncbi.nlm.nih.gov/sra) under project PRJNA484270. All stocks and reagents available upon request. Supplemental material available at GENETICS online.
