# Supplementary material for: Comparative genomic analysis and epigenetic profiling of the Drosophila melanogaster B chromosome
Source: Genetics. 2026 May 5;233(4):iyag113. doi: 10.1093/genetics/iyag113 (PMC13439713; doi:10.1093/genetics/iyag113)
Supplement: Supplementary file 1 [file iyag113_peer_review_history.pdf]

GENETICS

# Comparative genomic analysis and epigenetic profiling of the Drosophila melanogaster B chromosome

Diogo Cabral-de-Mello, Ana Beatriz Ferretti, Diogo Milani, Emiliano Martí, Rhavenna Alves-Gomes, Maria Vibranovski, and Stacey Hanlon

**NOTE:** The reviews and decision letters are unedited and appear as submitted by the reviewers.
In extremely rare instances and as determined by a Senior Editor or the EIC, portions of a review may be redacted. If a review is signed, the reviewer has agreed to no longer remain anonymous.
The review history appears in chronological order.

**Review Timeline:**

| | |
|---|---|
| Submission Date: | 2025-10-01 |
| Editorial Decision: | 2025-11-03 |
| Resubmission Received: | 2026-03-26 |
| Accepted: | 2026-04-22 |

1st Editorial Decision: November 3, 2025

November 2, 2025

GENETICS-2025-308652
Comparative genomic analysis and epigenetic profiling of the Drosophila melanogaster B chromosome

Dear Dr. Cabral-de-Mello:

Three experts in the field have reviewed your manuscript, and I have read it as well. I concur with their assessments that the manuscript requires further improvement before it can be considered for publication in GENETICS.

While your manuscript is not currently acceptable for publication in GENETICS, we would welcome a substantially revised manuscript. All reviewers have comments and concerns to be addressed in a revised manuscript. You can read their reviews at the end of this email.

In particular, please pay close attention to the reviewers' comments regarding the quantification of simple satellites, as well as the claims about B chromosome silencing. I think it does require more specific methods to quantify simple repeats using short read data. Additionally, I agree that, given our current understanding and the cytological evidence presented, the statement that B chromosomes are silenced in this and other systems is too strong and should be tempered accordingly.

We look forward to receiving your revised manuscript. Please let the editorial office know approximately how long you expect to need for revisions.

Upon resubmission, please include:
1. A clean version of your manuscript;
2. A marked version of your manuscript in which you highlight significant revisions carried out in response to the major points raised by the editor/reviewers (track changes is acceptable if preferred);
3. A detailed response to the editor's/reviewers' feedback and to the concerns listed above. Please reference line numbers in this response to aid the editor and reviewers.

Your paper will likely be sent back out for review.

Additionally, please ensure that your resubmission is formatted for GENETICS
https://academic.oup.com/genetics/pages/general-instructions

Follow this link to submit the revised manuscript: Link Not Available

Sincerely,

Ching-Ho Chang
Associate Editor
GENETICS

Approved by:
Kelly Dyer
Senior Editor
GENETICS

------------------------------------------------------------------------

Reviewer #1 :

The paper investigates the genetic and epigenetic composition of Drosophila melanogaster B chromosomes (Bs), which are supernumerary elements found in the genomes of many species across diverse taxa. Using cytogenetic mapping (FISH), immunostaining, and comparative genomics, the authors identify nine satellite DNA (satDNA) sequences enriched in flies carrying B chromosomes and discover three new satDNAs (Sat-228, Sat-307, Sat-597) located on the standard chromosome set.
The B chromosomes are shown to be transcriptionally silent, exhibiting heterochromatin-associated histone marks (H3K9me1, H3K9me2), a lack of acetylation (H4K5ac), and the absence of RNA polymerase II activity. The results support the conclusion that these Bs originated recently from chromosome 4 and remain heterochromatic and epigenetically repressed. Interestingly, these B chromosomes appear to be a laboratory phenomenon, likely the result of laboratory evolution. The low level of satDNA

divergence is consistent with their recent origin.
This study provides valuable molecular and cytological characterisation of D. melanogaster Bs, expanding our understanding of their repetitive DNA composition and silencing mechanisms. The integration of bioinformatics with cytogenetic validation strengthens the conclusions, and the discovery of new satDNAs adds further significance to the research.
Minor Comments and Suggestions
• The first Abstract sentence appears to generalize the characteristics of B chromosomes based on a limited number of well-characterized cases. While earlier studies described Bs as largely composed of repetitive DNA and transcriptionally inactive, more recent evidence indicates that transcriptional activity (Navarro-Domínguez et al. 2017; Dhar et al. 2019; Rajpal et al. 2023) occurs in certain taxa. This may depend on their "evolutionary age," but inactivity is certainly not a universal feature. As Abstracts are often cited in subsequent publications, it would be advisable to moderate this claim, particularly considering that fewer than 1% of known B-chromosome-bearing species have been studied in sufficient molecular detail to support such generalisations.
• While the study infers a recent origin of Bs, it would be informative, if such data are available, to include the frequency of B+ individuals (both males and females) within the B+ stock.
• Lines 321-330 - The reported values (2.63% for 0B-WT, 3.28% for 0B-TM3, and 3.50% for +B stock) show greater differences between the 0B stocks than between 0B-TM3 and B+. Could the authors clarify what might cause such variation among the B0 stocks?
• Table 1: Please round the value 55.75 to 55.8 in the last two columns for consistency.

Reviewer #2 :

This manuscript characterizes the sequence composition and epigenetic state of supernumerary B chromosomes in Drosophila melanogaster. The authors compare short-read genomic libraries from +B and 0B strains, identify satellite DNAs (including three novel repeats), quantify transposable elements, map several satellites cytogenetically, and use IF/FISH to demonstrate that the B chromosomes are heterochromatic and depleted of RNA polymerase II. The study provides a valuable resource and establishes a baseline model for investigating satellite DNA dynamics and B chromosome biology. Beyond its relevance for understanding B chromosome evolution and maintenance, this system also offers an excellent opportunity to explore how additional heterochromatic domains influence overall chromatin organization and genome regulation.

Overall, the manuscript is clearly written and well organized. I have one major concern regarding the quantification of low-complexity satellites, along with several minor comments listed below.

Major comments

1. The +B library was sequenced on HiSeq while the two 0B libraries were on NextSeq. Different platforms, run conditions, and library preps can introduce platform-specific biases especially regarding low-complexity sequence (such as short simple repeats). This is particularly worrying because the core claims about enrichment of specific satellites in +B are quantitative (fold-enrichments). This could explain why the authors report nine satellites enriched in +B genomes but cytogenetically confirm only three on the B. At least provide explicit controls to rule out/quantify batch effects, like a PCA or hierarchical clustering on satellite abundance (all satellites and A-linked satellite) to check whether samples cluster by genotype (+B vs 0B) versus by sequencing run/platform. If there is indeed a batch bias, an orthogonal approach might be necessary to confirm the quantitative difference for the most abundant satellite candidate.

2. The authors claim that the AAGAGAAGAT probe is specific and does not cross-hybridize to AAGAG or AATAT, based on high stringency hybridization, but no co-hybridization or direct control images are provided. Because AAGAGAAGAT is composed of AAGAG + AAGAT subunits, cross-hybridization is plausible. Please provide FISH co-hybridization experiments showing AAGAGAAGAT together with AAGAG and AAGAT probes to demonstrate non-overlap/overlap patterns. Provide the hybridization temperature and buffer stringency conditions used for each probe. State whether the same conditions were used across probes.

3. The methods used for repeat identification and quantification do not appear optimal for low-complexity satellites (such as AAGAG). First, the authors use SatMiner (based on RepeatExplorer) to identify satellite sequences in each library. While this pipeline performs well for complex and longer satellite arrays, it probably to underestimate or filter out short, low-complexity repeats, such as those composed of 5-7 bp monomers. Second, for quantification, the authors apply RepeatMasker using a library of consensus sequences, where simple satellites are represented by single monomer units, and subsequently estimate genomic abundance and Kimura (K2P) divergence. It is unclear how reads were mapped to such very short consensus sequences.
The authors should specify: How reads aligning to 5-6 bp consensus sequences were handled (e.g., minimum alignment length, mismatch tolerance, multi-mapping strategy); How K2P divergence was calculated for such short motifs, given that this metric is not typically robust for monomeric repeats.
Overall, this analytical framework is widely used for transposable elements and complex satellites, but it is not well suited for simple satellites, where k-mer-based approaches are more appropriate.
Moreover, the abundance estimates reported here are unexpectedly low. The total satellite content is estimated at only ~3% of

the genome, whereas previous studies report ~20% for D. melanogaster. Similarly, the AAGAG satellite-one of the most abundant repeats in D. melanogaster-is reported at just 0.0028%, compared to prior estimates around 5%. Such discrepancies strongly suggest that the current approach underestimates simple satellite abundance.
The authors should:
- Compare their estimates to previous genome-wide quantifications and explicitly discuss potential sources of discrepancy (e.g., platform bias, filtering thresholds, pipeline limitations).
- Reanalyze simple satellites using tools specifically designed for short, low-complexity repeats, such as k-seek (Wei et al. 2018) or other k-mer-based quantification methods, to validate and refine their results.

4. I would like to see a clearer and more explicit comparison between the genetic composition and epigenetic state of the D. melanogaster B chromosome and those described in other species where B chromosomes have been extensively studied (e.g., grasshoppers, wasps, or beetles). Such a comparison would help place the findings in a broader evolutionary context and highlight whether the D. melanogaster B shares common features-such as repeat composition, gene content, or transcriptional silencing mechanisms-or represents a distinct type of B chromosome.

Minors comments
- Page 4, line 109: Reference missing for Hanlon et al. 2018 - please add the citation.
- Page 4, line 114: Please cite Jagannathan et al. 2018 when discussing the role of satellite DNA in centromere function.
- Page 7, line 189: The manuscript states that a coverage plot (RepeatProfiler) was generated to validate the tandem organization of the novel satellites. Please include this coverage plot as a Supplementary Figure.
- Page 13 / Figure 1a: Materials indicate three replicate libraries per genotype. Show replicate variation in Figure 1a and add error bars.
- Do the authors find the three novel satellites (Sat-228, Sat-307, Sat-597) only in D. melanogaster, or are they present in other Drosophila species?
- Page 20, line 507: Incorrect citation year for Courret et al. - should be Courret et al. 2024 (not 2019).

Reviewer #3 :

The authors analysed the repeat composition of 0B and +B Drosophila melanogaster and performed immuno/FISH experiments to decipher the general chromatin composition of the B chromosome. A similar chromatin composition was found for chromosome 4 and the chromosome 4-derived B chromosome.
The results are of interest, but more care should be taken in interpreting the immunostaining results (see line 461 and others). The conclusion that chromosome 4 and the Bs are transcriptionally silent is likely too strong. Because it has been shown that chromosome 4, although mostly heterochromatic, contains ~80-100 protein-coding genes. These are expressed during development. See: 3 Genes|Genomes|Genetics https://doi.org/10.1093/g3journal/jkac019.

Whether the same is true for the B chromosome of Drosophila is unknown.
I suggest talking about low transcription activity rather than no activity. Alternatively, a comparative RNAseq analysis of 0B and +B specimens would be the best way to address this question. In the future, ChIP-seq with antibodies recognising eu- or heterochromatin-specific histone marks should be considered for a detailed analysis of the B chromosome's chromatin composition.

Additional points:

ABSTRACT:
line 35-36
The statement that Bs are generally silenced is not correct any more. Recent literature has described many B chromosome-encoded active genes across diverse species, and it has been shown that the B chromosome can influence, in trans, the transcriptome of the standard A chromosomes. Thus, tone down this general statement.

Introduction
Line 73
A non-disjunction event alone will not result in a B chromosome. It may be that non-disjunction triggered chromosome rearrangements of micronucleated chromosomes. Please rephrase the sentence.

Line 87
Do you mean that B chromosomes do not recombine with a B chromosome homolog, or do you refer to A chromosomes?

Discussion
Line 583 - 594
To improve the study's impact, I suggest comparing the findings from Drosophila B with those of other species regarding their chromatin composition and transcriptional activity.

**The point-by-point responses to our manuscript (GENETICS-2025-308652) are below. Original comments/feedback are in black, our responses are in blue, and references to the location of the revisions in the manuscript are in red (CLEAN manuscript version).**

**#Editor comments**

In particular, please pay close attention to the reviewers' comments regarding the quantification of simple satellites, as well as the claims about B chromosome silencing. I think it does require more specific methods to quantify simple repeats using short read data. Additionally, I agree that, given our current understanding and the cytological evidence presented, the statement that B chromosomes are silenced in this and other systems is too strong and should be tempered accordingly.

**AUTHORS:** Thank you for the opportunity to revise the manuscript based on the insightful comments from the reviewers. We addressed all concerns and incorporated the suggested modifications in the text, as detailed below. As recommended, we paid close attention to (1) the quantification of simple satellites and (2) the claims regarding B-chromosome silencing. For point (1), we performed the K-mer-based analysis based on Wei et al. (2014; 2018). The resulting values differed from our previous estimates (showing higher quantities), but they also differ from those reported in other studies. We checked a few additional studies, and the quantification of these same repeats varies considerably across different libraries and publications, suggesting substantial variability among *D. melanogaster* stocks. Since our primary focus is the comparison between 0B and +B genomes, we refrained from drawing strong conclusions about absolute quantities and instead emphasized the relative enrichment of certain repeats in the B chromosome. Importantly, both our previous and current estimates consistently show that some repeats are enriched in the +B genome, regardless of the quantification method. For point (2), we softened some conclusions since our cytological evidence cannot rule out that part of the B chromosome may escape silencing. We believe that the revisions have substantially improved the quality and rigor of the manuscript and hope that it is now suitable for publication.

**Reviewer #1:**

The paper investigates the genetic and epigenetic composition of Drosophila melanogaster B chromosomes (Bs), which are supernumerary elements found in the genomes of many species across diverse taxa. Using cytogenetic mapping (FISH), immunostaining, and comparative genomics, the authors identify nine satellite DNA (satDNA) sequences enriched in flies carrying B chromosomes and discover three new satDNAs (Sat-228, Sat-307, Sat-597) located on the standard chromosome set.

The B chromosomes are shown to be transcriptionally silent, exhibiting heterochromatin-associated histone marks (H3K9me1, H3K9me2), a lack of acetylation (H4K5ac), and the absence of RNA polymerase II activity. The results support the conclusion that these Bs originated recently from chromosome 4 and remain heterochromatic and epigenetically repressed. Interestingly, these B chromosomes appear to be a laboratory phenomenon, likely the result of laboratory evolution. The low

level of satDNA divergence is consistent with their recent origin.
This study provides valuable molecular and cytological characterisation of D. melanogaster Bs, expanding our understanding of their repetitive DNA composition and silencing mechanisms. The integration of bioinformatics with cytogenetic validation strengthens the conclusions, and the discovery of new satDNAs adds further significance to the research.
**AUTHORS:** Thank you for the positive evaluation of our manuscript and for recognizing its contribution. We also appreciate your suggestions, which have been incorporated into the revised version as detailed below.

Minor Comments and Suggestions
• The first Abstract sentence appears to generalize the characteristics of B chromosomes based on a limited number of well-characterized cases. While earlier studies described Bs as largely composed of repetitive DNA and transcriptionally inactive, more recent evidence indicates that transcriptional activity (Navarro-Domínguez et al. 2017; Dhar et al. 2019; Rajpal et al. 2023) occurs in certain taxa. This may depend on their "evolutionary age," but inactivity is certainly not a universal feature. As Abstracts are often cited in subsequent publications, it would be advisable to moderate this claim, particularly considering that fewer than 1% of known B-chromosome-bearing species have been studied in sufficient molecular detail to support such generalisations.
**AUTHORS:** This sentence was modified accordingly. Please see lines 35-37.

• While the study infers a recent origin of Bs, it would be informative, if such data are available, to include the frequency of B+ individuals (both males and females) within the B+ stock.
**AUTHORS:** The information about the number of B chromosomes in the stock is presented in line 364 “On average, each fly in this stock carries 10-12 B chromosomes…”.

• Lines 321-330 - The reported values (2.63% for 0B-WT, 3.28% for 0B-TM3, and 3.50% for +B stock) show greater differences between the 0B stocks than between 0B-TM3 and B+. Could the authors clarify what might cause such variation among the B0 stocks?
**AUTHORS:** We believe such variation among 0B stocks can arise from differences in their genetic background (e.g., 0B-WT does not have the *TM3* variant of chromosome 3 like 0B-TM3) and maintenance history (0B-WT and 0B-TM3 are each inbred stocks that have not interbred for decades), both of which could manifest as a difference in repetitive content. Such inter- and intrastock variation in satellite DNA has been previously reported in *Drosophila*, including pericentromeric satellites, and can contribute to differences in read depth across stocks (Courret and Larracuente, 2023, https://academic.oup.com/genetics/article/225/4/iyad176/7285264).

For this reason, we intentionally included two independent 0B stocks in our analyses. This design allows us to evaluate satellite DNA abundance in the +B genome relative to more than one 0B background, thereby reducing the likelihood that observed

differences reflect idiosyncrasies of a single control line. Importantly, although the two 0B stocks differ from each other, both show consistently lower satellite DNA abundance than the +B stock.

Through the more detailed analyses presented in the Results, we were able to demonstrate that part of this higher abundance of satDNA between 0B and +B stocks is associated with the B chromosomes, which represents a key finding of our study. Please see lines 378-393.

• Table 1: Please round the value 55.75 to 55.8 in the last two columns for consistency.
**AUTHORS:** Corrected.

**Reviewer #2:**
This manuscript characterizes the sequence composition and epigenetic state of supernumerary B chromosomes in Drosophila melanogaster. The authors compare short-read genomic libraries from +B and 0B strains, identify satellite DNAs (including three novel repeats), quantify transposable elements, map several satellites cytogenetically, and use IF/FISH to demonstrate that the B chromosomes are heterochromatic and depleted of RNA polymerase II. The study provides a valuable resource and establishes a baseline model for investigating satellite DNA dynamics and B chromosome biology. Beyond its relevance for understanding B chromosome evolution and maintenance, this system also offers an excellent opportunity to explore how additional heterochromatic domains influence overall chromatin organization and genome regulation.
Overall, the manuscript is clearly written and well organized. I have one major concern regarding the quantification of low-complexity satellites, along with several minor comments listed below.
**AUTHORS:** Thank you for the positive and rigorous evaluation of the manuscript. We agree with the comments regarding the quantification of low-complexity repeats. Accordingly, we estimated their abundances using k-mer–based analyses. All additional suggestions were carefully considered, and the corresponding modifications have been incorporated into the manuscript. A detailed explanation of these changes is provided below.

Major comments
1. The +B library was sequenced on HiSeq while the two 0B libraries were on NextSeq. Different platforms, run conditions, and library preps can introduce platform-specific biases especially regarding low-complexity sequence (such as short simple repeats). This is particularly worrying because the core claims about enrichment of specific satellites in +B are quantitative (fold-enrichments). This could explain why the authors report nine satellites enriched in +B genomes but cytogenetically confirm only three on the B. At least provide explicit controls to rule out/quantify batch effects, like a PCA or hierarchical clustering on satellite abundance (all satellites and A-linked satellite) to check whether samples cluster by genotype (+B vs 0B) versus by sequencing

run/platform. If there is indeed a batch bias, an orthogonal approach might be necessary to confirm the quantitative difference for the most abundant satellite candidate.

**AUTHORS:** We thank the reviewer for the thoughtful comment regarding potential sequencing-platform biases between the +B (HiSeq) and 0B (NextSeq) datasets. We agree that platform differences must be carefully evaluated, especially for low-complexity sequences such as simple repeats and satellite DNAs. We therefore examined several independent indicators of potential platform effects, as detailed below.

First, prior to any quantification of satellite DNA abundance, all libraries were processed using two preprocessing scripts from the satMiner workflow (Ruiz-Ruano et al. 2016), which are now explicitly described in the revised Methods, see lines 228-236. Initially, *fastq_edit_ids.py* was applied to standardize FASTQ read identifiers by removing platform-specific metadata and enforcing a uniform paired-end format (readID/1 and readID/2), ensuring full compatibility among libraries generated on different Illumina platforms. Subsequently, raw paired-end reads were processed with *rexp_prepare_normaltag.py*, which performs adapter trimming and quality filtering using *Trimmomatic* with Phred+33 quality score encoding, applying a sliding-window filter (4 bp window, minimum Phred score of 30) and a minimum read length of 101 bp. This script also performs random subsampling to equalize sequencing depth across libraries, read shuffling, and conversion to FASTA format. Together, these steps ensure that all datasets enter downstream analyses with comparable read structure, quality, depth, and formatting, thereby minimizing discrepancies that could arise from HiSeq versus NextSeq sequencing.

As a general indicator of potential platform-specific biases, we examined the GC content of all libraries using FastQC. All genomes (+B and both 0B strains) exhibited an identical GC content of 41%, with no systematic shifts between HiSeq (+B) and NextSeq (0B) samples, suggesting the absence of major GC-related batch effects. We have added this information to the Methods section, lines 160-164.

Following the reviewer's suggestion, we performed PCA analyses based on satellite abundance, including (i) all satellite families and (ii) only satellites previously identified as A-linked. In both analyses, samples clustered primarily by genotype (+B, WT_0B, TM3_0B) rather than by sequencing platform.

When all satellites were included, $B^+$ genomes were clearly separated from both 0B backgrounds, consistent with the amplification of B-linked satellites. This separation was markedly reduced when only A-linked satellites were considered, while the relative positions of the two 0B strains remained stable.

To quantify these patterns, we calculated Euclidean distances between genotype centroids. Distances dropped by more than half when only A-linked satellites were considered, whereas the distance between the two 0B strains, , sequenced on the same platform, remained similar across analyses. Notably, the magnitude of the distance between the two 0B stocks was similar to that observed between +B and 0B genotypes in the A-linked PCA, indicating that sequencing platform alone does not account for the observed divergence in satellite abundance. Importantly, no configuration indicative of HiSeq–NextSeq clustering emerged. Please see Supplementary Figure 1 and explanations on lines 164-173.

Taken together, these analyses indicate that while platform-specific effects cannot be entirely excluded, they are unlikely to represent the primary driver of the observed divergence in satellite abundance. (i) The standardized preprocessing of all datasets using the satMiner workflow, (ii) PCA analyses showing that samples cluster primarily by genotype rather than by sequencing platform, and (iii) the identical GC content across all libraries collectively suggest that the enrichment of satDNAs observed in +B genomes is not driven primarily by HiSeq–NextSeq diferences. Instead, these results are consistent with a biological contribution of B chromosomes to the increased abundance of specific satellite families.

2. The authors claim that the AAGAGAAGAT probe is specific and does not cross-hybridize to AAGAG or AATAT, based on high stringency hybridization, but no co-hybridization or direct control images are provided. Because AAGAGAAGAT is composed of AAGAG + AAGAT subunits, cross-hybridization is plausible. Please provide FISH co-hybridization experiments showing AAGAGAAGAT together with AAGAG and AAGAT probes to demonstrate non-overlap/overlap patterns. Provide the hybridization temperature and buffer stringency conditions used for each probe. State whether the same conditions were used across probes.

**AUTHORS:** We thank the reviewer for this valuable comment. Indeed, given the shared motifs between these sequences, some level of cross-hybridization could be theoretically expected. Since the melting temperatures of the FISH probes are so similar, we were concerned that adjusting hybridization temperatures may not resolve the issue of probe specificity, therefore we performed a competitive inhibition FISH experiment. Briefly, two unlabeled FISH probes targeting *AAGAG* and *AAGAT* repeats and two labeled FISH probes targeting *AAGAT* and *AAGAGAAGAT* repeats were applied to one sample and imaged. If the *AAGAGAAGAT* probe was binding to either of the 5mer repeats, then we would expect its signal to be diminished, however if the probe was specific for the 10mer repeat then we should see no loss of signal. Our results show that *AAGAGAAGAT* signal from chromosomes X, Y, 2, and 3 are completely abolished, indicating the *AAGAGAAGAT* probe was binding nonspecifically. When we perform the same experiment but add in a third unlabeled probe for *AAGAGAAGAT*, all signal from chromosome 4 and the B chromosomes disappears, leading us to conclude that the signal we observe on chromosome 4 and the B chromosomes in the competitive inhibition FISH experiment is real. The full details for this experimental approach are in the Methods section (lines 279-315). We want to reiterate our gratitude to this reviewer for their insightful suggestion as our new results are likely reflect the true biology and are more consistent with our genomic results. We also revised the text (lines 407-423), updated Figure 1b, and included a new supplementary figure (see Supplementary Figure 2) showing the complete competitive inhibition FISH results in the presence of (a) *AAGAT* and *AAGAG* and (b) *AAGAT, AAGAG,* and *AAGAGAAGAT* unlabeled probes.

3. The methods used for repeat identification and quantification do not appear optimal for low-complexity satellites (such as AAGAG). First, the authors use SatMiner (based

on RepeatExplorer) to identify satellite sequences in each library. While this pipeline performs well for complex and longer satellite arrays, it probably to underestimate or filter out short, low-complexity repeats, such as those composed of 5-7 bp monomers.
AUTHORS: We appreciate the reviewer's comment. We acknowledge that RepeatExplorer alone may have limitations in detecting low-complexity satellite DNAs, particularly those composed of short monomers. However, in our study RepeatExplorer was not used in isolation, but within the SatMiner framework, which improves satellite detection by filtering reads and performing iterative clustering analyses. This approach progressively enriches for repetitive reads and reduces background noise, thereby increasing the sensitivity for detecting abundant tandemly organized satellites, including low-complexity families.

Accordingly, low-complexity satellite DNAs were successfully identified during the initial SatMiner/RepeatExplorer analyses. Importantly, RepeatExplorer was not used for quantification. After satellite identification, genomic abundance was estimated using RepeatMasker with a custom library of satellite consensus sequences, where short monomers (5–7 bp) were concatenated to a minimum length of 200 bp and low-complexity masking was disabled. We agree that quantification of low-complexity satellites can be challenging, and therefore, following the reviewer's suggestion, we additionally performed a k-mer-based analysis using k-seek to obtain more accurate abundance estimates. The results obtained with *k*-Seek were concordant with those from the RepeatMasker-based approach, providing independent support for the observed abundance patterns.

Second, for quantification, the authors apply RepeatMasker using a library of consensus sequences, where simple satellites are represented by single monomer units, and subsequently estimate genomic abundance and Kimura (K2P) divergence. It is unclear how reads were mapped to such very short consensus sequences.
**AUTHORS**: For satDNAs with short monomer units, individual sequences were concatenated until they reached a minimum length of 200 bp prior to RepeatMasker analyses. This strategy is widely used in satDNA quantification to ensure reliable read mapping and accurate divergence estimates for low-complexity repeats. By increasing the effective length of the consensus sequences, this approach minimizes ambiguous alignments while preserving the underlying monomer structure. This clarification has now been added to the Methods section; please see lines 243-249.

The authors should specify: How reads aligning to 5-6 bp consensus sequences were handled (e.g., minimum alignment length, mismatch tolerance, multi-mapping strategy); How K2P divergence was calculated for such short motifs, given that this metric is not typically robust for monomeric repeats.
**AUTHORS:** We thank the reviewer for this comment and clarify that reads were not aligned to 5–6 bp monomeric consensus sequences. RepeatMasker was run using the SatMiner pipeline (repeat_masker_run_big.py) with a custom library of satellite consensus sequences, in which short low-complexity monomers (5–7 bp) were concatenated to a minimum length of 200 bp prior to analysis, a standard approach

widely used in satDNA research. This information has now been added to the manuscript, please see 243-249.

RepeatMasker was executed using default alignment scoring parameters based on a Smith–Waterman local alignment algorithm with a minimum alignment score cutoff (default value 225). Importantly, the -nolow option was applied to prevent pre-masking of low-complexity regions, ensuring that simple satellite sequences were not excluded prior to alignment. This score-based framework implicitly constrains alignment length and mismatch tolerance, such that only statistically significant and sufficiently long alignments are reported.

Reads mapping to multiple instances of a satellite sequence were retained, as RepeatMasker reports all significant matches above the score cutoff, which is appropriate for highly repetitive sequences. Kimura 2-parameter (K2P) divergence was calculated using calcDivergenceFromAlign.pl based on the full concatenated alignments, ensuring robust divergence estimates for low-complexity satellite DNAs.

Overall, this analytical framework is widely used for transposable elements and complex satellites, but it is not well suited for simple satellites, where k-mer-based approaches are more appropriate.
**AUTHORS:** As suggested, we additionally quantified the abundance of low-complexity satDNAs using k-seek analysis.

Moreover, the abundance estimates reported here are unexpectedly low. The total satellite content is estimated at only ~3% of the genome, whereas previous studies report ~20% for D. melanogaster. Similarly, the AAGAG satellite-one of the most abundant repeats in D. melanogaster-is reported at just 0.0028%, compared to prior estimates around 5%. Such discrepancies strongly suggest that the current approach underestimates simple satellite abundance.
**AUTHORS:** As suggested, we additionally quantified the abundance of low-complexity satellite DNAs using a k-mer–based approach. This analysis resulted in slightly higher abundance estimates for low-complexity satellites compared to RepeatMasker-based estimates; however, these values remain lower than some previous reports in the literature.

Importantly, estimates of total satellite DNA content and individual satellite abundance in *Drosophila melanogaster* vary widely across studies and are known to be controversial. For instance, early reports suggesting that satellite DNAs occupy >20% of the genome (e.g. Lohe and Brutlag, 1986) relied on dot-blot hybridization, whereas more recent studies based on genomic approaches report substantially lower values. In particular, Wei et al. (2014) explicitly discuss inter-population variation in satellite abundance, although the values of AAGAG in % is given explicitly in figure 2 and in the dataset S1 it is abundance values of approximately 1,259,486 bp. When normalized to the *D. melanogaster* genome size (~180 Mb), this corresponds to ~0.7% of the genome, rather than ~5%.

Similarly, more recent genomic analyses (e.g. de Lima and Ruiz-Ruano, 2022) estimate total satellite DNA content at ~6.6%, further highlighting the variability among

studies and methodologies. These discrepancies reflect not only methodological differences but also the highly dynamic nature of satellite DNAs, whose abundance can vary substantially among laboratory stocks, as documented for pericentromeric satellites in *Drosophila* (Courret and Larracuente, 2023), due to multiple non-exclusive factors, including high mutation rates, genetic drift and inbreeding effects, heterochromatin polymorphisms, chromosomal rearrangements, independent laboratory histories, and stock maintenance practices.

In addition, the RepeatMasker- and k-mer–based approaches differ fundamentally in the type of satellite organization they capture. The RepeatMasker strategy relies on Smith–Waterman local alignments against concatenated consensus sequences (~200 bp) for simple satellites, and therefore preferentially detects long, tandemly organized satellite arrays. In contrast, k-mer–based methods quantify short sequence motifs independently of their genomic context, capturing not only canonical tandem arrays but also dispersed, fragmented, or degenerate repeat occurrences. As a consequence, k-mer approaches may yield higher abundance estimates for simple satellites by including non-tandem signals, whereas RepeatMasker provides a more conservative estimate of organized tandem arrays. These differences further contribute to discrepancies in absolute abundance estimates, particularly for low-complexity satellites. As expected, comparisons between RepeatMasker- and k-mer–based estimates revealed quantitative differences for individual satellite families, with some satellites showing higher abundance in one method and others in the alternative approach.

Crucially, however, both methods consistently revealed increased abundance of specific low-complexity satellites in B-chromosome stocks relative to non-B stocks. This concordant pattern across independent quantification strategies strongly supports our conclusion that these satellites are enriched in B chromosomes.

For these reasons, we consider it important to retain both quantification approaches in the manuscript. Methodological differences and comparisons with previous estimates are now explicitly described in the Methods and discussed in the Results section, please see lines 246-249; 378-406.

The authors should:
- Compare their estimates to previous genome-wide quantifications and explicitly discuss potential sources of discrepancy (e.g., platform bias, filtering thresholds, pipeline limitations).
**AUTHORS:** Done. Comparisons with previous genome-wide estimates and a detailed discussion of potential sources of discrepancy, including methodological differences, quantification strategies, and stock-specific variation, have now been incorporated into the Methods and Results sections (lines 244–247; 345–372). As detailed above, PCA analyses indicate that sequencing platform effects are unlikely to be the dominant factor underlying the observed patterns. Please see lines 246-249; 378-406.

- Reanalyze simple satellites using tools specifically designed for short, low-complexity repeats, such as k-seek (Wei et al. 2018) or other k-mer-based quantification methods, to validate and refine their results.

**AUTHORS:** Done as suggested. We observed differences in quantification depending on the satDNA. Most important in our analysis is that the increase in abundance was consistently observed in the +B stocks, independent of the method of quantification. It gives more support to our previous observation of the enrichment of these specific satDNAs in +B stock. We believe that it is important to maintain both estimations, and we explain these details in the Methods and discuss the differences between our estimations and previous analysis in the Results. Please see lines 246-249; 378-406.

4. I would like to see a clearer and more explicit comparison between the genetic composition and epigenetic state of the D. melanogaster B chromosome and those described in other species where B chromosomes have been extensively studied (e.g., grasshoppers, wasps, or beetles). Such a comparison would help place the findings in a broader evolutionary context and highlight whether the D. melanogaster B shares common features-such as repeat composition, gene content, or transcriptional silencing mechanisms-or represents a distinct type of B chromosome.
**AUTHORS:** We included some information about this topic based in the data published for insects. Please see lines 644-665.

Minors comments
- Page 4, line 109: Reference missing for Hanlon et al. 2018 - please add the citation.
**AUTHORS:** Included.

- Page 4, line 114: Please cite Jagannathan et al. 2018 when discussing the role of satellite DNA in centromere function.
**AUTHORS:** The role of satDNAs and their function at the Drosophila centromere is discussed in detail at line 585. This reference was added at this point.

- Page 7, line 189: The manuscript states that a coverage plot (RepeatProfiler) was generated to validate the tandem organization of the novel satellites. Please include this coverage plot as a Supplementary Figure.
**AUTHORS:** The plots are presented in Supplementary Figure 4.

- Page 13 / Figure 1a: Materials indicate three replicate libraries per genotype. Show replicate variation in Figure 1a and add error bars.
**AUTHORS**: Figures 1a and 1d has been revised to show variation among the three replicate libraries. Due to the very small standard deviation values, error bars were not visually discernible at the scale of the plot; therefore, SD values are now displayed above the bars. The figure legend has been updated to reflect this change.

- Do the authors find the three novel satellites (Sat-228, Sat-307, Sat-597) only in D. melanogaster, or are they present in other Drosophila species?
**AUTHORS:** We checked the presence of these satDNAs only in *D. melanogaster*. Other species were not analyzed.

- Page 20, line 507: Incorrect citation year for Courret et al. - should be Courret et al. 2024 (not 2019).

**AUTHORS:** corrected.

**Reviewer #3:**

The authors analysed the repeat composition of 0B and +B Drosophila melanogaster and performed immuno/FISH experiments to decipher the general chromatin composition of the B chromosome. A similar chromatin composition was found for chromosome 4 and the chromosome 4-derived B chromosome.

The results are of interest, but more care should be taken in interpreting the immunostaining results (see line 461 and others).

The conclusion that chromosome 4 and the Bs are transcriptionally silent is likely too strong. Because it has been shown that chromosome 4, although mostly heterochromatic, contains ~80-100 protein-coding genes. These are expressed during development. See: 3 Genes|Genomes|Genetics

Whether the same is true for the B chromosome of Drosophila is unknown.

I suggest talking about low transcription activity rather than no activity. Alternatively, a comparative RNAseq analysis of 0B and +B specimens would be the best way to address this question. In the future, ChIP-seq with antibodies recognising eu- or heterochromatin-specific histone marks should be considered for a detailed analysis of the B chromosome's chromatin composition.

**AUTHORS:** Thank you for the positive evaluation of our manuscript and for drawing attention to the point regarding B-chromosome silencing. First, we have made it clearer in the manuscript that we are referring to the heterochromatic portion of chromosome 4 since our FISH probe localizes to the heterochromatin portion of chromosome 4. Second, we have softened our language regarding B-chromosome silencing and talk about decreased level of transcription in comparison to other chromosomes (e.g. arms of chromosomes 2 and 3) or directly low level of transcription. Please see lines 51, 52, 131-133, 539, 540, 545, 546, 631, 632, 665-667. We hope in the future to obtain RNA-seq (or other) data to explore this topic in greater detail.

Nevertheless, we recognize that our data are cytological and do not directly measure transcription. To avoid overinterpretation, we have revised the manuscript throughout to adopt a more cautious tone, replacing statements of “inactivation” or “silencing” with “decreased level of transcription”, “decreased transcriptional activity” or “low level of transcription” and emphasizing the comparative nature of our observations relative to other chromosomal regions (e.g., chromosome arms 2 and 3). These changes can be found in lines 51, 52, 131-133, 539, 540, 545, 546, 631, 632, 665-667.

We hope that future RNA-seq–based analyses, as suggested by the reviewer, will allow a more direct assessment of B-chromosome transcription and further refine these conclusions.

Additional points:

ABSTRACT:
line 35-36
The statement that Bs are generally silenced is not correct any more. Recent literature has described many B chromosome-encoded active genes across diverse species, and it has been shown that the B chromosome can influence, in trans, the transcriptome of the standard A chromosomes. Thus, tone down this general statement.
**AUTHORS:** Yes, you are totally correct – we changed the sentence accordingly. Please see lines 35-37.

Introduction
Line 73
A non-disjunction event alone will not result in a B chromosome. It may be that non-disjunction triggered chromosome rearrangements of micronucleated chromosomes. Please rephrase the sentence.
**AUTHORS:** The sentence was rephrased based on the information in Houben et al. (2025); only this reference was maintained in the sentence. Please see lines 71-75.

Line 87
Do you mean that B chromosomes do not recombine with a B chromosome homolog, or do you refer to A chromosomes?
**AUTHORS:** We meant that the B chromosomes generally do not recombine their progenitor; revised. Please see lines 85-86.

Discussion
Line 583 - 594
To improve the study's impact, I suggest comparing the findings from Drosophila B with those of other species regarding their chromatin composition and transcriptional activity.
**AUTHORS:** We have added findings from other B chromosome systems to our Discussion. Please see lines 644-677.

April 22, 2026
RE: GENETICS-2026-309266

Dr. Diogo C. Cabral-de-Mello
Universidade Estadual Paulista Julio de Mesquita Filho
Biologia Geral e Aplicada
Avenida 24 A
Rio Claro 13506-900
Brazil

Dear Dr. Cabral-de-Mello:

Congratulations, your manuscript titled "Comparative genomic analysis and epigenetic profiling of the Drosophila melanogaster B chromosome" is accepted for publication in GENETICS! Many thanks for submitting your research to the journal.

To Proceed to Publication:
1. Format your article according to GENETICS style: https://academic.oup.com/genetics/pages/author-guidelines

2. Ensure that you comply with data and community resource citation guidelines:
https://academic.oup.com/genetics/pages/author-guidelines#section-5-9-2

3. Upload your final files at https://genetics.msubmit.net

4. Add oupsupport@scipris.com and genetics.oup@novatechset.com (or the domains @scipris.com and @novatechset.com) to your email program's "safe senders" list. You will be contacted by both at various points during the production process.

Notes:
- Your currently-accepted manuscript (unedited, as submitted, reviewed, and accepted) will be published at GENETICS and deposited into PubMed as an Advance Access article. Notify sourcefiles@thegsajournals.org before signing your license if you do not wish to publish your article via Advance Access.

- We invite you to submit an original color figure related to your paper for consideration as cover art. Please email your submission to the editorial office or upload it with your final files. You can submit a small-sized image for evaluation, and if selected, the final image must be a TIFF file 2513px wide by 3263px high (8.375 by 10.875 inches; resolution of 600ppi). Please avoid graphs and small type.

- After files are sent to Oxford University Press we use SciPris to manage article licensing and payment. If you do not have a SciPris account, you will receive an email from no-reply@scipris.com to sign up to use Oxford University Press' author portal. After logging in, follow the online instructions to sign your license and arrange any payment due.

If you have any questions or encounter any problems while uploading your accepted manuscript files, please email the editorial office at sourcefiles@thegsajournals.org.

Sincerely,

Ching-Ho Chang
Associate Editor
GENETICS

Approved by:
Kelly Dyer
Senior Editor
GENETICS

Review comments (if applicable):

Reviewer #1 :

Dear Authors,
You have made substantial efforts to revise the manuscript, resulting in a clear and significant improvement. The inclusion of extensive supplementary material effectively supports the presented results. Importantly, the discussion of B chromosomes has been updated in line with current research, appropriately challenging the outdated view of their inactivity.
The figures are of satisfactory quality and suitable for publication. This study is particularly valuable as it characterizes the genetic composition of the D. melanogaster B chromosome that evolved under laboratory conditions, and provides important insights into its epigenetic profile within a highly controlled environment. I expect that future research building on this foundation will yield further interesting and meaningful findings. As such, this model system represents a valuable platform for advancing research on B chromosomes.

I wish you all the best in your future research.
Jelena Blagojevic

Reviewer #2 :

I really appreciate the effort made by the authors to revise their manuscript entitled "Comparative genomic analysis and epigenetic profiling of the Drosophila melanogaster B chromosome". The authors carefully addressed each of the reviewers' comments and detailed the modifications they made to the manuscript.
I particularly appreciate the rigorous effort made by the authors to further validate their low-complexity satellite analyses by adding control and orthogonal approaches (e.g., PCA, k-mer-based approaches, and competitive FISH).
I have no concerns regarding the methodology used, and their results clearly support their conclusions.

Reviewer #3 :

I'm satisfied with the improved manuscript.
