# Supplementary material for: Comparative genomic analysis and epigenetic profiling of the Drosophila melanogaster B chromosome
Source: Genetics. 2026 May 5;233(4):iyag113. doi: 10.1093/genetics/iyag113 (PMC13439713; doi:10.1093/genetics/iyag113)
Supplement: Supplementary file 2 [file iyag113_supplementary_data.zip › Supplemental_Material_Legends_GENETICS-2026-309266.docx]

**Supplementary Material**

**Supplementary File 1.** FASTA file containing monomers of all the repetitive sequences used for quantification in the genomes of *D. melanogaster* analyzed.

**Supplementary Table 1.** Oligos used for probes production for each of the repetitive sequences mapped on the chromosomes by FISH.

**Supplementary Table 2.** Antibodies used in immunostaining analyses, including their type, host species, catalog number, and producer.

**Supplementary Table 3.** Estimation of abundance based in K-mer analysis (Wei et al. 2014; 2018) for low-complexity satDNAs in all libraries studied (0B-WT, 0B-TM3 and +B) and the sequenced pulsed field electrophoresis B (PFGE-B). Note that the ratio between B+ libraries and 0B libraries falls in the same pattern observed when the abundance is estimated through RepeatMasker.

**Supplementary Table 4** **.** The table displays the average genomic abundance (%) and divergence (K2P %) of TE superfamilies across the three genomes (0B-WT, 0B-TM3 and +B) of *D. melanogaster* and the sequenced pulsed field electrophoresis B (PFGE -B). The last two lines provide a summary of the total genomic abundance and average divergence for all TEs examined.

**Supplementary Table 5.** Genomic abundance (%) and divergence (K2P %) of each multigene family in the three genomes analyzed (0B-WT, 0B-TM3 and +B) of *D. melanogaster* and the sequenced pulsed field electrophoresis B (PFGE -B).

**Supplementary Figure 1.** Principal component analysis (PCA) of satDNA abundance reveals genotype-specific clustering. PCA based on (a) all identified satDNAs and (b) autosomal (A-linked) satDNAs shows separation among +B, 0B-TM3, and 0B-WT genotypes. Points represent biological replicates, colored by genotype. Dashed arrows connect genotype centroids, and Euclidean distances between centroids are indicated, illustrating differences in overall satDNA abundance profiles among genotypes. Notably, the +B genotype shows a larger distance from the 0B genotypes when considering all satDNAs, whereas distances are more similar when only A-linked satDNAs are analyzed.

**Supplementary Figure 2.** The *AAGAGAAGAT* satellite repeat is enriched on the B chromosomes. (a) Same image as shown in Figure 1b but with the *AAGAT* channel included. FISH using fluorescently labeled probes targeting *AAGAT* and *AAGAGAAGAT* in the presence of unlabeled competitive inhibition probes for *AAGAT* and *AAGAG* to ensure hybridization specificity (see Materials and methods for details). A FISH probe targeting the *AAGAGAAGAT* satellite repeat hybridizes to the B chromosomes (inset) and chromosome 4. (b) Same approach as in (a) but with the addition of an unlabeled competitive inhibition probe for *AAGAGAAGAT*, resulting in no signal in the *AAGAGAAGAT* channel. See Materials and methods for brightness and contrast adjustments. Scale bar, whole karyotype = 5 µm; inset = 0.5 µm.

**Supplementary Figure 3.** FISH mapping of the *AAGAT* probe for validation in mitotic metaphase and interphase of brain cells, as well as in late primary spermatocytes of male germline cells, from Oregon-R (0B) and +B stocks. White arrows indicate chromosome 4, and the dotted line encircles the territory of chromosome 4 in 0B cells, or the territory of chromosome 4 plus the B chromosome in B+ cells. Note the heightened signal amount in interphase and spermatocytes with B chromosomes.

**Supplementary Figure 4.** RepeatProfiler analysis of each of the three new satDNA families using Illumina reads from +B, WT, TM3 and additionally three Oregon genomes (see methods) genomes of *D. melanogaster*. Note the presence of similar coverage in the connection of monomers (shown by the red arrow), evidencing occurrence of hybrid reads

and in tandem arrangement.

**Supplementary Figure 5.** Mapping of the three novel satDNAs onto PacBio reads (accession number SRX26679649) revealed their tandem arrangements, confirming them as true satDNAs, (a) Sat-307, (b) Sat-597, and (c) Sat-208. The names of the satDNAs, along with the corresponding read accession numbers, are indicated directly in the images. For each analysis, the reads with the highest similarity were selected through BLAST (megablast) using trimers of each satDNA (left panel). Specific regions showing tandem repeat accumulation are enlarged in the right panel.

**Supplementary Figure 6.** Tandem pattern of the Sat-228 satellite DNA within the protein-coding gene Ankyrin 2 (FlyBase ID: FBgn0261788), also present in two of its transcripts: *Ank2-RU* (FlyBase ID: FBtr0303125) and *Ank2-RAA* (FlyBase ID: FBtr0344224), with the same array pattern observed in the gene.

**Supplementary Figure 7.** Dotplot images with lined squares highlighting the *Ank2* repeat in each polypeptide sequence. For the two polypeptides translated from the isoforms containing Sat-228 (*Ank2*-*PAA*: FBpp0310630 and *Ank2*-*PU*: FBpp0292244), purple-lined squares highlight the satDNA repeat. Note the presence of the *Ank2* repeat, independent of the presence of the satDNA repeat.

**Supplementary Videos 1 - 12.** 3D imaging from the immunostaining using the same antibodies from Figure 3 and 4, for male brain somatic interphase cell and late primary spermatocytes male germline cell of *D. melanogaster* +B stock. The antibody is colored in green, the B chromosome territory in red, and the whole DNA (DAPI) in blue.
