# Supplementary material for: Comparative genomic analysis and epigenetic profiling of the Drosophila melanogaster B chromosome
Source: Genetics. 2026 May 5;233(4):iyag113. doi: 10.1093/genetics/iyag113 (PMC13439713; doi:10.1093/genetics/iyag113)

Supplementary Figure 1. Principal component analysis (PCA) of satDNA abundance reveals genotype-specific clustering. PCA based on (a) all identified satDNAs and (b) autosomal (A-linked) satDNAs shows separation among +B, 0B-TM3, and 0B-WT genotypes. Points represent biological replicates, colored by genotype. Dashed arrows connect genotype centroids, and Euclidean distances between centroids are indicated, illustrating differences in overall satDNA abundance profiles among genotypes. Notably, the +B genotype shows a larger distance from the 0B genotypes when considering all satDNAs, whereas distances are more similar when only A-linked satDNAs are analyzed.

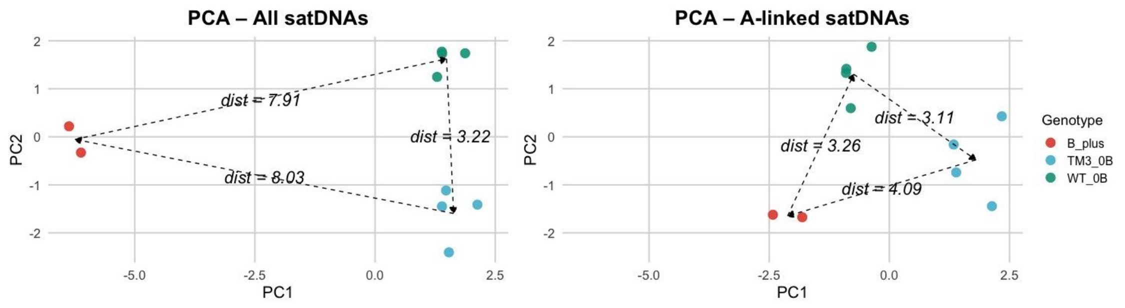

Supplement: Supplementary file 2 [file iyag113_supplementary_data.zip › Supplementary_Figure_1_GENETICS-2026-309266.pdf]
