# Supplementary material for: Comparative genomic analysis and epigenetic profiling of the Drosophila melanogaster B chromosome
Source: Genetics. 2026 May 5;233(4):iyag113. doi: 10.1093/genetics/iyag113 (PMC13439713; doi:10.1093/genetics/iyag113)

Supplementary Figure 2. The *AAGAGAAGAT* satellite repeat is enriched on the B chromosomes. (a) Same image as shown in Figure 1b but with the *AAGAT* channel included. FISH using fluorescently labeled probes targeting *AAGAT* and *AAGAGAAGAT* in the presence of unlabeled competitive inhibition probes for *AAGAT* and *AAGAG* to ensure hybridization specificity (see Materials and methods for details). A FISH probe targeting the *AAGAGAAGAT* satellite repeat hybridizes to the B chromosomes (inset) and chromosome 4. (b) Same approach as in (a) but with the addition of an unlabeled competitive inhibition probe for *AAGAGAAGAT*, resulting in no signal in the *AAGAGAAGAT* channel. See Materials and methods for brightness and contrast adjustments. Scale bar, whole karyotype = 5 µm; inset = 0.5 µm.

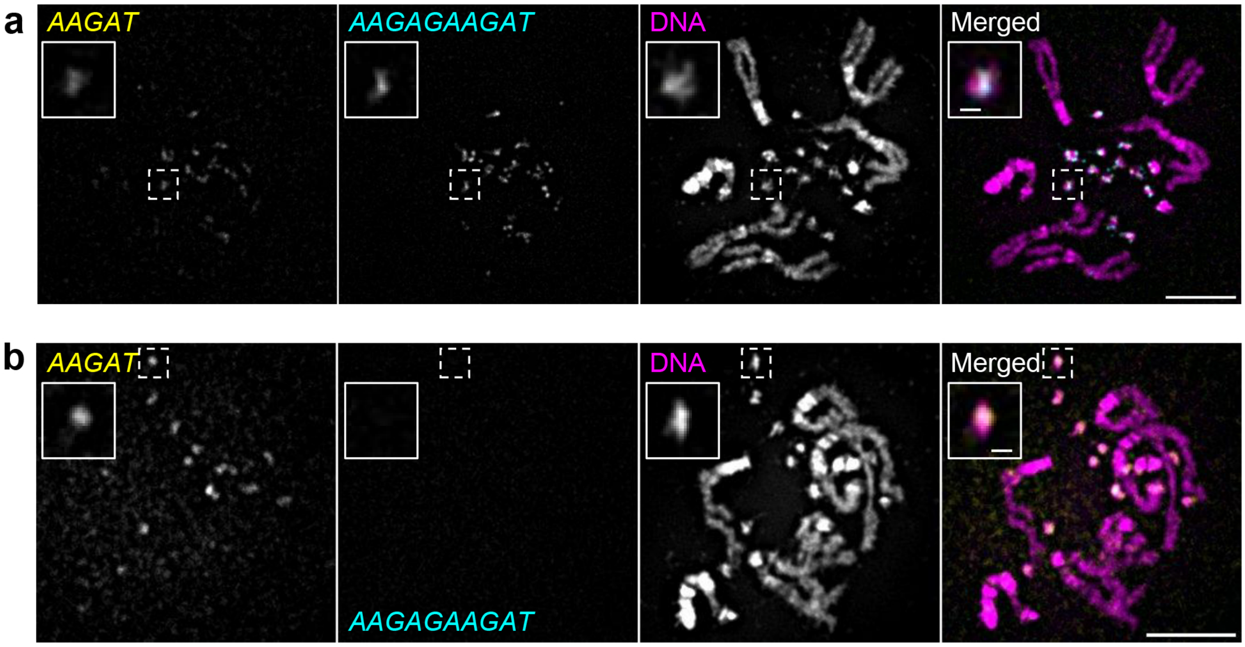

Supplement: Supplementary file 2 [file iyag113_supplementary_data.zip › Supplementary_Figure_2_GENETICS-2026-309266.pdf]
