# Supplementary material for: Comparative genomic analysis and epigenetic profiling of the Drosophila melanogaster B chromosome
Source: Genetics. 2026 May 5;233(4):iyag113. doi: 10.1093/genetics/iyag113 (PMC13439713; doi:10.1093/genetics/iyag113)

Supplementary Figure 3. FISH mapping of the AAGAT probe for validation in mitotic metaphase and interphase of brain cells, as well as in late primary spermatocytes of male germline cells, from Oregon-R (0B) and +B stocks. White arrows indicate chromosome 4, and the dotted line encircles the territory of chromosome 4 in 0B cells, or the territory of chromosome 4 plus the B chromosome in B+ cells. Note the heightened signal amount in interphase and spermatocytes with B chromosomes.

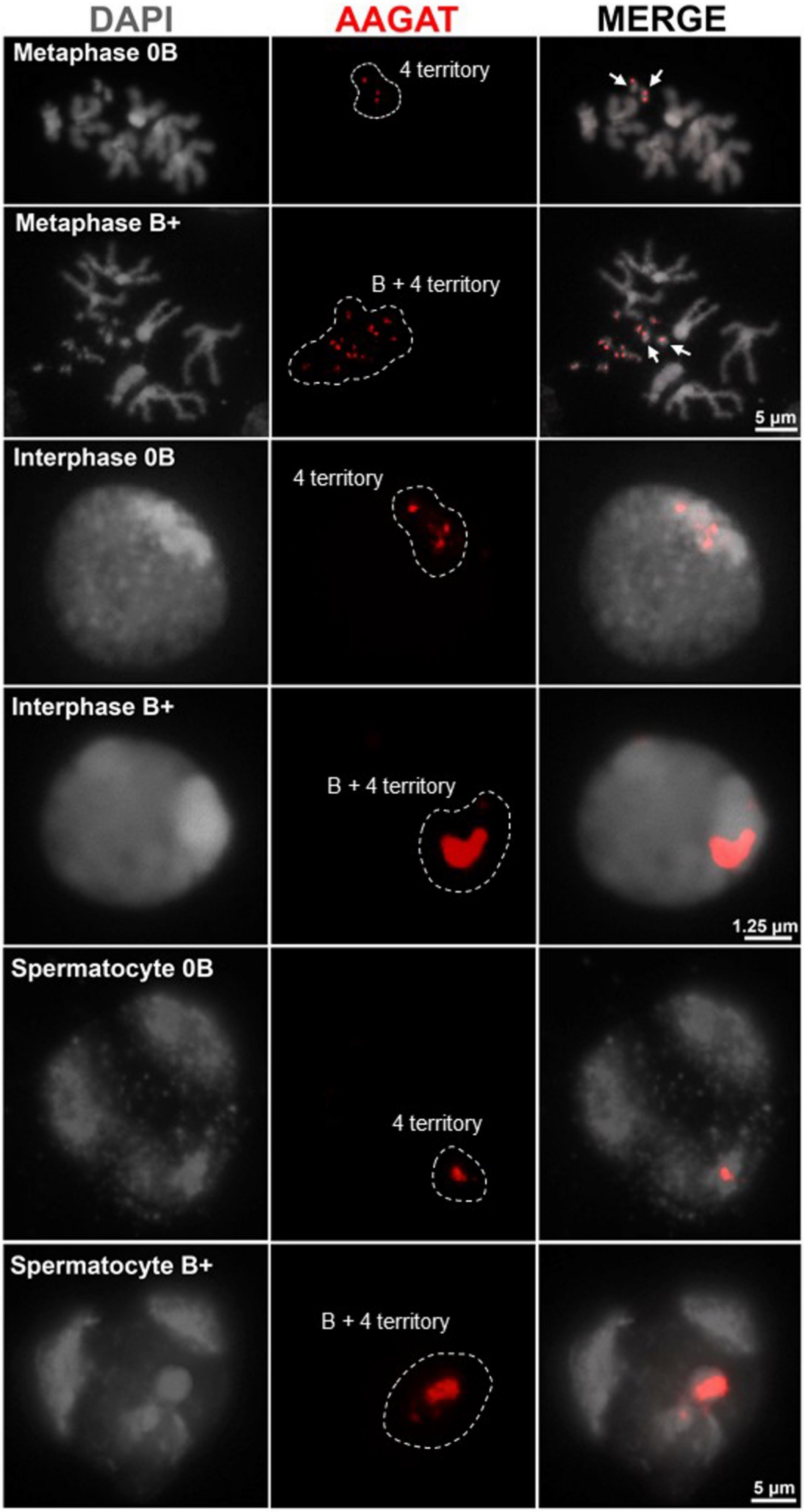

Supplement: Supplementary file 2 [file iyag113_supplementary_data.zip › Supplementary_Figure_3_GENETICS-2026-309266.pdf]
