# Supplementary material for: Comparative genomic analysis and epigenetic profiling of the Drosophila melanogaster B chromosome
Source: Genetics. 2026 May 5;233(4):iyag113. doi: 10.1093/genetics/iyag113 (PMC13439713; doi:10.1093/genetics/iyag113)
Supplement: Supplementary file 2 [file iyag113_supplementary_data.zip › Supplementary_Figure_4_GENETICS-2026-309266.pdf]

Supplementary Figure 4. RepeatProfiler analysis of each of the three new satDNA families using Illumina reads from +B, WT, TM3 and additionally three Oregon genomes (see methods) genomes of *D. melanogaster*. Note the presence of similar coverage in the connection of monomers (shown by the red arrow), evidencing occurrence of hybrid reads and in tandem arrangement.

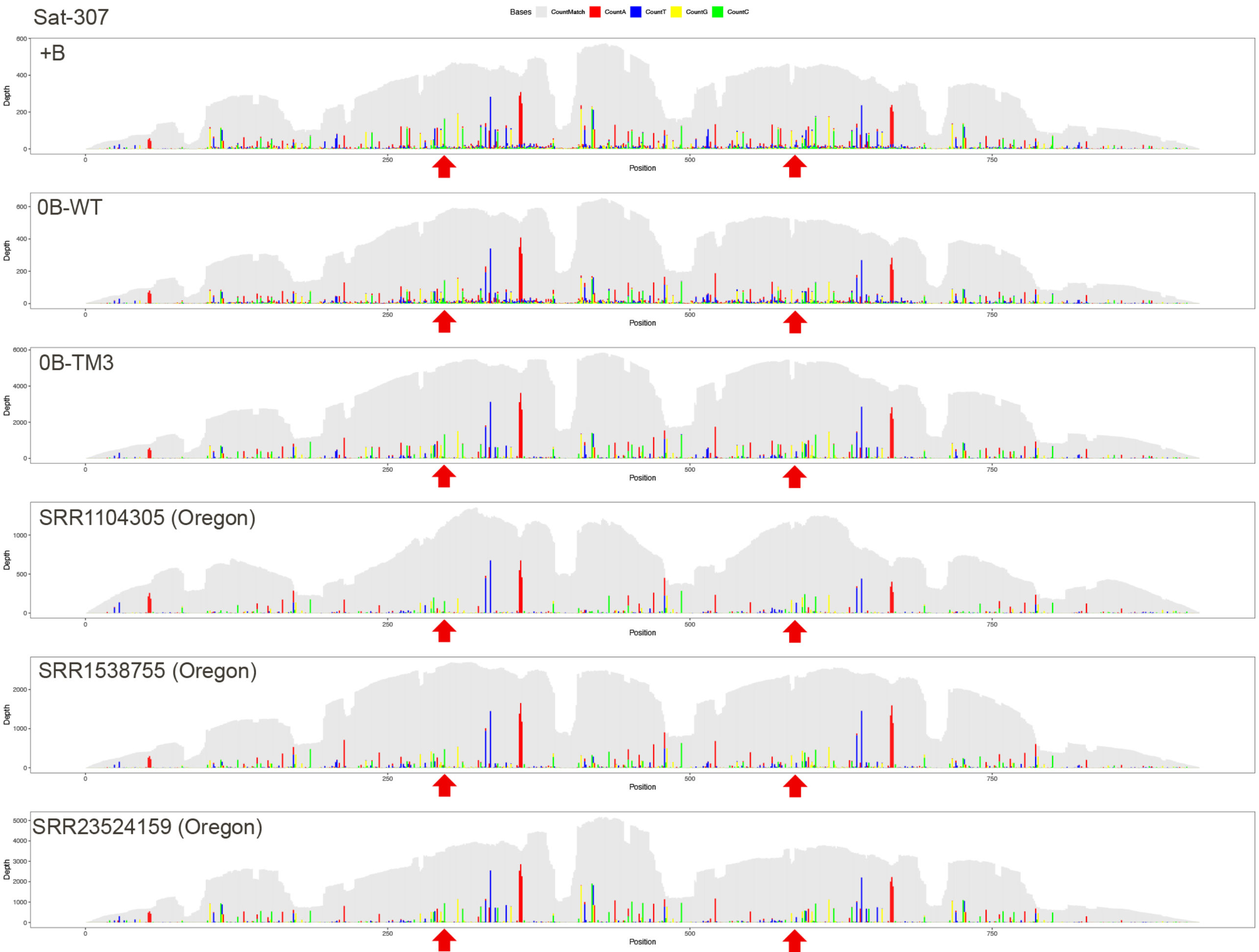

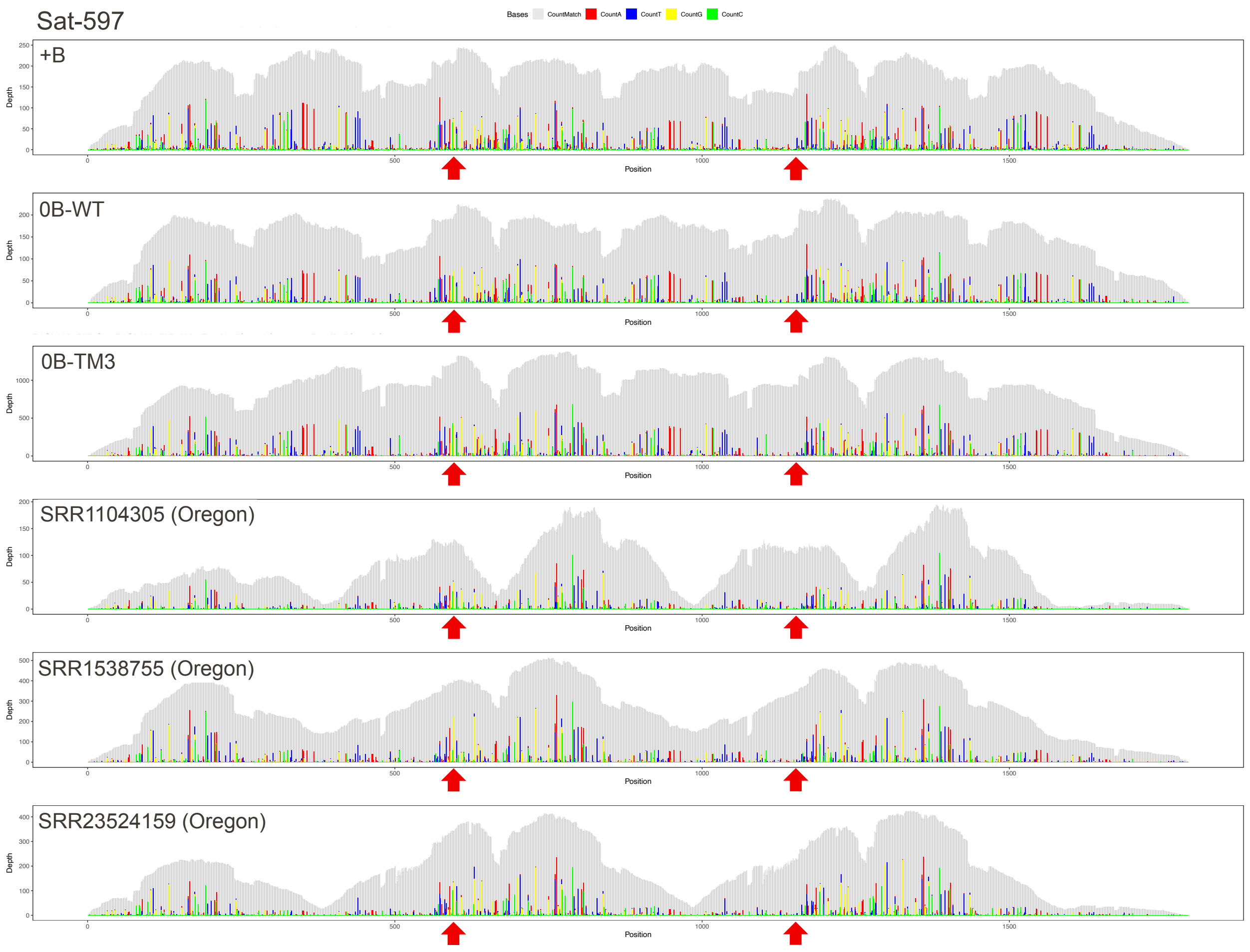
Sat-597
Bases CountMatch CountA CountT CountG CountC
+B
0B-WT
0B-TM3
SRR1104305 (Oregon)
SRR1538755 (Oregon)
SRR23524159 (Oregon)
Depth
Position
0
500
1000
1500

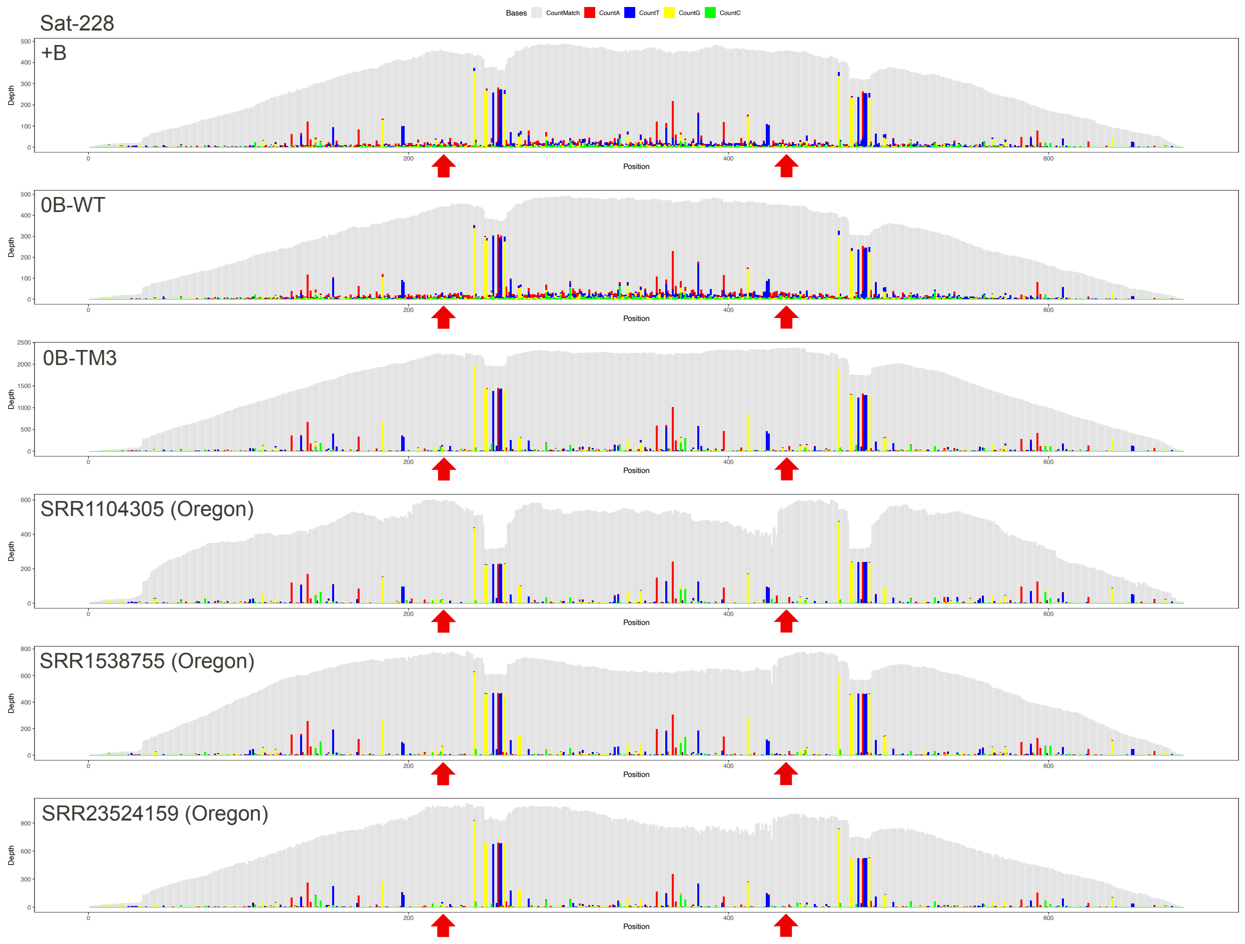
Sat-228
Bases CountMatch CountA CountT CountG CountC
+B
0B-WT
0B-TM3
SRR1104305 (Oregon)
SRR1538755 (Oregon)
SRR23524159 (Oregon)
Depth
Position
0
200
400
600
