# Supplementary material for: Comparative genomic analysis and epigenetic profiling of the Drosophila melanogaster B chromosome
Source: Genetics. 2026 May 5;233(4):iyag113. doi: 10.1093/genetics/iyag113 (PMC13439713; doi:10.1093/genetics/iyag113)

Supplementary Figure 5. Mapping of the three novel satDNAs onto PacBio reads (accession number SRX26679649) revealed their tandem arrangements, confirming them as true satDNAs, (a) Sat-307, (b) Sat-597, and (c) Sat-208. The names of the satDNAs, along with the corresponding read accession numbers, are indicated directly in the images. For each analysis, the reads with the highest similarity were selected through BLAST (megablast) using trimers of each satDNA (left panel). Specific regions showing tandem repeat accumulation are enlarged in the right panel.

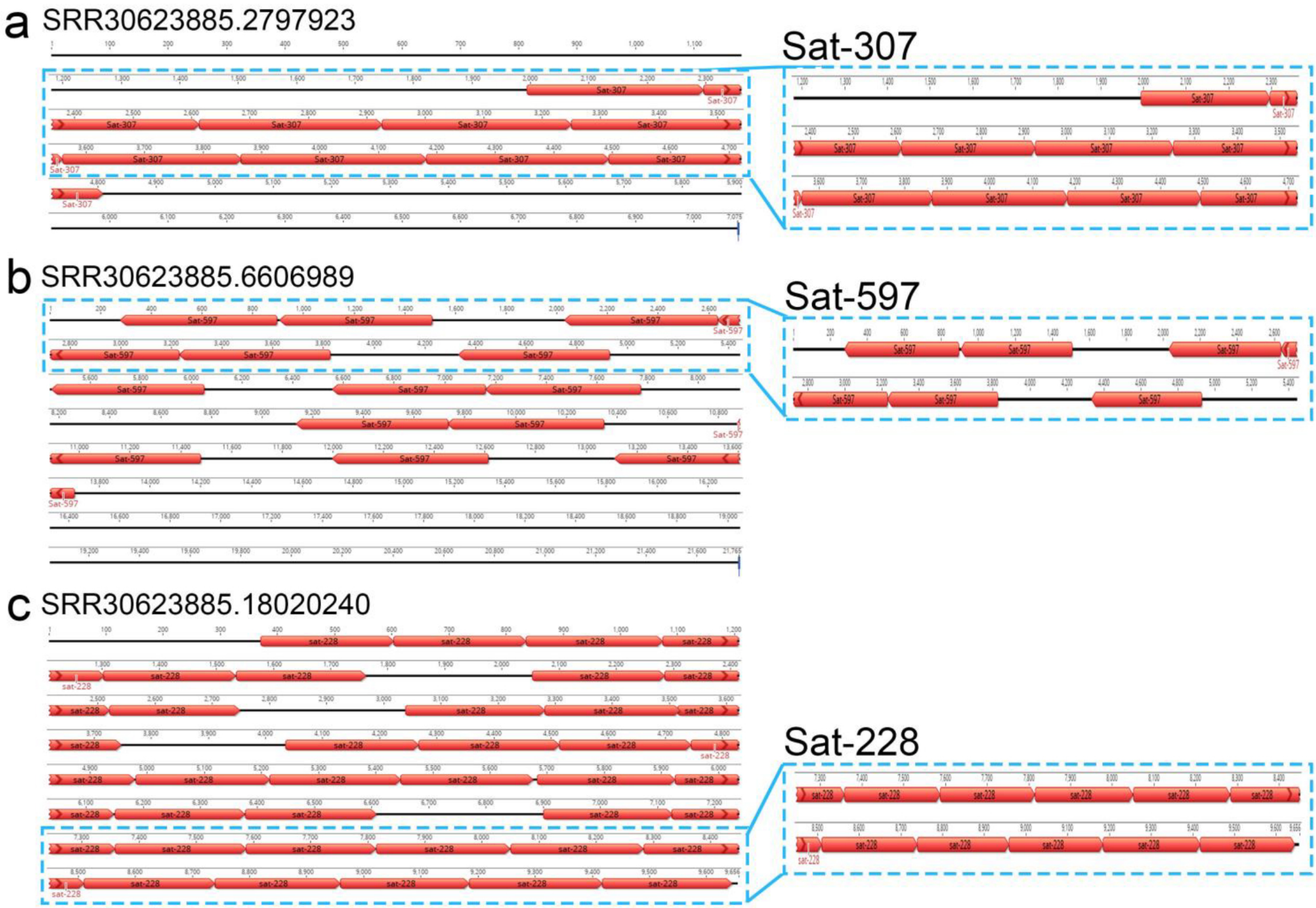

Supplement: Supplementary file 2 [file iyag113_supplementary_data.zip › Supplementary_Figure_5_GENETICS-2026-309266.pdf]
