# Supplementary material for: Comparative genomic analysis and epigenetic profiling of the Drosophila melanogaster B chromosome
Source: Genetics. 2026 May 5;233(4):iyag113. doi: 10.1093/genetics/iyag113 (PMC13439713; doi:10.1093/genetics/iyag113)

Supplementary Figure 6. Tandem pattern of the Sat-228 satellite DNA within the protein-coding gene Ankyrin 2 (FlyBase ID: FBgn0261788), also present in two of its transcripts: *Ank2-RU* (FlyBase ID: FBtr0303125) and *Ank2-RAA* (FlyBase ID: FBtr0344224), with the same array pattern observed in the gene.

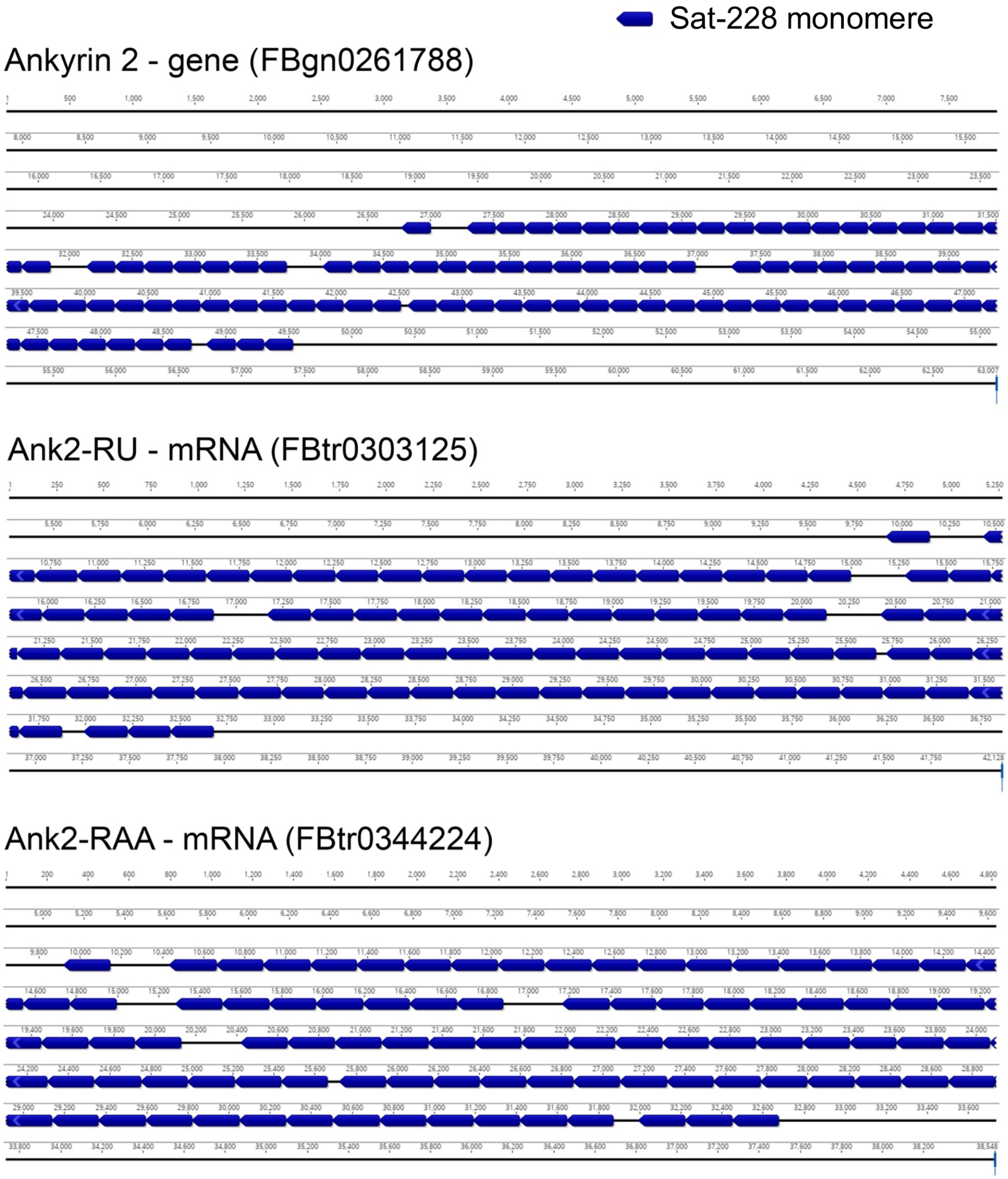

Supplement: Supplementary file 2 [file iyag113_supplementary_data.zip › Supplementary_Figure_6_GENETICS-2026-309266.pdf]
