# Supplementary material for: Comparative genomic analysis and epigenetic profiling of the Drosophila melanogaster B chromosome
Source: Genetics. 2026 May 5;233(4):iyag113. doi: 10.1093/genetics/iyag113 (PMC13439713; doi:10.1093/genetics/iyag113)
Supplement: Supplementary file 2 [file iyag113_supplementary_data.zip › Supplementary_Figure_7_GENETICS-2026-309266.pdf]

Supplementary Figure 7. Dotplot images with lined squares highlighting the *Ank2* repeat in each polypeptide sequence. For the two polypeptides translated from the isoforms containing Sat-228 (*Ank2-PAA*: FBpp0310630 and *Ank2-PU*: FBpp0292244), purple-lined squares highlight the satDNA repeat. Note the presence of the *Ank2* repeat, independent of the presence of the satDNA repeat.

Ank2-PAA: FBpp0310630

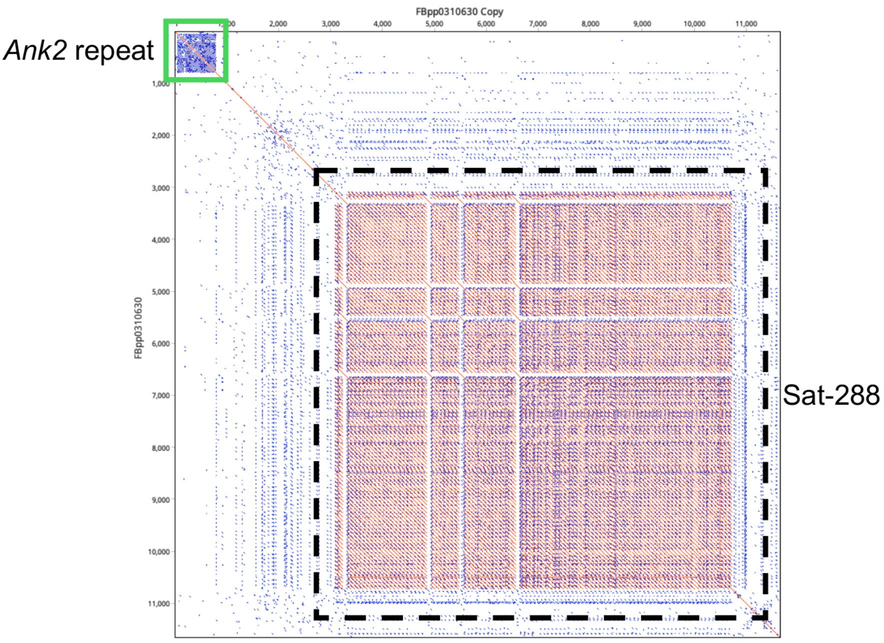

Ank2-PU: FBpp0292244

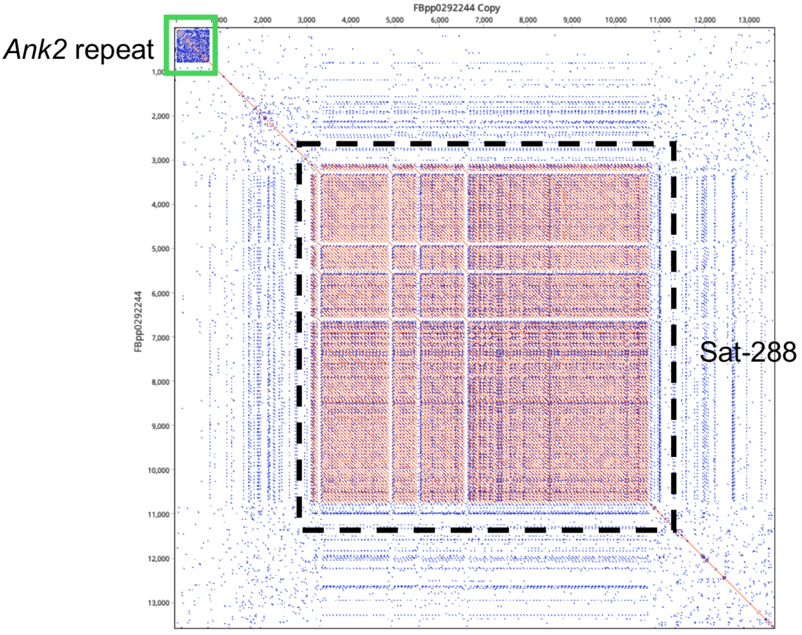

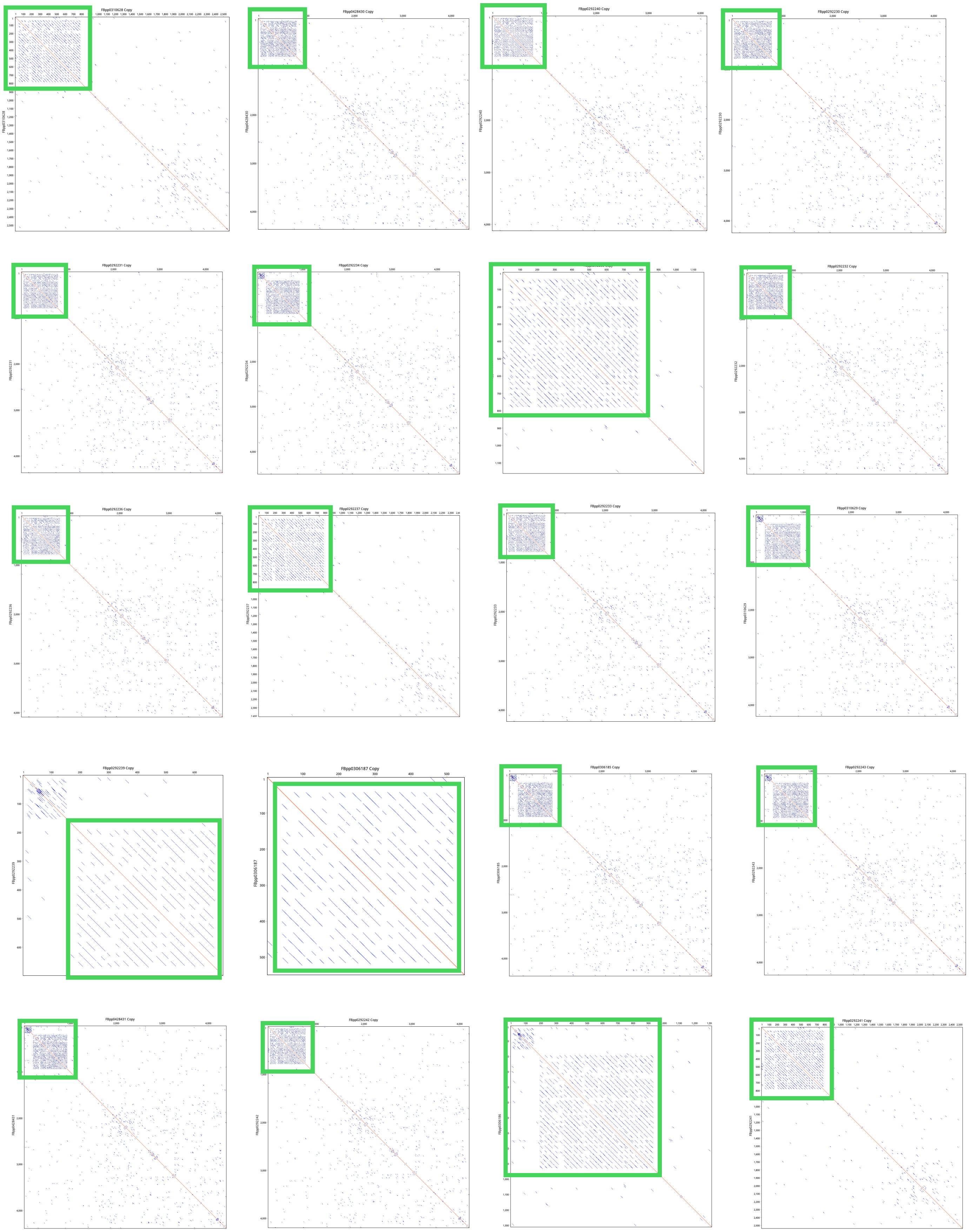
FBpp0310628 Copy
FBpp0428430 Copy
FBpp0292240 Copy
FBpp0292230 Copy
FBpp0292231 Copy
FBpp0292234 Copy
FBpp0292232 Copy
FBpp0292236 Copy
FBpp0292237 Copy
FBpp0292233 Copy
FBpp0310629 Copy
FBpp0292239 Copy
FBpp0306187 Copy
FBpp0306185 Copy
FBpp0292243 Copy
FBpp0428431 Copy
FBpp0292242 Copy
FBpp0292241 Copy
