# Supplementary material for: Comparative genomic analysis and epigenetic profiling of the Drosophila melanogaster B chromosome
Source: Genetics. 2026 May 5;233(4):iyag113. doi: 10.1093/genetics/iyag113 (PMC13439713; doi:10.1093/genetics/iyag113)
Supplement: Supplementary file 2 [file iyag113_supplementary_data.zip › Supplementary_File_1_GENETICS-2026-309266.pdf]

Supplementary File 1. FASTA file containing monomers of all the repetitive sequences used for quantification in the genomes of D. melanogaster analyzed.

>ZAM_LTRGypsy#LTR/ZAM_LTRGypsy

AGTTACCGACCCATCGGTACCATACACCACCCCTCCCTCTAAGCCACCACGCC
TACACAAGTAGAAGACATCGAACCGGGAAGCTTTGCGATACAAAGTTGCAG
CATAAACATCAACAACGGGTCAGACGCCGACATCCGCCCAAAATGCTGACAC
CACATCCTTTTCGCTCAGACAGAACAACGCATACAATTCCATATACATACGTAT
AAACATACTCATACTTTCTGCTGTGTCAGATACTTTATTTCTAAGAACTTTAAC
ATTGTAATACATACACACATATTCACTGTTAGCCCATTTAAGACGAAGAATAA
AGACGACCACAGTCGAGTGCAAGCAGCAAACACTTGTAGACGTACATAATCT
CCGATCAAAATTCTCCCAAGACGACCGTGGCTACGTTCTGGACCCGCATAAC
TCCTCTATCTTTCTGAGTGATAATACCTCCGCAAGACTCCCCGGAGGTAAC

>ZAM_IGypsy#LTR/ZAM_IGypsy

TGGCGCAGCCGGAAAACTGGAATGGAAAATACTTTATTAAACCTTCTATTAGT
TCTATTGTAAGTAGTTGTGGAAAAAGAGTGAGAATGAAGTGCAGAAATGTCT
AAAAGTGATTACAACAAAAATCCTAATACAATACATAAACCGCCTTAACAAA
CATACAAAACACGCATATAAAAAAAAAAAAAAAAAAAAGAAAAAAAA
AAACCCAAAACTTAAAAATGCCGTAACCGCGAAACATGATATGCGTTGTACT
TGTGTGAAATCAATCGCTGATAGTCACTGCCGAAGTTTATTAAGGCCAAGTAC
CATATCATTACTTTCATGTTTACATACATATATATGCCCCACAATTAAAACAACA
TACACACACACAAATATTTCAAATGCAAAAAAAAAAAAAAGAATGTAGTGTA
CCTGCGTGGCATCAATCGCTGATAAACCACTGCCGAAATATTAAAGGCCCGGT
ACTACATCACAAAACACGTATATATGCAACAAAAATATACACAACAAAACCAT
ATATACAAACGTGTATGAGTGACGTGTAATGTACTTGTGTGAAATCAATCGCT
GATAATCACTGCCGAAGCTTAGTAAGGCCAAGTACCACATCATTACTAACATG
TGTACATATATATATATGCAAAACAATTAAAACAACATACACACACACAAATAT
TTCAAATGCAAAAAAAAAAAAAAAAAGAGGAAATGTTGTGTACCTGCGTGGC
ATCAATCGCTGATAAACCACTGCCGAAATATTAAAGGCCCGGTACTACATCAC
AAAACACGTATATATGCAACAAAAATATACACAACAAAACCATATATACAAAC
GTGTATGAGTGACGTGTAATGTACTTGTGTGAAATCAATCGCTGATAATCACT
GCCGAAGCTTAGTAAGGCCAAGTACCACATCATTACTAACATGTGTACATATA
TATATGCAAACCACCAAAACAAATACATATACACATACAAACACTCCAAAAA
AAAAAACAAATAATACTATATGAACGGCGAAGCGTATGTTTTCTAAGGCTGGA
TACAAAACCACAAAACCAAATATAAATTGCACACCTTAATAAAGAAAAGAAC
AAAAATGATAATAAACAAAAGAAATTTTTTTTGGAACATGCACCCATACTCTC
ACTCTTTCAACACAAATAAAGTATTCAAATTATACATACATACAATAATACCAC
TATATTACAGAAATTAACGCACAAGAAAACACACACACTATCCAACAACAAA
CAAGTAATTAAGAGTTATTAAGTACATTGTAAACTACATATTTTTATCTTAAATG
TCAAAGAAATTAACACAAACTATTAAACAAACAACTCGCTCCGTGTTAGAAT
CACACACATTTCCCAAAAGAGTTACACGATCAGTTTCGAAAACAAACACCCT

CCCCGTAATAAGAGAAAGCACCCCCTTACCGCCCCTTCAACCTATAAATATGG
ATTCGGGCAACGCCTCCGTAGGTAATTCCGCCCCCGTAACACCTACTGTCAGT
GGCTTTAGCAGTATTGCTACGGCACTTAGTGCCACCGATATTTTAGCCTTCGTT
AAAGAACTTCCGACCTTCGATGGTACTCCAGGCCAACTCGACAAATATATAA
CTAGCGTTGAGGAAATAATCATGCTCATTAGGGGTACCGACCAAACTCCGTAC
GGACTTCTGACACTCAGGGCAATTAGGAATAAAATAGTTGGAAGAGCAGACG
AAGCTCTAAACCTAGCCAACACCAAACTTATATGGGACGATATCAAAAGTAA
CCTACTACGTTTATACTCTAGCAAGAAAAGCGAAGCTACCCTCTTAGGCGAG
CTCCAATCTCTCCCAGATAACCTAACCCTAGGGCAATTGTTCTTCGGCTTATC
GAGGATTAGGAGCCAACTTATATCCATTACTTCCAATAGTGGACAGTCGGCCA
CAATCATCGAAGCCAAGAAAACACTATATGACGAAGTCTGTTTAAACGCCTT
CATCTCAAGAATTAGAGAACCACTTAAAACAGTCATCAGATTGAAAGACCCC
AAGACTATCGAAACAGCTTACGAGCTATGTCAAGGAGAAAGGGCTCGTTACC
AGAACAGAAACCCATATCCCCCAACACAAAACAACACCGAACGACGAACTA
ACAACTACAATAACAATAACAACAACAATCACAGAGACAACAACAACCGCA
ACAACGTAACTCGTCTTACACCCAAAACCACTCAAACCATTACTCAAACCCC
AATTCCCAATATCGTCAATCAAACAACGGCAACAGAACTAGTAACCCGTTTA
AAGATAATAAAACAAATTATGGGCTACACAACATAGAAGAAGAAAAACTCAC
CCAACACTGCCTTACCAACTTAAATTTTCAGGCACCCGCCTCAGGAACCCAA
CAGGATACATAAATCCTACCACACATGCAACATCCCTTCCATACATAACTCTAA
ACCTCCAACAAAAATTCCCTTTATCATTTCTTATCGATACAGGATCCAATAACT
CCTTCATTGACCCAGAATCTGCAAACCAACTAGAGTGCACAATTCTACCAAC
ATCCACTTCAATTACAACAGCATTAAATAGTTTCAAAATTGAAGAAAAGGCA
ATATTCCCAATGCCACCCGAGTTCAAAACCGAAGGTCAAATTACCCTACTTAA
ATTCAAATTTCACTCTTATTTCAATGGCCTCATAGGAATGGACCTATTATCACA
CCTAGAAGCAAAAGTAGACCTAGTAAACTTACAACTAGTAACTTCAAAGTCT
ACACTCCCAATATTCTTATACACTAACCAGGCTTCAAAAATTTTTAACATCCCC
GCCTACAGTAAAGTTATCTTACCACTACCAGTAAAGACTAATCATGGGGAATT
CTATTGTTGTACTACACAACTAAATAATGAGTTATCGTTGTCAGAAGGACTATA
TAAATCAAACAATAATATTGCCAATGTCGAAATCTCTAACCAATCCGACTCAG
ATAAACTATTATACCTAGAATACCCCCTAGAAACCATTCCATACAATAAAAACG
ACCATATAGAGCTCTTTAATATATCAGCTACACCTCTTAATAACGATACCCCTC
AAGCCCCATTACATATCCTCACAGAACACCTCAATCCAGAGGAAAAAACAGC
CTTAACAACCCTATGTAAACAATTTCGCGACATATTCTACAACCCAGAAACAC
CATTAACTTTTACCAACAAAATCACACACTCCATCCCAACCATAGATAACACT
CCTATCCACACAAAATCCTACAGATACCCTTTTGTCCATAAAACAGAAGTCAA
AAAACAAATCGAATCCATGTTAGACCAACAAATTATTAGATCTAGCCACTCCC
CTTGGAGCGCCCCGGTGTGGGTGGTCCCAAAAAAACTAGACGGGACAGGGA
ACAGGAAATGGCGACTTGTAATAGACTACCGGAAACTCAACGACAAAACCAT
TTCGGACAGATACCCCATCCCAAACATAAATGACATATTAGATAGCATAGGCA
AAGCAAAATATTTCTCAACGCTCGACCTAACTAGCGGTTTTCATCAAATCGAG
ATGAATCCAAAAGATATCGCCAAAACAGCCTTTACAGTCGAAGGGGGTCACT
ACGAATTCACACGGATGCCCTTCGGCTTAAAAAACGCACCGGCTACCTTTCA

ACGGGTTATGGACAGCGTTCTTGGCGATCTCAACGGCACCATTTGCCTATTCT
ATCTTGACGATATTATAATTTTCTCGCCTTCCCTACAAAAACACCTGTTGGACA
TAAAAATGGTATTCGAAAAACTCAGAGCGGCAAACTTTAAACTACAACCTTC
AAAATCAGAATTCCTAAGGAAAGAGATAGAATTTCTAGGCCACATAGTCACA
CAAGACGGAGTTAAACCAAACCCGAACAAAATAAGTGCGATCAAAAAATTT
CCTTGCCCCACCAACAGAAGAGCTATCAAATCTTTTCTCGGGTTACTGGGTTA
TTATAGGAAGTTTATAAGAGACTTTGCACGAATAACGAAGCCCATGACTAAAC
AATTGAAAGGGAAAAGACAAGTTACTACAGACAAAGACTTTGTAGACGCAT
TCGAACAGTGCAAAACTCTTCTGTCCAATGACCCAATACTCATACACCCAGA
CTTCGAAAAACCATTCATTCTTACTACGGATGCTAGTAACTTCGCGTTAGGAG
CCGTACTATCTCAAGGCTCCTTACAAAACGATAGACCTGTATGTTTTGCCAGC
AGGACCCTCTCCGACACCGAAGTCAACTATTCAACCATAGAAAAAGAAATGT
TGGCAATAATATGGGCAGTAAAATACTTCAGACCATATATTTATGGCGTAAAAT
TTACTATTGTTACAGATCACAAGCCACTAATATGGCTTATGAATTTCAAAGAAC
CCAACTCAAAAATAATTCGTTGGAGACTCCAACTCATGGAATACAATTTTGAA
ATAATTCACAAGAAAGGTTCACAAAATGTAATTGCAGACGCCTTAAGTAGAG
CGGACCCAAATTTAAACTACAACGAAACACTGACTGTTAAGCCTTGCCCCAC
ATCCGAAAAACCTATTAACGAATTTAACACGCAACTCATACTAGAAATAGATA
CAAATACGTCTTACCAAACTACAACACCATTTAAACAAAAGATTAGGAAAAA
ATATTCACAGCCTTGCTTCGATTTCGATAATATTGTTAAAATCTTGAAAGGAAC
CCTAAAACCTAACAGGATTTGCGCATTCTTGGCGGACGATAATAATTCCGCAT
TAATCGAAAAAGCATTCTCAACGTATTTTGCACATAAAAAACACTTTAAAATT
ATCAGATGCAAATCACTTCTCCACGAAATCGTAGGAAACCCCGAACAAAACA
AATTCATTCAGGAATATCACACTAACAGCAACCACAGAGGCATAGACGAAAC
ATTCCTTCACCTCAAACGAGAAACCTACTTCCCCAATATGAAAAACAAAATC
TCTGAATTAATTAGGAATTGCGAAACCTGTCTAAAACTCAAATACGACAGGC
AACCACAAAATATAGTATTTGAAACCCCAGAAACCCCATCGAAACCCCTCGA
CATAATACACATAGACATCTATACTATTAACAATAATTTTAACCTGACAATCATA
GACAAATTCTCAAAATTCGCAGCTGTCTACCCCATCCCAAATAGAAACGGCA
TCAATTGCATCAAAGCAATCAAAAATTTTTTCAGTCAATTCGGACTACCCAAA
AAACTAATACACGACCAAGGAGTAGAATTTTGCAACGACATATTTCGAAAGT
TTTGCTCTCAATATAATATACTTCTCCATGTCACATCCTTCCAGCAATCTTCAA
GTAATTCTCCAGTAGAACGTTTACACTCCTCTTTGACAGAGATTTACAGAATA
ATACTAGACACACGGAAAAAACACAAATTACCTACAGACCACGAAGAAATA
ATGTCAGAAACTGTAATAACATATAACAACGCAATCCACTCCACCACCAAAC
ACACCCCTTTTGAACTTTTTAATGGTAGGACCCATTTATTCGAGAAAACAATA
ATACCCAATAATGAGCATGACTATTTAAATAAACTAAATACGTTCCAAGACAA
ACTATACTCCGAAATAAAAGAAAAATTGTCCACAAACACCCAACAAAGGATA
GAAAAGCTAAACACAAGCAGAGTAGAACCAACAACAGTACAACCTAACAGC
ACAATTTTCAGAAAAGAAAACAGGAGAAATAAATTAACACCACGGTTTTCCT
TACACAGAACAGCAAAAGACAAAGGAAAAACTCTAGTAACCACAAGAAATC
AAAAAATCCACAAATCAAAAATTAGGAAAATATCCAAACCTCCAAATGACTT
AAGCCTTTCCACCTGCATTCCAGATCTTGCCATGGGGCATACCAATCTATCTTC

ATCCACAACTTCAATAGCACCAACCTCCTAGCAAAAGTGCCGGTAGGGAAAA
CACTCGTGATAGGAAACTATAAAAAAATTAGCCACATAATCGATCTGTCCGAA
TACACCAACTGTATTGAAAAATTATACCACACCATCGATACCCTAAGACAAGA
TGAAACACTCACCGATTCTATATCAATACTAAATGCTAAACTGGCCCAAACTC
AAAGTAAAATAGACGCACTAACACCCTTTTCACGCCACAAAAGAGGTCTTAT
TAACGGGTTAGGTAGTTTAGTCAAAGTCGTCACCGGCAACATGGACGCCAAT
GATGCAAAGAATATAGAAACAGAAATTAACCACTTAAAAAGCCAGTCCACCA
CTATCTCAGATAACTTCGAAATACAGAACTCGTTCAATGATGAAGTTCAACTA
CGGTTCAAAAACTTAACAAGACACATTAACAATGAACAGAATTTGATTAAAA
ACTTCTTCGAAAACACTCAAAATACAATTTACACAAAAATATACAACAACGA
AGAAGAAATAAAGAAACTACAATATATAAATAGGCTTAACTACAATATAGATT
TATTAGTTAGCCACCTAAGCGACATTATAGAAAGTACACTGCTTGCCAAAATT
AATGTTATCCCAAAACTCATCTTAGACAAGACAGAAATAACCAAAATCAAAC
AAATTTTTAAAACACAAAACTACACAATAAAATCCGAGCAACACATTTATAAC
CTCTTAAAAATGAACGCACTCAATTACCAAAACAAAATAATTTTAGTATCAA
AATTCCTATTTTTTTAAGTTGTAACTACGAAATGGCAAGATTAATTCCACTTCC
AATAAATTCCACACAATTTGTAATAGCACCTAAGTACTTAATATATAATAACAA
AAGTAACAGCATGTTTTCAACTATGTATAAATGTCCTGTAATAGAAGAACAAT
TCGTCTGCGAAATCGACTCCATCAATAATCTTAAAAATAATACTTGCCTGGGA
CACCTTATCCAAAATAAGACCAGCTACTGCGACATAAAGGAAACGGGACTCA
CGACTGATGTGTTCGAACCGGAAAAAGGCTTCATACTTGTATTTAACGGGAA
CAACCTCCCAATCATCTCCTCCAACCAGACCATAACTAGTATCAATGGATCAG
CTATAATAAAGTATAACAATTGCACATTAAAAATCAATGAAATAAACTACGAC
AACAGGGCGGTATCAACAGAAGAGCACCCCGACTTCTTCCTACCACCAATGC
GGAAACTAATAAAAAATGCCACTATCAACATACTCACCTTGGAAAGACTTCA
CCTGGATACACTCACAACATCCAATAAGCTACTGGTCGTCGCCGCAGGAAAC
TCTCGACACTCGACAACCTTGTATATCCTCTTCACCGTATCCCTAGTCGCCGTA
ATACTCACCTGGACACTTCGAAGGGACACCCACATCTTCCATACCGGGCCCG
ACCACATTCTTCCAATCGTCGCTCCACCAATTCCTCCGTCTATGGCCTTCGCTC
CAAACTGGGGGGGGAGG

>TRANSPAC_IGypsy#DNA/TRANSPAC_IGypsy

GGCGACGAGGATCAACACAGAAAAAATAAGGAAACCAGCCATACGTCAAGA
AATTCATTCNGAACCACAGTNAGCTGNTNTTATGTAATTTAAGTCCGGAAATC
TTCAATACGTTAACCAATGCACGTGGCCGAGTATAAAGAGGAACATAAAACA
TAACTGGAAAGCTTTGGAACACCTGCATGTACGGAGTGACGAAACCCAACC
TGGAGAAGAAGAACTTAAGCTGATAAAATTTTTTTTTCTTCGACAAAGCTCT
TTATACTAGCTAAGATAAGAAACTAAAAGCTAACTATTAATACTTGCGAACCT
CGAGAGAAAACAAAGAAATCTAAAATGAACGAAAATAGCTTAGCCGCATTTA
ACTCAACAATGAACTGCATCAAGTTAATACAAAATTTTGATGGTACTGATACA
AATCAATTGCCAGATTTAATTACACAAATAGAAAATATCCTTCCTTCTTTTGAT
GTTTTCGACACGAATACAAAAAATATTTTGTTTGGCTTTATAAAAAATAAATTT
GTGGGATTATGCAGGCCAGTGATTCATAGGCATGGAAATATGACAGAGTGGG

AAAGTTTTAAACGTGTAATTCTGAAAAATTTCGGGGAAAAAGAAACAAGCG
ATGTCTTAATGGATATGTTAAAATTGTGTCGAGTGGAATCAACTATAGAAGAA
TATTATAACAAAATTAATGAAATTTCGAATAGGGTACACAACCGGATACTGAT
CCATGACGACAAAACATACACCACATTAGAAGTGAACCGAATTGCACTCCGC
GTTTTTAGAGACAACTTGCCTGAACCTACAAAAACGTTAATATTCGCGAGAA
ACCCAAATTCCGTAGAGGATGCCTATAAAATCATTGAAGATGCTCGGCATCAA
AGCTACACATTATACGGACCAATTCGAAGAAATAATAGGTACAATAAACCCAA
CTTCAGAACCAATTTCAGTAATGATAATAGAAATGTAAACGAACCAGTCGTTA
CAGAAAGAAGCCTTGATGAAGCCAATAGGTTTCAACAAAATAATGTTGAACA
ACAAGAAAATTCAAGAACTGAAGGTACGCCTAGGGTAAATAATAGAAGGCA
GGGATATCAATATCAATCGAATAATAGCTCAACTTCTAATTCTGCTCGAGTCTC
AACAAATAGTCGAAATGTGCAAAGCTCTGAGCAAAGCAGGCGTAGTTTCGA
GCCAATGGATATTAATCAATCAAGTGTAAATTTTCAGATAGAGGATCAAGACG
AATACCATATATAGAAGTTCAAGGTAAAAAGAGGCCATTATTATTTATTATTGA
CACTGGAGCAGAATTCAGTGTAATAAATGATAATTTGTGTCACCCGAAGTGG
AAAACAGACATTGACATAGAAGTAAAAGCAATGGGAAACAAAATTAAAATA
AGAAAATTATGCAGGTTTCCATGTTTTCAAGAATTTGAAGCTGAAGGTTATAT
TTGTGAATTTCTAGAGTATAATTTTCACAACTATTTCGATGGGCTAATTGGCTG
TAACATACTTAATGACTTGCATGCAGTCATCGATTACTCAAACAAAAAGATTC
AACTCAATAATGTTTATTTAGATCTTTTAGTTCAGGAAAACCACTTGGATCTTG
GCATACATAACTTAATTCCAATGAACTTAATTTGTCCACAGGCCAGATCCTCA
ATAAATGAGCTTATACAAAAATTCGAACATATTTTTTACAAAGAAGGCCAAGA
CCTAAGTTTCACAAGTGAAATCAAACACAGGATTATTACAAAGAATGATCTTC
CCATTTATTCGAAAACATACAAATACCCAGAAATTCACAAAGACGAAGTTAA
CCGGCAAATAGCAGAAATGTTAGATCAAGGAATTATAAGACACTCTAAAAGC
CCATATAATAGTCCTTTGTGGGTTGTTCAGAAAAAAATGGACCAGTCCAATAA
GCAAAAGTGGCGGCTCGTCGTAGACTACCGTAAACTTAATAAGGAAACCATT
GAAGATCGATTCCCGATCCCAAATATAGACGAGATTTTCGACAAATTGGGCGA
CTGTAAAATTTTTTCAACTTTAGACCTTGCAAAAGGTTTCTATCAGATAGAAA
TGGACAACAAAGATGTGCACAAAACAGCCTTTTCAACAACCAGTGGCCATTA
CGAGTTCCTTCGTATGCCTTTCGGACTACGGAACGCACCTTCAACATTTCAAA
GGCTAATGAATAATATCCTTAGCCCCTATACCGGACAATTTTGCATTGTTTACA
TGGATGACATCCTTATATTTTCTAAGAATATAGGGGAACACGTCAATCATTTAA
GCTGTATCTTCAGCTGTCTATCTAAGGCAAATTTAAAACTCCAATCAGACAAA
TGCGAGTTTGCCAGAGAGGAGATTGAATTTCTCGGACATACCATTAATTCAGA
AGGTCTAAAGCCAAGTCAGAAGAAAATTGACGCAATTTGTAAGATAAATTTA
CCGACAAACCAGAAACAAATTAAAAGTTTTCTAGGCATCACAGGTTATTTGA
GGCGCTACATAAAAGACTACTCCAAAATTGCCCAGCCCTTAATAAAATACTTA
AAGAAAAATTCTAAAATAAACACACATGATCAANAATACGTAGAAGCTTTTC
AAAAACTAAAAATCCTAATAACAAGCGATCCGATAGTCGTTTACCCAGATTTT
AACAAACAATTCACGATAGTTACAGACGCAAGTAACTATGCATTAGGCGCCG
TGCTCATGCAAGATAATAAAGTAATTTCTTATGCTAGTCGTTCTCTTAAAAATC
ATGAACTTAATTACAGTACAATAGAAAAAGAGCTTCTTGCAATCTATTGGTCA

ACAAAATTCTTTAAATATTATATTTATGGAAGAAAGTTTATAATTAAAACCGAC
CATAGACCCCTAGTTTGGCTTAATAATCTAAAGGAACCCAACCTAAAGTTACA
ACGTTGGAAAGTGCAATTAAACGAGTTTGATTTTGACATAACATTTATAAAAG
GTAAAGAAAACGCTTTAGCAGACGGACTTAGCAGAATTGTCAAAGCATCAAC
AGAAGAGTATGATAAAAATGCCCAAACAAATATAGAAATTAATAACTTAGATA
TATTTATGCTAGAGAACACTAACACATTACCGGAAGTTACGCCTAAGACAAA
AATTATAAATGAAGATTTCTCGAAAGATCTAATTCATATATTTGCCAACGATATT
GACGATTTGGAAACTATTCACAGTGCGGACTCAGACGATCTTAATTTTATTAG
TATTACAACTAATTGTCTAAACGTTTTTAAAATCCAAATACAAATAATAGAAGC
GGAAAGCGATTTAAGTACCTTCAAAATTTTGCATAACAAGAAAATACGACATA
TCTTTAAATCAAGCGGAAATCAGGAAAATATGCTTAAGTTCATGCAAGAGAA
ACTGCCAGAAAAAGGCTTAGTTGTAATATTTTGTGAAAATTTAAGTTTATTTG
TAAAATTTCAAGAAATTTATAGGCAGTATTTTTCAAGTAATAAAAATTTAAGA
ATTCTAAAGTCAGGTACATTGTTAGAAGACATAAATGATAAAGAAAAACTCCT
GAAGATTATCGAAAACGAACACATAAAGAATAATCACAGAGGCATTAACGAA
ATTTTTATATCTATAAGAGAAAAATACTATTATCCAAAAATGCAAAAGTTTATT
CAAAATTATATTAACAATTGTAAAATTTGTAATTTAGCCAAATATGATAGACAA
CCTATAAAATATAATTTTAATATTACAGAAACGCCAGATAAAATAAACGATATA
ATTCACATAGACATCTGGTATCCTAAAAGAAACATTATGTACGTTACATCAATT
GACAAAATGTCTAAATATGCCACTGCTCAACATATTAAAGACAGATCTTGGAT
TTCTTTGCTTAACGCAATCAAATTAAGAATTCAATACTTAGGAAAGCCTAAAA
AGATTGTAACTGATAACGAATTCGATATTGTCGTAATTAAGCAATTTCTCCTCG
AAAATAACATAGATATACACTTTACAACTCCTTATAAGAAAACTGGAAACTCT
GATGTGGAAAGATTGCACTTAACCCTAAATGAACATATGCGATTGTATAATGC
TGATCCAAACAATTTTGACACTATTCAAGAAAATGTTTATAAAGCAATTGTTT
GCTATAATAACACAATTCATTCAACCACAAATATTAGACCGATAGATTTATTTA
ATAATATACTTTGTCACGAAGACATCTCTAAACTATCCGAAAAGCTAGTAAGA
AACAAACAAACTTTAAATGCAAAACAGGATAACATAAACGAGTCAAACTTCT
CAAATATATTTGTAAAAAATAATCAAGTAGGAAAATCAAATCCAAAATATAAA
GAAATAAGTAATTACACTCAATACGGTAACTACTTGATTAATAATAAAGACAA
TAAACGAAAAATCTATAAGGATCAGGTGAAACGGAAATATAAATATCAAAATG
AAGATTGTGAAACCTAACATTTCTGTACTCCTATTATTTACATATGTATTACCTA
ATGAAATAATTTCAACCACATACTTGAAACTTANGCTGGGGGA

>Transib5Transib#DNA/Transib5Transib

CACAGTGGTTCCGTCAGAGGCCAAAAATAAAAAAAAACATCTAAATATATTT
ACGCATAAAGTAACCAAATTGTCTCCAAATAGTCAAAATATTTCCAGCCAAAA
CCAGTTTTTGCTTCAAACCTAACGGTACTTTCTAAAAATAGAATTAAAGTTAT
GCACACGTGTTCATTTGTGTAGCGAAATTGCACGCATTGTAATTTCTGCATTC
CAAAAGCAAACTTCAAAGGCATCATACTTTCAGTGAGGAGCTTCGATTTAAA
TAATTTAAAATGGATTTTAAAGTAGAAGGTATTTACTTTGTATACAAAAACTAA
TAATTAACTAATCAGTTAACTAAGTTAAGTAAAATTAATATCTTCAAATGGCCT
TTTTATTGTCTTTTTTTGTGGGTATTTTCATGTTTACTTAAACTTTTAGTTATGTT

CCTTAAATATTAAATTAAGCATAGTTTTAAGATAATAAAAGTGTTGAGTTCAGC
TGCGGAAACTTGCGGATGTGCTTGTAATATTGAATCTACTAAACGCCCATATTT
GTTATTTTTGGGTTAAGTTTGCCCAGTGAAGTCCCTTTGTGTCTTTGTTTTTGT
GTTTCGCGTTTGTGTCGTTTTGTCTTTTGTGTTTGTGTCGTTTTGTCTTTTGTG
TTTGTGTATAATCTTCCATGTTAATTTAACTTTTATTATATTAAACAAGTAATTAT
ATTGATTTGTTTAAATTTATTTTTGCTTATGGAAGACGATTCAGAGCTCTGTGT
GTTTCCACACCTATATATTTTTGATTTATGGTGCACAAAGAAGGGTACCCACG
GAGAAAAACTGAATGTGTTGAAAAAATTTGTAAACAAATTGTTTATTTTTGG
ACTCGCTACAATCGGAAAAAAGCTTCCTTCCTTAAGTACCAATGTGAATGGCT
GAATCAAAAGAAAACAATTTTATTAAAATGCAACCATAATATATCTGAGAAAA
TTATGGAAAGTGATCATCGTGGCCGTCCAAAAGTAGATTTCTCGATGTCATCA
TCAAGATCAAAGCGACGCCGTTTAGCTGAACTGGCCAAAATAGATGAGACCG
CTGTATCTGCTTTACTCAATGAATTTGAAAAAAAAAAATTATTGCCAGCAGAT
CCGATTGAAATAATTTCGCTATTAATTGAGGCAAGCTTGTCTAAAAACCAATAT
CTTTTGATAAGAAACTTTGTAAACACCAAAATAGGCTTTGATTTATTTCCAAG
TTATCAAAGTGTACTACATTCAAAGAAAATGAGATACCCTGAAAATATTTTCG
TTGATGAATCTCACGCTGAAGTGGAGTTACAGAGCTTATTAAATAACACAGC
GTCAAGTATATTGGAGCTTCAAGCTGAGGTAGTCGAAACACTCACTGATAATT
GTGTTAAAAATCTTTTATTAATTGGAAAGTGGGGATTTGATGGAAGTACCGGT
CATAGCGAATATAATCAAAAGTTTTCCAATAATGATTTACAGGATAAAAGTTTA
TTTGTCACATCCTACGTACCTCTCCAACTAATTTCAAAAGATAACCAACAAAT
AATTTGGAAAAACAACAGACCATCGTCAACACGTTACTGCAGGCCAGTACG
ATTTCAATTTAAAAAAGAGACAACAAAACTTTCAATCGAAGAGGAAAAGTAT
TTTAAGGAAAAAATAAACCTTCTGAATCCAACTGAAGTATTTATAAATGATAA
AAAGATAAATGTTGAACATAGCTTACAACTAACCATGGTGGATGGGAAAATAT
GCAACGCCTTAAGTGAATCTTCGTCTATGAAATGTTACTTATGTGGAGCAAGT
CCCACAGAAATGAATAACTTGGCAAAATGTATGAGTAAAGACGTAAAAACTG
AATATTTTGAATTTGGTTTATCTCCACTTCATGCGAAAATTCGATTTTTCGAGT
ATTTTTTACATCTTTCCTATAAGTCCGAAATAAAAATGTGGCAGGTGAGAGGT
TCTGACCACAAAATAAAAGTAATGAAAAGAAAGCAAAAAATACAGAAAGAA
TTTCGCGAAAAAATGGGAATCATAGTAGATAAGCCTAGAGATGGTGGTAAAG
GTTCTTCCAACGACGGAAATGTGGCAAGAAAATTTTTTTCTAATCCAAAATTG
GCTTCAAAAATTACCGGAATAAATGAAAATTTGATATATCGTTGTGCCACAAT
ACTACAAGCTATTGCATCGGGGTATAAAATTAATATTAACAAATTTAATACATAT
GCCCTCGATACAGCAAAGGAGTTGATCAATGAGTATCCATGGTACTATTTGCC
AGCTACAGTCCATAAGGTGCTAATTCATGGATCTGCAGTAATACAGCATGCGT
TAGTGTCAATTGGAGAACTTTCGGAGGAAGCTGCGGAATCTAACAATAAAGA
ATTAAAAAAATGTAGACTTAATCACAGCCGGAAGATGTCACGCATTACAACA
AATGCAGACATTATGAATTCACTTCTAATAAGGTCCGACCCATTTATAAGCGG
ACACAGAAAGCTGGGAAAAAAGGATAAGTCAGCGTTATTTGAGTCTGTATAT
GCCTTACTAGAGGAGGGGTAATAATTATTATCATATTGTTCATTATCATTAATTT
AAATTAATAAAAATTGCAACAATTTAACATTTCCTCACTTTGTTTATATTTGTTT
CTGGTTATGGTGGAGTTTGGGGTTTCCCTGGGGGAGGTATATTACGCCAAATC

ATATTCATGTTCTTACTACATTTAAAAAATATTTTTTAGACCATAAGTTATGAT
TTTATCAGTCTTCTATGATGTATGGTCATGTTATATTTGATTTAAAAAAATGCTG
CATACTTCTTCGGGGGGAAGAATATATTGTTTGCCGAAAAAATGGGGTTGGA
GGGCCCCAATCTCAAAAATCTTTTTCGCATGTTTTCATAAGCTCTATTAGATGC
CTAAAACGAAATCTTAGGCTTCTAGCTATATTGGAAGTATTTATGGCCGCTGCT
CCATACAAGCGGAACCACTGTG

>Transib4Transib#DNA/Transib4Transib

CACTATGGGTAAAAAGCCGGAAATTTTGGTATTAATGTAATTTTTTTTGTGATT
GATATTTTAATGCTGCTTTCATTTTTTGATTCCTAAGTAACAAGATAGCATAAA
AATAATTTTAAAACATTTTTACGAGTTATCGTTTATTTTTGGCGCGCTTTCAAA
GTCAGCTGATTCAGAGGTGCTTTGAATTGTGAAAATTGGCGGAAAAAGTTGA
ACAAATTGCAGCGCAAACATTGTCTTCATAGCAAACTTTGAGTTTATTTTTAA
TTATGGAAAAAACAAGTCAGAGTATGTAAATTATTCGTGAATACACGTTTTCC
GATAATTGTGTGTGGTTGTTGTTGAATTGTCCAATGTATTGCTTGTGAGTATTT
TCAATTGTTTACTAACAAACGTTCTAGTGTTCTGTGGAATATTACTTTTGTATG
GCACAACGAATCACCAAAGTTAAAGTTGTATGTTTTTAACAAATATATGTGGA
ATCAAATGGTATTAATGTTTTTTGCCCCTTTTTGCCCTTTTTTGCGAAACATTT
TATGTTGTAGGTGTGTATGAGTTTAGTCATGCAGTTCTGCTGGATGTTTGGAG
CAACAACAAACGCGATGCAGACGCTGTTAACAGTTATATTTTGGAAAATATAG
AAGAGAACTTTGAGACTAAAGAGCAAAAGATTACAATTTCAAAGAAAGTGA
ACCAATTTATTGCTTATGCGAACAAGCACTTAAGGAAATGCAATCGAATGTTG
GACCGATTCAAACGAAATCATTTATTGTGGCTTGCGTCTAAAATTGTTGTCGT
TATTCATAAGCCAGAAATGACACTATGCAAACCTGGCCGAAAACAGTTAACA
TATGAAGATGCAGGGCCTCAACCTAAAAAAAAAATTAGCAAATTAGCTGGCT
GCAGAACAAGGGCATTCAACACCACTTCTCGTACATGCTGCTTCAATTTCCG
CAAAAAAAACGAAGCAGGCAGAAACGGCCTTTGTATTAAAAAAAAAAGCAT
AAGCAGTGAAAACATACATGATATAAAAAAGAAAATTGAGACAAATCATCCT
ATTAAAATAAGTGCTGATAATGCTTTAGCTCTCTTAATTGAAAATGGATTTACA
AAGCAGCAATATACTAATATAAAAGCGCTCAATAAGCAGCAAGGATGCGATAT
TTTCCCTCCTTATTCAAAAGTAGCAGAAGCAAAATTGAAATGCAGGCCTTCC
GAACATAAAATTTCTGAAAAAAGGCCAGGAGTATCGTNGCAAGATTTGCTAG
ATCATACAGCTGGCCACATACTTTTAATACAAGAGNAAGTTTTTTGTGTTCAT
CCAGACCCAACGAATTGTACATTTATTGTGAGCTATGGCTTCGATGGATCTAC
GGACCAAAGCACGTACAAACAACGCTTTCTATCAAACGAATGTATCGCCCTA
GATCAATTCCTTTTCGTCACTACAATTATACCGTTGAAACTAATAGATGAAATC
AACAATCGAGTTATTTGGATACATTCATCTCCACAATCGGTGAGATTTGTCGG
CCTTTGAAAATTGAGTTTATAAAGGAAAGTGCTGCCCTTATATTGAAGGAAAA
ACAAATTTTGGATGCGGCTATAAAAAACCTTAGACCATATTCCTACACTTCCA
AAAACCAAATAATAGCATATACTGCAGACCAGCGTTCGTTCTCGAACAGCAA
GATATTTTCGTCCGTTTTGAATTTGGACTACAAAATCATTTACAATTTTTATATA
ATTTTGATTTCAATTTCTTGTGAGCACACGATTGATGCGGAAAAATTTAAATCT
CTATACCTCTATTCTTGTCACCAACCATCCACAAAATATTGGTACATGGATATC

AAATTATAAACCAATTTATTGTTCCTGTTGGTGTCCTTGGAGAAAACGCGTCA
GAAGCGCGCAACAAACTTTACAAGAGCGATAGAAAATCTCACGCCAGGAAA
TGTAGCCGACTAGCAAACATTACAGATGTTTTCAACAGAGCAGTGGATTCATC
TGATCCTTTGCTTTCAAGTCTTTGTTTAAGAGATCGAAGTAAGTTTGGAAAAA
AGAAACTCCTCCCAAAAGAAGTAATCGATATTCTTCAAAATCCGGATGCAGA
ACTTTATAAATCAAGTTATCATTCTAATAATAAAGAAGAAGAAGTGGAAGTGG
AAGACGAATTCAATTGCCTTAATATATTTTTAGAGGAGGAAGTTGAAGAATGA
CAATCAACTTAATAAAATATAAAAAAATTTATTGACTTTTATCTCATAATAATGG
TTTTTGCATCTTGTGTCCTCACTTCGCTCCCACGCCAGTCCCCCAGGGAAGAC
ACCTCCGGATCCACCGGTTGAACCACAGACATATTAACACCCACACCTCCTT
CGCCACCCATCCATCTCCCTTTAAAACCGCTTTCATTTGTATATAAATACATTTT
TCACTTTATTTTCAAGGATTGTTTTTGTGGGCGGAGATGAAGGCGTGGTCAGA
TCAAAAAATAAACAAAATCCGAAATTCTATTTAAATCCTAATTATAAATATAAA
AAAAATCAAGTCGCTATCTTTAAAATTGTAATTAATACCAAAATTTCCGGCTTT
TTACCCATAGTG

>TRANSIB3Transib#DNA/TRANSIB3Transib

CACACTGGGCCAGATTCCCTTTTTTTTGGCATAAAGTCAAAAAATTTTGTAAA
TTGTATTTTGAATTTAACTATATATTATGAAAGAGCATACATTAGGGACACCAA
AACGATATTTAAAAACATTTAAGTTGCAACACTGCATTTTTTATGCGATATCAA
ATTCATATGTTTGATCAGCTGTTTGTTCAAGCAAAATTGGCGCGAAACTTTTA
CTCAACTTTGTGATTCTGTTAAAAATACACCACGTGTCCAAATATTTTCAAATA
AAATGGAATATAAAGGTCAAGGTATGCTTAATATAGATATAACTTACTGATTTA
GAATTATGTGTTCCATGTTGTAATTGTTGTTGTCTTTGTTTACGTCTTAGTTTAG
GCAAAATCTAACCAAAGTGTTCGTCTATTGTGGAGTGAGAAATGATTTGTGC
CAACTTGTGCCAAAAACTAATAACTGATGTGTTTACTCAAAACTGTAATTAAA
TTGATGTTAGTAACGCCTTTTGCCTAAATTTGCCTATGCTCTTTATATTTTGCCT
TTTAAGACAAGTTTGAGATTAGCCGATCGGTTCTGCTGGATGAATGGATCAAA
GGAGATCGAAATGACAAAGCCTTAGTTGACTGGGTTGTTGGCGAATTTAAAT
TAAATGATATTACTATTGACGAGAAAGCTAAAATCGCTAAGCAAATCAGAAGC
TTTCACCTATATATAATGAAAAATCTGGCTAAATGTAACCGTACAATTGAAGCA
TTAAAAAAAAAACATTCCAGTTGGCTTTCATCCACGATGATGGTTGTCGTGG
ACCAAAACAACAACCAAGTCGCTTCAGACTGTCTAATGGGACGACCTCGAC
TCTCTTACAGTGATGCAGGATCACGGCTCAAAAGAAAAATAGCAACGGATCT
GGCTAATATTGAAGAAAATAATACAAGTCTTCTTGTCCATGCAGCATCAATTT
CAGCCAAAAAGGAAAAAATGGGCGATACTGCTTTTGTATTAAAGCAAACCAT
TTCAACTTCAACTGCATCCACTGAAATTAGAAAGAAATTGATATTCTCAGAGC
CAGTACCGCTCACACCAATTCAGGCTCTTGCATTTTTGATTGACAGCTCTCTA
TCCAAAGCAAAATACAACGACATGAGAGCTTTGAACAAAACACAATTTAGC
AACATTTATCCATCGTATAATAAAGTCAGAGAAGCTAAATTAGCGTGTAGACC
TGCGGGAGTTCATGTGACAGAGACCTTTGCCCAAGTATCTTTCCAAAATTTGC
TGAATCATACGGCCAGTCGAATTATTCTTATGCAAGAGGAGGTTTTTAGGTGG
CATGAAAACATAACAGGTGTCAAGCTGATGGCAAGCTATGGATTTGACGGTA

CTACCAGGCAGATTATGTACAAGCAGAGATTCCTAGAAGATGAATCCCATTTC
AAAGACTCTATTTTCGTTAATAGCCACATTCCAATAAAATTAACAGATGATTTT
GAGACACCTTTATGGATAAATAGAAGCACCCAATCAGTAAGATTTTGCAGAC
CCCTAAAGATTGAATTCGCTAAAGAAACCAAGGAACTTATTTGGAGGAAAA
GTCGAATTTGGAGTCACAAATAAAAAATAACGTCACAGACTTTACTTACAATT
TTTCAATCGACAGAAAAATAGTTACATTTTCCCTTCATCTCACATTAATTGACG
GAGAGGGGTTTGAATATAATAGCTGGAAAGAAATCTTGCCAGGTGTGTCCTAT
CTGTGGAGACAGTCCAATGGATTTTATAAAAACCACCGAAATATCAAATATCA
AGGCTAAAATTGAAAATCGAAATTATTTCATCAGTCCTCTGCACGCATGGATC
AGATTTTTCGAGTTTGTACTCAAAATCTCATACAAACTTAATTTGGAAAAGGG
GCAAAAGAGGGATAAAGAAGAAAGAGACATGATAACATCCCGTAAACAAGA
GTTACAACAATTATTTTTTACTCAGATGGGATTACATGTCGATGAGCCAAAAC
GAAACCGAAGTGGAAATACGAATGACGGAAACACCGCAAAAAGAGCATTTA
AAAGGACAAGGCAACTTTCATCAATACAAGGATTAAAGTATGATATTTTGCAC
AGGTTTTATATTATTTTGGTTGCTATGTCTTGTGAATTTCGTATATATATTTCTAA
ATTAAAACAATGTATATGGATCACTATAAATGGTATCCGATGTCAGCAACTTTT
CGCAAAGTTTTGATTCACGGTCCGCAGATAATAGCATCGTCGCTTTTATCTATC
GGCTGCTGGAGAGAACGCATCTGAAGCACGTAATAAATTCTACAAGCGTGAT
AGACGGTCATATTCGAGACAAATTTCTCGTGTTAACAATTTAACGGACGTATT
TCACCGATCCATGGATTCATCGGATCCTTTACTTTCAAGCCTAAATATAAACAA
AAGATCGTCTCAAAATAAAAAAATCGCTTTGCCGAAAGAAGTCATCAATCTT
TTAGATGCACCAAATGTAGAACATATTAATAATGAACATTTTTTGGGAAGCGA
TGATAGCGACTCGGATTCGGATCACGATGATCCATTTTCTATGCATTTGGACGT
AGAAAATTTTGATTAATATAATAGTATAATTATATGTATATTAAATAGATACATAA
GAAAAATTCAGCTTAAATTTATTATATTAATTAATAAAAAGAAATAATCATGCC
AAATCTACTCCTTAACTTTATATCCTAATATGGGCGGTATGGGTGGGCGTGGTC
CGATTTACTTGAAATTTCACGTACGTATTTTAAATGTCAAATGCACCAATTCTA
CAAAAATGCAGCTCTCTAGCTTTATTATTTGACTTTATGCCAAAAAAAAGGGA
ATCTGGCCCAGTGTG

>TRANSIB2Transib#DNA/TRANSIB2Transib

CACTATGGTCCAGAATTCGTTTTTTTTGGCATAAAGTCTAAATTTTTATGTCAA
TATATCGTCCTTTAGCAATTATTTTCTAATAGAAAATTGATCATATAAATAAAAA
CCAAAAACAAAATTGACCGAAAGCTTCACTAAATCGTTTTATGAGCTTTTAA
AGGTGAAGTGTTAAACAGCTGATTGAGAGAATGCAATTCCCGCCAAATTATT
TGCTTACTTTTGCACGTGGTACGATCGTTAATACGCATCCATAAATTATAGTAA
AATATGGAAAATAAATGCCAAGGTATGTTGTTTTTAACGTATGTAGTTAATATA
TGTGTAGTTGTACTGTTTTTGTGTTGTGTAACATCCAATTTATGTGTATTTTAAC
AGAAAATTCTAACGTGTCCCCTGTGTTTCGTGGAGTATAACTAATTTTCTGAC
AATTTCTGACAAAAGTTAAAAACGTCAGTTTGTACCTTTAACTATTGTCTTTG
ACGGTAAAGTAATATCTTTTGCCCATTTTTGCTCGTTTTCTTAAAATTTGATTTT
ATATAGGCAAGTTCGAGTTCAGACATAGCGACCTCCTGGACATTTGGAGGCG
TAACAATCGGCAAATAAGTTATGTTTCAGATTACGTTTATTCCGCAATCAACA

ACAATAATCTGGAATCGAAAAAGACAGAAGAGATTGACAAAAAAATAGTAC
ATTTTGAATTATTCATTAAAAGAAAACTGCCCGAGTGTAACAGACAACTAGA
CCGATTTATTTCCAAACATACCAAGTGGATGGCGTCTAAGATAACAATTCTTG
TTAGTGATTTTGAAAACTCCAAGACACCACAGCAAATGGGACGCCGAAACTT
ATCTTACCAAAATGCAGGAGAAAGATTAAAAAGGAAACTGGCATCCAATCTG
GCAAGTGAAAGTGATCACGACACAAGTCTTTTGATTCATGCAGCAACTGTTT
CTGCTAGAAAAGAATGCAAGAGGGACGTTGCCTTTGTTCTTAAAGAAACTTT
AAAAACACCAGAATTTCCAAGCGAATCGAGGATGCAAATTCAATGGCAAAA
ACCAACTCCTCTTTCACCAGATGAAGCTCTAGCTTATCTCCTGGAAAACACG
CTGACAAAACAGCAATATATAAGCACCAGGCTTTTAAATAAAAGCCATAACA
GCGACATATATCCGCCCTATAATGAAGTGATCGAAGCAAAATTACAGTGCCGA
CCAGAGGGTATAGAAGTAATGGAAAACACTGCTCAAGTGCTATTACAAAATC
TCTTGGATCATACAGCGCAAAGATTAATTAAGTTGCAATCTGATGTTTTCAAG
CAATTTCCAGATATCTTTAAAATCAAATTAATTTGCAGCTACGGATTTGATGGG
ACAACTGGTCATAGTGCTTACAAGCAGAAATTTGAAACTGAAGCACTTGGCA
CACCAATTTCTGATCAATCTTTATTTGTAACTTCTGTCATACCCATACAAATTAT
AGATTCGTTTAATCGGCATATCTGGATAAACAGAGCACCGCAGTCCATTCGAT
TTTGCAGGCCTCTAAAAATTGAGTTGATAAAGGAAACTGCTGTCCACATAATG
ATGGAAAAAAATCAGTTAGATAATCAAATTAATAATCTTACGCCATTCACTTAT
AAATGTGATGAAACTCGTGACATAGAAGTTACCTATGAAATGCACATGACACT
TATAGATGGAAAAGTCCTAAACGTATTAACAGACACTAAATCTACTCAATGTT
GTCCGATCTGTGGAGTCAGCCCAACACAAATGTTGAAAATTACAAATTTTAG
TTCAGAAACCTTTGCACCAAAAGTAAAAGCTTTACAATTCGGGGTAAGTCCA
CTACATGCTTGGATCCGGTTTTTTGAATTTGTTTTAAACGTATCATACAGAGCC
GAGATAAAAAAGTGGCATATAAAAGGAGAGGACAAGGTTAAAATAAGTATTC
GAAAAAAATATATCCAGGAGGAAATGTGGAGAGAAATGGGCCTTCGAGTAA
GCATGCCGAAGCAGAATGGCAGCGGCAATAGCAATGATGGTAATACGGCAAG
AAGAGCTTTCGCAAGTACCAAATTCTCGTCGATAACCAGCTTCAACGAAAAT
CTTTTGGAAAAGTTTCACATTATTTTAATAGCAATATCGTCCAACTATTTTATAA
ATTCCGACAAATTTCGTTCTTTTTGCGATATAACTTTTCAATTATATATCCGAAC
ATATCAATGGTATGGATATCAAATCAATAATTCGGCGTTGTTTCCACTTGGATG
CCTAGGCGAAAATGCGAGTGAAGCCCGGAATAAACTTTACAAAAGGGACCG
ACTATCACATGCAAGGAAAAATAGTCGGATCAATACGATGAGTGATATTTTCC
ACAGAGCGATTGATTCCTCGGACCCACTATTATCAACAATATGTTTAAAAGAA
AGAGAGAGAAAAAATAGGAAAAAACCACTTCCGAAGGCAGTAATAAGTCTT
TTGGAAATACAGTCTAGTTTTAAACCTGATCCATCAGACAAAATATTGGATTC
ACATTCTGATTCTGATTCGAGTTCAGATTCGGAAATTTATAATGTAGAACTAGA
AGAAGAAGAGGGCGATTATTTTTAAAACAACAATATACACACTTTTATTTAAA
AAAAAAAAAAAACAATGAAATTTCGTTGACTCCTGAGTCATACCAGTAGTTA
ATATGGGAGGCAAGAGAGGGCGTGGTCGCACAGACTTCAAATTTTGTGTGCA
AACTATTTGTGGTTTAACAACAAAATGTACAAAGTTTCAAGTCTGTAGCTCAA
TTTTTAGACTTTATGCCAAAAAAATCGAATTCTGGACCACAGTG

>Transib1Transib#DNA/Transib1Transib

CACACTGGTTTTTTATGTGTTTTTTTTTGGCAGAAAGTCAAAAAAAAATATTT
TAACGGAATAAAAAAATGTATGGTTTCTTTATATTTAGGATAGTCTTATTAGATA
AAAAGTCGATTTTTACTTTTTTACGCAAAACTGGCTTATCGTCGACCTCTCAA
AGACAGCTGATTGTCGATAACATGCGTAAGACAAAAATTGATGGCAAATCAT
TTTGCAACATTTGTGGTTTATATTTCCTCAGAGAATTAAAAAAGTTTAAAGTG
TTGTCATGGAAAACAGTAGCACAGGTATGTAGATTTGTAGTTCACTAACATTT
TTGTGTATGTTATATGTTTTTGTGTAGTTGTATCGTGTTTTGTTTATGTGCCTTT
TTTACAGTCAACTTAACAAATGTAGCTGTGTTTCGTGGAATATAACTTTTGTAT
GGCAAACCACTTCAAAAAAATTTTAACGGCAAGTTATTAACTAACATGTGTAT
AATATTATACAACTAGTTTCATATGCTCCTTTTTGTTCCTTTTTGAAAAAATTAC
GTTTTTGTAGATGAATACTTGTTTGGGCATGCAGAGCTGCTAGACATTTGGAG
CAACAATAGTCGCTCTGAGGATGCAGTTAGGCAGCATATATTTAAAAAAATTG
GTCAGAATAATTTAAATGAATGTTCTATTTCATTGGTAAATAAAAAAGTTAAGT
CCTTCTTTGATTACATAAGGAAACATTTGCCAGGTTGCAATAGATCGTTGGAC
AGGTTGAAGAAAAAACACTTCAAATGGCTGGCATCTAGTATTAGTATAATCGC
TGATTGCAAAACTCCAAAACTAGTTTATGAAAAGGCTAATGACCGACTTAAA
CGAAAATTAGCTTCGGACCTTTTCGGACCTGAAAATAATACTTCTGTACCATT
ACTCTTGCATGCTGCTTCAGTTTCGTCTAAAAAAGGAAATGAAAAGGACATG
GCAGTTGTATTAAAAGCAGCTGGTAACAGCGATAATATCAATAATATAAAAAG
AAAAATGTTCTCTTCTGAGCCCACACAAATGTCCGTCGACAATGCATTAGCCT
TTTTATTTGAAAACGGATTTACAAAAAGCCAATACATCAATTTAAAACGGAAT
ACAAAAGTACACGGCTTTGATATTTACCCATCATATCCTGATGTTCTTAATGCT
AAACTAAAGCTTAGACCAAATGGCATCGAGTATTTTGAGAATAAGGCGCAAG
TTAAGTTACAACAACTCTTAAACCACACAACTTCGCGGATTCTTGAAATGCA
GTATGAAACTTTCAAAGCAAATACCAACTCGATAGAATGCCAATTAATATTTA
GCTATGGATATGATGGATCAACTGGTCAGAGTATTTATAAGCAACGTTATGAG
GAAATCGGTGCTGTATATGATGGATCGCTGTTTGTTACTACAATTGTGCCATTA
AAATTAGTAGACAATGAACAAAAAATAATTTGGATCAACAGATCGCCACAAT
CTATACGGTTTTGTCGTCCTTTAAAAATTGAATTTATTAAGGAAACTCGAGAG
CTTATATTAGCAGAAAAAGAAAACTTGGATACCCAGACTCGTAATCTGCACAT
ATATTTGCATAAAATAAACAACATTAATATATTTGTAAAATATGTCGGTCAAAT
GACACTAATAGACGGTAAAGTCTTAAACATATTAACAGGATGTAACTCTTGCC
AATGTTGCCCGATTTGCGCAGCAAAGCCAACGCATTTAATGAATGTGAATGAT
TTTAATTCAGATATTTTTGATGCTAAATCTCAAACTCTTCAGTTTGGAATAAGC
CCTTTACATGCGTGGATTCGCTTCTTTGAATTTGTTTTAAAACTTGCATACAGA
GCTGATCTTAAAACTTGGCATGTAAAGGACTTTGATAAACCTAAATTAGTGGA
ACGAAAGCATTTTATACAAAAACAAATTATGGAGAAAATGGGTCTCAATGTG
GACAAGCCGTTACCGAATGGAAGCGGAAATTCAAATGATGGCAATACAGCCA
AAAGGGCATTTTCTAATACAGCCTTACTGTCGTCAATATTAAATATTGATTACG
ACATTCTTTATAGCTTTTATATTATTTTAATAACGATTTCCTCAGAATACGAAAT
TGACTCAGAAAAATTTAAAACATTTTGTAAAAATGTTTTTTTGGCCTATCAGA
ACAAATATCCTTGGTATCCTATGTCACCTACTGTTCACAAGGTGTTAGTTCATG
GGCATCAAATTATAGATAATTGTTTGGTGCCTGTTGGCTGTCTTGGTGAGCAT

GCGTCCGAATCGAGGAACAAATTATATAAGAGCGATCGGAGAAGACATGCAA
GAAAATGCAGTCGCCTAGATACTATGACCGATATTTTTAACAGAGCTCTAGAT
ACGTCTGATCCTTTGTTGTCTAGTATTTATCTAAAAGAAAGACAAAGGAAAA
ATAAAAACAAAAATATGCCGCAAGAGGTCATTTCTCTACTTAAATCACCATGC
GTTGGAAGCGATCATGATGATCATGGTGATTGCCAGGACATCGACGAGAGCG
AGGAGGACGATGAAAGAGAACTTCAACAGAAAGATCATGAACTTGATGTAA
TAGCCTCAGACAAAGAGTTTTTTTAAATTAATAAATAATAAGTTGAATTTTTTT
GTAAGTATAATAGCATAACAATGACGTATGATAAATTTATTTTTTAATTTCAGG
ACAAAATGATTTGGACCATAATTTACATCTTAATATGATTTTATTTTATTTTATTT
TCTATATATAATATCAATAAAATATTTAATTAAATACAGATACAAAAATATTTTTT
CTTTGGTAAGGTAAATATTTTTATGGCATATCGGCCAGATCGTTTATATGGCAG
CTATATGAAAGTTGACCAAATCACTTGAAATTTTGTGAATCATCTTGGGGGAA
GTAAAGAGTAACACAAACCAAATTTGGTGAAGATAGGTCATCATTTCGAATTT
CTGCCAAAAAAAAACACATAAAAAAACCACTGTG

>Transib-N1_Transib#DNA/Transib-N1_Transib

CACTATGGGAAATTTGACCACTTTTTCTGGCCAAAATTAATAACTCCAGAACG
GAGAAAGATATTAAGATAATTTTTTTTGCATTGGGTTAYACTAGCACATATCTT
TTATGACATTATGACTTTGACTTAWGTCTTTATCACAAAAATATTTTAGTAAGN
CRACTTAYATTTTTCCGGTATTAAGGGGATAACACGATTTTTTTTTAGTANTTT
GACAGTCACAAACACATAATACAAGCCACACTGAATACAAYTCATTTTGCAG
AATAAAAAAAAACGCTTTGGTTTAAAAGATATTAAAATATTTGTATGGAGGGA
AAAACTTRTCGTTGCAGACCTKTTTTTGTGCGTGCAACAGCTGATTTCTTTGT
TGTGGCGCCATCTATATTTATTTCTGGTATGCAGAACTAAACATTGGTCAATTA
TGAATGCATGCATTTAATATTATCTTAATCGACCGACTTTCATAAAATAGGTTTT
GGCCAAAAAAAGGGGGTAAATTTCCCATAGTG

>TIRANT_IGypsy#LTR/TIRANT_IGypsy

GGCGCAGCCGGTAGGATGTCCTACCTATTAATAATTACCTACCTGTAAGTAAA
CATGTAAGAAACGAAACAAACTATATGCAAGATGTCGACTGAAAGTGACTAG
GAACAAATTTTTATAAAACAAAATTGAAGTTGTGAAGTACCAAATGAAACTC
AAACATATATTCAAACACAGGAAAAAAAAAGAGAGAGGAAAAATGTAAAAT
AAATAAATATACAAAAAAAAAGTGCAAGTGTACCGTACTGCCGCGCTGACGT
GGAATCTATCGCTGATCATCACGCCATCGGTATGTCCATACTCTGCCGAACGT
CATAATTTTTTAAAAAAGTGCAAGTGTACCGTACTGCCGCACTGACGTGGA
ATCTATCGCTGATCATCACGCCATCGGTATGTCCATACTCTGCCGAACGTCATA
ATTTTTATAAAAAAGTGCAAGTGTACCGTACTGCCGCGCTGACGTGGAATCTA
TCGCTGATCATCACGCCATCGGTATGTCCATACTCTGCCAAACGTCATAATTTT
TATAAAAAAGTGCAAGTGTACCGTACTGCCGCGCTGACGTGGAATCTATCGCT
GATCATCACGCCATCGGTATGTCCATACTCTGCCAAACGTCATAATTTTTATAA
AAAAAGTGCAAGTGTACCGTACTGCTGCGCTGACGTGGAATCTATCGCTGAT
CACCACGCCATCGGTATGTCCATACTCTGCCAAACGTCATAAGTTTTTATAAA
AAAAAAAGAGTGCAAGTGTACCGTACTGCCGCGCTGACGTGGAATCTATCGC

TGATCATCACGTCATCGGCACTTACATACGCTGGCCAACGCATCGCCAAAGCC
TCTATATACACTTATATATGTGAGCATACAATATCAACTACAATCCAATACATCC
ACGTACTGTACCGCCTCGTTGGCATGGAATCAAACGCTGATCACCATGCCAC
CGTGGTAAACAAACAAAGCACCAAAGCCTCTCTAATACATTGTACACTCAAA
ACGCACACTGCCATACGTCGGCGAAAAATCAAAACATAAGCAAAAATCATTT
CAAACCAAGCGAGGCTCATTCTGCGTACCACAACGACAACGACACTGCATGT
GTAGTGGCGCACCCATGTCTGGGTAGCCGAGGTAAGGGGAAAACGCTTGAG
TATCGTCAAGTGTTCTTGCCTTTCACTCTTCTACAATGGGTTGCTACGCTCATG
TATTGCACATTCAAAATAACCAAAACAAATGTACTAAAGAAGTCGACATATAC
AGATATATTTTGTTTCCTTTCATTGTGTAATTTTGTATATCAAACAAATACTAAT
ACCAATCACATTGCAGAATATAAAAGGGAAAATATAAAGCCAAAGACAGACA
CCCATACACTCTAGTAAACAAGAAATTTGTTCATTATTTTTCAATCATACATAA
TATACTAAGTAACCTCAAATTTAATGTCAAAAAAGTTCGTTTACAACCTTAGG
AAAACTACACGTTCAGTTGTTGGAGTTCCACCAAACACTAATAGGCCCCCAC
ATCCCGTTAGACGTCCTGACTCCCTTCTCCCGATTTCGGAAGAACCCAAATCA
ATATCTTCCCAAACCCCCAATATGGACTCGGGAAACGATTCTGCCCGCCCCAC
TCCATCCCCTCTGGCGCCCACTGTCAGTGGTATTAGCTCCTTAATTTCAACTAC
GTTCAAGCCTAAAGATATCATGGCATTTGTTGAGCATTTGCCAACCTTTGATG
GTACACCTCGTCTATTGGACAGGTTTATCACTAGCGTAGAAGAAATCCTGATG
CTCATCAGGGGAGCTGACCAAACACCGTATGGCCTGCTTACTCTGAGGACCA
TCAGGAACAAAATCATTGATAGGGCCGACGAAGCCTTGGAACTGGCAAATAC
CCCCTTGGTTTGGGATGAGATTAAAAGCAATCTCATCCGCCTCTACTCGAGCA
AGAAAAGCGAGGCCAACTTGTTAAGCGAGCTTAACACATTTTCGGACAACCT
GACCTTGGGCCAACTGTTCTTTGGTATATCAAAGGTGAGAAGCCAACTCTTC
TCCATACTCAAAAACAGCGAACACAACAACACTGTTGTAGATGCAAAAAAG
GTTGTCTACAACGAGGTTTGTCTCAATGCTTTTATGACTGGTTTGAAGGAACC
TCTCAAGACTTTCGTCAGGATAAAGTCCCCTTCTACACTTGAACAGGCGTAC
GAGCAATGCCAAATAGAGCAGACCTTATATAGGGCACAAAACAAGCGAACC
AACAGACCAGAGCAGGGACCCAATGGATCAGACAATAAAACCTACCGAAAT
AGCTACGACAGCAATTACCGCAGCGGACGTAACGACCGAAATGACCGTAGG
GGACCCTACTCTAACTCTAACTCTAACTCTAACTCTGGCCAAAATAGACCATT
TAATTCACACAATCGCACACCCCAATCCGGCACCAAGGACAACCGGGCCAAT
ACATCAAACCCCTTTCGAGCACCTTCACATAGTTTGAATAATATAGAGGAGAA
CCCTCAACCTGATTCGAATTTTCAGCAAACGGCCTCGGGAAACCAACAGGGT
ACATAAGCCCAGCCACGCACAACCCCTCGCTTCCTTTTATAAAAATCAAACTA
TCCCAGACAAACCCCCTGAAGTTTTTAATTGACACAGGCTCTACACACTCCT
TCATCGACCCAAAATATGTCGACCCTAGGAACTGTGTGACCTTAGATACGCCC
ATAACACTCAAAACAGCCCTGAACAGTTTTAAAATATATCAAAACGTCTCTAT
ACCATTTCCACCGGAATTCCAAATCACGGGCAAAATGACCCTTCTACCTTTCA
AGTTCCACTCTTATTTTGACGGATTGATAGGAATGGACTTATTATCTTACCTAA
AAACAGAAATAGATTTACTTAACCTAAATCTAAAAACCCCAAGTACCATTATA
CCCTTATGGACCCACAGTAACTCAACTTCAAACGTATTTAATATCTCTGGACA
TACGAAAACTATTTTGCCACTACCAGTGGAAACCAAACAGGGCGACTTCTAC

ATCGATTCAATTACAATCAATGATGACTTAATAATATCAGACGGGATTTATAAT
GCCCAAAACAATATTGCTAATTTCGTTATCACAAACTATAGCGAGAGGGATCA
GTTATTGTACCTCGAGAGCCCGATAAAAGGCATGCCATACTCCACGGCCAAC
AATGTTGAACTTTTCAGTATCACTTCAGACACCCCACAGCCCCAAAACTCCG
CAGCGTCGTTACAAGCCCTTGGCGTCGATCACCTCTCCTCTGAAGAGAAACA
AAGCCTACTTTCACTTTGCAAAAGTTATCTAGATATCTTCTACAATGAAGACA
AATCATTGACCTTCACCAACAAGATTACACACACGATTAAAACCACGGACGA
CACCCCCATTCATACAAAATCTTATAGATATCCTTACATTCATAAAGAGGAGGT
CAAAAAACAAATAGAGGCAATGTTAAATCAGGACATTATCAAATCCAGTTATT
CCCCGTGGAGCGCCCCCGTCTGGGTCGTCCCAAAGAAAATCACTCCTACGGG
AGAGCAAAAATGGCGTCTAGTTATCGATTATAGAAAACTCAACGAGAAGACT
ATATCCGATAGATATCCAATACCTAACATCGCGGATATCTTAGACAGATTGGGC
AAAGCCAAATATTTCTCCACACTTGATCTGGCAAGTGGATTCCATCAGATAGA
AATGAATCCCGACGACACACCCAAAACTGCATTTACAGTAGAGGGGGGCCA
CTACGAGTTCATTAGAATGCCGTTTGGCCTCAAAAATGCCCCAGCCACATTCC
AAAGGGTGATGGACAATATTTTTGGAGACCTTATCGGAACTATCTGCCTAGTT
TACCTAGATGATATAATAATTTTCTCAACCTCCTTACAAGAACACTTCATACAC
TTGAAAACTATTTTTGGAAGACTCAGATCTGCCAACTTTAAAGTCCAACTCA
CAAAATCCTACTTCCTCAGGCGGGAGACAGAATTCCTTGGCCACATCGTTTC
ACAAGAAGGTGTTAGGCCAAATCCCAATAAGATCGAAGCTATAAAAAACTTT
CCATGTCCCCACAGTAAAAAGTCAATTAAGTCTTTCCTAGGCTTGTTGGGATA
TTACAGAAAATTTATCAGAGATTTTGCGAGACTTACCCAACCCATGACACAA
AAATTAAGGGGAAACAATAAATCGATCATAATAGATGATGAATTCAAAAAGG
CCTTTGAATATTGCAAAACCTTACTGTCTAACGACCCAATCCTCCAATACCCG
GACTTTACAAAACCTTTCACACTAACCACGGACGCAAGTAATTTCGCAATAG
GAGCTGTCCTATCCCAAGGTCCGGTGCATAGTGATAGGCCCGTATGTTTTGCT
AGTAGAACCTTGTCGGCTGCGGAAACAAATTATTCCACAATTGAGAAGGAAA
TGCTGGCCATTATATGGGCGGTCCAATACTTCAGACCCTACCTCTTTGGCAGG
AGATTCACTATAATCACCGATCACAAACCACTAACTTGGTTAATGAATTTCAA
ACAACCAAATTCTAAAATAGTTAGGTGGAGACTCCAGCTTCAGGAGTACGAT
TTCGAAGTCGTCTACAAGAAAGGCTCTCAAAATGTAATTGCTGATGCTCTCA
GTAGACCAGAGGCCTCTGTCAACCATAACGAAGCCCTATCAATTCCTCAAAA
TGTTTGCCCCATCTCAGAGAAACCCCTTAATGATTTTAATATTCAGCTCCTGTT
CAAAATAACCCCAGATACAAATAACGCCACACTGACCCCGTTTAAACACAAA
CTTAGGAGGGAATTCTGTAAACCCAATTTTCAGTATGACGACGTAGTTTGCAT
TCTTAGGCAGTCGTTAAAACCAAACAAGACATGCGCGGTATTTGCCCCCGAC
CACATTTTTCAAATGGTGGAACAAGCCTACCAAACCTACTTCTCAGCCCACA
GTCAATTTAAACTCATTAGATGTTTGATCTTCCTCCCCGAAATTACTGATAGTA
CGGAGATCGAAAAAATTATAACCGACTATCACTATAATAGTAACCATCGAGGG
ATCGATGAAACATATTTACACATAAAACGACAACAGTTCTTCCCACATATGAA
GGAGAGAATAACTCAGTTAATTCGAAAATGTGAAACATGTTTAAAATTAAAAT
ACGACAGACAACCTCAAAAGATCACTTACCAAATATCCGAACTACCTTCAAA
ACCGTTGGACATCTTACATATAGACATTTATACTATTAACAAAAATTATAACCT

TACTATTATCGATAAATTTTCTAAATTTGCGGCTGCCTACCCTATAACTAATAGG
AATTGCATTAACGTAGTTAAAGCCTTAAAACATTTCATTTCCCAATTTGGTATT
CCCAAAAAGCTGATCTATGATCAGGGAGCAGAATTCGCTAGCGATATGTTCAA
TAAGTTCTGCACTCAATTTAACATTGACCTACACGTTACGTCCTTTCAACAAT
CCTCTAGTAACTCTCCCGTTGAACGGCTTCACTCGACACTAACTGAGATTTAC
AGAATAATACTTGACGTCAGGAAACAACAGAAACTCAGTAGCGAGCATGAC
GAGATAATGTCCGAAACCCTAATCACATATAATAACGCTATTCATTCTGCAACT
AAACATACCCCCTTTGAACTATTTAACGGACGTACTCATATATTCAACCAAAC
AATCCAGTTCAATAACGAACACGACTACTTAACGAAATTAAATGAATTTCGCG
AGAAGTTGTACCCCCTCATCACGGACAAACTTTCAAATGACGTAGTTAGGAG
AACCCTAAAATTAAATGAAACCCGAACAGACCCCGTAGACCTACAACCAGAC
ACTTTAGTCCTTAGGAAGGAAAACAGACGTAATAAGATTACACCCAGGTTTT
CGATTCACAAAGTCAAACACGACAAAGGTCATACATTGATAACTGCTAGGAA
TCAAAAACTACACAAATCAAAAATTCGAAAAACAGTTTTGAAAAAAGACAA
AAGCAACAACGTACCCAACACTGATAATAACTGACCCCACTACCTCTTAACT
TACCATTTCAGGTTCACCCTTGTGCCAACTCAGGCTATCCATGTCCATTATTTA
AATGATAACGCCCCTATAGCCAAGATAGAACTAGGGAAAGCCTTACTAATTGA
GAGGTACAAAATAATTAGTCATGTAATCAACCTACAAGACTACAGCAGATGTA
TGGAACAATTCCATCTGACCATTAATAAATTTAACCCCGATTCCACGTTGACG
GACTCCGTCACAATTTTAAAAACCAAATTAACCCAAGCCCAAGTAAAGCTCA
AAGCCCTTACACCTTCATATAGAAACAAACGGGGTTTGATTAACGGATTGGG
GAGTCTAGTAAAGGTGGTTACCGGCAACATGGATGCCAACGACAATAAAGAA
ATACATGAAGAACTTGACAATATAAAGAAAAATTCCGAAGTCAGTAACGACA
ATCTCCAAAAACAAGTAATGTTTAACAACGAAATACTTATCCGGTTCGAAAAT
ATCACGGACCATATAAATAATGAACAAATTTTGATAAGTAAATTCTTTGATACC
TCACAAAACAAAATATACAAACACTTAAACTTACAAGATACCCTTCTGGAAG
AAATACAATATTTAAATAGGATTAATTATAACATAGAATTATTCATTAACCACCT
AAACGACATAACAGAAAGTATGCTATTGGCGAAAATAAATATAATTCCCAAGT
TCATCCTAAATGAACAAGAAATGGATAAAATAAAAACAATACTGGAAAAACA
AAATATCACAGTCAAAAATGAACAAAGTATATACAATTTCCTACAAATGAATA
CACTAAATTACGAACAAAAGATTATTTTTAATATCAAAGTCCCAATTTTTAAAC
AACCTTTTCATACCCTCGCCAGACTAGTTCCATTACCAATAAATAACACATATT
TTGTAATAACCCCAAATTACCTAGCTTATAATATTAATAATAAGAAATTTCATAT
GACCCGTAAATGCCCCAAACTGGATAATACATTCTTGTGCGACGAGAACTTCT
ACGTTGATACACCACAGAACAACACATGCCTGGAACACCTTTTGAACGGAG
AAAACAGTTCCTGCGATGTACGGGAAACCGGCCCCATCACCGACGTGTTCGA
GGCAGAGAGAGGTTACATCTTCGCATTCAACGTGAACAAACTGAAGGTATCC
CTAACAAACGGCTCCGAGCTCTCAATAATGGGGTCAGCCATCATCAGATACAT
TAACGAAACAATACAGATTAACGGTATCGATTACGACGGCACGGTTGACACG
TTCCCTGAACAGACGGATTTTGATCTTCCCCCCATGCGAAAAGTAACTAGGA
ATACCACTATTACGGTACTAAGCCTAGAAAAACTGCACCTCGAAGCCACCCA
AACAATGGATAAAATCCTGGCCGTCCATCACAATACTATACAGCACACCTGGA
CACTCTACACTCTGCTCGGATTGGTAACGTTCCTAGCAGTCATCTTATGGCTG

CACCGACGAACGAAACACATCGTCCACATCCACGAGGATCATCACGTACCAA
TCTACGCGTCATCCATACCTTCGCTATGGCCGTCACTTCGAACTGGGGGGGA
GG

>TC1_Mariner-Tc1#DNA/TC1_Mariner-Tc1

TACAGCTGCGGTTAAAATAATAGCACTACTGCAGGTGGAAAGTTGATTTCCTA
AAAAAAAATTATTAAATGTTTATATTTTTTAAAGTCAGATTGCATGAATAATAA
GTACCATATGTTGGCTCTCTGAGCAAGAAATTTTTACTCTCTCAATGTAACGG
TTCTTTTTGTTTTTGGGCACTTGCTGCAAAAGTGCGCGAAATGAGGCGGTAA
CAAAAATAGCACTGACCACGTATTTGCTGAATAAAATTAATAGGAGTGATTGC
TTTGGGTTTTTTTTCGACAAATTTTGAAAAAAGGAGTTGTATTAAAGGTTTTA
ATTGAATTTTTTCCAAACGAAGACCAAAAATTCTCTAGTTATGGGTCGCGGAA
AGCATTGTACCGTCGAAAAAAGAAATTTGATTAAAAACATGATCTCTGAAGG
TAAAACCTACGCTGAAATTGGTCGCATTGTCGGTTGTTCAAACAAAATGATCC
GCAAAGCACTGCTGTTTGTCGAAAAGAACGAAACACGGCGAAGAAAGCCCT
CAATGTCCAACGTGGAGATCAAGCGCTTGGTTCGGCAAGGCAAGAAGGAGC
CTTTTAAGCCGGCGACGGAACTGAAGAAGGAGCTTCAGATAGCTGAAAGCG
TGGAAACTGTTCGCAAACGCTTAAGACAAAACAACCTTAATGCGTGCAGCCC
AAGAAAAGTCCCGCTTTTGACTGTTAAGCATGTGGCAAAGCGAATCGAATAT
GCCAAGATACACAAGGACTGGCCTGTGGAGAAGTGGCGCAACATTTTGTGG
TCAGATGAGAGCAAAATTGTGTTGTTTGGTGGGAAAGGCTCTCGGTCTTATG
TTCGGCGTCCACCACGAACTGAATATAATCCTCGCTTCACCTTTAAGGCGGTA
AAGCACGGGGGATCAAACATCATGGTATGGGCGTGCTTTTCATACTATGGAGT
AGGTCCGATCCATTGGATTCAAGGCATCATGGATCAGCACATTTACACAGATA
TCCTGGAAAATGTGATGCTGCCATATGCCGAGGATGAAATGCCGTTGGTTTGG
ACATTTCAACAGGATAACGACCCAAAACACACGAGCAAGAAAGCTCGAAAG
TGGTTTGAGCAGAAATCGATCCGAGTAATGAAATGGCCTGCTCAGTCACCCG
ACTTGAATCCAATCGAAAACCTTTGGGCGGACGTGAAAAAAAAGGTTTCTG
AAGCCAAACCCAATAATAACGAGGATCTTTGGGGTGTGGTCAAGGATTCATG
GAGCAAAATTCCTCAAAAACGGTGCCAGGACTTGGTGGACTCCATGCCAAG
ACGATGCGCAGCTGTCATTGCCAACAAAGGTCACGCAACCAAATATTAAGAT
TCTTTAAACATAGTTCTTAAGATATAATCCATTTGTTGAATTATTTTTTTATTTTT
TTGGTTAGTTTTAGCAAACTACGAGAAACAGTGCTATTTTTGTTACCGCCTAA
ATTTGAAGGTTTTTTTTTTTGTTTTACCAATTATTTTTAAAATATCCATTAGATC
TGTTACTAATTTTTTTATTTCGATTGAAAATCATTGTAGTATTTAAGTTTAGTGA
GTAAAATGATGAAAAAGTGTCAGAAAATAGAGAAACGCTGGGACAAACACG
AAATGTGCTTATGGTGCTATTATTGTTACCGCAACTGTA

>TC1-2_Mariner-Tc1#DNA/TC1-2_Mariner-Tc1

CACTAGTGGGCATAAGTATTTAGACACCATTAGCTTGCAACTGCCGGGCTATG
CTTTTCATAGTACAGTCAAATAAATAAGTAAACTATTGATGATAAAACAAAGA
GTGCAATTACAGTTACACGCGAGAGGTATGTTTTGCCGTTTACCTTTTCAATT
ATCCTTCTGCTGATAAGAAAGCTAGACAGACAACGATTTGGGCGGTGGCAAA

AGTATTTAGACATCACATTTAATTAGTTCGCATTGATCTGTTGTTCGAAGAATA
TCAAATTTTTGACTTTTTTAGATCATTTGAACATTTGTTCTTGATAACGTGTTA
CTACCTACTTTTATTTCATTCATAAATTTGTTAAACATGGGGAAAACAAAGGA
ACTGAGCGAATTTATAAAAAATGAAATAATAATTAAGTATAATTCTGGTATTTC
GGTACAAAATATCATTGATTTATACAAAATTCCACGGGCAACGGTGTATTACC
AAATAAATAAATATAAAAAAACACACACAACCAAAAACGTTGCGCGTAGTGG
CCGACCACGTAAAACAACTCAAAAGGACGATGGTTATATTTTACGGAAGTTT
AAGCAGAATGTTCTACAAACTCCCCGATCGGTTGCCAAAGAACTAAAAGAA
GGAGCAGAAATCGATATCAGTGAAAGAACGGTTCGCAGACGTCTTAAGGAA
GCCGATTTTGGAACATATGTTAGCAGAGTTATACCACTAATAACTCCACGAAA
TAAGTTAAAGCGCCTCGACTTTGCCAAAAAATATGTTGGTCAGCCTGCATCAT
TCTGGAACAATGTTTTGTGGAGCGATGAAAGCTCTTTTGAGTTTCACTGCTC
AAAAAAAATTTTTTTTGTTAGATTACCGAAACAATATCGGAAAAAAGTGGCA
CCAGTATGTCAGAGAATAAATCATTCAGGAGGGTCTGTTATGTTTTGGGGATG
CGTAGCCTTCACTGGCTTGGGAGATTTGGTTCCTGTTGATGGAACCATGAATC
AAAGAAAATATTTAGATGTCCTTAATAATCATGCATTCCCCTCTGGTGATAAAT
TGATTGGAGAGTCCTTCATACTCCAGCAGGATAATGCTCCCTGTCATAAGGCC
AAACTGATTACGCAGTTTCTGAAAGATGTTTGCGTAAACACATTGGATTGGCC
ACCTCAAAGTCCAGACCTCAACATAATAGAAAACCTCTGGTCATATTTAAAA
AGAAAAAGGAGTGCAAACTTATCTAGAAGTCGCGAAGAAACGATTTTGGAA
ATTCAAACCTTATGGAAGGATATTTCAATAGATTATATCCACAGCTTGGTACAG
TCTGTACCAAAACGTCTGCAAAAGGTGATAGACGCTAAGGGAGGGTATATTT
TTTATTAATTTGTCATAATTTTTTAGTTGACTTTTTTTTTTCAATTTATAACATTT
AAATAATTTGTCCAAATACTTATGCCACTGCTAATTTGTAAAAAAGTAATTTTT
GTTAAATCAACAATGAAGTTATCCATATGTTTATGTTACCTAATTTAACTACGAT
AGTCTACCTATTTACTAAAATTATTAAACAATTTTGCTTTAAGTAAACAATGAG
TTACAAACAAATATATTGAATATATTTCTTGTCCAAATACTTATGCCCACTAGTG

>TART_B1Jockey#LINE/TART_B1Jockey

GATCCGGCCGGATGGCAGCTCGACGACGGTAGCAGGCCACCATGCCCGCCA
GCCCGCAGCTAGGATAGCAGCAACATCAGCGTCTTATTAACGCGAACGGCGG
CACTGGCCCTTAGCTGACCAGCTGCAACAACATCAGCGACGCAGTCTCCACC
AGCAGCAACAACAGCAGCGGCGCTTGCGACAGCAGCAGCAGGGAATGGTTC
TGCCGCAGTCGCCTATTGCTGCCTATAAAGAGCATTCCTTGCCAATTGGCTTT
CTGTACGACGACGATGATCCTGCGGACAAATCCACCTTTCCGGACGCCTGAG
TATTATGGCGAGCCTGCTGATCATTGTTTCTCCGACTCCTTACTTGACCACTGG
ACAATCATACAATCACCGGCTTTTCACATCTGGAAGAAAGAAACCTGGATTA
GTATTTGCCCTTCAGATTTGTATGAAACTGCATTTCTGGCCAACCGGAGTGTT
TCCTGCAGACGACGCTGGACTGCGACGCACTTTTTTCTACCAACACAGATGG
GACGCTTGCATCAGATCTGACGGGCCAGACTGGCCGATGCCTGGTACACGAT
GGCATCTCCGCGGCGGCGAGATGTGCAGCGACGCTGAAAGCAATAATAATTC
TTTTACTTTGCAGTATCAAAAATGAGAAGCCACATCACAATTTACATCAGCAA
AAATTACACGTGATATCTTTTTCTTTTGCCTAGGAGTAGTGAGACCTGCTACC

AAAATGACCAGTAATACCGGCTAACGGAAGCGAGATATCGATAATCATTCTGG
CGGTAAAATAGCCAGAACATCGATAACCAGCTGTGCATTTTCCCTTCGATTTA
TCGAAGTCAAATAGCCCGCCTAGGCTATCGGACCGGATCGGCAAGTCAGTAG
TGATTAAAAATCCGGCAGAGGCGCCTGTGCAGCAGCTACCCAAAGTGAACA
CGACGAAACGCAACAACAAAATGGACGGACATAATGGAGATCAAAGTGAAG
GATGGGCAACAGTGCTATCTATCTCCTCGGATGATAGTAACTCCTTGTCGTCG
CCGCCGTCTATAATAGTTTCATCGCTGGACACCACGCCAACTTCGCACGAAAC
CACCATAGTAAGAAGAAGCCTATATCAAACAAATGCTGACATGAAATCTTACG
ATTTTGAAAATATTGTATTAAATGAGAATAAAAATACCATATTGCCAGACCCTT
TATTTGTTGATAAATGCGGGAGTACTGCTAATACCACAGAAGCCAATGAAAAA
AAGCCTGCCAATAGCCCCTTCCCCATTTCCATTAGCAAGAATTTTAGCACTTC
CTCGCCACTAACACATGTAGACACGCCTACACAAGAAGATGACGCCAGTGCG
TTCAATACATTGAAAGCAGCTAAAACAGCTAGAATAATTTTTCCCACACACAC
ACATATAAAGCCCACCAAACCCTCGCCCCCCTCCAAAGAATTAAGCACAAAC
TCAGCACTAAAAACTCTCAGCTACACAGACAAAATAACAGGAACACAGAAA
AATCTACCCGACAAAACACATGTAGACACACCTACACAAGATGACGACATTA
ATGCAACCAAAGCATCAAAAACCGCTAAAATAATTTCTACACAGACACACCT
AGGTGAAACCAAGCCAATACAGCCCGCCAAAGACCCCTCACCCCGCACCCA
AAAGCCTATTGCACACAAAGCTGACGAGACACTTACCCACACAGACAAACT
CACAGCATCACAAAACCTCGTTCCCGCCAAAACACATATAAACACACCCACA
CAATATAACGACACTAATGCAACCAATGCATTAAAAACCGCTAAAATAAATTT
CTCTTCACACTCACACCAAAGTGAAATCAAGCCAACACAGTCCGCCAAAAA
CATTTCACCCCTCACACAAAAGCAATTCACAAGCGAATCAGCCGGGACACAC
ACCCATACAGACAAACACAAAAACACAGCATCACAAAATCTCTTTTCCGCCA
AAACACACATTAACTCACCCACACAACATAACTACACTAGTGCAGCCACAGC
ATCAAAAACCGCTAAATTAATTCTCTCCCCACACTCACACCTAAGTGAAACC
AAGCCAACACAGCCCGCTTTAAGCCCCTCGCCCCTCTCCCAGAAGCAAATAA
CAAGCATAGCAGCCAAGACACTCACCCACACAAACAAACACACAGCATCAC
AAAACTTTATTCCCGCCAAAACACATATAAACATACCCACACAATATAACGAC
ACCAATGCAACCAAAGCATTAAAAACCGCAAAAGCAGCTTCTCCCTCCCACA
CATACTCACGTCAGACAAAACCAATAAAGTCCGCCATAAACGCATTGCATCC
CGCCCAAGACACAAACCCAAGCCCAGCAATCAGTGCTGTCACTTACACAGA
CAAACCCACAGCTACTCAGAATATTTTTCCTGTCAAAACTTTTGCAGAGCTG
GTTAGGGAAAATGCAAAACGCTTACCAACTCCAATGCAGAATTCCCATCAAG
CTAAAAATGACTCTGCCGCTCTCGGACGCCCCCCGACTGCAGCTAGAAAAAA
TCTAAATAAAACACTGATTTCTCCTAAAACTCCTGGGAAGCGCCGTGGGGAC
TGTCTTGATGAAGGCCTACTACAAACCTCTAACAAAAAGGTTAGAATACGCG
ATGACTTCTCTGACGATGATCTTGGGGTCACAAATCTACTCTCTGAAACACCC
TTATTCAAAAGCAAAGCAGCTATCAAGATCCGGCAAGACTCGAGAAGAGATT
CCCTGCAGAAGTCAGCCGAAATGGACACAGCTCCAGCAATTAGTCCCTCAAA
CACAGCAGCCGATTCCGACCTACCGCCCTGGAAAACTGTTCCAGCTAGCAGA
AAACCACCATCAATCTTCTTGTCCAATATACAGCAGATTATCCCGCTAATAGA
AAAACTAAACTATAAAGCCGGGGTAAATAGCTTTACCACCAAATCTGAACTT

GGCAACAATATTAGAATCCAGGCTAAAACGATGGACGCCCACAATGCAATTC
AGAATGTTCTCCTTGAGGCAAATATTCCCCTACACTCCCACCAGCCAAAGAG
TGCAAAGGGCTTCCAAATTGTAATTAGGCACCTCCACCAGTCAACCCCGACC
AAATGGATTGAAAGCCAACTTCAAGACATCGGTATAGCTACAAAATTTATCAG
GGCAATGCAGTTTAGGGACACGAGAAATCCTATGCGCATTCATGAGGTTGAA
GTTGTACCCAAGGCTGACGGCAGCCATCTCAAGGTCCTGCTACTAAAATCCC
TTGGAGGTCAAACGGTCAAGGTTGAAAGGAAACGGGTATCGAAGGATCCCA
CACAATGTCATCGCTGCCAATGTTTTGGGCACACAAAAAATTATTGTAGAAAC
CCGTTTAAATGTATGAAATGTGGCCAGCTGCACGCCACGGTTTCATGCACTAA
ACCCAAAAACCTTCCGGCTACTTGTGCAAACTGCAATGGAAGCCACGTAAGC
AGCTATAAAGGATGTCCCGCTTTCCAAGAAGCAAAGCAAAGACTATCTATCA
ACAAAATTCAATCCCTACACTCACAACCCACGCACCTTCAGACCCCCCGCAA
TAAACATCCCTACCCAAAACCCACCCACCTTCAGACGCCCCGCAATAAGCAG
CCCTACACACACCCCCTCCCTCGCACATCAGTAAACAACACAAAACTACCTG
CCAAAAGAATCCAAGGAAAGAAGATATCGCAAAGGAATCTATCTATAAACAA
ACGCTTACACAGAATGAAAAAACCGAGGAAGGAGACAAGCCCGCCGACAA
CTAGCAAAAAGGTCTTGGCCTCTCTAGAAGAAAGCAGAAAAAACCCAAATA
GCGTCCTAAACCCGGCCAACACCCATCTCACTCATTTCCGCCCACCCCCTTTA
GCACAAAATATTCCTAATGACGAACCTAAGGAGCTGAGTGGGGAGCAATACC
TTCTAAATCGCATTGAAGGGATGGAAAAGAAGATCAACAACCTTCTTGAAAT
CGTCACCCGCCTACTAAGACAAGGAAAAGACTGTCCAAAGTCTCCAAAAAA
TCCTTTCCGAGATCCAATCTTCGTGTAAATGCTCTTTCTAGTAACATCAGAAA
GTGACGTTTCCTATGACTCGGGCTGACAACAGGGACATCCTTAAAATCGCTTT
CTGGAATGCTGGTGGGATTAACAATAAAATAGATGAGCTTAAGCTGTTCATTC
TAAATATTGATGCCCACATAATCATAGTCACCGAAACTAGACTAGACAACAAT
TCTACCAAACTAGAGCTGCCAGGATATTTCACATACTTAGCCCAAAATCCTGT
CTCTAGCAAGAGAGGAGGGGTCGCTACTATAGTAAACAGCAGTATCCGACAC
ATGGCCCTAAAACCGATCGAAAAAGAATGCATACAAAGCGCCCCAATAGTCC
TGCTGCCTGAAAACAACAGACGCAGCGAAATGATAGTAATAGCATCTGTCTA
CTGTCCGCCCTCGCTAAGGTGGTTGCCCCACCACTTTACTGACGTTCTCAATT
TTGCAGAGAAAACTTTAGGAGGGCAGACCAAGTTTATCCTATGTGGCGACTG
GAATGCAAAACATAGACAATGGGGTTGTACACGCGCCTGCCAACGTGGCACC
GCACTCTACGAAGCAGTCCAAGCCGACTCCACTGCAGAGATCATTGCCACTG
GCTGTGCTACACATTTCCCACATGACACAAGAAAAAACCCGTCAGCAATAGA
TTTCTCCATTTGCAAAGGGCTTGGCAGGTTTGAAAAAAGAATTTCTTCAGGG
GCGGATCTATCCTCAGACCATCTTCCCATCTTGTTAGAGATAAACCTAGACAC
AAACACCCTCTTCTTGCAAAAACAAAACAACAATATACTCAAGAAAAATACA
AACATCGAGCTGTTTAAGAAAGTTCTAGAAAGAAAGATACTATTAAACACTG
AGATAAGGGTAGCAGAAGATATAAATGACGCCATAAGCACCTTTATGAAAAA
CATCAAGGACTCGGCTGCGGAATCAACTCCCTCCCCAAGAATACGTGACAAC
CCCAGAAGAAGACATAGGCAAGCCAACAGAAACAGTCATACCCTCGCACTA
GACGAAAACACAAGCAGACTGCTTGAGGAAAAACGCATATTAAGTAGAGTT
TTCAAAGCTACCAAAAATTACGAGGACAAAGCTAAACTAAAGGCAGCTGAA

AACCGACTAAAAAAAGCGATAAAAATATTGAGAGAAAACAGAATCAATGAG
CAAGTTGAAGGAATAGATACAAGTAACCCGGACAGAATGAGAAAAATGTGG
AAACTGCTAGATGAAGGGAAAAGGACAAATCAGCCTAATTTTCCCCTCAAAT
TAGAAACCCAAAAAGGACCCAAATGGACAAAAACGATTAAGGAGACAACA
GAAACATTTGTCTCCCACTTGGAAGGAAGATTTAAGCCAAACAATAATGTAC
CTGATTACCACATAGATAGAGTCAATACTGGACTAAGAATAATTAAGGAAAGC
ATGCTAACTGAACGACATAATCTGAACAAAAACCCCCATAACCAACCTATTAC
TCTAAAGGAATTAAATGACGAAATAAAAAACTTAAAGAATAGTAAAGCACCG
GGAAAAGACCTTATAACAAACCAGCTAATAAAAACCCTACCGACTAAAGCTA
CCCTTTACCTTATCCTAATCTATAACTCCATACTTAGACTAGGATACTACCCAG
AAGCCTGGAAACATGCGCAGGTAAAAATGATTCTCAAGCCAGGGAAAAGTG
CAAACGAGCCAAGGTCATATAGGCCGATTAGCCTACTCTCGGGACTCTCAAA
AATCTTTGAAAGACTACTCTTAAAAAGACTTTTTAAGGTAGATTTATTCAAAA
AAGCTATTCCTCTGCACCAATTTGGCTTTAGAAAAGAACACGGATCGGAGCA
GCAAATAGCCAGGGTCACCCAGTTCATTCTTGAGGCCTTCGAGAGAAAGGA
ATACTGCTCCGCGGTCTTCCTTGACATCTCTGAGGCTTTCGACAGGGTATGGC
ACGAAGGCCTTTTACTTAAATTAGCTAAGATCCTACCTTACAACTTGTACATTA
TACTGGAGAGCTACCTAACAAATAGAACGTTCGAAGTTAAAGACCAAGCTGG
AGAGACTTCGAGAACAGGACAAATTGGTGCTGGTGTGCCTCAAGGAAGCAA
TCTTGGACCACTACTCTACTCTATCTTCTCCTCAGACATGCCCCTTCCATATAT
CTACCGCCCCTCACCAACAGAAAGAATCATGCTCTCAACATACGCAGACGAC
ACTATAGTCCTCAGCTCAGACACACTAGCAACTGCCGCTACAAGAAACAACG
AAAACTACCTCAAGTCATTCTCGGACTGGGCGGACAAATGGGGTATATCAGT
AAACGCTGCTAAAACCGGACATGTCATTTTTACATTAAAAAACGACTTACCTA
CAAGTCTAAGGACTATGAAGATCAAGGGTCAAGTAATAAAGATTGAAAGCAA
ACAATCATACCTTGGCGTAATCCTTGACAGCAAGCTAACCCTCAGCTCCCACG
TCACAAAGCTGATGGGCAAATACACTACAGCCTACAGAAAAATGACATGGAT
TCTAAACAGAAGAAGCAAACTCCCTACCAAGACTAAAATGCTGATCCTTAAG
TCAGTCCTTTCACCAATATGGCAGTATGCCATAGCAGCTTGGGGTCCCCTTGT
GACGGATGCACAGATAAGACGGATCCAAGTCGAGGAAAATAGGAAAATGAG
AGACATATGCAGAGCGGGTAGATACACGAGCAACCAAACTATAAGGGACCGC
TATGGAATCAAAACAGTAGAAGAATTCTATCAACAGGCACTCCACAGGTTCT
CAGAAACAATAAAATCTCACCCAAATATAGCTGTACGCAGGATATTCACAAG
GCACTATATCCCGAACAGACTAGAAAGAAGCAGGCAGAGGTACTTGAAAAT
GACAAATGAACACATCACGCAAAAACAGACTGGACAAACACTCTCACCTAA
ACTCCTAAAAATCCCTGATCTAAATGACTGCAGAACCCTTAAAAAGCGGAAT
GAAAGAGATAAAATAAGGCAAACCCATCTAATTGAACTCCCCACCCTGCTGA
GACTAGAGGAAGAAGAGGAAGAGCTTAGAAGAATAAAAAAACAGGAAGAG
AGGGAAAGAAGGGAAAAGGAAACCCAAAAGTGGCCTCCAGACAGATGGTG
TGAATTAGAAATTAACCTATATAATAAGCAATATAGAAGGGGCGATCTAACCA
GGCAGGAAATCATACAAAAATTTAGAGGACAACCACTAAATGTACAGAGGAT
AATTTTACCCGACTATAAAGGAGACCAGGAACACAATTAAAAGTAAATCATG
GGAAAGCAGGGGAAAAAAAACAGAAGTAACAAAATGGCGGAAGGGGTGGC

AAAATATATATAAAAATAAGAAAAGATAGAAATAAAAAGGCTAAAGGCTCAC
TTACAGGTTAATTCAAAAGGGAAACGTGCTTACATATCTAATGGTAAAAACTA
ACTTATAACACCTACTGGATGACATAGTGAGGATGCCACAGGTTGCGCACTTG
AATCACGCTTTCCTGGCGAGATTGTTAAGGCATCACCGGGATCCCGTCTCTCT
TTCCTAACTGGAGGACTCATGCCTGAAATCTTTTCCGCTTCACCGGCACCTGA
TGGCCATATCTGCTACTGCGCCTTGCGTCCACGGACGCTCCTTCCTGAAAGA
AAGAGAATTTGTTTTAGTTTTCCTTTCTCTTCTTAAAAATATAACAAAAACAG
CAGCAGTAGCCGACCAACAACATACTTAGCTTACCAAGGCAGCAGCAATGAT
ACCAACAGAAGCCCTACAGTGTGCTGAACAATATGCCGCCCACGCCCACCAC
TTCTGGAACTCAGGAATGGGGGGTACGGAATGCCGGAAACTGGAGGATTCG
CCTCAAATGTTACGCTTACATCTTTGAGCAAATCGCCTGCCTCCTCGTCTAAC
GGCCCGGCGTCTTGGTACGCCCAGAAGTAGCAGCGGTAGCAGGATTAGCAG
CCTCCACAGAGATATGTTCATCCCAGCATCAGTCCAGTTGGCATTGTGATGCC
GCCGTAGCGTACGCCCTCAGAGACCCGACTGGAATCCCAGGACAAGAGACG
GCAGCACGCCTGGGCTTAGGCGCGGGACAACATGCAGAGATTCGTCAATGA
GAATGGCGGCACTGGAATCACCTTTCAGCTGAGCGGGATAATGAGTCGGCAA
CGCACCGCACTATCAGCAGCAATAACAACATCGGCAGATGACCTGGAAGAA
AGGAAAAAACATGTATCAGTCTTTTCTTTTCTTTTTTTTTCCCATTAGCATGCG
TTTTTAAATTCCATTCCTGAAAACCGGCGTTTTCCTGTAAAAGACACTGGACG
GCGGCGTTTGTTTTAAATGCAAAGATTGGATGCTTGCATCAGGGCTGACGTCC
TTGAATAGCCGATGCCTTGTAATTGCCTGCATAGCCGCGGAGGTCCTGATGAC
GGATGCTGGCCTGTATTTGGCCACCACTCCGATGCCATGACAGCAGCAACAG
CAGCGGTTTTTGCAAATGAGAGGGATCAGGAGGCGGCAACACATTCACCCG
TAGCAGCAACAACAAGCAGTGGTAGCATCAGCAGGGAGTCTTCTCGCCACA
ATAGCCATTTGGAAATTTCTCGACGACCCGACTTCCTGCTTGGCCCATGGTCA
ATCACACTGGACCAGTTAACAGCGGCGTTTTACCCTGGAAGAAAGAAAACTT
GTATTATTCATTCCCTTTTCTCTCACAGTTGGGTATTTAAGTGCATTCCTGGCC
AACCTAGCGTGTCTCCTGGCAATGACGTCGGACTCAACGCATGCTTCCCTTC
CGACACAGTAAGGATGTTCGCATCTGGGCTGACGGGCCAGGCCGGTCCGTTG
CCATACATGCTAGCGTTTCCGCGGCGGCGTAGTGAACATCAAACCGTATAATG
GCGAAGACGACGATGATCCGGCCGGATGGCAGCTCGACGACGGTAGCAGGC
CACCATGCCCGCCAGCCCGCAGCTAGGATAGCAGCAACATCAGCGTCTTATT
AACGCGAACGGCGGCACTGGCCCTTAGCTGACCAGCTGCAACAACATCAGC
GACGCAGTCTCCACCAGCAGCAACAACAGCAGCGGCGCTTGCGACAGCAGC
AGCAGGGAATGGTTCTGCCGCAGTCGCCTATTGCTGCCTATAAAGAGCATTCC
TTGCCAATTGGCTTTCTGTACGACGACGATGATCCTGCGGACAAATCCACCTT
TCCGGACGCCTGAGTATTATGGCGAGCCTGCTGATCATTGTTTCTCCGACTCC
TTACTTGACCACTGGACAATCATACAATCACCGGCTTTTCACATCTGGAAGAA
AGAAACCTGGATTAGTATTTGCCCTTCAGATTTGTATGAAACTGCATTTCTGG
CCAACCGGAGTGTTTCCTGCAGACGACGCTGGACTGCGACGCACTTTTTTCT
ACCAACACAGATGGGACGCTTGCATCAGATCTGACGGGCCAGACTGGCCGAT
GCCTGGTACACGATGGCATCTCCGCGGCGGCGAGATGTGCAGCGACGCTGAA
AGCAATAATAATTCTTTTACTTTGCAGTATCAAAAATGAGAAGCCACATCACA

ATTTACATCAGCAAAAATTACACGTGATATCTTTTTCTTTTGCCTAGGAGTAGT
GAGACCTGCTACCAAAATGACCAGTAATACCGGCTAACGGAAGCGAGATATC
GATAATCATTCTGGCGGTAAAATAGCCAGAACATCGATAACCAGCTGTGCATT
TTCCCTTCGATTTATCGAAGTCAAATAGCCCGCCTAGGCTATCGGACCGGATC
GGCAAGTCAGTAGTGATTAAAAATCCGGCAGAGGCGCCTGTGCAGCAGCTAC
CCAAAGTGAACACGACGAAACGCAACAACAAAATGGACGGACATAATGGAG
ATCAAAGTGAAGGATGGGCAACAGTGCTATCTATCTCCTCGGATGATAGTAAC
TCCTTGTCGTCGCTGCATATATAAAATAATAAATTTCTTAAAATAATTTCATCGC
TGGATTCCTGCGCCACCATAAATTACTTATAGAATACACTTAACACTGTACCCA
CTAATGCTAAAAATTTTCTTATACAATTTTACTTTAAAGCTAAAACTACTTAAA
CTATGGCGCGTAAAACGGCCGTCAAAAATATCCAAAAATTGTACTTGCCATAA
ACAAATTCTAACATAATGTAAATCCTAAATCGTAAAATCATGGCGAACATAAC
CGCCGCCAAGCAAATCAAAAAATGTTCCTAGAAATTGTAAATCTTAAACCTA
CTTCTCTAACTAAAGCGCGCACAACTGCCGCTGATCCGAAATTGTAAAATGC
CATAAATTGTAACTATATATTTTATAAAAAAAATCAGAGTACAATAAAATGACA
GCGCCGAAAGGCACTGAAAATATTTAAAAAAAAAAAAAAACTGCCGCTGATC
CGAAATTGTAAAATGCCATAAATTGTAACTATATATTTTATAAAAAAAATCAGA
GTACAATAAAATGACAGCGCCGAAAGGCACTGAAAATATTTAAAAAAAAAA
AAAAAAAA

>TART-AJockey#LINE/TART-AJockey

AGTAGCAGCATTTAATGTCTTGCAGCAGTTTCCATTTGACAACTTCCGACGAC
CAGACTCCTCGCTTGGCCCCTGGCGATTCGCCCTGGAAGAAAGAAAGAAGC
ATGGATTAGTCAAATATCAAAGCACAACAAGCATGCACGAAATCTTCGCAAC
AAGGACTATTCCAAGGCAAGCGATGGTGTGTTCTTTGGCTCTACATATATCGT
TGAAATCCGTCTCCAACTGCCGCGAGTCACCCACGACTTCCACTCCCTGCAG
ACGGACGTAATGCACTACGATGTGTAAATTTTCCGGATCCAAGTGAACGCCA
GGCGGAAGAGGATTCCCTGGATGTGAAGGATCACAAACTGCTGCTTTTTCAA
GAGTCCCCATTTAACAACTTCCGACGACCAGACTCCTCGCTTGGCCCCTGGC
GATTCGCCCTGAAAGAAAGAAAGAAGCATGGATTAGTCAAATACCAAAGCA
CAACAAGCATGCACGAAATCTGCGCAACAAGGACTATTCCAAGGCAAGCGA
TGACGGACGGAATGCACTACTATGTGTAAATTTTCCGGATCCAAGTGAACGC
CAGGCGGAAGAGGATTCCCTGGATGTGAAGGATCACAAACTGCTGCTTCTTC
AAGAGGTAACCGGCCTCATTTCCACTTGGGTCACATCGATTGTGGAGGAAGC
CGATTGGGACTTTGAGCGAGCCCTAAAATTGTTCATTCAGAAGAACGCTGAT
CACGAAATCCCAGATCTAGCATTCGCCTAGAATATGTGATCTCGTTGGACTGC
GTGCTGGACTGCGGCGCATGCTTTCCCTCCCGCCAGAGACGGGATGCGCGCA
TCAGGATCGACGGGACAGACCGGCCGACGCCTGGCTTCACGTCGGCATCTAT
GCGACGACGTGATAATCAGCTTCACAGTACCTGCGGATGAGGGCACAGGCGC
CAGTCCTCTGCCTAGCGACACATACACCAACAGCAACATCTAAAACAGTCGC
AGTAGATGCAGGGAATTTTCTTGCTGCAGTCTCCAACTGGAAAAATCCGACG
GCCCAACTCCTAGCTTAGCCCGGCCAATCTTCTTGGACTGGCAAACGATGTG
ATGAATCGTCCTGGAAGAAAGAAGCATGGATTATTTCTTCATACACAGCTTGT

CTACTAAAATGCATTTCCCGGCAAACTGGCACTGGGGCGTTGAAAATACGAC
ACTACTCACCAAACAGCAGCAAAAACAACAGCATCGGCAGCAGGCGACCAG
CAGCTGCTCTGGCTCACCCAATGCGACAGCAATATCTGCGGCATGGCCAGCA
ACATGCAGCAAGCAAAAGACCGGCATGGTGGTGAACAACATATCGCTCACG
CCCAACGATCTGGAAGCCCTGAGCTAGGGAACTGAGTATCCGCCAGCGGATA
CTTATGCAGCGGCGAGGCGTCGGAAATCCGATAGCCGGAAGAGAGGCCGGA
GCAGCATGTTGGGCATTTTCGCCCCAGTAGCAGCTGCTGGGGCAGCAAGGGC
AGAGGTAGCAGTGTCAGCGCTCCTGGTAAACCGGCCTTTTCTGTTGAAGACG
CGGGACTGCGGCGCAATTGTTCCTGCCAACCGGGAGTTCTCTGCGCCTGAGG
TCCTGTTGTGCTTCAGCGGCTCCATCTGCTGTTGCATCTGGCGCTTCTCCAAC
TTTCCTCCCTGAAAGAAGAGAATATTAGTAGGAGCATGAGACCAACAACAGC
CCTAGCTTACCACGTCAGCGGAAACAGCAACTGCAGAAGCCCGGTAACTTTC
TGATAATATGCAGCAATAAGAGCGGCTGGATCAGCATGTTGTGTAGCTTGGCC
CCAGAAGCAGCAGCTGCGGTACCAAAGGCAGCGGAAGCAGGGTCAACATAG
TCTGCTATGCTGTGTCCATACTAGCATAGTCAAGTCAGCGGTGGTGACGTTGC
CGCAGCAGCTGATCCACAGGGTACGGCCTGGAATGTTTTGTCAGAGACGGCA
GTGGCCTGGGCGTATTAGTCAGATACGCCAACAACATGATACCCATGCGCAA
CCATTCTTCCGGTGGCGGCATGCTCGTAAAGTAGCGGCGGCGCATGCATCTCT
CGAAGCAGCAGCAGCAACAGAGGCGTCTCATAAGTGCATGCGCAGCAGCGG
TAGCAGCGGTGGTGGCAGCAGCAGGGAGATTTCATTGCTGCAGTCGCCAAAT
TACATGCATCCTACGACTCGACTCACGCTTTTTGGCCCCTGGCCGATCTTCAT
ATATCTCAAATCGACGGCTGCTTTTACCCTGGAAAAAAAAAAGGAGCTTGGG
TTATATTGAACTACACTAGGCGGCGACACACTCACCAGCAGCAGCCAGAACA
ACAACAGCGGCAGAAGTAGCAGGAAATTATCTCACTGCAAAATCCAATTGGC
ACTTTCCGAAGTCCGTATTTCTCGCCTGGCCCCTGGCCAGTAACAGGGGCCT
GTGCGACGGAGATGACGACGATGATCTGCCCTGTGGACGGCCTCAAGAAGG
AATCGGCCCTGTACTGCCCACCTGACCTGTGCCAGGATAGCAGCAATGTCAG
CGGACCATGGATGGAAGAGGCGGCACTGGCGACGGTCCTCAGTTTTGCGGT
TTCCGCGGGGCGTAGGCGCAGTAATCGGCGGCAGCAACAGTGGCGGCAGCA
GAAGGGAATGTGTATCTGCAGTCGCCGAATGGCAATTTCCTGCCCAGCTGTA
ATGGACCGACAAATGTTTTTTGGCATGTCGACCTGTAAGAAATAAGCAAGGA
TAAGTTTTTTCTTTACAAGATTGTATATGAATTTGTATTTACGGACAACTGGCT
TGTTTCCTGTCGACGATAAATGGCGCTTCATGTTTTCACTCTGCCAAAATCTG
AGTGCAAGCATCGGGGCTGACGGGCCGAATTTGCTGATGCCTTGTACATGCT
AGCATCTTTATGCCGCCGTGTAATTTTGCGACAGCAAAACACATGATGGAGA
AGACGAAGGTGATCGATGGCGCCCGGATGACGGAAGCAGGCCCGTGCTACC
CACTGGTCCGTTGCCAGGACAGCAGCGTTTCAATTATGCGAACAGAGGCACT
GGAGCCACTTCTCAGGCGATAGGGATCATGAGGCGACGACAACTCGCCAGC
AGCAGTAACAACAACAGCGGCAGCAGCGATCTCGGACTTGCTGAACATTGC
GGCTTTTTCCCAGCCTGGACCGTCATCACAAACGCTCTCAAAGATCGGTGCG
CCTGCAGCCTACGGAAGCGTACAACTGCCCAAAGTCGTGCTATTGCATAATG
TTAGGTGTCCTGGCTGCTCCGGCGGATGAGCTCCACCCGATTGAGAGAGGTA
AGAAGGCCCAGCAAGTCGCCCTTAAATAAAAGAAACAAGCATTAGACTCGG

CTGAAATTGTAACATCAGTCAGACTGTACTCACGCATGCACTTAGACAAACC
GAAGCCTATTCCACAAAAGGCTGTGAGTGCACCAAAGTTGACGGGCCACGC
CGGCCGATGCTTGCTGCATACCGGCATCTCCGCATCAGCGACGTTACAAGCA
CCACTAAAATTAGAAAGAGGCTTACTATAGGCAAAGCGCAGAAAAACAAATA
TTACAATAAGAAATAGCACGCGATTTTTCTGCTTCGTTTCCGCTTGGTACAAG
TGAGACCATTCTCCCTATATGCCATTCTAGTATATTTGCCCGATAAGCGATATGC
TGGCGGCAAAAAAAAAAAAAAAAGATAATAGCCGATAAGCCCGCCAAAAAAA
AGCGCGAAATATCGATAACGAGCTGGCACGAAAATTGCTGGCATATCGATCA
ACCGCTGTGTCTTTGAGCTATCGAAAAAGCCCGCCTACGCGCCTAGCCCAAA
TTTGCAAGTCAGTTGAGATTATAAAACCGACAGAGGCGCGCGCGCAACAGTA
GCTAAAAGTGAAGCCAGCAGCAACAACAACAACTAATGGACGGCCAGAACG
TGAACCAAAGCGGAGGATGGGCATCGGTTTTATCCATCTCATCGGACGATGG
TAACTGCTCATCCTCGCCGCCATCAGCTATAGTCTCATCGCTGGATACCACGC
CAACGTCAAATGAAACCACCATAGTAAGAAGAAGCCTATATCAAACAAATGC
CGACATGAAATCTTACGATTTTGAAAATATTGTATTAAATGAAAATAAAAATAC
CATATTGCCAGACCCTTTATTTGTTGATAAATGCGGGAGTACTGCTAATACCAC
AGAAGCCAATGAAAAACAGCCTGCCGATAGCCCCTTCCCCATTTCCATAAGC
AAAAATTTTAGCACTTCCTCGCCACTAACACATGTAGACACGCCTACACAAG
AAGATGACGCCAGTGCATTTAATACATTGAAAGCAGCCAAAACAGCTAGAAT
AATTTTTCCCACACACACACAGATAGAGCCCGCCAAACCCTCGCCCCCCTCC
AAAGAATTAAGCTCAAACTCAGCACCCAAAACTCTTAGCTACACAGACAAA
ATTACAGCAACACAGAAAAATTTCCCCACTAAAACACATGTAGACACACCTA
CACAAGACGATGACACTAATGCAACCAAAGCATCAAAAACCGCCCAAATAG
ATTCATCACACTCACAACTACATGAAACCAAGCCAACACAGCCCGCCAAAAA
CCCTTCACCCCTCACCCAAAAGCTAACCACAAACAAAACAGCCAAGACACA
CACACACACAGACAAACCGACAGCGTCACAAAATCTTTTTCCCACCAAAAC
ACATATAAACTCACCCACACAATATAACGACACTAATGCAAGCACAGCATCA
AAAGACGGTAAAATAAACCTCTCTTCACACTCACACTTACGTGAAACCAAGC
CAACACAGCCCGCCAAAAACCCCTCACCCCTCTCCCAGAAGCAAATAACAA
GCATAGCAGCCAATACACTCACCCACACAAACAAACACACAGCATCACAAA
ACTTTATTCCCGCCAAAACACATATAAACATACCCACACAATATAACGACACC
AATGCAACCAAAGCATTAAAAACCGCAAAACCAGCTTCTCCCTCCCACACAT
ACTCACGCCAGACAAAACCAATAAAGCCCGCCATAAACGCATTGCATCCCGC
CCAAGACACAAACCCAAGTCCAGCAATCAGTGCTGTCACTTACACAGACAA
ACCCACAGCTACTCAGAATATTTTTCCTGCCAAAACTTTTGCAGAGCTGGTG
AGAGAAAATGCTAAACGCTCACAAACTGCAATGCAAAATCCCCCTCATGCAA
AACATGACTCTGCCGCCCTCGGACGCTTACCGAGTGCAGCTAGAAAAAACCT
AACTAAAACACTGAGTTCTCCTAAAACTCCTGGGAAGCGCCGCGGGGACTG
TTTGGATGAGGGCTTACTTCAAACCTCTAACAAAAAGGTTAGAATACGCGAC
GACTTCTCTGATGATGATCTGGGGGTCACAAATCTACTCTCCGAAACACCCAT
ATTCAAAAGCAAAGTAGCTATTAAAATTCGGCAAGACTCAAGAAGAGAATCC
CTGCAGAAGTCAGTTGAAATGGACACAGCACCAGCAATTAGTCCCTCAAAC
ACAGCAGCTGAGCCCGACCTACCGCCCTGGAAAACTGTTCCAGCTAGCAGA

AAACCACCATCAATCTTCTTGTCCAATATTCAGCAGATTATCCCGCTAATAGAA
AAACTAAACTATAAAGCCGGGGTAAATAGCTTTACTACCAAATCTGAACTTGG
CAACAATATTAGAATCCAGGCTAAAACGATGGATGCCTATAAAGCAATTCAGA
ATGTCCTCCTTGGGGCAAACATTCCCCTACACTCTCACCAGCCAAAGAGCGC
AAAGGGCTTCCAAATTGTAATTAGGCACCTCCACCAGTCAACCCCGACCAAA
TGGATTGAAAGCCAACTTCAAGACATCGGTATAGCTACAAAATTCATCAGGG
CAATGCAGTTTAGGGACACGAGAAATCCTATGCGCATTCATGAGGTTGAGGT
TGTACCCAAGGCTGACGGCAGCCATCTCAAGGTCCTGCTACTAAAATCCCTT
GGAGGTCAAACGGTCAAGGTTGAAAGGAAACGGGTATCGAAGGATCCCACA
CAATGTCATCGCTGCCAATGTTTTGGGCATACAAAAAATTATTGTAGAAACCC
GTTTAAATGTATGAAATGTGGCCAGCTGCACGCCACGGTTTCTTGCACTAAAC
CCAAAAACCTTCCGGCTACTTGTGCAAACTGCAATGGAAGCCACGTAAGCA
GCTATAAAGGATGTCCTGCTTTCCAAGAAGCAAAGCAAAGACTATCTATCAA
CAAAACTCAATCCCTACACTCACAACCCACGCACCTTCAGACCCCCCGCAAT
AAACATCCCTACCCAAAACCCACCCACTTTCAGACGCCCCGCAATAAGCAGT
CCTACACACACCCCCCTCCTCGAACAACAGTAAACAACACAAAATTACCTGC
CAAAAGAATCCAAGGAAAAAAGTTATCGCAAAGGAACATATCTATAAATAAA
CGCTTAAATAGAATTAGGGCTTTTGACAAGAAACCGAGAAAGGAGACAAGC
CCGCCGACAACTAGCAAAAAGGTACTGGCCTCCCTAGAAGAAAGCAGCAAA
AACCCAAACAGCGTCCTAAACCCAGCCAACACCCATCTCACTCATTTCTGTC
CACCCCCAATAACACAAGATATCCCTAATGACGAACCCACGGAGCCGAGTCA
GGAGCAATACCTTCTAAATCGTATTGAAGGGATGGAAAAGAAGCTCAACAAT
CTCCTGGAAATCGTCACCCGCCTACTAAACCAAGGAAGAGAGTGTCCAAAAT
CTCCAAAAAATCCTTTCCGAGATCCAATCCTCATTTAAACGCTCTTGCTAGTA
GCATCAGAAAGTGACGTATCTTATGACTCGGGCGTGCAACAGGGATATCCTTA
AAATTGCTTTCTGGAACGCTGGTGGGATCAATAATAAAATAGACGAGCTCAA
ACTGTTCATTCTAAATATTGATGCCCACATAGTCATAGTCACTGAAACCAGAC
TTGACAACAAATCGACCAAACTAGAACTTCCAGGATATTTCACATACTTAGCC
CAAAATCCTGTTTCTAGCAAGAGAGGAGGAGTCGCCACTATAGTAAACAGCA
GTATCCGACACATGGCCTTAGAACCGATTGAAAAAGAATGCATACAGAGCGC
CCCAATAGTCTTGCTGCCTGAAAACAACAGACGCAGCGAAATGATAGTAATA
GCATCTGTCTACTGTCCGCCTTCGCTAAGCTGGTCGCCCCACCACTTTACTGA
CGTTCTCAATTTTGCCGAGAAAACTCTGGGAGGGCAGACAAAGTTCATTCTA
TGTGGCGACTGGAATGCAAAACACAGGCAATGGGGTTGTACCCGCGCCTGTC
AACGCGGCACCGCACTCTACGAAGCAGTTCAAGCAGATCCCATGGCTGAGAT
TATTGCTACCGGCTGTGCGACACATTTCCCGCACGACACACGGAAAAACCCG
TCAGCAATAGATTTCTCCATATGCAAAGGACTGGGCAGGCTCGAAAAGAGAA
TTTCCTCAAGTGCGGACTTATCTTCAGACCACCTTCCCATCCTGCTTGAGATC
AACTTAGATACAAGCAACCTCTTTCTGCAAAAGCAAAACAATAACATCCTCA
AGAAAACTACGAACATTGAGCTCTTTAAGACTGTTCTTGAAAGAAAGATACT
TCTTAATACGGAGATAAGAGTAGCAGAAGACATAAATGACGCCATAAATATCT
TTATCAAAAACATCAAGGACTCGGCTGATGAATCAACTCCCTCCCCAAGAAT
CCCTGACAACCTCAGAAGAATGCATGGACAAGCTAACAGAAATAGTCACAC

CCTCACACTAGACGAAAACACAAGCAGATTGTTGGAAGAAAAACGGATACT
AAGTAGAATTTTTAAAGCTACTAGAACGGACGAGGACAAAGCTAAACTAAA
AGCAGCTGAAAATCGACTAAAAAAAGCGGTAAAGATCTTGAGAGAAAAGAG
AATTAATAAACAAATTGAAGGAATAGACACGAAAAACCCGGACAGAATGAG
GAAAATGTGGAGACTGCTAGATGAAGGAAAAAAGACAAACCAACCGAACTT
TCCCCTCAAACTGGAAACCAAAAGAGGCCCCAAATGGACAAAAACGATAAA
GGAGACAACAGAAGCATTTGTTTCCCACTTAGAAGGAAGATTTAAGCCAAAT
AACAACGTACCTGATTACCACATAAATACAGTTAACTCTGGACTAAGAACAAT
TAAAGAAAGCATGTTAACAGAAAGATATGATGTAAACAAGAATCCCTGTAAC
CAACCTATCACACTAAAGGAATTAAATGACGAAATAAAAAACTTAAAGAATA
GTAAAGCACCGGGAAAAGACCTTATAACAAACCAGCTTATAAAAACCCTACC
GACTAAAGCTACACTGTACCTTATCCTAATCTATAACTCTATACTTAGAATTGG
ATACTACCCAGACGCCTGGAAACATGCCCAAGTGAAAATGATCTTAAAACCA
GGGAAAAGTGTGAACGACCCGAAGTCATATAGGCCGATCAGTCTACTCTCGG
GACTCTCTAAAATGTTTGAAAGACTACTTCTAAAAAGACTTTTTAGGGTAGAT
CTATTCAAAAAAGCCATACCACTGCACCAATTTGGCTTCAGAAAAGAGCACG
GAACTGAGCAGCAAATAGCCAGGGTCACCCAGTTCATCCTCGAGGCCTTTGA
GCGAAAGGAATACTGCTCAGCGGTTTTCCTTGACATCTCTGAGGCTTTCGATA
GGGTCTGGCACGAAGGCCTTTTACTTAAACTAGCTAAAATATTACCATACAAC
CTATACATTATTCTCGAAAGCTACCTTACAAACAGAACATTTGAAGTTAAAGA
CCAAGCTGGAGAGACTTCGAGAGCAGGACAAATAGGCGCAGGGGTGCCCCA
AGGAAGCAACCTTGGACCCATACTGTACTCTATCTTCTCCTCAGACATGCCAC
TCCCACACATATACCACCCCTCCCCAACAGAAAGAATTATGCTCTCAACATAC
GCAGACGACACTATAGTACTCAGCTCAGACATACTAGCAACTGCTGCCACAA
GAAACAACGAAAACTACCTCAAGACATTTTCGGACTGGGCAGACAAATGGG
GTATCTCAGTAAATGCTGCTAAAACAGGACATGTCATTTATACATTAAAAAAC
GACATACCTACAAATCTAAAGACCATGAAGATTAAGGGTCAAGCAATAAAAA
AGGAAAGCAAGCAATCATACCTTGGCGTAATCCTTGATAGCAAGCTAACACT
TAGTCCTCACGTCACGAAGGTAGTAGGTAAATACTTGACAGCCTATAGAAAA
ATGTCATGGATCTTAAATGAAAGAAGCAAACTCCCTACTAATACTAAGATGCT
GATCCTCAAATCAGTTCTATCGCCAATATGGCAGTATGCCATAGCAGCCTGGG
GTCCCCTTGTGACAGATGCACAGATAAGACGGATACAAGTTGAGGAAAACA
GAAAAATGAGAGACATATGCAGAGCGGGAAGATACACGAAAAACCAAACTA
TAAGGGACCGCTACTGTGTCAAAACAGTAGAAGAGTTCTATCAACAGGCTGT
CCACAGGTTCTCAGAAACTACAAAATCGCACCCTAATGTAGCTGTTCGCAGG
ATATTCTCTAGGCACTATATCCCGAACAGACTAGAAAGAAGCAGGCAGAGGT
ACTTGAAAATGACAATGGACCATATCACGCAAAAACAGACTGGACTGACCCT
CTCACCTAAACTCTTAAAAATCCCTGATCTAGATGACTGCAGAACCTTAAAAA
AGCGAAGCGAGAGAGAGAAAATAAGACAAACGCATCTAACTGAACTCCCCA
CCCTGCTGAGACTAGAGGAAGAGGAGGCAGAGCTCAAAAGAATAAAAAAA
CAGGAAGAGAGGGAAAGAAGGGAAAGGGAAAACCAAAAGTGGCCTCCAG
ATAGGTGGTGTGAATTGGAAATAAACCGATATAATAAAAAATATAGAAATGGC
GATCTAACCAGGCAGGAAATTATAGAAAAATTCCGAGGGCAACCATTAAATG

TACAACGAATAATCCTACCCGACTACGAAGGTGACTAAAAGTAAATCAAAAC
AAACTAGGGTCGGAGCATAAAGCAGAAATAATTAACCTAGCGGAAGGGGCG
GCAAAAATATTTAAAAGAAAGGAAATAAATACAAAAAGGCTAAAGGCTAAGT
TACAGGTTACATAAAAAGGGAAATGTGCTTATAGATATAATGGTAAAATTAAC
TTAACTAAACACCTACTGGAAAACAAAACCATGATGGCTGCGTACTTAAAAC
GTTTCTCCTGGCGCGATTGAAAATCCATCTTTCCTATCCAACCGAGGGATCTG
CGACTGCGGCCTTCTGCCGTTCGGCTCCTGATGGATCCATCTGCTGCTGGAAT
GGGTGCCACACCGGCGCTCCAACCTAAAAGAAAGATAATATGTCTTAGTTTT
CTTTCCCTTTTTCTCATAAAAAAACAACAGCTCATCTGGAGTCTGATAATAGC
TTTAGCTCACCAAGACAGCAAAAACAACAGCATCAAGGCCAGCAACAGTGG
AGGCAGAGACCAAGCGGTTTGCTGAACATTTCGCTGCCCACATCCACCATTC
CTGGAGGCTCTGAATGAGGGAGCTGAAGCCTCTGAAAATTGAAGATCCTACT
CCAATGTGACACTTTCGGCTCCGAGCTCAACGCCGGCGCTGCAAATCCGGCG
TTATAAGGAAAGGGTGCTCCAGCTTGTGGAGTGGTCTTTCCCCAGAGGCAGC
AGCAGCAGCAACGCCAGCGGCAATTGGCGACCTCTGCGTAGCCGAGTCAAT
CCCGTTATCGGTCCGTCTGGCGATGCGTTCCGACGCGGCGGTATGCGTCCATA
GGGACCGCCTAGAATGCTTGGCCAGGAGACAGCAGTAGACCTGGGCGTGGG
CGTCACCAGCAACATGGTAGCCATGCGCAGTCAATTGGCCGGTGGCAACAAT
CTTGCCATGCAGCAATAGCGGCGCATGCTACCCTTGGAGCGACGTCAACAGC
TACATATTTTTCAACAGTGTAGCGCGACCTTTAGGCGGCTACACCTACACCAG
CAGCAGCGATATTGGCAGTAGCAGCATTTAATGTCTTGCAGCAGTTTCCATTT
GACAACTTCCGACGACCAGACTCCTCGCTTGGCCCCTGGCGATTCGCCCTGG
AAGAAAGAAAGAAGCATGGATTAGTCAAATATCAAAGCACAACAAGCATGC
ACGAAATCTTCGCAACAAGGACTATTCCAAGGCAAGCGATGGTGTGTTCTTT
GGCTCTACATATATCGTTGAAATCCGTCTCCAACTGCCGCGAGTCACCCACGA
CTTCCACTCCCTGCAGACGGACGTAATGCACTACGATGTGTAAATTTTCCGGA
TCCAAGTGAACGCCAGGCGGAAGAGGATTCCCTGGATGTGAAGGATCACAA
ACTGCTGCTTTTTCAAGAGTCCCCATTTAACAACTTCCGACGACCAGACTCCT
CGCTTGGCCCCTGGCGATTCGCCCTGAAAGAAAGAAAGAAGCATGGATTAGT
CAAATACCAAAGCACAACAAGCATGCACGAAATCTGCGCAACAAGGACTATT
CCAAGGCAAGCGATGACGGACGGAATGCACTACTATGTGTAAATTTTCCGGA
TCCAAGTGAACGCCAGGCGGAAGAGGATTCCCTGGATGTGAAGGATCACAA
ACTGCTGCTTCTTCAAGAGGTAACCGGCCTCATTTCCACTTGGGTCACATCGA
TTGTGGAGGAAGCCGATTGGGACTTTGAGCGAGCCCTAAAATTGTTCATTCA
GAAGAACGCTGATCACGAAATCCCAGATCTAGCATTCGCCTAGAATATGTGAT
CTCGTTGGACTGCGTGCTGGACTGCGGCGCATGCTTTCCCTCCCGCCAGAGA
CGGGATGCGCGCATCAGGATCGACGGGACAGACCGGCCGACGCCTGGCTTC
ACGTCGGCATCTATGCGACGACGTGATAATCAGCTTCACAGTACCTGCGGATG
AGGGCACAGGCGCCAGTCCTCTGCCTAGCGACACATACACCAACAGCAACA
TCTAAAACAGTCGCAGTAGATGCAGGGAATTTTCTTGCTGCAGTCTCCAACT
GGAAAAATCCGACGGCCCAACTCCTAGCTTAGCCCGGCCAATCTTCTTGGAC
TGGCAAACGATGTGATGAATCGTCCTGGAAGAAAGAAGCATGGATTATTTCT
TCATACACAGCTTGTCTACTAAAATGCATTTCCCGGCAAACTGGCACTGGGGC

GTTGAAAATACGACACTACTCACCAAACAGCAGCAAAAACAACAGCATCGG
CAGCAGGCGACCAGCAGCTGCTCTGGCTCACCCAATGCGACAGCAATATCTG
CGGCATGGCCAGCAACATGCAGCAAGCAAAAGACCGGCATGGTGGTGAACA
ACATATCGCTCACGCCCAACGATCTGGAAGCCCTGAGCTAGGGAACTGAGTA
TCCGCCAGCGGATACTTATGCAGCGGCGAGGCGTCGGAAATCCGATAGCCGG
AAGAGAGGCCGGAGCAGCATGTTGGGCATTTTCGCCCCAGTAGCAGCTGCTG
GGGCAGCAAGGGCAGAGGTAGCAGTGTCAGCGCTCCTGGTAAACCGGCCTT
TTCTGTTGAAGACGCGGGACTGCGGCGCAATTGTTCCTGCCAACCGGGAGTT
CTCTGCGCCTGAGGTCCTGTTGTGCTTCAGCGGCTCCATCTGCTGTTGCATCT
GGCGCTTCTCCAACTTTCCTCCCTGAAAGAAGAGAATATTAGTAGGAGCATG
AGACCAACAACAGCCCTAGCTTACCACGTCAGCGGAAACAGCAACTGCAGA
AGCCCGGTAACTTTCTGATAATATGCAGCAATAAGAGCGGCTGGATCAGCATG
TTGTGTAGCTTGGCCCCAGAAGCAGCAGCTGCGGTACCAAAGGCAGCGGAA
GCAGGGTCAACATAGTCTGCTATGCTGTGTCCATACTAGCATAGTCAAGTCAG
CGGTGGTGACGTTGCCGCAGCAGCTGATCCACAGGGTACGGCCTGGAATGTT
TTGTCAGAGACGGCAGTGGCCTGGGCGTATTAGTCAGATACGCCAACAACAT
GATACCCATGCGCAACCATTCTTCCGGTGGCGGCATGCTCGTAAAGTAGCGG
CGGCGCATGCATCTCTCGAAGCAGCAGCAGCAACAGAGGCGTCTCATAAGTG
CATGCGCAGCAGCGGTAGCAGCGGTGGTGGCAGCAGCAGGGAGATTTCATT
GCTGCAGTCGCCAAATTACATGCATCCTACGACTCGACTCACGCTTTTTGGCC
CCTGGCCGATCTTCATATATCTCAAATCGACGGCTGCTTTTACCCTGGAAAAA
AAAAAGGAGCTTGGGTTATATTGAACTACACTAGGCGGCGACACACTCACCA
GCAGCAGCCAGAACAACAACAGCGGCAGAAGTAGCAGGAAATTATCTCACT
GCAAAATCCAATTGGCACTTTCCGAAGTCCGTATTTCTCGCCTGGCCCCTGGC
CAGTAACAGGGGCCTGTGCGACGGAGATGACGACGATGATCTGCCCTGTGG
ACGGCCTCAAGAAGGAATCGGCCCTGTACTGCCCACCTGACCTGTGCCAGG
ATAGCAGCAATGTCAGCGGACCATGGATGGAAGAGGCGGCACTGGCGACGG
TCCTCAGTTTTGCGGTTTCCGCGGGGCGTAGGCGCAGTAATCGGCGGCAGCA
ACAGTGGCGGCAGCAGAAGGGAATGTGTATCTGCAGTCGCCGAATGGCAATT
TCCTGCCCAGCTGTAATGGACCGACAAATGTTTTTTGGCATGTCGACCTGTAA
GAAATAAGCAAGGATAAGTTTTTTCTTTACAAGATTGTATATGAATTTGTATTT
ACGGACAACTGGCTTGTTTCCTGTCGACGATAAATGGCGCTTCATGTTTTCAC
TCTGCCAAAATCTGAGTGCAAGCATCGGGGCTGACGGGCCGAATTTGCTGAT
GCCTTGTACATGCTAGCATCTTTATGCCGCCGTGTAATTTTGCGACAGCAAAA
CACATGATGGAGAAGACGAAGGTGATCGATGGCGCCCGGATGACGGAAGCA
GGCCCGTGCTACCCACTGGTCCGTTGCCAGGACAGCAGCGTTTCAATTATGC
GAACAGAGGCACTGGAGCCACTTCTCAGGCGATAGGGATCATGAGGCGACG
ACAACTCGCCAGCAGCAGTAACAACAACAGCGGCAGCAGCGATCTCGGACT
TGCTGAACATTGCGGCTTTTTCCCAGCCTGGACCGTCATCACAAACGCTCTC
AAAGATCGGTGCGCCTGCAGCCTACGGAAGCGTACAACTGCCCAAAGTCGT
GCTATTGCATAATGTTAGGTGTCCTGGCTGCTCCGGCGGATGAGCTCCACCCG
ATTGAGAGAGGTAAGAAGGCCCAGCAAGTCGCCCTTAAATAAAAGAAACAA
GCATTAGACTCGGCTGAAATTGTAACATCAGTCAGACTGTACTCACGCATGCA

CTTAGACAAACCGAAGCCTATTCCACAAAAGGCTGTGAGTGCACCAAAGTT
GACGGGCCACGCCGGCCGATGCTTGCTGCATACCGGCATCTCCGCATCAGCG
ACGTTACAAGCACCACTAAAATTAGAAAGAGGCTTACTATAGGCAAAGCGCA
GAAAAACAAATATTACAATAAGAAATAGCACGCGATTTTTCTGCTTCGTTTCC
GCTTGGTACAAGTGAGACCATTCTCCCTATATGCCATTCTAGTATATTTGCCCG
ATAAGCGATATGCTGGCGGCAAAAAAAAAAAAAAAGATAATAGCCGATAAGC
CCGCCAAAAAAAAAGCGCGAAATATCGATAACGAGCTGGCACGAAAATTGCT
GGCATATCGATCAACCGCTGTGTCTTTGAGCTATCGAAAAAGCCCGCCTACGC
GCCTAGCCCAAATTTGCAAGTCAGTTGAGATTATAAAACCGACAGAGGCGCG
CGCGCAACAGTAGCTAAAAGTGAAGCCAGCAGCAACAACAACAACTAATGG
ACGGCCAGAACGTGAACCAAAGCGGAGGATGGGCATTGGTCTTATCCATCTC
ATCGGACGATGGTAACTGCTCATCCTTGCCGCCATAGTCTCATCGCTGGATATC
AAGTCATCGCTCAACTATACCTTAAAATTACTCACCAAAACTCTTTTCTCCTTT
TCCATAAGATAAAGTAAAGTAAAGTAAAGTAAAGTAAAGTAAAGTAAATTAA
AGTAAAGTAAAGTAACGTAAAGTAACATAATATTTAAATTTAAATTCTACACTT
GAACAATGGCGCGCAAAACTGCCGCAATGTAATTCAAAAATGTCTCCCGCAA
ATTTAAAACATAGATTAATACTTGTAAGTATGGCGCGCATATCTGCGGCCAAG
AAATCCGAAAATGTAATTTGCCACAAATTGTCACTATATATTTTTAAGCAAAA
ATCAGAGGACAATAAATGCCAACGTCATTAGACGTTGAAAATATTTAAAAAA
AAAAAAAAAAAAAAA

>TAHREJockey#LINE/TAHREJockey

TTAAAAGCAAAGTTTTTTAAAAGTTCAAAAATATAATAAAATAAAGCAAATTA
AATTTAATTAATAAAACAATTAATTTTATTTAATAAAATTAAACGCGCTTCGTC
GGCAAATAACTCTCACGCGCAAATTTTATTAAATTCGCCTTTCAAGTTGAAAA
ATTAAAAGTTAAAATCGTCTTCCGGCCGCAAAGTTTGAACCGCGACGATACA
AACATTTAATAGACAAACAAAAAGCGAACAATAAATCAGTGAATTATTTGTG
CAAGCTGCCGCCATAACCAAAAGGAGAAGAAGCCAAAAGACGAAGAGGAG
AAAGTAAACCAGGAGAAATTAAGAAGACAAGGAGGAAAACTTATTTAAAAA
GACGACCTAAACTGGAGGACAAATTACAAATTAAAAGCCAGGGTATTTATAC
CTTACAAGTATCGACCTAATATAATATAAATAAATATGTCCACGTCCGACCACC
TATTTTCTGACGATGAGGTTCACTCAATCTCCTCAAGCCCAGAACAGCGAAAT
TCACCATTCCACCTTGAAATATCGCCCATGTCCCATGAATCTGACAATTCTCA
GTCTAATATAAGCATTATTAATCTGAGGAAATTGCCCTTAAAACCAACAAATA
ATATTTCAAAATGCTCTTCTGGGACTGCCATAAATATTTTTCATTCCCTTTCAC
ACAAGGAGAAAGAGAACATGAACACCAATATTGCCCAAAAAGACCCCCTCT
CGATCGACAATACAGCTGCAGACACGGACGGCGCCAAAAGTAGCATCTTGA
AGGGGAAATTGCCTTCCCCTCCGCTCTCATCACACACATACAAGGGGAAACT
ACCTCCAGCAACGACCCACACTAACATATCTGCTCTTACTCACACTGACGCTT
CTCAAAGAGAAAAAATACCCACCTCAGTGATCTGCACTAATGCAGCTGCAGC
TACTAATACAAATGCAGATCTAGGCGCCAAAACGAGCGACGCCTTGGGAAAT
TTCCCCTCCCTCTCACACAGCGATCATAGCATGGAGAACAACCTAAGTTCCTC
CACCAAAATTGGACCCAATACAAATTCCCCTTCTTCTCACATACTCACCAACA

CAAGCCAGGCCACAAACATAAGCGCAGAAAGCCGCTCAAAATTTCCCGCGC
CCACCAATACTGACGCACGCCTCAAGAAGGCCATTACTAGTGACAAAGGGG
AAATTCACACACAAATTCAAACAAACAAAAGCAAGGAACACAACCAAGAA
AATAAACCATTTAATTACTTAAGTTGCTACGCTTCATGGTCAACTTCAAACCC
AAAGCCAGACATTTCCAAACTAAGTTTAACTAGGAAATCCACCAACCGTACT
GGAAATAGTGGGAAAAGAAGCATTTCCCCCCACCAAAAGAATGCTTCGTTAT
GCCCTTCTGCTCAGGGTAATTTAAATTCAAATTTAAATTCAAATTCAAATCCCA
AATCTAGCGCCACTCCCACTGAGGTGAATTTATCAGCAGCCCGCACCCTCAG
CCGGCCGGCTGCCAAGCGCGATTTATTTAATTCATCTTCCAGGAGCCCAGAA
GAGCAGCCTATGAGTTTTTCGGAAGTGGTTGCTGGAACAGGTCCAGATATAA
TAGCACCCTCCGCTCCTGCACCACTAACGAAAACTCCGGGCAAACGAACAA
ACAGCGATCTGGACTGCTCTAGCTTTAAGACACCTAATAAAAGATTACGTGCG
ACTCCTAACTTTGAAACTCCAAGCCTTTTCCCCCCGCTCATTACACCCGTTTT
TAAAAGTAAGGCGGCTCAATCTGTTTATGAGGAGTCCAAGGCCAGGAATGGA
CCCCCCCGCCAGCCGTTACCCTGCAGCAACAATGCTTCTGCTCGCAGCGCAA
CAGCACCACCCGGGATTGCCCCCCTACCCCCTCAGAACACAGATGTAGAGCT
GCCCCCCTGGAAAATCGTTCCCCAGAGCCGTAGAGCACCCCCTATACTAGTC
AACAATGTGAGGGAAATTGTCCCACTGCTGGAAAAGCTGAATTATACAGCAG
GAGTCTCCAGCTACTCCACCAGGGCAACAGAAGGAAACGGGGTCAGGATCC
AGGCCAAGGATATGACTGCCTACAACAAAATCAAAGAAGTCCTGACCGCCA
ACGGTTTTCCTTTATTCACTAACCAGCCCAAATCCGAGAGGGGCTTCCGAGT
CATCATCAGACACCTCCATCATTCCACACCATGCTCGTGGATAGTCGAGGAGC
TGCTGAAGCTCGGATTCCAAGCACGCTTCGCTAGAAACATGACGAATCCAGC
TACAGGTGGCCCCATGCGAATGTTTGAAGTGGAGATAGTCATGGCCAAGGAC
AGCAGCCATGGCAAAATTATCTCATTGAAACAACTCGGTGGGCAAAGGGTGG
ATATCGAAAGGAAAAACAGGACTCGGGAGCCGGTCCAGTGCTACAGATGCC
AGGGCTTCAGGCACTCCAAAAATTCATGCATGAGGCCGCCCAGATGCATGAA
ATGCGCTGGCGGTCACCTGTCATCCTGTTGCACAAAGCCAAGAACCACCCCT
GCCACCTGCGTCAACTGCTCTGGTGAGCATATTAGTGCGTACAAGGGATGCC
CCGCTTATAAGACTGAAAAACGAAAACTGGCGGTCAACAACATTGACATCAA
TAAAATAAGGACAATCAAGGACGCGAACATTACCAACTATGGACGACAGGGC
CCTCCCTCTCGCAACAACTTTCCCCGGCTACCATTTAGCTCCTCAACCTCCAA
CAGGACAACAGCCGAATCCCGCCAAGACACAGCAAGAGCACGGCGGAATAA
CCCCTTCCGGCAAAACAGAAACGAAGCAAGGCCAATCCAGCCACGCTTCTC
CTCCCACGACTTTGCCATCCAGAAACGTCTGAATAAATGGCGCCGCAACTCT
GACAACGTCTCCAAAAAAGGCACGATAAATCCCAAGGACAAGCCAAAGCCA
CGAACACCTAACATGACGAGCAATCCTGCACAAAAACACCTGGAAATGTTCC
AGGAAAAGCTCCGCAAAGCAAGATGTGAACGCAAGGAACAGGACCCGGAA
GAAAAGAAGACAAACATCAGGATGGGAGACGATGAAAGCCCGCCAACCAC
CAGCAGAGCTGCCAGAGCATTCCTCAAGCCAAGGATCATAGACGACAACATC
CCCACGCCTATGGATACATACTCTAACCCGCAAAAGAGTCCCTCTGACTTCGA
CAGCAAAAGCCTTACACAACGAGTGGAAAATATTGAAAAGAAAATTGATAAT
CTAATGGAACTATTATTCAAGTGCCTCGAATCCACGAAAGAGTCTACCTTAGC

ACACCTCATGACCTCCTAAATCACTAAATTCTCTACGCAAATTAAAATACTACT
ATGACCTTTAATCTAAACAACGGACAAGCTTCCAATACCCTAAAAATAGGGTA
CTGGAACTCATGTGGAATTACAAATAAAACAAATGAACTGGAGGCCTACATC
AAAAAAGAGGGTATTCAAATTATGTTAGTAACTGAAACCAGGTTAGAGCGAA
ATTCCAACGCACTTAATATCAAAGGCTTCCACACCTACCTAGCACAAAACCCT
ACATCCCACAGAAAAGGCGGCACGGCCACCATAGTCAGTCAAAACATTAGA
CATGCCTGCCTAAATCCCATAGAGACTGACTTCATGCAGAGCGCACCCATCGC
ACTTATCCCCTCAAGTCGAATACGCGCAGACATGACAATTGTTGCCCCAATAT
ACTGTCCGCCTGTATATAAATGGACCACTGAACAATTCTCGAAGCTCTTCAAC
CACTTCGAGATGCTTCTCGATGGTAAGTCCAAATTTATACTTGGCGGCGATTG
GAATTGCAAACACAGACTCTGGGGTAACTATTTATCCTGCGCTAGGGGAAGA
TCTCTGTCACAGTCAATCCTTGCAAGGAAAGACCTTGACATAGTTGCTACCG
GCCATGCCACACACTTTCCCTTCGACAAGAAAAAACAGCCCTCTGCACTGGA
CTTTGCAATATGCAAGGGCTTCCACACTCAAAAACTAAAAACCTACTCCACA
GATGAACTCAGCTCTGACCACCTTCCCATCCAAATAGTCCTAGATCCTGACGA
CTCAGACTGGCAACATAATAAGACAAACAATGCTATTATACAAAAAAGAACA
GATCTCACTAAATTTAAGAAAAACCTAGAAAACAAAATATTACTAAACACCG
AAATTCGCACAGGACAGGACATCGACGACTGCATAGACATTCTTATCAATAAC
ATAAAATCCGCAGCCAATGAAGCCACCCCACCTAATCGCCCCCAAAATAACC
GCACCTACAATTCTTCAAGAAGATCCACAAACCTAAGGCTAGATGAAGCAAC
TAAAAGGTTACTTGAGGAGAAAAAGGAGCTAAATCGAATCTTTAGAGCAGTA
GGAACTGACGAAGCCCGAAGATGGTTTAAGAACGTGCAAAATAGACTGTCT
AAAGAAATCAGAAAGCTCAAACAAAAACTACTCAACCGCAACCTACAAGAT
ATTGACACTACGGACCGATATAGAACGCAAAAACTATGGAAAACCACAAATA
CAATTAAAATGCAACCTCGACCCTGCTGGCCGATAAAAAAGGACAATGACGA
CAGCCGTCAAAGAACTGACTACCCCTGGACCAGGACTTTAGATGAAAAAGC
TGAAGCCTTTGCATCTCACCTTGAGTCCCGCTTCAGGACAAACCAAATTAAC
GACGCCAAAGACAGGGAATTCGTCAGAAATGAGCTAAACAAATTTAAATCAT
TAAATGCGAGCGGCGAATCCGGAAACAGCAACTTCAAACCAGTCACTCTGG
CTGAACTAAATGGCCTGATAAACTCACTGGAATTAAAGAAAGCCCCAGGAAC
TGACAATCTTAACAACAAGACCATAATAAACTTACCTACAAAGGCCAGAATAT
ATTTAATACTTATTTATAACAACATCCTGAGAACTGGACATTTCCCGAACAAAT
GGAAGCACGCTAGCATCTCAATGATTCCCAAACCAGGGAAATCACCATTTGC
TCTAAATTCATACCGCCCAATCAGCTTACTCTCTGGTCTTTCCAAACTACTCG
AAAGAATACTACTGAAACGACTGTATGACATTGACTCTTTTGCCAAAGCAATC
CCTTCCCATCAATTTGGTTTCAGAAAGGATCATGGAGCGGAACATCAGCTGG
CCAGGGTGACCCAATTTATTCTAAAAGCTTTTGATGAAAAAGATGTCTGTTCT
GCCACATTCCTTGACATTACGGAAGCCTTTGACCGAGTATGGCACGACGGCT
TGCTATATAAACTATCCAGACTCATCCCCAGATACCTATTCGACCTACTTGAAA
ACTATTTATCTAATAGAACCTTCTCAGTAAGGATCGACGGTGAAACAACGTCT
AGGATAGGTAATATTAGAGCAGGAGTGCCCCAGGGCAGCATACTGGGACCGG
TCCTCTACTCAATATACTCATCCGACATGCCCTATCCCATCGTAAAAGACTATA
TGCGTAACATATCCTTCCCTGATTACCACCCAACTAATATTATCTTAGCTACATA

TGCAGATGATACCATAATTCTTAGCCGGTCCAAATATACCAAGCTTGCGATCA
ACCTAAATCAAAACTACCTTAACGTCTTCTGTAGGTGGTCAAAAAAATGGGA
CATAGCAATTAATGCAAAAAAAACCGGACACATTCTTTTCTCCCTAAAAAAA
GAACAAACTAATATATACACTCCCCCACTAATCAACGGACAAAGAGCTGCCA
AACTAAACAAACAACGCTATCTCGGACTTATGCTAGACAGAAGACTGACCTT
TTGTGCACACATGACGCTGCTAAAGGGAAAGACTATAGCTGCATATAAAAAA
CTGGAATGGCTAATAGGAAAAAACAGCCACCTACCCAAAAATGCAAAAATTC
TCCTCTGGAAGCAAATTGTCTCCCCCATCTGGCATTACGCCATAGCAATCTGG
GGCTCGCTGGTATCTGACACCCAAGCAAAGAAAATTCAAACAATGGAAAAC
AAATACATCAGACGAATCATAAACGCCAGCAGATACACGAGACAAGCAGAC
ATAAGGACAAAATATAACATTAAATCATTTGATGAAATTTTTGACAAAGCAAG
CCAACGCTACGCCAACTCCCTCACTGACCATGAAAACCCTTTAATATATGACC
TCCTTATCAACGCCTACAAGCCGAACAGACTGGAACTAAGCAAAAACAGATA
CGTCAAGCAATTATCAAAATATATACTGCCCCTTCAACAACACCGACCTAAAC
CACCCGCAGAACCCATCTACAGCTCCATACATAATTATACAAAAAAGGAAGA
AGCTGAAATAGTCGCCAAAATGAGAACCAAATTCAGACAGACCCTTCCAACC
CTCCTCCGGATCGCTGACCAAGAATTGGAAATAAGACAATCCATAGCTGAGG
AAAAGAGAAAGGAAAAAGAAAAATCCGAAAGGAGAAAAGCCTTAGAGAAG
GGACCGCCAGATAGATGGTGTGAACTTCAAATAAACAAATACAGCAAATTAT
ACAGAAAAGGACTAAGGGCCAGAGAGGAAATCCTTGAATTAATGCTCGGAC
AACCCGCCACTGTAATCAAAATAGTAATCCCAGACTATGAACCCGAAGACGA
CCTAACAAAAAAGTCTTGAAAACTAAATAAAAGCAAATAAAACCAAAAGTA
CATGTATTTACAATAATCATTGATTGTATAATTGGTGATTATAATATAACAATTAT
AATTATTAATATAATTGATTGTCATAATTGTTAGCTATTGATTATAAATAACTAAT
CAAAATACAAACTACAAACTATGACCGACGGAAAGACGCACGCCGACCTGC
TTCTCTTCCTACAATAGCGATACACATCTCCTTCTCCATAGTCAGCATCTTTCT
GTGGAAAAACAAACCAATTAGATGGATGATACAAAAACACAAATAATAACCA
CACCTCAACGCATCCGGATAAAAACAACCAACGACAACGCATTCCAGCTGAT
CATGACGAAGTGATGCGAATAAAATCACCACCTGGACATAAAAGAAGAATCG
GTAGATGGATATGAAAAGGATTGGTGCGGCGAAAGCATGATGAATATAAGGC
GACTCGCTGCAGCAATATATGCACAACGTCACTTACCTGAATCTTCTTGCCGC
ACAGTCTTTTGAAGATCCTTATCACCGCTGCAATCCACACACATCGCCGCATT
GCTAAAGACAGGCCATCTAAGCTGACCCAGCGCCGATTAGGACACTCTGTTC
GACGAGCGCCTACAGCGAATTGCCGCTAAAACCTAAAAACAAAAAATTTATT
AACAAATGAAATACAAATATACAAAATTCAAATAAAACAAAGCACTTACCTC
ACTGACCGCATCCAACTGCACGCAACACATCCATATCCAACATAACAGACAA
GAGGAGACGGGCCCTCAAACGTAAAACAATATCGCCAACTTTGCGCTTACAA
ACACAAAAAAATTTACAATTTTATGATGCCGTCTCCTCTTCCCGATGCCACTG
CCTCAATATGGATTACTAGCGCGGAGCCGACAGCACACTAAAAGGCTGAAAA
ATTTGTCCTCAAAATTAATATTTTTCCTTAGTACCACTATCCCAACGAATTTTCC
GCAAACCTGAAATAAAAAGAAAATTAATAAGAAAGTGATACAAAATTAACTA
AAAACAAATAGAAAATAGCAAACCGGACAAGCAAAGTAACAGATATAAATAT
GCTACTTCATCCTGCTGAAGACACGCATCCATGCATCCTTCTTCCAAGACTGC

AAAACAGAAAGAAGGAAACACAAGCTATACTGGGAAAAATATATTAAATCAC
AATACTTATCTAATTGCCAATTTGAAGAATCCTAAACCACGGCATCCCAGCGG
CGATGGCCTTATCTTTAGCTGCTACAAATGTACCTGAAAACAAAAATCAAAA
AACAAAAAGTAATTCAACTATAAAAACAAACATAATACTTACCTCCAGACTAT
TTTCCTCCCGAAAAACATACCTGGAAAACAAAAACAATGCAACTATATAAAC
AAATAAATACACAAATAATACTTACCTCCGAACTGCATTCCACCTAATGTACCT
GAAAAAATACAAAAATTACAGAAATCACAAAAATAAATAACAAATGCTATAC
TTACCTAATTTTAATATTACACCCATTCCCATGGCCCAATCTTTGGGCGGTCCC
CAGCAACAATTCCTGACCCGGAACATTCTAAAATAATAGGAAAATAAATAAG
ATTGCGACTCAAAATTAAGCAATAACACAAAAAAAAAAACAACAAACCTGG
CAGACAAATTAGCTGACGATAAATTACAACACCATCCTGCTGACGTCACGCA
CGAAAATTCTCTCTCCTAAAACACGGGATCCCAGCGGCGATGTCCTTAACTTT
AGCTGCTACAAATGTACCTGAAAACAAAAAAACAAAAATTAATTCAATTATA
AAAACAAACATAATACTTACCTCCAGACTATTTTTCCTCCCGAAAAACATACC
TGGAAAACAAAAACAAGGCAACTATATAAACAAATAAATACATATATAATACT
TACATCCAAACTACATTCCACCTAATGTATCTGAGAAATACAAAAATTAGAGA
AATCACAAAAATAAATAACAAAGGTTATACTTACCTAAAATTTAATATTACATC
CATTCCCATGGCCCAATCGTTGCGGCGGTCCTCAGCAACAATTCCTGACCCG
GAACATCCTAAAATAAAAGGCAAATAAAATAACACACATAAAATAGCAAACC
TGACAGGCAAACTAGCAGATGCTAATTTGCAACACCATCCCGCTGACGTCAC
GCACGAAAATCCTTTCTCCAAGAGCGCAAAAACTGAAAAAAGAAACACAAG
CTAACACTGGGAAATATATACTTCTTAACAAAATACTTATCTAAATTGCCAACC
GGACGACTTCAAAGCTGCGGCCTAATTTTAATATTACATCTATCTCCGTGGCC
CAATCTTTGCGGCGGTCCTCAGCAACAACTCCTGACCCGGAACATCCTAAAA
TAAAAAGAAAAACAAATAAGAATGCGACTCAAAATTAAGAAATAACACACA
AAAAATAACAAACCTGACAGGCAAACTAGCAGATGCTAATTTGCAACACCAT
CATGCTGACGTCACGCACGAAAATCCTTTCTCCTAAGACCGCAAAAGCTGAA
AAAGGAAACACAAGCTAATACTGGGAAATATATATTTTTAACAAAATACTTAT
CTAAACTGCCAAATTGACGACTTCAAAGCTGCGGCATCATAACTACAACGAC
CTCCAAAATAACTTTCAAAAAATGTACCTGAAAAACAGAAACTAATGAAATC
ATAAAAACAAATATAATACTTACCCCCAGATTAGCTATTACCTGAAAAACAAA
AAATTAATGCAACTATATAAACAAATAAAAACAAATATACTTACCATCAAACT
ATCCATCACATTGTACCTGAAAACAAAAACAAATGCAATCATAAAAAACAAT
CCTAATGCAATACTTACCTATCTCTAACTTTACATTCATTTCCATGCCCGTATCG
CTGCGGCGGACTTCTGCAACAAATCTTGAACCGGCGGCCCCAAGCTGCCAAT
CCCGACGCAATGGCCAATAGACGAGGCGCTCCTGGCAACTCTTGGCGAACA
ACTAAGCTGCATTCTACTGACGACTCCTCTGCCACAACGAGACAGATTCCTC
CATTACGATCCCAGCAGCTTTAAGACCTTGTAACGACGGCTGCGCTGGACCC
TCCTTGTTCTACCTTCTTTTGATGACCGGCGAAGTGGCCCTGCAAATTAACAA
TTTATCAAACAACTGCAATCATCTGCCACTGAGGTTGAAATATATACCTACTAA
ATGACAGCGGCGCGGATACACTCACCTATAATAGGCTGCTTGCAGCGCTGG
CCGGACATGCATATTGCAAGTGGCACGCATCCAGAGTCCACAACAAGCCCCA
GCCAGAATGCCAAAATTACTCACCTGCAATGTTTCCTGAGGCCTCCAGCGAC

TCGGTGCTTCCGTCCTTCCGACGGGGGTACCTGAAAAGAATAAATCAATAAC
AATGTTAGTTTTAAATTTCAATGTTTTCTATAAAATCATTTCAATTGTAAATTGT
AAACATACCATACAATATGATAATGTTACCTGTCCTAATTACTGTCAAAAGCCT
AAGTCTACAAAATACTAACTACTTTTACATTATTACTAACTATGTCCAACCCCC
AAACTCACCACATGTAATGTTACATTCAAAAATGCAAATTATTGTACCTATATA
TTACAAACTCTGTAATCAAAGGCAAAATAAATTGTGGATGCGGAACAGAATT
TATTCTGTCTCCGTACCTCCACCAGCAAAGTTAAAAAAAAAAAAAAAAA

>TABOR_LTRGypsy#LTR/TABOR_LTRGypsy

TGTAATGTGCACACATATCGAATAAGCACTGTATCAAATCAGAACATTGGGAA
CAATCACATTGTAGCACTTTTGCAACAATGTTTATGTAAGACTTTTCAGTTCCC
AGGCATATCTTGAGTGCTCAGCAATCTGACCACACAAGAATGCTATTCAGAC
CAGAAGTGCAGAGTCAGCATAATTAGCATGCGCGACTGCTCGATCTTTATGTC
CACATGACTCTCTTAGACAGCGGCGCTCTCGCTGCCAAAGTTAAGTACATAA
GAGCAAAGCAACGTCTCTGCCGACGGCCTCTCTGCCGGCGCAGACGGATTG
CATTTGGCTAGCTTGGACTCTTCTAGACCAAGTACAAGGCAGTCGTAAAGGA
GTCGTCAAAGAGCCTTCAACATGTCCTAATTGAATATTAATGAGTCTTAACAG
AAGTTACAATTTTACTGATATCATACTGAATCTCTATTTCAATAAAAGTAATATA
AAGAACACAAAAATGCATTACAATTATTACA

>TABOR_IGypsy#LTR/TABOR_IGypsy

TGGCGACCGTGACATGGTCACTTAAGCCTGTAAAACAATAATAAAAAGAAAT
ATATAAAAGTTAAAATGCGAACAGTGACCATTAAAAAATAAAATTGTGACAAT
TGATCAAACCCAGACGACAACAGAAGCCGCAAACTGGAGGACTCTGCTCCT
ATCGAGCAAAGGGACGCACCTACTCTACAAGAGGCACTAGAGGTGTGTCCA
AACGATGGACCACGCCCACTCACAATAGCTGAGTATAGGGCAAGAAGACAG
CCGAAACCAAAAATAAAGAAGAAAAGAAGCGGCAAAAGGATCAAGCTTCTT
CAACAACGGAGATTAATAAAGGACCTAATAAAGACGGCAACTACGGAGGAA
GAGAAAACTAACCAAGCCAAAAATCTGGAAGCGATTGAAGCCAAATTGTGC
AGTGGTGCGCAATAACGCAGTTGAGGCTGTATTTAATACCAATGCCCCAATCT
GCCTTAATATTAAAAATACATACCTCAAGCCGATGCGTGAAAACGCTGCTTGA
AAAATACTAATCTCTGTTAGGCTTTTACTAAAAACATGTGTACGGTATTGAATT
ATATAGAGCAAAACATTGTAAACCGTACACATGCCCTCTAACTAACTTTTTCT
TGGACAGACATACGTGTAGGGAGAAATTATAGCAATATACAATTTTTAATCAA
AATTTAATAAATAAATAAATAATAATAATCGTTCAATTAACAACTATGGGTTGG
TTCGGATCTGACGATAGTCAGACCAAAGACAACACGGCTAATGTCGTGAACA
ACGTCAAAATTGTAGATCACACAGAAGATATTCAAGCACTGTGGGTTTTACTC
CTAATTTTGACAATTACAAGTGTGGCCCAGTTCCTTCTGACGTTGTACATAAA
ACACAACAAAATATTAAAAAGAAGATATATGAGTAGGGCGAATAATCTAGAC
AGGGTTTAAAAGAAAAAAAAAAAAATATATTTTAAAGAATGAGTATATAAATAT
ATACTAACAAGTAGGAATATATAAAATAGTATGGAATGGAACGAGTTATGTAA
AAACATAACAAGAATAAGAAACGAATTTGAAAAATCTCATAAATGTTTGTCG
CAAAATAGACCTATTTTAGGACCGACAACAAAGAAACATGCAAATATTCTGG

TAAATTCCTTCAACGAAGCACGAATACTAGTGTATGATAACAAGGAAAGACT
AAATCCAGATCATTGGTCTCAGGTATCGAAAGTTCTCATAAAACTTAGATCGA
ACTTGTTATCTGTAAAATTGAAACTTGGTTTGGATATATCAATACCAACCATTT
TAAATTCGCCAATAAAAATAGAATCGGACGAACAAACAGAAACAGAAATAG
AAGACGAAGATTTAAACAACTTAACAATTCCAGCTATATTAACACTAGCTGAA
TTGACGGAGGAAGAATTGGCTGAGTCAGACATAGAAGAAACAGAAACAAAA
TCTGTCATCATGGTGGACGAAGCAGCTGCCCAGAGGGCATACATAAAAGACA
TTTCAACGGCGATTCCGGAATTTGACGGTAAAAAGATCAATTTGCGTAGATTT
ATCACGGCAATCAAGTTGGTTAACCTGACTAAGGGACCACATGAAGCTATTG
CCATTGAAGTGATTAAATCAAAAATAATCGGAACAACACTCTATAGAGTACAG
AATGAAGTTACAATTGATGCCATAATCAGGAAATTAGAAGAAGTGGTCGTAG
GAGAAACAACGGACGTAGTAAGAGCAAAAATGGCAAATGTTTACCAAAAAG
GTAAAACAGCCACACAATTTACAAATGAAATTGAAAACCTACGCAAGTCTCT
TGAATCTTCATATATAGATGAAGGATTGCAACCAGAACATGCTATCAAATTTAG
CACAAAGGAAGCAATCAATACAATGACAAAGAATTGCGATCATGGAAAACTG
AAAGCAATCCTAGAGGCAGGAACATTTAAGACAATGGACGAAGCAATAAGT
AAATACATCCATTGCAGCACTGAGATGACAGGAAGTGCAAGTTCCGTTTTGT
TTTACAAGAGAGGACAAGGAAGTTACACCAGAGGTAACTACCGAGGACGAG
GAAATGGTCGAGGTGGTAATAATAGAAATAATTATAACCAGAACACCGGCCA
ATACAACAACTTTAATAATTATAACAATAGCAATGGCAGAGGACGAGGAGGAT
ATAGAGAATATAACTACCAGAACAGAGGCGGTGGTAACTATAACAACCAAAA
CCGCAATTTCTCAAGTTATAGCCAAAATGGTAATGTCAGACATGCACAAGGC
ACGTCGGAAAACCAGCAGGCTCCCTTAGGGCATCAAGAACAGTAAAACAAA
AGGTTCATACCATCAATCTTAATCTAAATATTTTCATTAAAGTAAAAAATGAAC
ACACAAATAAGATACTTACATTTCTAGTAGACACAGGCGCCGATATATCAGTC
ATTAAAGAAAACTCAGATGAACTTTTGAATCTTGATCATAATAATATAACACA
AATTACAGGAATTGGAAAGGGTTCAATAAATTCAATAGGTTTAACACTTCTCG
AGATGAGAACCGGTAACTATATAGTACCGCACAATTTTCATGTTGTGGACGAC
AATTTTCCGATCCCTGGTGACGGAATAGTTGGAATCGACTTCATAAAAACATT
CAATTGCCAATTAGATTTTACTACAGAAAGTGATTTTTTCATTCTAAGACCAA
ACAATATTAAACAAGCGATACAAATACCAATTTTTCATAATATCGATAACACTG
AGATAACCATACCATCTCGTTGTGAGGTTATTAGGCAAATTACCGTAAGTTCG
GTGGATAACCAAATTCTAATACCCAATCAGGAAATAGAAGATGGTGTATTTGT
CGGAAATTCGATATCAGATTCAAAAAACACGTACATCCGAATACTAAACACA
ACTAACAGCAATAAAATTTTAAATGTAAACAAAATCAATTTTGAACCATTAAC
TAGCTACAAAATAGCAGACCTAAACGACTCCATAAGAGCCGAATCAATTTTA
AGCAGATTAAAGAAAAACTTTCCATCTGCACATAAGAAAATGTTGACTGAAC
TGTGTTCACAATATACTGATATTTTTGGATTGGAGACAGAGCCAATCACTACA
AATAAATTTTACAAACAAAAGATAAGATTGAGAGATGATGAACCAAGCTATAT
TAAGAACTATAGAACACCCCATTCACAACAAGCCGAAATATCGAGACAGGTA
ACTAAATTAATAGAAGACAAGATAGTAGAACCAGCTGTATCTGAATACAATAG
CCCATTATTGCTAGTTCCAAAGAAATCCTTACCGGATTCAAAAGAAAAAAAAT
GGCGTTTAGTGATAGACTATCGCCAAATAAATAAAAAGTTGTTAGCTGACAAG

TTCCCCTTGCCAAGAATAGATGACATACTTGATCAATTAGGCCGAGCAAAATA
TTTCTCGTGCCTGGATCTTATGTCGGGATTCCATCAAATAGAGTTAGAAGAAG
ATTCTCGAAATATAACATCCTTTTCTACGAGCAGTGGCTCTTATCGCTTCACGC
GACTACCATACGGATTAAAAATAGCTCCAAATTCCTTTCAACGAATGATGACA
ATGGCATTTACAGGTCTGGAACCATCTCAAGCATTTCTGTACATGGATGACTT
AATTGTCATAGGCTGTTCTGAAAAACACATGACCAAGAATCTTACAAATGTTT
TTGAACTATGCAGGAAAAACAACCTCAAACTGCATCCAGATAAGTGCTCATT
TTTCATGAGTGAAGTCACTTTTCTTGGACACAAATGTACTGATAAAGGCATAT
TGCCTGACGATACTAAATATGATGCTATACAGAGATATCCAGTTCCTACTGACG
CAGACAGTGCCAGAAGATTCGTAGCATTTTGTAACTATTATAGACGTTTTATAC
AAAATTTTGCAGATTATTCCCGTCACATAACAAGATTATGTAAGAAAAATGTA
AAGTTCGAATGGACAGCTGATTGCCAGCATGCCTTCGAACACCTGAAAAAAC
AACTAATGAATCCAACTTTGCTGAAATACCCCGACTTTAGCAAAGAGTTCTGT
ATTATAACTGATGCAAGCAAAGAGGCATGCGGAGCGGTACTGACCCAGAACT
ATAATGGTATCCATTTGCCAGTAGCTTATGCATCCCGTAGCTTCACTAAGGGTG
AGAGCAACAAGTCAACAACAGAGCAAGAGTTGTCAGCAATACATTGGGCGA
TAAATCATTTCAAACCATATATATATGGTAGACACTTCACCGTCAAAACAGATC
ACAGACCGTTAACATACTTGTTCTCGATGGTTAATCCAAGCTCAAAACTGACC
AGAATGAGGCTTGATTTAGAAGAATATGAATTTACTGTGGAATATCTTAAGGG
AAAAGACAATTACGTAGCAGATGCGTTATCCAGAATAACCATCGACCAACTA
AAAAATATATCCAAACAAGTACTTAAAGTCACTACAAGAAATCAAAGTAGAC
AGGAATCCTGCGCAGGAAAAGGAAATAAAAATGATAAAGTAGAATTGCCTAA
GCAAACTACTCAAGAAGCTTCTAAGCCCAAAGTATACGGAGTCATTAATAATG
ACGAAGTACGCAAAGTAGTGACATTGCATGTAAATAATATGATATGTTTTTTA
AACATGAAAAAAAAATTACTGCAAGATACAACGTTGAAGATTTGTATATTAAT
GGAACTCTCGACTTAGGTCAATTTTTCCACAGGCTTGAAAAGCAGGCCGGTA
TGCATAATATCAATCAACTTAAAATGGCACCGTGGGAAAATATCTTTGATAAC
ATTTCAATAGATACATTTAAGAAAATGGGCAATAAAACATTAAAACTATTAAG
AGTAGCGCTACTCAACCCGGTGACCTTAGTGAATACAAAGAAAGAAAAAGA
AGCAATCCTGTCTACACACCACGACGATCCAACACAAGGAGGACACGCAGG
CATTACAAAAACCCTGGCCAGGATTAAAAGACATTACTTTTGGAAAGGTATG
ACTCGGGAAATAACAGAGTACATACGGAAATGTCCAAAATGCCAAAAAGCTA
AAATTATGAAACACACAAAAACTCCTTTATCAATTACAGAGACACCAATAAG
CGCATTTGACAGAGTCATAGTGGATACGATAGGTCCACTACCAAAGTCAGAA
AACGGTAATGAATATGCTGTTACACTTATATGCGACCTGACAAAATATTTGGTA
ACCATTCCAATCGCAAATAAAAGCGCAAATACAGTAGCGAAAGCAATATTTG
AATCCTTTGTACTAAAGTTCGGTCCAATGAAGACGTTCATTTCGGACATGGGA
ACTGAATACAGAAATTCAATTATCAAAGACTTATGCAAATACCTGAAAGTAGA
AAATATAACTTCTTCAGCACACCATCATCAGACGGTAGGAACAGTGGAAAGA
AGTCATAGAACTTTTAACGAATACATCCGGTCATACATATCGGTTGATAAAACT
GACTGGGACGTATGGATAAAATATTTCGAATTTTGTTTAATACTACACCATCT
ATGGCACATAATTATTGTCCATATGAGTTGATTTTCGGTAAAACATGCAATTTA
CCAAAGCATTTTAATAGCACGGATAGAATAGAACCCATTTATAATATAGAAGA

CTATCCTAAAGAAAGTAAATATAGGTTAGAAGTAGCATATAACAGAGCAAGA
ATTATGCTCGAAGAACAGAAGAAAAAGAATAAAGAATTATATGACTTAAAATT
AAACGATATAAGTATATCAATAGGAGATAAGGTGTTATTAAAAAACGAAACCG
GACATAAATTAGATTTCAAATATACTGGACCATATACGGTAGTAAAGATTGAA
GAAAGGGATAATATAGTAATATCAAATGATAAGAAAAAACCACAAACGGTAC
ATAAGGATAGGTTAAAATTGTTTAGTTCTTAAAAAAAAAAAATAAATAATATG
GTGGCCACCAGCAAAAAAAAAAAATATATATAGAGAAAAGAGTTAACCCCAC
ATAAGTGCATTGAATGTAAGAAAACATTTTTCTTTTATCATCTTTGATGGTTGA
ACGATTATAAACTAAAAATTTGAAAAAAAAAAAAAAAAAAAAAAAAAAAAAA
AAAACCAAAAAAAAACCCCCAAAAACACGTATGTTTGTACAAAATGTTCCTT
AAATTTCCTTAACATTAAAATTACTTCCTTAAAAAAAAAAAAAAAAAAAAAA
AAAAAAAAATATATATATATAAAATTTTTAATTATAAAATAACTTCATAAAATTA
CGTTATTTTCCAAAAGGAGGGAGATG

>STALKER4_LTRGypsy#LTR/STALKER4_LTRGypsy

TGTAGCATATTGGACTAATCTACCCTAAGAATACAATAGATGATTGGGTATAAC
ATAGCGTCAATACATTGTGACACTTTGTCATAATAAATATAAATATACAAATATA
CAAAAAGACCACCAAAAACTACGTAAGCACTCCAGCGCCCCAGTAATACGAT
CTAACGCTTATACATAAGCCGATCGCGGAGCGTGGGAATGCTGAGCATGCAC
TTTGCAGCTCAAGTGGTCAATGCCTTCTGCATGCATATGTATATGTATAAATGT
AAGTAAGAATACATAGATATAAGCAATGTATGTGCGGGTTAGCTGAACCCAAC
TTCAGCACACTTTGATCATTCGAATAAACAGATTCAAACAGAGCAGAGGTTC
TGAGCTCGGAAACCAAATCTTTTACATCTATACCTGTTATATTTTTAAAACAAT
TACA

>STALKER4_IGypsy#LTR/STALKER4_IGypsy

TGGCGACCGTGACAGKGTTCACGCGTGCATGAAGTTAACGATTATAATAAAA
GCATGAAGTGTCCGTGGCCAAAAGAAATAAAATGAATAAATTCAAACAAAAT
TCAAATTCGTGGCCAAAGAAATAAAAACAATAAATTCAAACATTACATGGATA
AATGCATTACATACATAGAAATGCAGAAAGGACAAATCTTCTTAAAAAACTTT
TGAACATAAATGGCAGAGGAGCCACAATTCTCGAAGGCAGTACCCACTAAGC
AAAGGGACTTGCCAACTCTGGAAGAGGCCCTACAGGAGAATCCAGCTAATG
GACCACGCCCACTCACGATAAAAGAGTACAGAGCCAGGCAGCAGAAAAAAC
CGCAAAATAAACATAAAAGAAGCGGTCAAAGAATTAAGCTACTTCAGCAAC
GGCGACTGGTCAAGGACATGACCAAGTCGGCAAAAGACGAGGAATCCCGAC
AACGCTACATAGTGCGTCTTCAAGAAATAGAGACTAAGCTTCGCAAAGGTGC
GAAACAACGCAAACGGGCTGCATAAATGCCAATGCCCCAATTTGCCAAAACT
TCAATCAGATGCATCCCGACAACGCTACAAAGAGCGTCTTCAAACACAGGAA
ATAAGTTTTGCAAAGGTGCGAAACAACGCAAACGGGCTGCATAAATGCCAAT
GCCCCAATTTGCTTTAGATTTAATTTCTACCCCAAGCCGATGCGGGAAACCGC
TGCTTGGAAAATACTAAAATCTGTTAGGCTTTTTCACTAACAATGTGGTTGGA
GAATTTTTTTTTTTATTATTGTAAACGACTATGTGAGCCAACCACATATATTAAC
CATTAATATTTCCACGTCTCTGGCACTGAGTATACATATATACTCAGCTGCAAA

ATGTTATTTGTGTAAAATGACAACAATGAAAAAAGTTCTTATTTTGACTAATAT
AAAAGAAAATATTAATTTCATTTTTCCGATTTTCAAGAAGAAAATATTTTTCCT
TCCTTTAAGCTCCAAATAGAATATCTTATTTTTTTTTCCTTTTTTTAAAGATCCG
TTTCATTGAATATAGATAAAATGGGATGGTTTAGCGATTCTAGTGAGGCAAAG
GACAATACTGCCAACGTAGTTAATAACGTAAAAATTATAGATCACACAGACGA
TATAAATGCGTTGTGGATCTTATTGCTGATCATTACAATAGTACTACTTCTACA
ATTTCTGCTTACAATTTATGTTAAGCATAACAAGATCATCAAAAGACGTTATAT
AAATAGGGCAAATCGTTTAGACCAGATTTAAAAAAAAAAATAATATGGATTAGA
AGAAAGCTTAATAAAAACTTTTTTTTTACGACAAGAATGGAATGGAGCGAAA
TAGCGATACGAATAGACGAATTTCGCTTCAGGTTCGATAAGTCTTATAAATGT
ATCAATAGAGACGCAGTAATAAAATCCGAAACTTTGAAAAATCATATAGAGAC
ATTAGTAGGAGAATATAATAATATAGTTACATTAGTAAATAAATATGCAAATAG
GCTCACATCTGAACATAATAACAAATGTTTGAGGGTTATAAAATCCCTAAACA
CAAGATTAAATAACATCAGAAAAAGAAGGCATATTCTGATAGATGTACCAGA
AAGTCTAAGTCAATTGGTTGAATTCAACACAGACCAGTTCAAAGAACTAGAC
GAATCTGTTCAATCAAGCGGCGCTGAGTCCGATAGTGACATTGAAACGCTAG
AAGGAAGCGACCGAATTGAATTTAAATCTGAACCAATAAAAATTTCTGAGAT
GGCACAGACATTGATAGAATTTATCAGGCTAGCCACATCTCTGATACCAGAGT
TTGATGGTAAACCAGAAAATCTACAAAGTTTTTTGGATGCTCTAGGTCTACTA
GATAGCTTAAAGAGCACACATGAAACGACAGCAGTAAGCCTAATAAAAACTA
AACTTAAAGGCCATGTAAGAAACCTTATAAGTAATGAGCAGACGATTGCTGC
AATCATTACCCAACTGTCAAGTGCAGTAAAAGGAGAATCGGTAGAAGTGATA
TCTGCCAAGCTTCTGAATCTACAACAGAGAAATAAAACGGCTAACCAATACA
CCCAAGAGGTGGAGAAACTGACAAAGGCCCTTGAAGGTGCCTATATCAGTG
AAGGTCTCAGCCAGTCCTTAGCAAATAAATACAGCACTACAACAGCTGTAAA
AGCAATGACACAGAATTGCTCCATTGATAAGGTAAAACTTATCATGCAAGCA
GGCACATTCACAAACATGAATGATGCCATCTCCAAATTTGTAAACAGTTGCAC
AGAGATAACAGGTCAAAGTAACACTGTACTCTATTATCGACGAGGTGCAAAT
AATTATAATAGAGGCGCCCGGGGTTATAATCGTGGTAGAAATATCAACCACAA
CAATTACAACCGAGGTAGCAATAACAACAATAATAATAACTATAATAACCGTG
GAGGTAGGCGAGGCCAAAACCAAGGGAGAGGCCGCGGAAACTACAACCAT
GGTAATAATAATAATAGCAGTGTGAGAATCGCGCAAAATACGTCGGAAAACT
AACAGAACCCTTTAGGAAACAACCAATAAATGTAAAAGTTCATTCCATCAATT
ATAGTCTTAATATATTCGTAACCTTCTATAATCATTCCACTGAAAATAAACTAAC
ATTTCTCATAGATACTGGTGCAGATATCTCACTTTTGAAAGTAAATTCTGATAA
CTTCGTAATTCAAAATGAAAAAATAATAAACATCGAAGGCATAGGCCAAGGT
GTGATAAAGTCTCAAGGAACAACCTTAATAGAACTCCAATCAACAAAATATAT
TATCCCACATGAATTTCATTTGGTAAACCCAAATTTTGCAATACCATGTGATGG
AATAATAGGCATTGATTTTATAAAGAAATTCAATTGTCAACTAGATTTCAAACC
AAGTGAAGACTGGTTTATAATTAGACCCCAAAATTTAAATTATCCAATATATGT
CCCGATAACATATAGCGCTGGCAACAATACAGTTCTTCTGCCAGCCAGATCAC
AAGTTATTCGGAAAATAGACATTAATGTTGTAAATGATTTCATATTTGTTCCTA
ATCAAGAAATACACAATGGGATTTATGTTGCAAATACAATAGCAGCATCCAAA

CATGTATACGTTCGACTTCTAAATACAACTAATTTCGACCAAGTGGTCAAAGT
AAATAAAATACAATATGAAAATCTAAAAGATTATGACATTCATAATACCGACA
CTGGAAATAGAAGCGAACAAATACTTTCAAAACTAAAGAAAAATTTTCCAGA
CCAATTTAAAAATCAATTAACAGAATTATGCACACAGTATAGTGATGTGTTCG
GACTGGAAACCGAACCTATATCAACAAATAATTTTTATAAACAAACATTAAGA
CTTAAAGATGATGAACCCATTTATATAAAAAACTATAGAAGCCCGCATAGCCA
TATTGAGGAAATTCAAAAACAAGTAGGGAAATTAATAAGCGACAAAATCGTC
GAACCGTCTGTATCTGAGTATAACAGCCCACTCTTGCTAGTTCCAAAAAAATC
ATTACCAAATTCACAAGAGAAAAAATGGCGATTAGTAATTGACTATCGTCAAA
TAAACAAAAAACTTCTTTCTGACAAATTTCCACTCCCTAGAATTGATGACATT
TTAGATCAACTAGGTCGAGCTAAATACTTTTCATGCCTTGACTTGATGTCAGG
TTTCCATCAAATAGAACTTGAGGAAAACTCTAGGAATATAACATCTTTTTCAA
CGAGCAATGGCTCATATCGCTTCACGCGATTACCATTTGGTCTTAAAATAGCA
CCAAATTCATTTCAGAGGATGATGACTATATCATTCTCGGGATTAGAACCCTCT
CAGGCATTCCTTTACATGGATGACTTAATGGTGATAGGATGTTCCGAAAAACA
CATGATTAAAAACTTAACTGACGTTTTTAATGTATGTAGGAAATATAACCTAAA
GTTGCATCCAGAAAAATGTTCATTTTTCATGCACGAAGTGACATTCCTAGGTC
ACAAATGCACAGACAAAGGAGTATTGCCAGATGACAAGAAATATGACGTCAT
CAAAAATTATCCTGTCCCTCATGATGCGGACAGTGCAAGACGATTTGTAGCAT
TCTGCAACTATTATCGTCGATTTATAAGGAACTTCGCCGACTATTCACGGCAC
ATAACTAGATTATGTAAAAAGAATGTCCCTTTTGAATGGTCAAGCGAATGCCA
GAACGCATTCGAATACCTAAAAGAAAATCTTATGCACCCCACACTATTACAAT
ATCCTGATTTTCGCAAAGAATTTTGCATTATAACGGATGCTAGTAAACAAGCT
TGCGGAGCGGTTCTAACTCAGAACCGAAACGGGATTCAGCTCCCAATAGCTT
ATGCATCACGTTCATTTACAAAAGGAGAAAGCAATAAGAGTACAACGGAACA
AGAACTAGCGGCAATCCATTGGGCAATTACCCATTTTAGACCATACATTTATG
GCAAGCATTTCACCATTAAAACGGACCACAGACCATTAACGTACCTATTTTCT
ATGACTAATCCCAGTTCTAAATTAACTCGCATGCGGCTAGAACTAGAAGAATA
CGACTTCACAGTAGAATACCTAAGGGGGAAAGATAATTTTGTAGCAGACGCA
CTCTCACGTATAAATATAAAGGAACTCAAAGACATGCAACATAAAGTCCTGA
AAGTCACTACCAGGCAACAAAGTAGACAAGAAAACTGTACAGTAACAAACA
AGGAACTATTGCCTAGGCAAAGTATCCAAAATGTATCTAAGCCCAACGTACAC
GAAGTCATAACAAATGATGAAGTACGAAAAGTAGTGACCTTGCGAATAACTG
AATCTATTTGTTTACTAAAACGAGGAAATAAAGTTATTGCAAGAATTGATGTT
GACGATTTATATACCAATGGAATTTTTGATTTAGGTCAGTTCTTCCAAAGGCTT
GAAATGCAAGCCGGTATACTAAAAATCAGCCAACTCAAATTGGCACCGAGTG
AAAAAATCTTTGAAACCATTTCAATAGATAATTTCAAAAATATGGGCAATATA
AAATTGAAAACATTAAGAGTAGCGCTACTCCAGCCGGTGACCATTATAAAAA
CTGAAAAAGAGATACAATCGATACTGTCTACATATCATGACGATCCAATTCAA
GGAGGTCATACAGGCATTACAAGAACGCTAGCGAAAATAAAAAGACACTATT
ATTGGAAAAATATGACTCGTCATATAAAAGAGTACATACGTAGATGTCATAAA
TGCCAAATGTCAAAAACAACGACACATACAAAGACCCCATTGACTTACACAG
AAACCCCAACAAATGCTTTTGATATAGTGATAGTGGACACAGTTGGTCCACTA

CCGAAATCAGAATATGGCAACGAATACATCGTCACACTAATATGTGATTTGAC
GAAGTATCTAGTAACCATACCTGTTGCGAATAAGAGCGCAAATACTGTCGCAA
AAGCTATATTCGAAAATTTTATACTAAAGTACGGTCCAATGAAGACGTTCATTT
CGGACATGGGTACCGAGTATAAAAACAATGTAATTCAAGATATGTGTAAATAT
ATGAAAATTGAAAATCTTACATCCACTGCATATCACCACCAGACTTTAGGGAC
AATCGAACGAAGTCATAGAACATTCAATGAATACATTCGTTCATACATCTCTG
CAGATAAAACTGATTGGGACGTTTGGATACAATACTTTACATATTGTTTCAAC
ACAACACCATCAGTCATGCATAATTACTGTCCATATGAACTAGTCTTTGGAAG
ATTACCAAGGCAGTTCGCAAATTTTAATAAAACAGATAGAATAGAACCACTGT
ATAATATAGAAGATTACTCAAAGGAAATAAAATTTAGATTAGAAATAGCATATA
AAAGAGCTAGACTTTTGTTAGAAAAAGCTAAGTCTTATAGAAAACAACTTTA
TGATAAGAAAACTTCAGATTTTCAATTAAAAATAGGAGATAAAGTTATACTAA
GGAACGAATCGGGTCATAAGTTAGATCCAGTATATATAGGCCCTTATACTGTAG
AAACCATAGAAGACAGAGATAACATAGTAATTAGAGATACAAAACAAAAGAA
GCAAAAAGTACATAAGGATAGACTAAAAATATATAATCAATGAAACGTTTCAT
TTCACTTAAGAAAAGGTCTGATCAACCTCAAAACAAAAAAAAAAAAAAACA
CAAAAAAAATTTAATTATTATTTTTCCTTCTAAGAAAGTTAAACATAAATCCAA
AAACATCGTAATTCAACATACATTTTTTGTATTATTCTGTCATTATACAAAAATG
CTTTGAGACAAAACATTGCTAATAATTAATAAGAAAAATCAATTTCAAAAAAA
AATTTTTCCTTTCTAAACACAATATTAATATTGAGAACTCAATGACTACATATAT
TACGTCATTTCTTTAAAAAGGGAGG

>STALKER3_LTRGypsy#LTR/STALKER3_LTRGypsy

TGTAGTGTATCTACCCTCAATATGTARAGTAGAGTTAATATGTAAGTAAGTAAT
ATGTAAAGTAGAGTTAATATGTAAGTAAGCAAAAGACCACCAACACTTACAT
GAACACTCCAGCTCTTGAAATACGATCGAGCGCTTAAACATAAGCCGATCGC
GGAGCGTGAGAGTGCCGAGCATACACCTAGCAGCTCAAGTGATTAAGATAAG
ATAAGATAAGATAACAAACACGTAGTCTTAAGCGCGTCATGTGCGGGTGGCT
GTACCCAAGAACAGCAAAGTGAATTCATTCGAATAAACCGCTTCAAGCAGAG
CAGAGCCAAGTCTATTATATCAACTTCAAAAATACCGTATAACCTTGAACCTAT
TACA

>STALKER2_LTRGypsy#LTR/STALKER2_LTRGypsy

TGTAGTGTATCTACCCTCACTATAACTCTACTCTACATATATATAAGTAACGTAC
ATACATTGTGACACTTTGTTGCAAACACAAATAAACATAATTCACATCAAAGA
CCACATGCACTTACATAAACACTCCAGCCAATGAAATACGATCTAACGCTTAT
ACATAAGCCGATCGCGGAGCGTGAGAATGCTGAGCATGCACTTAGCAGCTCA
AGTGGTCAAGCCATACATAACATATGTATGCCTTCTGCATACACATGTATATGT
ATATACAATATGTACAATATGTAAGAACACCATGTACGGGTAGCTGTACCCAA
AGACAGCAACATAGGATTCATTCAAATAAAACGATTCAAACGGAACAGACGC
TCTGAGCTATTCAATATCTATTACACTGAGCTATTACTTATTACTTATTACA

>STALKER2_IGypsy#LTR/STALKER2_IGypsy

TGGCGACCGTGACTTGGTCTCGAGTCTGCCTCTGTGTTGTGCCTCTGTGTATT
GTGATTATGTATCGTGTATTGTGTTTGTGTTAAAAAAAAAAAAAAAAAACAAC
ATTTTGTGCCTATTATATTCAACGTGTGAACACACAAACATAACTAAAATGTAC
TTGTAGTACTTGGACGCATTAAATGCAAATATATGTTCAATTGAAACAAACAA
AGCACAAAAACACACAAAAACACACACAAAAACGCACAAAAACACGCAAA
CACAGCACACACAGAAGAACAAGCGGCGATTGATGGCAGACGAGCCACAAT
TCGCCAATGCACAACCACAAGTGCAACGGGACCAGCCAACGCTAGAGGAAG
CGTTGCGGTTAAACAACGCCGATGGACCACGCCCACTCACAGTAGCTGAGTA
CCGGGCACGGCAGGAGAAGAAACAACTACGAAAGCACAAACGCTCAGGAC
GGAGGATTAAACTACTACAACAACGCCGACTGGTCAAGGAAATGACCCAGTT
GGCAAAAGAAGAATCAGCACGACAACGCTACCAAGAGCGTTTGGAAGCCAT
TGAACAAGAACTTCGCCAAAGTGCGAAGACACGCAAACGGGCTGCTTAAAT
GCAAATGCCCCAATTTGCCCTAGAATTAAAATAAACCCAAGCCGAGGCGGGA
AACCGCTGCTTGAAAATACTAACAAAACTTTATGCTAGGCTTTATACTAAAAA
ATGTGGTTGGAGATTTTTTTTTAAAATCCTATTGAATTGTAAATTGTGACTTG
TGAGCCAACCACATTAGCACTATCAAATTTCCATGTCTCTGCCACTGAGTATAT
ATACTCAGTTGCAAATAATTTGTGTAACTTTAACAACTTTAAATTTTCTATTTC
AAAAATAAAATTTTACATTTTAAATTTAAATGAATAACATTTTAAAAATGGATA
AAAAAAAAAAAAAAAAATAACTTTCTTAAAGAGAATTTTCTTTCTAAAAATAC
AAGAAAAAATAGATTTTTATTTTTCATATTATATATATAATAACATTATATATAAT
ACTTCAAAAAAAAAACGTTTCATTGAATATATAAAAATATTCAACACAAATTA
AAATAAATTTTCTCTTCAATTTAAAATTACACAAATCAAACAAAAATTAGAAA
TAAAATTAGGAAACCTAATAAGTAAAACAATTATATTACATGTCATTAAAATTG
AATGAGCATTAAAGATAATTCCATACCGAAGATAGACCCCCTAAAGACAATCA
TTTCGGAACTGTCTACTATGTTAGACAAAAATTCCAATATAGCGGACTCCACC
GCAAACGTAATCAATACAGTTCAGGTATCACAGCCCGAACTTAAAATAATTAC
GGTACTCCTCATAATAATAGTAGTACTGCTATGTGCGAGTATGATAACGAAATT
GTACAAACTACATAACAGATGCCTCAAGAAGAAATACTTGAGTAAGGCATTG
GACCTAGATAAGGTCTAAAGTAGTACCCTGCGTACAAATTTCGAATATTCAAT
TAAATAGAAATTATCTTTACATATATATATAAAAAAAAAAAAAAAAGAATATCAA
ACATACTATAAATGAAATATTAAGTAGAGACCCCCACTTCGCAAGTAGACTAT
ATTTTATACTTGCCGAGCAACCCGTAAGCCTTTACGAGTTGCAAATATTATACA
TTAACACCTACAACTGCGAGTTCTTTCCAATGTTCGGGTTGGAAAGAGAACA
AGAAATCATGGTATTAAATTCAATACCAAATGTTATCGCAGAAGTTGTAGAAA
TATCAAACCCAATACTAGGGTCAACAGAAATAATAACAGTTCGTAAGAATTAG
AGCATGGAATGGCATGAAATATGCACAACAATTAAAAATATTAAAACTAAATT
CGATAAGACATATAAATGCTTATCGCCCAATAGGCCTATACAGGCTGAAACAA
TTAAAAAACATGCATCTACATTAGTCGACTGCTTTAATGAAGCACGAACACTA
ATATACGAACATAGAGAAACACTTAATTCTGAACACTGGTCTAAGTTATCAAG
ACTATTAATCAAACTTCGATTAAACTTGATAGCAGTTAAAAGGAAATTTAGTT
TGGAAATTTCAATTCCAACAATACTAAATACCCCTTTAACGATTGATACAGAC
GAACAATCTGAATCAATAGAACCAGAAGAAATCGATTTAGAGGTCACCGAGT
CGGAAGAAAAATATACCGACATCGAGGATAAAGACCTACTAAACCTAACAAT

TCCAGCTATATTAACATTAGCTGAAGACACTAATCAGGAAAAATTTAAAACAG
AAAACATAATGGCACAGTCCAATATTGATTTTCTGAATACAGCATCAAAGCTT
ATACCCGACTTTGACGGTAAGGCTGAAAACTTAACAAGTTTTATAGATGCTCT
AAACATTGTAGATACAATCAAAGGTGAGCATGAGTCTCTAGCTGTTTCGGTTA
TAAAAACCAAACTTAAAGGCCATGCAAGAAACCTCATAAGTAATGAGCAGAC
AATTGCTGCAATCATTACCCAACTGTCAAGTGCAGTTAAAGGAGAATCGGTA
GAAGTTATATCAGCTAAGCTTCTCAGTCTCCAACAAAAGAGTAAAACGGCCA
ACCAATACACCCAAGAGGTGGAGAAACTGACAAAGGCTCTTGAAGGCGCCT
ATATCAGTGAAGGTTTAGGCCAAACTCTTGCCAATAAATACAGCACTACCACA
GCTGTAAAGGCCATGACGAAAAATTGCTCCATTGATAAGGTAAAACTTATCAT
GCAAGCAGGCACATTCACAAACATGAATGATGCCATATCTAAATTTGTAAACA
GCTGCACGGAGATAACAGGTCAGAGTAACACTGTACTCTATTATCGACGAGG
TGCAAATAACAATAATAGAGGGGGCCGAGGTTATAATCGCGGCAGAAATGGC
AACAATTACAACCGAGGCAACAATAATAGTAATAACTATAACAACCGTGGAG
GAAGACGAGGCCGAAACCAAGGTAGAGGCCGCGGCAACTCCAACCAAGGT
TACAATACCAACAATGTGAGAGTGACGCAAAACACGTCGGAAAACTCACAG
ACCCCTTTAGGAAACAATCAATAAATGCTAGAGTTCATTCCATCAATTATAGTC
TTAATATATTCGTAACTTTTTACAATAATTCAACTGATAACAAGTTAACATTTCT
CATTGATACTGGTGCAGATATCTCACTTTTAAAAGTTAATTCAGATAACTTCAA
AATTCAGGATGACAAAATAATAAACATCCAAGGCATAGGCCAAGGTGTAATA
AAGTCTCAAGGAACAACCCTAATAGATCTTCAATCAACAAAATATATTATTCC
ACATGAATTTCATTTGGTAAACTCAAATTTTTCAATACCATGTGATGGAATAAT
AGGCATTGACTTTATAAAAAAATTCAATTGCCAACTTGACTTCAAACCAAGT
GAAGACTGGTTTATAATTAGACCCCAAAATTTAAATTATCCAATATATGTCCCA
ATAACATATAGCGCTGGCAACAATACAGTTCTTCTGCCAGCCAGATCACAAGT
TATTCGGAAAATAGACATCAATAGCGTAAATGATCAAATATTCGTTCCTAATCA
GGAAATACATAATGGAATTTATGTTGCGAATACAATAGCAGCATCAAAAAATG
TATATATTCGACTTCTAAATACCACTAATTTCGACCAAATGGTCAAAGTAAACA
AAATCAAATATGAAAATCTTAAAGATTATGACATTCATAATACCAATCTAGAAG
ATAGAAGCGAAAAAGTACTATCATTACTGAAGAAGAATTTTCCAGAACAATT
TAAAAGTCAATTAACTGAATTATGCACAAAGTATAGTGATGTGTTCGGACTGG
AAACCGAACCAATATCAACAAATAATTTTTATAAACAAACATTAAGACTTAAA
GATGATGAACCCATTTATATTAAGAACTACAGAAGCCCGCATAGCCATATCGA
AGAAATTCAAAAACAAGTAGGGAAATTAATTAACGACAAAATCGTAGAACCA
TCTGTATCTGAATATAACAGCCCACTCTTGCTAGTTCCGAAAAAATCATTACC
GAATTCAGAACAAAAGAAATGGCGATTAGTAATTGACTATCGTCAAATTAATA
AGAAACTACTTTCTGATAAATTCCCACTCCCTAGAATTGATGACATTTTAGATC
AACTAGGTCGAGCTAAATACTTTTCATGCCTTGACTTGATGTCAGGTTTCCAT
CAAATAGAACTTGAAGAAAACTCAAGAAATATAACATCTTTTTCAACAAGCA
ATGGCTCATATCGCTTCACGCGATTACCATTTGGTCTCAAAATAGCACCAAATT
CATTTCAGAGAATGATGACTATATCATTCTCTGGTTTAGAACCTTCTCAGGCAT
TCCTTTATATGGATGACTTAATGGTGATAGGATGTTCCGAAAAACACATGATTA
AAAACTTAACTGATGTTTTTAATATATGTAGGAAATATAACCTAAAGTTGCATC

CGGAAAAATGTTCATTTTTCATGCATGAAGTGACATTCCTAGGTCACAAATGC
ACAGACAAAGGAGTTTTGCCAGATGACAAAAAATATGACGTCATCAAAAATT
ATCCTGTCCCTCACGATGCGGACAGCGCAAGACGATTTGTAGCATTCTGCAA
CTATTATCGTCGATTTATAAAGAACTTCGCCGACTATTCACGGCACATAACTAG
ATTATGTAAAAAGAATGTTCCTTTTGAATGGTCAAGCGAATGCCAAAACGCAT
TCGAATACCTTAAAGAAAAGCTTATGCACCCCACATTATTACAATATCCTGATT
TTCGCAAAGAATTTTGCATCATAACGGATGCTAGTAAACAAGCTTGTGGAGC
GGTTTTAACCCAGAACCGAGACGGAATTCAGCTCCCAATATCTTATGCATCAC
GTTCGTTTACAAAAGGGGAAAGCAATAAGAGTACAACGGAACAAGAGTTAG
CGGCAATTCATTGGGCAATTACCCATTTTAGACCATACATTTATGGCAAACATT
TCACAATCAAAACAGATCACAGACCTTTAACATATCTATTTTCTATGACCAATC
CCAATTCAAAATTAACTCGCATGCGACTAGAGCTTGAAGAATACGACTTCACT
GTAGAATACCTAAAGGGAAAAGATAATTTTGTGGCAGATGCACTGTCACGTAT
CACTATAAACGAGCTGAAAGACATATCAGCAAATGTACTAAAAGTCACTACA
AGACAGCAAAGTAAACAGAAAAATATCTGCGCAGATACTAATATAAATAAAC
AAGAAGAAACTCTTGTTAACGCTTCTAAGCCCAACGTATATGAAGTCATTAAT
AATGACGAAATACGAAAAGTAGTGACTCTGCGAATAACTGAATCAAAATGTT
TATTCAAACATGGAAATAAAGTTATAGCAAGAATTGATGTTAGCGATCTGTAC
ACCAATGGAATTCTAGACTTAGGTCAGTTCTTCCAAAGGCTTGAAACACAAG
CCGGTATACATGAGATCAGCCAACTCAAAGTGGCACCGAGCGAAAAGATCTT
TGAAACAATTTCAATAGACTCTTTTAAAAAAATGGGCAATAAATTATTGAAAA
TATTGAGAGTAGCGCTACTCAAGCCGGTGACCCAAATATATGATCAAAAAGAT
CAAGAAGCGATACTGTTTACATACCATGACGATCCAATTCAAGGAGGTCATAC
AGGCATTACAAGAACGCTAGCAAAAATTAAAAGACATTATTATTGGAAAAAT
ATGACACGTCATATCAAAGAGTACGTTAATAAATGTCAAAAATGCCTTACGTC
TAAAACAACGACGCATACAAAAACACCCCTTACAATAACGGAAACACCAAC
TTGCGCTTTCGACAGAGTGATAGTAGACACTATAGGTCCACTACCCAAATCGG
AAAATGGTAATGAATATGCAGTCACTCTAATCTGTGACTTAACAAAATATTTA
GTTGCCATACCAATTCCCAACAAAAATGCAAATACAGTCGCAAAAGCTATATT
TGAATCATTCATTCTGAAGTACGGTCCAATGAAGACGTTCATTACGGACATGG
GAACAGAATACAAGAATAGCATTATAGCCGACTTATGCAAATATCTGAGAATA
GACAATATAACGTCTACAGCACACCACCACCAGACATTAGGTACCGTTGAAA
GAAGTCATCGAACCTTCAATGAGTACATTCGTTCATATATATCAGTAGATAAAA
CTGACTGGGACATATGGTTACAATATTTTGTGTACTGTTTTAACACTACACCAT
CCGTAACGCATAATTACTGTCCATATGAACTAGTATTTGGTAAGACTAGTAATT
TAGCAAAACAATTTAGTAACATAGATAATATAGACCCTATATATAACATAGATG
ATTACGCTAAAGAAGTTAAATTTAGACTAGAAAACGCGTATAAAAGAGCACG
CATATTATTAGAAAGWAATAAAGTAAAACAGAAAACCAGTTATGATAACAAA
GTTAGCGATTTTAAACTAAAAGTAGGAGATAACGTTTTAATAAGAAACGAAA
CAGGTCACAAACTAGAACCAACGTATCTAGGGCCATTCGAAGTAATTAGAAT
CGAAGAAAACAATAACATAGTAATTAGGAATAAAAAGAACAAAGAACAGAA
AGTTCATAAGGATAGGTTAAAAATATATAATTAATGAAACGTTTTATACAAAAT
AAATAACTAAAAAAAAAAAAAAAAAAAAAGGTCTGATCAACCGAAAAAAAAA

AAAAATTTAATAAGTTTTTGTCTAAGAAAGTTAAAAATAGAAGCATAACGTAA
TTATAAACAACAAAAAAAAAAAAAAATATAAAAAAAAAAATTCTTTTACACAA
ATTATTCTGAGACCAAACTTTTTCTAATAGAAAGGATAAATAAGAAATAATATA
AGAAAAAAAATGTTTTAATTAATTAAATGACTACATATATTACGTCATTTCTCT
AAAAAGGGAGG

>S_Mariner-Tc1#DNA/S_Mariner-Tc1

CAGTTTGTCAAGAAACTGTTTACACACCGCAAAATAAGTAGAATTTTTGACTT
TAAAGGCCAAAATTAAGGGTTTTTTGCTTAATTAAACGCAATTTTTTTATGAA
ATATAATTAAACAATATTTATTTTACTTATAAATTAAAAAACAAATTCAATATAT
CAAATATACAAGAAAATAAACAACAAATTTCTTGTTTACACACTTTTGAGAGT
GCCAAGAAACTCTTTACACAGTTTTGGGTTCCTACTTTGTTTTGCTCTTTTTCT
TAGAAACAATCTCATTTTTCCGTTATTTTTGTCTTATGCATTCCTTTTTACAACG
CTTCTATTGCAATTTTTTCACTTTGCTTGTGAAATTTTGTTGATCTAACGTGCT
TAAAGCGAATTATTAAATTTAATGAAATGCCTGGAAAGAGATTGGCTTTTGAA
GTGACCCAGCTAATATACTATAACCACCAGTTGGGAAAATCTATTCCTGAATT
AGTAGAAATATTTTCCGTATCCCGTAAAACCGTCTATAATATTTTAAATCGTNN
NNAAAAAGAGGGCAGGCTTGAACCTAAGAGTGGTGGTGGGTGTAAAACGA
AAATTAACAAGCGAGTAGACCGCCTTATTATGCGAAAAGCGATTGCGAACCC
CCGAATCTCGGTCAGATCACTTGCTCAGGATATCAGGGAAGAATGTCACCTA
ACTGTATCACACGAAACTGTGCGCCAAGTCATCCTACGCCATAGGTACTCTTC
AAGAGTTGCAAGAAAAAAGCCTTTGCTATCAGAGATCAATATTGAAAAGCGT
CATTCATTCGCTGTGAGCATGATGGATCATGCGGAAGAGTACTGGGATGACGT
CATATTTTGTGACGAAACAAAAATGATGCTCTTTTATAACGATGGGCCAAGCA
GAGTATGGCGCAAACCGTTGAGTGCGCTAGAAACACAAAATATAATTCCAAC
AATCAAATTTGGAAAATTGTCAGTGATGATTTGGGGCTGTATTTCCAGCCATG
GAGTGGGCAAACTAGCCTTTATTGAAAGCACTATGAATGCCGTGCAATATCTA
GATATTTTAAAAACAAATTTGAAGGCCAGTGCAGAAAAATTTGGTTTGTTTAG
CAACAACAAGCCAAATTTTAAGTTTTATCAGGACAATGATCCCAAACATAAA
GAGTACAATGTACGCAACTGGCTACTCTATAACTGTGGCAAGGTGATCGATAC
GCCCCCTCAGAGTCCTGATCTAAACCCCATTGAAAATTTGTGGGCCTACTTAA
AGAAGAAGGTTGCAAAAAGGGGCCCCAAAACTCGACAACAACTCATGGCTG
CGATAATCGAAGAGTGGGAAAAGATCCCGCTTGAATATGACCTAAAAAAACT
CATACATTCCATGAAAAAAAGGCTTCAACTTGTAGCCAAAGCCAATGGGGGT
CATACTAAATACTAAAACTTTTCAAATATTATCAAAATAATTAAAAAATTTAGG
ATTAAACTTAGGTTTAGTGTTTTGTGTAAAGAGTTTCTTGACACTCTCAAAAG
TGTGTAAACTTGAAATTTGTTGTTTATTTTCTTGTATATTTGATATATTGAATTT
GTTTTTTAATTTATAAGTAAAATAAATATTGTTTAATTATATTTCATAAAAAAAT
TGCGTTTAATTAAGCGAAAAACCCTTAATTTTGACCTTTAAAGTCAAAAATTC
TACTTATTTTACGGTGTGTAAACAGTTTCTTGACAAACTG

>S2_Mariner-Tc1#DNA/S2_Mariner-Tc1

CAGTTTGTCAAGAAATTGTTTGCACAATGAAAATAAATTGTATTTTCTGCCTT
TCAATGCAAAATTAAGGGCTTTTTGTTTACCTTATGGATTTTTTTTAATTTACAT
TTATTTGTTTATATGTTTTTTATCAATATGTAAAAAAATAATAACAAAACTATTA
ACAATGAACGAGAAAAACAACTAAATCCAAGTTTGCACAGATTTGACACTGT
CAAGAAATTGTTTGCACACTTTTCTTTTGTTTTCTATTTTGCTCCTATTCCGAG
GAATTTCGATAATCAAACGGTACATAATCGTCATTTACGCTTTATGCGTTGTGT
GGGTGGCCGATTTATATGCATTTGGTTGTGAAATTTGCCAAAGTGATCATTGTT
CCAGTTAGTTTTGTGAAATTTAGAAATGCCTGGCAAAAGATTGAGTTTTGAA
GTAGTTCAACTGATTTTTTATAATCACCATTTGGGAAAATCTGTCAAGGAATT
GTCAGAAATGTTTTCTGCATCGCGGAGAACAATTTATAACGTTATTAATCGCG
CAAAAAACGAGGGCAGATTGGATTCAAAATGTGGAGCAGGACGTAAAAATA
AAATTTCCAACCGTTCAGACCGACTTATTATGCGAAAAATTTATGAAAATCCG
CAAATATCCTTAAGAACTGTTGCTAAGGAGCTTAAGGAGGAGTGCGATCTGG
ATGTGTCACACGAAACAGTGCGCCAAGCCATACTCCGGCATAAGTACCACTC
ACGGGTAGCAAGAAAGAAACCGATGCTGTCGGCAATTAACATCGAAAAACG
GCTTACTTTCGCTATCAAAATGCAACATCAGCCAGAAGATTACTGGAATAACG
TAATTTTCTGCGACGAAACCAAGATGATGTTGTATTACAACGATGGACCCAAC
AGAGTGTGGCGTAAGGCGCTAACTGCGCTGGAGAACCGCAACATCATACCG
ACAGTCAAATTTGGCAAACTGTCGGTTATGATTTGGGGATGCATATCTAGTCG
AGGAGTTGGAGATTTGGCATTCATAGAGAACACAATGAATGCAGAACAATTT
TTAAACATTTTGAAATGCAATCTGACATCCAGCGCCAAAAAATTCGGTTTTTA
TAAGAACAATAGACCTGATTTTAAATTTTACCAGGACAATGACCCCAAGCATA
AGGCGCTTAATGTGCGGAACTGGCTCGTTTACGACTGCGGCAAGGTCATCGA
TACGCATCCTCAGAATCCAGATCTGAACCCTATAGAAAATTTGTGGGTTCATT
TGAAGAAAAAAGTGGCAAAAAGGTCACCAAACAACCGAGCAGCACTGAAA
GCTGCTATACTAGAAGAGTGGCATAAGATCCCACAACAATATGATCTTGAAAA
GTTATTTTTTCAATGAAAAAAACGTCTACAATATGTTATTAATGCTAAGGGTAT
GCATACAAAGTATTAGAATTACCTCAAGTTATTATAATTTGTTATAAACTATAAG
TCTAAGATAGCTTAAGTACTTTGTGCAAACAATTTCTTGACAGTGTCAAATCT
GTGCAAACTTGGATTTAGTTGTTTTTCTCGTTCATTGTTAATAGTTTTGTTATTA
TTTTTTTACATATTGATAAAAAAACATATGAATAAATAGATATTAATAAAAAAA
ATTCATGAATTTTAATAAAAGGCCTTTATATTTTTGCATTGAAAGTCAAAAATA
CAATTTATTTTCATTGTGCAAACAATTTCTTGACAAACTG

>ROVER_Gypsy#LTR/ROVER_Gypsy

AGTAACATAATATGCTTCTCATATTACGTTTACATACTTACACTAATTGTACATA
CAATCTTGCACATGCATAAACACATGAAACCAGTTTACATTTTTACTTACACTT
AAGCGCATAATTTGTTGTGCATCCATACCGTTATTTTTCCGTTCTTTTTTGTAC
ACATATACTGATTAGACATTCCCGTTTCTCGCGACTCACTTCGAGCCGATCAA
AAACTCTGTACAGTCAGTCTTAAGCCGACAACGAAGAAATAAAGATCTAAAC
TAAAAAATACCTCGTGTTGATTCTTTCAAATCCGAAACTTCTTTAAAGGCGTT
GATCTTAGTCAAACGACGGATCATTTGTTCGACTCGAATAGTAAAATACGTAA
GTGGCGCAGTCGGTAGGATAATACATTGTTGATGCGATAGTAACCCTATTCAA

TTCTTCAATTATTATCCACAAAAGAACTAACGCAGTCGCGTCGTGCTAAGTGG
AAAGTGTTAACCAATGGTACTCAATCCCATAAAGTGACCACGAAGTGAAATA
ACTTAAAATTACAAAAAATTTTAAGAAGACGCCTTTGAATTCGCTGAGTAGTA
AATCCACAAAAGGTTACTCAAACCAAAGCGAAACAGCTAAGAGCGCAACAC
AACAAACTTAAATTAAAAACTTACAAATAAGTGAATAACAGAAATATTACGA
ACAAATAAGTGCGAAAATTCAACAAAACCTAAGCTAAAGAACTATAACTAAG
TGAATAACAAACATTATAAGTGAATTAAATTAATAAGTGAACAACACAACAA
ACTTAAATTAAAAACTTACAAATAAGTGAATAACAGAAATATTACGAACAAAT
AAGTGCGAAAATTCAACAAAACCTAAGCTAAAGAACTATAACTAAGTGAATA
ACAAACATTATAAGTGAATTAAATTAATAAGTGAACAACACAACAAACTTAA
ATTAAAAACTTACAAATAAGTGAATAACAGAAATATTACGAACAAATAAGTGC
GAAAATTCAACAAAACCTAAGCTAAAGAACTATAACTAAGTGAATAACAAAC
ATTATAAGTGAATTAAATTAATAAGTGAACAACACAACAAACTTAAATTAAAA
ACTTACAAATAAGTGAATAACAGAAATATTACGAACAAATAAGTGCGAAAAT
TCAACAAAACCTAAGCTAAAGAACTATAACTAAGTGAATAACAAACATTATA
AGTGAATTAAATTAATAAATGAACAAACACAAACACAAAGCTCAGACCTGGA
ATACAAACTAATAGTGGAAATCAAGAACCCATAGTGAAAACCAAAACCCTAT
CCAAAACATAAACCTACAGTAAAAACTTGAACTTTATCGAAAAACGAAACCT
ATCCGAAACTGCAAACCTTACCCAGAACGTGAACCTTACCTAAAACATTAAA
TAACAATAAATACAACAACAACAATGGCAACAGCAAGCCCTATAATACTATCC
GACTCAAATATGAGTCAGGTCGAGAGACAGATAAATGCTGTCGAAATCTTCA
ATGGAGACCCGAATACTCTACATACCTTTATAACTCGCATCGACTTCATTCTGG
CCTTATACCAAACTACTGACGAAAGGCAGAAGTTGATAATTTATGGACACATC
GAACGCAATATCAGCGGCGACGTCATCCGTACTCTCGGGACCAACAACTTTA
CCAGCTGGATTGAACTAAGGACCAGATTGATCCTATATTACAAACCCCAGGCA
CCGAGTCATCAACTTTTGGAGGATTTTCGGAACATTCAATACAAAGGCAACA
TCAGGCAGTTTCTGGAGGAAGCCGAGAAAAGACGACAAGTTTTAATGAGTA
AGTTAGAACTAGAAAATAACTCAGCTGAAACCGCCTTATTTACCAGACTTATT
CAAGATAGCATAGAAAACTTGATCCTAAAACTCCCTAACCATATTCATCTAAG
AATAGTTAACTGCCAAATCCCCGATTTAAGATCCCTTATTAATATCTTACAAGA
AAAGGGTCTATATGAACCTCAAACATTAAACAAAGATAACTTTAAGCCAGCT
CCTAATTCTAATAGGACGCCAAATAACTTACCAAATAGACAAGTTAATGGCCA
AAATAAACCTATAACACCTTTTCAACCGTACCATAACAACGTCTTTCAACCCT
ATTATCCACCATACCCATACGCACCAATCCATACACCCAGGCCCAATCAAACC
CCTTTTCCCCAATATCAACCAAGACCAACCTACCCACAACCTAACATACCCAG
ACCGAATCCTGTCTTTAATCGACAGAATATCTTCGACCAAAATCGTTTCGGAC
CTAATCAAACTTACACACCGAATAACTTCCCTCCAACTGGGAACACACAAAC
CAACAACCCGGTAAAAAGACAACGACCATCAGACAGCGGACAGACTAAAAT
GAGCATAGAAGAACTAAGATACCAAGAAATGGTATCAAACCAACCCGAATAC
CCTTACCATTATTACTATCCATACTACCCAGATAACTTCCAACCACAACCATAT
CCTTATCAAATGGGTTATATCCCAGACCCAACTCAAATCCCTTTCGAACAAGA
TCATGACACTTCGGACAAAACACAACAAGAACAGACAGCACCAAACGATGA
CGAATCAGACAATACCAAAGAAGCAGAAAATTTTCGGCTAGTTGCCCCGGAT

CAAACCAATATATAATTATTAAACATAATGACATAGACTTAAAGTGCCTAATTG
ACACTGGATCCACCATAAACATGCTTACTTGGAACATTTTCGAAACACCTATA
CAACAAACCAACCACCTAATTCATACAAGTAATGGMTCGGTAACTATTAACG
AGACCACCACTATACCACCCAATAACTACTTTCCTATTGCTCAGGAGTTTCTA
ATTCACAAATTCTCAGATCATTATGATATGCTGATTGGACGCAAATTGTTGTCT
AAAGCTAAAGCCATAATAGATTATCAAAAGAAAACTGCCACCCTCTTCAACA
AAGTCTATAAAATAAAAGACACTGAAAAACTTACAGACCAAAATCATGCTCA
AGTAGACTCTTCGTTCCCACAAGACCCTACCTTTTTAGAAACCTCCACACTTC
AGGAGAATCTGTTCCGACTAAACCATTTAAATGCAGAAGAAAAATACAAATT
AACAAACTTACTGACAAGATTCAAAGACATTCAGTTTCACGAGGGCGACAA
ACTTAGCTTCACAAACCAAGAAAAACATACAATTAATACCACACATAATATTC
CAGTTTACTCTAAAATGTATAGCTTCCAACAATCATACGAACAAGAAGTAGAA
AGACAAATTCAAGAAATGTTAGAACAAGGCATCATTCGAGAAAGCAACTCA
CCGTACTGCAGCCCCATCTGGGTAGTACCAAAAAAATTAGATGCTTCCGGAC
AGCAGAAACTTCGAATAGTCATAGACTACAGGAAGCTGAATGAAATAACAAT
AAATGACCGATACCCTATGCCAAATATAGACGAAATACTAGGAAAATTAGGGA
GGTCTAATTACTTTACCACCATAGACCTGGCAAAAGGCTTCCATCAAATAGAG
ATGGATTCAGAATCAATAGCCAAAACGGCTTTCTCTACTAAATACGGACATTA
CGAATATACTAGAATGCCATTTGGGCTTAAAAACGCCCCAGCTACCTTCCAAC
GCTGCATGAATAACCTACTTCGTCCGCTTTTAAATAAAAATTGTTTAGTATATC
TAGACGATATCATTGTCTTTTCTACCTCTCTAGAGGAACACCTTCAATCCCTTG
AAGCAGTCTTCGAGAAATTATCTCAAGCCAACCTTAAACTACAGCTAGATAA
ATGTGAATTCCTAAGACAAGAAACTACCTTTCTAGGACACGTTATCACTAAAG
ACGGAATTAAACCAAATCCCGAAAAAATCAAAGCTATACAAGATTACCCACT
TCCATCTAAACCTAAAGAAATTAAAGCATTCCTTGGAATCACAGGATACTACA
GGAAATTCATACCCAACTTCTCTGACATAGCGAAACCACTGACTAAATGTCTC
AAAAAGGGAGTAAAAATAGATACAAAACATAAAGAATATATAGAAGCATTTC
AAAAATTAAAATTACTTATTTCCGAAGACCCCATATTAAAAATACCAAATTTG
AAAGAAAATTTGTATTAACAACTGACGCAAGCAATGTAGCATTAGGTGCTGT
TCTATCTCAAGATGGACATCCCATTAGCTACATTAGCAGGACCCTAAACGAAC
ATGAAGTTAATTACAGTGCTATAGAAAAAGAACTACTAGCAATAGTTTGGGCC
ACAAAGACCTTTAGACACTACCTGTTAGGACGACATTTCGAAATAGCTTCTG
ACCATCAACCATTGTGCTGGCTACACAAATTAAAAGAACCTAACTCAAAGCT
GACTAGGTGGAAGATTAGATTATCCGAGTACGACTTCGATATAAAATATATCAA
AGGAAAGGAAAACCATGTCGCAGACGCACTGTCTAGAATCAAAATAGAACA
AGCATTTTTCGGAGAATCTACGCAACATAGCGCAGAAGAAGATAATAGCGAC
TTAATAGGGTTGTCCGAAAAAGCCATTAATTACCATAAAAGACAAATTATTTT
CTCAAAAGGTCCCAATAGTAGTATAGAACACGAAACTTATTTCAAAAAACAA
ATCATACACATTTCCTACAAAGAAATGACCCTAGAAGCAGCCAAACAATATCT
ACTCAATCACTTTTGCTCAAAGAATAGCGCTCTTTACATCGAAAGCGATGCAG
ACTTCGAAATGATCCAAAATGCATACACAACAACAATGAATCCGAAATGTAC
AAAAATTTTCCGAAGCCTCGTTTTATTAAAAAACATCCCTACTTATGCAAGTT
TTAAGGAACTAATTCTAAACACCCACGAAAAGCTCTTACACCCCGGAATTCA

AAAAACTGTAAAACTCTTTAGTGAGAATTATTACTTCCCCAATAGCCAACTCC
TTATCCAGAATATAATCAACGAATGTCAAGTCTGCAACCTAGCCAAAACAGA
ACACCGTAACACGGAAATGCCAATGAAAATTACCCCTAAACCCGAACATTGT
CGTGATAAGTTCGTAATAGACATCTATTCTTCCGAAGGAAACCATTATCTGAG
CTGTATCGATATTTACTCTAAATTCGCCACCCTTGAACAGATTAAGACAAAAG
ACTGGGTAGAATGCAAAAATGCCTTAATGCGCATATTTAATCAATTAGGGAAA
CCTAAACTTCTCAAAGCAGATCGAGATGGTGCATTCTCCAGCCTAGCCCTCA
AACGTTGGCTCGAAACCGAAGAAGTAGAGTTACAACTTAATACTACAAAAAC
AGGTGTCGCTGACATAGAGAGACTCCACAAAACTATAAACGAAAAAATCCGC
ATCATAAAAAACTCTGACGACAACGAGACCAAATTAAGTAAAATAGAAACAA
TCCTTTACATTTACAACCACAAGACCAAACACGATACTACCGGACAAACCCC
TGCTCACATATTCATTTACGCCGGACAACCTAACTTAGATACACAAAACAATA
AAGAAAAAAAAATTAACGAAATAAATAAGAATAGAACTGAATATAATGTCGA
CACCAGATACAGAAAAGGACCATTACAAAAAGGAAAATTAGAAGCCCCTTTC
AAATTAACAAAGAACGTCGAACAAACCGACGAAGACCATTACAAAATTACTA
ACAGAAACAGAGAGACACATTACTATAAGACCCAATTTAAAAAACAGAAGA
AAACTAATCAACTCCCCATTTTACAGGCCCCTTGTTCACCATAATACTTTTTCT
TACTCTTACAAAACTATGCCAAGCCCAGCAACTCAAATAAACAACATCGACA
CCGACCACGGATATCTCCTATTCTCTGACAAACCTATTCAAATCCCTTCAGAAT
ACGAACACCACTGTCTCCGCATAAACCTCTTGGATATAGACAACCTAACTCAC
TATTTTAACACTAAAGTAACCAACTATACACATATCCCCCAAATTAAACTTCTA
CACAATAAAATGTTAAGGGAACTTAACGCTATAACGTTACACCAGACAAATA
GACAGAAACGCGGACTAATCAACATAATGGGATCTGCATTTAAATACCTTTTC
GGGACACTAGACAATAACGACCGGATACAAATTCATAACCAATTAGAGTCCG
CGACAAACAACTCAATTAACATACACGAAATGAGCGACATAATTCAACTTATC
AATAGTAACATGCAAAAGATCAAAGAATTTGAAGACGAACATAATAACAGAG
AACAAATGCTATACGAACTAATACAATTTACCGAATACATTGAAGACATCGCA
ATGGGAATGCAACTCTCACGACTAGGACTGTTTAACCCAAAATTACTTAACTA
TGACAAATTAGAAAACGTTGACAGTAGCAACATACTACAGACTAAAACCTCA
ACCTGGATCAACACTAACCAACTACTTATCATAGCTCATATCCCTACTAAACA
AAAAACCATTCATACTATTAACATAATACCCTATCCAGATAATAATGGCTATCA
ACTTGATCATATAGACAAAGACACTTATTTCGAAGAACAAGATAAAATTTATA
ACCAGAACTATCAAGAAATTAACAACGAATGTATCAAAAATATTATTAAGAGA
ACAAATCCAATATGTAACTTTGTCCCTATAACAGTCGAACAAATAATTAAGTAT
GTAGAACCAAATAGTATCATCACTTGGAATCTAAACAACACTATCATAGAACA
AAAATTGCCAAGAAATAAATAAAAATGTAACGGTTAACGGCAACAAAATAAT
AACAATAAAACAATGTAAAATCAAAATAGGAAACATAATACTTAACGAAAAC
AAGCTAAAAACCCCGAGATAAACCTTACCCCATTGTACACACCCTTAAGCCT
AATAAAAATAAAACCAATAGAACATAAGGACATTGTACAATTAATATCAAATA
ATAATATCACAGTTTGTATAGTAGTATCAATCATAAGCCTCACATCTTGTATTGC
TTGTATTTACTTAAAACTAAAAAACAAAATAATCATTGAATCACAAGATGCAA
ATCCAACTGCATCACCAAGATTGCGGCATCAACACCAATAGAAGGAGTGGA
AGCATAAGCTTCCCTTTTAAAGGGAGGGAAGTAACATAATATGCTTCTCATAT

TACGTTTACATACTTACACTAATTGTACATACAATCTTGCACATGCATAAACAC
ATGAAACCAGTTTACATTTTTACTTACACTTAAGCGCATAATTTGTTGTGCATC
CATACCGTTATTTTTCCGTTCTTTTTTGTACACATATACTGATTAGACATTCCCG
TTTCTCGCGACTCACTTCGAGCCGATCAAAAACTCTGTACAGTCAGTCTTAA
GCCGACAACGAAGAAATAAAGATCTAAACTAAAAAATACCTCGTGTTGATTC
TTTCAAATCCGAAACTTCTTTAAAGGCGTTGATCTTAGTCAAACGACGGATCA
TTTGTTCGACTCGAATAGTAAAATACGTAAGT

>ROOA_LTRBEL#LTR/ROOA_LTRBEL

TGTCTAAATATGTGTTTAGACATGATAAGTAGGCAAACTATAAAAATGTTCTAT
TTATGGGCTGCAATAAACATGTCACYGGACAGCATAAGTGGCAACTACAGAT
AAGTACGATTGCAGCGGCCTATTGCCGAAGTGTCAAGAGATATGACCAYGCG
GGAGGTGATTAGCGCGGTCATAGTCCTCAAACATAGATTTAAGAATAAAACTC
AGCTGCATTTACCAACGCAGACTGCGGCGTCTTACAAGCGCTGCATTATATAA
TTAGATGATAAGAACCTATGTAAGAATGAATAAAAGGCGAAGCCCTCGCAGT
AGCGAGTCAGTTAGATTCAAACACCCGAATTGAACTCATTAAGTGTACGCAC
AAGTTTATAGTGTGAACA

>ROOA_IBEL#LTR/ROOA_IBEL

TTTTCGTCCTTTCGAGAAATTCTGTTGTTTTCCTCCACCAGTGGTAAGAAACA
CAGAAGAAAAAACCAGCGCTTCAAAGTAAAAAGAGCAAGGTTATTCGAGTG
ATTCTGTTGTTTCCCCCACCAGTGGTAAGAAACACAGAATAAAAGACCACGC
CTTAAAGTACTAGGACTATAAGGTGAAACATTGTGTTCGTGCTTTTCCTTGGC
TGATCAATCAGCTGTGAGTCGAGGCACAGCTAGGTCAACTGGGCGACCAATC
AAAAAATACTCCAACGGATCACGCCAAAGAATACAAGCAGAAAGCACAAAA
AAGTCACAGTGATTGTTTTCCCAAGATGTTGTCGGAAAGAACTGTGAAATTG
ATGCAAGAACAAGGAGAGCTGCAGAACAAAATACTGCAAGCCATCAAGGAA
AAGGCATCTATACCAGCAATAGAAGTATTGGTAGTCCGATTTACTACTAACAA
CAATGCGCTGGTAAAATTGGGAGTTGGCGATCATCAATATTTTAATGATAAAA
TATTTATTAAAACTATGGATGCTATCAAGGGCTACAAGGAGTCACAATTAAAG
GTAAAAACACTTGAAGGGATAAATATACCAAGCGGATCTAAATTATCTGATAC
CGCATCAGCTCGGAGCTCCAGTCCAGTTGATAATCTGGTTCAACTGGTGGATA
GAAGAGCGGACAGGGTGAGGCAACAGATGAGCCTAATCTACGAAACCCTAG
TGAGATTGAACTAGTCAAGCAGCTGGCAATATGAAAAGTCATTGGTCCAACG
TGACGTACACATGTAAACAAGTATGACACAGAACTGCCTTTGATCAAGATGA
GGCAGACATTCTGCAATCTAAAGTTGCCGCACTGGTACTTCAAATGAAGTTG
GAACAGTTCAAAAGTTCCAACTACGACGTGCCAGAACTCCCAAAAGTGGAC
CTTCCAACATTCAATGGAAATGCGAAGGAATGGCCATCATTTTACGAGCTAAT
AGACAGCAGGAAGGATCTCAGCAACACAAGGAAGCTGGGATATTTAAGAGC
CTGCTTAAAAGGAGAAGCTCAAATGGTGGTTAGCCATTTGATAACGGGATCA
GCGGCTAGCTATACAGCAGCGTGGGAGCTTATCTGCAAACGCTATGAGAATA
GCAGAAAAATATTCTCCCAACACTTCAACAAATTAATGGAACTGGAGTGCTT
GCTGCCCCATGATGAGAAAAATTAAGGAAGTTTTGGATACTGCGACCGAG

AGCATATTCATCATAAAGGAAAAAGGAAAAATAGAAAGCTCTGCTGACGTAA
TTTTAGCAGAAAATTTTCGCCAGATGCCATTCAGCTGTATGAACAGCATGTAA
AAAAGGCAAGAGCGTCCTTACAAGACGTACTGGAGTTTATTGAGCAACAATA
CAACTCAGTAAATGCCATTACCAAAAATACCGCTCAGCTTGCTACAAGAAAA
GTGCAAGTTAGATCGTGTGCCTTTTGCTCTAAGGATGGCCACGATATGATAAA
GTGTCTCAAATTCAAAGCACAATCAATCGAAAAAAGAAAAGAATTCGTTCAA
AAGAACAGTATGTGCTTAAGATGCTTTGGAAAGCATAATGCTATCGACTGCAG
AAAGGAAATTACATGCAATCGATGCTCCAAAGGGCACAACAGCCTTCTTCAT
GAAGACACAAAACGCAGTATCAACAGCAATAGCCTCAAGCAAGGCCAAGAC
ACACTATTGGCTACAGCTGTTGTTTTAGTGAAAAACAAAGCTGGAGGTTACA
ACGAGTTGAGGGCGCTTATTGACGGTGGATCCCAGAAGACGCTGATTTCAGA
GGAGGCAGCACAAATATTAAGGATTCCCAGAGTAAGGAATACTATAGAGGTC
GAAGGTATCTCCCAGACTACTCAATTATCAAAAAATTGCGACCACCTGACGAT
CAAAAAAAATATTCCAAGCAGCTTCAAAACATCAACGGAAGCATTGGTTTTA
CCGACACTCCATAGAGCCCTTCCCAGCAAAATGTTTGATATTGATATCAACAA
AGAGTGGAAGGGCTACAGGCTAGCAGATCCAAGATTCAACGAGCCAAGCAG
AATTGACATGGTGATAGGTGTGGATCTATTTCCCCTGATTATGATGGAGAAAAT
AAAAACCGTGAATGGAATCTTGGGACAAAAAACCAGATTTGGATGGATTGTG
TCCGGAAACATAACTCGAGCAGCAAAGCAAAAAATTATAAGTGCCACTACAA
CAATAAATCTAAAGGACCTGGAACGCTTTTGGGAATTGGAAGATGAAGCCGA
TGAGACGATTACAGACAATGCAGAATGCGAAAGAAAATTCCAAGAAACAAC
TGTCACCAACGAGGAAGGCAGATTTGTGGTTTCAATTCCATTCCACCAATTAC
ACCACAAAGAGGCAAAGCTGGGAGACTCTCGCAAACAGGCAATGGCAAGG
CTTATGCAAATGGAAAAGAAGAATCGGGCTGCATACAACGAATTTTTCAAAG
AAACCCTTAAGATGGGACATTTGGAATCTGTAAAGACAACGGGTCAAGGTAA
ATACTATTTACCCCATCAAGCAATCATCAGGCCTGGAAGCTTAACTACGAAGC
TACGAGTAGTTTTTGACGCATCCGCAAAGACGACAAATGGACTAAGCCTAAA
TGACGTTATTATAGCTGGTCCTAAGATTCAAAAGGATATATTCGATATTCTAATT
AATTGGCGCAAGTGGCAATATGTTATGGTAGCTGACATTGAAAAATCGTATCG
CCAAATAAAGGTTGCTGAGAAAGACCAAGAATACCAATATATCCTATGGAGA
GATGATCCAAAATTGCCGATCAGTGAGTTTAAGTTAACAACCGTAACTTATGG
CACATCGGCAGCACCTTTCTTAGCAGTCCGATGTCTACGAGAGTTGGCAGAT
CGCTTTTGCCAAGAGGATAGCGTCTTAGCAGAAACAATTAGAGACGACTTTT
ATATGGATGACATCATAACTGGTGGAGACACAGTCAATGAGTGCTACGAACTT
CAAAGGAAATCGAGACAAGTGATGGAGAAGGTCGGCATGCATCTGCGAAAA
TGGGTTGCAAATGACGAACGTATTTTAGCTGACATTCAGGACGACGGTGCTAT
GGAGAAAATCTGCATTGAGGAGAATGAATCGATCAAAACCTTAGGACTTCAA
TGGGATCCGAAGAAGGATACGTTTACGTTTTTGGCAGAAAACCCAATGCTAA
CACGCATAACAAAGCGGTTAGTGTTATCACAGTTGTCCAGAATTTTCGACCCA
CTAGGATGGTTGGCACCTGTAACGATTCAAGGCAAATGTTTCATTCAGGAAC
TGTGGAAGTTACCGATGACTTGGGACGTTGAATTGGAATCCAACTTAGCTAA
CTGGTGGATGGAATATGCTAAAGGTCTATCATATTTAGAAGAAATTAGCATTTC
ACGCTGGACTGGATGCTCCAAAGGTATTATGGAGCTACATGGATTCTGCGATG

CATCAGAGAAAGCATATGCAGCGGCTGTGTATACAAAAGTAGGCGGCAGAGT
TACTTTGCTAGCAGCAAAAAGCAAAGTAAATCCTATAAAAAACAGGAAAACA
ATTCCAAAGTTGGAATTATGTGCTGCGCATTTATTAGCAAAGTTATTAGCGAA
AGTGCAGGCTATATGGAGCAACAAGATCACAACGCATGCATGGAGTGATTCG
CAAATTACTATTGCTTGGATACCGAACAAGCGCAGCAAAGATAAATTCGTCA
GAACTAGAGAGGAAGAAATTAATAAACTAATTCCCAATGTCAAATGGAATTA
CGTTAAATCGAAAGACAATCCAGCAGACGTGGCTTCAAGAGGGATATCACCG
CAAGCTCTTAAAATCTGTGAAATTTGGTGGAGAGGGCCGAATTGGCTAGCTA
TAGATGCACAACACTGGCCCACTCAAAAGGAATCGGAAATTGTTGTGGTATC
CACATTGATAAAATCCGAATATCTGCAAAATCATCTTTTATCGAAGTATTCATC
GATCGACAAACTTCTTAGAGTAATGGCGTATGTATTACGCTTCATAACAAAGC
TGAGAGGAAAATCGCAACAGCCGTCACATCTTACGGCAGAGGAATTAAAGC
TAGCAAAGATTGCCGTGGTAAAGATACAACAACAGCTGGATTTTGGACACGA
AGTCAGACTACTCAAAAACAAAAGACCATTAGACCCAAAGAGTAAGTTACA
GGCGCTAAATCCGTTTTTGGATAGTGATGGCGTACTTCGAGTTGGTGGACGAT
TACAAAACGCAATGATACCCTATAATGTAAAACATCCAATTATACTGGACAAG
TCACATTTGACTTGGTTAATTGCAAAGGATGCTCATAAAGAAACTCTGCATGG
CGGAATTAACATTATGAGAACTTATATTCAGAGGGAGTTCTGGATATTTGGCAT
ACAAAATTCCTTAAAGAAATATTTAAGGGAATGTATTGTATGCATACGATACA
AGCAAGAGATGTCCAGTCAACTGATGGGAAATTTACCAGTTTACCGAGTAAC
GACTGATTACTCGTTTCAAAATACTGGAATCGACTACGCCAGACCGTTCCAG
ATTCGCTGCTCAAAGGCAAGAGGTCAAAAAACGTATAAAGGATACATTTGTG
TATTTGTTTGTATGGCAACAAAAGCAATACATCTGGAAGCTGTTAGCGACCTT
TCGTCAGACAAATTCCTGGAGGCTCTTCGACGGTTCTTTGCAAGACGAGGCA
AGAGTGAGAACCTATACTCAGATAATGGAACAAACTTCGTGGGAGCTTCAAG
AGTATTGGACAAAGAATTTGTAGCTGCCATTAAAAACAATAATGAGTTAGCAC
CTACTCTAGAAAAAGAAGGCATCAAGTGGCACTTTATTCCCACGGGAAGCCC
CCACATGGGATGTTTATCGGAATCCGGTGTAAAATCAGTGAAGCATCACCTTA
AACGAGTTATTTGTGAAAACAGCTTTACATATGAAGAATTTGCATCGTTGCTA
TGTCAAATCGAAGCAGTGCTAAACTCGCGTCCATTAGTCACTGTAAGGAGCG
AAAACGATGGTCAGTACATATTACCGCCGGGTCATTTTCTGGTGGGAAGACCT
CTAATTGGAGCTCCTGAACATTTTGGAGAAAGTAAGACAATCAGCTCTTTGG
ATAGATGGAAGCTTATTCAACGCATCAGAGGTGATTTTTGGAAGAAATGGAA
AGAGGAGTATCTGGTGTCATTGCAACAGCGAACCAAATGGCGCCAAGAAAA
GCCAAATCTGAAGGAGGGACAGCTGGTTCTTATAAAACATGAGAACACTCAC
CCTGCAAGATGGCCTGCATAAAACAATCAGAGGACTTCCTGGGAGACTTCAA
GGACTACTGCGATTTCTTCGGCACCACAATACGGACAAAAATTGACAACATC
AAAGAAAAAGACAAAATACTACGGCACCGTACCAACCGAAAGAAAAGGTTT
ATACTGTTCTTTGATATCGGAAATGCAAATAGAATACAGGAAAATATGCAAGC
GATCATAAAAAACGAAAAACATCTAATGGAATCATAGCTACTGTGCTATGCTG
TATGTTGACAATCAGACCTCGGTCATCAATGCCACGGAAAAATTAGTAAGAC
CAACTACGATGGAAGCAAACCGGAATTTGGGAAAACTGACCCAACAAGTCA
ACATTATTGCAGAAACCATGAAGGAGCACTTTATGGTATATAAGGAGTCAATT

AAATTCCTTATGTTATCAAATCAAGTGCGAAACTGGATTGAACAGGCAGAAA
GCCTACAAGCAACAGCGATCTCAATGATAACGGACATTAGTGAAGGAAGAAT
TCATCCTACACTAATTGCGCCTAACAAAATGCTGGAGGAGTTCGAAAAAGTT
AAGCAAAAATTAGGACGAAATCAAATGCTACCGAGTGGAAATTCAGTTATAC
AATTACCACTGATCTATAAACTGATGAAGGCCCAAGCTATGTTGTGGGAAAAT
TTACTATTCATCGAAGCAAAATTGCCGATATACAACAATCAGGAAACGGATCT
CTTTGAAGTAATCCCAATACCACTGTGGACAAACGGAACAAAGCTTATTCCG
AAATTGAATTCTACATTTTTTGCGTATTTTTTTTCAATACAGACATAAACGCA
TATCACCTAATGTCTGAAATGGAAATTAACCAATGCAGACAAGAGGATTCGA
CAAGGCCATGGCTTTGCCAGAAAATAATTGGGCATGGAAAAACGCGGATGAT
CACTCTTGCGAAWTATCACCATTGAAACCAAGCAAGGCACACTCATGCGAA
ATGATGGAATTCCAAGGCAATTCGTTCATCAAAGAGATAAGTGGATCCAACC
GTTGCCTATTTAGACTGTTTCGGAATACAACAGCTAATATAAGATGCAACGAA
CAGCATCAAGACAGAACAGCTTATAAGACTGCCCAATCAAGGCATTATACAA
CTACTTGCAGGATGCACAGCAATATTAGGGGATACAACAATAATTACTCCTCA
AAAAGTAATTTCGACAGCGTCTGAAATGTCTATTATCTTTCCCAGTTTACGAA
TTATAGACGACAAGGAGAAGGAGGCATGGAACGTGGTCCCGCTGAAGCACT
TGATTGTCAACAACACTAATGAACTGCAAAATCTTCAAATGCGCATCAAGAC
TCTGAAAAATAACAAGGTACACATTGATGACTTGATTTTCCACACGGCAAGC
GGACACTCGGCTCTAGGGTTGACAACGATTATCATAATTATATTGGTCATTTAT
ATCCGGAGGCAACGCATAAATGAGAGACGACTACTGGCCGTACACTCAAGG
GA

>ROO_LTRBEL#LTR/ROO_LTRBEL

TGTTCACACATGAACACGAATATATTAAAGACTTACAATTTTGGGCTCCGTT
CATATCTTATGTAAATGAATCGAGAGCGATAAATTATATTTAGGATTTTGTTATC
TAAGGCGACATGGGTGCATTGCTCAAAAACATGTAATTTAAGTGCACACTAC
ATGAGTCAGTCACTTGAGATCGTTCCCCGCCTCCTAAAATAGTCCCTTAGTGG
GAGACCACAGATAAGGTCCTCGCCGCTCAAGATAGGCAGATGTGCCCGAGC
GTGGGACCTCGATAAGGCGGGGACTATTTACTTAGGCCTCTGCGTAGGCCATT
TACTTTAAGATGCGATTCTCATGTCACCTATTTAAACCGAAGATATTTCCAAAT
AAAATCAGTTTCTTACAAAAACTCAACGAGTAAAGTCTTCTTATTTGGGATTT
TACA

>ROO_IBEL#LTR/ROO_IBEL

TTTGGTCAATCGAGCCTTTAATCGACTCTGCAGTTTCCCCCTACCAAAGGTAA
GGAACTCAGAGAAAGGCCAGCTCCTTTAAGCATCTTACAGCTAAAGGTAGCA
AAAATAAGTGACTCTTGTTTCCCCCTACCAAAGGTAAGGAACAGAGTATAAA
TATAAAAAGCAAAAGATACAAAAGAATCTTTTATGTTTTAAAACAAGCACCTT
ATAGTCTATAGCTAAAGGTTGCTTTGTGTACCATTATAAATTGTGGTAAGGCGT
GCTTGAGGCCATACATCAGCAATTGTGAAATTAAAAAGTGCATAACAAAAGT
GCCTTATAAATGCTCTAATAGCATTAAATCAGCTCATAAATAGAGTGCAGTGTA
TATGCCATAAGAGCATAAATTAAATAAAAAGTGCCTGAAAACAGTGCCTTATA

AATGCTCTAATAGCATTAAATCAGCTCATAAATAGAGTGCAGTGTATATGCCAA
AAGAGCATAAATGCCGAAATAAATGGCTAAAAAACAAAAAATCTGACTGGAC
TACAAAAATAATAAAACGTGCCAAAAAAAAAAAAAAAAAAAAAAAAAATCAT
CTTTAAACATCGACGGAGCCTTAAAGAAGAGAAGGAAGTCAAATTCAAAGG
AGCCTCTACCAGCAGCAGAAGCAGCAACAACAGCAGCAGCAGAAGCAGCA
ACAGCAGTAGCAACAGCAGCAACAACAGCAGCAACAGCAGCAGCAACAAC
AACGACATCAGCTAAGTCAAAACAAGAATTTTCTGTTTATCCAAACACACAT
ATATATATAAATACATATAAAATACATATACACGTACTATATATATTAAGAAATTA
CAAAAAATTTTCAAAATGATGTCAGAAAAGACTATTCAATTCCTTAAGAAGC
AGTCCGAAATTATTTTGGAAATTAGAAAGTTGGAAGTAAAACCAACATTAAC
AGATGTAGAAATTCTAAAATTAAATGAGCTTCAAAAATGTTTCATTGCTAATC
ATAGCAATTTGTTAAAGATCGGCGTTGTCGATCATGAATATTTTAACGCGAAG
CAGTATGATTTAATAATGATGGTGTTAGAAAAAATTAAAAATAAAAATGAAAA
AATTAAGGGCGAGTCGGTAGAAAACACTTTCCCTAAATCAAACACTGTCCCT
AAATCAAACCCTCCCCCTACATTAAACCTTGAAATGCGTGGTCACCCTGAAA
AAGAGGGTATAGCACAAAACAACGCTTTAAAAGTAGAGCAGGCATTTCGTAA
TAATGTTGGCCAATTTCGAGTATATCTAGAAGATACGTCTAAACTAATAGACA
GTAGTCCAGATTTCCTTAAAATAAGGAAAAATAAAATTGAATTTTTATGGCAT
AAAATAGATAACCTGATTGAACAGGTGAATAGTCATTTTGAGAGTTCGCTATT
CGAAGAAGAAATTAGCGAACTTGAATTTGACAAACAAAATATTCTTACAGCC
ATTAATAGTCGACTCAGTGGCACAATAAATAAAGCTGAAATGTCGACGGTTGT
TAAGGCGGAGGAGTTACCAACCCTGCCTAAAATACAGATTCCCACCTTCTTT
GGTGATTCCAAAGAATGGGATCTTTTTAATGAACTCTTTACAGAGCTCATACA
TGTGAGAGAGGATCTCAGTCCTTCTCTCAAATTTAATTATCTAAAGTCAGCAT
TAAAAGGAGAAGCCAGAAATGTGGTTACTCATTTACTGCTCGGCTCTGGAGA
AAATTATGAAGCCACTTGGGAGTTTTTGACCAAGCGATATGAGAATAAAAGA
AACATATTCTCAGATCATATGAATAGGCTTATGGATATGCCAAATTTAAATTTA
GAATCCAATAAGCAAATAAAGACATTTATTGACACGATTAACGAGTCAATTTA
TATTATAAAATTAAAGGCACAATTACCAGAAGATGTGGATGCAATTTTCGCTC
ACATAATTCTTCGGAAATTCAATAAAGAATCACTCAATTTATATGAAAGCCATG
TTAAAAAGACAAAAGAAATACAGGCACTTTCTGATGTCATGGACTTTTTAGA
GCAAAGGCTCAATTCTATATCATCATTCTCACAGGAAGTAAAACCTGTAAAGA
AAATGATTAATAATAACAAGAATAAAAATTATAGTGACAATTGTGCATATTGCA
AACTACCAGGGCATTATTTAATTCAATGCCATAAATTTAAAATAATGAATCCAG
CAGAACGGTCTGACTGGGTAAGAAAAAATGGGATTTGCCTAAGATGTCTGAG
GCATCCGTTTGGTAAAAAATGTATAAGCGAGCAGCTTTGTTCGACTTGTCGTA
AACCTCACCACACGTTACTTCACTTTGCAGGTCATAATCCAGAAAAAGTGAA
TACGTGTAGAACAACAGGTCAAGCCTTGTTGGCCACGGCCTTGATTCAAGTA
AAGTCGAGGTATGGAGGCTTTGAACAATTAAGAGCATTGATTGATAGTGGCT
CTCAAAGCACAATTATTTCAGAAGAGTCTGCACAGATTCTAAAATTGAAAAA
ATTTCGGTCTCATACTGAAATAAGTGGAGTATCTTCCACAGGAACGTGCATCT
CCAAGCACAAAGCGGTTATTTCGATAAGAAATTCTCCGAAAAATTTAGAAATT
GAAGCAATTATTCTCCCAAAACTTATGAAGGCACTTCCAGTCAACACGATTAA

TGTTGATCAGAAAAAAATGGAAGAACTTTAAATTAGCCGACCCCGATTTTAATA
AACCGGGTCGCATTGATTTAATCATTGGAGCAGACGTATATACTCACATTCTG
CAAAATGGAGTTATAAAAATAGACGGTCTCCTTGGGCAAAAAACTGATTTCG
GGTGGATAGTTTCTGGATGTAAAAAATCCAAAGGAAAAGAAACCATTGTAGC
CACAACAATAGAAATAAAAGAGTTAGATCGCTACTGGGAAGTGGAAGAAGA
AGAAAAAGATGATATCGAGTCTGAAATCTGTGAAAATAAATTTATCAAAACG
ACAAAAAAAGATTCAGATGGGCGATACATTGTGTCAATTCCATTCAAGGAGG
ATGTCACCTTAGGAGATTCAAAGAAACAAGCGATAGCTCGTTACATGAATCT
GGAGAAAAAACTAAAAAGAAATGAAAAACTTAAGGTTGACTACACTAAATT
CATGAATGAATACATGGATTTAGGACACATGATTGAAGTGAGTGATGAAGGC
AAATATTTTTTACCGCACCAGGCAGTGATTAGAGATTCAAGCCTTACGACCAA
ATTGAGAGTAGTTTTTGATGCTTCAGCAAAAACTACGAATAACAAAAGTTTG
AACGACATAATGTGGGTTGGGCCACGAGTTCAAAAAGATATTTTTGACATTAT
TATTAAATGGAGAAAATGGGAATTTGTTGTTTCGGCAGACATTGAAAAGATGT
ACCGACAAATTAAAATAGATAATAATGATCAAAAATATCAATATATTTTATGGA
GAAATTCTCCAAAAGAAAAAATTAAAACATATAAATTAACCACAGTCACTTAC
GGAACTGCATCTGCACCATATTTGGCTACCAGGGTTCTGGTAGATATTGCAGA
TAAATGTAAAAACCAAGTTATTAGTGCAATAATTAGGAATGATTTCTATATGGA
TGACCTAATGACTGGAGCTGATTCGGTAGAAGAAGCTAATAAATTAATAACAT
TAATTCCCCATGAATTGCAGAAAGTTGGATTCAACTTAAGGAAATGGATTTCC
AACAATTCCAAAATATTAACCACTGTGGAGGACACAGGGGACAATAAGGTTC
TCAATATTATCGAAAATGAATGTGTTAAAACTTTAGGACTAAAATGGGAACCT
CAAAAGGATTTATTTAAGTTCAGCGTAAATTGTAATGATGAATCAAAAAATAT
AAATAAGCGCGTTGTGTTATCAACGCTAGCAAAAATATTTGATCCGTTAGGAT
GGTTGGCACCAGTCACGGTTTCAGGAAAACTTTTTATTCAAAAACTTTGGAT
AAATAAAAGTGAATGGGATCAGGAATTATCCATAGAAGATAAAAATTATTGGG
AAAAATATAAAGAAAATTTATTATTGTTAGAGAATATTCGAATCCCAAGGTGG
ATTAATTCAAACAGTTCTTCAGTCATTCAGATTCACGGATTTGCGGACGCCTC
CGAAAAAGCATATGCTGCAGTAGTCTATGCTAAAGTAGGACCTCATGTTAATA
TAATAGCTAGCAAAAGTAGAGTCAACCCTATAAAAAATAGGAAGACAATTCC
CAAACTCGAGCTGTGTGCAGCTCACCTGCTTAGTGAATTAATCCAAAGACTA
AAAGGATCAATTGACAATATAATGGAGATCTATGCTTGGAGTGATTCCACGAT
TACCTTAGCATGGATTAACAGTGGTCAAAGTAAGATCAAATTTATAAAAAGAA
GAACGGATGACATTCGGAAATTAAAAAATACTGAATGGAATCATGTTAAGTC
AGAGGATAATCCAGCAGATTTAGCATCCAGGGGAGTGGATTCTAACCAGTTG
ATCAACTGTGATTTTTGGTGGAAAGGTCCGAAATGGCTAGCAGACCCAAAAG
AACTTTGGCCTCGGCAGCAGTCTGTAGAAGAACCTGTCTTAATAAATACGGT
ATTAAATGACAAAATAGATGATCCTATTTACGAATTAATAGAAAGGTATTCCAG
TATAGAAAAACTTATACGTATAATAGCATACATAAATAGATTCGTGCAGATGAA
AACAAGAAATAAAGCCTATTCATCAATTATTTCAGTAAAGGAGATAAGAATAG
CGGAAACAGTTGTTATTAAGAAACAACAAGAATACCAGTTTAGGCAAGAGAT
AAAGTGCCTTAAAATCAAAAAGGAAATCAAGACAAATAATAAAATATTGTCA
TTGAATCCATTTTTGGACAAGGATGGGGTTCTAAGAGTTGGAGGAAGATTGC

AAAATTCCAATGCAGAATTTAATGTTAAACATCCAATCATTTTAGAAAAATGC
CACCTAACAAGCTTATTAATAAAAAATGCTCATAAGGAAACATTGCATGGAGG
GATAAACCTAATGCGAAACTATATCCAAAGAAAGTATTGGATTTTCGGGTTGA
AAAATTCGTTGAAAAAGTATTTAAGAGAATGTGTAACGTGTGCAAGGTATAA
ACAAAATACAGCTCAGCAAATAATGGGTAACTTGCCAAAATATAGAGTGACG
ATGACATTCCCGTTTCTTAATACTGGAATAGATTACGCAGGTCCTTATTATGTT
AAATGTTCAAAAAATCGTGGCCAAAAAACATTTAAAGGATACGTTGCTGTATT
CGTTTGCATGGCCACCAAAGCCATACACTTAGAAATGGTAAGCGATCTAACTT
CAGACGCATTTTTAGCAGCACTCAGAAGATTTATTGCTAGACGGGGAAAATG
TTCCAATATCTATTCAGACAACGGAACAAATTTTGTAGGAGCTGCAAGAAAAT
TAGATCAAGAGTTATTTAATGCAATACAAGAAAATATAACGATTGCAGCGCAG
CTTGAAAAGGACAGGATTGATTGGCATTTTATTCCCCCGGCAGGACCTCACTT
CGGAGGTATTTGGGAAGCTGGAGTTAAGTCAATGAAATACCATTTAAAGCGT
ATAATCGGCGACACTATTTTGACTTACGAAGAAATGTCAACTCTTTTATGTCA
AATAGAAGCATGCTTAAATTCAAGGCCATTATACACTATAGTTAGTGAGAAGG
ACCAACAAGAAGTTTTAACACCAGGTCATTTTTTAATTGGAAGACCACCTTTA
GAAATAGTCGAACCAATGGAAGATGAAAAAATCGGAAATTTGGATAGGTGGA
GACTTATCCAAAAAATGAAGAAAGATTTCTGGGTTAAGTGGAAAAGTGAATA
TTTGCATACGCTCCAGCAAAGGAATAAATGGAAAAAGGAAATTCCTAATATAG
AAGAAGGGCAAATAGTTTTATTAAAGGATGAGAATTGTCATCCTGCAAGATG
GCCTTTAGGAAAGGTGGAAAAGGTGCATAAGGGGAATGATGATAAGGTCCG
AGTGGCTAAAGTAAAGATGCAGGAAGGATATATCACTAGACCCATTACTAAA
ATTTGTCCCTTGGAAGGAATAAAGTCTGTTGACAAAAATGAGGCTGACCAAG
AGCCAAAAAGACGAACTAGAGCGACATCGGGAATGTCCAAGATCGGAATCA
TTATGGCAATGTTGTTGTTTGTGTTAAGTTGTCAAGTTTCTAGCGCATTACCTA
AAGATATAGCACCAAGATATTCTATAGACAAAATAAATAAAACCTCAGCAATA
TATCTAGACCCGCTAGGAGATGTTGAGATTGTGAGTACTTCTTGGAATTTGGT
TATCTATTATAAAATGGATCCATATTTTAAAATGTTAACAAAGGGTAATGCGCT
TATACAAAGTATGAGGAAAGTTTGCGAAAGACTTCATAGCTTTGAAGAGCAA
TGTAGTCTAGTCTTAGATAATATGCAAAGTCAGTTATCGGAACTTGAAGAAAA
CAATAAATTGTTTATGATACAGTCTAGATCTAGAAGCAAGCGTGCTCCTTTCG
AATTTATGGGTTCCTTGTATCATATTTTATTTGGTATAATGGATGAAGATGATAG
AGAGCAATTAGAAGAAAATATGAAGAATTTGTTAGATAACCAGAACAACCTT
GATAAACTAATTCAAAAACAAACATCTGTGGTTGATTCAACTTCTAATCTATTA
AAGAGAACAACAGAAGATGTTAACTCCAATTTTAGAAGTATGCAAATAAGAA
TTGAGAACATGACAGAAGTTCTTAAAGAAAATTATTATGTTTATAAGGAATCA
ATAAAATTCTTTATGATTACGAAACAGCTACACTCATTGATTGAAGAAGGCGA
AAAAATTCAAGCAGGCATTATAAGCCTGTTGATTGATATTAATCACGGTAGGC
TAAATACAAATATTCTCAGGCCAAATCAGCTTAAAAAAGAAATTGCCAAAATT
CAGCAGAGTCTTTCGGAGAACCTAGTAATTCCAGGAAAACGGTCAGGTACG
GAACTTAAGGAGGTGTATACACTGTTAACAGCCAGGGGTTTATTCATCGACG
ATAAATTGATCATTAGTGCAAAAGTGCCTCTGTTTAGCAGGCATCCATCCAAA
TTGTTCAGGCTTATTCCGGTGCCAATTCGAAATGAAGATCGGATAATAATGGT

GCATACAACGTCCGAATATTTAATTTATAATTTTGAGATAGATTCCTATCACATA
ATGACGGAAGCCACATTAAATCAATGTCAGAAATGGCAACTAAATAAGAGAA
TATGCAAAGGAAGTTGGCCCTGGAATTCAGCGAATGATAATGCATGTGAGATT
CAGCCTCTAAAGCCAGATAAAGCGGCGAACTGCATCTATAAAACAGTAGTCG
ACTCTAAAAGTTACTGGGTAGAGTTAGAAAAGAAAAGTAGTTGGTTGTTTAA
GGTTCCTGCGAATTCAAAAGTCCGTCTGCAATGTACTGGCTCTCAAATTGAAT
TGTTTGATTTGCCTCAGCAAGGAGTTTTAAGCATTGCGCCATATTGTACGGCA
AGAACCGACGATAAAATTCTAGTTGCCCACCATAACATTCAGTCCGAAAGTG
AAGAATTATTATCAACACCTTATATAGGAGAAGTTAGTGAAGCGCCGAAGATT
ATTTGGGATCCGCTGAAACTATCAATATTAAATCATACTGAGGAATTTGAACG
ATTGAATAATGAAATTAAATTTATGAAAGAGAACCATCAAAAATTGAAAGATT
TACATTTCCATCATATTTCCGGACATGCTGGATTAATTATTGCTTTAATACTAAT
GATAGTATTAATAATATATTTCATACGGAAATGTGCTGTGCAACAAAGAATGCA
AGCAATAACCTTTGCAGGTCCGTTGCCAGTACTATAAATATCAATAGTAAATA
AACAATAAAATAATATAACAAATAAAAATATACAGTCCACTATATCGTTGTTTA
ATAGAAAATGTACTTCTACATAGAAAAAGCAAAATGTTTAAAATAAGTTAATT
GAGTACAAATTGTTGAATTAAAAATAATATAAACCATAATTGTAATCCAATAAA
ATTAAAAGCCAGAAAAACTAGGCCCATTGAAATCTTAGTTGCAAAATAAATG
AACATATATCAAATAAATACAGTCCACTACTGTTATAAATGCAACTAATATACT
AATGTACATCTCAGCTTTGCTGGCCCTTTGGCAGAA

>R2_R2#LINE/R2_R2

TTGGGGATCATGGGGTATTTGAGAGCAGAGGGGGAGTATTCTTCTGTAATTCG
TAAGTCATATCATATGATGTGCGGAAGGGGAATTTTACTCTGTAACTCACAAG
TCTCTCCTTTACTCAAGTCGACTCAAAACCTCCTCGTGGTGGTCCCGGTAATG
CTAAACTCGTTTAGCAGCTAATTTGAGCGGAAAAACTTTTCCGATGGGCTGG
TTCCCCAGAGGAAATTTATTCATATTGGAACTACAAGCACAAATAACGAGCCT
CGGATACCTTTACACAATCTGATGACGACCCGACCCTCCGTGGATATCTTCCC
GGAGGACCAATATGAACCAAACGCAGCGGCTACTCTATCTAGGGTTCCCTGC
ACAGTATGTGGCCGGTCCTTTAACAGCAAGAGAGGACTCGGTGTTCACATGC
GATCTCGGCACCCAGACGAACTTGATGAAGAACGTCGACGTGTCGATATAAA
GGCAAGATGGAGTGATGAAGAGAAGTGGATGATGGCGAGAAAGGAGGTTGA
GCTCACAGCAAATGGATGTAAACACATAAACAAGCAACTAGCGGTGTATTTT
GCAAACCGCAGCGTCGAAGCCATCAAAAAGCTAAGACAGAGGGGCGATTAT
AAGGAGAAAATAGAGCAGATAAGAGGGCAATCAGCTCTCGCCCCGGAAGTT
GCTAATCTAACCATAAGGCGCCGCCCTAGTAGAAGTGAGCAAGACCACCAAG
TAACAACATCGGAAACAACTCCAATCACTCCCTTCGAACAGTCGAACAGGG
AAATTTTGCGGACACTACGCGGGTATAGCCCCGTAGAATGCCATTCCAAATGG
AGAGCCCAAGAGTTGCAAACTATCATTGATAGGGCACATCTCGAGGGAAAGG
AAACCACTCTCCAATGCTTATCGCTATATCTCCTGGGAATTTTTCCGGCACAG
GGTGTACGACACACACTGACGAGACCTCCTCGGAGACCTCGGAACAGGAGA
GAAAGCAGAAGGCAGCAGTACGCTGTCGTCCAGCGTAACTGGGATAAGCAT
AAAGGAAGATGCATCAAGTCCTTGCTAAATGGAACTGATGAGTCGGTAATGC

CAAGCCAAGAAATAATGGTTCCCTACTGGAGAGAAGTAATGACTCAGCCTAG
CCCAAGCTCTTGCAGTGGAGAAGTGATACAAATGGATCACTCGCTTGAGAGG
GTATGGTCTGCTATTACAGAGCAGGACCTTCGGGCGTCAAGAGTCTCATTATC
CTCGTCTCCGGGGCCTGACGGGATAACTCCAAAATCTGCCAGGGAGGTGCCG
TCAGGTATTATGCTGCGCATAATGAACCTAATTCTATGGTGCGGTAATCTACCA
CACTCCATACGACTGGCCAGAACCGTCTTCATCCCGAAGACGGTGACGGCGA
AGCGACCGCAAGACTTTCGTCCAATATCAGTGCCTTCAGTCCTGGTAAGACA
GCTAAATGCAATATTGGCAACCCGGTTGAACTCATCAATCAATTGGGACCCGC
GCCAGCGGGGCTTCTTACCAACCGACGGATGCGCCGATAATGCGACGATAGT
CGACTTAGTCTTGAGGCATAGCCATAAGCACTTTAGATCTTGCTACATCGCAA
ATTTAGATGTAAGCAAGGCATTTGATTCTCTATCACATGCATCTATATACGACA
CCTTACGTGCTTATGGTGCGCCAAAGGGCTTCGTTGACTACGTACAGAACAC
GTACGAGGGCGGTGGTACCAGTCTCAATGGGGACGGTTGGAGTTCAGAGGA
ATTCGTCCCTGCTAGAGGAGTGAAGCAGGGTGACCCTTTGTCTCCTATTCTAT
TTAACTTGGTAATGGACAGGTTACTTAGAACCTTACCCAGCGAAATTGGTGCC
AAAGTCGGAAATGCCATTACTAACGCGGCCGCGTTTGCAGATGATTTGGTACT
ATTTGCGGAAACTCGGATGGGGCTTCAAGTATTGTTGGACAAGACGTTGGAT
TTTCTATCTATCGTCGGCCTCAAACTTAATGCCGACAAATGTTTTACCGTTGGC
ATTAAGGGCCAGCCGAAACAGAAGTGTACCGTGTTAGAGGCACAGAGCTTC
TACGTAGGCTCGAGTGAGATTCCATCACTGAAGCGCACGGACGAGTGGAAG
TACTTAGGCATCAACTTCACTGCAACCGGGAGGGTTCGATGCAATCCGGCCG
AGGACATTGGTCCAAAGCTACAAAGATTGACAAAGGCCCCCCTCAAACCAC
AACAGAGGTTGTTCGCCCTTCGGACTGTCCTTATCCCACAGCTCTACCACAA
GTTAGCCCTTGGGAGTGTGGCGATAGGCGTCCTAAGAAAAACTGATAAACTT
ATAAGATATTATGTGCGAAGATGGCTAAATCTTCCGCTGGATGTGCCGATAGC
ATTTGTTCATGCACCCCCAAAAAGTGGAGGTCTCGGAATTCCATCACTAAGAT
GGGTAGCTCCAATGTTAAGGCTAAGACGCTTGAGTAACATTAAATGGCCTCA
CCTCACGCAAAACGAGGTAGCCAGCTCTTTCCTCGAAGCCGAAAAACAACG
GGCCCGAGATAGATTATTAGCTGAACAAAATGAACTGTTATCGCGTCCGGCA
ATAGAAAAATATTGGGCGAACAAGTTGTACCTCTCAGTTGATGGTAGCGGAC
TCCGTGAAGGCGGCCATTATGGCCCGCAACACGGGTGGGTTAGTCAACCCAC
GCGTTTATTAACAGGAAAGGAATATATGGACGGTATTCGTCTGCGGATAAATG
CCCTACCCACAAAGTCTCGTACTACAAGGGGAAGGCACGAATTGGAACGAC
AGTGTCGTGCAGGATGTGATGCTCCCGAAACAACAAACCACATAATGCAAAA
ATGCTACCGCTCGCATGGGAGGCGGGTAGCTAGACACAACTGCGTAGTAAAT
CGAATCAAGCGGGGACTTGAGGAGAGAGGCTGCGTGGTCATTGTTGAACCA
AGTCTGCAGTGCGAATCCGGCCTTAATAAACCAGACCTGGTGGCACTACGAC
AAAATCACATTGATGTGATCGACACACAAATTGTGACAGACGGACACTCTAT
GGATGATGCGCACCAGCGCAAAATCAATAGATACGACAGACCGGACATACGA
ACTGAATTGCGTCGCAGATTCGAAGCCGCAGGTGACATTGAATTCCATTCTG
CCACCCTGAACTGGAGGGGGATCTGGAGTGGTCAATCCGTTAAAAGATTGAT
AGCAAAGGGTCTCCTCAGCAAATATGATAGTCATATCATTAGCGTCCAGGTTA
TGAGAGGCAGTCTCGGTTGTTTTAAACAGTTCATGTACCTGAGCGGGTTTTC

CCGAGATTGGACTTAGCTAAATCGTTTGGTTCAAAACATTTGCTTGCTGTCTT
GGCATAACATCAATAAAGGCATAAACATCGCAAAATAATGGTTATAATTAAAT
GGCTATGAGGATGGTTTTAGTACGTAGGCGTTGCGGAACTTCGGTTCATATAG
AGCAATGAATCGTGCATGCTAGGAAAACTGACCACACACAGTGTTGGCAGA
CCTAGTATCTTTCGAAGATTTCCATACCTCCGCGATCAAAAAAAAAAAAAAA
AAAAAAA

>QUASIMODO_LTRGypsy#LTR/QUASIMODO_LTRGypsy

AGTAGCATATGTGCCATAGTATAAGACCACATATACTTACACACACACACACA
TACATAACCACATAGGAACAGCCATACACAAAATCATAGAATGCCTTAGGATA
TAAGTCATAAAATCATGTATCCAATAAGAACCCAAATTCTGGTGACAATAACA
AATCCAGCCTACCCTAATGTAAACATTAACATCCGCTGCCGAATCAGTTATTC
CCACCAAAGGGTCAAAACCGCTTACGCAGCTCAAGTTCTCGGCTGATCAGTA
CCCATGAGTGATCCACTCAAGAAGGCACCCAATCCAACAGACATAAACATCC
GCAGCCGCATTCGCGACTCCCACCAGAGGGTCAAAACTGCTAACACAGCTTT
AAGGGTCAAAACTTTCCACGTAGCTCAAACTCGGCTGATCTATATCCATGCGC
AATCCACTCAAGAGAGCGCCTAATTAACGTAAACATAAATGAGAGCCTATGTA
CGCTCAATATGAAGAAGTAAATAAGCTCAGCCAAGGGTTTGCTAAGCGTTCG
CTTTGCAAATTAGATTTAGATTTTATTCATACTTCAATTGTGTAAGACGTGAAT
TTGTCTCCGACATTCGCTTAAGTTTTTGATCAATAAAAATACTTTTTTTTATAA
ACCAACAAACCGCGAAGTTGTAAT

>QUASIMODO_IGypsy#LTR/QUASIMODO_IGypsy

TGGCGCAGTCGGTAGGATCCGCGTGTGAACATAATCTCTTAGAGCTAATAGAT
TAGTTCGAGATTAATAAAGCGCAACTCGTGACGAACAAAGTTTCGCTTGTGC
TTCAGTTCATACAAAGCAGATCCGCAAAAGAACTTATGTGACAAAATATTTGT
GAACAGTTATCTAACTCTCGATGTTGGATACTATTAATACAAATATAAATTAAA
AAACTGCAAGTGACCATTAAAGTGTGACCCACCAAAATAAATAAAAGGTGTG
ACTGTTACCAGGTCAGTGTTGTGTAGCCCATCTAGCATTGATTGGACTGCTAC
AAAGGTGTGCGAATTTTAAATAAATAAAATAAAACCCCGGTATCAATCTACCA
AGTGATTCAAAGAAAAATCAACTATAGCCAACAACATGGCACAACCCAGCG
AACAAATTATGATTCTATCTGAATTACATTTGAATCAGGCTTTGGGCCAGATTC
GTCACATCTCAACATTTGACGGTTCCACAAGAGAACTGGCATCATTTGTGAG
ACGTGTGGACTTCGTGATGTCCCTATACCCGACAACAGACAAGAGGCAACAT
AGTGTACTCTACAGTGCCATCGAGAGGCAGCTGTCCCAACATGCACAAGAAG
TATCCCAGCTACAACAGTGCAACACTTGGGCTGAACTACGATCAGTCCTCATT
GATGAATTTAAAACCCAAATCCCATACGAAGAGTTGCTAAGAAGACTTTACA
ACACCTACTGGAGTGGTAGTATTCGTAAGTTTGTAGAAGAACTAGAAAATAA
GATGTTCGAAATTTCAAGTAAATTATCATTAGAAAATAATTACACAAATACAA
CTCTTTATACCGCCGCAATGGCTAATACTATTAAAGATGTAATTTATAAAAGAA
TTCCAGACAGAATGTTCATGACATTAGCAAGATATGATATTACAACAACAACA
TTATTAAGACAAGTAGCACAAAGGGAAGGCCTTTACGATACAATTGTATTAAA
TACAGAAAAAGCTAAAGCCAAATTAAACAGTCCATCTACACTACAAAGCAGT

AGTAAAAATAATGGTTCTCAGAAGAATAAAGGAAATAATAATGACCAAGGTA
TAAAACCTTATTATATTCAAGCACAGCAAAGTCAAAATCGAAATAATTATGCA
AGTAATAATTCAAAAAAAGTTGAAAGCAACCAAGCGACATACAATGAGTTCA
AACAAAAGTTAGAACAAGGTAGAGCGCAAAACGCTCTAAATTTTCAAAAAC
CCACATCTTATTCTACTACTAACCAGCCCCAAAAAAGGCAGCGTGAAAGTTC
CAGTGGTCAGTCTAAAATGGATACCAGTGAAAATTTTCATCAACTTGCCTCGG
GATCGGAATCAGAAGTAGAAGAAGAACATACCCATTCATACAAATAACCATG
AACGATATCAAACTCAAATGTTTGATTGACACAGGTTCATCAATAAATTTGAT
GAGTAAAAATTTTTTCAATTGTCCGATCAACTCAAAAGCTCCATTAGATGTAC
ACACAATAAACGGTCAAATTATTTTAAAATCAAAAATTACATTAAAACCTAGT
AAATTATGTCCGACAAAGCAAACATTTTATTTACATAAATTCTCAGAAAAATA
CGACATTTTGTTAGGCAGGGAATATTTGGAAGATTCCAAAGCAACTATATGCT
ATACATCCAATACAGTCACTTTAAATAAATACACATTTAAATTGCGACTTGAAG
ATATTAACGAAGAAGAGGAAACACCTAAAGAGAGTTTAAAAGAAAACTTATT
AGATTCCCCAAATATTGAGTATGTCAATTATGTAATTGACAACGAACTCAATG
AAACAAATGAATTTCGACTTGATCATCTAAACGATGAAGAGAGGAAAGCCCT
CACCAGTGTCTTATACGAGTTTAGTGACATACAGTACAAAGAAGGTGAAAAT
TTGACCTTCACAAGTACAACTAAACATGTCATAACAACTAAACACGAAGACC
CAATTTACAGACGTCCATATAAATACCCACAAAGCTTCGACGAAGAAGTCGA
AAACCAAATGAAAGACATGATTCGACAAGGAATCATAAGAAAATCGAATTCT
CCATATTGCTCTCCTGTTTGGGTAGTACCCAAAAAGCCAGATGCATCGGGAAA
ATCGAAATTTCGCATAGTCATTGACTATCGCAACCTCAACGAAATAACCGTTG
ATGACAAATACCCAATACCAGTAATGGATGAAATATTGGATAAGCTTGGAAAT
TGCCAATACTTTACAACCATTGACCTCGCAAAAGGTTTTCATCAAATACAAAT
GGATGAAAATTCTATAGCGAAGACAGCTTTTTCAACCAAAAATGGTCATTTTG
AATATACTCGAATGCCATTTGGTTTAAAAAATGCACCCGCAACTTTTCAACGT
TGCATGAATAATCTCTTAGCAGATTTAATATACAAAAACTGTCTCGTATATATG
GATGACATAATTGTGTATTCCACTTCATTGGAAGAACACATAATGTCTTTGCG
AAAGGTATTCTTAAAGCTCAGAGAAGCAAATTTAAAATTACAGCTAGATAAAT
GCGAATTCCTAAAAAAGGAAACTGAATTCTTAGGGCACATAATTACAACAGA
TGGTATTAAACCAAATCCCGCAAAAATTAAAGCCGTAGTGAATTTCCCTATAC
CAAAGTCCACTAAGGAAATAAAATCATTTCTTGGTCTTTGCGGTTTTTACCGC
AAATTTATTCCAAACTTCGCTAAAATAGCAAAACCAATGACATTACGACTAAA
AAAAGGCTCAATAATAAATATCAAAGATTCAGACTATTACTTAGCTTTTGAAA
AATTAAAAGTGTTAATAACGTCCGATCCCATATTAATACACCCAGATTTTAAAA
AGTCTTTTTCATTAACTACTGATGCTAGCAATTTTGCTATAGGCGCGGTATTAT
CGCAAGAGCATAAACCTATATGCTATGCTAGCAGAACTCTGAACGAACATGA
AGTTAATTATTCCGCAATAGAAAAGGAACTCCTTGCTATTGTTTGGGCCACTA
AATATTTCAGGTCCTATTTATTTGGTAGAAAATTCGAGATCCATAGTGATCACA
GGCCATTGGTTTGGCTTGATTCAATTAAAGAGCCAAACATGAAACTTCAGAG
ATGGAAAATCAAATTAAATGAATTTGACTATCATATCAAATATCTCCCTGGTAA
AGAAAACCATGTAGCAGATGCTCTATCGAGAGTAAAAATTGAAGAAAACTTC
CTAGGAGAAACTTCCAGTAACATTTCGTTACCAACACAAGCTACTATTCACAG

TGCTCAAGAAGATAACCAATCTTATATTTCCTTAACGGAAAGACCAATTAATT
ACTACAACAGGCAGATAGAATTTATAAAAGACGATAATAATAACGTAGAAACT
AAAAGATACTTTCATAAAACAAAAATTAAAATTCATTACATAGAAATGACAAA
TGTCCATGCCAAGGAATTAATTAAAGAATACTTATGTACCAAAAGAAGCGTAA
TTTTCTTTCATAACGAAATAGATTTCCTTACATTTCAAAACGCTTACATAGAAA
TTGTCAGTCCGAATAACATAACTAAAGTTATGAAATCAAACATCAAATTAAAA
GACATTGAAACATATTCCGAATTTAAAGAAATAATTATAAAAAGTCACAGAGA
ACTTTTACACCCAGGAATAGAGAAACAAACTAACCTTTTTAAAGAAAAATAT
TATTACCCAGACTACCAAAAGTTAATACAGAACATTATTAATGAATGCGAAGT
ATGTAACATATCCAAAACAGAACATAGAGATACTAAACTAAGATATGAGTTAA
CACCAGAAACTTTTAATCCCAGAGAAAAATATGTTATAGATTTTTATTTAATCA
ATAATAAAACGTTCCTGTCATGCATTGACATTTATTCAAAATATGCCGCTTTGA
TTGAAGTCAGTAGTAGAGACTGGCTGGAAGCAAAAAGAGCTCTTCTTAAAAT
TTTCAATGAAATGGGAAAACCGTCCGAAATAAAGGCCGATAAAGATTCAGCT
TTTATATGTTCCGCGTTACATTTATGGCTTAGGTCCGAGAATGTGAATATAAAT
ATCACAACAAGTAAAAATGGAATTTCTGACATTGAAAGATTCCATAAAACAG
TCAATGAAAAGTTAAGAATCATTAATAGTGAGTCCGACCCAGAAAATAAGAT
AACACAATTTGAAACTATTCTCTATGTATATAACCACAAAACTAAACACAATA
CAACTGGGAGAACACCAGCTGATATTTTCCTTTACGCAGGAACACCTGCATAT
GACACCCAAAAAGAGAAACAATCAAAAATAGATAAGTTGAATAAAGATAGA
CATAGCTATGAAGTAGATACTAGATATAAACAAGCACCATTGGTAAAAAGTAA
AACAACTAACCCATTTAAGAAAACAGGACACGTAGAACAAATAGATGAGAA
ACATTACGAAGAAACAAATAGAGGAAGAAAAGTTGAACATTATAAATCTAAA
TTTAAAAAACAAAAGAGAATTAATAAAAGTAAATACAATTCCAGCTCAACTC
CCGAAAATCAAGTGGGGTCCGATTAAACAGTTATTCATAATAACACTTATAAT
ATGTTTCATCCGTGCCGTCCGTGGTCAAAGCCTAGAGGTGAACCCGATACAA
GCTAAAAATGGATATCTTATATTTAAAACAGGATCAATTAATATACCTATAAACT
ATGAATATCACTATTTGTCCGTAAACTTAACAAAGACCGAACAAACTTTTGCA
AATTTAATTAAACAAGCTGAAGAATATGGTACTATAGCCCAAATCCAATATTTA
ACTGAAAAATTAGACAGAGAAATGAATGGAATAAGAATAATAAAACGCAATA
AACGTGGTTTAATTAATATTATAGGTACCGCATATAAATACCTTTTTGGCACTTT
AGATCAAAATGACAAAGAAGAATTAGAACAAAAAATATACGATCTTTCTCAA
CATAGTATTCAAATTAATGAATTGAATGAAGTTATAGAAGTAGTCAATAGAGG
CATTGAAGTAATCAACCATTTAAGCGCAATAAGTGAAGGTGACAGAAGACTA
GAACTTTTAGTATTTAACTTACAACAGTTTACCGAATACATTGAAGATATAGAA
CTTGGTATGCAATTAACAAGACTTGGTATTTTTAATCCCAAATTATTAAGACAT
GATCCATTATCACATGTAAATTCCGAAAAATTGCTAAATATAAAAACTTCTACG
TGGTTAAAATCAGATGCAAATGAAATACTTATTATTTCCCATATTCCTAGAGAC
ATTATAAAAACTGCACTTTTTAACATCGTACCATATCCAGACAAAGATAATAAT
ATACTTATAGAAAATGTAAATGATAAATATTATATTCAAGATAATCAAGTTTACA
AACAAAACTCAGGAAAACCAATAATAAACAAATGTATTACAGGAATATTAAAT
CAAATACCAACAGAATGCAGATATTCAAAAACACATAACAATATTGGAATAAC
TTATGTAGAACCAAACATAATTTTAACTTGGAAATTATCAAAAATTGTATTAAA

TCAAAACTGTATAATTAATAGAGAAATTATAATAGAAGGAGACAATATAATAA
AAGCTTTTAATTGTTCTGTTCAAATAGAAAACATATTAATTACAAGCACAACA
CTAGACTACACACAAACGGTCTATATCAATAACAATGTAACAAAATTAGAACC
ATTATCATATCTAAATGCTAAAGAAATAATTAAAGAACACACTAATACATACAA
CACATTACAAATTATTACATTAACTATACTTGCTATTATAATACTTACACTTATAC
TATATTTTATTTATAAATATAAAGGCATTCCCAAAAAACTAATTGTTAAATATAA
AAAAGAGAATCCAAAACAAATAGAACAACAAAATAACACAACAACAGAAA
ATATAAATACAGTACTTGATACTAATCCTGTATTGTATCCTAGAATATCCGCCTG
AGGACAGGCTAAATTTAAAGGATGGGGA

>QUASIMODO2-LTR_Gypsy#LTR/QUASIMODO2-LTR_Gypsy

AGTGACGTATTCACGCCAACTCATCACGCTCAGTTAATTGAATATTCGCGCCA
CCCAAACTGGGCCGCTCTTGTAAACGGATTCCCAAGCTCTCCTCAACGTAAA
CATCCAAGAACATCACATGATTTTACCGTTTAAAGCTATTTTCTGCGTTATCTG
CGTGTCGATCGTTATTACTTCACTTCACAACCTTGGGAGATATTCCGTTTTTTT
CCTATTCTTTATTTCTGTAACCCATAGAGGCCCCTCCTCAATTCTCACTCACAT
TTGTAATCAGTCTTAAGTTGAAAGTTAGACCGTAAAGTTCAATAAGTCTTAAA
CCGATATTTAATAAAAAACAAAACCGAATTTATTCAGTGTTTAATTTATTTCGG
AGTTGATCCTAGTTTAAATACGGATCATTTGTTCGACTTAAAAAAATAAAAAC
TATTTAACTTTCAGCGTTGATCTTAGTTTAAATACGGATCATTTGTTCGGCTCA
ATTAAAAACTATTTAAC

>QUASIMODO2-I_Gypsy#LTR/QUASIMODO2-I_Gypsy

TGGCGCAGTCGGTAGGATAACTCAAAGATTGCTTGATGCGAAAATATAAAATT
TTTAATTCCTGTAATCTGTGATATCCGCCAAAGAACTTACGTAGTGTTACTGTG
AAAGTGAAGTGTTGACCATTAATACGCCTACACTTAAAAGAACAGTGAGACA
CATCTGTTTAAACAACAGTGAACTTCCAACATTTACCGGAGCTATAAATCCAG
AATTTTTTTTTGGTTACTCCGAACAAGTGAATTGCTGGAAAATAAAAAATAAA
AAAAAAAGAACCTAAAACTAAATATATTAAGGAATTACCAAATAAGTGAAAA
AATTAAAATCTAAGTGAAAGAAAACATAAGGAAGTAAACCCAAATCAATCAG
TTAAAAAAAAAAATCTCTAATGCGAACTATTAAATAAGTGCATATAAAACAAA
ACTATATACACTTAAAGAAAATATATATCTAAATAAACTCTATCTAAGCTCAAC
GGAAAATAACAAAAGAAATAAATCTTAACGTGTTATCAAGAGAGGAGAGTTA
TAGACTTCAAACGTAACATAAGTGAAAAATCAATAGGAGTGAATAAAATTTA
AATTAAGACAAATAAAAAACTGAAGATATAAAGTTTGAATAAATTAAAAATAA
ACTAAAACTATACAATACAAAAATAAAAATAACAATAATAAAATTATGAAATA
ACCGCTACTCACGAAACATCAGCAACTATAAACATTTAAAAACAAAAAAAAA
AGAAACCAACAATTTTGTACTCTAATAAAAAAAAAAAAAAAAATGGCAGTA
GCAGCACCAAGCCCAATAATACTGTCCGACTCGAACATGATTCAAGTTGAAC
GACAAATAAATGGAGTCGAACAATTCAACGGGGACCCACAGACCCTGTATAC
GTTCATCAGCCGCATCGATTTCATCCTGGCACTATATCAAACGACCGACGAAC
GGCAGAAGCTCACCATCTTCGGACACATTGAGCGCAACATCGCGGGAGAGG
TCATCCGCACACTAGGAGTTACGAACCTCACCACCTGGACGGAACTCAGGAC

TCATCTGATCCTAAACTACAAACCCCAGAGACCAAATCATCTACTATTGGAGG
ACTTCCGGAATACCCAATTCCGAGGTAACGTTCGTGAATTTTTAGAAGAAGC
CGAACGTAGACGGCAGATATTAACAAATAAGTTAGACTTAGAAAGCGACACT
GCAGAAACTACCCTGTATAACCAACTAATACGAACCAGTATAGAAACATTAAT
TCTAAGATTACCAATTCACATACAGTTAAGAATAGTTAAATGCGAAATTCCGA
ATTTAAGATCATTAATAAATATATTGCAAGAAAAGGGAATTTATGAAATAGCAA
CTACATACAAGAACAATACAAAGCCAGTCTCAAATCCCATTAAATCACCTAAC
AATGCAACTCACAGACAAACAACTAACTACTATAATCATACAACACCATTCCA
ACCATCATACAACGCCATGTATCAACCAATTCGCCAACCAATTTCATATATACC
ACCTCAATTACCCAGAACTAACCCTAATCCGTTTTCCCAATACCAATACCGTC
AACTTCACCCTCAACCTAACGTATCTGTTATAGCTCAACCTAGACCATTGAAT
CGTCAACATACATTTGACCAGAACCGACCAGGACTTAGTTATTCAAACGCAC
TAAATACAAGAGAAAATATAACGACCGGTGGACCAGCACTAAAGAGACAAC
GACCGTCTGACAGTGGACAATCACGTATGAGTTTTGATGAGGCTCATTACCA
AGAAGAATTAGACCAAACTCATCAACAGTTCAATCCTTACATGCATTACGGG
AATCATCCCCAATATCCTTTCGATCTTTACATGCCTTACGGGCCGCCCCCCAAC
AACATCCCGATTTATTACATGCCTTACGACCCCCCACAACAACATCCGGTTGG
CATACAAGAGATGGCAGAGGCACGAGAAGAGCCAACTGAACCTCCTATGGA
ATTAATAACAGAAGCTCAAACGGCTGAGAATTTTCGGCCCCAAGCCTCGGAA
CAAGCCAATTCATAATTATTAAACACAAAGGACTCAATTTAAAATGCTTAATA
GACACAGGCTCAACAGTAAATATGGCCACTCAAAATTTTTTCAAATTACCAAT
CCAAGAGATAGCAAAGAAAATAAACTCAAGTAATGGCCAGTTCATTGCACAA
AAGAAAGTAACGTTACCCAAAAATAATCTTTTCCCTAAACCATACGATTTTTT
CATATATCCTTTTTCAACTAAATACGACTTGATTTTAGGACGACAATTACTTGA
CGAGGGAACTACATCAGTAAATTACGGACCCCGTACAGTTACAATATACGGA
CACGTGCACGAAATGATCGATGCCTTCCTTCCTTCAGAAGAAATACATATTCA
AGATACACAGAATAATTCCTTTAGGCTAGACCACTTAAATTCCGAAGAAAAA
GCAAAATTGATAACCCTCCTAAAAGAATTCCAGGACCTTCAATATAAGAAGG
GAGACCAGCTCACATTCACTAATAACGTAAAACACACTATTAGAACATCCCAT
AATGACCCAGTATACAGAAAGCCTTACGGATACGCACCCGGACTAGATACCG
AAGTAGAAAACCAAATAAAAGAAATGTTAGATCAGGGAATAATCCGAGAAA
GCAATTCCCCTTATTGTAGCCCCATTGTAGTAGTACCCAAGAAACCAGACATC
TCCGGACAGAAGAAATACAGAATAGCCATAGACTACCGTTACCTCAATGAAA
TAACAATAGCAGACAAATACCCAATACCAAATATGGACGAAATCTTAAGCAA
GTTAGGAGGCTGCAACTATTTTACTACAATTGACTTAATCAAAGGGTTTCACC
AAATAGAAATGGACCCCGAGTCTATCCCCAAAACAGCCTTCACAACTAAGAC
AGGGCATTACGAGTATACGCGTATGCCATTTGGACTGAAAAACGCCCCAGCT
ACCTTCCAACGATGTATGAACGATGTACTTCGTCCACTATTAAATAAAATCTGT
ATGGTATACTTGGACGACATTATTGTATTTTCAGCTTCCTTAGAGGAACATCTT
CAATCCCTTAGAGCAGTCTTTCAAGCATTATCTAATGCTAATCTAAAACTCCA
ATTAGATAAATGCGAGTTTTTAAAACACGACACATATTTCTTAGGACATATGGT
TTCTCCAGAAGGTATAAGACCTAACCCGGAAAAACTACGAGCAATAGAAAAT
TACCCCCTTCCTACTAAGCCGAAGGAGATAAAATCATTTTTAGGACTCACAGG

TTTTTATAGAAAGTTCATACCCCATTTCGCACAGATTGCAAAACCCCTAACAA
CATGTCTTAAAAAGGACAAAAAAGTAGATATTAAAAACCCGGAATATATTGA
GGCATTCAAAAGGTTAAAACTCCTTATTTCAAACGATCCCATACTTCGATCAC
CAGACTTCAAGAAAAAATTTGTACTCACAACAGACGCTAGTAATGTAGCTCT
AGGAGCAGTACTTTCTCAAGATGGTCACCCCATAAGTTATATTAGCAGAACAC
TTAACGACCATGAAACAAACTACAGTACGATTGAAAAAGAACTACTAGCTAT
TGTTTGGGCAACGAAAACGTTTAGACACTATTTGCTAGGTCGACATTTTGAA
ATAGCAAGTGATCACCAACCGTTGTGCTGGTTGCACAAGATGAAGGAGCCCA
ACGCTAAATTAACAAGGTGGAAATTCAGACTTGCAGAATACGACTTCGATATT
AAATACGTCAAAGGCAAAGAAAATCATGTAGCAGATGCCCTATCCAGGATTA
CTATAGAGGAAGCGTTCTTTACTGAAGCTACACAGCACAGCGCCCAAGAAGA
CAACCAGAATTTAATTTCCCTAACAGAAAAAGCGGTAAATAATTACAATAGAC
AAGTCATTTTCACTAAGGGACCAGAAAAAGTAAAACAGGAGAATTATTATAA
GAAAAAGATCATTCATATTTCGTACGAAACACTCACTCATAAAAAAGCCAAA
CAGTATTTGATAGATTACTTCGTAAACAACCACAGCGCTTTATACATAGACAG
CGACGCTGATTTTGAAACAATACAGGCAGCCCATAAAGAAATTATCAACCCA
AGCACCACAAAGGTAATTAGAAGCCTAACTTTACTAAAAAACATTAAATCATA
TGCAGAATTTAAGGAATTAATCCTTCAATCCCATGAAAAGTTATTGCACCCAG
GAATCCAGAAAACTAAGAAATTATTTAGTGAAAACTACTTTTTTCCAAATAGC
CAACTACAAATCCAAAACATTATAAACGAATGTCAAGTTTGTAATCTAGCAAA
GTCAGAGCATAGAAATACCAAAGTCCCATTTAAACTCACGCCAAGTATAGAA
TTCTGTAGAGACAAATTCGTTATAGATATTTATTCAGTCGAAGGCAAACACTAT
TTGAGCTGCATAGACACTTATTCAAAATTCGCTACACTAGAGCAGATAAAAAC
CAAGGACTGGATAGAATGCAAGAACGCCCTGATGCGCATATTTAATCAATTGG
GAAAACCGAAGCTATTAAAAGCAGACAGAGATGGTGCATTTTCAAGTCTAGC
ATTAAAAGAATGGTTAGAAACAGAAGGAGTAGAACTACAGCTAAATACCACA
AAAACAGGAGTAGCAGATATAGAACGTTTTCATAAGACCATTAACGAAAAAA
TAAGAATAATCAATACTATGAAAAATACTGAAACAGACCTGAGTAAAATAGA
AACTATACTTTATACTTACAATCACAAAACAAAACATGACACCACGGGACAA
ACGCCCGCTCACATATTCTTATACGCAGGACAACCTACCTTGAACACGCAGG
AACTAAAGAGAACTAAAATAGACAAATTAAACAAAGGTAGAGAAGACCACG
ATATCGACACAAGATACAGAAAAGGACCATTACAAAAAGGCAAATTGGACA
ACCCATATAAACCAACCAAAAACGTAGAACAAACAGACGCTGACCATTACAA
AATTACTAATAGAAACAGAGCTATGCATTACTACAAAACACAATTCAAAAAA
CGAAAGAAAATTAATCAAACCCATACCCCGACTCAAATGGCTACCAGTTAGA
CTACACAGATTCACAATTATATTTTGAGAAAAGAAAACAAAGTGTATAGTAAT
GAAAATAAAGAAATAAACAATGAATGTGTCACCAATATAATTAAGCACCTAAA
TCCAATCTGTAATTTTAAGCCAATACCCACAAATGAATTAATGAAATATATAGA
ATAATGATGTATACAAAACTAAAATTAAGAAAAAACAAAAAAAAAAAAATCA
GGACATACCACCACAAACTGGAATGGAAGAAGTTCCACTACCCTTACTATATC
CATCAGTCCCAGCCCAAGTATAGGCTTATCTTTAAGGGAAGGGAAG

>PROTOP_BP#Unknown/PROTOP_BP

CAAAGACACTAGAATAACAAGATGCGTAACGCCATACGATTTTTTGGCACAC
GATTTTTTCGCCGTGGCTCTAGAGGTGGCTCCAGGCTCTCTCGAATTTTTGTT
CGAGAGCGAGAGAGCGGAGAGCGCTACAGCGAACAGCTCTTTTCTACGCAT
ACAGTGATAGCAGACAACTGTATGTGTGCACACGTATGCTCATGCATTGTAAA
TTTGACAAAATATGTCCCTTCACCTTAGAAGTTCTTAGACTTTAAATCTATATT
ATTTTTGATCAATTGGCACCATGCGAAAAATTCTTGTTTTGCATTGCCTTAACG
TTATTATTATTTAAATATAGCTTAGAAATAGTAATAGCCGAATACATAATATCAC
AAAATAAATTTCAAAAATGACTTTATATTAGAATATTTGTCATTAGAGTATTCA
GCTTGCGACGTGTGAAAAATTAATAAGGCAATGATTGTTGAGTGCTTGTGTCC
GCACTTCGTGCCTCAAGATATGACCAAAGCAAAGACACTAGAATAATTCTAG
TGTCTTTGATATGACTTTTGCAATAAACAGTTTTCATATTTTTATTTATTTTATAA
ATTTTTATTTTCTACTACGTATTATTTTTATGAAATATTTATTTCTCAATGTAATG
TCTTCTTTTTGGAAAATTTATGTTTTAAATTACAGTAGTTATAATAATTTCCTTT
GTATTTGCTTTAATTATTTAGTATATTTATTAAGTCATTTGACTTAATATGATGTA
TGTAAATGATCCAWTTTTATTATTTAAAATGCACATTAGATTCAGTTGTTTTTC
AKTTTCAGTTTTTTTTTTTTTATATTCCAATTTGATATTTTTAAAAAGCGCGCAAT
TTCATCATATTGCTACGAAATTGGCCAAAACTCCCCAAATATGTAAATTCGTTT
CTTCGATCAGAATTGATTTCGGCCCGAAAATCGTCTTCTAGCACAACACGCAC
ACATATACGCGTTCTCGTCTCTTGTTTTTACTCACACAAGCAAGCAAATTCTAT
TTTTAGATTTCTTACGCTCTCAGCGTAAGCGAGCGGACAGAGGGCAATTTTG
GCCGTCACCAAAAAAGTGGCTGCATAGTGCCAAACCAATGTATGGCCGTTAC
GCATCTTGTTATTCTAGTGTCTTTG

>PROTOP_AP#Unknown/PROTOP_AP

CAAAGACACTAGAATAACAAGATGCGTAACGCCATACGATTTTTTGGCACAC
GATTTTTTGGCCGTGGCTCTAGAGGTGGCTCCAGGCTCTCTCGAATTTTTGTT
AGAGAGCGAGAGAGCGAAGAGCGCTACAGCGAACAGCTCTTTTCTACGCAT
AAAGTGATAGCAGACAACTGTATGTGTGCACACGTATGCTCATGCATTGTAAA
TTTGACAAAATATGCCCTTCACCTTAGAAGTTCTTAGACTTTAAATCTATATTA
TTTTTGATCAATTGGCACCATGCGAAAAATTCTTGTTTTGCATTGCCTTAACGT
TATTATTATTTGAAAATAGATTAGAAATAGCCAAATCTATGTACATATTATCACA
AAAATAAATTTCAAAAATGACTTTATATAAGAATATTTGTCATTAGAGTATTCA
TCTTGCGGCGTGTGAAAAATTAATAAGGCAATGATTGTTGAGTGCTTGTGTCC
GCACTTCGTGCCTCAAGATATGAACAAAGCAAAGACACTAGAATAATTCTAG
TGTCTTTGATATTACTTTTGCAATAAACAGTTATCATATTTAATTATTTAGTATAT
TTATTAAATCATTTGACTTAATATGATGTAACATTAACATTAAAAGTGTTTCAA
AAAAAATATTTCGCTTTTAAAAAATTGTCAGATGAGAGACAAATTAGAATTAA
ACATAAATATAAATGTGTAAACGGTAGCTAATTCGAGCGGCGATTTTAACAAA
CAAATTTAAAAAGCTTTAAAATTATAATAGTCAGGGCGCGAATTTTTAAAAAT
TTTTTTATTTTATCATATTGCTAGGAAATTGGCAAAAACTACCCTAATATGTACC
ATGTAAATTCGTTTCTTCGATCAGAATTGATTTCGGCCCGAAAATCGTCTTCTA
GCACAACACGCACACTTATACGCGTTCTCGTCTCTTGTTTTTACTCACACAAG
CAAGCAAATTATATTTTTAGATTTCTTACGCTCTCAGCGGGAGTGAGCGGAAA

GAGAGCAATTTTGGCCGTCACCAAAAAAGTGGCTGCATAGTGCCAAACCAAT
GTATGGCCGTTACGCATCTTGTTATTCTAGTGTCTTTG

>PROTOPP#Unknown/PROTOPP

CAAAGACACTAGAATAACAAGATGCGTAACGCCATACGATTTTTTGGCACAC
GATTTTTTCGCCGTGGCTCTAGAGGTGGCTCCAGGCTCTCTCGAATTTTTGTT
CGAGAGCGAGAGAGCGAAGAGCGCTACAGCGAACAGCTCTTTTCTACGCAT
ACAGTGATAGCAGACAACTGTATGTGTGCACACGTATGCTCATGCATTGTAAA
TTTGACAAAATATGCCCTTCACCTTAGAAGTTCTTAGACTTTAAATCTATATTA
TTTTTGATCAATTGGCACCATGCGAAAAATTCTTGTTTTGCATTGCCTTAACGT
TATTATTATTTGAAAATAGATTAGAAATAGCCAAATCTATGTACATATTATCACA
AAATAAATTTCAAAAATGACTTTATATTAGAATATTTGTCATTAGAGTATTCAG
CTTGCGGCGTGTGAAAAATTAATAAGGCAATGATTGTTGAGTGCTTGTGTCCG
CACTTCGTGCCTCAAGATATGACCAAAGCAAAGACACTAGAATAATTCTAGT
GTCTTTGATATGACTTTTGCAATAAACAGTTTTCATATTTTTATTTATTTTATAA
ATTTTTATTTTCTACTTCGTATTATTTTTATGAAATATTTATTTCTCAATGTAATG
TCTTCTTTTTGTAAAATTTATGTTTTAAATTACAGTAGTTATAATAATTTATATTT
TACTTCCTTTAATTATTTAGTATATTTATTAAGTCATTTGACTTAATATGATTTAT
GTATGTAAATGATCCATTTTTATTATTTAAAATGCACATTAGATTCAGTTGTTTT
TCAGTTTGTTTTTTTTTTTTGTTATATTCCAATTCGATATTTTTAAAAAGCGCGC
CATTTCGGGTGGGAAGAAAAGCGCGCCAATTTTTTTGTTTTTCCTTTTAATTT
CTCTTAGCCCGTTTGCTTAAAGTTGGCTATACGCAGAGCTCCTCGATTTTTTG
ATATTTTCGCTATATAATCGAATTTATATTCAACTAAAGTCGTTTTGTTGCAAAA
GTAAAGTACGGAAATATAAGCGCAAATAGTAATTGCGATAAATAGTGAATAGT
GAATAGTGTTTACGTATTTTCGTAATTTGACGCGCTAATACAACGTGCTGCTGC
TTTTCGACAAAGGTTTGAGTTGAAATTTATAAATTGTAAGTGAAAAAAAAAA
AACATTTTAATGGCCCATTTGAATAACCTTATGGATATGGCCAAGAAAAAAAG
AAGATACATAAGTGGGAACAAGTGCAGCTTATCCCACTGTCGGAAATCTAGG
CGCGATTTCCCCGCTCTTAAATTGTTTAGATTTCCTGAAAGGGATCCAACAAT
GCTAATAAGATGGGCGGAAAAATGCCAATTTACAGAAGATTTTGTGGCGAGC
ACTTCATCACTGTTTTTATGCCAAGATCATTTCTCCCCAGATGATATTGGGGTT
AAATATTTGAAGAAAGGGGCAATTCCAGATCGCAATCTTATAAAATATAAAAG
TTGCAATAATGCCGATATTAATTTAAATGCTGTAGGATCGCCTCCACGAAAAA
GACACCGCTCACAGTCCCCTGTTGAAGTTTGTCGGCAGTGCCATAAAAACGC
GAAAACATTAAAAGTGTTTCAAAAAAAATATTTCGCTTTTAAAAAATTGTCAG
ATGAGAGACAAATTAGAATTAAAATGCTGAGGAAAGAAAACTCAAATCTAAA
AAGAAAATTAGTTAGGTTGGAATCTAAGACCGAAAAAAACATATATTCTGAA
ATAGATAAAATTAATTTAACGGGAAATGAAAAAATATTGGCGAAAATGCTTCT
TAAGGAAAAAAAGGGCACAAATACAAGATGGAATGATTGCGAAAAATTGTTT
GCTCAGAGCATATATTACAGGTCCACTTCGACATATACGTTCTTAAGAGACTC
GCTGAAATTAAATTTTCCAAGTCCATCATCTTTGCAAAAGTGGAACAGCATAA
AAAAGTTACAGCCAGGAGACAACGAATGTTTGTACTCAGCCCTTAAAGAAG
CTATAAAAGAAATGAATGCATCTGATAAAGAGTGCATATTGGCTTGTGATGAA

GTAGCAATAAAAAAAAACCTAACTTACAATGTGTCAGTTGATATTATAGATGG
AATAGAGCATTTACTTGATCGCTCTAATAAAATCGGAAGCCATATTTGCGTATT
TGTTGTTCGGGGTATTTTAAAAAAATGGAAATTTATATTAAACTATTTCGTGGC
CGAAACGAATATAAAAGGCGATTGCTTAAAAAGTCTTATTTATAAAAACATTA
TTATTGCTGAAACAATTGGGTTCAAAGTAAGGGGTGTTGTGTACGATCAAGG
AGGTAACAATAGAAAATGTACCTCACTACTTGAAGTCACCAATGAGAAACCT
TACTTTACCTTAAATAATAAAAAATATTATATGTTTTATGACATTCCGCATCTTT
TCAAGTCTGTAAGGAATAATTTTCTAAAAGCTAATTTTGAAACCCCTGACGGA
CTAGTTGACTTTGATGTCATTCGAGAGGTTTACGAATTAGATCATGGATCAGT
AACGAGAATGACTAAATTAACAAGGAGTCACGTTAATCCAACTAGATTTGAG
CTAATGCGCGTATGTTTGGCAACTCAAACACTTAGCCACACTGTTGCAGCGG
CAATTAAGACTTGTAACCAAAACAAGCAATTACATCGAAATAGTTCCGAAGT
TGCAGCTTCTACGGCTGCATTTGTCCAAAAAGTTAATGATTACTTTGATTGCC
TGAATAGCAGAGTATTAACTGACAAAAACCCCATGAAATGCGCGCTCCAAGT
TAACAATGGAGTTTGGAATAAACTAAAGGAAATGCAGGAATACCTAAAGAGC
GTTAAATATCACGGGAATAAAATATATTGTGTTGATGGCTTGATCCAAACTACT
GAAGCTATATTTGGACTCGTCGAAGATCTCTTTAAGGATCACACAGACCACTT
TTTCTTTTTAACTAGTAGAGTTAACCAGGATCCCCTTGAAAATATATTTGCGTG
CGTTCGAGCAAAAGGTGGTAACTGCAGAAATCCTTCAGTTAACGAATTTAAT
ATAATAATTGCCAAGCTCATATCTCTACACATTTTTAAATTTTCACAAAAGTCG
AATTGCGAATCAGATGATGATGTTATGTTGCCAATAGAGTTCGATTCGATAATA
TATCAGCCTTTCATTGAAAAAAAAGAAATACAGCAACAAGAATACTCTGTATC
ATTTTCCAAAATTGTGCAAGACAATGAGAGGTACTTTGACCAAAATATAGATA
ACTTTTTGTGCAATGATGTACCCATTGAATTAACTTCCAGTAGATATTTTGTTG
GATATATTGCTAAGGGATCCAGTTGCGATAAATGCAGATCGGTAATTTTAAAA
GAAACCGAGCATTTAACAGCTCCATCAGAGCTTTTTATACATGAGAAAAACT
ACTCAATAGAATCGGACTTCGGGAAATTAAAGGCACCATCTGATTTATTTTTT
AATATTTGCAAAATACACATTAAAGCTTTCGAAAATATTTTTAAAAATAATAAA
AAACAAATGTGTATAAAAAAATTTATTGTTGAGCAATGCATTAAGTGCACAAA
TGAAAGTAGTGCCTTCTCATTATGGTTTTATGAAAATAATGAATGTTATGCACA
TAGAACAGATCTTTTAAACAAACTTATAAAAGTTTTGCTTTTTAAGCATTGTA
AGTGGACTGTGATAGCTGATAGACAAAAAAAGCAAGCTAAGCTCAGCATTCT
ATCTCATAAATAAAAAAAATGAGCGATATTTAAATAACTGACGACTGAAAAAT
CGAGCAATATATAATAAAAAACAATAGTTAGAAAAATAACTTAATTGGGGAAC
ATGGAAATGTTTTAATGTTAAACATTAAAATATTTCAAGTCGACTCGAAGGTC
ATATACGTAAATATAGGACCCCATTACAATTGTAATGGCCTCCCCGTGGTGTTC
CCTGGGTACCGATTATTACACATATTACACATAATTAATTATCAACATAAATATA
AATATGTAAACGGTAGCTAATTCGAGCGGCGATTTTAACAAACGAATTTAAAA
AGCTTTAAAATTAAAAAATTTTAAAATTAAAATTAAAATTTTAAAATTATAATA
ATCAGGGCGCGAATTTTTAAAAATTATTTTATTTCATCATATTGCTACGAAATT
GGCAAAAAACTACCCTAATATGTACAATGTAAATTCATTTCTTCGATCAGAATT
GATTTCGGCCCGAAAATCGTCTTCTAGCACAACACGCACACATATACGCGTTC
TCGTCTCTTGTTTTTACTCACACAAGCAAGCAAATTCTATTTTTAGATTTCTTA

CGTTCTCAGCGTGAGCGAGCGGAAAGAGAGCAATTTTGGCCGTCACCAAAA
AAGTGGCTGCATAGTGCCAAACCAATGTATGGCCGTTACGCATCTTGTTATTC
TAGTGTCTTTG

>POGON1Mariner-Tc1#DNA/POGON1Mariner-Tc1

CAGTATAATTCGCTTAGCTGCATCGATAGTTAGCTGCATCGGCAAGATATCTGC
ATTATTTTTCCATTTTTTTGTGTGAATAGAAAATAGAAAATAGAAAAAAAAAA
ATTAAGTTAGCTGCATTTTTAAGTTACCTGCATCGAGGCATTGTGCAAAGTAC
TCGAGGCAGCTAAGCGAATTATACTG

>POGOMariner-Tc1#DNA/POGOMariner-Tc1

CAGTATAATTCGCTTAGCTGCATCGATAGTTAGCTGCATCGGCAAGATATCTGC
ATTATTTTTCCATTTTTTTGTGTGAATAGAAAATTTGTACGAAAATTCATACGT
TTGCTGCATCGCAGATAACAGCCTTTTTAACTTAAGTGCATCATATCAGCTGTT
TTTTTTGCCAATTTCAATGAATATCATCAAAGTTAGCTGCGCCATCTATGAATC
ATTTTTGCATATCTAAAAGATGCAAGAATGCCAACTCGTTTCAGTATCTGCGC
ATGTCCGTTTTTGTTTTTGCTTTGATCGTGATTTTTGTGTTTTTGTTTCTTATGG
CACAAAGTTATTAAAATGGGTAAAACAAAGCGTGTCGTTGGACTAACACTAA
AGGAAAAGCTTCAAATAATCGAGTTAGTGACCAACAAAGTGGACAAAAAGG
AAATTTGTGCCAAGTTCAAATGCGACAGATCCACAGTCAACCGCATTTTACA
AAAAACAAATGAAATTCATGAAGCTGTGGCCGCGTCAGGTTTAAAAAGAAA
GCGTCAAAGAAAAGGAGCGCACGACTTAGTAGAAGAAGCCTTATACATTTGG
TTCGGACAGCAGGAATCAAAGAACGTAATTCTTGACCGGCACGTCATATTAG
CAAAAGCGAAAGAATTTTGCCAAAAATTTAACGACGCCTTTGAACCTGACGC
CAGCTGGCTTTGGCGCTGGCGCAAGCGCCACAATATAAAGTATGGCAAAATA
CACGGCGAAACTGCTACAAATGATTCCGTATCAGCAAATGAGTACAAAAATG
ATATTTTGCCAGGATTGCTTAAAGGTTATAACCCAGAAGACATTTTTAACGCT
GACGAAACTGCACTCTTTTATAAAGCAATGCCGAATGCGACATTTTTTACTTG
TGGAAAGCAATTAAATGGCCAGAAATCTCAGAGAGTGAGACTTACTTTGCTG
TTTATATGCAATGCAACTGGGACATACAAAAAAACTTTTGTAATCGGCAGATC
TAAATCGCCACGATGCTTCAAGAATGCTAATGTGCCCATTCCGTACTATGCAA
ATAAGAAGGCCTGGATGACTAAGGATCTCTGGCGAAAAATAATGACAGGATT
TGACGAAGAAATGAAAAAGCAAAATCGAAAGATTTTACTCTTCATCGACAAT
GCAACTAGTCACACGACTGTCAAGGACTTCGAAAACATAAAATTGTGCTTCA
TGCCACCAAACGCAACGGCTCTACTTCAACCTCTGGACCAAGGTATTATCCA
CTCATTCAAATTAGAGTATAGGCGTATTTTGGTCAAACAGCAGCTCATTGCTG
TTAATTGTGGTAAATCTACTGTGGAATTTTTAAAATCATTATCGTTATTGGATGC
TCTATATTTTGTCAACCAAGGATGGAAGAATGTTAAAATGTTAACTATTCAGA
ATTGTTTTAAAAAGGTAAGATGGGATTATTATTGATATGTATCTCAAATAACGA
ATTTATTATTTTCAGGCTGGATTTAAGTTCAGTTTTGAAAATGAAGACACCATT
GCTGAAAAAGACAAACAATGCGTAGAAGTTGACATTGTATCGAATATTAATTG
GAATGAATATGCCAATGTTGATGCAGATGAGGCTTGCCATGGTCAATTAGATG
ATGATGAAATCGTGCGCTCTTTAGTTCAAGATGCAAAAACCAGCGATAACGA

AGAAAGCCATAGTGATGAAGATGTGGACGATACTGAGCGTCCTACTTTTAAG
GATGGGTTTGCAGCAATTAAGGCTTTAAAGTCCATTTTTATGCGAAACAATAA
TGATGAGTTTTTGCAAAACTTGAATTCTATGGAAGACAAGCTGTTTAATTTAC
ATATAAACTCAGCTGTATTGCAAAAAAAAATTACTGACTATTTTTAAGTTAGTT
TTAAAAAGTGTTTTAATCAATTCACCATCACTTAAATTTATATGTCGATCTTACT
TATCATTAAGAATGAAATTATCAGTTCCTTTTATGTTTAACATTGTTATAAAGA
AATAAATTCTTTATTTTTCCTTAAAAAAAAAAATTAAGTTAGCTGCATTTTTAA
GTTACCTGCATCGAGGCATTGTGCAAAGTACTCGAGGCAGCTAAGCGAATTA
TACTG

>PLACW_P-element#Unknown/PLACW_P-element

CTTAAAAGTGCGTTTGAACATTCTCGTTGTGGGCGTGTGTGAATTTAGTACGC
TCCTTCCTGGTTTAAATCATTTTCGCACTAAACTTCTGCTCTCAGCGGAATTTA
CTTTTGCTTTATTAGAGATGGGAGCTCGCGCATCAGCTGAGCCGATACTTGCG
CAACAGGTGAGACAGCTGATTAGAGATGGCCCTTTTCAACTGTTCCCAGCAG
TGACCGCTGCCATAACCGTTTTTCAAATTTACGTGAGAACAGACATAAAATAA
ATATTACAGCTCGTAGTAAATGTTATTCTATATTTAAAAGGAAATTGTAATAGTT
AAAACTTGCAATGAATCAGTTACGTTCAAAAAAGGAAACACACTTTAGTTTT
TGGCTAGTTTATTGGGTTAATAATATTTTATTTAAAATAGTTCGAGTGTTCAATA
TAGTCATGTAAATGTGTACAAAAGATCCGGCATTTTATATTTAATATATCGATTT
CCCTTCACTTTCGCTCCTCGTATACCATGCTGGCGTCTTATCAAATTAAATAAC
ACAATATTTAAAAACTAACAATGCAGAGCGTGCTAATCGTGTATGTTTGCATT
AAAACGTTGCGAACTTATCTCCTCTTGCGCTTTTTCCTTATTTTTGCAAATTAA
ACAGAAAAAGATATTGAAAGGATATGTATGCATAAGGATGCCGATGGCACTTG
TAAATATTTAAATACAATAAAACTGTGCTTATAGTTCAACTAAATTCGTTATTG
CATTTTTTAAATATATATCTAAGTATCTAAATATCGCGAGAAGACACTATCCAA
AAGAGATTACAAAAGCTTTTCAGTTTAATTTGCGTGTAATGTTCTGCTGCTTT
ATAGTAAACTGCTTTATATCAGTCTTCTTCAATTTGTTGTTGTTCGTGAGAATA
AATTGGCATATAAATGGAA

>PEN4PEN4#Unknown/PEN4PEN4

GGCGGACGTGGCGCAAAGGGCAGCGGAGCCGGCGGCGTTGGTGCCAGCAA
TAATGCCGCTGCGGGCAATGCGGCTGGTGGCGCAGCGGGAGCTGCAGCGGG
CGCTGGATCTGTTGGAGGTGTTGGCGGTGCAGGAGCAGCGGGCGGCGGC

>PEN2PEN2#Unknown/PEN2PEN2

GAGGTGGTGGAGGCGGCGGCTACGGCGGCGGTGGTGGCGGAGGAGGCTAC
GACCGAGGAAATGATCGTGGATCCGGCGGCGGTGGATATCACAACAGAGATC
GCGGTGGCAACTCGCAGGGAGGCGGAGGCGGCGGCGGCGGTGGTGGTGGC
TACTCCCGCTTCAATGACAACAATGGCGGAGGACGCGGTGGCCGTGGAGGT
GGTGGCGGCA

>PEN1PEN1#Unknown/PEN1PEN1

GGAGGTGGCGGAGGACGCGGTGGTCCGGGAGGTCGTGCCGGTGGAAACCG
CGGAAACATGGGCGGTGGAAACTACGGCAACCAGAATGGTGGCGGCAACTG
GAACAACGGTGGCAACAACTGGGGCAACAACCGCGGGGGTAACGACAACT
GGGGCAACAACAGCTTCGGTGGTGGCGGCGGCGGCGGTGGTGGCTATGGCG
GTGGCAACAACAGCTGGGGCAATAACAATCCGTGGGACAATGGCAATGGAG
GCGGCAACTTTGGAGGCGGCGGCAACAATTGGAACAATGGTGGCAATGATTT
TGGAGGCTACCAGCAGAACTATGGCGGCGGTCCGCAGCGAGGTGGCGGCAA
CTTCAACAACAATCGCATGCAACCCTACCAAGGAGGTGGTGGATTCAAAGCA
GGCGGTGGCAATCAAGGCAACTATGGCGGAAACAATCAGGGCTTCAATAAC
GGTGGC

>NOMAD_LTRGypsy#LTR/NOMAD_LTRGypsy

AGTTAAGAACCCTCTTCTTGCGCTCTTCGTCAGGACTCACCAGCGCTCGGCT
CTCGTGTTTTCGGGCCCCGTCAGCAGGCGACTCGGGGCCTGTCTAGTAACAT
GTTCGTGTAAGTTACGAACCCTCTTCTTGCGATCTTCGTCAGGACTCACCAGC
GCTCGGCTCTCGTGTTTTCGGGCCCCGTCAGCAGGCGACTCGGGGCCTGTCT
AGGAACATGTTTGTGTATGTGTGCATTCGGAACAAGTGCCGTTGGTCGCACT
CAGGGTGAGGGGTCAACGGGGGAAGCGGATATAAAAGCAGCGGGGCGGGA
GAAGAGGCCCCAGTGTCGAACGGACACATAACGGAACCGCTAGCAGATCGC
GAACTGAATCTTAAAATAAAGCTAATCGTAAACTCGAACCCTCTTAACTATCT
TGACTATTATTTGGAGAACCACAGCATGTTGGTTGTCATATCAAGGTGAGGTA
TGCGGCAGCGAGTGCCGAGAACCCTGATGCAAGTGGAACTTGCGTTAACT

>NOMAD_IGypsy#LTR/NOMAD_IGypsy

GGCGCCCGAACAGGGACCGGCAATGTCCGGCCGATAAAAGTGATACGAAAA
AATTGTGGAAATTTGTGCGTAAAAATAGTGGTGGTGTGCATAAGTCAGATTAA
GATCTGAAATCCATAAATGAAAAAGAAGTGCTGCGTGAGCTGTGTATAAAAT
GATAAAATAGCAATTACCCGCTGCCGGGGGGAACTACGCCCATCCCGGGGCG
CAACAAATATTGCATAATTCAATAAAAGGTGTAAAATTTCTAAAATAAAAATG
TAAACCTATGTTGCGCCAAGACCTAATTTAAATTAATAAAACAACGACCCGCT
ACCGGAGGACGCCACGTCGCCCATGCCGAGCGCAAAAGTTGTACGATACCTA
TAACATAATTAAAACACGATCAACCCACTGCGGCGGTACGGCTTGTGGGAAA
ATTTTTTTTTTTTTCTCTCCTTGCCAATTCGCGAGTGCAAAAGATTGTGTATAA
TAAACCAATAATTAACCATTGCAGCAGTTTACCTGCGGCAGTACGAGTAATAT
GAGCGCCCAGAGTGATAAGGTGGTGTGTGGCAGCTTGTTGGATACGTTAAGT
GGTGTGGAATGCACCCAAAAAAAACCGCCCAACAAGTTGTGTGGCGGCCGT
ACCTTAGTAGGCAACCAGCCAAAAGGGATACTACGGAACCACCGTGCCCAG
TGCCGAAATAAATTAGAGGTCATCAATAAAAAACTGTAACAGCACGCACGCA
AGGAAAAAATATTGCAAAATGGAATAGCGCACAAAAATTGTATAAACACATG
CACAACACCACAATTCAAAGGAAAACAAGTATAGTACAATTTCCCCAATTTC
CCGAAACGAAATGAATAAAAATTCTCTACAATACACTAATTCACCTTTTTTA
CTTATCCCAATAGAATATTCATGCTGTAGGGGTACAACCTAAACGACGAAAAC
TAATAAAGAGCATACAAGGGTGAGTGAAATATTTCATTAAACTTTATTGCCAT

ATTTGCTAAATTTAGAGAAATAAAGAAAAAGCAAAGAAGAACAGATATTCTT
TTTTATCGGGTTAAAACCGTTGTCTCACATTTCCGTAAAGTAATAACGAATTCT
GTTGCCTTGAAAGCTTCCTGCATCTTTCCAACGCAAACTAAAAATCAAAATG
GAAGAGACCCTGCGTGCTCTTAGCGAGTCCCTCAATGCCCTGACCAACGTGG
TGACAGGCATTAAGGAAGATATTAAGAAAAATAATGATAGGTTGGCTATTTTA
GAACAGGAGCGCGGGAACGCTGACCCTACGGTCGACCAACCGCAACCCCTG
GTGCGCGCACGCACCGAGTATGAGCTGAGAGAGATATCGGTCCTCCCTGACT
GCGTCAAAGAACTGCAGGCGTTCGAAGGACGGCAGGAGGCTTACCTGTCTT
GGATAAACAGGGCACAGTCAATACTGACCGAATATGACTTGATTAAAACCAG
ACCCCTGTATAGGGCAATTGTCTTGCATATTAGACAGAAAATAAGGGGACACG
CCGACATGGCCTTGGCGGCCTATGGCGTCCAAGACGACGATTGGGACGACAT
AAAACGAGTCTTGGCGCTGCATTACGCAGACAAACGAGACTTACGTACGCTT
GAGCATGAGCTTGGCGCTATGTGCCAAGGTTCTAGACCACTAGATAGGTTCTA
TATGGACGTTAATGGCCATCTCTCGTTGATCTTAAATAACTTGAAGGCCAGAA
ACCACCCTCGTGAAGTAGTCAACGCTTTGATAGAAACCTATAGAGACAAGGC
TTTGGATGTTTTTATCCGAGGAGTGGGGAGAGATTGTTCCAAACACTTACTTG
TCCGCAGCCCGAAGAATCTACCAGAGGCTTACTCTTTTTGTATGGGATTGCAG
AATGTAATGTCAAGAAATTTCACAGCTCAGAACTATCAACCGTCAGGTGCCC
CAAGATTCGCAGGCCCATATCAACATCAGGCCAGGCCACCGTTCCGAACCCC
TTTTTCTCCTGGTTCAGGCAGATTTTCGCAAAACTCCTACAGAACTCAGGGTC
CTAGACAGGCCATAAAAATGGAATCCAATCGGTCGGGTCAATCTTACCAATCA
GGATACAGTGGTCGCCAGGAAGAAGGCTCCGGTATTAAGAGAATGTCCGAA
GGAAACAACCCATTCCAAAAGGCACAAAGATTGTACCACATGGAATTGGCAC
CACCCCCGCTAGCCCCGGCGGCTAGTGGAGATAACCAAGGACGTTCACACG
AGGGTTACTATGATGACGAGTCTCAAGCTGTCGAGAGAAGCAACAATTATCC
TCCGCAGAAAAACGTGGAAGGAGTTACAGATGCTCCACATAACCTTGAGACT
GAGGGAGGGGCAAATTTTATGACCAACGCCTCTCCAGTGTACCGTACTTAGA
GTATGCTACGGAGAGGGGAGAAAGGCTGAAGTTTTTGATCGACACGGGGGC
GAACAAAAACTTTATTAGCCGAAGACTTGCAGCCGGGTGTACCACAGTCCGT
AAACCCTTCTCCGTACTGTCCGCTGCGGGTAACATCATGATAACGCACCGCCT
AGTTGGTAAATTCTTCAAACCACTAGGGAACGACTCGGATATTACCTTTTTCG
TACTACCGAATTTACATTCCTTTGATGGTATCATTGGCGACGATACTCTCAAAG
ACTTAAAAGCCATAGTGGATAGGAAAAACAATTGTTTGATAATAACCCCAGG
AATTAAAATCCCTCTTTTGGCGAGAGCTTCAATAAACGTTAACCCGCTACTCG
CCGCCGAACACCCAGATGGTACACAAGAAATTTTGAATTCCCTTCTCGGGGA
ATTTCCCCGCATCTTCGAGCCCCCCTTATCTGGAATGTCCGTGGAGACGGCCG
TCAAGGCTGAAATCCGGACAAACACACAAGACCCGATCTATGCTAAAAGTTA
TCCTTACCCAGTCAACATGCGCGGAGAAGTCGAACGTCAAATCGATGAACTG
CTGCAGGACGGTATAATTCGACCCTCTAATAGCCCTTACAATTCCCCTATCTGG
ATAGTCCCGAAGAAACCTAAACCAAACGGAGAAAAACAATATCGCATGGTAG
TCGATTTCAAGCGGTTAAATACCGTCACCATACCCGACACTTACCCCATCCCA
GATATAAACGCTACGCTAGCCAGCCTTGGCAATGCCAAATACTTTACCACCCT
AGATTTGACTTCTGGATTCCATCAAATCCACATGAAGGAAAGCGACATTCCA

AAGACAGCTTTCTCTACTCTAAATGGAAAGTACGAGTTCCTCCGTCTACCATT
CGGTTTGAAGAATGCACCTGCAATCTTCCAAAGAATGATCGATGATATTTTGC
GCGAGCATATTGGCAAGGTCTGCTACGTTTATATTGACGATATCATCGTCTTCA
GTGAAGATTATGACACACACTGGAAAAATCTCCGATTGGTATTAGCGAGTTTA
TCAAAAGCTAACCTCCAAGTGAACCTTGAGAAGTCGCATTTTTTAGACACGC
AGGTAGAATTTTTAGGATATATCGTCACGGCCGATGGCATTAAGGCAGATCCG
AAAAAGGTCAGAGCGATTAGCGAAATGCCTCCTCCGACCTCTGTTAAGGAGT
TAAAAAGATTTCTAGGCATGACCTCGTACTACAGGAAGTTCATTCAGGACTAT
GCGAAGGTAGCAAAGCCCCTTACAAACTTGACGCGTGGATTGTACGCTAATA
TAAAGTCTTCACAATCAAGCAAAGTGCCAATTACATTAGACGAGACGGCCCT
ACAGTCTTTTAATGATTTAAAATCAATTCTCTGTTCTTCTGAAATACTGGCGTT
CCCATGTTTCACTAAACCTTTCCATCTAACCACGGACGCTTCTAACTGGGCCA
TCGGAGCTGTCCTCTCACAGGACGACCAGGGTAGAGATAGGCCGATAGCGTA
CATTTCCCGTTCATTAAATAAGACGGAGGAAAACTACGCTACTATCGAAAAG
GAAATGCTCGCGATAATTTGGTCATTGGACAATCTTCGGGCTTACTTATATGGC
GCTGGTACTATTAAAGTATATACTGACCATCAACCTCTAACGTTTGCCCTAGGC
AACAGAAATTTCAATGCGAAGCTAAAACGCTGGAAGGCTCGTATAGAGGAAT
ACAACTGCGAACTCATCTACAAGCCTGGGAAATCTAATGTGGTGGCTGACGC
GCTTTCACGCATTCCGCCTCAGCTTAACCAGTTGAGCACCGATTTAGATGCTA
ATCCCGAGGATGACATGCAGTCTTTGGCTACTGCCCATAGCGCTTTACATGAC
AGTTCACGATTGATTCCCCACGTTGAATCTCCAATCAACGTTTTCAAGAATCA
ACTCATTTTTGACACAACCAGGTCAAAATACTTATGCGAGCACCCGTTCCCA
GGTTATACTCGCCATCTGATTCCTCTCAAAGACGGATCACTTGCCGATTTAAC
CAACTCGTTACAATCGTGTCTACGACCTGTAATAATTAACGGCGTCAAAATCC
CGGAAGCACATTTGCAACGCTTTCAGTCCATCTGCTTAGCGAATTTTCTTTTA
TACAAAATTCGGATAACGCAGCGCCTAGTGGCGGACGTGTCTGGCGCAGAGG
AAATTTGTGAAATAATTGAAAAAGAACACCGTAGAGCACATAGGGGCCCTAC
GGAGATTCGTCTCCAACTTTTAGAAAAATATTATTTCCCGCGAATGTCCAGTA
CGATCCGTCTGCAAACTTCCTCATGTCAGTGTTGCAAACTCTACAAGTACGA
GAGACACCCTAACAAACCAAACCTACAACCTACGCCAATTCCTAACTACCCA
TGTGAAATACTTCACATCGACATTTTTGCGCTCGAAAAAAGGTTATACCTAAG
TTGTATTGACAAATTTAGCAAGTTTGCCAAACTTTTCCATCTGCAGTCAAAAG
CATCTGTGCATTTGCGAGAAACTTTGGTGGAGGCCCTACATTACTTCACCGCC
CCTAAGGTCTTGGTTTCGGATAACGAGCGAGGGTTGTTATGCCCCACAGTGC
TCAACTATCTTCGGTCTCTAGATATCGATCTGTATTATGCTCCAACCCAGAAGA
GCGAAGTAAATGGTCAAGTCGAGAGATTCCACTCTACGTTCCTAGAAATTTAT
CGTTGCCTTAAAGATGAGCTCCCTACCTTCAAACCCGTTGAGCTGGTACACAT
AGCAGTGGACCGCTACAACACTTCCGTTCACTCGGTAACGAATCGAAAACCA
GCAGACGTTTTTTCGACCGCTCGTCAAGGGTAAACTATCAGGGTCTGACAG
ATTTCCGGCGGCAGACTTTAGAGGACATCAAGGGCTTAATTGAGTATAAGCA
AATTAGAGGTAATATGGCTCGGAATAAAAATAGGGACGAGCCAAAGTCTTAT
GGGCCGGGAGATGAAGTTTTTGTTGCAAATAAGCAAATAAAAACAAAGGAA
AAAGCGAGGTTCAGATGCGAAAAGGTACAGGAAGACAACAAGGTAACAGTT

AAAACCAGATCAGGAAAAATTTTCCACAAATCTGATCTAAGAAATTGAGACG
TGGCTTTCACATTTAAAAAAGAAACGCGAAAAAGAATAACGAAAGTAATAA
AAGTACGTTGTGGCAGCTAATGAAATATTCCACCCATGCATACCCTATATAAA
AAAAACATTAATAAAAAAAAAAAAAAAAAAAAAAAAAAAAAAAAAAATGAGT
TAAGAAATACAAAAAGAAATACAAAAAAAACTATAAAAAAAATAATATAAAA
AAATACAGATTATAAGAAATAAGAAATAAGAAATATAAAAAAATAAAAATATA
AGTACACAAAATGTACCGTACCCCCACACACTACGTAGTCTTAGAACAACTT
AGACGACCAGATATTTACGAATTGTCTTTTTGTAAGCGCGATTTCTGCATGCG
GCGCAAATCCCGCTCACTGGACTGGCTGGGGTCGGCTTGGAAATGGGTAGCT
GGATCTCCAGATGCTGCTGATTGGAACGCCGTCTTGGCCGCGCAAGCGACGG
CTTCGAGGAACTGCAAAAACTGGAGGAGGCTAGCTGTATCCCTCGGCTACTG
AAGTAACCAACGAGTGGTTAAGCAAGTCGACGATGGAATGCTCCTCCTGACC
AACTTCAACGGAACTCTAAGAACGGCTGCAGAGAACTACGACCTGATCGGC
TCCTTTATCATCCAATTCGACAATGAGACGATAATGGTCAACGGTCAAAACTA
TTCCAGTTACTCGGTCAGTCATCTAATGGCGATGCCGGCCGTGTTGAGCCACA
TAACGGCCAGCAACTTTCAACTTTCTCTGGAATACGTCCACGACGTGAGCAT
GAAGAATTTGGAAAAGATGTCCAACATGGCGAGTGAGCTACTAGCCTCTCTT
CTCACCGAGGCGGCACTCGCAATCTGCATATTCCTAGGCTTTTATTTCCTATGG
AAGAAGCTGATGTCCACCAAAGGCATGCCCGATGTCCGCGAGATTGCCGCAA
ACTTAGAAGCATTGGGCCAAACCGAGCTGAACAAGGCTCACTAATCTGCGG
GACGCAGATCTTGAGGGGGGAGG

>MDG3_Gypsy#LTR/MDG3_Gypsy

AATCTCAGAAGTGGGATTAATAATCCAAAATGGACGATAAAATCATCCTGAAC
GACTTTTCGCTGACAACCCTAAAAGATTGGCTACGTATTCTGGGCCAAAATAC
GGAGGGCACAAAAACCGAATTAATCGCGAGGCTGCAAGACATCCCAACGGC
AGTTCGGGGCGATTGTCCACCGGAGCACCCCCAGAAAAACGCTCCACCAGG
AAACGACATTTTTCTTCACTGGATTTTCAGAATTGTGAAATTAACACCGATC
ACGTAAGTGTGAATGCGATGAACAGAAAAGAATCAACCGAAACTGGCAGTG
AGAGGGAGACAAACATGTTCGAGCTACAGCAACTACGCGCAGAGCTAGCAG
AAGCGAAGGCAATGCTTAACGGAACACGATCGAGCTTGCAGTTCCAAGAAC
AACAACAACCAGAGCAAAGCAAGGCTACAGTTAGTTCCGTTATCCAGACGG
CGCAGTTTACGCAGGCTGGCGCCACAAAAGAGAACACAACATTTCACTCGC
CGCAGCGATCCAACGAGAGAGCGGAGAGCCAGCGTTTTCCAGTTGATGCTC
TCGCTCTCGCCAAAGAGACGATAACCGATTACGATGGGAAAACTTGCGCGCG
TGCCTGGATAACAGTGGTCAAAAATATCGCACGCACTTTCAACATCGATGAC
AACCATTTACGCATCTTACTCATCACTAAACTTAAAGGAAACGCGCAAGTCTG
GTTACATGCGCACCCTGCTCGATTGATCGAACCAATTGACAATTTGCTTGATC
AATTGTCATTGACTTTTGGCGAGCAATCATCCAAGGCTGAGATCCGGCGAAA
ATTCGAGAGTCGCAAGTGGAAAACCGAGGAGAATTTCTGCAGTTATTACGAC
GAGAAGATGGCTCTCTCAAACGGGATAAACATCGACGACGACGAACTACTG
GACCAGATGATAGAGGGCATACCGCTACAAAATTTCCGTACCCAAGCACGGA
TTCAATGCTTCTCTACTCCATCGGAGATGCTACGCGCATTTTCGAACATCCGTT

TGCCAGCTCGGAGGGAGCCACCTGTACAGCCAACCGACTACAAAGATGCCA
TACGATGCGCAAACTGTAATTCAAGAGGACACAAAGCTGACATCTGCAAGA
AGCCCAAACGTGAACCAGGTTCGTGCTACGCCTGTGGACAACTTGGACACC
TGGTGGCACAATGTCCCACAAGGAAGAGCGTTTCATCTAATAATTATGTAAGA
TGGTTTAAAATTAATTTTTTTGAAAATGCTTATAAGCCCATAATTTCAGAATGC
CTCATAGACTCTGGCAGTCCTATATCTATCATTAAAAAGTCACTTATTAACGAG
ACAATGAAGTTAGCCCTAGTTAATACTTGCTATTTTGGTTTAAACAACTGTATT
CTCAAAACACATGGACAAACCACATGTTATGTTTTGAAAGGATCAATAAAAA
TATATTTTCGTTTAATCATTGTTTGCGACCAGTCTATGAGGTATAATGTTATTCT
CGGCAGAGATTTTTTAACTGCATGCAATTTAAATTTAGACCCGTACACCTTGG
GAATGATTGCGTTGAGAAAACCCATGGAAATAAACAAAATATCAATGTTTACT
GAAAATGATAGTCCTGAGAAATCTTTAGAAAATGAAATTGTTAGTCCAAAATC
GTTAGAGAATGAAATTGTTAGTTCACAATCGTTAGAAAATGAAATTGTTAGCC
CCAAATCGTTAGAGAATGAAATTGTTAGTCCAAAATCGTTTAAAAATGCAACT
ATTAGTCCGAAATCGTTAGAAAATAAAATCGTTAATCAACAGCATAAAGAAAC
TGGTCCAATATCGTTAAGAGATGAAATAGTTAATCAACAAAAGAATGTCAGTA
AATCAAAATTATCAGAAGATGAAATTGTTAACACTTCAAAAGAAATCGTTAGT
TTTAAATTGCCAAAAGATAAAAACGTTTACGAACAATTAAATCACAACTTTGA
TAAGGAAGTACTAAGAATATGTCATGTAACTGAAAGTGAGTTAGAATACAAA
ATAGGAGAAAATGTTAGCAATAGGTTACAACTAGAATTCGATAGGTTGTTTAG
AAATTTTTATATAAATGCAAAAAGGCCAAATGAACCGACAGTTAGAAGTGAA
ATACAATTGTGTTTGAAAAACCCGAAACCGTTTAGCTGTTCTCCTAGGAGGCT
TTCATACACAGAAAAAGACAGGTTACAAAAACTATTAGACGAATATTTGGAA
AACGGATTTATACGACCAAGCGACTCGGAATATGCATCGCCTATTGTTTTAGT
GAAAAAGAAAACTGGAGACTTACGTATGTGCGTCGACTTTAGAAAACTTAAT
AAAATGACAATGAAAGACAACTATCCTCTACCTCTTATAGATGACTTGTTAGA
TAGAATGAATGAGAAAACTGTTTTCACCAAACTCGATCTTAAAAACGGTTTT
TTCCACGTGCATGTTAAAAAAGAATCAATAAAATACACCTCTTTCGTTACACC
ATTAGGCCAATACGAGTGGCTGCGAATGCCATTTGGCCTCAAAAACGCCCCG
TCTGTGTTCCAAAGATTTGTTAACAAAATTTTTGCGGATATGATTAGAGAAAA
CAAAGTAGTAGTATATATGGACGACATTCTATTGGCAACCGAAAATATAAACG
AACACTTAGAAACGTTGAAAGAAATTTTTAAAAGATTAGTTGAAAATAAACT
TGAATTAAGAATAGACAAATGTGAGTTTATGCAATCAAGTATAAAATATCTTG
GGTTCATAATAAATAAAGACGGCATAATGCCCAATGACAAAGGAATCGAGGC
AATAAAAAATTTCCCAATACCTAATAATGTTCATACAGTACAAAGTTTTTTGGG
ATTATGCTCATATTTTCGACGGTTTATAAAAGATTTTTCTAGACTAGCTAAACC
ATTGCATGACATTCTAAAAAAAGATAAACCGTTCAAATTTGGTAGTGAAGAA
ATGATTTGTTTTAATATGTTAAAAGATAAATTAATACAGTCACCGGTCTTAGCT
ATATACAACCATAAACACGAAACAGAATTGCATTGTGATGCAAGTTCTTCTGG
ATTCGGTGCTGTACTTATGCAAAAGAAGGAGGACCAGAAATGGCACCCAGTT
TCATTCTTTTCAAAACGGACAACAGATATTGAATCAAAATACCACAGTTTCGA
GTTAGAAACTTTAGCCATTGTTTATTCGTTACGTAGATTTAGAGTTTATCTTCA
TTGGAGGACATTTAAAATAGTCACCGACTGCAACTCATTAATTTTGACCCTAA

GCAAAAAAGAGCTAAACCCTAGGATAGCCAGGTGGGCTTTAGAATTCCAAG
GTTATGATTTTGAAATTGTGCATAGGGCAGGTAGCCGCATGCAACATGTTGAC
GCACTGAGTAGGTGTACAAATATTATGGTAATACAAACAAACAGTTTCGAAG
ATAATCTAGTTATATGTCAAGGGAAAGATACAAAATTAAAAGAAATCAGGCAA
TTGTTAGAAAACACAGAAAATAAATTGTATGAGATGAGAAATGGTATAGTTTA
CAAAAAGACAAATGAAAATAGATTGCTGTTCTACGTTCCGATAGAAATGGAA
GAACAAGTGTTATACAAATATCACAACGAACTTGGACACGTAGGAAGAGACA
AAATGATAGAAGCTATAATGAAAAACTATTGGTTTCCAAATTTAAAACAGAAG
TGTAGCACACATATCAGCAACTGTTTAAAATGTATTTCATTCAGTCCCAAAAC
AGGAAAAACAGAAGGATTTCTACACAACATACCTAAGGGAAACAAACCTTTT
GAAATAATCCATATTGACCATTATGGTCCAGTAGACTTGGCTAGACCGAAGAA
ACATATTCTAGTGATAGTAGATGCATTCACAAAGTTTGTCAGACTATACGCAA
CAAAAACTACGAACACAAAAGAAGTCATACAATCGTTAAATGACTACTTCAG
AGCATACAGTAGGCCTAAGTGTATCATATCAGATAGAGGAGCATGTTTCACGT
CTGGCGATTTTGACTCATTTTTGAAAGAATGCAATGTTAAACACATAAAAATT
GCAACAGGATCGCCACAAGCCAACGGTCAAGTTGAACGTATAAACCGAAGT
CTTGGTCCAATGATTAGCAAGTTAATTGAACCTGATCAAGGTCTACACTGGGA
CTTAGTCTTAGAAAAGGTCGAATATACCCTGAACAATACACTACACCGCAGC
ATTAAACAGTATCCTAGCATAATGTTATTTGGGTTACAACAAAAAGGACAAAT
TATGGATGAGTTAAAAGAAAAAATTGAGGAAATTGGAGAAACGATTGAAGA
AAGAGATTTAGAAAGTATTAGAAATAAAGGCGAGGCAAGTCAGAAAATAGC
ACAAGCATACAATAAAGAATATGTTGACAAAAAACGAAAACGATCAGGAGT
GTTCACAAAAGGCACTACGTCATGGTTAAAAATTTTGACTCAACAACAGGCA
TAGCTAAGAAGTTAATTCCAAAGCATAAAGGACCCTATGTCATAAGCAAAGTT
CTCAAAAATGATCGCTTCCTTCTGGAAGATGTTGATGGATTTCAAATTTCTCG
CAATCCTTACCGGGGTGTATGGAGCATACAGAATATAAAACACTGGCAAAGA
AAAATTAAGAGTCTACAAAATAGAAAGTATAATTTGAGAAACTCTGTACAAA
ATAGAAAGTATAATTTGAGAAACTCTGTACAAAATAGAAAGTATAATTTAAGA
AACTCTGTACGAAATCGAAAGTATAATTTAAGAAGCAATTGTAAAACAAAGA
AAACAAACAAGAAGAAAAGAAAACCAAAAAAATGTTTAAGACCGTTCAAA
AGTATCTCCACTAAGAAGAATAAAATAAGAAACAGGACCCTTAGCTTTAAGA
AACGTTAATTGTTATAAAATCCTACGATCGGGAGATCTAGTTGTCAGGACGGC
CGAGT

>MDG1_LTRGypsy#LTR/MDG1_LTRGypsy

TGTAGTATATACGAATATAATAACAATAATAATAACAATAATAATAATAATATTA
ATAATAATTATAATATGAATCATAATAATAAGTCAACTAATAAGTAAACTTAGGA
CCACCCTAATTCCTTAGGGTCACCCTAGTAGATCTTTAGATACACCCTAATACT
AAATATGCGAATTCAGGATGTACGCCTTTAGGGGTCGGACTCGACTCCCATTG
GTTATCGAGTATGAACTTCATACATACATATTGCAGAATTTGCTAGTGTCAGCA
CTTGGCTGTCACAAGAGATCTCCCTGTAGACCACACTAAGATCAGTTATAAAT
CAGGAATAGATCTGGAATGTACACTCGCTTAATAAAAACCAAATAAAGATAA

AATGACCAACTGCGTTTTGAGACTTTATTAACTACATCAGAAGTATTAGAATT
CAAATTAACTACA

>MDG1_IGypsy#LTR/MDG1_IGypsy

TGGCGACCGTGACAAAGGATCGTTATAAGTTGTAGCAGAAGCTAAAGGAAA
CCGCTTGTGATAATTTTCAACTTCGATGCTCATCCACCAAGACGGCGGCAATT
ATGAAGAAAAAAGCGATCTGAGTGAGTAGAGTGTCAGTGTGATGGGAAAAA
ACAGGGGCGGAGTTCGACATAATATAAAAAAGAGAATAGCGCACATAAAGTG
GCTATTATATACGAACACTCCACCACCCCAATGGTCGAAAGCTCAAAAACTAC
AAGCTGAGCTAGACCACTGTGTCGAATATCTCAAGAAAAAAATCCCCACCAC
ACGCGCTCACTCAGAAAATCAAATAAAATCGTTAACAATTAACAAAACTCCA
ACTCCCAATCCGAAAAGCCTGCCTGTTTCAAGAAAAGATGCCCGAACGACT
GCGAGGGACCACTGTTCACACCGCATTGTGAACATACGTGCAGACATTGCAG
CTCCACCACATAACCCCTAAATGAGGAAATCATCATCAACGTGGTGAGCAGC
CCGCTCATTACGTCATCGAGGGAGTGTCAGCGTGCCAACCCGGCGACGACCA
GATGACGCAGGAGGGTCAGAGTGAAGCAAATAGGAGCTGAAAAATAAAATA
TTTTTTTTGTTGCCCTGCGTGGCACACCCTCGATGCACTGCGCTGCATATTAAT
ATTACACAAAATATTGTAACATTGAGCGGAACTTTTTCTGCCCGATGAGAAGA
ATGGCCCGTAAAGCCATACACCAACTAGGTAGGAAAATGTAACTATATTGAAC
AAAAAAAAAAAAAATCAAAACAACATATTTTTAAAGTAAAATAAACCAAAA
CCCAAAAATAAAAAAAAAAAAAAAAAAAAAAAAAATAAAAATATACAAAAA
TGGGTTGGTTTGGATCTGACGATAGTCAGACAAAAGATAATACGGCCAATGT
GGTCAATAACTTAAAAATAGTCGACCATACAGATGACATTCAGTCACTGTGGT
TACTCCTTTTGATCATGACGATCGTAACAGTCGCTCAGTTTATATTAACGCTAT
ATGTTAAGCATAACAAGATAATAAAGAGGCGTTATATAGGCAAAGCAGAGAA
TAGTTTGGATAAAATTTGAAAAAAAAAAAAAAAAAAAAAAAAAAAAAAAAC
ATCCGAGATGTATTTTGAATTAAGATGATCTAAAATTTTTATTTTTAATATCAGA
AAACTAGAATGAAAAAAAAAAAAAAAGATTATTGAAGAAACCTATTTGAGA
GAGGCCAAAATTTATAAAGTTCGATTGCATAGATAAATCCATAGTATTCTTTAA
ATGGACTGGCAAGAAATACAAAACGAGCTTAAAGAAATTAAAACAACTTTTG
ACAAGTCTTATAAATGCATGACACCAAATAGAGAAGTGCAACAAGACACTCT
CAACAAGCATGCGCAGATATTGGTAAGATGCTTTAATGGAGCACGCCAATTAA
TTTACAGAGAAAGGAAAAGATTAACAAAAAATCATTTATCACAAGCAGTAAA
ATTTCTAAACAGGTTCCGTGAGAACTTGTTAAACGTCAAGTACAGACACAAC
TTAAATATTACAATCCCAACGATTTTAAGCACACCTATAGTGGCTGAGATCGG
TGAGGATATCGAAAGTGTAGGAGAATCAGAAATAGAAATAAAAGAAGAGGA
TCTCCACGATCTTGCAATTCCAGCGGTAATAACATTACCCGAATTACTTGAAG
AAGAACTTTCAGATTCAAATACAGGAATAAGAATACAGGAAACGGACAAAAT
GACAGACTCTGCCGCAACAGCAAGGGAATATGTGCGACAAATTTCGTCCACA
ATACCTGAGTTTGACGGCAAAAAGTTAAACTTGAATAGATTCCTCACGGCTCT
CCGGCTGATAGATCTGACAAAAGGAGATCAGGAGATGCTAGCGGTTGAGGTA
ATCAAGACAAAGATACTTGGTCCATTATCACACAAAGTTGAAAATGAAAAGA
CCATTATCGGTATAATAAATCTATTAAAAGCATCAGTTAAAGGCGAATCGCCCG

ATGTCATCAAAGCAAAAATGCTTAGTACACAACAGCGCGGCAAAACTGCAG
CGCAATATACCACGGAGATAGAAAACCTACGTGGGTTGCTCGAAGCAGCCTA
TATAGATGATGGTTTAGATTCCAACAATGCAGACAAATTCGCTACAAAGGAAG
CCATATCTGCAATGACCAAGAACTGTGGGCACGATAAGCTCAAAACCATATTG
GAAGCTGGAAATTTCAACACGATGAATAGCGTGATTGAAAAATACATACACT
GCAGTACAGAAATGACCGGCAATTCAAATAGTGTATTATTCTATAATAATAGAG
GACACTATCGAGGTAATAATTACCGAGGAAATTACCAAAACAGAGGTAATGG
CCGAGGAAATTATAACTCCTACAATAACAACTATAGAGGCAGAGGTGGTTAC
CATGGTGGAAACAGAGGACGAGGTGGTAACCAAAATTATAATAGAGGTGGA
GGTTACTCAAGAGGTAACCAAAACCATAACTATAAAACAAGTCATGCCCACA
ATGTCCGAAACATACAATCGGAAAACGAACATACCCCCTTGAGCGACAATCT
ACAATAAAATTATACAAAATTAATCTCAATTTAAGCATTTTTATACGATTGAAG
AATATGAGTACCAATTCATGGGTAACTCTTTTAATAGATACAGGTGCAGAAAT
TTCCCTGCTTAAATGCAGAAACAATAATCTTAACGATTTAAATCCAAAAAATA
CAACAAATATATCAGGAATAGGGCAAGGGACAATTCAGTCTCTAGGTACACT
ACATTTAGAAATGTGTATTGCTAATGCAGCAATACCATATGAATTCCATATCGT
ACCTAACAATTTTCCTATACCAGGGGATGGTATAATTGGCTTGGATTTCATTAA
GAAATACAATTGTATTTTGGAATTCCACGACCAAGAAGATTGGTTCACTTTGA
GGCCCAAAAATTTCAGGAACATAAACATTCCTATTATACATACACTAGATAATG
AAATAATTTTGCCAGCTAGATCAGAAGTGATTCGAAAGATTCAACTAACATCT
ACTGACACACATGTTCTCATTCCCAACCAAGAATTACAACCTAGCATAATAAT
CGCAAGTGCACTCGTAAACACTCAGAACGTTTTGATTCGAATTATTAATACAA
CTGAAAAAGACGCTATAGTTAGTAGCGCAAATATAAAAAGCGAATCATTGGAT
GATTATGATGTATACAACGCAAATATAGAAAATAGTGCACAAAGAACTTCAGA
AGTATTAAAACTTCTTAAATTTCCATCGTTATTCAAAAGCGATTTAACAAAATT
ATGCACCGAATATAGCGATATTTTTGGTCTTGAAACAGAAACCATATCAGCTA
ATAATTTTTACAAGCAAAAATTGAGATTAAATGACAAAACTCCAGTCTATATC
AAAAACTATAGAATGCCAGAAAGTCAAAAACCAGAAATTCAAAGGCAAGTT
GACAAATTAATAAAAGATGGCATTGTCGAACAATCTATTTCAGAATATAATAG
CCCTCTTCTCTTGGTACCCAAGAAATCACTGCCTAACTCGGAGGAAAAGAGA
TGGCGATTAGTAGTCGATTATCGCCAAATAAACAAGAAACTGCTAGCAGATAA
ATTCCCACTTCCAAGAATAGAAGACATTCTTGATCAATTAGGCCGAGCAAAAT
ATTTCTCGTGCCTAGACCTGATGTCAGGATTTCATCAAATAGAATTAGACGAA
AGGTCAAGAAATATAACATCTTTCTCAACTTCAACGGGAGCATACCGCTACAC
GCGATTACCATTTGGTTTAAAAATAGCCCCAAATTCTTTTCAAAGAATGATGA
CCCTTGCATTTTCAGGTTTAACGCCTTCGCAAGCATTTCTGTATATGGATGATT
TAGTAGTCATAGGCTGTTCTGAAAAGCACATGCTTAAAAATCTAACCGACGTT
TTCAAATTATGTAGGCAACATAATTTAAAATTACATCCAGAAAAATGCACTTTC
TTTATGAAAGAGGTTACTTATTTAGGTCACAAGTGTACTGACAAAGGTATATT
GCCAGATGACTCTAAATATGAGGTAATAAAGAACTACCCCAAACCAGTAAAC
GCAGACGAAGCTAGACGCTTCGTGGCATTTTGCAATTATTACAGAAGATTTAT
TAAGAACTTTTCTGAGAAATCACGCCACTTAACGAGGCTTTGTAAAAAGAAT
GTTCCATTTGAATGGACAAGCGAATGCAATGATGTATTCGAATATCTCAAAAG

GAAATTAATGAAACCAACACTCCTTCAGTACCCAGATTTCAGCAAACAATTTT
GCATAACCACAGATGCTAGTAAACAAGCATGTGGAGCGGTACTATCTCAAGA
CCATAACGGTCAACAGCTACCAGTGGCATACGCTTCAAGAAGCTTTACAAAA
GGCGAAAGTAATAAGTCCACTACAGAGCAGGAGCTAGCAGCTATTCACTGGG
CAATAAATCACTTCAGACCATACGTATATGGTAGACATTTCTTAGTACAAAGT
GACCATAGGCCACTATCATATCTTTTTTCAATGAGAAACCCCAGTTCAAAATT
AACCAGAATGAGACTAGACTTGGAGGAGTTCGAATTCACAGTAGAATATCTC
AAGGGGAAAGATAATCATGTCGCAGACGCATTGTTCCGAATAACAATCGGAG
AACTTAAAGCAATAAATAGACAGATACTAAAGGTAACAACAAGATCAACAAC
AAGACAGAAAAATACCTGCGCAGGTGAAAAATTGCATGAACCAAATGAGAA
AGAAAATATAAAAATGCCCAATATCTATCAGGTAATCAATAACATTGATGCCA
AAAAATATGTTATACTCAAAATAGACAAGCATAAGTGTTTGTTGAAAAGAGG
AAAACAAATTATAACACGTTTTGATATGACTAATTTTTATTCTAATGAAATAAT
CGATTTAGATCAATTCTTTCAAAGGCTTAATGAAGAAGCAAGAATAAATAGCA
TCATTCAAACACAATTGTCACCAAGTGAACAAATCTTCGAATTTGTCACTATA
AAGAACTTTAAAGAAAAGGGCAATAAAATACTAAAAAATTTAAAAATAGCGC
TATTAAACAAGGTGACTAAGATAGATAAAAATGATAAGGTTCAAATAAAAGC
AATACTGTCTAAATATCATGATGATCCATCAGAAGGAGGCCATTCAGGAATTT
CTAGAACCCTGAGGAAAATGAAAAACTGTTGTTGTTGGCCACGAATGACGA
AGGCGATAAGTGAATATGTTGAAACATGTTTGAAATGTCAACAAGCCAAGAC
TACGAAACATACTAAAACACCGTTGACAATAACAGAAACGCCAGCAACAGC
ATTTGATAAAGTTTTGATAGATACCATTGGTCCACTGCCAAGATCAGAAAACG
GAAATGAGTATGCTGTTACTATCATTTGCGATTTAACAAAATATTTGGTAACGG
TACCAATTCCAAATAAAAGTGCAAAATCAGTTGCTAAGGCTATATTCGAAAAT
TTTATTCTAAAGTACGGTCCAATGAAAACAATCACAACGGACATGGGAACGG
AATATAAAAACCAAATTATAGACGACCTATGCAAATATATGAAGATAAAAAAC
ATTACTTCAACAGCACACCATCACCAGACATTAGGAACAGTAGAACGAAGTC
ACAGAACTTTCAACGAGTATGTTCGCTCATATATATCTGTTGACAAAACCGAT
TGGGATATATGGATACAATATTTTACTTATTGTTTCAACACAACACCATCGGTA
GTTCATGAATATTGTCCATATGAATTAGTATTTGGAAGATTACCAAGACAGTTC
ATAGATTTTAACAGGATAGACAGAATAGATCCTATTTACAACATGGATGATTAT
TCAAAAGAAGTTAAGCTACGATTAGAAATAGCATATAGAAGAGCTAAAAATAT
GTTAGACAAGGCAAAAGCCGATAGAAAGATAAAATATGATAGAAATATTAGTA
ACTTTGAATTAAAGATAGGAGATAAGATATTACTTAAAAACGAAACGGGTCAT
AAACTTGACAATAATTATTTAGGACCATATTTAGTTTCAGAAATAGGAGATAAT
GACAACATTACAATTATAGGAAATAAAAATAAAAAACAGATAGTCCATAAAG
ATAGGTTAAAAATTTTTAATTCATAATACATTTTGTTTGGTTGGCCAACCACAA
ATAAAAAACCACAAATAAAAAACCACAAATAAAAAACCACAAATAAAAAAC
CACAAATAAAAAACCACAAATAAAAAACCACAAATAAAAAACCACAAATAA
AAAACCACAAATAAAAAACCACAAATAAAAAAACCACAAATAAAATAAAAA
CCAATAAAAACATTATAATACAAAACTTTTACTTTGCAAAATATAATGAAAATA
TATATATTTTTTTAATATCTCTTTAATCATTCATTTCAAATATTAATGTACATTTA

AAAAAAAAAAAAAAAAATATTATATACTTGAAAATAACTTCATGTTATTACGTT
ATTTTTCAAAAGGAGGGAGA

>MAX_LTRBEL#LTR/MAX_LTRBEL

TGTTCACGCCAGAAGGGCGCAAACAGTTGAGCGGCAGTGTAAGCGGCCAAT
GCTTGTACTCGAAGCTCCTCCACGGCTCGCAAGCACCGCTGCTCGCGCTCTC
CTTCTCTTCTCTCGCTCTCCTCCCTTCCCGCAGGGTATTCACCACTCCGCGGT
CCCGCGGTATTTCTACTGTTAGTGTTAAGGTGGTTGTAATTTACCAGTGCGTG
AATAAAGAACGAAAGGTTGAAGTCCACCCCGCGCGTTTTTAATTTCGGGGAA
AATTTCAGCAGCCGCCGCAGCAACCACAACCAAAGGAATCTTTCCGCCCACT
CCATTTCA

>MAX_IBEL#LTR/MAX_IBEL

TGGTCCTTCGAGCCGGATGAGCTCTGATCCTGCCAGCGCTCCTTGCGGAAAA
TTCAAGATAAGTACGGCTTTAAATCCTATTCCGTCCGTGGTCGCCATTTTCGTT
TTTTCCAACGGCGTTTATTCTTTCCGTCCATCCGTATCAAGTGAAAAGTGAAA
ATATATAAGTACATGCGCCCAGCCACCGCGATTACTTTTTTTTTAAGTGCATA
AACAAGCACTTTGCCAATACCGGCCCCCTAATTCGTCTAACGGTGCTCCGCTG
ATATCCAAGCGCATGTACATATATACATATACACACTGTCCAAAATTCACTGCT
ATCGTCCGCTGCTAAACTGCTGTTTTTTTTTTTGTGCCTCAATAAAATATACAA
TACAATACAATATACAAATTTAATTAAATTCAATAAAATATACAATACAATACA
ATGTACAAATTTAATTAAATTCAATTAAATATACACATACATATAATCACCAAA
AATTTCACATACACACAAATATACATAGCCATATACATACATTACGACATATCC
ATTGTCATCCAACGATATTCACATACACACAGTCGCGACGTACACAGACTTGC
CAAGTGCAAGTTCTTTCGTCTCCTCTTTGTTCGTTATTGCCCTGACAAAAAAA
CCAGCGCATAGCGACGAGAGAATACCCTATTGTCGAGACATAAAACAAAATA
AATAAGTCCGACGATAAAATTTAAAAATAAATTTTAAAATATTTTATTTATTTAT
TTTTATTATTTAATAAATAATTTAATAAAAAAAAGGGAGAGAAACGATTGTTTT
AATTATTATTATTCTTGATTATTAGTTAATAATTATTTAACCCAAATAATAATTTT
TTTTTTTTTTACAAGCTATTTCTGGCTCCCATTTCTGGTTCCTAAATTACAATAA
TAAAATTGTGGTCCTAAATCCGGTAGCTATTTTTATAAATTTAAACTTGAATTT
TCTAGCTATTTTCCATATATTAGCTATTGTTTTTCGGGATTTCCAGTCGATAGCG
ATTTTTTTTATAAATCCTTGGCTATTTTCCAGACTCCAGCCGGTAGCTATTTTTT
TTGCTTTTCGGGATTTTTCAGTCGGTAGCGATTTTTTATAAATCCTTGGCTATTA
TCCAGACTCCAGCTGGTAGCTATTTTTTTTTTTTTTTTTAGCTATTTTTTCAGA
AATTGCAATAAACTGAATTTTAGCTATATTGTGCTTCAATCTCCAGATCCCAAT
TGCAACCTTCTCAATTTAAGTTTTTTTTCCAGTTGATTTAAGATCTATTGGGGC
CTTATTTCACAAAAATTGAGATTTTTTTTTTTTTTTTTCCTTCGTGCTCCAATC
TCCTTCTTGACTTCCTTCTCCTAGGGCATAGCTGAGCATGCCCCTAGAGGGAG
ACAAGAAAAAGACACCCGCACCTGGAAAAGAACAAAAGTTCAACCCGCAA
TCTCCGATATCCACTCGCGGTAGGCCAGCACCCAAGTCCGTTAGTCCCAGGG
TCAAAACCGCGACTCCCACTCCGAAACCAAAGGTTTTGCCATCGACCAGTTC
GAAGACAACTCCTAGGTTGGTCATTTCCCGACCAGTGACGCGAGCGTCTAGT

GAGTCTTCTCTATCGAGAAAATCCGAGAGTCCCTCCGCTGTCGGGACCGATT
TCCTTCGCGTCACACGCTCTACCACAAAGAGAATGGCATCCTCTGAACAGCC
GACGCCCGCAAACGCAGCGTTGCATAAATTCATCGCCGTCAGCGATCGCGTA
AGCCTTTTCGAAGCGAAGATCAACACTCCAGATCAAGCCTCTCCGTCCCTAC
ACACGTTACAAGTCCGTCTGCAACAGGTGCGAGCCTTATGGGACAAAGTGG
AAAGAGAGTACGAAACATGCTCTGACCTAATGGCCCAAGAAGGATCCCTAGA
CACAGTGCCTATTCTCCAGGCCAAATATGACTACTGCTACTCAGTATACGAGT
CATGTGCAGCACAAATTGGCGAAACAATCGACAGAGCAACGCCTCAAGTCG
CGCAAGCACCCTCTCAGCCGCTAATTTCGTCCGGCTGCCGCTTACCTCCATGC
GACACGGAAGTCTTCGATGGCGACTACCTCCGATGGCCCACTTTCCGGGACC
TATTCACGGCAATTTACGTAAACAACCCCAGGCTGACTCCGGTCGAGAAGCT
ATTCCACCTCCTTACCAAAACAAGTGGCGAAGCAAAAGCCATCGTGGCGAA
ATCTCCTCTCACGAATGATGGTTTTGCTTCGGCTTGGGAGGCGCTTCGAGATC
GGTTCCAAAATAAGCGACTTTTAGTCAACAGCCAGCTCAAGCTTCTTTTTAAC
TTGAGCTCAATTTCGCAAGAATCTGGTCATGCTCTAAAAGAGCTACAATCCAC
GATTCAGGGGTGTTTAACGGCACTAGAGCATTCCCAGGTATCCACAGAAAAC
TGGGATTGTATCTTGGTTTTCCTTTGCGCGAGCAAACTGCCAAAGCAGACCC
TGTCCTTATGGGAACAGTCGCTAACTGCCAAATCCGAGATCCCAGCTTGGGA
AGAAATGAATGCCTTCCTGAGCGAAAGGTATCGGACATTGGAAGCCATCGAA
GATATGAAACCGACTCAGGCGGTTCCCAAAAGGCTTCAATCCTTCGAGACAA
AGGTCAGCACCAAACAGAAAGGGTGTGACATATGTTCTAAGGAGAACCATC
CGGTACGATTGTGCCCGCGTTTTCTTCAAATGTCTGTTGACTCGCGCTCCGGA
TATATAAAAAAGAAGCAGCTATGTCTGAATTGTTTTGCCAGAGGTCATCAGCT
ACGTGACTGCACAAGCATGCACAGCTGCTTTACATGTAAAGGCAGGCACCAT
ACGCTGCTGCATCGCAGCCCCCCAAATTCCGAAAATGCGAGTTCCTCAACCT
CGCCCCCTACACAGCAACTTCCGAGACCCTCTAGCAGAAATGCATCGGCAAG
CACCTCAGCGGTGCAAAATTTCTTCGCGTCCGGCACTTCAGCCGTCCTACTG
AGCACAGCGATGATAGACGTGTGCCATTTGGGGACGAACTACCGAGCCCGA
GCCTTAATCGACTCGGGATCCGAGGCGACGTTCATTTCAGAACGCCTGTTCA
ACCTTATCAAGTTGCCATTCCGCAACACCCGGACCCAAGTCTCCGGGTTAAA
TCATTCGGTCTCCGCGAAATCCTCGAAGCTGTGTCACTTCGGGATTCGCTCTC
CGACTAAGCCAGGTCTACAGTTAGACACTGAAGCGTACGTCCTTCCGGAGCT
CTCAGGCAAACTGCCCTCCTATCCGATCCCTCGGAATTCTTTGAAGGACCTGC
CCGCACTCCGCTGGGCAGATCCTACCTTTTTTGAGAGCTCTCAAATTGATGTA
CTGATCGGGGCTGACATTCTACCATCCATAATGATGGATGGCACCCGACAAAA
TATTTGCGGCTCGCTCCTGGGCCAAGAAACTATTTTCGGGTGGGTGCTAACG
GGGCCGATTTCCAAGGGTAAGCCGAAACGGGTCGCATCCTTCACGACCCAG
GTGCACCAAACAGGCGACCCTGATTCGCTCGACACGCTTCTCAGCAAGTTCT
GGGAGGTGGAGGATCTACCAGTAAAGATGGTAAAAGAGTCGGACTCCTATTG
TGAAAGGAACTTCCTCCAAACGACCACAAAAGACGCAAGCGGGAGGTACGT
GGTGACGCTCCCGTTCCGGGATCCCGAGAATACCGGATCCGATTTAGGATATT
CAAGGTCCATTGCGCTAGCTCAGTTTCTTAGAAACGAAAATCGTTTAAAAAG
AGACTTTCCATTAAAAGAGCAATATGACAGCGTGATCCAGGAGTACCTGGAT

CTGGGGCATATGAAAGAAGTTCCGCCGACTCACAATTCTCCCTCGTATCATCT
TCCTCATCACGCGGTAGTTAAGCCCGAAAGCACCACTACAAAGCTTCGCGTT
GTATTCAACGCTTCAAGTCCGTCGGCAAATGGGATCAGCTTAAATGATATTCT
TCATGCTGGTCCAGTCCTACAATCCGATCTAACGATCCAGGTCCTGAAATGGC
GTTATTTTCAATACGTCTTCAGTGCAGACATTACGAAAATGTATCGTCAGATCT
GGGTCGATCCAAAACACACCCCGTTTCAAAGAATTCTGTTCCGAAACAAGG
AAGGAGATATCCGAGACTTCGAATTAAAAACAGTTACCTTCGGGGTCAACTG
CGCCCCCTTCCTCGCCATTCGAGTCTTGCAACAGCTGGCAGAGGATATCCAA
GTGCCATTTCCAAATGCCAGCCGCATCATCCAGCAGCACATGTACGTCGACG
ACGTTCTGGCAGGGGCGAATTCCGTAAACGAAGCCCAAAGTTCAATTCGAG
AGTTGCAAGCAGCCCTAAGTGCCTCCGGGTTTCCGCTAAGAAAGTGGACCTC
AAACAACAAAAGCGTCCTTAAAGACGTCCCGGCCGAACACCTCCTTCATAGC
GAGTTCCTCGACATCGATGCCGAAAGCACGGCCAAGACGCTCGGTATACGGT
GGAGGGCAAAGTCCGACGAATTCTATTTCGTTCCTCCAGACATAGTTGTGGA
GGCCTCCTATACAAAGCGAGAAGTTTTGTCCCAAATCGCTAGGTTGTTCGATC
CTGCCGGGTGGCTCGCGCCGTTCATAATCCGGTCAAAAATATTCATGCAGGA
GATTTGGTTGCAAAACTTAGGCTGGGACGATAAGCTTCCCACTGAAATGAGT
CAGCGGTGGCAATCGTTTTTAGAGGAGTATTCCGACCTCAACCAGATCCGGG
TTCCGAGATGGATCTGGTACCAGCCTGAGGTAGTCATAGAGCACCACGGTTT
CTGTGACGCGTCTCAGAGAGCCTATGGAGCGGCTATATACATCCGCGTCGAG
ATGGGGCAAAAGATCCTGACTCGCCTGCTTACAGCCAAAACACGAGTAGCCC
CAGTCAAAACCGTCTCCCTTCCTCGGCTAGAGCTCTGTGGTGCCGTGCTGTT
AACCGAAATGGTGACAGCAATCCTTCCGCACATGCCCTCCGCCAGTTCAGAT
ATCCGCTGCTGGACAGATTCCACAATCGTCTTAGCCTGGCTACGAAAGCCTG
CGTGCAACTGGACCACATTTGTGGCCAACAGAGTTGCCAAGATCACGCAGG
CGACGCCAGTCGACTGTTGGGCGCACGTTCGCTCAGAACAAAACTCCGCCG
ATCTAGCCAGTCGAGGCGTATCCCTTCAGGAATTGGCAGAAAACCATCTCTG
GTGGCATGGACCAGAGTGGCTGCAAGGGCCACGAGAGCTATGGCCAGCACA
AAGCGACACTCTTCCAGTGACAGAGCTAGAGCAGCGCGCGGTCAAAGTGCA
TTTTGTCAAGGGCCCGTCCATCGACTTTCTCGAACGTTTCTCCAAATTGGACA
AGGCCCTGCGAGTTCTGGTCTATGTTCAACGCTTCTTTAAACGCTGCCGCAA
AGGTTCGTTCTTGCCCAGTTCACGACCTACGAGTGAAGAGATCCGAGAGGCA
GAACGAACTCTGACCTCCATTGCGCAGCGCAGAGCATATGGCCAAGAGCTTC
AGCATTTGACCGAGAAAAGGCCTCTTCCAGTGTCAAGTCCTTTGGTGACTTT
GTTCCCGTTCATTGACCAACACGGCCTTTTAAGAGCGTGCGGCCGTCTCACT
GCATCCAAAACCCTGCAATATGATGAACGTCATCCGATTCTTCTCCCCTATGA
CTGTAGACTGTCGCGCCTCATCGTCCAATTTACTCACCAGATTACTCTTCATG
GCGGTAGTCAATTGATCGTACGCCTGATCCGAACCAAGTATTGGATTCCTAAA
ATCAAGAATCTGGTGAAGGCTGTGGTCAATCCGTGTAAGATTTGCACGATTTA
CAAGAAGAGACTCCAAACACAGTTGATGGGCGACTTCCCGACAGATAGAGT
CTCCTTCTCGAGGGCGTTCACCTACACGGGTATCGACTATGCCGGCCCTTTCG
AAATAAAAAATTATACAGGGAGAGCGTGTCTCATAACGAAGGGATATGTCTGT
GTGTTTGTGTGCTTTTCCACGAAGGCTATCCATTTGGAACCCACTTCCGATCT

AACAACCGAGAAATTTCTAGCGGCCTTTGCTCGTTTCGTAGCGAGGCGCGGT
TGTCCTCAGCGGGTCCATTCGGATAATGGTAAAACCTTTGTGGGGGCGGCAG
CTTTGATTTCCAGGGACTTTCTCCAAGCCATCAAAGAGTCCGTGACTGATGC
ATATAGCCACCAGGGACTCGTATGGCGGTTCATCCCACCAGGGGCTCCACATA
TGGGGGGTTTGTGGGAAGCAGGGGTGAAAAGCTTCAAAACCCTGTTCCTTA
AATCGACGTCAGTCCGCAAATATACATTTGAGGAGCTGGCGACGCTTCTCGC
TAAGATTGAGGCTTGCCTCAACTCTAGACCCCTCTCCCCTATGTCCGAAGATC
CCTCAGATTTGCTGGCCCTCACACCAGGCCATTTCCTTATTGGAGGGCCGTTG
CTTTCCACGGCGGAACCCGAGATAAAGGGCGAAGCCAAGTCGATAATAAATC
GATGGCAACACCTCAAGGCACAACATCAGCAGTTTAGTGCACGGTGGAAAG
AGGAGTATCTTAAGGAACTCCATAAACGAAGCAAATGGCAATTTCCGACCAG
GAATCTCCAAGCCGACGATATGGTAGTTGTCAAAGAGGACAATCTACCACCG
AACGAATGGCGGCTCGGCAGAATCGTTTCTGCCTTCCCAGGAGCCGACGAG
CGCATTCGAGTCGTCGAGATCCGTACGTCTCGCGGCACCATAAAACGCCCTG
TCCACAAAGTTATTCTGCTACCGATGGAGGACAAAGAGTCCTCCGTTCCTAG
GGATTAGGGCACTTCCCCCATTCCGGGGCCAAAATAGTAGTCGGCTCATACTA
AGCTTCTTCATTTCTTTTATTGGCAGACTTACTCACTCCTCCAATAATGGCTCC
TCGTCCTCGTGCCACCCAGTCGTTGGAGAGCAGACGTACTCGAGGTATTAAA
TCCTACCGCTGCCGAGTCTGCTCTGGAATCCATCCTCTTCGAAAGTGCACGA
GGTTCCACAAACTGAGCGTTGAAAAGCGCCTTCGGGCAGTACTTATTAATAA
GTACTGCTCGAACTGCCTCGCCCATCAGCACTCAGGAGGAGACTGTCGCAGC
CAAGAGGGATGCAAGAAGTGTGGAGGAGACCACCACACCCTACTCCACATG
CACGAGGTCCTCCCCGCTCCGAACCCGGCAGCGCTACCAGCTCCGACGCGC
AGAGAGCGCCATCCCGCTGCACCTCGTCCGGTCCGGATTTCCCGCACTCCGC
CACCAGCCCCAGTCGCCAACAATCGCCCGCGTCAGCAGCAGCAGAAGGCTG
TCCCCATACTGCCTACAGCCATCGTCGTGCTGGACACGGGCTCGAAGACCTT
CGAGACCGGGGCCATGATCGACCCATGCATGCCGGTGAGCAGCATCGACCGG
TCGTTAGCGGCTGCGTTCCGGCTGCCCATCACTCGGCTGGGAGGCAACGAGA
TCTGCTCGGTGACACTCCGGTCCCGAACCAGCACCTTCCGACTCAACGTCGT
CCTGAAGATCGATCCCATCCTAAGGATCCGGACACCCATCCGAGCTCTGAGC
GACGCCGCCAGGGCCAAGTTTGACGGTGTTCGTCTCGCGGACGAGCGCTTC
CACCGGCCGGCCTCCATCTCCCTCGTGTTGGGATCAGATGTATATGCCAATTT
GATCCAACCGGGGTTCCTAAAAATTGAGGATGGGTTGCCCGTCGCGCAGAAC
ACGGTGTTCGGATGGACCGTTTCTGGAACGTGCGCGAAATGAAGAGGACGA
TCCTGATATCTAGCCGGCCAGGGATACCTTTGCCTACGCAAGGGGGGGGAGA
A

>MARINER2_Mariner-Tc1#DNA/MARINER2_Mariner-Tc1

CAGGGTGCGGCAGCATAACTTCCTTTTTTAAAATGCGCGCCATTCAGTTAGTT
GATGTCATAGCGGAGCGCTAGTGGTCCCGTTCAAGAGGGGCTACTGCAAAGT
TTTGTCCCGACACGGTTCAGTCGCCATCATGCGTTGGAATAGTGAGGAGCGT
GCCTTTGCCGTTGAGGCCTACTTTTCAAGCGGATGTTCGGTTATTAAAACACA
GCGTGCATTTCGGAATCGCTTTAATTTAGCCCCGTTGGCTCCCGTCCCAGACC

GCAAATCAATTGTTACATGGGTCACTACATTCAGGCAAACTGCAAGTGCGAC
AAAACGAAGAACTGGAGTCCCTCGACCCGTTAGATCACCTGAGAACATTGA
AGCAGTGAGAGCGTCAATATTGCGATCTCCACGGCGTTCTGCGCGCAAACAC
GCATCTGCCCTTGGACTTGAGGATTTGGGGGACACTTGGTTCCAACAAGACG
GTGCAACAGCACACACTTCAAGAGCATCGATGGCTGTTTGAGGGAACACTT
TCCAGAGCGCCTTATCTCAATTAGAGGCGATTTGGAATGGCCGGCACGCTCTC
CCGATCTGTCCCCTTGTGATTTTTTTCTATGGGGTTTTTTGAAATCCCGTGTTT
ATGTGAACCGTCCAAGAACCCTACAAGATTTGAAAACCAACATCCAAGAAG
AAATTGCCAACATAACACCTGCTATGCTAACAAGAGTCACGACAAACGCCAG
AAATCGGTTTACGCAATGTATGGAGAATGGGGGACGTCACCTATCAGATTTCA
TCTTCAAAAAAATATAAATAAAAACTTTAGACATGTACCTACATTATAAAAATA
AATAAATATTTTCCGATGCCTACAATAGTTTTCATTGAGTTCTGAAAAAAGGA
AATTATGCTGCCGCACCCTG

>M4Transib#DNA/M4Transib

CACTATGGGGCATTTGGCCTGTTTTTTTACAAAAATTAATACCTCCTAAACTA
TTGGAGATATTTGGATGAATTTTTTTTATGCGTTACACATGCCTCCAGGAATA
TTTTGGAAAAGTGGGCGTGCCCCAACTCCGCCCCATTTTTTTTTTTTTTTTTT
TTTTTTTTAATAATATATTTTTAAAGTTTATTTTTAATTTCAATAATGTATAATTC
ATAACCGTCTTCCTCTTCACAATCAGTAGAGTCTGAAGAATTTTTATCAGGTT
CAAATTCGCAAGCTAACATTTCAATGACTTCTGGTGGAAGAGATAGTCGCTTA
TGTTTTCGCCTCTTTAAATTTATTGATGATATTATGGGATCCGAAGTATCCATTG
CTCTGTAAAAGACATCTGCGAAGCTACTAATAGTTTTTGCCGTGGCTGGCTTC
AACAAAAGAATTTTAAGTATGGCTGCAAGATCCCGCAGGCAGCACTTCCGTG
CAGCTTGAACCAAAAGACGTTCGTTGTGTTTCTGCGCCCTTACGAGTTCATCT
GCTTGCTGTCTTGGGCCACTCAAATTTTTTAAATAATATGACGTTTTCGGGAG
TCCAACTAATTTCCTTTCCTATTTATTTTTCTCCTTTACCTTCAGGACTAGGTGT
TCTTCTAACCAATTTGAAAAAAATTTAAAAATTCATATATTTTTCGATTGCAT
TTTCTCCAATTTCGTAAAAGATTGACTGAAATCATTCGTTATTATTATTATTAGT
TAATCGTTTATTAAAGTCTAGCTTGCTATCAGAAAAATGCCCACTGATAAAAG
TGCAAATAGAATTTTCCTTTTGACGAACACCCTTTTGCGTGCGCCACACTTCC
AGCAGGGCAGCATTGGAAATCGAGATATTGCTCCCTAAAAAATGAAATTTCT
CAAAAAACCGCAAAAAACGCACATAGAGACTACCTGATATGAGTTAGGAATT
GAACACACTACAACATGGATATAAACACTTACTGAACAAATTTGAACAAATT
GTTGTAGCTCTATTCAAAGTTGAAAATTTTTTCAAACAACTACATCTTGACAC
CACTTGTTAAATGTACAAATTGTTAGAAATAGGCGCACACAATAAACAATATA
TTAATAACAACACATAATAAGAACCTAAAGATTGATTATCCATTTCAAATTATA
CTCTCCTTCTTCTTCTTTTTAAATTTTAACACTTTGAAAGTTAAGCTAAATTTT
GTGCGCAAAGCAGCCACGTGGTATATGCTCGCAACAGCCGACTTTAACAGCT
GTTATTATAACAGTGCATTGTTAAATTAACTTATGCGGGCTATATCATAACAGTT
TAACGTATTTCCAATGTATTAATACTAAAATACTTCAAATTTGCATACTTGTGA
AAAACACATTATTGTAAAAAAAAACAGGCCAAATGCCCCATAGTG

>LOOPER1_piggyBac#DNA/LOOPER1_piggyBac

TTAACCCTTTCGCGCCCAACGTTGCTTGTGAGCAATTTCTATGTTTTCAGAGC
TAACTGTAAGCGTGCCTTTTGTTTTTATCTGCTCATTCTTTATGATAACGGTGC
TTTTATGTGTCTACTCTAAATATGAAAAGCAAAAAACGCATTTGGAGATTATTT
GCAGAAAATATTTGCAATAAACAGCACGCGCATCACAATAATTATTGTTTCGA
TATTCGGTGGAACTTTTGGAATTAGTAATTTACTTTTATAATACTTTAAAAAGA
ACCTAAAGTATAAACAAATAATTATTTAGCAAAGAGTTAACAAGACAGTGATA
TAGTCAAACTTAGTTAATAGTGAACAATAAGTAAGTAAATAATTTTCTAAGTT
GCTTGTGAGCAACCTTGGGCATTCGTGGTAATAATTTTCACCGAAAGTGAAA
GGTTTGTCTAACGAAGAAATTGAACGGGCTTTAAACTTCGATTGGGATATTTC
TACTGACGAAGAATGTGGTGATGAGATGGAAGACATTTCTACGGTACTGGAG
AAGAATTTAGAAGGAATTCTTGAAAGGGGAGAAAGTATCGAGATTGACCTTC
TGGAAAATACAGTGATTGAAGGATGTGAACCTGACTCTTGCGTTGTTGTTGA
CAGTTGCTTGGAGAAAATTGATCCAAAGACGCTAAAGTGGAGAACAAGGCC
GTTTGTAGCTCCAGAGAGTATTTGGGAAGATGACAAAACTTTTGATGTCGGG
GAGATAAAGACGCCAGTGGAGTTCTTCTACACACTTTTTGATACTCAGCTAAT
TCATTTAATGGCAAGGCAAACTGATATATATAGTTTGCAGGAGCACGGTATTG
AACTTAAATGTACTGATGAGGAAATCAAACGCTACATTGGCATTTTATTGTAC
TTTGGTGTTTTAAAACTACCGCAATTCAGAATGGCATGGTCAAAGGATTTAAA
GATTACCGCAATAACTGATTCAATGCCGCGTGGGAGATTTAAAAAAATAAAA
CAATGCTTACATTTCAACGACAACGCCAAACAATTAAAAAAAGGGGATTGCA
ACTATGATAAACTCTACAAGATCCGCCCTTTGCTCAGAATTCTCAAAGAAAAT
TTTGGAAAAACTAACGCAGGAAGAGCATCAAAGTGTCGATGAGCAAATAATT
GCATTCAAAGGTACGTTTTTAATTTTCTTTTAAATTTGCTTTATTTTTATTAATT
GCTTTTGTTGCAGGTCGATCCACGCTTATACAATCCAAACCTCATAAATGGGG
TCTTAAAATTGTTTACGCGGGCTGGAATATCTGGATTAGTTTATGATTTTACGC
TATATGTTGGAGAAGGCACTTCTCCTTCTTATGGCTTGGGAATATCATCTTATG
TTGTCTTATATTTGGCAGAAAGTCTTCCCAAAGACAAAAATTTTAAACTGTAT
TTTGATAATTGGTTTACGTCTGTAATCCTTCTGATTTCGTTGAAGGAAATAGGA
ATCTTTGCAACAGGTACTGTACGTATGATAAAGTTGAACATTGGGTAGTTTTT
GGAGAAAGAGGACGTTGCAGACTGTGCAAAACTGCAACACCGATGACCAA
ATGCCTTACATGCAAAGTCCATCTGTGCTGCAATAACAATAAAAACTGTTTTT
TTGTCATACCACACTTAAATTGTCATTATAAAGAAAAATATTTCATATTCTGTG
ATTTATAAAAAAAAAACAATGCTTACACATCACTACTGCCCGACGTTGCTCAC
AAGAAAACTTTCGCTACCGCCCAAACTAATGGGCGTGGCATACTAAAATTTT
GCTAAATTTTTCTAAAAATAAATGTAAAAACATTAATGATAAAACAAAATTTC
ACGGGTAAAAAGTTGGGCGCGAAAGGGTTAA

>JOCKEY2Jockey#LINE/JOCKEY2Jockey

CCTTCGTTGCATTCGGACGTTCGCGATTGTCTAGTCTTTTTGTAGTGAAACAT
CAACAAATCTTCCAATCTTACTGTGCACGCGCACAATTTCTATTTCTATCCACT
GCACAGCAGTGGTGACAGACAATTACGGTACTTATCATAGATAAGCTCAACC
AAGCCGACGCTTTGTACGCCCCTCGTCTACGCCTTGGGCAAGGCTGTTACAG
TTTGTTCTCCGCTTGTTATCTCTCGAGCTTGCGAAGTTGACAACTAGTAGTAA

CACCAATGTTACCAACACCATAACCACGACTCAAGCTAGCTGTTCGCAGGCA
AGAAGTGCTGCAATGTCTGATGCCATATTTTTGGGCACTCCAAAAACTATTGT
CTCCAGGATCCAATCTGTGGCAAGTGTGCTGGGCCCCACGCAACTGGCTCTA
CGCTATGCACGAGTGACAATTATTTGTGCATCAGCTGCGGTGGTGATCATGCT
TCAACGGACAAAAACTGCCCTGTCAGAATTGAAAAAGGAAAGAAGCTCTAG
CCAAACCCAAGGCTCCCTACTTTTGTTGCTCATGGCAACACTGTCAGCTCTA
AAAAAGGTGAAAGCTATACTCACGGATTCATTCCGGCTGAGGCCATCAGAAG
CAATATTTCAACCGCCGATATTGTACAGGCCAAAGGCCCTCAATTTGGTTCAC
TTGCGACTCACCAACACCACAGTGATCCAAACTCACCACAAGACTTAGGCTT
TGGAAAAAAGTTTCAGGCGCTGGAAAGCGCCATCCAGGATATAAACTCGAG
AATGGACAAAATCTTCAAACTAATTGGGGAGACTGTGGAATCAAAAAAGGTT
TTTAGAGAACTGGTTCAGATCTTCATATCACGGACGTCAAAATGACCCAACC
AATAATAAAAATCGGATTGTGGAATACACGAGGACTAGCTAGAAACTCTGAG
GAGCTTCAGCTTTTCCTAAGGATGCAAAATATTGATATCATGCTTGCCACCGA
AACAAACGTGCGGGAAGGACAGCGTATCTCTCTACGTGGTTACTCCACCTAC
GACGCCAGTGGAAACAGCAGAGGTGGAGCCACTGTCATAGTGCACTCTTCC
TTGAATCATTGTCCCCAACCCCCAATCTCAACCAACGACCGGCAGGTAGCGA
GCGTGCAGCTGCAGACCTCAGAGGGAACAGTTACTTACCCACCCAGCGAGC
GGTTGATAAAAGCTTATATTGAGTAACTTTTTGTAACACTTGGTGGCATATTTA
TGCAGGTGGAGACTACAATGCAAAACATCATTGGTGGGGAATCTCAAGAGCC
TGCGCCAGAGGTTTACAGTGCGTTATTTAAAAATAAATAATATCAATTGTAATA
ATGGATATTAAAACTGTGATTCGCTCCCTATCCAGAGAAAAAGCTAAAGGAAT
CATCCAAGGAATTTGCAAGAACAGGAGATTTCCGAACCCAGCAGATCTACAG
AAGGTTATCAAATCGGCTCAATAAAGTGCTGACCCAAAGAAAACAGCTACAT
ATATACAACCTTTTGGATTACATGGGCACCGATGCCCCTTCACAATTCTCCCTG
TGGAGAATCACGAAATGGTTTAAGTTTCAGGCTTGTCAGAAATCCGCTGCAA
GGAGCCCGTCTGGTGGTTGGTCCGCACATCACAGGACAAAGCAGAAGTGTT
TGCAAGGAGCTTGGAGCAAAGATTTAGCCGCTCGCATATGCCGATGCGAAT
CATTGCCGTTTGGTTGCAGAGTCTCTTTTAACGCCATTTCAAATGGCACTGCC
CGCGGATCCTGTCACTCTCGAAGAACTGAAACAACTTGTTTCCTTGTTGAAT
TCAAAAAAAAACCGCGGTTCATGACCTTCTCGACAACAGAACGATAAAAAC
CCTATCAGACCATGCTCTGCAATTTCTGGCACAAAATTTTAACAGCGTTTTAC
TTGAGGGTTATTTCCCAAAAGTCTGGAAGACTGCAAACATAATCCTGATACTG
AAACCAGGGAAAAGGCCAACAGAGGTAGACTCGTATAGACCAATCAGCCTC
CTCCTTTCCCTTGGTAAAATTATGGAAAGGATAATTCTCAACAGAATGCGTGA
CGTTGAGCCTGTAGTATTGGCGATACCTGATTTCCAATTCGGATTCCGGACTC
AACATGGAACATCCGAGCAACTCCAAAGAGTTGTCAATTTTGCGCTAGAAGC
CCTAGAAAGGAAAGAGTATGCGGTAGCAGATTTTCTGGACATTCAACAAGCT
TTTGACAGACTTTGGCATCCTGGACTTCTACATAAAGCAAAGAAAATACTAA
CACCCCAGCTATTCCAGCTTGTGACGAGCTTTTTGTTAGGACGAACTTTCTGT
GTGACGACCGATGGATGCACTTCGTCCGTTAAAGCCATCGAAGCTGGAGTAC
CTCAAGGAAGTGTGTTAGGCCCTACTCTATACTCCATCTTCTCCGCGGACATG
CCCACCCAGACAGCAGTCACTGGATTAGATCGTAAAGATGTGCTGATTGCAA

CATATGCTGACAACACAGCGGTACTGACAAAAAGCAACAGCATTATCGAGGC
CTCAGACGCACTGCAAGAGTACTTGGACGCATTCCAGAATTGGGCAGAGTTG
TGGAACATCTGCATCAATGCTGACAAATGTGCAAACGTCACCTTCACAAAGC
GCATAGGTAGTTGCCATTCTGTCTCACTTAAAGAAAGAGTGCTTGACCACAA
GTCATCATACAAATATCTTGGAGTGATTTAGATAGAAGCTTAACCTTCGGAA
AGCATGTCACGGCGATACAGCAGTCCTTTAAAAATAAGGTTTCTAAAATGTCC
TGGTTAATTGCTGCCCGCAACAAGCTTTCCCTCGCCAACAAGGTTAAAATTTA
CAAGAGCATATTAGCTTCTGGTCTATTCTATGCCATACAGGTGTATGGAATCGC
TGCCAAAACGCACCTGAATAAAATTCGGGTTCTTCAGGCAAAAACTCTGAGG
AAGATCACAGGCGCTCCTTGGTACATGAGGACCAGAGATATCGAACGTGATC
TGAATGTGACAAAAATTGGAGACAGGATTCAGGAAATAGCGAAAAAATACA
ATGACAGACTAGAATCTCATCCTAATAGTCTAGCTAGACGACTAAGTATCGCA
GCCCAAAGCCATCGGACAAGTACGAGAAGAAGATTAAAACGCCACCACCCT
CAAGACCTGACTGACCGGGACTTGACATAAATCCTACTTAATTAAAATCTTCT
AAATATTACTGCCTAAACATTCCATTTCTTTAATACTCATAATCATATAATCTTT
GTAAATAACTTAGATATTAAGAATCGTTTAAATTGATACCGATTAGATTAAGTT
GTCCAAGAAGGGTAATAGTACGCTAGTCAATTAAATAAAATAATTTCGTTTAA
TAAATTAATTAAAAAAAAAAAA

>JockeyJockey#LINE/JockeyJockey

AAAAATCATTCACATGGGAGATGAGCAATCGAGTGGACGTGTTCACAGAAGT
CGCGAGATAAAACAAAAACGTAATTGTGATCCATCACAAACATCTGCGCAGA
TCGTGTGCTTATCTCACAAACAAAATCTATTTTAGTCACTGCATAACGGTGA
CGGCTTCGGTTCGCGAAACTTATCAGCAACTAGCAATTTCTAAGCTGTGTTGT
TTTTGCCCCTCGCCCTGCGCGCTGCGCAAGCGGGAGGTTGTTACAATTTACCT
TACAAGTAAACCGGTAAATCTTATCGTGTTTAGTAAATATCAATTGCATTATAC
GGCATAAGTATAAAGACAATTGATATAATGGAGAATTCATTTGCTCAATCGCG
ACCTAGCAATGGGTGCGATAAATTTGAGAAAATGAGGAAAGTAGCAGGTGTT
GAGCCAGGAGAATTACGCTCCCAACTCCGCGCCAGCTGTGCAGTTGTTTCCC
CTAACCTGGAAGGTATGCCAACTCAATCTGCGGTCTCCAGCTTAATGGTGAC
AATCAGCAGCAACACCAATGCAAGTGTTACCTGCACTATTTCTAACGTACAG
GCCAACATGATCTGTACTCCTACATACACTGATTGCACAACCGTGACCACTAG
CATTTGCCCAACTACGCCTTATGACAATGGACTGCCGACACCTCTGTCATCAC
TGCCCAATAAGCCATCTAAAGCGAATTGCCCCTTTCAAGCACATGATCGTACT
GTCAACAGGAAACGAAAAGGCGTGTCTCAGCCCCCATTACCTATCCTCACCC
CTTCTCCAAGCCGTAAAACTAAAAGGCAGGCCACTATGCCACTCAATGAGGA
GGCCTCTACCTCCACTGCAGCAGCATTAAATAACAATCGCTTCGCGCTTTTGT
CCGCTGAAGCGGAGAATATGGAGCAAGACGTGTCGGATGCTGATTCTGACAT
TGAAGACTCTGCTGCCCGAGATGGTGGTGGACAATCCGCTAAATATAGCAAA
CCCCCAGCCATATGCGTACCAAGTGTAAGCGATCCGGTCACCTTGGAACGGG
CTCTCAATCTGAGCACCGGCTCCTCAAACTACTACATCCGCATTTCTAGATTT
GGTGTATCCAGAATCTATACAGCCAACCCTGATGCTTTCCGCACCGCTGTAAA
AGAACTAAATAAGTTAAATTGTCAATTCTGGCATCACCAACTTAAAGAAGAA

AAACCCTACAGAGTAGTGCTTAAAGGAATCCATGCTAATGTTCCTAGTTCGCA
GATAGAACAAGCATTTAGTGATCACGGCTATGAGGTCCTTAATATCTATTGCCC
CAGAAAGTCTGACTGGAAGAACATTCAGGTAAACGAAGATGATAATGAAGC
TACAAAAAACTTCAAAACTAGACAAAATTTGTTTTATATTAATCTTAAACAAG
GCCCGAATGTTAAAGAGTCTCTTAAGATAACTCGACTTGGCAGATACAGAGT
CACTGTTGAGCGCGCTACACGTAGAAAAGAACTGCTACAATGTCAAAGATGC
CAAATTTTTGGACACTCTAAGAACTATTGCGCCCAGGATCCTATTTGTGGTAA
ATGTAGTGGTCCCCATATGACCGGGTTCGCTTTGTGCATAAGTGACGTATGTC
TGTGTATAAATTGTGGTGGTGATCATGTCTCGACAGACAAAAGCTGCCCTGTC
AGAGCAGAGAAAGCCAAGAAGCTAAAACCAAGGTCCAGGCTACCGATGACT
AATAATATTGCCACACTCAAACCTCCACAACGTTCTTCAAGCGGTTACATACC
AGCTGAGGCATTAAGAACCAACATCTCTTATGCTGATATTGCTCGACGCAACA
CGACTCAATCTAGGGCTCGTGCTACTGTGCAGGCTGAAGTTATACCAACGTC
GGACAATAGCCTTAACAATAAATTTATGACGTTAGACAACTCCATTCGGGCCA
TCAATACGAGAATGGACGAACTATTTAAGCTTATACACGAAACTGTAGAGGCT
AATAAAGCTTTCAGAGAACTGGTTCAGGTTCTAATTACACGTATTCCTAAATG
ACTCAACCAACCTTAAAAATCGGATTGTGGAACGCTCGCGGATTAACAAGGG
GCTCTGAGGAGCTTCGGATATTCCTCAGCGATCACGATATAGACGTAATGCTT
ACCACGGAAACACACATGCGAGTTGGTCAGCGCATCTATCTCCCAGGGTATC
TTATGTATCACGCCCACCACCCCAGTGGTAACAGTAGAGGTGGCTCTGCAGT
CATCATAAAATCTAGACTTTGTCACAGCCCTCTGACACCTATCTCTACTAATGA
CAGGCAGATAGCGAGAGTGCACCTGCAAACATCGGTTGGGACCGTCACTGT
AGCTGCTGTTTATCTACCTCCAGCAGAAAGATGGATAGTAGATGACTTCAAAT
CCATGTTTGCTGCGTTAGGCAACAAATTTATTGCTGGTGGTGATTACAATGCC
AAACATGCATGGTGGGGGAACCCAAGATCCTGTCCTAGAGGTAAAATGTTGC
AAGAAGTCATTGCACATGGGCAATACCAAGTTCTGGCTACGGGCGAACCCAC
TTTCTACTCTTACAACCCTTTGTTAACACCATCAGCCCTTGATTTTTTTATAAC
CTGTGGGTACGGCATGGGCAGGCTAGATGTACAAACTCTCCAGGAACTCTCG
TCGGACCATCTTCCTATTCTGGCTGTATTGCACGCTACGCCGTTAAAGAAACC
ACAACGCGTACGACTACTTGCCCATAATGCTGACATAAACATATTCAAAACCC
ATCTTGAACAGCTGAGTGAGGTAAATATGCAAATTCTGGAGGCGGTGGACAT
TGATAATGCCACAAGCCTTTTCATGAGCAAACTAAGTGAGGCTGCTCAGCTT
GCTGCACCGAGAAATCGGCATGAAGTAGAGGCCTTCAGACCACTTCAACTTC
CTTCCAGTATATTGGCACTGCTCAGGCTAAAACGAAGAGTTCGAAAAGAATA
TGCTAGAACAGGTGATCCCCGCATGCAACAGATCCACAGTAGACTGGCCAAC
TGCCTGCATAAGGCCCTTGCTCGAAGAAAGCAGGCCCAAATAGATACCTTCT
TGGATAACTTGGGTGCTGACGCGAGCACAAATTACTCACTGTGGCGTATCAC
GAAACGGTTCAAAGCTCAGCCCACCCCAAAATCAGCAATCAAAAATCCGTCT
GGTGGCTGGTGTCGCACTAGCTTGGAAAAAACTGAAGTGTTCGCTAACAAC
CTTGAGCAACGTTTTACACCCTATAACTATGCACCGGAAAGTCTCTGTCGTCA
GGTTGAAGAATACTTGGAATCGCCCTTTCAAATGAGCCTGCCTCTGAGTGCT
GTCACACTGGAAGAAGTGAAGAATTTAATAGCCAAGCTGCCACTTAAGAAA
GCTCCTGGAGAAGATCTTCTTGATAATAGAACCATTAGACTTCTCCCAGATCA

AGCATTGCAGTTCCTTGCCTTAATATTCAACAGCGTTCTTGATGTTGGCTACTT
TCCGAAAGCTTGGAAATCGGCGAGCATAATTATGATCCATAAGACTGGAAAA
ACACCGACAGACGTTGACTCGTACAGGCCCACCAGCTTACTCCCATCTCTGG
GTAAAATTATGGAGAGGCTGATCCTAAACAGGCTGCTCACATGCAAGGATGT
TACCAAAGCGATTCCCAAATTTCAGTTTGGCTTCCGGTTGCAGCACGGTACTC
CTGAGCAACTACATAGAGTAGTGAACTTTGCTCTGGAAGCTATGGAAAACAA
GGAGTATGCAGTAGGTGCCTTTCTTGATATTCAACAGGCATTTGACAGAGTCT
GGCACCCTGGGCTCCTGTACAAAGCGAAGAGGCTGTTCCCGCCGCAGCTATA
TTTGGTTGTTAAAAGTTTCCTGGAAGAACGCACATTCCACGTCTCTGTTGATG
GGTACAAATCATCAATCAAGCCAATTGCAGCTGGAGTTCCTCAAGGAAGCGT
TCTTGGCCCAACCCTATACTCAGTTTTTGCTTCGGACATGCCTACTCACACAC
CAGTCACAGAGGTAGACGAAGAAGATGTGCTCATAGCCACCTACGCTGACG
ATACTGCTGTGCTCACGAAAAGTAAAAGTATCCTGGCTGCCACTTCTGGTCTA
CAGGAATACCTGGATGCATTCCAGCAATGGGCTGAGAACTGGAATGTGCGCA
TCAACGCTGAGAAGTGTGCCAATGTGACGTTCGCCAACCGAACAGGTAGCT
GTCCGGGTGTCAGTCTGAATGGAAGACTGATCAGACACCATCAGGCTTATAA
ATACCTTGGTATTACCCTCGATAGGAAGCTCACCTTCAGCAGGCACATCACAA
ATATTCAGCAAGCGTTCAGGACCAAGGTTGCTCGGATGTCTTGGCTCATTGCA
CCACGCAACAAACTGTCGCTTGGCTGCAAGGTCAATATTTACAAGTCCATATT
GGCCCCCTGCCTGTTCTACGGCCTGCAGGTATACGGCATTGCTGCGAAGAGT
CACCTTAATAAGATCCGGATTTACAGGCGAAGACCTTAAGAAGAATTTCGG
GGGCTCCTTGGTATATGAGAACAAGAGACATCGAACGCGACCTCAAGGTGCC
CAAATTAGGAGACAAGCTCCAGAACATCGCCCAAAAATATATGGAAAGGCTT
AATGTACACCCCAACAGCCTAGCAAGGAAGCTAGGAACTGCAGCTGTGGTC
AATGCTGACCCTCGGACTAGAGTCAAAAGAAGACTCAAGCGACACCACCCT
CATGACCTCCCTAACCTGGTTTTGACCTAGAAAGTCTTAGTTTTAAAATTCAT
TAGAATAATCAAATAAATAATAATTACTATGTTATATCAACTATTATAATTCTCCC
TATCATTTTTAGATTAAAAATCTGTTAGTCTTAAGTAACCAAGACACATTGTAA
AATAAAATAATTTAAGCAGATCAAATTAAGTTGCCGCATGGGTAACAGTGCGT
TGATCAAATAATAAAAACATCATAAAAAAAAAAAAA

>IVK_Jockey#LINE/IVK_Jockey

CAGTCACCGACTATCTCTCTTCGAGTGCGGACGTCTGTGTCTTTCCAATTGTG
TGCGCTTTGCTTATCGCGTAATTACCATCGCCCAGCTTATCGCCGTTACATTGA
TCTTTATCATCTTATCTTCTTGATCTTCTTATCAACGCGTACGTGCAAATCCAA
AAACATTCAATATCCATAAATTTATTAACCATGGCCCCCGGGCCACTCAATTTG
GGGGATAATCGCTTTGCCAGTCTCAGCGTTAGCCCCAAAGCTAAGAAAAAAA
GACCCTTTCAAAAATTACAAGTTGATTTCCCAGACCTCCCTGAAACGCAGTG
CAAAAATCCGAGATATTTAGTGGTCTCATCAAAAAACTCTCCGAAAACCATAT
CCGACTACAACTGCTTTGCTGTCCATAGAGCCCTTAAGTATATCAGCAAAGAC
ATTACATCCATCTCTAATCTTCGCGATGGTAACCTTCTCTTGCTGGTTAACTCC
AGGGAAGTAAGTGACAAATTTATCAAAGTGACCTCTTTACCTGGCTTATGCG
ATATCGAGTGCAAGCTCCACAACACTCTTAACTTTGTCAAAGGCACCATTTAC

GCTCCCTGTCTTATTAACATTCCAGAAAACGAAATTGTTGAGGAACTAAAATC
TCAAAATGTACACAGCGTATTTAAATTCACCAAAATGATCGACGGCACCCCCA
AACCATTTGGAAAAATTCTCGTAACGTTTGACCGTTTCACTCTTCCCAGTAAA
CTAACGGTTTCCTGGCACACAGTTAAGGTGTCAGAATATATACCCAACCCGAT
GAGATGTAAGTCTTGTCAATTATTGGGCCACACATCAAAGCACTGCAAAAAC
CCACCCGCCTGTGTATCATGCAATCTCGCTCCCCACCTTCCCGTGCCATGCAC
TCGCATTTTCTGTGCAAACTGCACCGGCGAACACCCAGCCTCCTCACCCGAA
TGCCCACAATACCAAACACAAAAACAACTGCTCCACATCAAAACAAGCAAA
AAATGCAGTTTCCATGAAGCTCGCACTATACTAAAACAACAACAAAACACAA
CAAATAACCCCACGCTTACCTACTCGACCGTCGCAAACCAAAGCACAGCATC
CGTGATGCTTGTCAAAAAATCCCGAAACCCTAACACGATTACAAACACCGAC
TTGCCCACCAGCTCGACAAAAGTACCAGCTCCGCTCTCACCAACGTATCCCA
CTTCTCGGACGAACTCTTTGAATCTCGAAGAGAATCTTACTTCCTCCCCAAGC
ACCTCTGCCAAAACTGATTTCTCTTACTTAAGCCCAACAAGTAAACTGAAAA
CATGTATGGAACGATCTCGCTCCCTTAGGGCGGAAGCAAATGCTCTATTAGGA
CAAACCTACAACGACGACAACAATACATTAATGACCTCAAGATCCAACTCAA
ACGAATCAATTGCTTCACAAACCTCAAACCTACAAACAAAAATCAACACAAC
AGACTCAGATGACACTATGGACGATCCCGACTCCTAGTCCTGATCCTCTCACC
CTTACCTTCATATACACACACCACAAATTAATAACAGTCCAACAAAAAACAAT
AAATAAAAAAAAAAAAAATTTTTAATCATGACAATTACTATATTACAATGGAAC
ATCCACGGCATTTTTAATAATTACAACGAACTGACACTACTCATAAAAGACCA
TGCACCCGATATTGTATTTTTGCAGGAAACCAACCTTCCATGTAATTCTACAA
ATTTTATTTGCCCCAAAGAATATAGTGGTTACTTCCACAATTTTTCTTATAACA
CCTCAGCCAAACAAGGCATTGGTGTTCTTATTAAAAGGAATGTCCCACATACA
TACCGTAACATTAACTCCAGCATACTCTGCTCCGCACTGCAGCTTAAATTTGA
ACAGGTCATTAATATTGTTAATGCGTACATCCCACCAAGTCAAATCTTTTCATC
CTCTGACATATCAGAAATTCTACAAAATCTTAACGGATCCACAATACTCCTGG
GTGATCTTAACTCATGGAGTCCTTTATGGGGTTCGCCTCGCACAAATACAAGG
GGTAAAAAAATTGAAAGCGTAATTCTTGAAAACAGCCTAATTGTTCTTAACG
ACGGTTCCCCGACTCACCTTTCAACTCACAACACGTTCACTCACATCGATATC
TCACTGATATCTCCACAGATAGCCCATATGTGCAGCTGGTCTATATCAGATAAT
CTCCATGGAAGTGACCACTTTCCCATAACCATCCACATAAACACACCTACCCG
TCCCGATAACACTTTACCCCTGCCTAAATACAAGACAGACCAAGCGAACTGG
AAACGCTTTAACGAAAGTTGCGAAAAATCAGCAGCATATTGGACGGTCGGTT
GCCTAAATCAGCAAGTCGCACAAATGACGAAAGTCATACGAGCAGCTGCAA
ACTGTAGCATTCCCCAAACGAAAAGAGTGATCCACAAAGCCAAAGTGCCAT
GGTGGAACGCTAACCTTCAACAGCTTAGGGACCAAAAGCAACGCTTGTTCTC
GTCCTACAAAGCCAATACGAATGATACAAACCTTATCCGATACAAAAAAGCA
AACGCGCTTTTCAAAAAAGCTGTACTTTCGGCCAAGCGTAACTCCCTTGAAA
AATTTACTTCCAAAATCTCCCCAGTATCCTCCACAAAAAAGGTCTGGTCAGAT
ATAAAACGCCTAGCCGGCATCCCTCCCACTCCGTTCAAATATATCAAATCCAA
CTCGGGTACTCTAACTGGATCATTTGATATCGCCGAAGAGTTTGCCTTATCTTG
GTCTAAATACTCTTCTGACCAAAATTTTTCAGCAGAATATATACGAGTAAAAA

ATCGGTATCTTTTAGAGCCCTATGCCATCGACTCACTTTCCCCATCGGCTACAT
CCTTAGATTCCAATTTCACATTGCTCGAAATAGAAAATTCAGTAGCAAAAGCA
AAAGGGAAAAGCCCTGGGGCTGACAGAGTATCATACCCTATGCTCAAAAACC
TATCTCCCCACCTAAAAACAAAACTTTTAGACATTTTTAATCAAATATTAACCA
CTGGAAAATACCCACATACATGGAGATCGGCTATCATTATCCCTATATCAAAAC
CTAACAAACCCCCTTCCGATATTAACAGCTACCGTCCAATCTCTCTGTTATCCT
GTCTGGGAAAAACACTAGAAAAAATAATAGCACAAAGACTTACCTGGTTCAT
TAAACGCCACAACCTAATTTCCCATAATCAAGTTGCCTTTAAAAGAAATCATA
GCACGATGGACGCGTTATTGCGTATCCAACACTTCGCGTCGAACGCTCTTTCG
ACCAAAAATCACGTCTCTATCTTGGCGACGGATTTTGAAAGAGCCTTCGATC
GCGTGGGGATTCACGCCGTACTTTGCAGACTCGAGCGCTGGGGCATTGGTCC
AAGGCTTTATAACCTTATTAAAGCCTTCATGACCAATCGGTCCTTCAGGGTTC
GAATAAACAATGTCACATCGAACTCCCACATTTTACACAATGGAATCCCGCAG
GGTTCGCCACTTTCGGTGGTTTTATTTATGATAGCTATTGAAGATATAAATGAC
ATTGTAACTCGGCATAAAGATATTTACATCTCACTATACGCAGACGACGCAAT
AATCTTTACAAAAATAAAAAATATTAACACAGTCAGAGAAAAATTCTTAGAA
ATATTGCAAGAAATTAACTCGTGGGGAGCAACCTCTGGGGCCTCTCTTGCCAT
TGAAAAATGCCAAACTTTACATATCTGTCGGAAACAACGTTGCAACCTTTCC
GACATTGTCTTTAACAGCCGCACAATAAAAGATGTAAACTTTTTAAAAATCTT
GGGGATAACCTTCGACTCCAAACTACTTTTTAAACAGCACTGTCAGACTCTA
AGAAAACAACTGGAAACTAGATTTAACATTATTAAATTTCTATCATCTAAATAT
TCTTACATACATATTAAGACTCTCATAGATATTACGCGCGCATTAATGCTATCTA
AAATTGACTACGGACTGCCAATCTTCGGTTGGTGTGCTAAATCACACTTAAAA
AAGCTACAAGCCCCATATCACGGAGCGGTCCGTCGCGCCATTCACGCATTTC
CCACATCTCCAGTAGCGTGCACATTGGCAGAATCGGGTCTCCCGAGTATCCA
ATCACGCGTAGAAGAAACTACATTGATGCTTATCCCGAAGCTGTACACCACGT
CCAACTGCCTGCTAACCAAAGACTTCGGAGCCATATTTAAACAAAAGCGGAA
ATTCAAGTGCATATCCACCCTAAGACGTTGCGCCAACTACATCAAGCTACTTG
ACCTTCCCCTGCCTAAGCCCAGGAGGCCCTTCAAATCGCCAGCACTTTGGGG
TTCCAAACAGCCTAACATAAACCTTCAAATATACAATGCTGCCAAAAAGGATA
CAGGTCGCCTAGAATACCAAAAACGTTTTATGAGCGCACAAGAAGATCTTGG
TGTGAAAAACTGGATCTACACCGATGGTTCTAAAGTCACCGGCGCAACTACT
TTTGCGGTTGTTGACTCTAACCGTAAAATAATTGCAGGAGGTAGGCTTCCGTC
CTACAACTCCATATTTACAGCCGAAGCTTTCGCCATTCTCAAAGCATGCCAAT
TCGCTTCCAAAAACGCTGGAAAATCTGTTATCTGCACAGACAGCCTCTCCTC
TCTTTCCGCTATACGCAACTGGAACCATAATGACCCCACAACACAAGAAGTT
AGGCACATTCTAAGTTCTCACCCAAAAAAAATCACCTTACTCTGGGTTCCCA
GTCATCAAGGTATTCATGGGAATGAACTTGCTGACAAAGCCGCCCAAGAGAT
GAGGCTTACACCATCAATCCTGTTCACTCCGTTTAACTCCAAGGACCTAAAA
AGTCGAATCAAGTTATACCTCAAAGAAAAGAAACTCTCCGAATGGGCACTCT
TCATGCACAGGTACCAGTCTATCAATCCGAATTGCATCATGTTCAAGCCACCA
ACGAACGTACATAAACGGGAATGCGCGACCTTTATCCGTCTACGCATCGGGC
ACACCCAATCCACACATCAACACTTACTGATGAGATCGGCGCGACCAACATG

CCAACTATGCGGGGACGAGCTCACCGTTGACCATATTCTCAACGCCTGCAGT
CAATTGCACTCAATTAGATCTCACTTATTTGGTACACATAGTCTCTCGAATTGT
TTAAGTATCCCATCCTGTGAAAATATCTCAAAAATTTACAAATTTGTCCAAAA
AGCTAAGTTTATTATATAAAGCAATTAACCCATAAGTCTCGAGTCGAAGGCCC
CCGTAGCTAGTACTCATTAAATTTAATTAGGTTTAGTATTGTATACTATATTTAAT
TTTGTTAAATAAAATAAATAAA

>INVADER6_LTRGypsy#LTR/INVADER6_LTRGypsy

TGTCGCATCATTATTAGTCTTATTTTTATTTTCTATGTTCCATCTCTAATAAACAT
GTCATCTCTATTAAATAAAATTCGTATCGAGCTGTTCTTGTCTTCGTTTCTCTTT
GATCGCTGTTCGCTGTGTTCCGTTATGCGAGTTTAACGGGTTTTGCTCTGTTC
TACATAGTCTCGGTTCGACGATGCGTTAGAGTGAGACAAATGCTTGTCCTGTG
GTGAGTTCGGACCAGCATGTATCAAGCGAGATAGAGCGATGTTGAAATGTAC
ACGGGGCACTTATGTTTGAAAACTCTGAGAAAGCGGACGCGTGAATATGTCG
CAACCGAGGAAGTGTACGACTCGCGGGCGGAGCGCGGCAACAGAGGACCC
CGAATCAGTTAACTTCCCGACA

>INVADER6_IGypsy#LTR/INVADER6_IGypsy

TCAGAAGTGGGATCGCCTCAGCCCCAAGCCATCACTGATGATCGCCTGTCCA
CAATTTTAGAAATGCAGCATCGTAATCTATTGGAAATTGTAAATGCCGTGAGG
GGCTCGCAGACTACACAAGTAGTTGTACTACCCAAGTTCAACCCTGAGTCCG
CCGGTTCAAGTGCAGCCACTTGGTGCTCTACAGTGGACCTTATTGTAGGAGA
AAATCCTCTGGATGGCAGTGCTTTGCTCATGGCCTTAACCAAATCATTGGAGG
GCAGTGCTTCTAATTGGCTGTCACAAATTTGTTTCGCCGGAATGACTTGGAGC
CAATTCCAAGAAATGTTCCTTCAGCAGTATGAAGGCAATGAGACGCCAGCAG
CAACAGTCTTTAATGTATTAAACGGGCGGCCAAACGACGGCGAATGTCTTGC
GCTGTATGGTAGCTGATTAATAACAACACTTATGGCAAAATGGAAGTCTATGA
CCGCAGAAGAAATTGCAGTGTCTGTTGCCCTGGCACATGCTGCAAATATAGA
TGGTAGGCTGCAACGCACTGTGTTCACAACTACTGTCAAAACACGCAACGA
GTTGCAGAACGAGCTAAGAGCGTTTTCGTATGGCAAGAGGAAGGACCATCC
CGTTCCAGAAAATTCTACCAGCAAACGAGCTCGCCTACACCCAAATGTTAAG
TGCCACTTTTGTGGAAAAATTGGCCACAAGATAGCTGACTGCCGCTCCATGA
AAAACAACTTAAAGAATCAACAAGGATCTAGTTCGAGTATTGGGCGCTTATC
TGACTCTAAACCTGGGTCAATTACTTGCTATAGATGTGGAAACCAGGGGCATA
TAGCGTCAGCTTGCCCTGCAAGACAATCGTTGTCAAACCAAACTAAAGCCGA
CGAGAAGCGTGTCAACGTGTGTCACGTAGTCGAGCCAATTGGGACATTGATA
TCATCTGGTGAGTCGTATCCATTTTATTTCGACTCTGGAGCCGAATGCTCACTT
GTAAGAGAATCTGTGTCCACCCAACTCTCGGGCACACGAATTAACAACAATG
TAGTTTTAAAGGGTATCGGAAATAATACTGTTACCAGTACATTACAAATTTTGT
CAAACGTAACAATAAGTGGTTACTGTCTCGAAGTGCTTTTTCACGTAATTCTT
AATGATTGCATTAATTATAATATTATAATTGGACGCGAAATTTTAAGTCAGGGA
TTTAGTGCTACTATAACAATAGATAAAATAGAGTTATGTAAAACAAGGTCTGT
GCAAACCCTATCTGCTTAGAGTAGTAGTTTTAGTCTTGAAAATGTTAATACCG

AATTGTGTGGCGAGGATAGGAAAATCTTGGTAAATCTTTTGAATAAATTCTGT
GACTCATTTATAGACGGTTTTCCCAAAAATCGTGTTACAACTGGCGAACTAGA
AGTACGCTTAATTGATCCAATAAAAACTGTACACAGACGACCGTACCGACTTA
GTATAGAGGAAAAACAAATTGTCCGAAACAAGGTTAATGAGCTGCTGTTAGA
TAACATCATCCGTCCTAGCAGCTCACCGTTCGCCAGTCCAGTTTTACTCGTTA
AAAAGAAAAATGGTTCTGATCGCCTTTGCGTGGATTACCGCGAACTAAATAC
AAACACAGTTGCAGAGAAATATCCCTTACCACTAATTAGTGACCAAATATCTA
GGTTGCGTGGAGCAAGTTTCTTTAGTTGCTTGGATATGGCCAGCGGGTTTCAT
CAGATACCTATTCACGCAAATTCAATTGAGCGCACGGCTTTTGTGACACCTGA
CGGCCAATTCGAATTTCTAACTATGCCCTTCGGGTTAAAGAATGCCCCATCCG
TGTTCCAGCGTGCAGTTATGAAAGCTTTGGGTGAGCTTGCCCACTCTTACGTT
ATCGTTTATATGGACGATATAATGATTATCGCAGAAACAAAAGAAGAAGCTTT
TGTAGGGTTAAGGACAGTTTTGAAAATAATATCGCAGGCTGGGTTTTCTTTTA
ATATCGGAAAATGTTCATTCCTGAAATCTTGCGTTGAATATCTGGGGTTTGTG
GTAAAAGAGGGCGAAATAAGACCAAATCCATCTAAGATAAAAGCATTAGTCG
CTTTACCGCCTCCACAGTCTGTTACCCAAGAAAGACAATTTATTGGCCTAGCC
TCTTATTTTAGGCAGTTTGTGCCAAAGTTTTCAGAAATCATGAAACCCTTATAT
AGACTGACCTGCAAAAACAAAATATTTGAATGGAAACTTGAACACGAACAA
ATTCGTCAAAAAGTCACTAAATTGCTTACAGATGAGCCCGTCCTTGTTATCTT
CGATCCTCGGCATCCCATTAAACTGCATACAGATGCCAGTATGGATGGCTACG
GAGCAATTCTACTCCACAAAATAGATAATAAACGTCGTGTAGTTGAGTATTAC
AGCAAACAAACTCCTTGACGGAATCTCGATATCATTCGTACGAGCTTGAAAC
TTTAGCTGTGTATAACTCCATGAGACACTTTCGTCACTATTTACATGGGCGAC
GATTTGTTGTTTTTACAGACTGTAATTCCCTAAAAGCTACTCGCAACAAGACT
GAACTAACGCCGAGAGTACACCGTTGGTGGGCATATATGCAGTCCTTCGACT
TTGACATAGAATATAGACCTGGTGCCAAAATGGCACATGTTGATTTCTTGTCA
CGCAATCCACTGCCAGCTGCTCGGGTTATTACTGGTGAGGAAGAAAAACATG
TTCTATTGGCCAAAATAACGGACAACTGGTTACTTGCAGAACAGCAAAAGGA
TTCAGAGATTTCCACGATTGTTGTTAAAATACAGAACAATGAATTGGGTGAG
AGCTTGGCAAAAAGTTATGAATTACGCTCGAAAATGCTTTTTCGCAAAATTCA
AAGGAACGGTAAAACTCGTTGCCTGCCAGTTGTCCCCAGATCATTCAGATGG
TCAGTAGTGAACCAGGTCCATGAAGCAGTTGTACATCTTGGGTGGAAAAGA
CTTTAGACAAAATATACGAATTTTACTGGTTTGAGAACATGGCCAAATATGTT
CGTAAGTTCGTTGATAATTGCATTACGWGTAAGTTAACTAAGCCTCCGTCAGG
AAAATTGCCAATCGAACTCCACCCCATACCAAAAGTAGAAATTCCATGGCAC
ACGATCCATATAGATATTACGGGAAAATTAAGCGGCAAGAGGGATCAAAAGG
AATATATTATCGTTCTGATTGACGCGTTTACAAAGTTAGTTTGTTTGCACCACA
CGGTAAACATTAATAGTGAGAGTTGTATAGACGCCTTGAAGGCGTCAATCTCC
GTATTCGGTGTACCAACACGAATCATTGCAGATCAGGGTAAATGCTTTACAGG
CTCTTGTTTCAGTGAATTCTGTTCCATGCACAAAATTCAACTACACTTAATTG
CAACAGGGGCTAGTCGTGCCAATGGACAGGTAGAACGCGTTATGAGTGTGCT
TAAACGCATGCTTACGGCTGTAGAGAGCAGCCATCGTTCTTGGCAAGATGCC
TTAGGAGAGATACAACTAGCTATAAATTGTACTACAAATCGCATAACGAAAGC

CAGTCCTCTTGAATTACTAATCGGAAAAGAATGTAGACCATTTAATATGTTACC
AATATGTGAACAAGTTAATAAAGTCGATGTAAATATTATAAGAAATATCGCGA
AAGAAAATATTAAGAAGAACGCCTTGTATGAAAAAACTAGATTCGATAAGCA
CAAAGCCAAATTTGATAACTTTGGTGTTGGCGATTATGTTTTACTTAAGAACG
AAGAAAGGCACCAAACAAAATTAGACCAAAAATATAAAGGACCTTTCCTCGT
GACAGAGGTACTTGAGGGAGATCGTTATATTTTAAAATCTTTAACTAATAAGC
GGACTTATAAGTACCCACATGAAGCTTTGCGCAGTATGCCAACAGAGGAGAT
CCCCAAAGAGTTAGATCTATGTGACGATCAAGAAAACGTTGAAAGAGACGTT
AGAAATCCCTTGGTGGATTCCAATGTGGATGAAAACGTTGAAACAGACGTTA
GAAATCCCTTGGTGGATTCCAATGGGGATGAAAACGTTGAAAGAGACGTTAG
AAATCCGTTGGTGGATGCCAATGTGAGCGAAAAGTTACTGAGTTGTTTGAAG
ACTCAAGTGAATGAGAGGCATTGATGGATTTCAATGCGAGATTGGGGACACA
TGCAACGTCGCCAAGTTGCCAGTGCTAGAAGGTACACGTGTTACTGTGTTGA
CTTATTTGATGTCTGGTGACTGGCGGCGTGGCGGGTTGAATTGTCCTAGTGTG
TTGCTAATAATAACAAACGATCTTCTTGGTACTTCTGTCACTCGAGTTGGTCG
ATAACAAGAAAAATAAAATTAAAAAAAATAAAAAGAGAACCTTTACTAATAC
TCTGAATTACGTTTAATGTTATCTTTCTAGATTAAGCTTGTTTAATTTCAAAACT
TATATTACACACGAGGACGTGTGCTGGTCAGGAAGGCCG

>INVADER5_LTRGypsy#LTR/INVADER5_LTRGypsy

TGTCGTATATTCCTTTTTAATTTCAAATTATAACGTACATTTTATATTTGCTAAA
AGCTGGGCATATTGTTTTGTTTGGGTATTTCTTCTGTATTCATAAAGCGAGGCA
GAAATAGTTTCTGTCAACGCAGTCTGGCATGGGCATGAATGGGACGGATAGA
GTCCGGTAAAACTCACCACAGTGTGTCTGTGTAGTGGTGTATGAAAGAGAAT
GAGAAAGGGAAACGCTTGGTTTTGGTCATTCTTGGTGATGAAATAAAGAGGG
ATAGTCATGCCACGTGCCAGAAAGCGTTTCGTTGAGGAAACAGAGGAAGAC
TCTTGTAACGAGGAGTCTACAGACCTTGGATCGACA

>INVADER5_IGypsy#LTR/INVADER5_IGypsy

TCAGAAGTGGGATCTTCCAAGCCTCACAACTGTGGAGACAACCTGCGAACA
CTCTTGGAGTCGCAAAATCACACCTTTGCGGAACTCTTACAGGCTATGCAGC
GAACGCAAAGAGTGCCAAATGACGCAGTAGCTGTAGTGCTTCCAAAATTTAA
CCCCGATCGCACTGGTTCAGACGGAGCATCATGGTGTTCAACAGTGGATTATA
TTCTATCTGAAAATCAACTACATGGCACCTTTCTCGTTATGGCTTTCAGCAAG
GCGCTGGAGGGAAGCGTGTCTCATTGGTTATCCCAAACATGCTTTCCGGGAA
TCACATGGATGCAGTTTCGGGAGTTGTTTTTGCAAAATTATGCAGGAACTGA
GACATTGGCCGCAACAGTTATAAATCTGCTGGATGGACGCCCAAAAGAAGGA
GAGTGCTTATCGTTGTATGGGAGCAGGATGGTTACTTCTCTCGTGTCCAGGTG
GAAGTCACTGGATGTGGAACAAATTGCAGTTTCGGTTGTGCTGGCGCATGCA
GCTGGTTTTAATAAGCGATTACGGCGTCTAGCTTTTACGACAGATGTGAACAC
TCGGAACGATCTGCATCAGGAATTAACAGCCTATTCTTTTGATGCCCGACAAA
GTCAATCTTCATATGGTACCACGTCAGCACCATCAGCGAAAAGAGTCAAGAC
ATTCTCTTCCGGAAAACTTGGTCATAAACAGGCGGAGTGTCGTTTAAAGAAG

GAAGCAACTCAAAGCCATCATTCTGCTGGTCAACGTTCTGGCTATACTGAAA
GTAAGCGCCGGTCCACCTTGACCTGTTTTAAATCTGGAAAAGTTGGACATGT
GGCCAGCGTGTGACCGGATCGTGAGGAGCGTCAATATATGCACAATACTGGA
GCCTGTGGAAACGCTGAAGTCCAGTTCATCTCCGTTTGCGAGTCCAATACTA
CATCCAAATTCAATCGAGATAACAGCATTCGTGACTCCAGAGGGACAATATGC
ATTTTTGGCAATGCCTTTTCGGTTTAAAAATGCCACTTCTGTTTTCTATGAGCG
ATAAATTGTGCTTTAGGTGATCTTAACCATAAATACGTCATTGTTTATATGGAC
GATGTACTTATAGCTGCCAGCACTAAGGAAGAAGCTTTCGATAGATTACAGGT
AGTTTTGCAAACACTTATTACTGTCGGATTTTCTTTCAATATGTTCAAATGCTC
TTTTCTTAAATCTCGTATTGAGTATTTGGGTTTTGGAGTTGAAGCAGGTGAGG
TGCGTCCAAATCCTGGAAAAAATACATGCTCTTGTTAATCTGCCGCCTCGCAAA
CTGTGACACAATTGAGACAGTTTATTCGGCTAGCTTCGTACTTTCGCGAGTTT
GTGCCCAAGTTCTCGCAATTGCTAAAACCCCTTCATGCTTTTACATCAAAGGC
CATATCATTTGCATGGAATAGAGAGCACGAGCAAATACGAAAGAAAGTAGTT
ACAAAATTTGACTTATGAACCTGTTCTTATAATATTTGATTCACAGTTTCCTATT
GAGCTTCACACAGATGCCAGTGCAGACGGCTATGGAGCTATTTTGTTACACA
AAATAGAAACCAAGCCTCGTGTTATTGGGTATTTCAGTAAACGGATGTCTCCC
GCAGAATCTCCGTTATCATTCATACGAGTTGGAGACGTTGGCGACATTGTAAA
ATTGTAATTGTAATTGTAATTCACCTAAAGCTTCCCATACTAAAGTCGAGTTGA
CTCCTAGGGTCCATCGCTGGTAGTCTTACAAGCAGACCTTCGATTTTGATGTA
GAATATAGACCAGGTACACGTATGGCTCACGTTTAGTTTCTTTTCCGAAATCG
TATACCTGAATACCTGATATAGTTGAAAAACACATTAATTTGACAGAGATATTT
GACAATTGGTTACTAGCCGTACAGCAAAAAGATGGACAGATATTGTTTCTAAG
TTGCGTAATAATGAACTTAGTGAGGAATTGGCTAAAAAATACGAGTTGCGGTC
AGGAATATTATATCGAAAGATTCAAAGGAACGGGAAAACCCCTTGCCTTCCT
ATTATACCAAAGTCGTTTATGTTAATAATGTACATACATGAAGCCATTATGCATC
TTGGTTGGAAGAGAACCCTTAAAAAAATGTATGAGTACTATTGGTTTGAGAA
AATGTCGAAATATGTTTGTAAATTTGTAGACAACTGCATCACTTGTAGGCTGT
CGAAACTATCATCTGGCAAAATACTACCATTATTTGGTGTCCTTCTCGTTTAGT
TGCTGATCAAGAGATTTTTGCTCTGCTCAAATTTTTGCTCTTGCAAATGGTCA
GGTGGAGCGTGTTATGATTAACTGTAGCTTAAACAGGCCAGGGGTGTTGGCA
GGACTCTTTGGTTGACGTTCAATTGGCTCTTAATTGCACGCCCAACATGGTGA
ATAAAGTAAGCCCATTAGAGTTACTCATTGGTAAAGAAGCGAGACCATTTGTA
CCTGTTGTGGAAAATTAACTACTTTTCATGTCCTCGTAGTAAGAGCAATGGAA
AAACTTAACATGGAAAAGATAAAATTAGATTTGACAGAAATGAAGTAAAATG
AAGTCGGGGACTACGTACTACTTAAGTATGAAGAGCGTCATCAAACTTAATTA
GATGCCAAATTTAGAGGTCCATTCTTGGTAGTTGAAACATTACAGGGTGATCG
GTATAAACTAAAAGCTTTGAATAATAATCGCACATATAAATATTCCCATGAATT
TTTACAAAAAATGTCTGAGACCGGGATTGCCAGAAAGTTTGAAGATGATCAA
GGTGATCATAAAGATGGTCCAGGTCGAGCCAGTGAGGGCTGCGACATTGAAA
TTGAATAATAAGATAATTTTCGGATAGTACGGCTCTCTGCACATATGGGTGCCC
AAGTTATGTGGTTCGCCATTTGTCGTAGTTGAGTTGGAATCTCGAAAGTGATC
GGAAGTATTAAGTTACTACTTGGCCGGTATAATATTAACTTTGTGCTATTTAAG

TTGGCCGGTTTAGTGATAACTGTGTGCTATGTGTGTTGGCAAGTTATGTTGTT
GTGTGTGAGCTAGAAAAGCGAAATATTGACTAGTAATGGTTTAAATGAAATAT
AGTTATTCTAAGAAAGTGCAGTATATCGAAGTTGTTTTGATGTTGGGTTGGTG
GTAATCACAGTCTTATGGCATAGATATAAAGAGAAATGTTAAGTAATTAAGAG
ATGCTTGTTGATGTTCTAAAAAGGAAAGGAAAACATAGAGAAAAAAAATGTA
TTTTGAGTTATCTACAAATTGTAAACTCAAGTGGACGTGTGTAGGTCAGGAA
GGCCGT

>INVADER4_LTRGypsy#LTR/INVADER4_LTRGypsy

TGTCGGAGCGCGACTCTTCGCATTCCAGAAATTCCTTCTGTGATTTCATATTCT
GATGAAATTGTAATATTGCGGTAAAATTCTGTCGAGCGCTGCGGCAGAGGCA
CGAACAGCCTCTGCAGCTGAGTGAGGTCCGATCGAGTGGTCATAAATAGTGT
GTTAGAGAGAGATGACAATGTGGCACACGCCAGTGTGTATAGCCATTAGAGA
ATATGATGAAGAAGGGACATGTAAGAAGATCCCTTCAGTGAAGTTTGACTGC
TGACGTCGATCGGAACTTGCTGCGCTGACGCACAAAATCGCGAAGTGAATAA
ATAATATGGATGAGACTCCTGTTTCGCCGACA

>INVADER4_IGypsy#LTR/INVADER4_IGypsy

TCAGAAGTGGGATCGCCTTCGCCACAAGAGACCAGATCTGATCAGTATATGG
CTGCGTTGGAGGCACAAAACCGTAACCTCATGGAAATAATAAGAACCATGCA
CGCACCGAGAGCATCGGCGTCAAATGAAAAGTCCTTGCACGTCACACTGCC
AAAATTCTGCGCTGACAGCGCTGGAGCAGATCCATCTGCATGGTGCACCACC
GTGGATTTAATCTTTGCAGATAATGCGCTTGTAGGCAGTGCACTCGTAATAGC
GTTAAGCAAAGCATTAGAAGGCAGTGCATCGCAATGGCTGTCGCAGATATGC
TTCGCTGGCATCACGTGGCCGCAGTTTAAAGAACTGTTCATACAGCGATTCGT
AGGAATGGAGACGTCGGCTGCTATTTTGATGAACGTTTTGAACGGACGTCCA
ACACCTGGAGAGAGCTTTGCCCAGTATGGAAGTCGCATTGTCACCTTGTTGC
TGTCTAAGTGGAAGGCCAAGGATTTGGAGGAGATTGCGGTTTCGGTAGCGTT
GGCTCACATGGCACAAATCGATAATAATTTGTTGCGTTGGGTGTTTACGACTA
ATGTGGCTACGCGCAATGAGCTTCAGCAACAACTGCAAGCTTACGCCTTCAA
GAAACGTAACAACGAAGATGATTCTGGCCCAGAAAAAAAGTTAAGAATGCA
GTTGCAGACCATGTGCCATTTTTGTGGGAAAGCTGGCCACAAATTCGCCGAA
TGCCGTGCTCGAAAGGAAGGTACATCAAACACAAAAGGAAGAAACTACAGC
GAAAGCAACACGCCTGGGTTAAAAGATCGTTCGAACATAAAATGTTTTAAAT
GCGACGAAATGGGACATGTGGCGTCTGTATGTCCCAAAGGCCACAACAAATA
CATCGAAAAGCGAGTTGACGTGTGCGAAACTAAGTGAGCCAAGTGGACTGT
GTTTTTCAATTGGGTGAGCCCTTTCCATTTTTTTTTTTGATTCGGACGCCGAAT
GTTCTTTAATAAAATTAAAATAAATTAAGTAACAAATTAAGAGCTTGGTTTCG
TTTTATTTATTAAATCTAATAAATTTAGTATGTACAAATCCAAAGTTATCAAAGC
CGTTCAAAATAATGCACATGAGAATAGTTTGGAAATACTCTTTGACATCAATA
TAGAAGTAAGCGATAATGATAAATGTAAATTAAGAAATATATTGGAAAAGTAT
GCCGATAGTTTCGTAACTGGAATACCAAATAAGGCGCATTGAAAATTCGTTTA
AGTAATAAAAATAAAAGTGTTCAAGGGCGCCCATAGCGACTCAGTTTCCATT

GCTACAGATTTTGGATCAAATAGATCGTTTGCGGTGTGGTAAATATTTTTTTCA
TTTGTTTTAGACATGGCCAGCGGATTTTATAAAATACCGATTCATCCAGATTCT
ATAGAAGAGTCAAGTATGTCTTTCCTTAGAGCTCCCAGTCGCATCATAGTGGA
TCAAGGCAGAAGCTTTGTCAGCAACAAGTTCCGTGAGCTTTGTTCAACAAA
CAGGATTGAGTTGTTTTCAATAGCTACAGATGCCAGCAGAGCAAATGGGCAG
GTTGAAAACAAATGAGCGTCCAAGCAAGGAAAACATGCGAAAGGATGTAGA
GTAGTTCGGTATGAGGTCATGTGTTAAGTGAGAAGTATCATCGAGTGATTAAT
GTACTAGATGGTCATCGACACAATTTGAAGTCGTTAGTTATCAAGCGAACATA
TAAATATTCGCATGAATGTAATGGTGACTCCCAACAGAGAAAAGAGTTGAAG
GATGGCACTAGTGATCGTGCATCTGTGTCCCAAGAGAGAAAAGAGTTTAAAG
ATGGCACTAGTGATCGTGCATCTGTGTCCCAAGAAAGGAAACATTTGGCGAA
CTGTTATGAATAGAATGGCGTGTGACCGCACTATAACAGCTAACTCGCAGGG
ACGAGAAGTGTTATGGCGGGGGTCAAGTGATGATGACTGCACTATAACAGCT
AACTCGCAGGGACGAGAAGTGTTATGGCGGGGTTCAAGTGATGATGACTGC
ACTATAACAGCTAACTCGCAGGGACGAGAAGTGTTATGGGGGGGGTCAAGT
GATGATGACTGCACTATAACAGCTAACTCGCAGGGACGAGAAGTGTTATGGC
CGGGGGTCAAGAGATTGGGTGTGAGAGAAGACACGCCAATGTTATTATGAGA
AATTAAAGTCATGGAAAATGTAAATAGTTTGAAGTTTTGATATGTAAATTGGA
GATGTCTTTGTTAAAGAAAAATCAGTCATGAGATGAATTGTCAATTAAATAAT
TACTGATTATTACTTGTTGTCATTAATTGTTCTTAAGTTGACGAAGTTGTGTGA
CTTGGACTTGATTGGTGGATTAGGCACACGAGGGACGTGTGAAAGGTCAGG
AAGGCCG

>INVADER3_LTRGypsy#LTR/INVADER3_LTRGypsy

TGTGGTAGGATGAACACTCATAAGGCCCACGAAGCATACGAGGAAGCATACG
AAGAAGCATACGATCATATCCCCGTTAGGAATAAGTATATCATTGCTATCTCGC
TCTTACTTATAAGCTAGCATTAAGAGATAAGAATGTACACCTAGCTCACTCTCT
CTTTTGAAAGAGCATAAGAAAAGAGGAATACGCGAGACGCGGCATTCGGTG
GCGTGAGCTTGAAGTGAGAAGTCGAATAAAGAGATTGTGAAGTTATTTAAAG
GAAGGAGCACGTGTATTCATTTTTCGGCGTCCACA

>INVADER3_IGypsy#LTR/INVADER3_IGypsy

ACATCAGAAGTGGTCATTACCCACTATGCCTGACACTAAATGGAATCAATTCT
CAGTGCCCCAACTAAAGAAGTGGTTGGAAGCTCTTGGATGCCAGACTTCCGG
CACAAAAGCGGAACTAATAGTGCGTCTGCAGGCCATTTCCCCAGAAACGCGT
GGCGATTCACCGCCAAATAACGGATCAGCAGCAGAAGAGTTGGAGGAATTG
GATGGAGATGCAGCCTGGCCAAAGCAGAGAGAGCCACAGGGTGGAGACAC
ACGCACGATCCCGTTCGACGAGCAGTCATTGCAGTCAGAACCCCCGGAGAA
CCTCGATCTGGATCAGCTATCGTTGGTGGATGCAGAGAGAGCCACTCTAAAA
GAGCTTCGGGACGAGATAGAGGCAAACATGGCTGTGCTGCAGAGGATCCAA
GCGGATATCGACGACGCGAACAAGAGCAAGTCCGCTCATGTTCGCCAGATCA
ATGACGTCATCGAGAACAACAGCAACGGAAACCACGGCAATGTTAACATATG
CGCTAACAGCACCGACGCCAATGTTAACAGCACCAAGGTGACTATCGCCGCC

GATAACATTGATACGGGACATACCAGTTTTGGAAAAGAAACCGCTGGTAGTG
TTGTTCAGCACCAGAGTCGAAATGTAAACAAGCTTCTGGAATCTCCTGCAAC
ATCGCTTGCCTTGGCAAAAGAGGTAACGATGGAGTTTGACGGCAGCTTATGT
GCGCGCAATTGGGTCACGCAGTTTCAAAACATCGGAAAGATTTACAATTTGG
ACGACGGATGCATGCACATGCTGCTAATTGCCAAACTAAAAGGAAACGCACA
GCGCTGGCTGCACGCAAGCGCCACACGCATCCTAGAATCGACCGACCAGCTA
TGCGAACAGTTAATCTTGACATTCGAGATCAAGATGTCAAAAGGGGAACTGA
GGAGCGCATTTCAAAAACGCGAATGGCATCCGGATGAGAAATTTGCTGTTTA
CTTCGAGGACAAGGTGATGCTGGCCAACGACATCAACATCGATCTAGAGGAG
CTCCTGGAAAACATCATTGAAGGAATCCCAGCACCAGCGTTGCGCAACCAG
GCGCGCATACAGCGTTTCTCCGAGCCGATGCAAATTCTGCGGGCTTTCTCGG
AAGTCCGTCTGCCGAAGCACAAAACAGGGAGCAGTTCATCAAAGCGCCTTA
CTGGAGGAGGTGCAGCCAATAAGGACTTACGTTGTGCCAATTGCAACTCCAA
AGGGCACTTCGCCAGGGAGTGTCTCAAGCCAAAGAGGGAGCCCGGATCCTG
CTATGGCTGTGGGGCATTTGGGCACTTCGTCGGACAATGCCCGGAGCGCAAG
AGCGCCAATATCAACAATTATGTAAGACTTGTCAAGATTTTTTTCACAATAA
CTATAAAACCCCATTAATTACAGAATGCCTCATAGACACAGGGAGTCCAATAT
CATTTACAAAGTTAAGCAGTGTATCCAAAGAAATTAATTTAGAATCCGCTTCA
GGAACCTATTTCGGGCTGAATAAAAGCCAACTGACATTACATGGAAAAAGCT
TATGTTACATTATAAAAGAAAAAGTTAAATGTTATTTTTATTTGTACGTGGTCC
CAGACGAGTCTATGAGTCATAATGTAGTTCTTGGACGAAATTTCATGGAAGCG
TGCCAGTTGAAACTGGATCCAGACGCCTTGGGAAGGATAACGGTCAACCCA
GTCGATAGTAAAAGATATAAAAAAGCAAATAGTAAAACAATCAGTTCTATTGT
TAGTGAAACAGTTAGTGAAACAGTTAGTGAAACAGTTAGTGAAACAGTTAGT
GAAACAGTTAGTGAAACAGTTAGTGAAACAGTTAGTCAAATGGATAATGAAA
CAGTTAATAAGACGATTAGTAAAACAGCCAACGAAGCGAGTAGTATAGTTAG
TCAAACTGTTAACGAAGCAGGTAGCAAAACAGTCATCGAAACAGTTATGCCT
TTAGCCAACTTCACGACGGATTCCGGACCTTATGCTGCGGTTGTGCCCAGTTA
TGAAGAAGCTGTTAGAGAAATTTGTAGCATTGAACACTTAGAGGATGAGCAA
ATGAACTTCATATGCGGCGAAAACAATAACCAAGCCTTAAACAGTCAGTTAA
AGGAGATTATTAAACGCAACTACTTAAGCGTTAATAAGCCTACCGAGCCTTTA
GTCAAATGCGAAATAAAACTTTGTTTAGAAACGTCAAAACCATTTAGCTGCG
CCCCAAGAAGACTTGCCTACTCAGAAAAGGAAAAATTACAAAAACTATTAGA
TGACTATTTAAAAGAGGGAATTATTCAACCTAGTGTATCAGAATATGCTTCAC
CAATTGTTTTAGTCAAAAAGAAAACAGGAGACATACGGCTATGTATAGACTTT
AGAAAACTGAACAAAGTTTTAATTAAAGACAATTATCCGATACCGCTTATCGA
TGACCTTCTAGACAAGCTAGTCAACAAGAAAGTTTTTTCGAAACTTGATTTA
AGAAACGGTTATTTTCACGTATTCGTTAATAAGGAATCAGTCAAATACACCTC
ATTTGTTACACCGTTAGGGCAATTCGAATTCCTCAGAATGCCAATGGGCCTAA
AAACTGCATCGGCGGTGTTCCAAAGGTTCGTAAACAAAATTTTCGAGGATCT
TATAAGGGATAACAAAGTAATTGTATACATGGACGACATCATGATTGCTAGCA
AGAACTCGGAGGAGCATTTAGAAACTTTAAAGGAAGTGTTTATCAGATTAGC
TCAAAATAAATTGGAATTACGAATGGATAAATGTGAGTTCCTTCAGACAAATA

TTAAATATTTAGGATTCATTATAAATGGTGAAGGCATTAGGGCTGATGACAAA
GGAATAGATGCCATACAGCATTTTCCAGTTCCAGACAAAATACAAGCCGTTCA
AAGTTTTCTGGGCTTATGTTCATACTTCCGTAGATTTATAAAAGATTTCTCAAC
ATTAGCGAAACCTTTATATGACCTTTTGAGAAAAGATGAAAAATTTAAATTTG
GAGAAAAGGAAATGAGCTGTTTCTCAACTCTTAAGGAAAAGCTGGTGCAAG
CACCGGTCTTAGCAATTTATAATTTCAAAGACGAAATAGAACTGCACTGTGAT
GCAAGCGCTCTTGGTTTCGGTGCGGTGTTATGTCAAAAAAAAGAGGATGGAA
AATTACACCCAATATTTTATTTTTCAAAGCGAACAACAGTCGCTGAGGCAAAA
TATCACAGTTTCGAATTGGAAACAATGGCAATTATTCACGCACTGCGTAGATT
TAGAATATATTTGTACGGAAAACGCTTTAAAATTATAACTGATTGTAATTCTCT
CACTTTGACGCTTAACAAGACTGAACTTAATCCGCGAATAGCTAGATGGGCG
TTAGAGCTCCAGAACTACGATTATGAGCTCATACATAGAGAGGGTAAAAGGA
TGCAGCATGTTGATGCTCTTAGTAGATGCACGAATATTTTGATAGTGGAATCA
AATACGTTTGAAGACAATTTAGTAATCTGCCAAGGCAAGGATCAAAAAGTTC
AAGAAATCAAGAAACTGCTGGAAAAAAATGAACACAAGTTGTTCGAAATGA
GAAACGGAATAATTTATAGAAAATGTAACGACGGCAGACTTTTATTTTATGTT
CCCACAGACATGGAAGATCATATTCTATATAAATATCACAATGAGATCGGACA
CATAGGTAGAGACAAAATGTTAGATGTTATTTCTAAATCTTATTGGTTTCCCAA
TGCCAAAGACAAATGTTTGAGCCATATAGAAAATTGTTTAAAATGTGTTGCGT
TCTCTCCTAAGACAGGCAAGGAAGGATTTCTGCACTGCATTCCGAAGGGCAG
CAAACCGTTTGAACTAATACATATCGATCATTACGGCCCAGTCGACTCAGGCA
GATCCAAAAAACATATTTTTGTTGTCATCGATGGTTTTACGAAGTTCGTACGTT
TATACGCCACAAAGACCACTAATACAAAGGAAGTCATTACAGCCCTGAAGGA
CTATTTTAGAGCATACAGCAAGCCTAAATGCATCATTTCAGATAGGGGAAGTT
GTTTTACATCCAAGGAATTTGATGACTTTATGTTAGAGTTCGATGTTAAGCATA
TGAAGATTGCGACTGGTTCACCCCAAGCAAACGGACAGGTTGAAAGAGTCA
ACAGAAGCCTAGGTCCAATGATAGCGAAACTGGTACAACCAGAGAAAGGCA
TATATTGGGACACAGTAATAGATAAAGTTGAGCACGTCTTAAATAATACACAG
CACCGCAGCATCAAACAAACTCCAAGCAGAGTTCTTTTTGGTTTAGAGCAAA
GAGGAAAAATAGTAGACGAGTTAAAAGAAAAACTAGAGGAATTAGAAAATA
CTGAAGAAGATAGGGATTTGGTAAAAATTAGAAATGAAGTTGAAAATAATCA
AAAAAAGGCTCAAGCGTATAATAAAACACATTATGACAAGACCGCCAGACAG
CCATCAGAATACAAAAAAGGAGATTATGTGATGGTTAAAAATTTCGACAGTA
CAGCAGGCATCTCTAGAAAATTGATTCCAAAGTGTAAAGGCCCATATGTGATA
TCTAAAGTATTAAGAAACGACAGATTCTTGTTAAAAGACGTAGAAGGTTTTC
AGATATCTCGTAACCCATACCAAGGTGTCTGGAGCATCCAAAATATTAAGCAT
TGGATAGGCAACAAGAAAAATAAACGAAGACACAATTAAACAATAATAATAA
TATAATAATAATAATAACAGATTAATATTGTATGTACATATGTATAAATAAGGTA
AAAGAATTCATCATACAACTAAGCAATCAATGTAAACCATGATCAGGGGATCA
GCTAGTCAGGATGGCCGAGT

>INVADER2_LTRGypsy#LTR/INVADER2_LTRGypsy

TGTAGTAGGCTAGAGGTACACATACATAAGTTCAGTTCATAATAGCAACATAG
TATATAAACATTAAGTACTTGTTCGCTTTCGGGTGGCATTCTCTCTGTTTCGCA
ACATCGGCCCTGTAGCAACAAGCATAGAGGCAGATCGCAAACGGAGAGGGA
GTCAGTCGAATGCTAAAGCTGAACACGGAAAGTAACAAATAAATCAAAGAG
TCCAACTCCTGCCTTTGTGTGTTTTAGTTAATTAAGAGGATATATAATACCCCT
ACA

>INVADER2_IGypsy#LTR/INVADER2_IGypsy

ATATCAGAAGTGGGCCGATCACGAGCCAAAGAGAAAAAAAAAATGAATATA
AGCAATGCATCGACGGCGCAATTAAAAGGATGGTTGAGTGAAGTAAAAGAA
CCAACCACGGGAACAAAAAGAGAATTGATTTTGCGTTTGAATAATTTAACAA
ACGAAAAGCGAAATCAATTGAAATTATTATTAAAAAGCGAGGAAGAAGATTT
CGACGGAAGCAGCAATAATAATGTTCAAAAAGGAAAAAGAAATAACGGAAA
AGACGGAGAGCATAAAGACGACGAAAGTGACAGCGAAGCTGGAGATAGCA
ACGAAGACGGCGATGAGAAGAGCACTGTGCGCAACGACAAAAGTAACGGC
GAACATGACGACAAAAGCGGGGTACGCAGTTACAAGAACAACAGTGCAGAC
GGCGCCCGCAACAACAACAGCGGCGACGGGAATAGCGACGAAAACGAAGT
TTGCGACAGCGAAGACGGCGACGAAGATGGCGGCGGTGAGAAAAGCAACA
AAGAATGTGGCAAGAATAAACGAAATACAGTGGGACAAGCTGTGCAAAATAT
GGATTTATTATTGCGTATGGCAACCGAGGCAGTAAATGAATTTTCGGGAGATA
CATGTGCGCGCAAATGGATCTTACAGGTGAAAAACATAGCCAGCGTTTATGG
AATAAAGGAACCGTATATAAAGATGTTGATTGTCAGTAAGATAAAAGGAAAA
GCATGCATGTGGTTGCATGCAGATCCAGAACGTGTATTGCTACCAACGGAGC
AGTTGGCGGCTGAGCTAATATCAATGTTTGGTGAGAGGAAGTCGAAGTTAGA
AACAAGGCGAAAATTTGAGGAACGAAAGTGGACAGCGAGCGAAAGCTTTG
TTGCTTATGCAGACGATAAGGTGATGCTTGCACATGGAATAAACATGGATAAG
GAAGAATTGGTGGCGCTCCTCATTGAAGGTATTCCGAATCAAATGCTACGTAA
CCAAGCCCGTATCCAATGCTTTGAAGATATCCAGCACATAAAGAAGGCATTTG
CGGAAGTGAAACTACCGAAAGTGGATGAAGCAGACAAGAAGGTGGCATCGG
TGAACAATAATAGCAGCACACTCTTGCGTTGTTTCAATTGTAATTCGAAGGGA
CACTGGGCCAAAGAGTGCAGGAAGCCAAAGCGCGAAAAAGGGTCATGCTAT
GCCTGTGGTGAGATGGGGCACTTTGCAGCAAAGTGCCTGAAAAACAAGAAT
GGGGATGAAAACAATTACGTAAGATATTTTGAAATTAATTTTATGAACAATGC
TAAATCAAAATTTATTACAGCATGCCTCATAGACACGGGAAGCCCCATTTCGT
TCATAAAAATTAGTAAAGTACCCAAAGATGTTATTGGAGTACCAGTTTTAAGT
TCATTTTACGGATTAAACAAAAGTCCCTTGAAAACATATGGAAAAATATTGTG
TTACATTATGAAAAATTTGAATAAAATACACTTTAATTTAATAATGGTGGCAAA
CGAGTCTATGAATCATGATGTAGTATTGGGAAGAGATTTTATGGAAGCATGTA
ATTTAATCTTGAATCTGGACACCTTGAAAATGATTACAGTGGATAAAATTGAA
AGTCGATGTAACAAGAAAAGCGATAAAATGTTAAAGGAAGAAAATATGTACA
ACAATAATAAAACAGTTAGCGATAGTGTTAGTCAAGGTAGTAGTAAAAGTCA
AATGAGTAGTGACACGGTTAAAAATAAATTAGAGAAAGCTGTCAATGCTGAA
ATAAGCAAAGTGGAGAGGGAAATGTTTGAAATTAATGTAATAGATGACTCAA

TTGATTACAAAATAGGTGATCAGGTGGATCATAGTATAAAATGTCAATTTATTG
AGTTTGTAGAAAACTGTTATGTTAATAAAGAAAGACCGAATGAACCGGAGAT
TCGGTGTGAAATACAATTAAGATTAAATGACCTGAAACCGTTTAGTTGTTCTC
CTAGAAGATTAGCATATACTGAAAAAGAAAAGCTGCAGATTATCTTAGATGAA
TATCTAAAAAACGGAATTATTAAACCGAGTGATTCAGAGTATGCATCTCCGAT
GGTGTTAGTGAAAAAGAAAACAGGAGACCTAAGATTATGTGTTGATTACAGA
AAACTTAACAAAACAATGGTCAAAGATAATTATCCTTTACCATTGATTGATGA
TCTGTTGGATAGATTAGTAAATAAAACGATATTCTCGAAGTTAGATCTTAAAC
ATGGATACTTTCATGTTTTTGTTAATAAGGAATCAATGAAATATACATCTTTTAT
GACGCCTTTAGGACAGTTTGAATTTTTAAGGATGCCAATGGGGCTGAAAAAT
GCACCGGCGGTCTTTCAGAGATTTATTAATAGAATTTTCGAGGATATGATAAG
GCAGGATAAAGTTATTATATATATGGATGACATTATGATAGCTAGCAAAGGAAT
TAAGGAACATATGGAAGTACTTAGGGAAGTTTTTGACAGGTTGACGAGGAAC
AAATTAGAGCTGAGAATGGATAAATGTGAGTTTCTGCAATCGAGTGTACAATA
TTTAGGATTTTTAATAACAAGTAAGGGAATACAGGCCAATGACAAGGGAATA
GAAGCAATTAGAGATTTTCCAGTACCAGATAAGGAGCATGGTGTCAGAAGCT
TTTTAGGATTGTGTTCATATTTTAGAAGATTTATTAAGGGTTTTTCAACGATTG
CAAAACCTTTATATGACTTATTAAAGAAAGACAAAGAATTTTCGTTTGAAGAA
AAAGAGTTAGAATGTTTTGAGACACTTAAGAAAAAGTTAGTGGAAGCTCCA
GTGTTAGGGTTATATTGTTATAAGGATGAAATAGAGTTACATACAGATGCAAGT
GCACAAGGTTTTGGTGCAGTGTTATTGCAAAAGAAAGAGGATATGAAATGGC
ACCCTATATTTTATTACTCGAAAGCCACAACAAAGGATGAGGCCAAATATCAT
AGTTTTGAACTTGAAACATTGGCCATCGTGTATGCATTAAGAAGATTTAGAAT
CTATGTACAGGGAAAGAGATTTAAAATTGTTACGGATTGCAACGCATTAAGTC
TAGCGTTAAATAAAGTAGAATTAAACCCTAGGATTGCGAGGTGGGCATTAGA
ACTATTAGAGTACGATTTCGAGGTAGTTCATAAAGCAGGAAAGCATATGCAAC
ATGTGGATGCATTGAGCAGAAATACGAACATATTAGTGATAGAAACTAATACT
TTTGAAGATAATTTAATTATTTGTCAAGCAAAAGATGAAAAGTTGAAAGATAT
TAGGAAAAAGTTAGAGAAAACGGAAGATAAAATTTTTGAAATGAGAAATGG
TGTACTTTATAGGAAGGCAAACGGTGGAAGATTGTTGTTTTGTGTACCAGAA
GAAATGGAAGAAAAAATTTTATATAAATATCACAATGAATTAGGTCACTTAGG
GAGAGATAAGGTTATAGATGCTATTGGTAAGACTTATTGGTTTTCGAATATGAA
AGAAAAAGTTGTTAGGCATATAGGAAATTGTTTGAGATGTGTGGCATTTTCAC
CGAAGTCGGGAAAAGAAGAAGGAATGCTGCATAGCATCCCAAAGGGAAATG
TGCCGTTTGAGATAATACACATCGATCATTACGGACCAGTAGATAACGGTAGA
ATAAAGAAACATCTGTTTGTAATAATAGATGGATTCACTAAATATGTTAGGTTA
TACGCAACAAAAACAACGAACACAAAGGAAGTGATTCTAGCGTTAAAGGAT
TATTTTAGATCATATAGCAGGCCGAAATTTATAGTATCAGATAGGGGGACCTGC
TTTACATCCGAAGATTTTGCAAAATTTATGGGAGAGTATAATATTAAACACATA
AAGATAGCGACAGGTTCACCCCAGGCCAATGGACAGGTAGAAAGAGTCAAT
AGAATAGTAGGGCCTATGATAGCTAAATTAACAGACATTGAAAAGGGATTGC
ATTGGGATGCAGTTATTGAAGACGTAGAATTTTCTCTGAATAATACCAAACAA
AGAAGTGTAGCGCAATCTCCCAGCAAGATGTTGTTTGGAATTGAACAAAAGG

GAAAAGTTGTTGATGAATTAAGACAAAAATTAGAAGAGTTAGATAATGTTAG
TGAAATTAGAGACTTGAAAGAAATTAGAAGAAAAGGATCGGAATCGCAATTA
AAAGCACAGAGGGATAATGAAAAAAGATATAATAATAAGAAAAAAGAAAGT
ACGCAGTACAAAGTAGGAGATTACATTATGGTTAAGAATTTCGATAGTACAGG
AGGAGTTGCTAGAAAGCTAATACCTAAACATAAGGGTCCATATGAAATAGAG
AAGGTATTGAAAAATGATAGGTTTTAATTAAAGATGTTGAAGGTTTTCAATT
GGCGCGAAATCCATATCAAGGTGTCTGGAGTAGTCAAAATATTAAACATTGGA
TAGGGAGTAGAAAGTAAATTTGTATAATTAAAGATAAGAAAAGTAAAAAAAA
TGTAACAAATAGCGGCTAAGAACAATGTAATAGTTATAAGTTGGAAATTTCGA
CAAGATCAGGTGATCTTAAATGTCAGGATGGTCGAAT

>INVADER1_LTRGypsy#LTR/INVADER1_LTRGypsy

TGTCGCAGATCATTTAAATGAAACGAAATTTCGTGTTTCTGCTTGGCACGCGC
CATGCAGACGCCTCTTTTTATTTTTCTGATGCGCGGCAGACAACCGTTAGAGT
TTCTGCCGAACGTAGTCTGGTCGCGGGTAGGAGCGGGGGGGGAAGTAGATG
TCTGTACGAAAACGAGAAGCATACAGAAAAATGCGGTGTGCATAAGTATTGG
TGTATGCGGACTAGAACAACTGTCATAATTGTGTTGGTATTGCATGTAAAGTC
AAGAACTACGCATAATTCTGATTTTGTGAAGAAGAGATCAGTCAGTCAGTTTT
CGATCGTTACGCATAACAGACATGCCTCGCTCAAGCGCCAGAAAACGTCGCG
CGAACCATAACGAGAGTAGTGAAGAGGAAGAAGGGCATCCTGATGACTCTT
CGACA

>INVADER1_IGypsy#LTR/INVADER1_IGypsy

TCAGAAGTGGGATCGCCTATACCTACTTTGAACAATGAAAATAGTTTGCTGAC
AATTTTGGAGTCGCAAAATCGTCATTTTTCCGAACTTTTGAAACAAGTGCAA
CACTCACAAAGAGAGTCAAGAGAATCGTTTGCTGTGGTATTGCCCAGGTTTA
ATCCTGAACGTGCGGGATCAGATGCGCAATCCTGGTGTTCAACGGTGGACTA
CATCTTGTCCGAAAACCAACTGGAAGGCAGCGCTCTTGTGATGACCCTTAGC
AAATCACTGGAAGGAAGCGCGTCTCATTGGCTTTCACAGACTTGCTTTCCTG
GTATATCATGGCCACAGTTCCGAGAATTGTTTTGCTACATTTTGCGGGAACA
GAAACATTAGCGGCAGCCGTTATGAATCTGCTAAATGGACGCCCTGCGGAAG
GCGAGTGCCTGTCGTTATACGGAAGCCGAATGGTTACTTCGCTTATGGCCAAA
TGGAAGACGCTGAGCGTGGAGCAAATCGCAGTCTCCATTACACTGGCGCATG
CAGCTGGAATTGACAGGCGTTTGCGGCGTTTAGTTTTCACTTCCGACATCAA
CACTCGGAACGAGCTGAACCAAGAACTAAAGGCCTATTCATTCGACGCTAGA
CCGAGCCACCTTCCAAACGATGGTACATCAGAGCCGCCAGGAAAACGAGCT
AAGCCCTACTTCAAGTGTCACAACTGCGGAAAGCCTGGTCATAAAAAGGCG
GATTGTCGATCAAAAACAGTGGCAGTGCACGTTCAGCAACCCGACAGGCAA
CCATCAGGGAAAGACCGGTCTACTTTGACTTGTTTCAACTGCGGAAAAGCTG
GACACATCTCCACCGTATGTCCTGATCGTCAGGAGCGTTCATCGTCTAGCAAA
AATAACATTGTTTAAGATGTCAACATCTGTACCGTTTTTGAGCCCGTACGGAC
GTTTTCGCAATCTGGTGAGTCGTTCCAATTTTGTTTTGATTCTGGAGCCGAGT
GTTCACTCATCAAGGAGGCTACTTCGCAGAGACTATCTGGATCCAGAATGGG

CAACGTAGTTATTTTAAGGGGTCTTGGTAGCAATACTATTTGTAGCACTTTGC
AGATATTGGCTAATGTTTTGATAAGTGAACGCTCATATGAAATATTGTTTCATG
TAGTGTTAGACAATTATATTAAGTATGACGCTCTGATTGGTCGAGATATTTTGA
GCCAGGGAGTCGGTGTGACAATAACATCAAAATCTCTTACTATGTTTAACGAA
AAATCTATATTGTCTGTTGAAGTTTCCGAAAAGAATCCCGATTTGGCTCATGT
TGACTTTCTGTCTCGGAATCCGGTTCCAAAAATTGTCAAGAATTCCATAGGCG
TAGTCGAAAGGCATATCAATCTGACAGAAATATCCGACAACTGGTTATTGGCT
GAACAGCAAAGAGACGAAGAAACTTCTTCCATTATCTCTAAATTGCGTAATA
ATGAATTGTCCGAGGATTTGGCCAAGACTTATGAACTACGGTCTGGAACTATG
TACCGAAAAATTCAAAGAAATGGAAAGACTCGATGTCTGCCCATAATCCCAA
AGCAGTTTAGGTGGTCCGCTGTCAACAACGTACACGAGTCAGTTATGCATCT
CGGGTGGGAGAAAACTCTTGAGAAAATGTACGAGTTCTATTGGTTCGATAAA
ATGTCTAAGTATGTTCGCCAATTTGTGGACAACTGTATCACTTGTAGATTATCA
AAACCTCCGTCTGGCAAAATACAATCCGAACTCCATCCCATTCCTAAAATTGA
CATCCCGTGGCATACGGTTCATGTGGATATTACAGGTAAACTTAGCGGAAAAA
GCGACCAGAAGGAGTATATAATTGTGATGATAGACGCATTTACCAAATTTGTC
TATCTCTCTCATACTACGAAGCTAGACACCGATAGTTGCATTAAGGCAGTGAG
GTCTGTTATATCATTATTCGGTGTCCCCTCCCGACTTATTGCTGATCAAGGTCG
TAGTTTTGCTAGTTCAGCTTTCCGTGATTTCTGCTCAGCACAGAAAATGGATT
TACATTTAATTGCCGCAGGTGCTAGTCGTGACAATGGCCAAGTGGAGCGAGT
CATGAGTACTCTAAAGTCCATGCTGACTGCGGTTGAAACTGGCCCAGGATCT
TGGCAGGATTCTTTATATGAGATTCAGCTGGCCCTTAATAGCACGCCCAATAG
AGTTACTAAAGTCAGTCCATTAGAGATACTTATTGGCAGAGAAGCAAGGCCA
TTTGGGCTTACACCTGTTGTAAAAGAACAAAATATTGTCGATGTTGGCTTAGT
TAGAGAAATAGCAAAACGCAACATGGAAAAGAATGCCAAAATTGATAAAGC
TAGATTCGATAAAAACAAAGCCACTATTGTAAGACACAAGCTAGGAGACCAT
GTTTTACTTAAAAATGAAGAGCGTCACCAGACCAAATTAGATCCTAAGTTTAA
AGGTCCATTTGTAGTGGCCGAAGTATTACCAGGAGATCGATATAAACTCAAAG
CATTAAATAATAACCGAACTTATAAATATTCTCATGAATCTTTGCGATGTATGCC
TGAGAAGAGGATTACCACAGTATTTGAAGATGAAGGCACAGAAGATGCCAG
TGATCAAGCCAGTGAGGGCTGTGATCTTGAGAATGACTAAAAATTTGTAAAC
CTCCGGACCTATTCCAAATGCGACTTTCTCTACCTCATGGGTGCCCGAGTTAA
GTGGTTCGCCATTTTGTTGCAGTTAAGCTTAAATTTGGTAACGGTTGGTGGTG
TGAGTTAACTGGCCGGCTTATAGACTGCGTCATCTGGGTGCCACGTATGTTGG
CCGGATTGTCGAGTTGTAAGCTGTGAGCTAGGCTAACAAAAGATGGACTGGC
CGGTTTGAAAAACTGTGCGCTAGTAGAAAAATGTATTGAAAATCAAGAACGT
TAACGATTTATGTTTGAAGAAGACAAAATTATGATTGTTTGATTGAAAGCTGA
TGGTGGTCCGGTTCTCATGGCTTAAATGTAATAAATGAAATGTTTTAAATATTT
GTATTGGTTTGATTTAAAGTAATAAAGAATGAAGAATAACTTTATATTTTGAAA
AAAAAAATAAGCCTTTGATAGATTCTGTTTAATTATTGAGAGACTGTTAGCAC
ACGAGGACGTGTGATAGGTCAGGAAGGCCG

>IDEFIX_LTRGypsy#LTR/IDEFIX_LTRGypsy

AGTGACATATCCATAAGTCCCTAAGACTTAAGCATATGCCTACATACTAATACA
CTTACAACATATACACCCCAATACAACATACACTACTCCGGATGTACCCAACA
GATACCAGAAAGAATAAGATTGTTAAAAAAACCCCAATTCTAGATAAGTCAC
CCACTGGTAGACTAAACATCCGTCCCCTAATTTAAACAATTCCTTGCTTAAGC
CTCACCCCATCGTCACATTCCCACGTTCAAAGCTCGGAGCCGCAATCCCGAA
AAACAAAAGTATCGATTTCAATAAACAAATTATAAGAATCTAAGAGCACTTGT
ATCCAAGAGCAAATGCACTTGAATCCAAGAGAAACGCAAAGCTTTTTCTCTT
CACGATCAGAATCCTAAAGTCTAAAGTCCATATTAGAAAAGCTCGATACCGA
GGCTTGAACGTCAATCAAATCAGAATAATTATCAGAGTTCAGTTTGAGACCTA
ATTGTAAAAGGTCGGTGTTCTTCTCAAATAAAAAGATTGTAATCATTTAGTGA
AATAAAAATTATATTTTTTTCACTTATAAATATTGCAAGTATTTAATT

>IDEFIX_IGypsy#LTR/IDEFIX_IGypsy

GGCGCAGTCGGTTAGGATCCAATAAAATAAAAGAGTCCTTTTAGTACGGTACT
GATCAACTGAAGGATATGCTATACGACTAGCTATCCAAGATCAGCGAATTAAA
ATAGTGATTCAAAAATATTTTTTAATCCGCAAAAGAATCTACGTGAAAGTAGT
ATTCAAAATAAAATCCCGTGCGGTCGGAAACAAAAATTAATTTAAATTTTTTA
ATTCCGAAACTTAAAACCAAGTTTAAAGAAAACTTAAAATCAAGAAAACTTA
AAACCAAGTTTAAAGAAAACTTAAAATCAAGAAAACTTAAAACCAAGTTTA
AAGAAAACTTAAAATCAAGAAAACTTAAAACCAAGTTTAAAGAAAACTCAA
AATCAAGAAAACTTAAAGCCAAAATAAGCTAGAAAACTAAAAGACATCATG
GCAGTCCCACAACTCTCAGAAACACACCTAAACCAACTGCTAAACCAAATC
AAAGAATTAAACTACTACGATGGCGCACCTGGCAAATTATCTGGATTCGTCAA
CCAAGTGGAACAACTGCTCAGTTTATACCCAACACAGGAAGCAAGACAGGC
ACACGTCATATATGGAGCAGTGAAGCGGTTATTAGTGGATTCAGCCTTAGAAG
TCGTAACCCAGGAAAGAGCTAACACATGGCTGGACATGAAGAAAGCACTGG
CAATGGCATTCAAAGACCATAGACCTTATGTAACTCTCATCAGACAATTAGAA
GACATATCATACCCAGGAAGTATCTGTAAGTTTATAGAAAAATTAGAAACACA
ATACTGGATTATGTTCGATAAGTTAGAATTAGAAAGTGACCATGTTGATAAATC
GAATTATACCGAAATGTTAAACAAAACTGTTAAATCAGTAATAGATCGAAAAC
TGCCGGATAGAATTTATATGTCTTTGGCACGTAAAGATATTGATACAATTTATA
AATTAAAACAAGCATCAATGGAATTAGGCCTTTATGATGCTATTCCAGAAAAT
CACCGTTCTAATAGAACAGAAATGAATAAACGTAGGAACAGGGGAAACTATA
ATCAAAATAATAATCAAAAATATTACAATAATAGAAATCACAACTACAGTAATT
ATTATCCTAGCATGAATCAGAATCATAATACACAACCACCTCAGAATCCGACT
CAACCTATGACAAATCAAAACCAATATTCACCGCGTTTCATACCGAATAATCA
AAGAGGGAATTATTATGCATTTAGACGAGACTTAACACAAGCTCAGCAGAAC
AACCCACTTAATAACACCCTTAACTTCCAACCTTCGACATCGAATAATATTAA
CAGACAAGGGCCAGTAAAAAGACAACGCGAGAGTCAGAGTGACCAAAGCA
GGATGGATGTAAATTTTCATCAAGCTGCCTCGGACACTCAAATGATAGAGAA
GGACATACAAGTCCCTATGTAAAAATAATTCATCATAATAAAAATTATAAGGGA
ATGATCGATACAGGATCATCAATTAACATCATAAGAGAAAATTTTGAGAACTT
AGAAGAAAAGGAAGAAAACCTAATAGTATACACTATTAAAGGACCAATAACA

CTAAAGAGAAGTATAATAATAAAACCTACTTCAGTATGTCCGTCTGCTCAAAA
ATTCTACATTCACAAATTTTCTGATAACTATGATTTCTTGTTAGGTCGAAAGTA
TTTAGAAGATACAAAAGCTAAAATAGATTATGCTAACGAAACAGTAACACTA
GGCTCAAAAGTATTTAAGTTTCTCTATGAAGAAAAGAAGGGCGAGACCGCAT
CCAAATGCCTTGACCCACAAGAAAAGAATGATTCCGCTCTAGTGGACAGAAC
CAAACCAAAAATGCAAAAGGTTAAGACCGCACCTAAGTGCCTTAAACCAAA
GCATCAACAGCAGAAGAAAGAGACCGCATTACCCAAATGCCTCATTTCAAAT
GTTGTTAAAGACACAGTGGACAATGATGTAACACATCTCGATCCCATGTCCGT
TGACAACGATATAGTCAACTTCGCGATTAACAATGAGTTACGCGAATGTAACG
AGTATAGACTCGAACACTTAAATGCAGAGGAAGTTGAATGTTTAAAGAAGTT
CCTATACGAATATAGAGACATTCAGTACAAAGAGGGCGAAAATTTGACCTTCA
CCAGTACTATTAAACATGTCATCCAGACTCAACACGAAGACCCAGTATACCGT
AAACCCTACAAGTACCCTCAAAGCGTTGACCAAGAAGTTAACAAACAAATTA
AAGAAATGATAGAACAAGGGATTGTTCGCAAATCGAAGTCCCCTTATTGTTCT
CCTATTTGGGTGGTCCCCAAGAAGGCAGACGCCTCTGGGAAACAAAAATTCA
GGTTGGTAGTCGATTACAGGAACCTAAATGAGATAACTGTTAACGACAAATTT
CCCATTCCCCGAATGGATGAGATATTGGACAAACTAGGTAGATGCCAATACTT
TACCACTATAGATCTAGCCAAGGGTTTTCACCAAATCCAAATGGATGAAAATT
CTATTGCAAAAACAGCTTTTTCAACTAAGCATGGGCATTATGAATATACTCGTA
TGCCCTTTGGTTTAAAAAACGCTCCAGCTACTTTTCAGAGATGCATGAATAAT
CTTCTGGAAGATTTAATCTACAAAGACTGTTTAGTCTATTTAGACGATATTATT
GTTTATTCCACTCCATTGGAAGAACACATTTTATCCCTAAAGAAAGTCTTTGA
AAAACTGAGAGACGCTAATTTAAAGTTGCAACTAGATAAATGTGAATTCATG
AAGAAAGAAACTGAATTCCTAGGACACATCGTCACAACAAATGGCATCAAA
CCAAATCCAAATAAAACTAAAGCAATTACAAATTTTCCATTACCCAAGACACC
TAAGCAAATAAAATCATTTTTGGGATTATGTGGATTCTATCGCAAGTTTATTCC
TAACTTTGCCAAAATAGTTAAACCCATGACCCTCAAATTAAAGAAAGGTGCT
ATAATAGACACCAAATGTAAAGAATACATCGAATCATTTGAAAAATTAAAAGT
TTTGATAACTTCAGACCCGATATTAATCTATCCTGATTTTTCAAAACCTTTTTCT
TTGACAACTGATGCTAGCAACGTAGCTATTGGTGCAGTGTTATCACAAAATCA
CAAGCCAGTTTGTTATGCCAGTAGAACGCTAAACGAACATGAAATCAACTAT
GCTACGATTGAAAAAGAATTGTTAGCTATAGTTTGGGCTACAAAATATTTCAG
GTCATACTTATTCGGCAGACCATTTGAAGTATTAAGTGATCACAAGCCACTGG
TATGGCTCAACAACATTAAAGAACCAAACATGAAATTGCAAAGATGGAAAAT
AAAACTTAATGAATTCGATTATAAAATCAAATATCTTCCAGGCAAAGAAAACC
ATGTCGCGGATGCTCTTTCCCGCACGAAAATAGAAGTTATGGTTGGCGAGGT
CGCAAATAGCGCAGACGCAACTATACACAGTGCCATTGAAGATAATCTAAATT
ACATACCCATAACAGAAAGACCAATAAATTACTTCTCTAGACAAATAGAGATA
GAAAAAGGCGATAACGATACAACAAGTGTACAACATTTGTTTCAAAAATTAA
AGATTAAGATAGTCTATAAAGAAATGACACCTGAACTCGCCAAAAACCTCAT
TAAGGAATATGTGTGCACCAAAAAGAGTGCAATTTATTTCCCTAATGACGAAG
ATTTTCTGATCTTCCAGAGAGCGTTTACCGAAATTATAAGCCCTAACAATTTC
ACAAAACTCTTGAGATGTACCACAAAGTTAATTGATATACTAACGTATGCAGA

ATTCAAAGATTTAATCTTAAAGAAACATAAGGAACTTTTACATCCGGGTATAG
AAAAAACAATCAATTTATTTAAAGAAGAATATTACTATCCTGATAGTCAAAAG
CTTATTCAAACCATTATCAATGAATGTCAAATTTGTTATCTAGCAAAAACGGA
ACATCAAACACAAATGACATATGAGACTACACCAGAAATATTTAACACAAGA
GAAAAATACATGATAGATTTTTATCTCACAGGAAACCAGATCTTCTTATCTTGC
ATTGATATCTATTCGAAATTTGCATCACTAGTTGAATTAAAAAGTAGAGATTGG
CTAGAAGCAAAAAGAGCCATTACTAAAATATTCAATGACATGGGAAAACCGC
AAGAAATTAAAGCAGACAAAGACTCAGCTTTTATGTGTTTAGCCTTACAAAA
TTGGTTAAGATCTGAAGGTGTACAAATTTCTATAAGCACTAGCAAAAATGGTA
TATCTGATATAGAAAGATTCCACAAGACCGTAAACGAAAAGCTAAGAATCATT
GGTAGCCAACAAAATGTTGAAGATAGGTGCACAAAATTCGAAAGAATTCTAT
ACATATACAATCACAAAACTAAACATAATAGTACTAAAAGATTTCCAGCAGAC
ATTTTCCTATATGCAGGCAGTCCAGATTTTAATGTACAACAAAACAAAATCGA
TAGGATAGAATACCTCAATAAGAATAGACACGATTTTGAAGTTGATATAAAAT
ATAGACAAGCCCCACTTGTAAAAAGTAAAATAACCAATCCATTTAAAAAGAC
AGGAAGAATTGGACAAGTAGATGATAAACATTTCGAAGAAACAAATCGTGGC
AGGAAGATCGTTCACTATAAGTCAAAATTTAAGAAACAGAAAAAGTTTAATA
AGAGCAAATATGATAATTCCAGACCAACCAAAGAAGCACAAAGTACACAAC
ATACTTCTAATAATGCTTAGTTGCATACTATCACTTATCATCACGGTCAAGTGC
AACAATATAGAAGTAAATCCAGTAAACGCGAAAAATGGATACCTTATATTCCA
AACAGGAACAATGGAAATTCCAACCAGCTATGAATACCATTATTTAAGCATAA
ACATAACAAAGACAATGCTCATGTTCGAAGATATAGTAAGTGAAGCAAACAA
CTATCCTAATGTACCACAAATACAATATTTAGTCGACAAATTAAAACGAGAAA
TAAATGGGTTAAGAATTATTAGTCGAAGTAAAAGAGGTCTTTTAAACGTAGTA
GGAAAAGCATACAAATACTTATTCGGCACATTAGATGAGGATGACAGAGAAG
AGTTAGAAGAAAAAATAAACAACATGTCAGAAGACTCTGTAAAAACCCATG
ACCTAAACACGATTCTAGATGTAATCAATAGTGGTATAGATATAATTAATAAGC
TCAAAGTAGATAAAGAACAACACCAACAAATTGCGGTACTAATATTTAACCTA
GAGCAATTTACAGAATATATAGAAGACATAGAATTGGGTCTGCAATTAACCAG
ACTAGGAATTTTCAATCCAAGATTACTAAAGCATGACTATTTAAAACATGTAA
ATTCAGAAAAAATGCTAAAGATAAAAACGTCAACCTGGCTTAAAACAGACAC
GAACGAAATTTTGATTATTTCCCATATTCCTAGCGAAGTTACTAAAGTTCCAAT
ATTCCAAATTGTTCCGTACCCAGATGAACATAATTATATTCTAACCGAGCAAAT
ATTCGATAAATTCTACATATTTGATAACCAAGTATTCCATAAAGATACCAATAG
GGATATATTCGACAAATGTATTATTGGAATCATCAAACAAGAGCAAACTCAAT
GCAAATATATTAAAACACATAAAAATTACCAAATAAATTATATAGAACCAAATA
TACTATTAACATGGAATATTCCTGAAACAGCTGTTAACCAAGACTGTACACAC
AATAAAATATTAATTTCAGGAAACAACATCATTAAAATTAAAAATTGTACCATA
CAAATAGATGAATTCTTAATCTCTAATAATCTAGCAGACTTTACACAAACAATT
TATATCACCAACAATGTAACACGTCTAGAACCAATAAATCACTTACAAACGAG
AGAAATGATAGAAACCCATGTAAAACACTATAACTTTTTTCAAATTATATGCAT
TACAACGTTCGTCATAATGATAATTAGTTTGACTCTGTATGTAGCATATAAGTT
TAAAAATATACCTAAGAAAATTATTGTCAATATCGTAAGCAAAAAGAACACAC

GCACCTTGAAAATAATGTCAATGAAAATATTCAACAAGGAAATAATATTACCT
TATACCCAAATTTAACGACCTGAGGACAGGCCAAATTCAAAGGTTGGGGA

>I_I#Unknown/I_I

CATTACCACTTCAACCTCCGAAGAGATAAGTCGTGCCTCTCAGTCTAAAGCCT
CGCTTCGCGTAAGCCCAAAACTCTTATCAGCAAAATCTTGATAAACAAATATC
AACCACAAAGAGAAAATAAAAAACTTAACAACAAAAACAACAATACCGCTA
ATCCGGGCTCAAGCCCTTAACCAACAATCATGACAGACCCACCAAACATTTA
CAAAATCACTTCAAAAACATACCAATCCCAATTAGGCGAACCTAAATTTATAA
TTATTAAAAGAAATGACAACAACTCTTTCGAAAGAACTTCACCATTCATCATA
AAAAAATCGGTGGACTTTGCCTGTGGAGGAGAAGTTGAGGGATGCAAACGT
ACAAGAGACGGCAACCTGCTAATAAAAACCAAAAATGAATTACAAGCCAGA
AAACTCCTAAAACTAACAAAAATTGCAGATGAGGATGTAACAGCAAGTGAA
CATAAAACATTAAACTTCTCTAAGGGAGTTATTTACTGTAACGACCTTAGACA
CATCGACGAAGACACAATTCTACAAGAACTAAAACCACAAAAAGTATCTGAA
GTTAAAAAAATAATGAAACGGCAAAACCCCAACTCTAACTCCGACACCAAC
AACATCACATTAGTTGAAACTGGACTCATAATTATAACCTTTGAATCGCATAA
GCTCCCCGAGATAGTACGAATCGGGTACGAAACAGTCCGAGTACGAGACTAT
ATCCCACTCCCACTTCGATGCAAAAAATGCCTCCGCTTCGGTCATCCAACACC
CATATGCAAAAGTGTAGAAACTTGCATCAATTGCTCTGAAACAAAACACACA
AACGACGGAGAAAAATGCACAAACGAAAAAAACTGCTTAAATTGCCGAAAT
AACCCAGAACTTGACCATCAACACAGCCCAATTGACCGCAAATGCCCTACGT
TCATAAAAAACCAGGAATTAACAGCAATTAAAACCACACAAAAAGTTGACC
ATAAAACGGCCCAACACATATATTTCGAACGTCACGGCTTCCAAACGAAAAA
CACCTACGCCAAAACACTTACAAACGGCACAACCCAGAGGACAACAAACAC
TCCATCACCTAATATTCACACAAACACAACCCAATCACAACAACAAAATCCG
CACCACACACCCAAATCAGCAGCACAAAACACTTCAGCTAAGACACCAACA
ACTGAACCAGCCAAAACAACCTTACTATCCAACCAACCACACCAACACCAC
CACCACCACAGCTACGACAAACTAGAAGACATGGATACCGACTACACACCTA
CCAGAAAACCATCTACGACATACTCATCACAACTCACAGAAGACCTAAAAAT
AAAAATCTTCCCTAAAGATAAGTCCAATAACCTATCCATAAACCTTAAAGCAT
CAAAACTAAAGGCCAAAGCCCACAAAAACAAGCACACTAACAACAGCGAC
AGCGAATCCATATAGAACTCTACACAAAACCCTAACCGTTAACACTACCTTTA
AGTAAGTTATAAGCTTTAATTTTCTCACAAATGTCCCTAACTATAATCCAATGG
AATCTAAAAGGATATCTAAACAACTACAGCCATCTCCTTATTCTAATCAAAAA
ATACTCCCCCCACATAATTTCCCTCCAAGAAACCCATATACAATACACTAATAA
CATTCCAACCCCAATAAACTACAAACTATTAACAAATATTGCCACCAACAGAT
TTGGGGGCGTACGACTACTAGTGCATAAGTCAATACAACACACTGTCCTCAA
CATAACAATCGATATAGAAGCAATAGCCATAAATATAGAATCTAAACTTAAATT
AAACATATTTTCCACATACATTTCTCCGACCAAAAACATAACTAACCAGACAC
TCCATAACACATTTAACATACAACAAACACCCTCTCTAATTACGGGAGATTTT
AATGGATGGCACCCATCCTGGGGCTCCCCAACAACAAATAAACGAGGAAAA
ATAACTCATAGATTCATTGACAACATGCACCTTATCCTGTTAACGACAAATCT

CCCACACACTTTTCAACACACAATACATACACACACATAGACCTCACACTCTG
CTCTCCAATCCTAGCCCCCCACGCCAAGTGGAAAATACTAAACGATCTTCAC
GGTAGCGACCATTTCCCTATTATCACAACACTATTCCCAACAACCAATCCACA
AAAATTCTACAGACCCTTTTTTAAACTCAAAGAAGCCAACTGGGAACAGTTC
AACGCTCTTACCCACCAAACCAACAAGAAATACCCCACCTCCCACAACGTAA
ACAAAGAAGCCGCTCTAATCAATAGAATCATCCTTTATAGCGCAAACCTCTCC
ATCCCACAAACCTCACCTAACACACATCCATACAGGGTTCCATGGTGGAATAA
ACACCTCGACCAATTACGTAAAGAAAAACAACTTGCCTGGAAAAAATTAAAC
CGCACAATTACTGTTGACAACATTCTAGACTATAGACGCAAAAACGCAATATT
TAGATACGAACTAAAAAAGAGGAAAAAAGAAGCTTCCAGCTCTTTCACCTC
AACCATCCATCCCACTACTCCCTCATCCAAAATATGGGCCAATATAAGACGCT
TCTGCGGACTTAACCCAGCAAAACAAATTCATGCCATCACAAACCCAGTAAA
TAACGAGACTACATTGGCTAGCAACGAAATTGCTAACATATTCGCACAACATT
TCTCTGACCTCTCCGGCGACTGGAACTTCTCAGAGGAGTTCCGGAACAATAA
ATATAGAAATAACATACATCTCTACACCCCCTCTCCAATAGCCCAAACCATAG
AAGAGAACATAACGTATCTAGAACTTAGCTCAGCACTACAAACATTAAAAGG
ATGTGCTCCAGGACTAAATAGAATCTCGTATCAAATGATCAAAAATAGCTCCC
ACACAACAAAAAACCGAATAACGAAACTATTTAATGAAATATTCAATAGCCA
CATACCTCAAGCCTACAAAACAAGCCTAATCATCCCAATCCTTAAGCCAAAC
ACCGACAAAACGAAAACTTCCTCATACCGACCCATCTCCCTCAACTGCTGTA
TAGCAAAGATACTTGATAAAATAATTGCGAAAAGACTCTGGTGGCTAGTGAC
ATATAACAACCTAATTAACGACAAACAATTCGGGTTCAAAAAAGGCAAATCG
ACTTCGGACTGTCTACTCTATGTAGACTATCTCATAACGAAGTCAAAAATGCA
CACCTCCCTCGTCACTCTTGATTTTTCAAGAGCCTTCGATCGAGTAGGTGTGC
ACTCCATAATCCAGCAATTGCAGGAATGGAAAACGGGTCCCAAAATAATAAA
ATACATTAAAAACTTCATGAGCAACAGAAAAATAACTGTCCGCGTCGGTCCG
CATACATCAAGCCCGTTACCCCTATTCAACGGAATCCCCCAAGGTTCACCCAT
ATCCGTAATACTTTTCCTCATAGCATTCAACAAATTATCCAACATCATATCCCTA
CATAAAGAAATTAAATTCAACGCATATGCCGACGACTTCTTCCTTATAATAAAT
TTCAACAAAAACACAAATACAAATTTCAACTTAGACAATCTATTCGACGATAT
AGAAAATTGGTGCTCCTACTCAGGGGCATCGCTTTCCCTATCCAAATGTCAAC
ACCTCCACATATGCAGAAAACGTCACTGCACATGCAAGATAAGCTGCAACAA
CTTCCAAATTCCTAGCGTTACGTCCTTAAAAATTCTAGGAATAACCTTAAACA
ACAAATACAAATGGAACACACACATAAACCTACTTCTACCCAAACTACACAA
CAAGCTAAATATAATAAAATGCCTATCTAGTCTTAAATTTAACTGCAACACGCA
TACACTACTTAATGTCGCAAAAGCAACAATTATAGCCAAACTAGAGTATGGTT
TGTTTCTGTACGGCCATGCTCCCAAAAGCATTTTAAACAAAATAAAAACACC
GTTTAACTCCGCTATCCGTCTAGCTCTCGGCGCATATCGCTCTACCCCAATAAA
TAACTTACTTTACGAATCGAATACTCCCCCCTTAGAAATGAAACGAGACCTTC
AAATAGCCAAACTATCCCAAAACCTAATCCTCTCCAAAAACACACCAATACAT
AAGTTCTTAAAGCCTAAAAAAGCTAATAAGAAAAAAACATCAACAATAGACC
GAACAATCAAACTTAGCCTAGAACTTAATCTACCCTACAAACCAATAAAACTC
CATAAAAACAAACCACCATGGACCCTCCCCAATCTAATAGACACGTCACTTA

GAATCCATAAGAAAGAACAAACATCTCCAGACCAATACAGAAAATTATACGA
ACACACAAAGAATAACCTCAAAACACACAATTTCATATTCACTGACGGTTCA
AAAATTAATTACACAATATCATTCGCCATTACAACGGAGACAGACGTCTTGAA
ATACGGCATACTGCCCCCATATTCATCCGTCCTCACCTCCGAAACAATCGCCAT
CCTAGAAGCAATAGAACTTACTAAAAACCGAAGAGGCAAATTTATTATCTGCT
CCGACTCCCTATCAGCAGTAGATTCAATTCAAAACACAAATAATAACAGCTTT
TACCCAAGCAGAATACGATCGCTAATAACGCAACACGCACCTAAAATTAAAA
TAATGTGGATTCCTGGCCATTCAGGAATAAAAGGAAATGAATTAGCCGATCAA
GCTGCAAAATCAGCAAGCAGTATGCCACTTATCCTCACCCCAAACATAAATAC
CACAGATATAAAAAAACACCTTAAAGCCGACCTTGCGACAAAACAGAAAGA
ACACATAATAAACTGCAGTCCATGGTACCAATCTATTAACACGAACACCTCAC
ACCCATGCGATTACCTTAAACAATCCCACCCAAATTGGACCAGACTCGACCA
AATAAAAATAATACGACTTCGACTAGGACACACAAACATAACCCACCAACAC
TACCTAAATCCCAATTCAATACCAACTTGCCCGTTTTGCCAAGGTGATATTTCT
TTAAACCACATATTTAACTCATGCCCATCCCTCCTACAAACCAAGCAAGATAT
ATTTAACAACACCAACCCTCTAGACCTTCTTAGCAAACCCAATCCAGATAACA
TACAAAAACTCATACTTTTCCTCAAAAAAACTAAATTATACCACAAAATCTAA
AAACAAAACAGGCATTTGTACATAACAAGCCAGCAATTAGTTACCAAATTAG
ATATTAACTAAATTAAGATATAATAACATTGTAAATAAATATAGCTGTAAGCCCC
GTAGCTAATGCTATACTATCTAAGTTAGTCTAGTTTTGTAAACTATTCTATCTAT
CATAATAATAATAATAA

>HOBOhAT#DNA/HOBOhAT

CAGAGAACTGCAAGGGTGGCACTTTTTTACCACTCGACTCACACCCTACAAT
TTTGTGTGCGGGTGCTACTCGCCACGCACATCGCGGGTACTTACAAACACAC
AGTATAAATCTGAACATGCAGACAAGACACCCCGTTGTGTGCGCACCCGAAT
CAATACGGTGTTTTGCGTCGCGGGTGCCGCTCACACAGTGCCTAAAAAGGGA
TGAGTGAGAAAAACACTTGTGGGTATACCGTTAAACACATGGGTGTTTCCAA
AAATACTCGGGTGTTTCCAAAAATACTCGAGTGGTCTCGTAGGTAGTCGAGT
CAAATGGCGCCATACATAATGATTGTTGAGTTCTTGTGTCTTTGGTCCAGTGT
CTCGGCTGTTAATTGCCCCTTTTTTGTTTTTTACGATGCAATTACTAGCTTGTT
AGGATTCAGTATTATTTGGAAGCCAAAGGAAAAGGTCACAATAATGGCAGAA
GCGGCTGATTTCGTTAAAAATAAAATTAACAATGGAACATACTCAGTTGCCAA
TAAACATAAAGGGAAAAGTGTTATTTGGAGCATTTTATGTGACATTTTAAAGG
AAGATGAAACTGTTCTGGACGGATGGCTGTTCTGCAGGCAATGCCAGAAAGT
GCTCAAATTTTTACACAAAAACACCTCCAATTTATCCCGCCATAAATGTTGTC
TAACATTAAGACGACCAACGGAATTAAAAATTGTTTCGGAAAACGACAAGAA
AGTAGCTATTGAAAAATGCACCCAATGGGTTGTCCAAGATTGTCGGCCGTTTT
CTGCAGTAACCGGAGCCGGATTTAAAAATTTGGTGAAGTTTTTCCTACAAATC
GGCGCTATCTATGGGGAACAGGTAGACGTCGATGACTTACTACCTGATCCAAC
AACATTAAGTCGGAAGGCCAAATCGGATGCAGAAGAGAAGAGGAGTCTAAT
CTCGTCCGAGATAAAAAAAGCTGTGGATAGCGGAAGAGCAAGTGCGACCGT
CGACATGTGGACTGACCAGTATGTCCAAAGAAACTTTTTGGGCATCACTTTC

CATTACGAAAAAGAATTTAAACTTTGTGACATGATTTTGGGACTAAAATCGAT
GAATTTTCAAAAATCGACTGCCGAAAACATTTTAATGAAAATTAAAGGTTTAT
TTTCGGAATTCAATGTTGAGAACATTGATAATGTTAAGTTTGTGACTGACAGG
GGAGCAAATATAAAAAAGGCTTTAGAGGGCAATACCCGTTTAAATTGTAGCA
GTCACCTGTTGTCAAATGTTTTAGAAAAATCGTTTAACGAGGCCAATGAACT
CAAAAAAATTGTGAAATCATGCAAAAAAATCGTGAAGTACTGCAAAAAATCA
AATTTGCAGCATACTCTAGAAACCACTTTGAAAAGCGCCTGTCCGACTAGAT
GGAACTCCAACTACAAAATGATGAAGTCCATTCTGGATAACTGGCGTAGTGT
GGATAAAATATTAGGTGAAGCTGATATCCATGTAGATTTTAATAAATCATCTTT
AAAAGTTGTGGTAGATATTCTAGGAGACTTTGAACGAATATTTAAGAAGTTGC
AAACATCTAGCTCACCATCTATATGCTTCGTATTGCCATCCATCTCTAAAATTTT
AGAATTATGCGAGCCGAATATTTTAGACCTTTCTGCAGCAGCATTGCTTAAGG
AAAGAATTTTGGAAAATATTCGTAAGATTTGGATGGCAAATCTAAGCATATGG
CATAAGGCGGCATTTTTTTATATCCACCCGCAGCACATCTTCAGGAAGAAGA
TATTCTTGAAATAAAGGTGTTTTGCATTTCACAAATTCAAGTCCCAATTTCATA
CACATTAAGCTTAGAATCTACAGAAACTCCAAGAACTCCAGAAACTCCAGAA
ACTCCAGAAACTCCAGAAACTCCAGAAACTCCAGAAACTCCAGAAACTCCA
GAAACTCCAGAAACTCCAGAAAGTCTAGAAAGTCCAAACTTATTTCCAAAA
AAAAACAAAACAATATCTTCTGAAAACGAATTCTTCTTCCCAAAGCCAGTAA
CTGAGTCTAATTCCAACTTCAATGAATCTCCATTAGATGAAATTGAACGATATA
TTAGACAAAGAGTTCCATTGTCTCAAAATTTTGAAGTAATTGAGTGGTGGAA
AAATAACGCAAACTTATACCCTCAGTTGTCAAAGTTAGCATTAAAACTTTTAT
CAATACCAGCCAGTAGCGCAGAGCTGAAAGAGTGTTTTCCCTAGCAGGTAAT
ATAATAACAGAAAAGCGAAATAGATTATGCCCAAAATCTGTAGATAGCCTCCT
TTTTTTGCATTCCTATTACAAAAACCTAAACAACTCGCAATAGATATTCCTTCT
TATAAGTATATTTTATATTATTAATTCTTATTATTTGCTTAATTTTTGTATAAGTGT
TAAGTATTAAGTATAAGTATTAATTAATAATATATAAGATTGTTATTTGTTAAGA
CATTAGATGCAAAATCCTAAAAATGTGAAAGTAATGAAGTTCCTTATATTTAAT
AGATACTTTTTAAGCCCACTATGTTTTTATTATTTAGATTGAGACATTAAAAAA
CGTAAAAATCAACAAATGCCGTCTTTAATTGCAATTACTTTATGTGTTTGAAAT
GGGAGGCACCCATTGAGTCCATCAAAGAGCAAAGACATGAGCACAAAAATT
TTCTTGGGTATTCCCTTTTACCCTTCATTTCTTATACCCGTCACGCTTCCACCC
ATACAAATTTTAGGCGTACAAAAAATGAGAACTGCAGCCCGCATACAAAAAA
TGACCTGCGGCCGATCGTTGACTGTGCGTCCACTCACCCATACGGCTCTTGC
GCAGCAGGCCTCGGGTGGTTTTTTTACTCGTAACAAAAACACAACGTCGGTA
AAACACTCGAGTATTTTGTGTTGCCGCAAGTAGGGTGTCAAAAAAAACGGG
GTGCCTAGAGTACCGAGTGTTTATCGGGTGGACGTAGAGTGCGAGTGGCGGG
CTGCAGTTCTCTG

>HMSBEAGLE_IGypsy#LTR/HMSBEAGLE_IGypsy

TGGCGCCCGAACAGGGACCAGCGAATAAACGCGAACGAAAGACAAAATTCT
AAGTCGCGGAGCAAAATCAAAATTTTGCTAAAAAATATTCGTTGGTTAAATTG
TGCCGAAGAAACTCCCGCGAGTTATTAACAAACAAAATTCACGGTCGGCTAT

AAAATAAAATTGTTTGAGAAAAAAACAGATTTTCCGGAAGAGGAAAATACTT
ATCGGCATAGCATTGCCCATTGGTGGATCACAGTTTCTGTCAGGCCAGCCCGC
AGAAAACCTTCTTTGGAGTGCTGACGTGGATTCCCCAGTAGCCCAGGCAAAA
AGGACAACATTCACCCAGCCAGGAGGAGTGACCGCAACAATTCTATGTTAAA
TAAATGCAAAACGAAGAACAGCAATTTCATCGAAAAGGAGAAGGAAAAGTA
GAAGTAGAACGCCGAGAATAACGGAACTACAGAAGAGCAACACGGATAACA
GTTAACAGTGCAACCCAAAACGTAACGGCAGAGGTAAAGACGACGAACGGC
AGAGAACGGCTGCGTGAACCAAAGTGCAGACAAGAAGGAAAAAAAAAGAA
CAACAACAGCACAGCCGTAGCAGCAGCAGCAGCCCGCCAAAAAGAACAGG
AATAAGAACACGTTGACGACGAAGAAGCTGAAGCTGAAACAACCGAAGAG
GAGGCAGAGAGACTACAAGAACCTGGAAAGTACACTGGAAAAAACATCAG
CAATTTGAAATTCAGGCATCCACCCATAATAAAACATAAGTATACCAAAAAAA
AAAAAAAAAAAAAAAATTTTAAGTATATTATTATTATTATATTATTATTATAATAT
TATTATTATTATTATATTTTTATTATTATATTATTAGTATTATATTATTATTATTATATT
ATTATTATTATATTATTATTATTACATTATTATTATTATATTACTATTATTATTTTATT
ATTATTATTGATATTATTATTAATACTATATTTTCAACCCAGTTCCTAGAGATCTT
CTGAAAGGAAAATTTTCCTATTTACTGTTCCTTTCTGGTACACTGTTCTCAAA
GCAAAATAACCGCGGTGAGCTAAAATTTATTGCATGCAAAAATAAAAAAAAA
AAAATATAAAAATAAAAAATAAAAAAACAAAAACAAAAGATAAATAAGCAA
ACACATACACACGCATTCCATATTTTCTGCCCACAACTTTTGTTAAGTTCAAAT
TGGTTTAGGCTTGTTTTGTGCAGGTGTTGTCCGAATTAAAAATCAGTATGGCA
AATCCTAATAATATAATAAGACCATCAGCTTTTAGTTCAAACGAAAGACCGGT
GCCCTCAGAAAGACACGACCTGCCTGAACAGCATCGCGTAGACATGAGTGT
CGAACAGTTGACAGCATTAATTGGCCAAACGGTGGCCCAAGTTTTACCTGGA
TTGATAAAACAAATGAACAACAATACTCTTGACTTTACTGACGTGAGCGACC
AGGTCGTCGAGCCCGAATACAGAAATAATTTAGCGGACTTTGACAGGGTGCC
TGATATCGTAAAATCGATCAGGGAATTCTCTGGAAATCCAGCAGAATTCGGGT
CTTGGAAAAAGAGCGTTGATAGAATCATGGAGACTTATACCCCATTTGTGGGT
ACTCCAAAATATTATGGTATACTTCATACCATAAGAAATAAAATTGTTGGAAGT
GCTGATGTGGCACTCGAGTCCTACAGCATTCCGCTGGACTGGAAGGCCATGT
CGAGATGTCTCACTCTGCATTACGCAGATAAACGGGACATAACTACCTTGGA
ATACCAAATGTCAACTTTGGTTCAAGGCCACCAGCAGTCGGTGGAAGACTTC
CACCAAGACGTCTACAAAAATCTGTCGCTTATTCTAAATAAAGTCGGCTGCAT
GCAAATGAGCCGAGAATCCGAGCACTTTGTTACAAAAATGTACAGAGAAAA
AGCTCTGGATACTTTTATCAGAGGCCTTCGAGGAGATTTACCTCGCCTTTTGG
CAATAAAAGAGCCAGCTGATCTCCCCTCAGCTTTACATTATTGCCTTAAACTT
GAAAATCAAACTTTCAGGTCAAACCATGCGGCGAATAAGGGTCAACAGTCG
AGTTTCAGAGGAAACGAAAAACCCATTCCGGCACCCCGCAATTTTCAGCCGT
CGAATGCCTTTGGCCATCGACAGCCTCCACCGGTCCCTCCACGACATCCCAT
GAGGAGACCTCCACAATATTTAGGCCAGCCATTCCCGGCGCCAAGGCAACAC
GTGTCTAACTTCGGTAATGCACCGTTCATTCCACCCCGACAAAACTATCAACA
GCAATGGCAACAATACAATGCCCCACCCCGTCCCTTTGCGACCAAACCACAA
CCAAGACCAGAACCAATGGACGTGGACGGCAGTGTACAGACGCGAAACATT

AATTATATGAATAGACCACACTCGGACATAAACAAACGACAAAAGAACTACA
ACATCCAAACAGTGAACATGAGACAGCCAAGTATTGAAGTAACTGGGTCCA
GTTCAAGCATGACAGGCTATCAACGGTCGATGCAAGATTATGAAACCCAGAA
CAATATAAACGGCACACTAAATGAATACTGTGATGGACAAATAGACAATTTGG
ACAGCGAACAACGAGACCACGTGTTGCATTTTTTAGAGTAGAAGACTCCTCA
CTACCATACTTCGAATGTAGAGTGGGGAGTGGAAAGGTTTTAAGGGTGTTGA
TTGACACAGGCTCTAATAAAAATTACATCCAGCCCAATTTGGTGACGAACGC
GATACCAAATAACAAGCCTTACATAGCTGATACTCCAGGCGGTGATGTAAAAA
TATCGCATTACAAAAGAGCAAGCCTTTTCGATTTCGAAATAAAATTTTTTTTGT
TACCAACGCTGAAGACTTTTGACGCCATACTTGGCAAAGACACAATGAAGGG
AATGGGAGCGCAAATTGATTTGAAGAACCTAACCATGACACTGGAAAACGG
GAAAAGAGTTGTTCTCAAAGAAAAACAGTTCGAGGCTGTTAGCACAATTAG
TCCGAGAATAAAACACTTAGAGGTGGAACAGAGAATGATATTGAATAAAATA
ATTGATTCGTATCCAGGTCTCTTCGCAGACCCGAATCAAAAACTAACCTACAC
AACAAGTGTAAGGGCAGCAATCCGAACTATATCGGATACGCCAATATACTCAA
AATTCTATCAGTACCCAATGTCTCTTAAAGACGAGGTACACAAACAAATTTCC
GAACTTTTACACGATGGAATCATTCGACCCTCAAGGTCACCTTACAATTCACC
AGTGTGGATTGTACCAAAAAAACTCGACTCCTCTGGCAAGAAAAAATACAG
GGTGGTAATTGACTATCGAAAACTTAACATGGTAACGGTAGCGGACAGATAC
CCTATCCCTGACATTAATGAAGTGTTGGCCCAATTGGGAGACAACAAGATTTT
CTCAGTGCTCGATCTGAAAAGTGGGTTTCATCAGATTCTTTTAAAGGAATCTG
ATATCGAAAAGACCGCCTTCTCCATCAATAATGGAAAATATGAGTTTACACGA
CTCCCATTCGGTCTGAAAAATGCACCGTCAATTTTCCAGCGCGCACTGGACG
ATATTCTTCACGAACATATCGGTAAGATATGTTTCATTTATATTGACGACATCAT
CATCTTTAGTAAAGATGATGAAACACATTACCAGAACCTTGACACTATTTTCA
GAACTCTTCAGCAAGCCAACATGAAATGTCAGTTGGATAAATGCGAGTTCAT
GAAGAGAAAAGTGGAGTTCCTAGGCTTCGTCGTGTCCGACAAGGGCATTGA
AACCAGCCCAACCAAGGTACAGGCAATCTCAGACTTTCCAATTCCAAGGACA
CTCAAAGAACTGAGATCATTCTTGGGATTATCCGGATATTACAGGCGATTTATT
CCTAACTACGCTAAGTTAGCAAAACCACTTAGCTCGCTTTTGAGAGGGGAGG
ATGGTCGAATTTCCAGGACATTATCGTCAAAAAAATCCGTCTCCCTTAATCGC
GAAGCAATAGAAGCCTTCAAGAAATTGAAGAGCAGCTTGGTTTCTCCAGAC
GTAATACTCCACTACCCGGACTTTAAGAAAGAATTTCACCTAACAACGGATG
CTTCCAATTTCGCAGTAGGTGCTGTTCTTTCACAAGAGAACAGACCCATCTC
ATTTTTATCGAGAACACTCTCGAAGGCGGAAGAAAATTATGCCACGAATGAG
AAGGAAATGTTAGCCATTATCTGGGCTCTAAAAAAGCTAAAAATTTACCTTTA
CGGTAAAGCAAAGGTGAAAATCTTTACTGACCATCAGCCTTTGACCCATTCT
CTCAGTAGTTGGAATGGAAATGCGAGGATTAAGAGATGGAAAGCATACCTTG
AGGAATATGACTACGAAATTTTCTACAAGCCAGGCAGAGAAAATACTGTAGC
CGACGCTCTGTCCAGAGGACCGACAGTCGAACAAATTAACACAGTAGCCTC
AACAATGCACAGCTCTGACAGTTCGAGCCATGGGCTGATACCTAGCGTTGAA
GCCCCGATAAACGCATTCAAGAATCAAATTTTCTTCAGGGAGTCCGAGTCAG
AGAACTACTCATTTAGCATTCCATTCCCGACATTTCAAAGGCATTTAGTAGATC

GCAACTTGTTCACACCCGATAGTCTCTTGTCAGATTTGAAAGAATATCTTAAC
CCATCCGTGATTAATGGAATTTTCACATCCGAGGATGTAATGGGGAAAATTCA
AATTCTCTACCCCATCCATTTTCAGGGTTTCAAGATTAGATTCTGCCAAAGCA
AAGTCAAAGACCTTGTTAACGAAGCCGAACAAGAGGAAGAAATACTTAGGA
CACATAACAGAGCACACAGAAATGCTGTGGAAAATAAAGCTCAGTTGTCCG
AAAGAGTATACTTTCCTAAAATGAGGAAAAAAGTTTCGGCTATCGTGAATCA
GTGTTTGGTGTGTAAGACTGCTAAATATGACAGACATCCCACGCATCCAGAA
ATAAGACAAACTCCCTTGCCAGAATACCCCGGACAAATTATTCATATTGACAT
CTACTCGACAGAACGACATCTGGTGCTCACGGCGATTGATAAATTTTCCAAA
CTGGCCATGGGAAGAGTTATCAAATCCAAAGCTGTAGAAGACATTAGGAAAG
CCCTAAGAGATATCGTGTTTTATTATGGAGTGCCTAAATTAATAGTAATGGACA
ATGAAAAGTCCCTCAACTCAGCCTCTATCAAATTCATGTTGACAGACCAGCT
GGGTATTGAGCTCTACAAAGCACCTCCGTATAAGAGTACAGTAAATGGACAG
ATAGAAAGATTTCACTCCACACTCTCTGAAATAATGAGATGTTTGAAAGGAG
ATGGAACACATAGGGGCTTCGAGGAACTTCTCGATAGGGCCATCTATGAATAT
AACTACACTGTCCATTCTGTCACAAAAAAACGACCTCTAGAGGTGTTCTTCG
GCAGGATAGCTACCGTAGCTCCAGAAAAATATGAACAAGCTAGACTGGACAA
TATAGACCGACTTAGGCAAAAACAGGAAACCGACATAGAATACCACAACCG
GACAAGAAAGCCCATAAAAACCTATATCAAAGGGCAAGAAATTTTCGTTAGG
GTTAATACAAGATTAGGTTCTAAGTTATCAAGTAGATTTAGGAAGGAACTAGT
TAAGGAAGACCGAAGTACCACAATATTAACAGAATCAGGGAAATTAGTACAT
AAAAGTAACATAAGATCATGATTGCTTCAGAACAATTACTGTGGCCACATCGA
TAGACAAGTAGAACATAGAAACATTAAACATACTGATTAACGTAACCACGAG
AATTAAATTTGAAACGATTCGACTTTGACAGACGAGTTGGCCATAGGTTTGC
AGAATTAGATTGGGCTCGTGAAAGAAGAGATTGCGTTGTGAATGCGTTTCTG
CGGAGCGAGTTTAAATTGAATGAGACCAGTAGCCTACTTGAAAACAATATAC
CGATTTCTGCCTTGACGGTCAAATAAATGTTAAAACTTTCACACGTTCTCGCT
TTACACAACGGAACTACAATTTGAATGCCATTGAAATCTCCATATTGAATCAG
TTAATTCCTTATTTCTTATATTTTAAAACATGATCAGTAAAGGAAACTAATAATA
ATAAATAAATAATAATTTAATTTGAATAATAATAAAATTTACCAATTAAAGACG
AAATGTACGGAATCAATGCCTTCTGCAAAGGTTCCAATGGTCTAAAAATATGT
AGATATAAATTCATTAGTAATTTAGTAAAAACCAGAATTTTAATAGAAGCAATC
AAGTTGTCAATGCAGAACACATCCGAGTTGCACAAACCTCGAACGCACGCA
ACAATAAACACCTGGCATTCAAACCATTAATGCTGTGCCCAAACTACCACGA
ATCACCCCACCAACGATGCGACTCAAAAACATCCCATCCTTATGATGACCGCT
CGAGGACAAGCGTCAATTAAGAGGGGAGG

>HETAJockey#LINE/HETAJockey

AAATAAATAAAATAAATTAAACAATTAACTAAATAATTAAATAACTAAAATTAA
TAATATAATCCGTTCGCTTGCCAAAGACTCTCACGCGCATAACTAATTAAAATC
GATTTTCAAGTTGACAAATAAATGGTTTAAAATTGTCCTCAGGCTGCAAAGA
AAAGCCGCGGCAACAATAAACATTTAGTGACACGCGAAAAGCGAACATTTG
ATTAGTGTAATACTTGTGCAAACCGACAAGCTGCCGCCATAACAAAACGGAG

ACGAAGAATCATAAAGAACAAAAGCTAAATCCACCAGCATAGCAAAAATAA
ATTAACAAATAAAATAAAAGCAAATTTAAATAACATAATAAATTAAACTTATTT
AATAAACCAATTAATTTTAATTAATTCAATTAAACGCTAAATCTACATAATACTC
CACGCGCAAATTAATTGAAATCGTCTTTCTAGTTAATAAATTAAAAGTTTAAA
AATTGTCTCCGGCCGCAAAATTTGAACCGCGACGATAAAAACATTTAATTGA
CAAACAAAAAGCGAACAATTATTCAGTGAACTATTTGTGCAAAATTGACAAG
CAGACGCCATAATTAAAAGGAGAAGAAGCCAAAAGACGAAGAGAAGAAAG
CAACCAGAAGAACTCAAAGAAGAAAAGGAGGAAAGCCCAATTAAAGAAAG
CCAGGGTATTTATACCTTACACTTATCGTTTAATATAACAAAAACCCAACATGT
CCATGTCCGACAACCTTTTTTCTGACGATGAGGTACTTTCAATTTCCTCAAGC
CCAGAACAGCGATCTTCTCCGTTCTACCTCAATATATCGCCCATGTCCCACGG
ATCAGACAATTCTCAGATTAATACAGTCATCATTAATTCGAAGAAATTGCCCTC
AAATCAAGCAGACATAAGTTTAAAAAACTCTTCTGGGGCTGCTATAAAAATT
GTTAATTCCCTTTCACACAAGAAGAAAGAGAACACAAACGTTAATAATGCCC
AAAAAGACCCCCTCTCACTCACCAATACTACTGCAAGCACTTGTGGCGCCAA
AAGCAGCATCTCAGAGGGGAAATTGTCTTCTCCTCCGTCCACCTCACACACA
TATGAGGGGAAATTACTCACAAAACTTACTCACACACACACAGACTTTAGAG
GCGCCAAAACGAGCGATGCAATGGGAAGTTTCCCCTCTCTCTCGCACAGCGA
CAATAGCATAGAGAAAAATCTGAGTTCTTCCACCAAAATTGGACCAAACGCT
TCTTCCCCTCCTTCTCATGCACACACTCACACTAGCAAATCCACTGATATAAG
CTTAGAAAGCCGCTCAAAACATCCCGCGCTTGCCAATACGGACGCACGCTCT
ATAAAAGCCAATGCTAATGACAATGGGGAAATTTTCTCCTCACTTATACAAAT
TGACGAACGCAAGCAAGAGGAAAGGCCTTGCACAACTATCAACGCTTTTTG
GTCTATTTTTAAACCCAAGCCGGACGTTACTAAACTAAGTCTAAAGAGGAAA
CCCACCAATCCCACTAAAAACACTGGGAAAAAATGCATCTCCCCTCATAAAA
AGAGCGCTTATTTATGCCCTTCCGCTCAGGATGATTTAAATTTAAATTTAAACC
CCAAATCTAGCGCCAAGCCCACTGTGGTGAATTTACCAGCTGCCCGCATCCTA
AGCCGGCCTGCAGCCAAGCGGGATTTATTTAAATCATCATCCTCCCGAAGCCC
AGACGAGCAGCCTATGAGTTTTTCGGAAGTGGTCGCTGGCACGGGTTCAATT
TTTGCGGCACCCTGTGTCCCGGCACCTTTAACGAAAACTCCAGGCAAGCGGA
CAAACGACGATCTGGACTGCTCCAACTTTAAGACGCCCAATAAAAAATTATG
CGCGACTTCCAACTTTGTAACTCCCAGCATTTTTCCGCCGCTCATCACTCCCG
TTTTCAAGAGCAAGGCAGCTCAATCTGTTTACGAGGAATCCAAAGCCAGAAA
TGGACCCCCCCCGCCGGCCCTCGCCTGCAGCATCAATGCCTCTGCTCGCAGC
GCACGGCGCCACCCGGGATCGCCCCCCTACCCCCTCATAATACAGATGCAGA
GCTGCCTCCATGGAAAATCGTGCCCCAGAGCCGTAGAGCACCTCCTATACTC
GTCAATGATGTAAAGGAAATTGTACCTCTACTGGAAAAGCTGAACTACACAG
CAGGAGTCTCCAGCTATACTACTAGGGCTATAGAAGGAAACGGGGTCAGGAT
ACAGGCAAAGGACATGACCGCCTATAACAAAATTAAAGAAGTCCTGGTGGCC
AACGGACTTCCTTTATTCACCAACCAGCCCAAGTCCGAGAGAGGCTTCCGAG
TCATCATCAGACATCTCCACCACTCCACACCATGCTCGTGGATAGTCGAGGAA
CTGCTGAAGCTCGGATTCCAAGCGCGATTCGTCAGAAATATGACGAATCCGG
CTACAGGTGGCCCCATGCGAATGTTTGAAGTGGAGATCGTCATGGCCAAAGA

CGGCAGTCATGACAAAATACTCTCACTCAAACAAATCGGTGGGCAAAGGGT
GGACATTGAAAGGAAAAACAGGACACGGGAGCCAGTCCAGTGCTACAGATG
CCAAGGCTTCAGGCATGCCAAAAACTCTTGCATGAGGCCGCCAAGATGCATG
AAATGCGCTGGCGAACACCTGTCTTCCTGTTGCACCAAACCAAGAACCACCC
CCGCCACCTGCGTAAATTGCTCTGGGCAGCATATTAGCGCGTACAAAGGATG
CCCTGCATATAAGGCGGAAAAACAAAAGCTGGCGGCAAACAACGTTGACAT
AAACAAAATAAGAACAATCAAAGACGCAACAAATAACTTTTATAAACGTCAA
GGCCCCCCTCTACGCAACAACACCCCTCGGCTACCGCACAGCTCAGCAATCC
TGAGCAAATCAATTGCCGAAGCTCGCCAGGAGGCAGCCAGAAAGTCGATGT
TAAATCCATTCCGACAAAATATAAACGACAGAAGACCACGATTCTCCTCCCAC
GACACGGCCATTCAGAAGCGTCTGAATAAATGGCGCCGAAACACCAACAAA
ATACCCAAAAAGGGTAGGATAGCCTTAAAGGATAATGCAAAGCCACGACCGG
CACATAGGACAAGTAACCCAGCGCAAAGACATCTGGAGGACTACCAGGACA
TGCTCCGAAGGGAAAGGAGTGAAGAAAACGACCAGGAATCTGAGAAGGGC
ACCCCCAATACCAAGCAGGTCGGCAATGACAGCCCTCCGACCACGAGCAGA
GCAGCCAGAGCCAGCTTTAAGCCAAGAATCATTGACGATACCACGCCATCGC
CAAAAATCTGCAATCCCAACTCACAAAAAGGCCTCTTGGACGACCCCACAA
CAAGCTTAGCTAATAGAGTCGACAATTTAGAAAAGAAAATTGACATTTTAATG
GCCTTAATCATACAAGGAAGAAATAACAATCTTGACATGGATACATCCAATTA
ATCTTACAACTACTTATATATTCTTTAATAAATATATCCAATAGAAAAGCGCACG
TCGGTCTGCTTTTAAAATCCTTCACCGTCATCACCTTCCTCGACGGAGCCTAA
TTTATTGGAAAAATAAATCAATTATATGTTGGCACAAAAATGTAAACACACAC
TCACCTAAACGCACCCGGACGAACAAGCCTATGACAACGCACTCCAGCTGAT
CTGTAAGAAACAAAAAATATGAATAGATAGATCGATATGAAAAGGATATGTGC
GGCAGAAACATGATGAGCAAAAGGCGACTCGCTGCAGCAACTTATGCACAA
CGTCACTTACCTGAAATTTCTTGCCGTACGATCTCCTGTAGTATCCCTTATCAC
AGCTGCAATCTACTTGCAATGCTGCACTGCAATAAACGTACTACAAAAGCTG
CATACGTTTTGATCAGGACACCTCGTGCGGACGTGCTAAAAAAAATTTCCTTT
CTGCTGCTCTTATTGACGCTAAAACCTTAAAACCTACAAACAAAACAATTAA
ATAATAACAAATCAAATAAGACAACCAAATAATACACTTACCTCATTGACTGC
AGCTAAATCGCTGACCCACATTCAGTGCAGCCGACAGCAGGAGACGGGCCC
GCAAAAGCAAAACAAAATCGCCAATTTTGCGATTATAAACACGAAAAATTGA
CAATTTTGCGATGCCGTCTCCGCCTCCTGATGCCACTGCATTGACAAGCATCA
CTAGCGAGGAGCTGACACCACACCAAAAAGCTGTAAAATCCGTCCACAAAT
TGTATATTTTGCCTCAGTGTCGTATCTGCAATGTTTTTCCGATAACCTGTAAGG
AAAGAAAAATTAATAAGAAAATTATACAAAATTAATTAAGGACGACAGAAAA
TAGCAAACCAGACAGGCAAATTAACAGATACAAATATGAGACTCCATCCTGC
TGCCGACACACAAGTAAATCCTTCAACTCGACAACAGGAGACGGGCCTTGC
AAAAGCAAAACAAAATCGCCAACTTTTGCGATTATAAATACAAAAAATTGAC
AATTTTGCAACGCCGTCTCCACCTCCTGTTGCCACTGCATTAATAAGGATCAC
CAGCGCGGCGTGACGCCACACTAAAAGGCTGCAAAATCCGTCCACAAAATG
TATACTTTTCCTCAGTACAATACTTTCTAATGAACTTCCGCCAACCTGCAATGA
AAAGAAAAGAAATAGGTATATAAAACAAAACAAACAAAAGGACAACCTAAA

ATTAGCAAACCAGACAGGCATACTAGTAGATGCTAATATGCAGCTCCATCCTA
CTGACGACAACCACGCAACTCCTTTCTCCAAGACCGCAAATACTGAAACAA
GGAAGCACAAGCTAATACTGGGAATTATTTATTTAAACAAAAATACTTATCTA
ATTGCCAATTCGACGACTCCAAATCCGCGGCTAACCGGCGGCGATGGCCCAT
AAATAAAGGGCCTCCTAATTAATTACAAAATGTACCTGAAAAACATAAAATTA
ACGCAACTATAATTAACGCAATTAATAAATCAAATAAATACAAGTATAATACTT
ACCTCCAAGCAAACGTACCTGAAAAACAAAACCAAAAAAAAAATTAATGCA
ATAAATAAATCAAATAAATACAAACATAATACTTACCTCCAATTTACCTCCCAG
CCAATCTACCTGAAAAACATAATCTAATACAATCTCAAAAACAAATAACAAAT
GTAATACTTACCAAATTTTAATTTTGTATTCATTTCCATGACCCCAACGCTGCA
ACTGTCCTCGGCAACAATTCCTGTTCCGGCGGCTCCATGCTGCCAATCCTGAC
GCACTGGCCACAAGACGCGGCGCTGCTGGCAATCTCTCGATGAACAACCGAT
CTACAATTTCCATGACGACTCCTCTGTCACGATGAGACAGAAGACACCACCA
ACGCCAGCAGCTCCAAAACAATACAACAACGGCCGCGCGGAACCCATCTTC
AGAATTCCCTCTTCCTGACGACCGGCGAACGAGTTCTGGAATAAACAATGTA
TTAATTGCAAACATCTACCGATGAGGGTAGAAGAGATACTCACCAAACGACT
GCGGCGCGGGAACAAACTAACTGCAACGCCGGCCGGACCTATTTGTTGCAA
GTGGCGCGCATCCAGCGCCTGCAACATGCCCCAGCCCAAGTACACAACTACT
TACCTGCAACGTCGCCAGAGGCTCCCAGCGAATCGGTGCTTCCGTCCTTCTG
GCGGGGGTACCTGAAAAGAAACAAATTAAACAATATTAATCCTAAATTTCAAT
GTTTTTTGTAAAATAATTTAAATTGTTAAATGTAAACAAGCCTTGCAATATGTT
AATGTTACCAGTCCATGCTACTGTCTAAAAGCCAAGAATACAAAAAATACTAA
TTATAAACTAACTCACCACGCCCAACCCCCAAACTCACCCCATGCAATGTTAA
ACCTATAAATTCAAATAATTGTACCTATATATTGCACATACTGTAATCAAAGGC
AAAATAAATCGTGGATGCGGAACAGAATTTACTCTGTCTCCGTACCTCCACCA
GCAAAGTTAAAAAA

>HELITRON1_Helitron#DNA/HELITRON1_Helitron

CTGCGCGCGAAAGAATACATTAATTTGCGAGACGCTATCAACAACAACGCCG
ACGTCGCCGAAATCGGTAACCATATCATTTTACCGTAATCGTAAATAGGCAGT
CCACGTCATATGCAAGAATATTTACAGGATGCTCTGACTTTCGTGCGCGAATA
TGGACGACCATGTTTATTTATCACGTTCACATGTAATCCAAAATGGCCAGAGA
TTACATCTTTACTACTGCCTGGCCAAATGCAATGCATCGCCATGACATTACAG
CACGTGTGTTCAGACAAAAGTTGAAGTCTTTAATAAGTTTCATTACTAAATCA
CATGTATTTTGTCCTACACTTTGCTGGATGTGTTCGGTTGAGTGGCAAAAGCG
GTTTGGTTTGGTTCAACGACAGAATCTGTCCTGAAGTAATCGATAGTATTATTT
CTGCGGAAATATTGTAGATCCATCCACTTCATTGCTTTGCATGGCTAATAGAAA
TGTACTAAAAATGTCCCAAAAAATTTTACCAATAATACGGTCAGAAATGTCGA
CGAATACCTAATATATCGTCGAAGAAATCCTGA

>HELENA_RTJockey#LINE/HELENA_RTJockey

AAAGCTCCAGGAATTGACAGGATTTGTCATGCCACGCTAAAGGTTTTACCTAT
AAAAGCGATAATATATATAGCACTAATCTTTAATGCTATTTTAAGGATCCAAGT

GTTCCCAAGACAGTGGAAAATGGCTGCTATTTTGATGATCCACAAGCCTGGA
AAGCCWGAAGATGATCCTGAGTCGTATCGGCCTATAAGCCTCTTACCCTCCCT
TTCTAAATTATGGGAGAGACTTATTGCCAATCGGATAAACGACATTATAAGAC
AAGGCAATATCTTGCCGGATCATCAATTTGGATTTCGAAAGGGACACGGAAC
TATTGAACAGGTCCACAGACTGGTGAAACACATATTACAGGCTTTTGACGAC
TGCGAGTACTCCAACGCTGTCTTTATAGATATGCAACAAGCCTTCGACAAAGT
ATGGCATGTTGGATTATTATGCAAGATAAAGACCCTTCTACCTGCGCCCTACTT
CTGTATTTTAAAGTCATATCTGGAAGAACGACAATTTAAAATCACGGTGAGAA
ATAGCTACTCCTCTATATACCCAATGAGAGCTGGAGTCCCACAGGGCAGTGTT
CTCGGACCGCTACTATATTCCTTGTACACTGCTGATATCCCTTGCCCGAATTTC
GAACACATGGCAGCACCGAACAGGRCTCTTATTGCAACCTATGCAGATGACA
TCGCAGTTGTATATAACTCTAGGGACAGCAGAGAGGCAGCTAACGGACTACA
AGAATATATTAATGCTCTGGCAGCCTGGTGTAAACGGTGGAACCTAAAAATAA
ACCCACTGAAAACAACAAATCCATGCTTCACATTAAAAACGCTTATCCCGAA
CACCCCTCCAATTCGGCTAGAAGGAGTTACCCTGAATCAGCCCCTGCAAGCA
ACATATCTAGGTATCACCCTGGATAAACGGCTCACCTTTGGGCCGCATCTCAA
AAACACAGTAAAGAAATGTGGTCACAGATCACAACAGCTGAGATGGCTCAT
GAATAGAAGGAGCACTCTTTCGATGAGGTGCAAAAGAGCTGTGTATGCGCAC
TGTATCGTACCGATATGGTTATACGGGATCCAGATTTGGGGAATTGCAGCCAA
ATCGAATTATAATCGTATCCAGGTGATGCAAAATCGCGCATTACGACAAATAA
CCAACTGTCCCTGGTATGTACGTAACTCTACACTCCATAAAGACCTCAATATT
CACACAGTTGAGACACAAATTGGGAGACATACAAGTCGATATAGTGACAGAT
TACTGAGCCATAGCAGTCTTCTTGCAAGACGTCTCATCCCCGCTCGACCTCTG

>GYPSY_LTRGypsy#LTR/GYPSY_LTRGypsy

AGTTAACAACTAACAATGTATTGCTTCGTAGCAACTAAGTAGCTTTGTATGAA
CAATGCTGACGCGCCCAGAATTGGGTTCAACGCTCCACGCGAAGAATGCTGG
CAGCGGAAAGCTGACACTTCCTACCGGGAGTGTTGCTTCACGCTGCAAGAA
ATGCTGAGTCGGCTTGCCGACTTGTGGCGGCGCGATGCATTGCTCGAGGGTA
AACTTAGTTTTCAATATTGTCTTCTACTCAGTTCAAATCTTGTGTTGAAATAAA
CCACAGCTTGCTCCGGCTCATTGCCGTTAAACATCATTGTTCTTATTTACAATC
AAATCGCTATCGCCACAAGGCTAGTGATAATAACTAAGGGGGCGAAGTCAAG
CCCTCCAACCTAATCTCCATAAACAGTGTCTAAGACGAACCTCAGCGAAAGA
AGGAAGATCTCTAGACCTACTGGAAATAACATAACTCTGGACCTATTGGAACT
TATATAACT

>GYPSY_IGypsy#LTR/GYPSY_IGypsy

TTGGCGCCCAACCAACAATCTGAACCCACCAATCTAATTTAACACACTTTGTC
AGGCGACAAACAGGGTAGTTAAGTTAGAAAAGCATGTAAGTTTTACAAGAC
ACTTCTTTGACGCAATCAAGAAATTTACGAGTGAAAAAAAAAAAAAAAAAA
AGTTGTGTATCTGGCCACGTAATAAGTGTGCGTTGAATTTATTCGCAAAAACA
TTGCATATTTTCGGCAAAGTAAAATTTTGTTGCATACCTTATCAAAAAATAAGT
GCTGCATACTTTTTAGAGAAACCAAATAATTTTTTATTGCATACCCGTTTTTAA

TAAAATACATTGCATACCCTCTTTTAATAAAAAATATTGCATACTTTGACGAAA
CAAATTTTCGTTGCATACCCAATAAAAGATTATTATATTGCATACCCGTTTTTAA
TAAAATACATTGCATACCCTCTTTTAATAAAAAATATTGCATACGTTGACGAAA
CAAATTTTCGTTGCATACCCAATAAAAGATTATTATATTGCATACCTTTTCTTGC
CATACCATTTAGCCGATCAATTGTGCTCGGCAACAGTATATTTGTGGTGTGCC
AACCAACAACCAATGAGTTGGGCACATAACTACAGAAAGGTTAAGGTCGAAT
ACGAAAGCGAGGATAGCTGGGAGGAGGAGCAAGTAGGCCAAGCATTAGGTC
GGCCGTTAGATAGTGCCACGGTAGATATTACCATGGACCCCAATCAGATTCAA
GCTCTTATCGACAATGCTGTCAGACAGGCATTGTCGCAACAGCAATCCCAATT
TCAGACACAACTCAATTCCCTAGCTGCGCGGGTACAGAGTTTGCAGGTGGAA
GCACCGCAAATCAAGATTTACGAAAAAGTCTCTGTTAACCCCGATGTTAGGT
GCGACATTCCCCTTGACATAATAAAGTCTGTACCAGAGTTCTCCGGTACCCAA
GACGAGTATGTGGCCTGGAGACAATCGGCCATATACGCCTACGAGCTCTTCA
AACCATACAATGGCAGCAGTGCCCATTATCAGGCTGTTGCCATATTAAGGAAT
AAAATCCGTGGCGCAGCCGGGGCTTTACTGGTCTCCCACAATACGGTATTGA
ACTTCGATGCTATTTTGGCCAGACTAGACTGCACGTACTCGGACAAAACATCC
TTACGCCTGTTGAGGCAAGGATTGGAAATGGTTAGGCAAGGAGACCTACCAC
TAATGCAATACTACGATGAAGTTGAAAAGAAGCTAACGCTTGTCACTAACAA
AATCGTAATGACGCATGAACAAGAGGGTGCTGACCTGCTTAACGCTGAGGTC
AGAGCCGACGCCCTGCATGCTTTTATTTCGGGGCTCAAAAAGGCCCTCAGAG
CTGTGGTCTTCCCGGCCCAACCAAAAGACCTGCCATCTGCACTGGCTTTAGC
TAGAGAAGCAGAGGCAAGCATAGAGAGAAGCATGTTCGCTAACTCCTACGC
CAAGGCCGTAGAGGAGCGAGCGCATTCGGGGGCAAACGGCAAGAGCCGTTT
CCAGGGGAAGCCAAATAAAGAAGAACAGGGACAGGACAGGAATCCCCACTT
CACCAAACGCCCCAAAAATAACGGACAAACCAACAAGGACACTCAGGCGCA
AGCACCCCAGCCAATGGAGGTCGATTCATCCTCCAGGTTTAGGCAGCGTACT
GAACATTATCAGAATCATCCTAACGAGTCGAACGCGTTTAAGAGGAGAAATT
CCTCAGAACGCTCAACAGGACCGAGACGACAACGTCTGAATAACGTTGTCC
AAGAGGCCCCTAAACAAAAGGACCCCAAAGAAGAGTATGAAAAAACAGCA
AAGGCTGCAGTCGAGGAAATCGACAGCGAAAATGAGTACGCTCCCAGTGAC
GACTCGTTGAATTTTTTAGGGGGCGCTCCCGGTTGCCGTTCATTGAACGACG
GCTGGCTGGGAGAACCTTAAAGATGCTAATCGATACCGACGCGGCAAAAAAC
TACATTAGGCCCGTAAAGGAGCTGAAAAATGTAATGCCGGTCGCCAGCCCTT
TCTCGGTGAGCTCAATACACGGCTCCACCGAAATCAAACACAAATGCTTGAT
GAAAGTCTTCAAGCACATCTCCCCATTTTTTCTTTTGGATTCTCTCAATGCGTT
CGACGCTATCATAGGCTTGGACCTGTTAACACAGGCCGGGGTAAAACTCAAC
CTTGCAGAGGACTCCTTAGAATACCAGGGCATCGCTGAAAAGCTTCATTATTT
CAGCTGCCCCAGTGTAAATTTCACTGATGTAAACGATATTGTTGTACCTGACT
CCGTTAAAAAGGAGTTCAAGGACACAATAATAAGGAGGAAGAAAGCTTTCT
CCACAACAAATGAAGCTCTTCCTTTTAACACCGCTGTCACTGCCACAATTCG
GACAGTTGACAATGAACCGGTGTACTCAAGAGCGTACCCAACTCTTATGGGT
GTCTCCGACTTTGTGAACAACGAGGTCAAACAACTGCTGAAAGACGGCATT
ATCAGGCCCTCAAGGTCTCCCTATAACAGCCCGACCTGGGTTGTTGACAAAA

AGGGGACCGACGCCTTCGGGAACCCAAACAAGAGGTTGGTCATTGACTTCA
GGAAGCTAAATGAGAAAACTATTCCTGACCGGTACCCGATGCCTAGCATTCC
CATGATTCTAGCGAATCTGGGCAAGGCAAAGTTCTTCACTACCCTTGATCTTA
AGTCAGGGTATCATCAAATTTACCTCGCGGAACACGACCGCGAGAAGACATC
GTTCTCGGTGAATGGTGGTAAATACGAGTTTTGCCGTCTACCGTTCGGCTTGA
GAAATGCAAGCAGCATTTTTCAAAGAGCCCTAGACGATGTGCTTAGAGAGCA
AATCGGGAAGATATGTTACGTCTATGTAGATGACGTCATAATTTTCTCTGAAAA
CGAGTCCGACCATGTCCGCCACATCGATACAGTACTAAAATGCCTGATCGATG
CCAACATGAGAGTAAGCCAGGAGAAAACTAGATTCTTTAAAGAGAGTGTAG
AATACCTCGGCTTTATTGTCAGTAAGGACGGAACTAAATCCGATCCAGAGAA
GGTGAAGGCCATTCAGGAGTACCCTGAACCAGACTGCGTTTACAAGGTTAGG
TCCTTCCTTGGTTTAGCCAGCTACTACAGAGTCTTCATCAAAGACTTTGCTGC
CATAGCCCGCCCGATCACCGATATCCTAAAAGGGGAAAATGGTTCGGTGAGC
AAACACATGTCTAAAAAAATTCCTGTTGAGTTTAATGAAACTCAACGCAACG
CGTTCCAAAGACTGCGAAACATACTAGCATCCGAGGATGTCATACTCAAATAC
CCCGACTTTAAAAAGCCTTTTGACCTTACTACAGATGCTTCGGCAAGTGGTAT
CGGTGCAGTCCTATCCCAGGAGGGCAGGCCAATCACCATGATATCGCGTACC
CTTAAACAGCCCGAGCAGAACTACGCCACAAACGAAAGGGAATTGCTGGCG
ATTGTATGGGCCCTAGGTAAGTTGCAGAACTTCCTGTATGGCTCTAGGGAGAT
TAATATATTTACCGACCATCAACCCCTCACTTTCGCTGTTGCCGACAGGAACA
CGAATGCCAAGATAAAGAGGTGGAAATCTTACATAGACCAGCATAATGCCAA
GGTTTTCTACAAACCTGGCAAAGAAAATTTCGTGGCAGACGCCCTCTCTAGG
CAGAATCTGAATGCCTTACAAAACGAACCCCAATCAGACGCTGCGACCATTC
ACAGTGAGCTCTCCCTGACCTACACGGTCGAGACAACAGACAAACCGTTAA
ATTGCTTCAGGAACCAGATCATTCTGGAGGCAGCACGTTTTCCGCTCAAACG
AAACCTGGTGCTCTTTCGAAGCAAATCTCGCCACTTAATCAGCTTTACTGATA
AAAGTTGGCTATTAAAAACACTTAAGGAGGTGGTAAACCCTGACGTCGTGAA
CGCTATTCACTGCGACCTGCCCACTCTGGCAAGCTTCCAACACGACCTCATT
GCCCACTTTCCAGCCACCCAATTTCGTCACTGTAAGAATGTCGTGTTAGACAT
AACCGACAAAAACGAACAGATCGAAATCGTCACTGCCGAGCACAACCGCGC
TCACAGAGCCGCACAAGAAAACATTAAACAAGTCCTTCGGGATTATTACTTT
CCCAAAATGGGCAGTTTAGCTAAAGAAGTAGTAGCTAATTGTAGGGTCTGCA
CCCAAGCAAAGTATGACAGGCACCCGAAAAAGCAAGAGCTCGGGGAAACG
CCCATACCCAGCTATACAGGTGAGATGGTGCATATTGACATATTCTCAACCGA
CAGGAAGCTATTCCTGACGTGTATTGACAAATTTTCTAAATATGCAATAGTGC
AACCAGTGGTGTCTAGAACAATAGTGGACATCACAGCACCCCTGTTGCAGAT
CATTAACCTGTTCCCCAATATCAAAACGGTCTATTGTGACAATGAGCCCGCAT
TTAACTCAGAAACTGTCACCTCAATGCTCAAGAACAGCTTCGGCATTGACAT
AGTAAATGCGCCCCCACTCCACAGCTCATCCAATGGCCAAGTTGAACGGTTC
CACAGCACATTGGCAGAAATCGCCAGGTGCCTGAAGTTGGACAAAAAAACG
AATGACACAGTAGAACTAATCTTGAGGGCGACGATAGAATATAACAAAACCG
TGCACTCAGTTACTCGTGAGAGACCAATTGAGGTGGTTCACCCAGGGGCCCA
CGAGCGCTGCCTAGAAATCAAGGCAAGATTAGTAAAGGCTCAGCAAGACAG

CATCGGAAGAAACAACCCTTCCCGACAAAACCGCGTGTTTGAGGTGGGAGA
ACGCGTGTTTGTAAAAAACAACAAGAGGTTAGGAAATAAGCTAACTCCACTA
TGCACCGAGCAAAAAGTGCAGGCAGACTTGGGAACGTCTGTTCTTATTAAGG
GGAGGGTGGTCCACAAGGACAACCTCAAGTAGACATTCCCTCTACAGTTAGG
TAGTAAGTTATGTCAAGGAAAATCCGAGCACTGTAGTATCACCTTGTCTTTAA
TTTCCAGGTTCACCCTCATGATGTTCATACCCTTGGTAGTAGCGAATGCTCGG
ATCACCGACTTTTCGCATGCCAACTACATTCCTGTGTTAGATGGGGATGTGCT
GGTGTTTGAACAGCGTGACCTCTTGAAACATTCGAGTAACCTTTCCGAGTAC
GCTAGTATGATAGATGAAACACAGAAACTGTCCGAGTCCTTTCCCCACTCAC
ATATGCGTAAGTTGCTAGAGGTCGATACTGACCATCTTAGAACCTTGTTGTCC
GTTCTCAAAGTCCACCATAGGATAGCTAGGAGTCTAGATTTCTTAGGTACAGC
CTTAAAGGTTGTGGCGGGTACTCCCGATGCCACGGACCTCTTTAAAATTAAG
ATCACAGAGGCCCAACTAGTAGAATCTAATTCCAGGCAGATAGCTATAAACTC
CGAAACCCAGAAACAGATAAATAAGTTAACTGACACCATCAATAAGGTGATC
AATGCCCGTAAAGGCGACTTGGTTGACACTCCACACTTATATGAAGCACTACT
AGCAAGAAATAGGATGCTGTCTACAGAAATTCAAAATTTAATTCTCACTATTA
CTTTGGTCAAATCAAACATTATAAATCCCACAATTCTTGATCATGCCGACTTG
AAGCCTCTTGTAGAACAGGATACCCCAATTGTCAGCTTAATAGAAGCATCTAA
GATCAGGGTCCTCCAGTCCGAGAATAGCATTCATATTTTAATTGCCTATCCTAG
AGTCAAGTTCAGTTGCAAGAAAGTCGCCGTCTACCCTGTATCTCACCAACAC
ACCATCTTGCGCCTCGACGAAGACACTTTGGCCGAATGCGAACATGACACCT
TTGCGGTCACCGGATGCACAGACACCACACACTTCACGTTCTGCGAGCGGTC
TCGGCGCGAAACTTGCGTGCGCTCACTCCATGCTGGAAACGCTGCTCAATGC
CACACTCAACCCAGCCACTTGCGAGAAATAAACCCCGTAGATGATGGCGTTG
TGATTATCAACGAAGCCGCAGCTCACGTTAGCACTGATGGCAGCCCCGAAAC
ACTGATAGAGGGAACCTACCTGGTAACCTTCGAGCGAACGGCAACCATCAAC
GGCTCTGAATTCGTAAATCTAAGGAAAACACTAAGCAAGCAGCCAGGCATCG
TGCGTTCACCACTACTTAACATCGTCGGCCACGACCCTGTGCTCAGTATACCT
CTGCTACACCGGATGAGTAACGAAAACCTACATTCCATCCAAAACCTTATGG
ATGACGTGGAATCTGAAGGCTCGCCCAGACTCTGGTTCGTGGCTGGTGTGGT
CCTAAACTTCGGCTTGATTGGCTCTCTCGCCCTTTATCTGGCATTAAGGAGAA
GACGAGCCTCTAGGGAGATACAGCGCACCATCGATACTTTCAACATGACCGA
GGACGGTCATAAACTTGAGGGGGGAGT

>GYPSY9_LTRGypsy#LTR/GYPSY9_LTRGypsy

AGTTATCATAAGTAAGATCAATAATACTGGACTTAATACTAAGTCAGTATCCGG
TCCGAATCTACTACGATAAATCAGCAGAATTTGCGGTTACTGTGAATGGACAC
CCACAGTGGGAACCACAGTGGTGCAGCGTTAGCAGTCTGGACGTTTTGGCG
TGAGGAGTCACGAGGCGCAACGTCGACAGAAATAACGGCAGGTGTGCCGCT
ATGAATATCTGAATGTGAAATTGTACTTTAGTTTTAAACAGAACTCAAAAGTG
ATCAATCGTATTCTGATGCACTTCATAAAGATAAAAATAATTATTATTAATAATA
ACTTAATAATTGTGTAATT

>GYPSY9_IGypsy#LTR/GYPSY9_IGypsy

GGCGCCCGAACGTAGTAAAAGTGATCTGAAACCGGATCAAAAGAACTCGCA
ACGGAAAATATTTAATTAAAGAACAACAAACAAAATAACAGTGAAATCGTTT
TATATTAACATATAAAACAAAACAACAACAACGAAAAACTAAAAATTAAAAG
CCCTAGTGCAACTACACTAAGCGAGTGAAAAACAAAAACCACGGCCGGTTC
ATATGTAACATATAAAACGAAAACGTACAACAACAACAAAAAATAAAACCCC
AGTGCAAATGCACTGAGTAAACGAAATATATAAAACGAAAAATACATAACCG
ACAAAAGAAGAACACAGGAGCGGACCAGCACACAACGGCACTAGAATAATA
ACGAAAAATGTATGTGAGAAGTTCATTGCCTTTTCTTTCTAAAACAAATTAAA
TAAAAATTATATTTAAAAGTGATTATCTGTCGATTTATTGGCCTAGTGAGAGTA
GTAGTAAACTAATATCAAAATTTTGTTCAGTACAATAAAAGCCATTGTTTCTTG
CAAACAAACACGAATGTATAGTAACTTGAATCGAAGTAATTCTAGTAGTGAC
GAGGACACAATACCTAGTTAGATGGAAAAAATTAAAAACGAACAAGTTACGA
TAATGAATATAGAACAGTGCACAACAGGAAACCTCGCAGCAACACAAGTTGC
GTAGGTTATCTGAATATGGTAGAAAATATGAATATGGTAGAAAAGCGGTCAAA
GCCGCGCTTGAGCACCAATCGACGGTTTTCGAACAGAAATTATCTGACACGA
CACATTATATGCTACGACAAATAGATACTATTTCTATAAAATCAGAAACTCCAA
AGGTAGTAGTATACGAGGCAGCTAAAGTAATACCAGGGATTACGTGTGACGA
ACCTTTAGATATCGTAAAGTCCATACCAGAATTCGATGGCAAACAGGAAAATT
ATGTTTCTTGGCGGCAAGCAGCAACGGTTGCTTACGAACTCTTTAGACCATAT
AATGGGAGTTCGAAACMCTATCAGGCTGTTGCAATTATACGCAACAAAGTTA
GAGGAACTGCCAATTCAGCACTATCCTCATTTAGTACGGTGTTGAATTTCGAC
GCGATTATATGCACGCCTAGACTTCACATACGCAGATAAAACCCCACTGCGTG
TTATACAACAGGAACTCGCAACACTGATGCAGGGCGATATGCCCCTGCTAAA
GTATTATGACGAGATAGAAAAGAAGCTAGCACTTCTAACCAACAAGACAATA
ATGTCACACGAGGAGGCAGGAGCAATGATCCTTAATGAACAACATAGGAATG
ATGCTCTACATGCCTTCATTGATGGCCTGAAAAAATCCATCAAACAGACAGTT
TTTTCGGCACGTCCAAAGGATCTGCCAACAGCCTTAGCATTGGCACAAGAGG
CAGAAACGAGCAATGAGAACTCTGTATTCAAAGCCGTCTATGCTAAGCATAA
CGAAGAAAAATACCAGAAACCCTCGTCTCAAAAGACCCAAGGGTGGCGTAA
CAACGCACAGGGTCAACAAAATTCAGACGGCTCCAACTATCAGGGTAAAAA
CCCACATTTTAATAAGGGCCAGGCCCTCGAGAAGTCCAACAACGGACCAAAT
AATCAATCACAGGGTCAGAAGGCTGGTAGTTCCAAACAACGCCACGCCCTCC
CAATTAATAAAGGCTTTGAGCCTATGGAGGTTGAAACCTCAACACGCTCTAG
GCAACAGACCCAGTTTAGGGGATATAATGACCAATTCATGCGTAGCAGACAA
ATGCAGTTTCAAACAACGCCACGCCCCCCCAAATAATCAAGGCGTTGAGCCT
ATGGAGGTTGATAGCTGAACACGCTATAGCCAACAGACCCAGTATAGAAGAA
ATAATGACCAATCCATGCGTAGCAGACAAATGCTGCATCACACGTCCCTGGA
ATCACAGGGGATCAGAATACGATTTGTTAGCACAATCAGAGGTATTAGCAACC
GATGACGATGACGCATCCGACGGCGAACAGTGCAATTTTTTAGGGGAAACTC
CCTGCTACCGTACATTACAAGAACAGTAGCAGGGCATATTATAAACCTCCGGA
TAGATACGGGAGCATCAAAAAATTATATAAAACCACTTCCCTTTCTGAAAACC
CTTACACCAGTTGACAACCTGTTTCAAGTCAATTCTATACACGGGCATACCAA
GATAGAGCAGAAATACCTTATCTATTTATTTGGGACGAATACTTGTTCGTCCTA

AACAGTTAAAAAAATTTCGACGGCATTATAGGCCTCGATTTATTAAGAAATGT
TGATGCAACCATTAATCTTACGAGCAATTTAATTGCACACAAATTCGGCTCAG
AGCCCCTACAATTTATAAAGTATCAAAACGTGAACTTCATCAAAATTGACGAC
AGTAACATTCCACTGGCGGTCAAAGAAAATTTTAACAAAATGATCATGACGA
AAATGTCACTAGAAAAAACGTCTGGTTATTGATTATAGGAAACTAAATCAAAA
AATAATAGACGACAAATACCCCATACATGCCATTTTCACCATACATGGGTAGG
GCTCAATATTTCACAACATTGGACCTTACTTCGGGTTTCCACCCGATTGAACT
AGCTGAAAGGGATCGAAAGAAAACAGATTCAAAGAGAGGAGAACCTGGCA
TTCATTGTGTCAAGTAAGGGATTAAAAACTTCGCCTGAAAAAATTCATATAAA
AATTTTTATGTCCAACCCCCAAATACTCTATTCGGCCTAAGGTCCTTCTTAGGC
TTATCCAGTTACTACAGGTGTTTCACTAAAGGCTTTGCAGCCATGGCCAGACC
TCCTACAGATATGTTAAAAGGTGATAACGGAAAAGTAAGTACCAACCAATCC
AGGAGAGTTAGAATAAACCTGAACCAAAGCCAACAACAAGCATTCGAAAAG
TTGAAAAACGTCCTAGCATCAAAAGATGTGCTCCTTATTTACCCAAACTTCAA
CAGACCGTTTGACCTCACAACTGATGCTTCGGCACATGGAGACAAATGAGAC
AAACTTCGCGACTAACGAACGAGAACTTCTTGCGATAGTCTGGGCATTAAAA
AACCTAAGAAACTATCTGTACGGAGTCAAGAACCTTAACATCTGCACCGACC
ACCAACCGTTGACATTCACGGTATCGGACAGAAACCCAAACGCAAAAATAA
AACGCTGAAAGCTTTTATCGACGAACACAACGACAATATCATTTATACACCAG
GCAAGGAAAATCGTGTAGCTGATGCCTTGTCCCGTCAAAATGTTAACGCGCT
AGACAATCACCCTGACTCGAACGAATCCGACAATGACTCCAACATTGCTACA
ATTCACAGCGAACAATCTCTTACATACTCAGTTGAGACATCCGACATCCATCT
GTTAAAACCTTTATACTATTCAAGGACAAGCACCGACAATATAATCCAATTTA
CAGACCGAGAAAGTCTATGGCACACTGTACTAGACTCAGTAAATGCAAACGT
TGTGAATGCCATACACTGCGAACTCCCAATACTAGCCTTCCTACAACACAGAC
TAATAGAAACATTCCCCTCAACGACATTCAGGTATTGTAAATACGTGGTAACG
GACATTACTGACAAGACAGAACAAACCGAAATAATAACCACAGAACATAAC
AGAGCACATCGCGTAGCCCAAGAAAACGTCAAGCAGATATTGCGCGACTACT
TCTTTTCGAAAATGAGCCGCACATTCTGTCATCATCTTAATCTTAATTATTGCG
ATGGCGATCGCTACAACAAAAATAACTGACTACTCACATACGGACTATATACC
AATTATGGACGGAAACGTAACGATTTGGGACGAGTACTTAGGACACATGACA
AACGTGACATCCTACGAGATATACGCAGACGAGACCAAAAGGACTATTGACT
TATTACAAAAGGATCACATGAAAAGGATTCTTACTACAGACCTCGGACACATT
GACACGTTGATCGCTACAATAAGAGTAAAACATAGAAATAAGAGTAGTATTAA
CCCGTTAGGGACCGCATTAAAAGTAGTAGCCGGCACACCAGATTTTGACGAC
TGGGAACAGATAAGGTTCCGGCAGGAACAATTGTTAGAGTCAGAAAAAAGA
CAGGTAGAAATAAACTACAAATTACAAAATCGATTGAACCTTTTGACAAAAA
CCTTAAACGATATCAACAAAGCTGACGAAATATACACCGAACATTTATTTGAA
ACAATGCTAGCAAAAAATAGAATACAAATAGAGTAAACTAGTAATGTTTAGA
AAATGGTTTGAAAAAATGGTAACTTCACTCACCTTGGCAAAAATCCATTTGAT
CAACCCTGTCATTTTGGACGATATTGATATAAAAGAAATTAACAATGAACAAC
TCACGAATGCCAGCGTAGCTGACATTCTAGAAGTAGCCAAAGCTAAAGTTTT
CCAAAACAATAATATGATATATTTTTTAATTACATTTCCAAACCCTGAGTTAGT

CTGTAAAAAGATCAAAATTTTCCCCGTTCAACATAAAAATACAATATTAAACT
TAGAAGACAGCAACATATTCGCAGATTGCGGCTCAAAAACCATTGCAATAAA
CCAATGCGAAGCCACAGTGAGCACGACATTCTGCAAAACAACAAGTACACC
CACCTGTGCCGAACAACTAACCGTCGCACAATGCGGCACCCGATCCAGCCAC
CTAAATCCAATAACAGAGGTCGACGAAGGTATCATCATCATTAATGACGCTGC
CATGAGAGTCACTGATCAAGAAGGCCTCAACCGAACAATCACGAGGTCTTAC
CTCATAACCTACGTTGATCGGGTCTCCCTAAATGGGACATTGTTCATCAACCA
ACCAAGTCTCTGAAACAGAAGCCAGCAGCATCCGTCGGGACTCAAATCAAT
GTCACGAGCCACAAAAATCACCACAGCCTGCCCTACCTCCATGAGCTAAGCT
TGAAGAATCTCATATGTAGCTTAAAAGCTGAAGTATTGTCGAGACCGATCGTA
AGCAGTGTCATCACTTTCACCATCTCACTCGCCTGTTTCGGCATTGTTTACATC
GCCTATCACTGGTTCAAACGATAACGCCTAATCGAACCAGAGACAGCTCTGC
AGAGACCCGAGGACGGCCCTCACTTAACTAAGGGAGG

>GYPSY8_LTRGypsy#LTR/GYPSY8_LTRGypsy

TGAGGAGTCTTAAAACTCCCCATAAATCTCGTGTAGGGGAGTGGCATAGCAA
CAGCCGCGGTGCAGGGTAAAGCGGAAAGACCGTGAATTACAGTACAGTACT
GGACCTTGAACTCCAGCGGGTCAAGGGCGAAGAGGTCGAACGGTAACGGTG
CGTGCACAAAGAAGACGGACGGTGAACTAACGGTATCGCTCTGAAACTTGG
AGCGGGCCAAGGGCAAATAGGTCGAACGGTAACGGTGCGTGCGCAAAGAG
GACGCACCGTGAATTAAAGGCACAATATGTGGACCTTGTACCCGGGCGG

>GYPSY8_IGypsy#LTR/GYPSY8_IGypsy

GCCGTGCGCAAAAAGGGAAATAACGGGATCCCTGCGGCCTATAAAAGGTAG
GCGCTAGCACGGATGCGGGGCAGTCAGTGAGACAGCAAGATCGCGCGTGGA
AGGAGATAGTGCGTCGAAGCGTTACCAGTAGGAGTTCGGGAGTGAGAGCGC
GCGGTCAAGTAACAAACAGTGGATGCGTCGGCGGCAAAAAGCCCGAAGGAC
ACGGAAGGAAGATCACTACAAGCGAGTACAGAAGGTGCCAGGATGACACCG
CAGGCCCGTCGAGGAACGACAACAACAGATGAGAACTGGACCCGGTGCGG
CGCGGTTTTGTGCCAAACCCAGCACAAGCCTGCCACAGATGCGGTGGCCAG
GGCCACTGGTCTCAGGAGTGCAGGAACCGGCCGATTAATTTTTGCTGGACCT
GCGGGAGAATCGGTCCAAGGACTTCGGAATGCTGCCACAGATCGGGAAACG
CCATGCGACCCCAGCCTCAGAGGGGCAACCAGGGTTCGCAAGAATTAATGG
CCAAACTGAAGGCGGAAGAAAGGCAACTGTCCGCCACAGTGCTCATAGACG
GAGTAGAGATAAAAGCCACTGGCGGACAGGTTGCAGGCGGCAGGAGAGGTC
TTACCTACGAGGAGAGAAGTGAGAATGGCAGATGGACGGTACGAAGAAGTC
ACATCGGTGATCGAAGTCAACATAGGATTTGGCGAGAGGACCGTTCGGATGC
AACTGCTGATCCTACACAACATAATCGACGCACTGGTATTGGGTTGGGATTTC
CTAACGAGAGTGGGAGCACGTATGGAGTGCGCCGGACTGAGCGTAACAATA
CCAGTTTGCTCAACGGGACAAAGCAGGCCAAGGGAAAAGCTTTCAGTGGCA
GTCGTGGAGAGGGCTGACTTCTCAGAGAAGGACGTGGACGAATTCCTGAGG
TCGGAGTTGACCAGTTTAGAAAACATACAAGGGACGTCAACCGTGACAGTG
CACCGAATAACGATGAAGGACGATCAACCAGTCAAGCAGCGGTACTATCCGA

AAAACCCCAAGACTATGGGCTGGGCGCAGCCCTCACACAGCATTCCGAGCG
AGGCGAGCGAGTAATCTCCTACTCCAGTAGAACGCTGAACGCAGCGGAAAG
AAACTACTCTGCAACGGAAAAGGAATGTTTAGCAATATTATGGGTCGTCAGA
AAGTTGAGACCGTACCTGGAGGGCCGATGTCATCACGGACCATATGGCTCTG
AAGTGGCTGAATAGCATTTAGAGTCCGTCCGGCAGGATAGCAAGGTGGGAGT
TAGAGCTGCAACAGTACGATTTCGAAATCTCGTACAGAAAGGGCCAGTTGAA
CATCGTCGCGGACGCGCTTTCAAGACAACCTCTGCAGGAGACGAGCCGAAG
AGTGAGCGTGGAGGACGACGAACAATGGAGAGAGCAGCAGGGATGGAGGA
GATGCAAAGAAAAGTGAAGCAGCAGCCGCAGAAATTTCCGGACTATTTGGA
GGAGGACGGCAAGCTTCCGCATCGGGCGGGCAACGAGGATGTGGCATCGTG
TAAGATGTGTGTACCAATCGGTCAGAGGCAGTGCGTCATGACCGAAAACTAC
GACATCCAACTACGGGACACTTGGGAAGTAGCAAGACGATTGCCAGGATTGC
AGCTCGTTTTTATTGGCCGGGAATGCATCGAGACATAAGAAAGTACGTGCGA
AACTGCGAGAGATGCATGAAGTACAAACCCAGCCAGCTACAGGCGGCCGAA
GAAAAGGTTAACACAGGAGCCGGTAGAAGCATGGGCAACCGTGTGCGCAGA
TTTCGTGGCCCCTTGCTGCGGTTGAAACACGGAAATGTTCGTGCAGTTCACA
AGTAGGGCGTTCAGGAGGTTTCTGGATGAGCTGGGGGTGCGACACCAGCTC
ACGGCTCCATATACTCCGCAAGAGAACCCTACGGAAAGGGCCAACAGGACC
GTCAAAACAATGATCGCTTAATTCACAAGTGCCGATCAGAGGACATGGGACG
AGCAGTGGCCGGAGCTGCAGCTGGCGGTTAACACAAGTGTGGCGGATCACA
GGATACTCGCCGGCGTTCATAACACAGGGAAGGGAGCCGAGTCTGCCCAAC
GCGTTGTTTACGAAAAGACGACCGGGACCGGCAAGTGCACGCAGACACCAG
CGGAAAACGCGGAGAAATTGAAGGATATCTTCGAGATGGTGCGGAGAAACA
TGGAGAGGGCGGCACAGGATCAGGCACGCCACTATAATCTCCGAAGACGAA
GGTGGGGGACACAGCATGGGCCAAAGAGCACCACCTGTCAAAGGCAGCCG
AAGGTTTCGAGGCAAAATTGGCCCCGAGATTTGACGGGCCGTACACAATAAA
GAAGTTCACATCACCAGTAATATGCGTCCTAGAACACAACACAACCAAAAAA
GAAAAGACGGCACACATCAGCGATCTGAAGCCGGGAAGTGCGGGCGCCGGC
GTGCCAGGAGACTTCGAGGAATAAACGGAAAAAAAAACACAAAACGTTGGT
GAGGAAGGGGGGAAGACGATACAAGGTATCGATACGGATCAGAAGTCAACA
TCACACACACACACACACAGGCCCGTCAGAGCGCGAGAGCGGGACAGGGA
GGGTACGCAGCCAAGAACCGTTAACGGGTAACACACACACTCAGCTGCGAA
TTCCGTGGGAGGCGAGTGTACGGACGAGAAAAGCTCCGTTACCGGTACGGG
CCCACGGAATAACGGCACAAGGAGCAGAAAGAAAACCGAGGTTTCGTTGCG
GAGAGTGCGGCTCGAGTCTGTCTTTTCAAATTCGAGATCGTCATGGCAGCAC
ACAGTTTTCACGGCCGGGGAGTACGAGAGACCGGCGCCGGGGAACAGCCCG
GGGCGATATGGGATTCCGATTCCTATCTCCAGCTCCTGGCGTCACCCCTGGTA
TCGCGATCTCCATCCCCGGGGAGTGGGGAGGACCCGGAGGAAATTCCGGAC
GTGGAACTTGAGGAAGACCAACCGATGGCGGATACGGAGGGCGCGGCTGAG
GTCATCACCCTCTCTTCGGAGAGTGAGGAACATGACGACGGAGGCGAAACG
GTCACCCCGGAGTTGTCATCGGACGAGGATGAGACCGCGCGGATGGCTTACC
GAAGCGTCCGGCGGGCTACGCAGGGCCAGTCGTGCCGCCGACCGAGGATCC
GGTGGACATGAAGAGATTCATGGAGGAGCGAGTCGCCACCCTGCGGCGATT

CCAGCAGATGGTGGATGAGCGAAAGGTCGCCGACGCCAAGGCCGAATGGCA
GGAGCGGTTGGCTTGGCTGCAAGAGGAAGAAGCGTTGTGGGCACCAACAG
GGGAGAAGAGCCCGGGAGAAGCGAACCCGGAGAAGAGTGGAGCTGGCCCG
CGCCAGATGAGGCGCCGCCACCACCAAAGCCGCGCGGCAAGGGGCGCGGG
GGCGAAAGGTGCACTGGGCGAAGTTGTTAGAGCTTGGCGGGCTTCAATGTG
CGGGGGGACGGAGAGGTAAAAGTCACGATATCAGAATAAAAGATACCGGAC
GGCAAAAAACAAACCGTATGTCCGAGTTTGTCGTAAACGGGCACTGGCACA
ATAACTTAAACAAAAAATGGTTGCGGGTGTCATCTGCAGCAGCCGAATTAAC
GCGCGAGGGGGTGACGCGCGGCCAGGCACGCAGGCCGGCAAACGACGCGT
GGCTGCTGCTGGCGCGGGACGAAGGAGGGGCCGTTGCAGATGCAAGCGATC
CTCCGGGGCGCTCGGGGCCAGGGGCAGCCACCCCAGAGCATGAAGGGGCAT
ACATGTGTTGGTGGAGCTGGAAAGAAGAGAGGATTCGTTAATATGGCGATAC
GCAAGAAAGCGTTAGACTCACCTTTCAATGAGCTGCTACACCGGATCACGTG
TGAAAAAGAATTGGGATGGTGGCGTCGCGATAATGGCGACCGATCGATAGTG
TGATCGATAGAAATACTGGATGAGCACGGATGCGCAAATGCGGCCACACTGT
GGGCAGCGATCCGCACGCGATGGCAGCGCCGGAATATCGGTAGTGACAACG
CCGCTTACCGATATCGATAACACGAGTTCGGCAATGTTGCCAGGCGGGAAAA
TTCAAATATCGAACTGGTGGAGCAGAGCAGACAGCAGCAGCACATGGAAGG
TGAAAGAAGACCGCTAGGACAGAGGAGAACGGAGAAGGATATGTGAAGGG
TCAAGATGCCCCGTCGAGATGGCTAACGCCAAAGCTGAGACCAGAAGGATG
TAGGACAACGAGCGGAGAAGTTGGAAGGAAGAGACACCGGAGCGGAGGAC
CGTCGGGATCATTAAGTTTTAAAATCTTCTGAAGAAAGGGGGAGAT

>GYPSY7_LTRGypsy#LTR/GYPSY7_LTRGypsy

AGTTAACACATTCAACGAGCAGCAAAGAGAGGCCAATGCAAGTACTGAAGC
TTTCACGATAAGAGGCGAGCTTTTGGCTTGGGATCAAAGGCGAGCTTAAGAA
ATAAGGGAAGAGCGAATTGGATCATCGAACGCGTCGGTCGTCGCCATGCAGA
CACATATTATTCAATAAACTTACAAGCTCAAATATAATCCAAATATAATTCGTA
GGCCATAGACACGACATCCATAAAAACACAAATCTATTAACT

>GYPSY7_IGypsy#LTR/GYPSY7_IGypsy

GGCGCCCAACATTCACCTACATTTCCCGAGACAACAAAACTACGTTCAACCG
ACTTCAACTGCGGATTTTCTTTCAACATCAACAATCAACATCAATACAATAAC
AAATATTAATTAATAAAAAAAGGATTAACTTAACTTCTGTAAGTACAACGCAT
ATTGGTTACTTAATAAGATCTCTAACTTTCATAACGACCTTTAAATTAACGAAG
GTTATTGATGGTTATTAATGACAAATAGAAGAAGACGAAGTTTTAGGCGACTT
AGAGTAAAGTTTGAATAGGTGGCGAGAGCATTTACAAAACCAAAGTGTGGG
AGGTGGGAGAGAAGAAAATTGATGGTTAAAGAAGAATAAAGCTTGCGAAAT
GAACTTATCGGCAGATCATTTGAAAGCAATAGTGGATAGTGCCGTTGCTGGTG
CGATTGCTAGTCAAAAATAAATATTTGATAAACAGCTGCAATAAATAAGTGCG
CGTATAGAAAAAATTACAGTGAACACCCCAGAAGTGGAAACTTATAAAGATG
CTGAAATTGTACCAGGTGTAAGATGTACTGAACCATTAGACATAGTAAAGTCT
TTGCCAGATTTTGACGGAAAAAGTGAAACCTATGTGTCATGGAGAAAAGCTG

CCCATGTCGCTTTTAAAGATTTCGAGAATTACGAGGGCAGTTCAACATACCAT
CAAGCACTTGGCATAATGCGAAAATAAAAAACTCGGCAAACACTGTGCTGGC
GTAGTTTAACACTCCGCTGAATTTCAAGGCAATGATAAATCGTCTCGATTTTA
CATACGCCGACAAAAGGCTAGTATATTTAATAGAGCAGGAGTTATCGACGTTA
AGAGAGGGCGACATGGCAATGACTGAGTTTTACGACGAAGTTGAAAAAAAA
CTTACTCTCCTTACAAACAAGACAATAATGACATGCGATACCACTTTAGCGAT
TAACGAAAAATACAGGTCAGACGCGTGTATTCATAAGGGGAACATAAAAGTC
TTTGAGAGAGACTTACCAACAGCTCTCGCTCTAGCACAGGAAGTCGAATCAA
ACCACGAACGCTACCAATTTGCACTGAACTATTCTAGAAGCTTAGGAGAAGG
CCAGAAGGCTGAGAAAAAGCAAACTGATAGGGATTGGCATACGAACGTTCA
TCAATAGGGCATAAACCCTCATTTTAGTAAACGGAAGCCAGTGCCCAACCCT
GGCAATCAGGCAAGTCAGACACGACACACATGATCAGTTAATGGACACTGAC
GTATCAAAGAGAACTATTAGAACGGGTCAATACCAACCCGGGCAGCCACCTT
CCTTGCATCAGGAAAGCGTACCCTTACATCAAAACACTTGGCCGGCTCAGCA
ACAAAACACACGGCAATACAGGCAAACTAGCTATGATGCAACAAAAAGACC
CTATAGTGGAACTGGCTAACAGCACCAAAGCAAAAAAGAATTAACCATCTAG
CTTACGAAGAAGATTTAGCTGATACAGAAAATTACGAACAGGAAGCAGAAGT
GGCTGTGGAGGAATGGGAGGACGAACTTGCATTTCATGATAGTGTTCATTTTT
TAGACTTAAGTCCCTGTTACCATTCATTGAAAGGAAAATTGCAGGGAGAACC
ATAAAACTCTTGATTGGCACCGGGTCGTCGAAAAACTACATACAGCCTCTTG
CAGAACTAAAATATATAATGCCGGTACAAAAAGAATTTAAAGTAAAATCGCTT
CACGGTTACAACACAATAAAACTTAAATGCCTAGTTAAGATATTCGATAAAAA
CGTTCCATTTTTCATTCTTCCAGACCTCTCAAGCTTCGACGCCATAATAGGTCT
TACCACATTGACACAGACAAATGCAATATTGGACCTAAAAAATAAAACTTTAA
AAACTGGTAATACAGTTTAACCAATTAAATTCATAAGGTGTAACAGCGTTAAT
TTCTCCGATATTAAGGACATCATCGTACCTAAGCCAATTGCTGATAAATTTCAC
ACTATGCTGGCAGACAGAGTGGGTGTCTTCGCAGAGCCGGAAAAAGCACTG
CCTTACAATACACATATTGTTGCCACCATACGTACCCAAGATAATCAACCAATA
TACACAAGACTTTACCCTTACCCCATGAGTGTGGCAGATTTTGTGAATAAGGA
GATGAAAGCATTGTTAAAAGACGGCATAATCCGACCCGCACGATCACCATATA
ATAGTCCGGTTTGGGTAGTCGATAAAAAAGGCACAGATGATCAGGGGTGTAG
ACATAAAAGAATGGTTATAGATTTTCGAAAGCTTAACTTTAAGACCGTGGACG
ACAAATATCCGATACCAAATATAACATGCATACTGTCAAATCTGGGGAAAGCC
AGGTTTTTCAGCACGTTAGACCCTAAATCAGGATTCCATCAAATCCTACTCGC
CGAAAAGGATAGAGAGAAAACAGCCTTTTAAATTGCAAACGGCAAGTACGA
GTTTTGCTGACTTCCCTTCGGCCTAAAAAATCCACCTAGTATACTTCAACGTG
CCATAGACGATGTACTTAGGGATGAAATAAGAAAGTCAATTATTTTTTTAGAA
CGACTAGAGGATCATGTGGATCATATTCGGTGGGAACTGGATAGATTATTCGA
AGCTAACAGAGAGTTTCTAGGAAAAAATCTCAATTTTTAAAGAAAGCGTCGA
ATATTTAGGATTCATAGTATCTAGCGGGGGTATTGAAACCAATCCTTATAAGGC
AGAGGCTATTAAAACCTATTAAGAACCCACAAATTTTTTCAACGTGAGATCCT
GTTTGGGACTAGCGAGTTATCGCAGTTTTATAAAGGACTTTGCATCTATAGCC
AGACCATTGAGTGACATTCTTAAAGGCGAAAATGCCCAAGTTTCAGCCAGTC

GCTCTAAGAAGATTCAAGTTAGCCACGACACTAAACAACGTTCTGCCTTTGA
AAAACTTAAGAATATCCTTACATCCGAAAACGTAATGCTGCTTTATCCAGACT
ATAAAAAGCCCTTCGACTTGACAACGGACGCCTCGGCACTGGGCCTCGGAG
CGGTTTTATCCCAAGGCGGCAAGCCTATTACAATGATATCTAGGACTTTAAAG
GATAGAGAGCTCAGTTTCGCAACAAACGAACACGAACTCCTAGCTATAGAAA
GAGAAAGAGTTCCTTAAAAAGTTTAAGGAACTATCTGTATGACGTCAAAAAT
CTGAACATTCACACTGATCACCAGCCGCTAATTTTTGCCGTTTCAGACAAAA
ACCCAAACGCAGAAATTAAAAGGTGGAAGGCGTTTATAGACGAACATTTCTA
TAAATGTCTCTAACATTTTCTATAAGGCAGGGAAGGAAAACTTCGTTGCTGAT
GCACTATCTAGACAAGCCATTCATGCTGTTGAAAGCGACGCTCGGTCAGATAT
ATCGACAATTCATAGCGAAATTTCACTTTCATATACCATTGAAACAGTCGACA
AGCCAGTCAATTGTTTCGGAAATCAGATAGTCTTAGAAGAAGGCACAACATA
TTGTACTCGTACGTTTGTTATATTTAGAAATAACTCGAGACATTTGATACAATT
CGCAAACAGGGAAACTTTGGTTGGCAGAATCCGTGACGTGGTTAAACAGGA
TGTAACGAATGCAATATACTGCGAACTGCCCGTATTGGCATTCATACAAAACA
GACTTGTGGAAGAGTTTCCTAGAACGACATTTCGACATACTAAAAAAATTGT
CAACGACATTTATAACAAAGACGAACAAAGGGAAATAGTAACCATCGAGCAT
AGCAGAGCACATAGGGCAGCACAGGAGAATGTAAAACAGATTTTACAAAATT
ATTTTTTTTCCCAAAATGTCACAGTTAGTAGCCAGTATTGTAAGCGACTGCTT
AGTCTGTACAAAAGCCAAATACGACCGTCATCCTCGAAAACAAGTCCTTGGG
AAAACACCTATTCCCTCACTAATTGGCGAGACATTACATATAAATATTTTCTCC
ACTGACAGAAAGTTCTCCAAATTCGCGATAGTGCAACCGATAGGCTCTCGAA
CAATAGCTGATATAGAACCTGCAATAATGCAGCTAATGAATTTCTATCCATGGG
TAAAAACAATTTATTGTGACAACGAACCATCTATGAACTCTCAATCTATCAGG
TCACTCTTACTAAACCGATTCAATGTAACAGTGGAGAATGCACCGCCACTCC
ATAGTATTTCCAATGGACAGGTGGAAAGGTTTCACAGCAAATTGGTAGAGAT
AGCACGATGTTTAAAACTAGAAAGAGGCCTAGATGATACGGTCAACCTCTTA
CTCCAGGCAGCCATTGAACATAATAAACGAACGAACTGTCACTAACAAAAGA
CCGATCGACATTATTCATGCAATGCCTCCCGAACTTGCAGAAGAAATAACAA
ATAGAATTGACAAAGCACAGGAGATACAGTTAAAAAGGATGAACGAATCAA
GGTCTTTTTTTCAGGTTGGGAAAACGGTCTTGGTAAAACAAAATAAACACTT
AGGAAATAAAATCACCCCACGGTACAAGGAGGAAAAGATTGATGCAGATATG
GGAACTAATGTCCTTATAAATGGAAGGGTAGTCCATAAAGATAATCTACGCTA
GGTATCTGTTGTTTAAATAAATAAGGATTTGGCAATTATTTAATTAAATAAATCT
TTGGCAAGTCTACTTCCCCAGAAGATAGAAAAGAATTCGAATCCAAAAATTG
GAAAAAAACCTTTACCAGTATTTTTCAATAACAAAAAAAAAGAAAAAAAAA
AACAACAAACTTAACCAATTGGCACTGTGCATAGTTTCTTAATATATTTATATT
ATATCCAATTATACTAGTCTTTAATGTTTGTAATTTGTAATGAATAAGGCACGGT
CATCGCTTTAGCCATTATAAGAGGTAGCCGGCAATGTTATCGTCACCACACAT
AAACCTTCTCAAGAAGTCTGTGGACAGTCTTCGTCTTAGAAGGGGAGG

>GYPSY6A_LTRGypsy#LTR/GYPSY6A_LTRGypsy

AGTTAACACAAGCCAACGAACTAGAACAGTTGGCTCAGTGCATGAAACATA
ATATCAAAGTGCTGACCCAGCATTGGGCCGGAAACCCGTGCGCAGCCGGCA
AACACAGCATATTTGCGTGGCCGCTGCGAGTCTTTGTTAGCTATAAGAATCAT
TTTAAGTTTCAGTTTGAATTTGCATTTACCAGAATAAAGAGCGGCAGCTCGTC
AACTCCGACTAAGCGTCGTAAAACATATTTTTATTACTTACTCTGCCAGACCA
CAAGGCTGGCAGTTAAAAGAGGAAAGTTCCTCTCAACGTAAATAATAATCTT
CTGCTCCCCGGACTCCCCGGAGCAGCCAGCGAAGGAAGCCCTTCACCTCTG
GAGGAATCCTGACGACGACAGTATCGCAAGACGGTGCGATCAATCTTTAAAT
AATT

>GYPSY6_LTRGypsy#LTR/GYPSY6_LTRGypsy

AGTTAACTAAGTTAACCGGGCGACGGCTACGACCGATCGTCCGAAGACTCGC
TCCGGCCAAATTGCTGACACAGCGTCTGGCCGGAAGCCCGTGCATAGCCGGC
AAACACTACCCATTGGAGTGGCCGCTATGAGTTTCATATTTTGTTAGCCTTAA
GTTCAGTTTGATTTTATATTCAATAAAGAGCGCATCGCGCCTTGAATCGACTCC
AGCTTTTGCTGTCATTATTAAATTGGTTGGTTGGTTGACTAGCCTTAAGGGCA
CTCAACAACGGAGAGACGTTCTCCCACCATATCTTCTAATTCTGAGGAGAAG
AGGTCTGCGGCAACCGCCCTGCCTCCCGGATACCTGCAACCTACGCCGGAGA
CCGCGGCGAGGGATCTACACCAACATAATTAGTTTAATT

>GYPSY6_IGypsy#LTR/GYPSY6_IGypsy

GGCGCTCAACTAGCGGGAAGTACGCACCCGCCCACATCATACAGAACATGTA
CATCTTCGACACAAACCATAGTTTCAGACCAGTGTTAAAATATTAAATAAACC
AGTCTGAAGAGTGTTTTTAAAATCTCAATATTGAATGTGACGGCTAATAGGTG
TTTGGTGTTTATTTTCACTGCGAAAAAAATTTAATTAACATCATAATCCGTTGC
AAACACGGCAAAAAAAAAAAAAAAAAAAAAAAATAGCCTAATTTAATTCGCT
CACAGTTGTGTTACGGCGCATTGCTATTTCGCCGAGCAGTTAAATTTTTTTTT
TGCAAATATTTTTGGTGAGGAAATTAACATATTGCGGGGGCTAAATATCAGAG
CCAAGGCTCTAACAGTTTAATATACCAGATTTGAATTTTTTATTTGACATTTAA
TTCAGTTTTGTATACTTGGTATTAGCTTAACAGTCCATTTTGTATCTTTTATTGT
CCCCACTCCACCGCTGTGAACCCGATACGGCCGCGCATGCATTTGTATTTCAA
TTATTTATTCTTCGCTTCTTTGTTTATAGTGGCGGAAGCTCAGCTCCCACCCAC
CCCTTACCTTGCCGCTGCTCCGCGAGGTGACGGCTTGGTTCGAACAGCTTTAT
ACATCGTTGCAAAGGCGGCGGCTACAGTCCCCGCGCACCCCTCACCCTACCG
GTGCTTCTTTGTTTATCGTGGCGGAGGCTCAGTCCCCGCGCACCCCTCACCAT
ACCAGTGTTGGCACTTGGAAAGGCGGCAACTGGGCTCACATACATAGTTGTT
AAGGCGGCGGCTCCGTCTCCGCGTACTTCTTGCGTTGCCAGCTGCCTTCGTG
GGCAAAATAGCTGAGCGGGCATTGCATACCAGTGCAGTTTAACCTTAAGCGC
ATATGTGCTCCCCGTTTACACAATATAGGGAACATAAACCTAGCGACTCAGAT
TCTGACAGTGAAGGGCCCCCGCGTATTTTCATCCCAATACGGGCACCCACCA
ACCCCCCTTCAGGTAGAACCATGGACTCAGATCAGCTTAAGGCTGTGATTCA
GACCGCCGTTAATCGTGCCTTGGCAGAAGCAGCCGGTGAAGCCAGGCGTAG
AGAAGAAGAAATGCGCCAAGTTATACAACAGTTGGCTACCCAAGTTGCAGC

GGTGCAAATCGCACCCACACAAGCGGCAGTCCCTATAATCAAAGTATACCAA
CCCATTGATATCACGGGCAACGTCGAGTGCAGCGAGCCTCTGGATGCCGTAA
AATGCTTGCCCGAATTTACGGGAGCACAAGAGACATATGTCTCCTGGCGGCA
GGCGGCGGTAGCCGCATATTACATATTTAGGAATTATGTAAATAGCTCACGCCA
CTACCAGGCGGTCGTTATAATCAGAAGTAAAATAAGAGGCCCCGCCGATGCG
GTGCTATCTTCGTTCGGCACTGTATTGAATTTCGATGCGATCATAGATCGCCTC
GATTTCACATATAGTGACAAACGCGCGATTCACGTCATCGAGCAGGAAATGG
GCACCCTCAGACAGGGAAGCCTGACTCTATTACAATACTACGACGAGGTCGA
GAAAAAGCTCACCTTGCTTACCAATAAGGCCACCATGTCGTATGAAACGGCG
GCGGCAAAAATCTTATGCGACAAATTCCGGGATGATGCGCTCCGTATATTTATT
TCGGGACTCAAGCGCAGTCTTTCCGATGTCCTCTTCTCGGCGAAGCCGAAGG
ACATGTCAACTGCGTTGGCATTAGCTCAGGAAGTGGAATCAAACCACGAAAG
GTACACAGTCGCAACATCGTTTGCAAAAAGCCTGGAGGATAGGGATAGGAA
GCAATACCCAATGGCGCAAGAACGCCAACAAACGCCCTACCAAGCGCACTC
CCAAGTAAGCAGGGGAAAAAACCCACACTTTATTAAACAGGTTAAGGCTCA
GGTACACTCGGCTCCGCATAACGACAGGCGTCGTGAAAACACATCAGAACC
CATTGGAGGTTGACCCGTCTATGTCCAGGTTGAGACAACCCACTCAGGCCTA
CCAAAACGGGAAGCCCGCCCACTCCGGTCGTTCACACCCTCACAAGAGACA
GAGGGTTAATCACATCGCTCAGACCATAGGTCAGGCCGAAGGCACGTACGCA
ACCACAGCGTCCAGCGCGGCGGCCAAAGTGGATGACGACACCATTTCTGAA
TATGATTTAGAAGTGATTAATTTTTAGGGGAAAATCCCTGCTGCCCGTCATCA
GACGAAGAGTAGCGGGGAAAGAGATGAAGTTCCTCATCGACACGGGTGCGT
CAAAAAATTTATCCGGCCTCACAAAGGCTTAAAAGGCGTTCGCCCCGTCGA
TTCCCCATTCACCATCCATTCACTCCATGGCGTTACCACGATCACGAAAAAT
GCTTCGTGTCACTTTTTGATTGGAAGGCCACGTTCTTCATTTTACCTGATTTGT
CCTCCTTTGACGCGATAATTGGCCTCGACCTACTTAAACAGGCAGGGGCATC
GCTTTGTCTGGCCTCTGGCCACCTCAGATGGGGCAATGGAGAAGAGAAAATT
GAATTCCACCCATGCCCCGACGTCAATTTTACCGAAGTGGACTGCTCAGATG
CGCCACCCTTGGTCAGAAACGCGTTCTTAGAAATGCTGAAGACTAGGAAGA
AGGCTTTTGCAAACCCTAACGAGGCTTTGCCCTATAACAGATCGGTGGTAGC
TACCATCCGAACAGTTAGTGAGGAGCCCATATACGCAAAGTTATACCCGTACC
CGATGGGGACGGCGGACTTCGTCAATAAAGAGATTGAAGACCTGCTTAAAA
ACGGGATAATTCAGAAGTCGGTATCCCCTTACAACAACCCGATATGGGTTGTA
TATAAGAAAGGGACCGATGACCATGGCAACCGGAAAATGAGACTAGTTATCG
ACTTCCGCAAGCTAAACGAAAGAACAGTGCCCGATAAATATCCCATGCCAAA
TATTAACATGATATTGAGCAACCTAGGCAATGCGAAGTATTTCTCCACGCTGG
ACCTCAAGTCTGGCTATCACCAAATCATTCTTGCAGAACGCGACAGGGAAAA
AACCTCTTTTTCTGTGAATGGGGGAAAATACGAATTTCGCAGATTACCGTTTG
GCCTCAAAAATGCAGGTAGCATTTTCCAGAGGACAATCGATGACATCCTACG
GGAACAAATCGGCAAGTTCTGCTATGTTTACGTTGACGACGTAATTATCTACT
CCGAAGACGAAAACTCTCACATCAAGCACGTAGATTGGGTTCTAAAGAGCCT
GCACGATGCGAACATGAGAGTATCGGCAGAAAAGTCCAGCTTTTTAAGAAA
AGTGTGAGCTTCCTTGGGTTTATAGTCACTTGTAACGGTGCTACAACAGACCC

AGAAAAGGTTAAGGCTATAAAAGAATTTCCGGAACCCAAAAGTGTTTTTGAG
GTACGGTCATTTCTAGGCCTAGCCAGCTACTACAGATGTTTTATTAAGGACTT
CGCGGCAATAGCAAGGCCTATATCAGACATCCTAAAAGGGGAAAACGGAAC
AGTTAGTAGACACAGGTCACGAAACATTCCGGTTCAGTTCTCGGAGACGCAG
CAACAAGCGTTCCAGAAACTACGAAACATCTTGGCATCCGACGACGTGATGC
TAAGGTACCCCGACTACAAAAAGGCATTTGATCTAACGACAGATGCCTCGGC
CCATGGCATTGGCGCAGTATTGTCCCAAGAGGGACGCCCAATAACAATGATC
TCAAGGACATTGAAGGACAGGGAGGTTAACTACGCCACCAATGAGAGGGAA
CTCTTAGCCATAGTCTGGGCTTTGGCCAAGCTACGGCATTACCTGTATGCGGT
TAAAGACATCAACATCTTTACCGACCACCAGCCGCTAACCTTTGCAGTATCGG
AATCCAATCCGAATGCAAAGATTAAAAGGTGGAAAGCGCGCATTGATGAATC
AGGAGCGCGTATTTTCTACAAACCCGGCAAAGAAAACTTGGTCGCCGATGCA
TTGTCACGGCAACAAATTAACGTCATGGAGGAGCAAGAAGCTCAATCGTGCG
TGGCCACTGTTCACAGCGAACTCTCCTTGACGCACACTATCGAAACGACGGA
TAAGCCCCTAAACTGCTTTCAGAACCAGATAACTCTGGAGGAGGCACGCTTT
CCGTTAAAGCGCAGCTTCGTCCTCTTTGGAAACAAGAGGCGGCATGCGATTA
ACTTCCCCTGCAAAGAGTCATTGATTGATGAACTCGCAGATGTAATCGTTCCG
AAGGGCGTAAACGCCATTCATTGTGACCTGCACACGCTGGCACTAATACAGG
ACGAGTTGGTTCGGAGATTTCCAGCCACCAAATTTTGGCACTGCAAAAACCG
TGTAACGGATATTTTTGCAGTCCCTGAAAGACGGGAAATTCTTACCGTAGAAC
ACAATAGGGCCCACAGGTCGGCCCAAGAAAACGTTAAGCAGGTACTCTCCG
AGTACTACTTTCCGAAGATGACCAAGTTGGCTACCGAAATCGTAGCAAACTG
CAAAACATGCGCAAAAGCCAAATACGATAGGCACCCAAAACAACATGAGCT
AGGCGAGACTCCAATCCCCTCTCATGTGGGAGAACTACTGCACATCGACATT
TTTTCTACGGATAAAAAGTATTTTCTCACTTGCATTGACAAGTTTTCGAAGTT
CGCAATTGTGCAACATGTCCACTCTAGGACAATCGAAGACCTTAAACCGGCC
ATACTACAGATTATGAATTTTTTCCCTAGGGCTAGAGTAGTTTACTGCGATAAT
GAACCTTCATTAAATTCGCATACTATCTCGACCATGCTTGACAATCACTTTGGC
GTTAGCATTGCCAATGCACCGCCACTCCATAGCGTGTCAAATGGGCAAGTGG
AGCGCTTTCATAGCACCCTTTTGGAGCTTGCTCGTTGCCTAAAAATCGACAA
GGGCATAACCGATACTGTGGAAATTATTTGTTGGCAACCGCCAAGTATAATG
AGTCAATTCACTCCGTCGTTGACAAACGACCAGTTGACATCGTGCAGGAGCA
CCCAGATGACCCACAGACGGAAGTCCGGAACAGAATCATTAAGGCACAAAA
CACGCTCAGGACCAGGGAAAACGCCTCCCGACAACACAGAGTATTCGAAGT
CGGCGAGAAGGTATTGGTTAAATCCAATAGAAGGCTGGGAAATAAACTCACA
CCATTGTACGAGGAGAGAGCCGTAGAAGCGGACCTGGGGACCACGGTCCTC
ATCAAGGGGAGGGTGGTCCACAAGGACAATCTTCAATGATATGACCAACAAT
ATCCTTTATGGTTTTTTTTATTATTCATTTTTTATATTATATTTTTTATCACACCTT
CAAGCCATCTGGCGCACTTTATTTTTTATATTCATTAGTTATTACACCTTCAAGC
CGCTTGGCGCACTTTGTTTTTTACTATAGGTTCGTAGCCACTGGCATAGTTTGT
TTTAAGTTCATTTTATCGGTGGTGGGAAAACCCTCTATAAGCATAAAAATAGC
TAAAGCTTAATTTCACAGGATCGGACCAACATTTTGCATACTCCTGCCCTTGG
CGTCGGCCCACGTTACCGATTATTCACAGGCCAGGTACATCCCCGTTATAGAT

GGCGAAATCCTAGTATGGGAGGAGTTTGCTTACGTCACGCACACAGCAAACC
TCTCAGAATATGGGCGTGTAATAGAGGAGACAACCAACATGATAGACATGTTT
CCGCTATCCCATATGAAGAAGCTTCTGAGCGTGGATACCGCCCACCTCCAGG
ACTTGTTAGAGTCGTTGGGCGTTCATCACAGAGTAGCTAGGAGCTTGGATTTC
TTAGGACCTATGCTAAAGGTTGTAGCAGGGACACCAGATTCCAGCGACTTAG
AAAAAATCAGGTTTACCGAAATGAGGTTAGTCGAGTCTAGCAATAGACAGAT
CCAAATTAACACCAAAACCCAAAATCAAATTAATCAACTTACCTCTACCGTCA
ATTCAATTTTGAGATCAGCCAAAACCTCACAAATAGACACTGGACACTTATAT
GAAACACTGCTTGCTAGAAACCGCATGTTTATGGCGGAATTACAAAATTTAAT
GCTTGCAATAACCCTAGCCAGGATTAACATTGTTAGTCCGAACATTTTAGATC
ACGAAGACTTAGAAACAGTTTGGCTTGAGGAACCCACCGACACACCTATAG
GAGATCTTTTGTCCGTCTCGTCTGTAAAGGTTTTACAGTCCCGTAACATTTTA
CACTTTATCATTAAATTCCCCAAAATTAAATTAGCCTGCAAGAAGATCACTATT
TTCCCAGTTGCCCATGGTGGAACGATGTTGCAGATCATAGACAATGTCATAGC
CGAATGCAGCGGAGAAGTTTACGCCATCAAAAACTGCTCCGAATCACCGAG
AGCCACATTCTGCCGCCTAGCTTCAGAGAGTTCGTGCGCCAAAGACCTGCAC
GCCGGTGGGGTAGCACACTGCCGAGTACAAGAGAGTGACCTGCATCCGATA
ACCTACGTAGACGAAGGAATTATTATCATCAACGATAGGTCGGCCAAAGTGC
GAGTGGACAACGGCACAGAAATCTGGACTCATGGCACACACCTCATAACCTT
TGACAAACAGGCCACCATAAATGACACACTCTTCATCAACCACAATAACACC
CAGAAGAGAGCTCCAGGGACAGCAAGTCTTCCCTTGTTGAACATCACCGCC
ACCCAAGATGTCCTCAGCCTCCCGTATCTTCACCGTCTGAGTGAACGAAACT
TGGAGTTCATTAAGGAGTTCAGAGAAGAGATTGAGAACCAAAGAACACGTC
TCGTAGCAATTATTGCAGGAGCAATATGCTGCGCACTCATCTGCATCGGGCTT
ATTTTTAGGCGTTTCACTGAGGCAAGAAAATCCGCAGGCCAAGTTAGGCAGA
TTATCGCTGAACTACAGACGGCCGAGGGCGGCCTTAATTCTGAGGGGGGA

>GYPSY5_LTRGypsy#LTR/GYPSY5_LTRGypsy

AGTTACCACATCTACATCTACTTCCCCACCCCCTAAACCTCCACGCCAGAACC
ATTAGTTCGTTGGCCCACATCTGACCACATCAGCAGTCCACGCCACTACTTCC
CCACCCCCCTAAACCTCCACGCTGAGCCGCTAGTTCGTTGTCCCCTCATGTTG
CAACAGTGTAGGCGGAAACAGCGCAGCAAAACGGAGGCATTCCCCAAATCC
GCTGGGTCAGCAGATTCGCGACAACACAGAGCCATACATAATACTTGATTCTC
CTTTGTAATAAGTCAGTCTTAAGCTACACTCGAGGGGAGTGAGATCAAAATA
AAATCACAACCTTAAAGGGAAATACCTTTTAATTAAAATCGGGAAGAAACAA
TAGAGGGAAAAGAAACACAAAGGACCACAGAAGGAAGACACTGGAAGGCT
TCACTCGTTAATT

>GYPSY5_IGypsy#LTR/GYPSY5_IGypsy

GGCGCAGTCGGAAACTGGACCTGATGCAGCTCCTTTTCATCACAATTGTACTT
AAGTGAGTAGAGTGAGAGAATAAAGAATAATAAAATGCCAATACACAAAATG
AATTGCCACAACACACAAAATACACAACAAATAAATAAGCGAGTTAAAAAG
ATGCAATCATATTTAATGAAAATTACATAACTAAAGAAATTTTTATAAAAGAAG

AAATTAAATATCTAATCATGTAAAAGTTCAAATAGAACAATTATAGTGATTAAA
CAAAATATAAAAATCTTGGTAAAATGATTTAGACAAATCAGAAAATATTAAAA
ATATATTTATGTTGTGAATCAGAATATACAAGTGAGTGATTGAGGCTCGTATGT
TGGAGCGTAGTTAGTGGATCGCGTCAAACTTGTGTGAAAGTTGGCCTCAGCG
GTTGGGTTGATGTTTGAACAGATCGCTGACGGTTTAATTCAAATTCGAAGGC
AACTCAACAGCGACGATACACTGGCGTGTCTAGCGGTTGGGTTGATGTTCGA
ACAAGTTGCGCTAGTTGTCAATACATGTCGAGACAGCTCAACGGCGACGATA
CACTGGCGTGTCTAGCGGTTGGGTTGAAACTCGAACAAGTTGCGCTAGTTGT
CGATTGCACTTCGAAGACAGTTCAGCAGTGACGATACACTGGCGTGTCTAGT
TGTTGGACTGATGTTCGAAGAGGTTGCACTAGTTGTTCATAAGGAACAATTG
GTTCGACGGCATTCGTGACGTGGTTGACCCTCAATTATTAGACTGATTCAATC
GACATTGGTATTCAGGGCGAATATCCAATGCGGCAACAAAACGATCCATCAA
ACCTGTATGAGATAAAAAACACCTACGGCCTGATTTAGGCAAACAATCCAGC
AAATTAAGACAAAATAATAATACAAGAATATATTTTTCTTCTCTCTCTCTCAAT
AAAGATTGATATAAATATGAAATTATAAACACATACTAATGCATCTGTAATTAC
AGATTCAACACGAATCCATCGTTCATCATCTGCAAACAAAAATATTCAACACA
CAATTAATTAAGTATACATAAAAACACACAATTAATTAAGTATACATAAAAACA
CACAATTAATTAAGTTCACGTACACAGATATACACTATTAATTAAATACATACA
CTTAATTTTTTTTTTACACAATTACACAGAATTAAGAATTAAAGGATTTCTCT
AAAAGAGTAAATGTCTAAACTTGCTAAAGTAAAGAAGCTTATTCCGGCGACT
AGAAGTAGTACTCGTTTGAGTGCGAGCTTATCAGACGTTAGGGAAGACCAGG
TTGCCCTACCACCCAGAATTAGACCTTCTTCGAGTAGTGCACCAAGCACAAT
GACTAGTTCGTCAGAGACAAGTAACTTTTCTTCCCCACCCCCTCCAATTTCCC
CTCAGGACATTTGCCTATCCCTAAAAATTCCGGATGGCATTCGCGACCTTACT
CCCTATGATGGAGATCCAACTACCCTAAACGAATTTATAAATAATATCGAGGA
AATTCTTCTTCTCATTAGAGGCACGGATAAAACTCCGTATGGTCAGTTCTTGT
TAAGAGCAATAAAAAACAAGATAGAAGGTAAAGCAAAGGCGTTATTATTATC
ATTAGGCATAGGCTTAAACTGGGATGACATCAAAGAAGCATTAATAGGGAAAT
TCGCTGACAAAAGAAGCGAAGAAACTCTAAACTTTGAACTTCACGGACTTG
TTCACAAGAGATTGCCACTGCAGGAATTTTACGAACGAATTTTAGACATCCA
AGCAACTTACGGTAGAACCATGGAAAACATGGGAATAGAAACAGCAGCACT
AAATCTTAAAAAGGAATCTTTTGAAAAAACATGCTTGTATACGTTTATACACG
GCATTAAAGGCCCATTGGGGTCTACTGTCAGAGCATTTAGACCTAGGAGTCTC
CAAGAAGCCTATGATGTGGCAATTAAGGAAAGGGATATTTTTATGAGAGAAA
ATTGGTCTAGACAAACACAGAGTTCGAGATCAAACAATTACAATTATAATGGG
AGAGATTATCGTAGAAATGACGAACATCGGGTTGAGAAATATAATAAGAAATC
GGAGTTCGACCGAAACATCAGATCTAGGGATCAAAGAGGAAGTTATTCAAAG
CCCAGAGACAACGACAGAAATTTTAACAAAATGAGGGCTATTATGCCAAAAG
AAGAGGACAGAAAACGCTATGACAGAGACAATAATTACTCACGAAATCACG
GAAATCCGGGACAGCAAAGACAGACCCTTAACAATATTGAGACAGCAGGTG
TACAGGAGTTACATAATATTGATGATAAAAATTTTCAATTAAAAGCCTCGTCG
GACAGACGGGATACTTAGAAAAATTTAAAGTTGGAGGTTCCTTATTGTACATT
TTAATTAACTTCGGTCCAACCAGTCCACTAAAATTTCTAGTGGACACAGGTGC

CACTTCTTCCTTCATTAATCCAGAACTTATTGAGGAACATAACCAACAAACCC
TAAATACACCAATCTTAATCACAACAGCACTAGGCTCTCATAAAGTAAACAAG
ATAGCCAAAATAAAATTATTTACTAAACACAACTGTAACGATACCTTTCCGTTA
CTTCTCTTCAAATTTCATAATTATTTTGACGGTCTTATAGGCATGAACACGTTG
AATAAAATTTTAGGAGTAATAGATATACCAAGTAAAATCTTATCAACACCCAA
ATTCAATTTTGAATTACTGCAGCAAGTAAAACAAAAAGCTGAATTATTTAACA
TAGAAGAAGCATCCAAGATATTGGTAATGCTACCCGTCACAATTAAGGAGGG
TACATTTTTATTTGAAGACCAAAAGGTTTCAGGAAATTTGTTTATTACCGGAG
GCCTGTACACGGCCCACAACTATTACAGTTGTATGGAGGTGGTAAACTATTCC
AAGAAGCAAGAAAAATTATACCTAGAAACACCAATAGAGGTAAATCACTTTA
ACACGGACGAATTCTATATAGTAAATGATATTAAAGACTATGGTACGATAAACT
CCGAAAAGGAAAAAGAAAAGTTCAGTACCCTAAGATTAGAACTTTTAAATTC
AGAAGAAACAACGGCGTTCAAAAAATTATGCAAACAATTTCCTAATATATTCT
ACAAAGAAGGAGACAAATTAACATTCACTAATAGAGTCAAACATAGCATTAA
AACCACTGACGAAATACCAGTACACAAAAGACCTTTCAGATACAGTCCTGCA
GAAAAAACAGAAATAACCGACCAAATTAACAAACTCCTAGAGCAGGACATA
ATTAGGCACAGCCATTCACCTTGGAGTGCTCCAGTTTTTCTGGTCCCAAAGA
AATTGGACGCTTCAAATAAGAAGAAATGGAGACTGGTTGTAGATTTCAGACA
ATTAAATGACAAGACGATCAAAGATAGATACCCAATGCCCAACATCAATGAA
ATTCTAGACAAACTAGGGAGAGCTCAGTATTTTTCGGCCCTAGATCTAGCAAG
TGGTTACCACCAAATTGAAGTGGAGCCTAAAGATAGATCGAAAACAGCCTTT
TCCGCAGTAGGTGGTCATTTCGAATTCATCCGAATGCCATTCGGCTTATCTAAT
GCACCCGCAACCTTCCAAAGGGTGATGGATAATGTCTTGGCAGAATTCAATG
GCAAATTTTGTCTGATTTATCTCGACGACATAATAGTATTCTCGACGTCCTTAC
AAGAACATATCAACCACCTAAGCTCGATCTTCAAAAAACTCACTCTGGCAAA
TTTAAAACTTCAGCCAGATAAATCGGAATTCCTCAAGAAGGAATTAGAATATC
TTGGCCACATAGTGACCGAAAAAGGCGTTAAGCCGAATCCAAAGAAAATTG
AAACTATTAAAGCCTTCCCTATGCCTAAAACCAGAAAAGAAATTAAGTCATTC
CTGGGGTTGCTGGGGTACTATAGAAGATTTATCAGAGATTTTGCCAAAATCAC
TAAACCTCTAACTCAGCAACTTAAAGGAAAGTCAGACGTGACCATCGACGAT
AACTATATTAAAACATTTGAATTTTGTAAAACACTCTTATGTAATGACCCCATA
CTACAGTATCCCGACTTCACTAAATCGTTTATTTTAACAACAGATGCTAGTAAT
GTAGCAATAGGAGCAGTGTTGTCCCAAGGAACTGTAGGAAATGATAGACCTA
TAGCATATGCAAGTAGGACACTATCGGAGTCCGAAACGCATTATGCCACCATA
AAAAAAGAATTACTGGCAATAGTGTGGGCAGTGAAATATTTTCGACCATACTT
ATTTGGAAACAAATTTCTACTGGTCACTGATCACAAGCCTTTAGTTTGGTTAA
AAAACCTAAAAGAGCCTAATTCCATGTTAGTGAGATGGAAACTCCAACTCTT
AGAATATGACTGTGAAGTCATTTATAAAAAAGGTTCTCAAAACGTGGTGGCA
GATGCATTAAGCAGAATTGTTGTCGAACTCAACACTAATGAAGCCAGACCAA
TAACCTCAGATACAGAAATTTTAACCTCTAACAGACCAATAAATGAATTTGCA
ATTCAGATCATATTAAAGATTGCAGATTGTGCAAACGACTTATTGGAAACACC
TTTTAAAAATAAACTCAGACGAATTATTTCCAGACCTCTGTTCGACGATCAGA
CAATGATAGACGTATTAAAAACGGCACTAAGATCAAATAAGACGCATGCAATT

TATACCACTGATACAATTTTCGAATCAGTGCAAAAAATTTATGCAGCATTTTTC
GCTCCAAGTAAATCATATAAAATTATAAGGTGTACAACATATTTAGATGAACTC
CGAACACCAGAAGAACAGGTCGAATACATATCTGACTACCATATCAAGAACA
ACCATAGGGGCATTGACGAAACCGTTAGTCATATTAAACGACAGATTTATTTC
CCCTGTTTAAAAGAGAGGGTTTCCCAACTGATAAACAAATGCGACATTTGTC
AAACCCTCAAATATGATAGACAACCTCAGAAACCAATATTTCAGCTAACAGA
AACACCTAATAAACCACTAGACATTGTACACATAGATCTATACTCAATTAACA
ATAAAACCATTTTAACAATAATAGACAAATTTTCAAAATTTGCAGAAGGTTAC
ACTATTCCATCTCGAGATTCCATTAACATCACTAAACATATGATGTTCTTCTTC
AAGACCCATGGTATTCCCAAAACAATAGTTTGCGATCAAGGCCCCGAATTTG
CAGGAATTATTTTCAAAGAATTGTGTAATCAATACAATATAACTTTACATGTAA
CATCTTTTCAACAATCAAGTAGTAATGCCCCAGTTGAAAGATTACATTCATCG
TTGACGGAAATTTATAGGATAATTTTCGAAAAGAAAAAGGCATTAAAACTCA
ACCTAGACCATGATTGTATCCTAACAGAAACATTTATAACGTACAACAACGCC
ATACACTCGTCTACAAAATTGACTCCATATGAAGTTTTTACAGGACGAACCCA
TATTTTCGAACAAAATTATAAGGCAGTCAGTCAACAAGACTACATGAGGGAA
CTGGAAAGTTATAAGAACAACTTATATGTAGAAGTAAAGGAAGAATTTGAAA
GWCAAAAACTCAACAAAATTCTAAAACTAAATGAGGACAGAGTATTACCAAT
AGCGGTACAGGAAGGAGATACTGTTTTTAGAAAGGAAAATAGGAGAAATAA
GCTAACACCCAGATTTACCCAACACAAAGTAGCTGATGATAACGGGGTAACC
TTTACAACGAACAAAAGACAGAAAATACATAAATCAAAAATTAAAAAAAGA
ATCAGACAAAATAGTAGATAAATATATAATAATATCATTTCAGGTTATACATGAC
AGCAGCTCAACTGCTCCATGTTCAACATGTTAAGGAAAATACCCCCCTAGCA
GAGATCAAACTGGGAGAAGCAAGGACCATTAAAACATATAGTTCAGTGGCCC
ATGTAATAGAACTAACGGAGTTAAAAAATAATTTGAATAAATTAGAATCAAGT
CTACAAAATTTACAAGATGAAGACTCTAATGATTCAATAACACCCATACAAAG
AAAGATAGACCAAGCAAGAGAAAAGATCTATTCCTTGACCCCAAACCGAAG
AGAAAAGAGAGGCTTAATAGACGGTCTAGGTAATGTTATAAAGAAAGTAACT
GGGAACATGGACGCAGAGGACGCAGCTAAAATCGAGACAAAATTTAAGCAA
GTATTTGAAAATGAAGAAAACATTAACAAGAACCTCGGGCAACAAATTTCTT
ATAACAACGACATAATCGTAAATTTTAATAATATGTCTGATCATTTTTATAAAGA
ACAGGAGAAAATTGAGAGGTACATTAATAACTTTAAAAATGGCATTTCTAAG
GAATTAATAGTAGAAGATAAGAAAATTAGAATTTTACAAATATTGAATATAATA
AATAATAATATAGATATTCTCCAAAACCACATACATGATATAGCTGAAAGTATA
CTATTGGCTAAATTCAAAATAATTCCCAATTTTATTTTACCTATACAAGAATTG
AAAGATATTGCACAGATTTTTAAACTTCAAAATTTTTCTATTAATTCAGAAGA
GCATATTTACAGACTACTCAACATAGAGATAGAATCCAAAAAAACAAAAATA
ATATTTCATATAAAGATTCCAATATTTGATAAGTACACTTACACACTTTCTCACC
TAATTAAATTACCTATCAATGGCACGAATTTTCTCACTCTACCCAAATATATATT
GTACGATGATAAATTTAATTATATGTTAAGTTTTCAAGAAAAATGTTCAAGAAT
ACATGGCACTTACATCTGCGACCCCGGAACTGCGGAAAGTAACATGGACAAC
AGGGAGTGCGTGAAACAAGTGCTGCAAGGAATGAACCCTGTTTGCGAGGCA
AAGATTATCAACACGGACGAGGCGGTAGTAGAACCGGAACAACAGTGGATT

GTCATTATCAGCAAGAATCAACTAAGGGCTGAACCAAGTTGTGCTGAACCGT
TCACTATTGAGGGAACGACGATTATCAATCACATAAACTGTTCCATATTTATAG
ACGGTACCAAATACGATGACAGCGAATTGTTCTACCAAGAAAGGATTAAATT
ATCACTACCAGTAACGAAGAACATATCTATAAATGCAACATCGGAGGATTTAA
GCCTAAGCAAAATAGTACTGCATCTTCAAGAGAATAAAGCTAAAATATTCCAA
GTCAAGGAATCTACGATTAACCTTCGATTCATAACCTACGCATCAAGTATCATC
CTGGTCATATTTATACTGTATGCTTTAGCAGTAAGGAGAAACACTACTTACCTG
CCCAACCCTGTTACCTCAGTCAACTACGTTCCGTCTATTCCTTCGTTATGGCC
GTCACTTTATTCAAGGGGGGGAGG

>GYPSY4_LTRGypsy#LTR/GYPSY4_LTRGypsy

AGTTAACACGCGCAGGGCCAAAATCGCTAACTCCACGATAAGACCGGACGC
GGCCGCAGTAACGTCAGCGCGACTGCAACTAAGTCGCGAAATATGACTCCTG
CATTCCAACATTCTACTGCCCGGAGCGTGTGAAGCGCAATGTCAGCRTTCTGC
CGTGAGCGCTGCTTCAGAAGACGGGCTACTTCATATTAAGCTTAAGTTTTCTG
TCTTTAGTTTTAARAACTCATCWGAACGCGCATAGTCGCAKAATAAAYCACA
ATAACTAAAATTGTTGTCTGTTAATT

>GYPSY4_IGypsy#LTR/GYPSY4_IGypsy

GGCGCCCACGGCACTCTTATAGAACTCACAATATTTGTTCACCAGCCAAAGCT
GTGACAAATAAACGAAAAAGTGCGAAGTGAACTGTGACAAACAAATTACAA
GTGCAAAGCTAATTATAGAACTCAGAATATTTGTTCACCAGCCAAAGCTGTGA
CAAATAAACGAAAAAGTGCAAAGTGAAGTATTTTTTCCGACCAAGAACACA
GCAAACAAAGCGAAAACAAGAATAAAAATAAAAAAGCTAAACAAACGGTTA
AAACAACATACAAATTAAAAAGAAAAAAACGGTTAAAACAACATACAAGTT
AAAAAAATAGAACCCCAACACGAAGTGTCGAGAGAGTAAAGAAAAAATCA
ACAAATCAACAAGTGCACGCATCGGCAGTTTAAGCAGTTACCCAGAAATGAA
ATACATGTAAGTGATTTGTTCATTGTGCCATTATAAATGCAGAAAAGATTTAGC
GACAGTAGTTTAGAAGACTATATCCCCTCTTACCGAGAATACAAAAACCGCA
CAATGACGCAACAAGAATCAGTACAGATTGACCTTCACATACGAAATTGTCT
GGCAGAGCAAGCACAAATATTTGACCAAAAAATAGCAACTTTAACAGAACA
ACTGAACGCTTTAAAGGCTACTGCGCCAGAGGTGACAGCTTATAAGCCTATA
GAAATCATACCAGAAGTTAGATGTGAAGAGCCCCTAGACATAGTTAAATCTAT
TCCTGAGTTCGACGGCAAACAAGAACATTATATCTCTTGGCGCCAAGCGGCC
AACGCCGCATATAAAGTCTTCGAAGCTTATGACGGAAGTTCTAGGCATTATCA
GGCTGTTGCCATAATACGCAACAAGGTTAGGGGATCTGCAGATGCGATCTTAG
CATCATTTAATACAGTTTTAAATTTTCACGCGATAATTGCTAGACTGGATTTCA
CATATTCAGACAAAACTCCGGTTCACGTAATTCAACAAGATTTAAGTACTTTA
AGGCAGGGTGACCAGCCCCTTTTGAAATATTACGATGAAATAGAAAGAAAGC
TGACGCTTTTAACTAACAAAACTCTCATGACCCACGACGCAGCTTCGGCAGC
TGTGTTAAATGACAAATATAGAAACGATGCACTTCACACATTTATCTCAGGCC
TGAAGAAATCTCTAAAATGGGCAGTTTTTCCAGCCCAACTTCGAGACTTACA
GACTGCCCTGGCATTAGCACAAGAGGCAGAATCAAGTAACGAGAGAAGCAT

TTTTGCCGCAAACTTTGCTCGGCACATAGAGGAAAAAGCGCAAAAATCTGGG
AATCAGAGGTCCCAAGGTTCGCGTCAAAACCCCCAGCAAGAGAGCGACGCT
CCCACTATCTTTAGGAAAAATCCCCATTACCAGAAAGGTAACGGTCAAAAGC
CGAACACTTCAGGTAGCGCTAGACAGAGATATTACCAGAAGCCACAAGAATC
AACTTCCGAACCCATGGAAGTAGACACGTCATCACGCTTTAGACAGCCCACT
CAAGCAACAAATTTTAAAGGTGGTCATTCAGCGAAAAGTCGCCAATCATTGA
ACCACATGCCCCAAGACCAAAAAACCGAATACGAAGAACAGGCAGAATCCG
AGGCAAATGCGGCTGAAGACGATTTGTCTGAGGTCGAAAGCTGTAATTTTTT
AGGGGTCACTCCCTGCTTCCGTACATCACACGTACAGTAGCGGGGCAGACCA
TAAAACTTCTTATCGATACAGGGACTTCGAAAAATTACATTAAGCCACTTCTA
GGGCTGCCACACTTTGTGCCAGTCGACAAGCCATTCCAGGTAAAATCACTAC
ATGGTCATACAAGAATTGAACGGAAGTGCCAAATTCAACTTTTTAAAACTAA
AACATATTTTTTTATTTTAGAAAATCTAAGTGATTTTCACGGCATTATTGGCCTA
GACTTACTAAAAAAGATTAACGCCAATGTTGATTTCGAAAAAAATTGTATCAC
TTATGATCGCGGTTCAGAGCCAATCAAGTTTACAAAATGCCAGAACGTAAAC
TTCATTAAAATAGACGATGGCGATGTTCCTGAAGTCATTAAAGATGACTTTAA
CAAAATGATTAATAAAAGAACAGGGTCATTTGCAGATGTTAATGAGTCCCTGC
CCTACAATATCAATACAGTTGCCACAATACGAACTGACGGGGAGCCAGTATAT
TCTAAACTGTACCCTTACCCAATGGGCGTATCCGAGTTTGTCAACGCAGAGGT
TAAACAACTTCTAGCGAATGGCATAATAAGACCGTCCAAATCACCTTACAAC
AACCCGATCTGGGTTGTCGATAAAAAGGGGGTAGACCAATCGGGCCACAAG
CTAAAGCGTATGGTCATCGATTTTCGTAAACTTAACCAAAAAACGACTGATGA
TAAGTACCCCATCCCAAGTATTTCAACCATATTATCAAATATGGGCGAGGCAC
AGTACTTTACTACTCTTGACCTCAAATCGGGATTTCATCAAATCGAATTAGCG
GAAAGGGACCGGGAAAAAACCGCTTTCTCCGTAAACAACGGAAAATATGAA
TTCTGTAGACTCCCTTTTGGTTTGAAAAATGCGCCAAGCATATTTCAAAGAGC
CATTGATGATGTCTTAAGAGAGCAGATAGGGAAAACCTGTTATGTCTACGTCG
ACGACGTAATTATTTCTCTAACAACAAAGAAGACCACGTTAAACACATCGAT
TGGGTGTTAAACAGTCTTCAGACGGCTGGCATGAGGGTGTCTCAAGAAAAAT
CCAAGTTCTTCAAAAAGAGCGTGGAATATCTGGGATTCACGGTATCCCGCGG
CGGGATTCAAACTTCGCCAAGTAAGGTTCAGGCCATAAAAGACTTCAAACCG
CCAAAAACCTTGTTTAGTCTCAGATCATTTTTGGGGCTAGCAAGCTATTATAG
GTGCTTTAGAAAAGGCTTTGCCAACATCGCACGACCCCTAACGGACGTCTTA
AAAGGCGAAAATGGTAAAATAAGTGCCAACAATTCAAGGAAAGTAAACCTT
GAATTAACACAAGATCAACTAAAGGCTTTTAATAGACTAAAAGACGTATTAGC
CTCTGAGGACGTTATTTTAGCATACCCAAACTTTAAAAAACCATTCGATTTAA
CTACGGATGCCTCAGGGCACGGTCTTGGAGCTGTGTTGTCTCAGGACGGACG
TCCAATTACCTTGATTTCTCGAACATTACGAGACAACGAAGTAAACTTTGCAA
CAAATGAGAGGGAACTCTTGGCCATTGTTTGGGCCCTAAAAAATCTTCGAAA
TTATTTATATGGCGTAAAAAATTTAAATATCTTCACGGACCATCAACCGTTGAC
TTTTGCCGTGTCGGATAGGAACCCAAATGCGAAGATAAAACGCTGGAAAGCT
TTTATTGACGAGCACAACGCAAATATTTTTTACAAGCCTGGCAAAGAGAATTT
CGTGGCAGACGCCCTATCTCGCCAGAATGTAAATGTACTCGAAGATTGCCCA

AACTCTGATATTGCCACAATACATAGCGAAGAATCTCTTACCTATACTATCGAA
ACGACCGAAAAGCCTGTAAATTGCTTTAGGAACCAGATCGTAATTGAAGAAT
CTAACGCCCCATCAGTTAGATCTACTATTTTGTTCAGGGAAAAAACCAGGCAC
GTTATAAGATTTGTTGACCGTAACACACTTTTGCAAACAGTACAAGACGTCGT
AAATGCCAAGGTAGTTAACGCAATACATTGCGACCTACCCATTTTGGCATTCA
TACAACACAGTCTAGTAAAAGCGTTTCCGTCAACCACATTCCGACACTCCAA
AAATATCGTGATAGACATTGTCGATAAAACCGAACAGAGAGAAATAATTATTG
CAGAGCATAACCGAGCGCACCGAGCAGCGCAGGAAAATGTGAAGCAAATTC
TCCGGGACTACTTTTTCCCAAAGATGAATTCGCTGGCAACAGAGATTGTCGT
AAACTGTAAGGTTTGTTCAATGGGTAAATATAATCGACATCCGGTCAAGCAAG
CGATGGGTGAGACCCCAATCCCATCATATGTTGGGGAAATATTGCATGTTGAC
ATTTTTAGTACCGACAAAACATTTTTTTTAACATGCGTCGATAAACTCTCAAA
GTTTGCGGTCGTCCAATATATTACTTCACGCGCAATCGTGGACGTAAAGGCAC
CGATTTTACAGCTGGTAAACCTGTTTCCCAAAATAAGGATCATTTATTGCGAC
AACGAAAAATCTTTAAATTCCGAAACAATAAAAAATATACTAAACAATTTTGA
TATTCAGATTTGTAATGCCCCACCACTCCACAGCACCTCTAATGGTCAAGTGG
AAAGATTCCACAGTACCCTTACAGAAATAGCCCGTTGTCTAAAAATAGATAAA
CACCTTAACGAAACCAAGGACATAATCCTTTTGGCGACCATTGAGTATAATAG
GTCAATTCACTCAGTAACAAACAGAAAACCGACAGAATTAATTTGCTCCGCC
CCTTCGGATTTATTAACAGAAACTAGGGATAAAATTGTACTTGCGCAAGAAAA
ACAACTTGGGTACATGAACCGAGATAGAATCCATAAAAAGTATAAAGTAGGC
GAAAAAGTATGGCTAAAATCAAACAAACGATTGGGTAATAAACTGTCACCAC
TGTGCACGGAAGAAGCCATTGATGCTGACTTAGGTACGACGGTTCTAATGAA
AGGAAGGGGGTGGTCCATAAGGACAACCTTAAGTAAACCCAAGAAAAAAAA
ATTAAAAACAAATATAAATAAATAAAAAATTTCATCGCTTATAGTACTTTTAAT
TTTAATATTTTTATTACAGGTTAGGATATTTGTGTGTGCTGGCTTCCGCAATCA
CCTTGACAATAACGACTATGAAACTTAATGACTATTCACACGCAGACTACATA
CCTATAATAGACGGTGACTTAACGATATGGGACAGATACGGATATCTCGGACA
CACTTCTAACATAACTTCTTATGAAACTTATGTTGAAGATACCAGACGGCAAC
TGAACTACTTTGGAAAAGACCATATGCGAAATTTGATAACTACTGACTTGGGG
CACATAGAATCACTAATAGCTACGATTAGGGTACACCATAGACATGCTCGCAG
TATAAATCTACTAGGTACAGCACTCAAGGTAATAGCCGGAACCCCAGACTTCG
ACGACTGGGAACAGATTAAATTTAAACAAGGGCAATTAATAGATTCAGCAAA
TAGACAGATCGAAATAAATACGAAACTTCAATCTCGACTAAACGAAATTACA
AGGTCAATGAATGCAATAAGTAAAACAGACAACGCGGACTCAGAACACCTAT
TTGAAAGTATACTTGCTAAAAATAGAATAATTATTACAAACCTAGAAGACTTG
ATACTGTCGGTAACTCTAGCTAAAATAAATTTAATAAGCCCACTTATTTTGGAT
AGTGTTGACATTCATGAGCTGGCCAATGAACTTCTCACAAATACGAGCTTAG
CAGATATTTTAAGAGTATCTAGCGTTAAAGCCTTTCAGAACAACGATTTATTGT
ATTTCTTAATAAAATATCCTAAACCAGAAACAGTTTGTAAAAAAATTAACGTT
TTCCCAGTTCAACATAATAAGATAATATTAGACTTTGAGAATAATAATGTAGTA
GCCGAATGCGGTGCACGAATCTACGCCGTCAAGGAATGTAACGCAGCCATGA
GTACCACCTTCTGCAAGAGATTGCAGAACCCGACTTGCGCTCAACAATTGGT

GTCTGGAACCGTCGCACAGTGCACCACTCTCCTTGGCCATCTGGATCCGGTG
ACACTTGTTGAAGAAGGTGTCCTGATTATCAACGACGCAACCTTCAGGGTCG
AAGATAGCCTAGGCAGCAGTAAGATGATCTCGGGAACCTACTTAGCCACATAT
GACGATCGTATATCTCTAAACGGCACCCACTACGAAAACCACCAGGGCGTCC
TAAAGAAGAAACCCGCTGCGGCAGTGGCCTCTCAGATCAATGTGACTAGCCA
TCGAGATCAACTAAGCTTGTCGTTCTTGCACGAGCTGTCGCTGCAGAACCTC
CAACATATTGGTAGTCTGAAATCTGACATCATTTCGAGACCCATTTTAAGCAG
CGGCGTCACCATCGCCGTGGTCCTAATTGTTTATGGGATCATCCAAAGCATCC
GTTACTGTCGAGTCAAAAGACGACACCCCGACTCCAGCATCGAGATGACGAT
ACCTTCCCGAAAAGCCGAGGACGACTTTAACCTAACGCGGGGAGG

>GYPSY3_LTRGypsy#LTR/GYPSY3_LTRGypsy

AGTTAACTAAGTTAACCGGACTGATCGTCCGCTGACCAGCATTTGGCCGGAA
GCTCGAGCATAGCCGGCAAAGCTCTGCACTTTGGCAGAGGCCGCTATGAAGC
TCTTCTCTTTGTAAGCTTTAAGCAGTTCGTTTTGGACACTCATAAAATAAAGA
GCCATCGCTCATTAACCAACAACTCCGGCATTCTGTCGTTAATTTTATATTTGA
TCATAGAAATCTGAGTCCCTAGGCCAATTGCATTAAGGAAGACAGTCATCTTC
CCAACGTAATCATCGTAATTCTCCAACTGGATTTATCACCCTCTTCCCCTGTAA
GAGAGACACCATCAGCCAACCGGCAAATCATGGGAGACTGCAGCGAGGGAC
CCCCTGCAGCACCCCCACATTTAATAATT

>GYPSY3_IGypsy#LTR/GYPSY3_IGypsy

GGCGCCCCACTAAGGAAACACACAACAACACACACAAACACACAGATTATG
TAAGTGAGCGCCTATCTCCAAACCCCAACTGCATTTAAACAAAGTTTATAACA
TTTAAAATATTTCTGCGATAAATAAATAATCTGTATTTCCATGACAATCCTAATC
TTTGTATGTGTTGTGAATTTAAATTATATTAATGTATGAAATTTAAACATGGAAT
TTTAAATTTTGATTGACTGTGCGGATTAAATTATTCTTTGGCACTGTCGCAGAG
ATTTATTGATTTACAATTTACATCATGTGCATTATTTCGTTCCACTTCTGCATAT
GTCTGCACACTGTCGTTCCACTTGAACTTGAATTCTCTCCCCACAGCCGTCTC
GGGCCCTCCTATCGCTGCCCCGACACCCGATACAGCAGCGCATGCTTTGCTGT
AGCAATTTTTTGCCTTTTATATATCTCTTTGTCTGTGCTGCTACGATTTGCTTGT
CATTTATATATTTGTTTGGCTTAGCGGATGCGAGCTCCCCGCGGCTCCGCTCAA
CGAGCCCTCGGCCAATGTTATTAAACGCCGACTCAGCCCCCGCGTCCCCTCG
CCGAACTAATACTTCCCTTGGGCAATACGACTTGAGTATTTATATACTCAGCTT
CTCATGAGCAACTCATTCCGACAATATAGGAATTCTAAACCATTCGCTAGCGA
CTCAGAGTCAGAAGGCGACGATTCTACAGAAAACTCTGTACATAAGAACACC
GCAGTTAACGCATTTGTTGCATATAAAATGTCGCTCGAAACGGAACAAATTAA
AGTCCTCATAAGGGCGTTACAAGAGCAAGCCCTAGAAGGTCAACGCAGGGA
GGCTGACCTGCGTAATACAGTTCAGGAACTTGCCAGCCAGCTCGCGGCTATG
CAGGTTGCCCCTGTGCGGGCAGAAACTCCACAAATTAAGGTTTACCAACCGG
TAGATATCACCGGAGAAGTCCGTTGCGAAGAAACATTGGACGCTGTAAAACG
CCTCCCAGATTTTATGGGCACACAGGAGACATACGTCTCCTGGCGGCAGGCG
GCGAAGGCCGCTTACCACATGTTCAGAAACTATGAAAATAGTTCGCGACACT

ATCAGGCTGTAGTTATCATCAGGAGCAAAGTAAAAGGCCCTGCTGATGTAGT
TCTGTCATCCTTCGGAACTGTGCTAAATTTCGATGCAATTATAAATCGCCTCGA
TTTTACGTACAGCGACAAGCGCCCGATACACGTTATCGAACAAGAGTTAGGC
ACTCTCAGACAGGGAAGCATGACGCTCCTTCAGTATTATGACGAGGTTGAGA
AAAAACTCACCTTGCTCACCAATAAGGCAACCATGTCTTATGAAGCCTCGGC
AGCAAAGGTGCTGTGTGAAAAGTTCCGAGATGATGCTTTGCGAGTTTTCGTC
TCGGGACTCAGGCGCAACCTCACGGACGTGCTGTTCGCGGCAAAACCGAAG
GACATGCCCTCAGCACTCGCCTTGGCACAGGAGGTGGAATTTAATCACGAGC
GATACACCTTCGCAACTTCGTTTGCAAGAAGCCAAGAAGATAGGGACCGCAC
GCAATATCCCAAAGTGCAGGAGCGCCAGCAGGCCCCTCCACAAGCCAACCC
GCAGGGAAGTGCCGGAAAGAACCTAGGCAGCACAGAGCACAAGTGCACTC
TGCTCCACGTAGCGACCGGATGGCTCGAGAGAACACACCGGAACCTATGGA
AGTTGACCCTTCATTGTCCAGGATGCAACCATCTCACGCCCCGGCTTACCCGA
AATCGAAGCCGGCCACATCTGGCCGCTCGATCCCGCCTAAAAGGCAAAGGGT
CAACCATGTTGCCCAGGCCTCAGATTACACGGCTTATGCCACCGCAGCCTCC
AGTGCAGCGGTTAAAGTCGACGATGATGCCATCCTAGAGTATGACTCGGATG
CCATTAATTTTTAGGGGTAAGTCCCTGCTACCCGTCATCAGACGAAGAGTAG
CGGGGATTGACATGAAACTGCTAATTGCATGGCCAAAAATTTTATCTGACCTT
TTATGGGGTTGAAAGGCGTCCGCCCGGTGGAGTCCCCATTCGCAGTCCATTC
GATTCATGGCGTGACTACAATCGCAAAAAAATGTTTTATCTCTATTTTTAACCT
TAAAGCTACATTTTCCTTACCACCAGATTTGACCTCCTTCGACGCGATCATTG
GCCTAGACCTATTAAAACAGGCCGGCGCGTCGCTTTGCCGGCCAGCTCAAAT
GGGGCTCCGAAGCAGAGCAAATTGACTTTCACACTGACTTCGTCAACGGCG
AAATTCAAGAGCTGCTGAAAAAGGGCATAATCCAAAAGTCGAAGTCCCCCTA
CAATAACCCAATATGGGTCGTAGACAAAAAGGGCACCGACGATGCTGGCAAC
AAAAAAATGCGTTTGGTACTGGACTTTCGCAAACTGAACTTGAGGACTGTAC
CAGACAGATACCCCATGCCAAATATCTCAATGATATTGGGGAATCTCGGCAAG
GCCAGGTACTTCACGACACTCGATCTGAAGTCTGGCTATCATCAAATCACGCT
CGCTGAACGCGACCGTGAAAAGACATCATTCTCAGTAAACGGAGGGAAGTA
TGAGTTCCGAAGATTGTCATTCGGACTCAGGAATGCTGCAAGCATCTTCCAG
AGGACAATTGACGATATTCTGCGAGAGCAGATCGGCAAGTTCTGCTACGTTT
ACGTTGATGACGTCATCATCTTTTCAGAAGATGAAAATGCGCATGTCAAGCA
CGTAGATTTGGTTCTGAAGAGCCTGTACGATGCTAACATAAGAGTATCTGCAG
AAAAGTCACGCTCCTTTAAGAAAAGCGTGAGCTTCCTAGGGTTCATCGTCAC
CAACAATGGCGCGGCGACTGACCCAGAAAAGGTTAAGGCCATAAAGGAATT
TCCAGAACCCAAAAACGTATTTGAGGTGAGGTCATTCTTGGGCTTAGCCAGC
TATTATCGATGCTTCATCAAAGATTTCGCATCAATACCAAGGCCCATTTCGGAC
ATACTGAAGGGCGAGAACGGAAGTGTTAGCCGACACAGGTCCAGGGGTATC
AAGGTAGAATTTTCCGAAGCGCAGCAACGTGCCTTCGAGAAACTTCGCAAC
ATTTTGGCGTCTGAGGACGTCACCCTTAGATACCCTGATTACAAAAAAGCGTT
TGATCTAACGACAGACGCTTCGGCCTACGGCATTGGCGCAGTGCTGTCCCAA
GAGGGACGCCCCATTACAATGATCTCTAGGACATTGTGTGACAGAGAGGTTA
ACTACGCTACCAATGAAAGGGAGCTATTAGCCATAGTCTGGGCGCTAGTCAA

GTTGCGACACTTTATACGGTAAAAGAAATAAACATCTTTACCGACCACCAAC
CTTTGACGTTTGCGGTATCGGAATCCAATCCGAACGCCAAGATCAAGAGGTG
GAAGGCACGCATCGATGAGTCCGGCGCAAGAATGTTTTACAAGCCCGGAAA
AAATAATCTCGTTGCAGATGCCCTTTCGAGGCAACAACTCCACGTTGTTAAA
AACCAGGAACCTGAGTCGTGCGCGTCCACGGTTCACAGTGAGCTTTCGCTC
ACATACACAATTGAGTCTACGGACAAACCCGTAAATTGCTTCCAGAACCAAA
TAAATTTAGAAGAGGCGCGCTCCCCTTGGAAACGCACTTTTATACTATTTGGA
AATAAGAAGCGTCACTTGATTAACTTCTCGTGCAAACAGACTTTGCTAGAGG
AACTAGCCAACACCATTATCCCTAATGGTGTGAATGCCTTCCACTGTGATCTT
CACACGTTGGCAAAAATCCAGAACGAGGTGGTCCGACAATTTCCGGCCACA
GAAGCTCGGTGAAACACCAATCCCGGGCCACGTAGGAGAAATATTGCACATC
GATATTTTCTCGACGGATAGGAAATACTTCCTCACCTGTGTGGACAAATTTTC
GAAATTCGCCATGGTGCAGCCGATCCCGTCTAGAACCATTGAGGATCTAAAG
CCACCACTGCTTCAACTTATGAATGTTTTCCCCAAAGCCAAAGCCATCTACTG
CGACATTGAACCATCACTGAAATCGCACACAATAGTGGCCATGCTGGAAAAC
CATTTCGGCGTCAGCATCTCGAATGCACCGCCCCTCCATAGCGTCTCCAACGG
ACAGGTGGAACGCTTCCACAGCACGTTGGTCGAGCTCGCTAGGTGCCTAAA
AATCGATAAAGGCATAAATGACACGGTGGAGTTGATCTTGCTGGCCACAGCC
AGATATAACAAGTCCATCCACTCCGTCATTAACAAAAAACCGGCCGAAGTCA
TGCGGGCAGATCCGGACGATCCACAAAGTGATGTCCAGGAAAAAATCAAAA
ATGCCCAAAACGTGACACGAAATCGAGAAAACGCCTCTCGGCGGAACAGAG
TCTTCCAGGTCGGCGATAAAGTCCTAGTAAAGTCAAACAGACGACTAGGCGA
GGAGAGAACTATCGAGGCAGAATTGGGGACCACAGTCCCAATTAAAGGGAG
GGTGGTCCACAAAGACAACCTCAGGTGACCCAAACAGAGCCCAGTTGCGTT
TCCCCCGGATTTTCTTTTCATTCTTTTCATTTATAGCCACTTGGCATAAGTTTTT
TATTTGTTTTTCATTCGTAGCCAATTGGCATAAGTTTTCATTATTTTTCGCTTCC
CATAGCCACTTCGCGTAAGCCTTTCTTATATCCTTCATTACTCATAGCCACTTG
GCTTAATTTTTTAACGTTTAATTTACTTCCTCTTATTCGTCTATTGGTGGTGGGC
AACTCCATTCCGAGTATTTATTAATCAACCACGCATATTACAGGTCGTTTCCAA
CGCTTCTACTTTGTCTCCTGGCGGTGGCATCGGCCCACGTCACAGATTACACC
CACGCAAATTACATCCCCGTCATTGATGGCCAAGTCTTAGTAGAATAAGAATA
TGCCTACGTCAGACACTCTGCTAACCTCTCTGAGTATAGGCGAGTGATTGACG
AAACCAACGGTATGGTTGATATGTTCCCCGAGTCCCATATGAAGAAGCTCCTG
AGCATTGACATCGCTCACCTTCGTGACATGCTCGACTCGTTGAGCGTCCATCA
CAGAGTGGCTAGGAGCCTAGACTTCTTGGGAACTGCGCTAAAGGTTGTCGCA
GGGACACCTGACGCAGAAGACTTTGAGAATATAAAGTTCACCGAAGCAAGA
CTAATTGATGCACACAAGAGCCAAATCGAAATTAACACCAAAACACAAGTTC
GAATCAACGAACTTACCGACACCATTAATCAACTTTTAAAGATTTCAAAAGAT
AAGCAAATCGACACAGGCCACCTTTACGAAATGCTATTAACACGCAATAGAA
TCATTGTCATGGAACATAGAACCTTTTGCTTACAATCACACTCGCAAAAATTA
ACGTAGTTAGTCCAGTCTTTCTGGACCATACAGACTTGGAGAATGTTTGGGG
CGAGGAGCACACCAACACCCCCATAAGGGAAATTTTGTCCGTTGCGTCCGTA
AAGGTTTTACAATCCCTTAACGTTTTACATTTTATTATAAAATTCCCCAAGATT

GTTATGGCCTGTAATAAATTCACCGTCTTCCCAGTGGCTCACCATAACACGGT
ATTGAGGTTAGAAGACAACATGGTGGCAGAATGCAATGGAGAAATCCGTACC
GTGAAGAACTGCTTCAGATCACTCGGGGCGACATTTTGCCAGTTATCTTCGGT
GAGCTCGTGTGCCCAAGAACTCCACGCTGGAGGCATGGCACATTGCAACAC
GCAGCAGAGCGACTTACATCCTATCACATACGTCGATGAAGGAAATATTATCA
TCAATAATAATAAGTGACAGGCACTCTCCTGTCCTTAATATCTCGATGAAACA
CGAGATCCTGAGCCTCCCATACCTTCACCGCTTGAGTGAAAGGAATTTGGAG
CAGATCAGGAAATTCGAACAAGACGTCGACGGATACCGACTAGGTCAGATAG
CGCTAATTGCAGGAGCAATTTTCTGCGCTCTCATTTGCATCGGTCTAACCTGC
CAACGAGCCATTAGGGTCAAGAAGTCCACAGCCCAACTTAAGGAAGTACTCT
CCAAAATAGGGTCGGCCGAGGACGGCCTCAATCTTGAGGGGGGAGT

>GYPSY2_LTRGypsy#LTR/GYPSY2_LTRGypsy

AGTTAACCAAAGCTAACGTCGCTCCCACGGGCGTATGAAATACGACAACATC
GGCATACTGCAACGTAAGCATTATGCCGGATGTCGATACGTAGCCGGCAGAC
ACTGCAGATTTGCGTGGCCGCTACGAATATGAGATCGCAACAATGTGTTTCGT
TTTCATGCTAGCTCCAAAGTTTGAATTCTTGTATTTTGTATTCAGTTCTGATTTT
GTCTCCGACGCTACAGCTCCGCTTAGCTCGGTATAATAAAACATTAAAAGTAA
CTCCTAATCTTTTATTGTCACGGCTCCGCCTGACACTTGGTTAGTAAAAGCCG
CGCGCGTTAACCGCCGGCCAACCCTTTTTCTAATT

>GYPSY2_IGypsy#LTR/GYPSY2_IGypsy

GGCGCCCAACCAGTGGTATTTGACAGTGCGTTAGTCATTACCCACGAACAAA
AAAAAAGAAAAAAACAAACTTAAAAAAAAAAAAAAAAAAATCTAACTACA
AGTGCAAAACAAACAAAATAAAAACCTAACTACAAGTTAATCAGACTCAGC
CGCATTAACAGGGCTGTAGCAACAAAACAAGTGCTTGTGACCACTGTTGTTT
GTAACAGAACTATTTAACAAGTGGCTGCTGCTGACCTACGTCAACACAGGCA
GCACAGCTACGTCATTAACCTCGCCTCTTGAGTGCAGTTGGCTGAGGTGCGC
TATAGCGCAAACTTAATTTCACGTCACACCGAAGCCGCGGGTGCTCTTCGGG
CAGCGACTTATCGCCAGATTAGCGCGCAAGCTATCTGGACAGCGGAGACGGC
AGCGCGGGAGGCAGAGACAAGCAGTGTACAAAAAAACAAGAAACCAACTC
CAGTGTAGAACACAACACAACACCAAAATAAAAATGTAAGTGGGCACCTTTT
TTTAAAACTGCTCTTAAACCTGCGAAATAAAAATAAGCATATAAACACATTTT
TGCAAGTGTCTTCTTAAATGAAATTAAATAATAAATAGTTTTAACATACATGAA
ACTAGTAATGGATTAGGTTGACGGAAAACTATTGGCAGCAAAAATATAGTAA
ACATTTGTTGAATAATTACTAAAAGCGGCGCATCAAATGCATGCACGCACAAT
TTTTTTTTCCGTCTGCCTTCATCTTGTTCGTTTTGTTTGGTCGTTCAACGTAAT
ACGATCCATGGAATTTTGCAACTTTGTTGCCTCCGTCCTCAAGCGTTGTGTTC
GAGTTTTCGTTGTTTACATTAGAGGCATATGACTTCACCCTTCCTTTTCTATAG
AAACACTCGCCGAAAGTGTAGTGATTCTGACTCTGATTGCGACGACCATCCC
ATTCGCTGTTCAGTGCCAAGACGCACACCCTCACCCCCACCCTCACCACGTC
CTGCAGACAGCGTCATGGACCCAGACCAGTTGAAATTCGTAATACAGGCAGC
CGTCACCGCTGCCTTAGCTGAGCAAGCCGCCGCAAATAAGGTCTTGCTTGAC

AAAGTCAACTCAATGTCCCAGCAGCTGGCCGCAGCGCATATTACGCCACCAG
AGGTGCAAGCCTATGCACCCATAGAGATAAGAAATGACATCCGCTGCGACGA
GCCACTAGATGCCGTCAAATGCTTGCCCGAGTTTGCAGGCGCACATGAATCG
TATGTTTCATGGAGACAGGCGGCTCTCGCAGCCTATCGTATTTTTAGACCGTA
CGACGGCAGTTCACGCCATTATCAGGCGGTCATAATTATAAGAAACAAAATCA
GGGGGGCCGCGGACGTTGTTCTAGCCTCTTTCGGCACAGTTCTTAACTTTGA
CGCGATTATAAGTCGCCTCGACTTCACGTACAGCGACAAACGTCCAGTTCAC
GTAATCGAGCAAGAGCTGGCCACGCTGAGACAAGGCAACATGTCTCTGCTG
CAATACTATGATGAAGTCGGCAAAAAGCTTACATTGTTGACTAATAAAGTCAA
CATGTCATACGAGCCGGTCCTAGCAAAGGGCCTCTGCGAAAAGTTCCGCGAA
GATGCACTACGTGTGTTTGTTTCGGGACTCAAGCGTAGTCTCACAGATGTGCT
GTTCTCAGCAAAGCCAAGGGACTTGCCGTCAGCTCTGGCGCTCGCGCAAGA
GGTGGAATCGAACCATGAAAGATATGCGTTCGCTGCCAACTTCGCGAGAAGC
ATTGAGGAAAAAGAGTACAAGGCAAGTACCCAACAGCGGGTCAAGCCACCC
CAACAAGAAGCACAGGCAAGCGGGTCAAAGAACCCACATTTAGTAGGCAG
AACAAGCAGCAAGGCCAATCCGGCCAGCGAAATGCCGAGCAGCAAACTCGC
GCCAGTGCTCCAGAACCAATGGAGGTAGACCCCTCGCTGTCAAAATTTAGAC
AGCCCATCCAGGCCTCCGCTGGCGTCAGCAAGCGGTCGGCCATGTCCGATCG
CTCTAACGGAAAGGGCAAGCAACAAAGGGTGAATCATGTAACACAAGATGC
TGGGCAGGGGCAGACCTCGTACGCCGAAGCCGCCAACGGCGCTGTGGCGCA
GATCGAGGATGATGCCGAAAGTGATTCTGACGCCCTCAATTTTTAGGGGAA
AATCCCTGCTACCCGTCATTAGACGAAGAGTAGCAGGGCAGGATATGAGCTT
CCTTATTGACACCGGCACGTCAAAGAATTACATCAGACCACACAAGGGGTTG
CGTGGCGTCCGTCCGGTTAACTCGCAATTCACCTTGCATTCCATCCATGGCTC
AACCACCGTCACACAAAAATGCTTTGTTGCGTTGTTCAACGTGAAAGCAACA
TTTTTCATTTTACCAGACTTGTCAACCTTCGATGGGATAATCGGTCTCGATTTG
TTAGCTCAGGCTGGGGCGTCACTCTGTTTGGCCTCCGGTCAGCTCAAATGGG
GTACGGAAGTTGAGAAAATCTCCTTCCACAAATGCACTGATGTCAATTTCAC
CGATGTGGATTGCTCAGATGCACCCGCTTCAGTGCGGGAGACTTTTCGGAAA
TTATTAAAGGCCAGAAAAAAGGCCTTTGCAGACCCAAACGAGGCTCTACCGT
ACAATACTTCGGTGGTTGCCACCATCAGAACGGTGAGCGAAGAGCCCATCTA
TGCCAAGCTGTACCCATATCCCATGGGCGTAGCTGACTTCGTTAATAAGGAAA
TCCAGGATCTCCTAAGAAACGATATAATTCAGAAATCGGCATCCCCCTACAAC
AACCCCATATGGGTGGTAGATAAAAAGGGCACCGATGATGCGGGAAACCGCA
AAAAGCGCTTGGTGATAGACTTTCGCAAGCTTAACGAGCACACCATTCCCGA
TAAATACCCCATGCCAAATATATCAATGATATTAGGCAATTTGGGCAAAGCAC
GCTATTTTTCGACACTGGATCTTAAATCAGGATACCATCAAGTCGTACTAGCG
GAGCGCGATCGCGAAAAAACTTCTTTCTCGGTAAACGGAGGAAAGTATGAG
TTTAAGAGATTGCCATTCGGCCTCAGGAACGCCGCCGGCATCTTCCAAAGAA
CGATCGATGACATCCTACGGGAACAAATAGGCAAATTTTGCTATGTTTATGTT
GACGATGTGATCATCTTCTCGCAAGACGAGGAGGCTCACATCAAACATGTAG
ATTGGGTGTTAAAGAGCTTACAAGAAGCTAACATGAGGGTATCGATCGAGAA
ATCGCGTTTTTTTAAGAAAAGCGTGAGCTTTCTCGGGTTCATTGTCACCAGTA

ACGGTGCAACAACGGACCCAGAAAAGGTAAAGGCCATAAAAGAATTTCCAG
AGCCTAAGACGGTATTCGAAGTCAGATCGTTTCTGGGCCTCGCAAGCTACTAT
AGGTGTTTCATCAGAGACTTCGCTGCTATAGCAAGGCCCATTTCAAACATATT
AAAGGGCGAAAATGGAACAGTTAGTAGGCATAGATCGAGGAACATTCAGGT
GCATTTCTCTGAGTCCCAGCGAGAGGCGTTCCAAAAGCTGCGCAATATATTG
GCATCAGAAGATGTCATGCTCAGCTACCCGGACTATAAGAAGCCATTTGATCT
AACGACAGATGCTTCAGCCTATGGTATAGGTGCGGTGTTGTCTCAAGAGGGC
CGCCCTATAACAATGATTTCAAGAACTCTTAAAGACAGTGAAGCCAACTACG
CGACTAACGAGCGTGAGTTATTGGCCATCGTTTGGGCCCTGGCTAAACTGCG
GCATTACTTGTATGGAGTGAAAGATATAAATATCTTCACTGACCATCAGCCGTT
AACATTTTCTGTGTCGGAATCAAACCCGAACGCAAAAATTAAGAGGTGGAAG
GCCCGCATAGACGAGTTCAATGCTCGTCTATTTTACAAGCCCGGTAAAGAGA
ACCTGGTAGCGGACGCCTTGTCCAGACAACAGCTTAATGTGCTGGAACAAG
AAGAGCCCGAATCTTGTGCAGCAACGATTCACAGTGAGGTGTCTCTCACCCA
CACAATCGAGTCAACGGACAAGCCATTGAACTGCTTCCAGAACCAGATAATA
CTGGAAGAAGCACGTTTCCCGTCTAAAAATACCTTGATTTTATTCGGGAATAA
AAGGCGCCACACGGTTAACTTCGTCTGCAGGGGATCTTTATTAGACGAACTG
GCAGACATAATCGTCCCAAGGGCCGTAAACGCCTTCCATTGCGATTTGCACA
CGCTCGCAATGATACAAGATGAGATAGTCCGGAGGTTCCCAGCCACAAAGTT
CTGGCATTGCAGGAACCGTGTCGTGGACGTACTCAGAATCGAAGAAAGAAG
GGAAATCTTAACTGCTGAACATAATAGGGCGCACAGAGCAGCACAAGAAAAT
GTAAAGCAGGTATTGTCGGAATACTATTTCCCCAAAATGGCCAAGCTGGCCA
ACGAGATCGTGCAGAATTGCAGGACTTGTGCAAGGGCAAAGTATGACAGGC
ACCCGAAAAAACAAGAACTCGGTGAAACGCCAATACCGTCACATACAGGGG
AAATGTTGCACATCGACATTTTCTCTACCGACAAAAAGTTTTTCCTCACTTGC
GTCGACAAGTTTTCTAAATTTGCCGTCGTACAACCAATCGCTTCGAGAACTAT
TGAAGACCTGAAGCCAGCGCTGCTTCAGCTCATGAACTTTTTCCCAAGGGCA
AAGACCATTTATTGCGACAACGAACCGTCGTTGAAGTCGCACACGATCGTGT
CCATGCTGTGTAACAACTTTGGTGTAAGCGTTACAAACGCACCACCACTGCA
CAGCACCTCCAACGGGCAGGTGGAGCGTTTCCATAGCACGTTGCTGGAGCTA
GCCAGATGCATAAAGATAGACAAGGGCTTGAGTGATACGGTCGAAATAATAA
TGTTGGCCACTACCCAGTATAATAAGTCAATTCACTCGGTCATCGACAGGAGA
CCGGCTGACATCGTCCTAACACACCCCGAGGAGCCACAGCTAGAGATCCACA
ATAGGATCCAGAAGGCTCAGACCGCGCTGAGGGCCAGAGAAAACGCCTCGC
GGCAGAATAGGACATTCGATGTTGGCGAGAAAGTGTTGGTAAAATCCAACCG
AAGACTCGGCAACAAGCTCACGCCGTTGTGCGAAGAAAAAGCTGTAGAAGC
GGACATGGGGACCACGGTCCTTATTGAGGGGAGGGTGGTCCACAAGGACAA
TTTAAAATGACGCGCTCAGCAGCGAGGTCAGTGTTTCGCTTAATTTTAAATTT
TTTCTAGCCACTTGGCGTAATTTTTAATAGATCTAAGCGTAGCGCCATCCGCGC
ACATTATATTCTTAAGCATTTTTATTATTATTGGTGTTGGGTTCCCCTTGTCGAC
AAAATAAAAAAATCAACCATTTAAACTTTCACCCACAGGACCAATCTTATCCT
TCTCGTGTCTCTATCATTGGCATCGGCTCACATCACCGATTATTCGCGCGCCAA
ATACATTCCCATAGTCGATGGCCAGATCTTGGTGTGGGAGAATTTCGCCTACG

TGAGGCACTCCGCGAATCTTTCGGAGTACGCACGGGTAGTAGAGGAGACGG
TCGGTCTGCTTAGCCACTTCCCGCAGTCACACATGAGAAATTTGCTGAATGTA
GATTCAGCACACCTCCGGGACTTGCTGGATGCGCTGGGCGCGCACCATCGAG
TGGCCAGAAGCTTGGACTTCCTGGGCTCCATACTAAAGGTTGTAGCCGGTAC
ACCTGATGCCAGCGATTTGCAAAACAGTAGGGTCATAGAGGCGCGATTGATA
GACGCGAACAATAGGCAGATAGAAATCAATACAAAAATCCAGAGTCAAATTA
ATAAATTAACTGCCACCGTCAACTTAATCCTCAAAACAGCAAAAGCGTCGCA
AATTGACTCCGGTCATTTGTATGAAACGTTGCTCGCGAGAAATAGAATGCTCA
TGATGGAGCTTCAAAATTTAATGTTGGCCGTGACACTTGCAAAAATGAACATT
GTTAGCCCAAATATTCTTGATCACGCAGATTTGAGTTCAGTTTGGCTAGAGGA
GCCCACCAACACCCCCATAGGGGACCTCATGTCCGTATCGTCCGTAAAGGTT
CTGCAGTCCAATAACGCATTACACTTTATCATCAAATTTCCGATAATTAAGTTC
GCCTGTAAGAAAATTACTATTTTCCCCGTCAGTCACGAAGGAGCCATGCTACG
TTTAGATGATGGCATTATCGCAGAATGCGACAACGAGATCCACACCGTCAAA
GCTTGCACCGCATCAACTAGCGCCACCTTCTGCCAGCTATCATCAGGCAGCTC
TTGTGCGAAAGAACTCCATGCAGGCGGCGAGGCACACTGCGAAATACAACC
CAGCAGCCTGGACCCCATCACCTATGTAGATGAGGGGATCGTCATCATCAAC
GACAGGGCCGCCAAGGTACAAGTGGACAACGGCTCTGAAATCTGGGTGCAT
GGTACGCATCTTGTTACCTTCGCCCGACGTGCCGTCATTAACGGAACCCTGTA
CGTCAACCAGAACGGGATCCAGAACAGAGTACCAGGGGTACCAAAATTCCC
TCTGCTGAACATCACCTCCCACCAAAGCGTGCTCAGTCTCCCGTACCTTCGTC
GTCTAAATGAAGGTAACTTAGAGACAATCCGGAGGTTCAGAGAAGAATTCGA
CGCCGAAAGACCCCAGCGAGTGGCACTGACCGTAGCAGCATCATGTTGCCTC
CTATTATGTGTTGCCATTGTGGGCTGGCGCGCATGGAAGGCCAAGAGGTCCG
CAAGACAACTGAACGTGGCGATTGCGGAGTTAAGGTCGGCCGAGGGCGGCC
TCCGTCTTGAGGGGGGGGGGG

>Gypsy2-LTR_Gypsy#LTR/Gypsy2-LTR_Gypsy

AGTTATTGCCCTATAAGTTATCGTCCCACATCTTATATTTCAACATCATTTCTGG
GTAAACACAAGTGCCCAAAATGCTGACCAAAGGTCTGCATTCTTTCCGCTGT
CAACGACCAGCTAAAAGTGCGATCATCTGCACTTCTCCGCCGCGTAGCCGCT
GACGACCACTGATTCGCTGCCGACGCCTGCTGCGACGCTGCCGACGCCCAC
ACTTGATTGCTAGGGACTTAGGGAAATATTTTGTATCTTAGCTTTAGTTTCAAA
TGAACAATTACAATAAACGGTCGCTTGCGATCTACAAAATAAAAATCAATAAA
CTGTAATTATTTACT

>Gypsy2-I_Gypsy#LTR/Gypsy2-I_Gypsy

GGCGCCCGAACAGGGACCAGCGAATAACGCGTACGACAGACAAAATTCTAA
GTCGCGAAGCAAAATAAAATTTTGCAAAAAAAAAATTCGTTGGTTAAATTAG
TGCCGAAGAAACTCCCGTGAGTTAATAAAAAATTCGCGGTCGGCATTTATAA
AAAAAAAAAAAAAAAAAAAAACTTTTGTTTTCCGGAAGAGGAAAATATTCA
ACGGCAGAATATTGCCCATCGGTGGATCACATCTTTTGTCAGGCCAGCCCGCA
GAAGACCTTCTTTGGAGTGCTGACGTGGATCCCCAGTAGCACAAGGCAAAA

AGGATGACCTTCACCCAGCCAGGAGGAGTGACCACAGCAATTTTATTGTGAG
ACAAGAAAGAAATATTTTTTTTTATAGAATTTATTTTAAGATTTAAAAATTTA
ATACACTTGTACTTTAACATTTCATCGACCCCAGATCGGTTTCGACAGTACATT
TAATTTTTTAATTTCCCTTTTAATTTACTTACGGTTTTGGAAAATATAATTCAAG
CTCGAATAGTTCGAATGATTTAAATTGCCAAGATCATAACACTCGTGCCTTTTT
GTTTTGGTTAAGATCGTTTTGTGTAAGTGTTGTCCGAGTTAAAAGCCAACATG
GCAAACCCCAATAATCTAATAAGACCACCGGTTCTCAGTTCTAATGAGAGAC
CGGTACCCGTAGAAAGACCCGACCTGCCTGAACGGCAGCGCGTAGATATGAA
TGTTGAACAGTTGACAGGGTTAATTGGCCAAACGGTAGCCCAAATTTTACCT
GGACTGATAAAACAAATGAACAATGACACACTTGACTTTACTGACGTGAGAG
ATCAGGTCGTCGAGCCTGAATACAGAAACAATTTAGGGGATTTTGACAGGGT
ACCTGATATCGTAAAATCGATCAGGGAATTCTCTGGGGACCCAGCAGAATTC
GGCTCTTGGAAAAAGAGCGTTGATAGAATCATGGAAACTTATACCCCATTTGT
GGGTACTCCAAAATATTATGGTATACTTCATACCATAAGAAATAAAATTGTCGG
AAGTGCCGACGTGGCACTCGAGTCATACAGCATTCCGCTGGATTGGAATTCC
ATGTCAAGATGTCTCACTCTACATTACGCAGACAAACGAGACATAACTACACT
AGAATACCAAATGTCAATTTTGGTCCAAGGTCGCCAACAGTCGGTGGAAGAC
TTCCACCAAGACGTTTATAAAAATCTCTCGCTTATTTTAAATAAGCTTAGCTGC
ATGCAAATGACCAGAGAATCCGAGCATTTCATTACAAAAATGTACAGAGATA
AAGCTCTGGACACTTTTATTAGAGGCCTTCGAGGAGACTTACCTCGCCTCTTG
GCTATAAAGGAGCCAGCCGATCTCCCTTCAGCTTTGCATTATTGCTTAAAACT
TGAAAACCAAACATACAGATCAAACCATGCAACAAATAAAGGGCAACAGTC
GAGCTTCAGAGTAAACGAAAAACCCATACCGGCACCCCGCAATTTTCAGCCA
TCGAACGCCTTTGGCCATCGACAGCCTCCACCGGTCCCTCCACGAAACTTCA
TGAGGGGACCACCACAACACTTAGGTCAGCCATTCGCTGCGCCAAGACAAC
ACATGCTCAATTTCGGTAATGCACCGTTCGCCGCACCCCGACAAAATTATCAA
CAATTGCAACAATATAATGCCCCACCCCGTCCTTTCGCGGCCAAACCGCAAC
CAAGACCAGAACCAATGGACGTGGACCGCAGCATACAGACGAGAAATATCA
ATTATATGAACCGACCACATTTTGACGCCGGCAAACGACCGAGTGGACAGAC
TACAGACATAAACAAAAGACAAAGAAATTATAATATCCAAACTAGGGGCATG
GGACATCCAAGTGCCAAAATAGCTGGGCCCAGTTCAAGCATGACAGACTATC
AACGGTCGATGCAAGAATATGAAACCCAGAACGACATAAACGGCACCATGG
ACGAATATTGCAACGAACTGACGGACTACTTAAATGACGATGAGCAACAACA
ACAGATGCATTTTTTAGATTAGAAGACTCCTCACTGCCATACTTCGAATGTAG
AGTGAGGAGTGGAAAGGTTCTAAAGGTGTTGATCGACACGGGCTCCAACAA
AAATTATATTCAGCCAAAACTGGTGTCGAATGCAATACCAAATTACAAGCCTT
TCATAGCTGCCACTCCGGGTGGCGATATAAAAATAACACACTACAAAAGAGC
AGACCTTTTTGACTGGGAAATAAAATTTTTCCTCTTAACATCTCTTACGACATT
CGACGCAATTCTCGGCAAAGACACCCTGAAAGAAATGGGAGCACAGATAGA
TTTAGGAAATTTGACCATGACACTAGGAAATGGGAAGAGGATTGCTATCAAG
GAACGAAGGTTCGAGGCTGTTAATACAATCAGCCCCAGAATAGATCACTTGG
GACAAAAACAAAAAGAAAAACTGAATCGGGTAATTAATAATTATCCAGGTCT
CTTCGCAGACCCAAACCAAAAACTGACCTTCACAACAAATGTAAAGGCAGC

AATTCGAACCACATCGGATACACCTGTGTATTCGAAGTTTTATCAGTACCCGA
TGTCCCTAAAAGATGAAGTCAATAAACAAATAGCGGAGCTTTTGCACGACGG
AATAATTCGACCATCAAGGTCACCCTACAATTCACCAGTGTGGATTGTGCCAA
AAAAACTCGACTCCTCTGGTAAAAAGAAATACAGAGTCGTGATTGATTACCG
AAAACTCAATATGGTAACAGTAGCAGATAGGTACCCAATCCCCGATATTAACG
AGGTTTTAGCCCAACTTGGTGACAACAAAGTTTTCTCGGTGCTCGATCTTAA
AAGTGGGTTTCACCAGATCCCACTAAAAGACTCCGATATCGAAAAGACCGCC
TTCTCCATAAATCATGGAAAATACGAGTTCACTCGACTTCCATTCGGTCTGAA
AAATGCACCATCAATATTCCAACGCGCACTCGACGATATCCTTCATGAGCATAT
TGGCAAAATATGTTTCATCTATATCGACGATATCATTATCTTTAGCAAAGATGAT
GAAACCCATTACAAAAACCTGGACATAATTTTCAAGACCTTGCAACAAGCCA
ACATGAAATGTCAGTTGGACAAATGCGAGTTTATGAAGAGGAAGGTAGAATT
CCTGGGATTCGTCGTGTCCGACAAGGGCATAGAAACCAATCCAATCAAAGTA
CAGGCAATTTCAGACTTCCCAATTCCAAAAACACTCAAAGAACTGAGATCAT
TCTTGGGATTATCTGGATATTACAGGAGATTTATACCCAACTACGCTAAGTTGG
CAAAACCACTTAGCTCGCTTTTGAGAGGGGAGGATGGACGAATTTCCAAGAC
ATTATCATCAAAAAAATCCGTCTCCCTTAATAACGAAGCAATGGAAGCCTTCA
AGAAATTGAAGAGCAGTTTGATTTCCCCAGACGTGATACTCCACTACCCAGA
TTTTAAAAAAGAATTTCACCTAACAACGGACGCTTCCAATTTCGCAGTGGGT
GCTGTTCTTTCACAAGAAAACAGACCCATTTCATTCTTATCGAGAACACTCTC
GAAGGCAGAGGAAAACTACGCCACAAATGAGAAAGAAATGCTAGCCATTAT
CTGGGCTCTAAAAAAGCTCAAAACTTACCTTTACGGTAAAGCAAAGGTGAA
AATCTTTACCGACCATCAGCCTCTGACCCATTCCCTCAGCAGCTGGAATGGAA
ATGCGAGAATCAAAAGATGGAAGTCATACCTCGAGGAATACGACTATGAAAT
TCTCTATAAACCAGGCAAAGAAAACGCTGTGGCCGACGCTCTGTCCAGAGG
ACCAACAGCCGCGCAAATAAACTCGGTAACCTCAACAATGCACAGCTCTGAC
AGTTCGAGCCATGGGTTGATACCTAGCGTTGAAGCTCCAATAAATGCATTTAA
AAATCAAATTTTCTTCCGGAAAGCAGAGTCGGAGAATTACTCAGTTAGCATC
CCATTCCCGACATTCCATAGGCATTTAGTGGACCGTAAATTGTTCACACCTGA
TAGCCTCTTGTCAGATTTGAAGAAATATCTTAACCCATCCGTGATAAACGGAA
TTTTCACATCCCAGGATGTAATGGGGAAAATACAAATCCTCTACCCCATCCAC
TTTAAGGGTTTCAAGATTAGATTCACTCAGACTGAGGTCAAAGACCTTGTTAC
CGAAGCCGAACAGGAGGAAGAAATACTTAGGACACACAACAGAGCACACA
GAAACGCTTTGGAAAATAAAGCTCAGCTGTCCGAAAGAGTGTATTTTCCTAA
AATGAGGAAAAAAGTTTCAGCTATCGTGAGTCAGTGTTTGGTGTGTAAGACC
TCTAAATATGACAGACACCCCACACATCCGGAAATCAGAGAAACTCCCTTGC
CAGAATACCCCGGACAAATTATTCATGTCGACATCTATTCGACAGAACGATAT
CTGGTGCTCACAGCAATCGACAAATTCTCCAAGCTGGCTCTGGGAAGAGTCA
TTAAATCGAAAGCTATAGAGGACATTAGAAAACCCTTAAGAGATATCGTATTC
TATTATGGAGTGCCCAAACTAATAGTAATGGACAACGAAAAGTCCCTCAACTC
AGCTTCTATCAAATTCATGTTGGCAGACCAGCTGGGCATTGAGCTCTATAAAG
CACCTCCGTACAAGAGTACGGTAAACGGACAGATAGAAAGATTTCACTCCAC
ACTCTCTGAAATAATGAGATGCTTGAAAGGAGATGGGACACATAGAAGCTTC

GAAGAACTTCTTGATAGAGCTATCTATGAATACAACTTCACTGTCCATTCGGT
CACAAAAAAACGACCCCTAGAGGTGTTCTTCGGTAGGGGAACTACCGCGTC
ACCAGAACAGTATGAACAAGCTAGACTGGACAATGTAGACCGTCTTAGAAA
GAAACAGGAAACTGACATTAAAAATTACAACCGGACAAGAAAGCCCATAAA
GACCTACATCAAGGGCCAAGAAATTTTCGTTAGGGTTAATACAAGATTAGGAT
CTAAGCTATCAAACAGATTTAGAAAAGAAATAGTTAAGCAAGATAGGAATAC
TACAATACTAACAGAGTCAGGAAAAATAGTGCACAAAAGTAACATCAGATCA
TGATTACTTTCAGGATACTTACTGTGGTCGCATTGACAAATGGACTCATTGAA
ATAACTAACTACACCGACGCACAGACTGTGACGATTTACAACGGGATAGGAC
AGATACAGATAGGAACAACGAGAATTGTCCATATCATTGACTTGGACCACGTA
CAACTGACCATAGGAAAACTAACAGACTACATCGATCAGGACTTCAACGACG
ACAAGTCATATCACTTACTGAACTACGAACTGACACAGACGAAGAACTTGCT
CGACACTGTGATCCTGGCAAAGACAAGGAAAACTAGATCGATAAATCTTATA
GGGACGGCCTGGAAGTATGTTGCCGGTAGCCCGGATCACGATGACCTTGTAG
CTCTGACCGACGGGATAAACGACTTGACGGATAACAACAATAGACAGGTAAT
AATCAATAGACAGCTGGAAAATAGAATGAACCTACTAACCGACGTGACCACG
AAAATACAAAACTCAATCAGAAAAGATTCTACCTTAAAGGATGAGCTGGCCA
TAAGACTACAGAACCAGATTAGGCTCGTAAAAGAAGAGATAGTCAACATTAA
ATTTGCCATTCAATGGGCAAGGCTGGGTGTTATGAACACATTCCTGCTAAACG
AGTTCGAGTTGAAAGAGATCGACAGCCTACTCAAAATCAATAATATGCCGAC
TTCTGCCTTAACGGTCGAAGAAATGTTAAAACTCTCAGACGTTTCCGTTTTAC
ATAACGGAACTACAATTTTGTATGTTATCAAAATCCCAATTTTAAAACCAACTA
ATTTCCTAAATTATCAAATAAGGCCAGTAAAGAAAAACAATAATATCATTAATT
TACCAGCTAAAGAAATTTTTAAATTTAAAGACGAAATGTACGGAATTAATGCC
TTTTGCAAAAGTTCAAACAGTTTAAAAATATGTAAAGATAAATTCATTAGTAA
TTTAAGTAAAGACCTTTGTCTACCCGAACTTTTAAATGGAAAGCAATCAAGG
TGTGAGTTCGCCAATGCAGAACACATCCCTGCAATAGAAGAAATAGACGACG
GGCTAATATTACTCAACGACTTCAATGGCACGGTGAACTGGAGGGACATCGA
GAGACGATTGAGCGGCACTTACTTGATAACGTTCCTGAACGAGACCCTGAAA
ATCAACGGCCGAGAGTTCAGTAACAATGAAGTGGCTTCAACAAAACCTGCG
CCACCACTGATACAGCTGACTCCATACGAGGTGGGACGCCTTCGAATTCTCT
CGCTAGAAGTACTCGAGCAACTTCATCTGAATAACACATCTGAGTTACGCAA
ACTTCGAACGCACTCAACAATAAACAGAGTGTTCGGCGCAACAATACTAATG
ATGATTGCTGTCCTGATCTTCGTGACAAATCGGTGCTGCCGGAGAGGAAAAA
AGGTGGTCCTTCAAATCGACGCACCTGGCATTCAAACCATTGACGCGGTGCC
CAAACCACCACAGATCACCCCACCAACAATGCGTTTCAACAACATCCCGTTT
TTTTGATGACCGCTTGAGGACAAGCGTCGATTAAGAGGGGAGG

>GYPSY12A_LTRGypsy#LTR/GYPSY12A_LTRGypsy

TGTAGTGGCGACTGAAACTGTAGAACAATAAAGCTCCCGATAGACAAACTTT
AGGAAGCTTGCGCTTAGCTTTGGGATTCTAATTTAGGGTCATATTAAATGCAG
TATTATGCATCGGGAATATACTAGACGAGATGCAACTCTTTATCAGCTGCACCC
GGAAAGTTTTGGGATAATTTGGTTATCGTAAATAGAGTGACTTTCAGCTGATC

GTGATTTCCGCTGATGGCCTTAACCCCGGCTTGAGTTTCCGTTCTATTTCTTTT
CTAATCAAATCGTCGTGGAGTCAAGTCGTGAAAAGTTCGTGTTGTTGTGAAA
TTATAACCTGCGGGCGTGGAAATTTTGATTAGTGCTGAAAGATATTATTAGAG
TGAGTTTTCCTCGTCACAAAACGGCTAAATTGCTTCGCTTGGCGCAGACCCC
AAGGTTCGACTGGGGCAGTCACAGGTTAGTGAATATCGGGAGTGATCTGTTA
GTTGACGCGAAGGCTCTAACTTTTTAGGCGGGCAGAAAGATAGAAAAGAAC
CGAGCCAGGCCTAATGGAGGCCAGTGAAGCGCCCTTCCCATCGAGTGACCG
AAGAAGAGAAGCAGCCGCAGCGGGACCGCAGCCGCAAAATAAATCGAGAC
CCTGGGATGTGTATTTAAATTAATTGCCAGAAAATGTATTAATGAAAATAGAA
CCCCAAAGCAAGGTTAAGCGCGCGGGAAAATATAGCTAGTTAGGAGTCAAG
AATAAACAGTGAGAAGTTCCTGATGAAGTGAACATATAAGAGATCTTAAAAT
ATATATATAAAAAATGCTTGATGACCCCCTGTCTGACCTAACCCTACATCGGAA
AAATTAAAATTTACCGAACCGCCATTCTGAACTGAGAGAAATGAAGTTATATT
TTTAAATTATATTTTACAAAATTAAAAAAAAAGTTATAGAAATGTGTACTCGA
GGAGAAGCCTTAGTTTATATCACTTTTGTGTTTTTTAATTTTATTTAACTTTTTT
GTATTTAATGTTTTTTATTATATAAGCTAACCTAATCTAATTATTTTTCTAAGATG
AGTACATTTTCAGAGAGTGCGCGTTTAAATTTTCGGGTTAAATTTGAAAAGAA
AAAAGTCTTTTTAAAGTCGTTTGATTCGAAATTCGAGTGAAAGTACATGTGTG
TTCGTACATTTTCAAAGTATTCGTGGAAAATGCCACGCTTTCATTTCAGCTCAT
CTGCGCCGGGAGATCGATCTTTCGCCGAGGAGTAAAAATTTGAGTGAGTTTT
ATTTACAAAAAAAAATTTTTTATTACGATAAGTTATTAATATAGTAGCGAACAA
ATTTTAATTTTTCAATTATACACCGGAAAAAATCATGGAAGANANNTNNNNRN
AGTAANNAAANNTGTTTTATTTATTTTTGAACTTTGCTTTCCATGTGATTTAAA
AAATTCTAATGAATACACTGCAAACAATTTATTGGACCCTAATGACATTGCGG
AAGTAAACAATTAAATATTAAAGGAATAAAATCTAACCGAATTTACTCGCAAC
CCACTCTCTTACGTTTTTTTTCTTTTTCGATTAAAGAAACTGATTTAAGAAAA
ATAATAATTCATGGAAAAAGAATATAGAGAACTGAAAAAACTGAATTATGGG
AAAATTTAAATAAATAATTTGTTCCTCTAAAATATATTTAGCAACTAGTGCTCT
TCGCATCTATTTAATTATAACTTTCTTAAATAACTTTTATAAAAACTTTCTTTTT
GAATTTACGACAAAAACAAAAACCCATTGCCTTATATTTCCTCAACTTACTTT
AGTTTGTATATTATAAATATTGTTATTAATTATTAGTCACCACCAGAAATACAAA
AATTAATCTGTAAAATTTTAAATACTGAACTCGGCTGATGTTTCTCCGGACATA
GGGCACCTAAGGGGCCATTGGAGCCATGCTCTTAAGGAAAGTGGTCATAGGT
AGGGTAGGGCAATCCGCGAACACGATTACACCGGTGTTTCGCAGGCAACAA
ATTCTCAAGCTTTTCCTCCTCTTCTAGAACTGTCTTCTTCCGAATATATTAATTT
GCTTCCTCCGATTTTGTATACTAGCTCCTCTATTCTTTTGTCGCAAGCACGCTC
AATTCTTGATCTTATTCATTAATGTCTGTAGCGAAAAGGTATCTGTACAAGCAA
GACCACCACACCCAACGCCAACGATCTAGAGCCGGATTCTAAAGCGTGCACC
CGCCAGTACAGGTTCCCTTGCCTTCCATCAATTTATTTCGTTACACGTTACA

>GYPSY12_LTRGypsy#LTR/GYPSY12_LTRGypsy

TGTAGAGGCGACTGAAGCAGTGGGTGAGAAAAATCTCGATGAATAATCGATA
GGGCGGTCAGCTGTGACACACTAAAGTGAGGTCTTACTAGATGTAGCAAGAA

GTAGCTAGAATATACCAGACTGGATGCAACCCTTATTAGCTGCACCCGATAAG
TTTGGTATAATTCGGTTATCGGAGAAAAGTGTTGTTCAGCTGATCGTGGTTTC
CGCCGTTGGCCCACGCCTCGACTAATGTTTCCGGTTTGCCTTCTTTTCGATCT
GAACAGTCAACGCGTCAAGTTGCGAAAGTGCGTCGAAAAATTTTAACTTGC
GGTCATACGGAATTTTTTTTTGTCGCGCGAAATATCGAAAATACGGCGTTCCT
TCGCCTATAGTGGTGCGCGAAATAATTCCGGCTTGTGTTTCCCGGCGATATAC
AGCCGAAGAGATCGGCGTTCGTCACGGACATCTCGCGGCCGCCTGGCCGCC
AAATCCTAGCTTCGACTGGGTCAAATTGCAGGTTAGTGAGTTTCGAAATATTG
GTGAACCGTAGTTTGATACGGGAATATAATCGCAGCCGAAATTGAAGCCCGG
AGGGTTTTCGCTGGACAACTTATACTTTCGCATCACTGCCCGGGTCCCACCAT
TCTTCGGAGCCGCTACCGGCGGGCGGAAGGAGAGACGGCAACTGCGCCAGG
CGAGTAGCGGAGCGCCTTTCCAGCAGGCCGGAGCGAAGGACCAGCCGCAGC
AGCAGGACAGCGACCGCCCTAAGCCGGGGCCATCAGGAAGGATCATTAAAC
AAAGAAAATGCAGCCTAAGTAGAGCTTAGAGTGAGGTTAGGGTGAATGGAA
AATTTTCTTGTGCTTGATTGGCAGTCCAGAATGCCCGAAATTAAATTAAATCA
AAGTTAAATCGAGCAAAATTTCTAATTATACAAAAAAAAAATGCTGAAAGCC
AGACCTAACCTTACTAGATAGTATTACTTCAACCTGATCGCCATCTTGGACATT
GAGTGGCCGAAATTAAAATTTTTCCTATGTAGTTTAAGTTTAACGTTAAGCTAT
AAGAATATGTACTCGAATAGGACACTCGGTCATACGGTTTAATAGTTTTCTTG
GATTTAAAAAAAAAAAAATGAATTCTTATTAGTGATTAAAATTTTTATATAGTA
AGTGATTTAACCTAAGCTAATTAATTGTTTTAGAAGAGTTCTCTTTCGGAAAG
TGCTCATTTAAATTTTCGAGTAGGGAATTACAAAAGTTTGATCGCAGTCGTGT
ATTCCAAAATTATGCCTCCTTATAGTGTTTGTGGAAAAAAAAGCCACCTTTTC
GTTTCAGCTCATCTGCGCCGGGAGATCGATCTTTCGCCGAGGAGTGAAAAGT
TGAGTGAGTCTCATTGATAAAACTTTATTTTATTGGAAATATTCGGTAGTAAAC
CGATTACATTTTGATAGAGGTCATCTTATGTTGTATACACACTGCAAATAAAAA
AAGTAATAATAATGGAGTGGCGAGAGCAGCCATTTGAAATATAATGAGAAAA
AGACAAAAAAAAAAAATGAAAAAAAGATCTAACCGAATTTACTCGCGGCTCT
CTCTCAAACAGAATTAAATTTTTTTTCGTTAAAGATTCGTTATAAAGAAAACT
CAAAAAAAAAAAATAAATGAAATTAATAATTCACAGAACGAGATTATAGAGA
GAACGAAAATTATTAAAGATTTAAATGAAAGCTTTGTTCCTTCCTAAAAGAAA
AAAACATATATTTAGCCACTAGTATTTTTAACTTTTAATTGTAATGATCTCGAAC
ATCGAATCGAATCTACATTTTCTGATCTCGCAACTGAGAGAAAAACTTTGCTT
TGAATTCCTTTGAGTTACACTAGTCCGTATTTTTTATATTTACTTGGTTCCTTTA
GTATGTACTTATTTTTAGATATAAATATTGTTATTAATTATTAAGCATTGATTGAT
TTTGAAAATATGAATTTCTAAAAACCTAAATGCTGACTCGGCTGATGTTTCTC
CTGACGTAGGGCACTAAAGGGGCCGCTAGAGCTATGCTCTTAAGGAAAGTGG
TCTAAGGTAGGGTAGGGCAATGCGCGAACACGATTCCACCTGTGTTTCGCAG
GCGAAAAATTCTCAAACTTTCCTCCCATTCTAGAACTGTCCGCTTCCGAAAGT
ATTAATTGGCTTCCTCCGATTTTTGTATTCTAGCTCCTTCATTTTGTTGTTGTTG
CAAGCACGCTCAATTTCTTAATCTTATTAATTGTTGTCTGTAACGAAAAGAGA
TCTGTACAAGCAAGACCACCACACCCATTGCCATGAGCTAGAGCCGGCCTCT

AAAGCGTGCACCCGCAAGTACAGGTACTCCAGTCGTCCATCCGTTTAATTCG
TTACACGTTACA

>GYPSY12_IGypsy#LTR/GYPSY12_IGypsy

GTTTAGTTAAGTATAATAATAAATAAGATAAAGTGGCGCCCAACGTGTGGGGC
CTTAGTGAGTTTTTTTAAATTCATTAAGACTCGCGAATTAATATCTTCAATCC
TTTCTTTACATTTTCTGTCTCACTTCCAAAAATTTTTGTGTTTAGAATTGGTTG
CGGAGTACACGTCAAGTCAATACTAATATTGCTGACTGGCCATAACTAAGACT
GAKCACAAAAATTTTCCTWAGTGTAGCAAAAGGAAATGTGGATTTTGCTGAG
GAAAACAGAGTAACAGTAACTTCGGTCTGGTTTCTGTCCCGGTTTAATCTAC
ACTTCCTTGAKCTAACTAAACGCTATTCAAAAAAAAAAAAGATCATTAACGC
AACATGACGGAAAATCTTTGCGTTACTCAAGAGAAATGCAAGATCCTCATATA
GTTGACGATTTTCCTCTTTTTTACTATTCGTTTTAAATAAGATAGAGTTTCCTAT
AGGTTGGAATATTCGTGCCAAGGAAAAGGCACGATCCAGGACTTCACTGTTA
CACCTACTGGTGGTGTTGAAATTTAGTCTAATATSGAATATCTTATTTTAACGA
AACTCCTTCCTCTCCTACGCCTTCGTGAGAATTTTGAGCCAAAATAATAGACA
GCCCTACGAAAGGAGAACATCAAGCCAAGAGTTCAGTTTTATTATCTTTTCGC
GCCTTATCGTTGATATTTTTACGTATAAGTTTATTCTATTTTTGCTCGCCATTTTT
GAACCGGTAATTTTTCGCAAGTATGATGGGGTTAGACCGATCACCAACTAGG
AAATCTCCTAGTGTCTCAAACCCTGTTTGTAAATTATGTGCAGCCGAGATCAG
CACACAAGATTTGTATGTGACAACCTGTCACCATGAATTTCACAGGGAGTGC
ATTGGCAATCATTTTAAAAAAAGTGAGATCTGTCCGAGGTGCAAACTAACCT
GTAGGCCTCCAGCCGAAGCGACCGAAAGGGTAGGGAGAGAAACTCGGAGTA
AAACTAAAAATCTTCTAGCCGCAACAGTAGACGGGGGTCCTTCGACATCAGC
CAGCGGTGCTAACGCAAATGAAGCTAGTTCAAGCGCTGTAAGTGCTAATGCA
GCTTTGTTAGCCATGGAGCGTAGGCTTCTAGCAACGTTGTCAGAGAAGATGG
CTGATTTAGTACAAAACGCTATCACCTCAAGCATGCAGCGAATAATGCCCACT
CCGAGTCCAGCTGTAGTCGTTACAGCCAGTGAAATGTCMGCTGATCACCCAA
ATGCATATGAGAGGCAGTATTTAGCATCGCCAAATCCAGTTCCTTCTCCACGA
AGCGCTTCCTCCGATTTGTTTGATCGGCCAGATAAAGTCGTCCACATATTAAA
TGGTTGGAAAATAAAATATTCTGGTGTAGGAGTGTCAGTGGACAACTTTATAT
ACAGAGTCGAAGCGCTTACAAGGCAGACGTTGAATGGAAATTTCAACTTGTT
ATGTAGAAACATAAGTGTATTGTTTGAAGGCAAGGCGAACGACTTCTTTTGG
CGCTATCATAAAGCCAACGGTGAAATTCTGTGGGAGAGGTTTTGCACAGCCC
TACGACTGCAATTTCGACAGAGTCGCGACGATGGGGACATAGAGGAACTGAT
CAGGAACACCAAACAAAAACCAAATGAAACTTTTGATAGTTTCTACGACACG
GTATCCGAGTTAGTCGACCAGCTAGAACAGCCTTGGACMGCCAACAAACTT
GTCCGCGTGCTAAGAAATAACCTTCGTCCTGAGATCCGCCACGAAATTCTAA
ACTTAGATGTCCGAACGGTGTCAGAGTTGAGAGAGATTTGTAAGCGAAGGG
AGGCATTCCTGGCTGACGTCAGAAGATGCAGTAGCTACGCGAAAGATACTCC
GTTTAAACGCGAGATCTCCGAGGTCTGTCACGAGAGTGAAGATGAGGTGAG
GTCAACATACGAAGCTGAGAACGACATCGAAGCTTTTTCCCTAGTGTGTTGG
AATTGCCGAGTCGAAGGACACCGATACCAAGAATGTATAGCCGAAAGACGA

GTGTTTTGTTACGGGTGTGGTGCAGCAAACACCTACAAACCAAGTTGTAGAA
AATGTTCAAAAAACTTCAAGGTCGGCATGTCGAAGTTGCCAGTCAAACCGA
AGACTTCGAATGCCGCAAGGAATCAGTCGACAATGACCGATCAGTAGAGAA
CACCGATAAAGGTGTGTCTCTACCCCTCCCTGACGTACCAATTAAACCGGCTA
TTAGACTGCTTCACAGCAAAATTTCGGAGTTACGAAACGTCTGCATTTCGGAT
TCCAGAAAAACTCCTGCAATTCTTAAACGGAAATCTCGTAGTGCTCGACGCT
TGAAATTGTTCTGGAAGAACGTAAAAAAATGTAAAGCCAGTTTGGATTACAT
CAGTTCTATTCCAACAGGACCTCGAGACCCTCGACCGTTCTTACCGATTCGCT
TATTGAATCGCCTGGTCTACGGATTGTTGGATTCAGGCGCATCGATTAGTTGTA
TTGGAGGAGGGTTAGTGCAAGCTGCGATGGAAAACGAGAAGTTTAAGTCKT
TAATAGGAGAAGCTGCGACAGCAGACGGGAATTCTCAACGTATAGTAGGACT
GCTAAAAATAGAAGTGGAATACGGTGACATCAAAAAGCTTTTGAAGTTGTAT
GTCGTGCCATCGTTAAAACAGGATCTCTATTTGGGAATTGACTTTTGGAAACT
ATATGACCTCCTTCCTGCTAATTTGAAWATAGCTGAGGTATTGTCGCCKGAGC
CCAACCAGCAAACGGTGGTGGATCAGCACGAATTATGTGAAGGTGACAAAG
CCAAGTTGGCCAATGTAATCAACTGTTTTCCTTCCTTTAGCCAGGAAGGGTTA
GGTAAGACGAACTTGGTCTCTCATTCGATCGACGTGGGCACCGCTAGGCCTG
TGAAGCAACGGCATTTCCCTGTCTCCCCCGCCGTCGAAAAAGCAATGTATGC
GGAAATCGACCGGATGTTACGCTTAGGGGTGATAGAGGAGTCTGAAAGTGCT
TGGTCTTCGCCGATCGTGATGGTGACTAAACCAGGCAAAGTCAGAATTTGTC
TTGACTGTCGCAAGGTTAATAGTTTTACGGAGAAGGATGCATATCCGTTGCCC
CAAATAAACGGGATACTGAGTCGTTTGCCGAGAGCTGAGTACATATCGAGCC
TAGATCTGAAGGATGCATATTGGCAGGTCCCTTTGGATCCTAAGTCTCGGGAC
AAAACAGCTTTTACCGTCCCGGGTAGACCGTTATATCAGTTTAAAGTCATGCC
TTTCGGGTTGTGTAACGCCACGAGCACCATGTCACGGTTAATGGACAAAGTA
GTGCCGGCTCATTTGAGAAACGAGGTTTTTATCTATTTAGACGACCTACTAAT
AGTATCTTCTTGTTTTGAGAGCCATTTGAATGTACTGAGGGAGTTAGCCCTGC
AGATAAAGCGCGCGGGCTTAACGCTAAACGTCGCCAAAAGTCACTTTTGTAT
GCGACGAGTACGCTATTTGGGCCACATTATCGGAGACGGTGGAATTCGCACA
GACCCTGAAAAGGTGGCCGCGATTACCAATTTCCCATTGCCTAAAAGTTTGA
AAAGCTTGCGCAGTTTCATGGGATTGTGCGGATGGTACAGGAAATTCGTCGC
AAACTTCGCGACACTTTCTGCACCATTGACTGACTTGATGACCACGAAGCGG
AAGTTTGTTCTAACGAAGGAGGCAATTGAAGCGTTCAGCAAGCTCAAAGAG
TGTCTTAGCAAAGCTCCAGTCCTGTGTAGTCCGGATTTTGCGAAGCCGTTTGC
CATACATTGCGACGCTAGCAAGTCAGGCGTGGGTGCTGTGCTGGTACAAGTG
TCTGAAGAAGGTGACGAGCGTCCTATCGCTTTCGTTTCGAAGAAGCTGAACA
AAGCTCAACGAAATTATACCGTCACAGAGCAGGAGTGTTTGGCCGCAATAGT
AGCTCTAAAAAACTTCAGAGCATACGTGGAAGGACTCCCTTTTAAAATAATA
ACCGACCATGCTTCGCTCAAGTGGCTAATGTCCAATCACGATCTAAATTCACG
ACTGGCGCGATGGGCATTAGCTTTGCAGAGATTCAAGTTCGAGATTGAACAC
CGTAAGGGTTCTTTAAATGTCGTCCCGGATACATTGTCTCGTGTAAACGAGGA
GATTGTAGCCGCGATGGACTTGCAAGAAGACTTAATTGTTGATTTCGATTCTG
AATTTTTCCAGTCCGGTGACTACGTAAAGTTGGTAGAGACCGTAAAGGAAAA

TACCTCGAATTTTCCTGATCTCAAGGTGGAGAGCGGGTTTTATATAGAAAAG
CCGAGCACCTGACTGGAGAACGAATGCATGACGAATACGCTTGGAAACTTTG
GGTCCCCAAAGAGTTGGTATCGAAGATTCTGGCTCGCGCACACGATAGTCCG
TTAGCTGCGCATGGTGGCATACACAAAACCTTGGAAAGAATACGGCGATATTA
CTTCTGGCCCGGTCTCGTTTCGGACGTGAGGGCTTATATTAGTGCGTGTGAGG
TTTGTAAGAGTACAAAATCTCCAAATTTCACTCTCAGACCACCATTGGGAAA
AGCGCCTGAGTCTCAGCGATTCTTCCAGCGTTTGTTCATTGACTTTCTCGGAC
CGTATCCTAGGTCAAGGAGCGGCAACATAGGAATCTTTATTGTTCTGGATCAT
TTTTCGAAATACGTGTTCTTGAAACCCGTAAAGAAGATTGATTCCAGTGTCGT
CATAAAGTATCTGGAAGACGAGTTGTTCATGACGTTCGGAGTGCCCGAAGTG
ATACTGTCTGACAATGGTTCCCAATTTCGAGCTAGGACGTTCCAGAAACTTAT
GGAGCAGTACGGCGTTAAGCACACGCTGACCGCTGTCCACTCGCCTCAGGC
AAATGCTTCTGAGCGCGTGAACAGGTCAGTAATTGCTGCAATCAGGGCCTAT
CTGCGTCTCGACCAGAAGGACTGGGACGAGTTTCTCAGCCGAATATGTTGTG
CGTTGAGGTCGGCGGTGCATTCTAGCATTGGCACCAGTCCTTATTATATGGTAT
TTGGGCAGCACATGATCACGTCAGGGTCGACGTATTCGTTGATCAGACGCCT
AAATCTCCTGGACGATCGTTCTCTAAAGTTCGACCGGCACGAATCTTTCGAG
ATAATGCGGAAGCAAGCTGTTGATCAGATGAGAAACAAGCACAACGAGAAC
GAGAAGCGGTATAATATCCGTTCTCGTGTGGTATCGTTTGTCGAAGGGCAAGA
AGTTTATCGGCGGAATTTCAAGCCAAGCTGTTTCCAAACCGGTTACAACGCC
AAGTTTGGACCGACGTTCGTGAAGTCTCGAGTTCGGAAGAAGATCGGCAAC
GCGTATTACGAGCTGGAGGATCTCCAAGGACGAGTTGTGGGTACCTATCATG
CCAAGGACATTCGGCAGTAAGGTGCTTCCAGTCGGTCTCAGCCTAGTGTGTC
TATGAGTACCCTAGTCTGAGTGTAGCGGGGGGTTTT

>GYPSY11_LTRGypsy#LTR/GYPSY11_LTRGypsy

TGTGGTATGCGTACATATATATTTAGAGTACATATATACATAGAGTACAATGTAC
TCAGAATACTTACATAAATCACTAACATAATTACATACGCATTGGCAGTGCTAG
CATAAACAATAATGTTCAAGTGGAAGTGTTATATGATCGCCCACTTATGAAGC
ACACTGCATAAGTGCATGGTCAGCATTCCCACGCTAACCAAATGCCGCTATGT
ACATAATGTGCGCACATAGCATTTTCTCTCAACTCTCTTAACATAARTATATAAT
ATAATATATAACAGCCGCTTCTCTGCCGACTTCACCAGGCAGCGAAGGCGCA
CTTCATAGCCTAAGAGGAATATTGTATTAGCTTTAAGAGTCAGTCGAGTTTTA
CAACTGATACGAGCTTCACGATAAAAATAAACGGAGCTATGCTCCAGAAAAA
CATCTAAAGGATTTTCAATATAACA

>GYPSY11_IGypsy#LTR/GYPSY11_IGypsy

TGGCGACCGTGACAGGACAGGGACCTAAGGCCTAAGGACATAGGGCCTAAG
GACTACAATCACAATCAACACAAAATTTGTATTGGACTGGACTGGAAAGGCA
GATGGAAGACAAAATTCTCAGTGAGTAGAGTAAGGCAATGAGAAAAAAGCC
CGGCGGTGGGCGGCAAAAGTTAAAACCGAGAATAGCTCACCTAAGGTGGCT
ATTATATTCTCAAGCAGAGCTTGACTTCCTACAACAAGAATTAACAAAAGCGC
CAATAAAAACCAAAAGCCAAACTCTTTCATTCATCAAAACCACAAATAATAC

AAAAATTAAAACTGTAACACCACCACCCAAAGCCAGCATACCAGTTTACAAG
GAACAATGCCCGTTGGACTTCGAGGGACAATGGAGAGCCTATTGCAAATACA
CTTGCAAACATTGTAAGGACATCAACCAGCAGAAGGAGACCTGTGCGACAA
ACAGCACCGGCACCGCTTAACGCGTCAAGTTAAGCCAGTAAGGGCACCTCTA
ACCACCAACAGCAGCAGCAGTATCAGCAGCAACAACAGCAGCTTGGGCAGC
CAGCACCAGCACCGTATGGAAAGGAAAAGGAAACCGACAGCGCTTCCGAGA
GCGCAATTTTTTTTTCTCAATAGCACTGCGTTGCAAAAATTTTTTTAATGCA
CTACGATGCATATTTTATTTATATAGCCTATAAAGCTATACACATCCAACAGGCA
GGAACATATGTAAAAATTATAAGCAAACATTTTTTACATATACATTTTAGCAAA
GAAATAATAAACCTAAAATTCAAAAATAAAGATACAAATATTAGAAAAATGTT
TAACGTAAATGTCAATAGTGACAAACAAAATATATAAAGAATAAATTTATGAA
AATACTGAAGAGATCTTAAAACCCAATATGGGCTTAGTTGATACAAAAACAG
AACCCACATTTTTTTCAAATGCAAAATGCCACAAACAAAGTGGAGATAGAGT
CAAACAATTTAAGCTATATAACAATAATCTTGTTAGCAATTTTTACTAATGATC
ATACATGCACTCATAAAAGTGTATAAACTCCACAATAAATGCCTGAAGAAACG
ATATAATAGCATGGCCGATAGCCTAGAAACAATTTAATTTTTAAATAATGAGCT
CATTAATGAAAGAAAAATAATAATAAAATCTAAAAAAAATTTTTTTAAATACT
ATTATGTATTATGTATAATCCGAATTATGGATTGGCAAAACTTAACAGCCATCTT
AAATAAAATAAAAGAAAAATTTGATAGGTCACATAAAAGTTTGTCTCAGAATA
GAACAATCCAAAAACAAACTGCTAATGACCATACAACCTTTCTGGTAGAATC
CTTCAATCAGGTAAGATCCCTTATACATGACCAAAGAGGGAAACTAGACAAA
AAACAGTGGACAACAGTGTCCAAATTTTTAATTAGACAAGAAATACTATCCCT
ATTGTAATAAATAGCAAAGAAAGGGTTCGATCTAAACATATCGATACCAACAA
TTCTAAATTCCCCTTTAACTCTAAGTGAAGAAGAAACAGAATAACTCAATGAT
TCTGACTCTGAACTAGAAATCAAAGAAGAAGACTTGAACGATCTAACAATAC
CAGCAATGTTGCAATTGCCAGAAGATTAAAAGAATATAGAAGATCAGAAAAT
AAAATTAAGCAAAATGACAGACAATTCAGCCGCCATTAGGGTCTACATAAGG
GAAGTGTGGAGCGCAGTACCGGAGTTTGATGGACAAAAGATCCATCTTCAGA
GGTTTATTGTGGTCATCAAATTAGCAGACCTTGCAAAAGGATAATTTAAGGAC
ATTGCAGTCCAAGTAATAAAATCAAAATTGATTGGCACTACTCTAAACCTGGT
ACTGAGCGAATCTACAATTGAAGCAATAATTAACAAACTGCACACTTCAATTG
TTGGGGAGACATCCCAAAACATCAAGGCAAAATTATCCACTGTACAACAAAC
AAAAAGGCAAAACAGCTACACAGTTCACAACCGAAGTTGATAATTTACGTGA
GCTCTTAGAAGCCTCGTATATCGATGAAGGTTTACAAAGTGAGCAAGCCATC
GATGTTATGATCAAAAAGACCAAACACGAAAGTGTGAAAACTGTGCTAGAA
GCAGGTACGTGCACAACTATGGATGATGCCATAGGTGCATAAAAACTAGCAC
CAGAGTAACCGGAAATGTGAATCATTTAATGTAAGAGAGGTTACTCTAACTCT
AGAGGCCAAGGTAATGGTCGCGGCAGAGGCAATGGCCATTACAATAGTCGAT
ACAATAACAATAATAACAATTACGGCGATAACGGTATCCAAAAGAATGGTAAC
AATTATAGATGCCGTGGCAACTCAAAGAGAGGAAGCCGAAACAATCAGAATT
GAAATAATAACAATAACAATGATAATGCTAGTGTGAGAGTAACCCAAAATAAT
TCGGGAAACTCGAAAAAAGCCTCAGATGCACGGCAATAAAGCTCATGTACAT
TTAATCAATCTAAGTATTAATATATTGCATTTGATCATGTGCATTTGCATTTGAC

CCAATTCGACAGAGTGATAGTGGACACTATAGGTCCTCTACCAAAGTCGGAA
AATGGCAACGAATATGCAGTCACTCTCATTTGTGACTTAACTAAATATTTAGTT
GCGATTCCCATACCAAACAAATGCGCAACAGCAGTCGCTAAAGCAATTTTCG
AATCTTTTATTCTTACTTATGGTCCAATGAAGACGTTCATTACGGACATGGGGT
ACATAATATAAGAATTCAATCATTACAGATCTTTTCAAATATCTAAAAATTAAA
AATATAACGTCTACCGCTCACCATCAAACAGTAGGTGTAGTCGAAGGAAGTC
ATAGAACTCTAAATGAATACATACGATCTTACATATCGGTTGATAAAACTGATT
GGGATGTATGGATTCATTATTTCGTCTATTGTTTAATAGAACCCCTTCTATGGT
ACAAAATTATTGTCCATATGAACTTGTTTTTGGTAGAACAAATAATTTACCCAA
AAATTTTACTAATATAACTAGCATAGAACCAATATACAATATAGATGATTATGCT
AAGGGAAAGTAAATATAGATTAGAAGTAGCATATAAAAGAGCTAGAACTATG
CTTGAAAAGCATTGAAAAAATAAGGAAAATTATGATTTACAAACACAAAATA
TAGAAAAAACAGTAGGATATAAAGTTCTATTAAGAAATGAAGTAGGTCGTAA
ATTAGATTTTAAATATACGGGACCCTATACGGTAGAAAATATAGAAGAAAGAG
ACAACATAACAATATCAAACAATAAAAATTAAAAACAAATATTACATAAAGAT
CGATTAAAAGTTTTTATTCATAAATGACGTCATGATTTAATACAAAAAGCATT
ATAATAATGGAAAAATACAATTTAAAATGTCAAAAACAAAAAATATATATATAT
AAAAAAAAAAACAACAAAATAATTATTTTTAAAAGGAGGGAGA

>GYPSY10_LTRGypsy#LTR/GYPSY10_LTRGypsy

AGTTAACACGAGCAAGTCAGAGGGGTGCGACGACCCGCCGAAATGCCATCC
AAGACCGGATGGACCAACAATAACATCGGCGACGCTGAGCCAGCCCAGTAA
CGCATTTCGCTTTAGCGAAAGCGATGTGCAAGGGTCAGCGTAATGCGAGGCG
GGCATGTGCGCGCTGGGTGACCGTCGAAGGCACGGAACTCCAGACGGCACG
AGGCGCTGACTAAGCGCTCTGCTGGGACCGCTGCCTCCAACCGAAGACTTG
GCGGCCTGAATAAATTTGATTTGCATTTAGTTGGTAAGATACCAGAATTCAGA
GCAGTTGGCTCTAATAAAAAGAACCATTCAATACAAACGTATTTGGTTTAATT

>GYPSY10_IGypsy#LTR/GYPSY10_IGypsy

GGCGCCCAACGCGCTTAAGTGTCGTAAAACCGTAAAATCCGTAGTAAAAAGA
TCTAGAAATCAAGGTCTCGTAATCGGGTTTTCTTTGAAAACACTTCGGACTCG
CGCGCTGCACAATAATAGTGAACAGCAACAAACAAACAAAATTATCTAATAC
AAAAACAAAACAACGCTGCACAAGCAACAGTGAAAACCGCTGCATATCAAC
AGTAATCAGCTAAATCAACGACAGCCAGAGGATAAACGGATCAACCAGCTAC
CGTCTTCGATGCATTTTTATTATACCAGTATACCAATATAACATGTAAGTGAGAT
CTTTATTTTCAATTTACCTCTTTTAACGTCAACCAGACATGTGTGCCAATTTTT
GAAAAAAATACACATAATAAGTAACGAAATAAAACAAAAAAAAAGAGTGAT
TGCAATTAAAAGTTCAGGTATAGCGATTTTCATTTACAAATTAAAAAAAAAA
AAAACAAATGATGAAGAAGGAAAATTAAGCTCTTTCTCATACAAATTATAAA
AAAAATTTAAAAAGTATCATTGTCCGATGTATAGAAACAGTAACGAAAACAA
CGACAGTGACGAAGACTTAATACAGAGCTATAAAGACATAAGAAATAATAGC
CATCAAGGGGGTTACATTGACGGTACTGAGACCGACGAAGATATAGAATTAA
GTAACTTAATTGACGGCTTTACAGACAAAGTAAAAATGGTTAACGACCAGGA

ATTTCAAAATCAACATCAGGACCTTCAACAAGAGACAACAGCAACACAAGA
ACAGGCGAAAACCTCAGGTACCATTAGTAATAAAGATGTAATTAGACTCATGG
AAGCGGCCGTAAATGGCGCACTAGAGCATCAACAAAAAATATTTTCAGAAAA
GCTTAAAGAGGTTGAGAATAGGCAGAGAGTAAATGAGGGTGGACCCCTAGT
GGTATACGAGCAAATACCACATAACTCAGCTATCTCTTGTAATGAACCGCTAG
ACTTAGTAAAGTCCATACCAAGTTTTGACGGAAAACAAGATGAGTATGTGGC
ATGGCGAACCGCAGCAGTTAACGCCTACGAAATCTACAAACCATATTTGGGT
AGCTCTAGAACCTACCAGGCCGTAGGAATCATACGTAACAAGGTAATAGGCC
CCGCGGGAGTAATGCTTACGTCACATAATACAGTACTAAATTTTGATGCTATAA
TAGCTAGAATGGACTGCGCTTATGCAGAACAAACTCCAATCGAGGTAACGCA
ACAACAAATGGTTACTATGCGTCAGGGAGAACTTCCCCTAATGACTTTTTATA
ATGAGATTGAGAGAAAGCTAACTCTTATTATTAGTAAGACTTTGTTGTCTTATG
ACACAAATACTGCCGCTATCTTAAATAATAGATCACGGCAGGACGCTTTGACC
GCTTTTGTGACCGGGTTAAAAAAATCAGTCCGACATGTAGTTCTCTCAGCTG
CGCCCAAAGATTTGCCATCAGCATTGGCTGTGGCTCAACGGGCAGAGTCTTG
CAATGAACGAGCCTGGTTCGTTGCAAGCTTTAATAAAAATTTAGAGGACAAA
TCTTACAATTCAGAAAACCGTCGCCAGGGTAACCGTTTTCATAACACCCCAC
AAGGTAATAACCACAACAACTCCCAGGGTGTTTTCCAGAGAAATCAAAACA
ATAATGCACCGGGCACTAGTAACCAAACTAGAGGCTCTCAATTTTCTAAAAA
CCAAGGACATAAAGGCCAAACGCAACAGAACAGTGAATCAAATAATCCTCG
TAAAAGCTATTCTCAAAATCTAGGTCCTGAACCAATGGACGTTGATCCCACAT
CACGTTCTAAATTTAGGGGCGAGCAGACAGCTCGCAGTCGTCAATGGTTGAA
CCATACGACCCAAGAGCAGTCACATGACCAGGAGTATAGGGTAAAGTCATCC
TACGAAGCAGCGGAAATCGAGGCAGATAACACGTCCGATTCCGAATCATGTA
ATTTTTTAGGGGAACGTCCCTGCTCCCGTACATAATTCGTACGATAGCGGGGC
GGGAAATCAGGTTACTGTTAGATACAGGAGCCTCCAAAAATTACATAAAACC
TTTATCTGAATTAAAACACTTCAAACCGGTGGAAACACCATTTGAAGTTACAT
CAATCCATGGGCACACAAAAATAAAACAAAAGTGTCTGATCCATCTATTCAAT
GTTAAGTCATACTTTTTCTTGTTAAACAACCTGAACGGATATGACGGAATTGT
TGGACTAGATGTACTAAAAAGGGTCAATGCAAAAATTGATCTAACAAAAAAC
ATCATTGAGCATGATCATGGTACAGAGCAGATTTTTTACTCAAAATGCAGAAA
TGTAAACTTTATTAACATCAATGACGTGGACGTGCCAAACGCCGTCAACGAA
GATTTTAAAAAGATGATCAAAAACAGATCAAAAGCCTTTGCGGACCCAAACG
AGTCCCTCCCCTTTAATATAAATACGGTCGCCACGATCCGCACTGACGGGGAA
CCTGTATATTCAAAACTTTACCCATATCCGATGGGTGTAGCCGATTTCGTCAAT
TCGGAAGTTAAACAACTTCTAGCAGACGGAATAATAAGACCATCCCGGTCGC
CTTACAATAACCCAATTTGGGTTGTTGATAAGAAGGGTTTTGACGAAGAAGG
TCATAGGAAGAAACGTCTCGTTATTGACTTCAGAAAACTGAATCAGAAAACA
ATTGATGACAAGTATCCTATACCATCCATATCGACCATACTGTCGAACATTGGA
AAAGCCCAGTACTTCACGACTCTTGATCTGAAGTCGGGCTTCCATCAAATTG
AGCTCGCGGAGCGCGATCGAGAAAAGACAGCTTTTTCGGTCAACAACGGGA
AGTATGAATTCTGCAGACTTCCCTTTGGTTTAAAAAATGCCCCTAGTATTTTCC
AACGGGCCATAGATGATGTTCTGAGAGATCACATCGGTAAAACTTGCTATGTC

TACGTCGATGACGTAATCATTTTCTCCCAAACAATGGAGAGTCATGTCAACGA
TATAAACACGGTCCTAAAAACTTTGTGCGATGCAGGCATGAGAGTGTCTGTA
GAAAAATCTATGTTCTTTAAGGAGAACGTAGAATATTTGGGATTCATAGTGTC
CCGAGGGGGAATTAAAACTTCGCCTGAAAAGGTTAAGGCTATAAAAGAATTT
CAACCTCCATCAACACTGTTCAGTCTCAGGTCATTTCTGGGACTGGCCAGTTA
TTATAGATGTTTCATAAAGGGGTTTGCTAGCATCGCAAGACCTCTGACGAATA
TTCTAAAAGGTGACAATGGAAAAATTGGTGCAAACCACTCAAAGAAAGTCA
AACTAGAGCTGACCAACGAGCAGCGAAAATCATTTGAAAAACTTAGAAACA
TCCTGGAGTCTGAGGATGTCATGTTGGCATACCCAGATTTCACTCAGCCATTT
GACTTGACCACTGACGCCTCTGGAAGCGGCCTAGGGGCTGTTCTTTCACAGA
AAGGTCGTCCAATCACCATGATCTCTCGTACGTTACGCGGAACAGAGATTTC
AATGGCAACAAACGAACGAGAACTACTAGCAATAGTATGGGCACTCCAAAAT
CTCAGAAGCTATTTATATGGCGTAAAGAAGTTAAACATCTTCACCGACCATCA
ACCACTGACCGGTTCAATGTCGGATAAAAATCCGAATCCCAGGCTCAAAAGG
TGGAAAGCGTTTGTTGATGAACATAACGCACAAGTGTTCTACAAGCCGGGTA
AAGAAAACCACGTAGCCGACGCCTTGTCGCGGCAAAACGTAAATGCTTTAG
ATAACGATACACACTCCGACATCGCTACTATCCACAGCGAGGAGTCGTTGAC
CTACACTATAGAGTCAACGGAAAAACCTGTAAACTGTTTCAGGAACCAAATA
GTGATCGAACAGGCAGATTTCCCGTCTATAAAGTCAATCATCCTTTTTAAATC
AAAAACGCGTCACATTGTGCGATTTACAGACCGCACAACACTGCTTCGCTCA
CTAGAAGATTTAGTAAAAATAGATGTCGTCAATGCAATCCACTGCGAGCTACC
TATACTCGCCTTCATTCAGCACGATTTAATCGTCGCTTTTCCCTCGACGACATT
TAGACATTGTAAAAGCTTGGTCACCGATATAACAAACAGTACCGAGCAAAGG
GAAATTATGACCACGGAACACAATAGGGCCCATAGAGCAGGACAGGAAAAC
GTAAAACAGGTCCTTCGTGACTACTTTTTTCCCAAAATGGGCCAGCTAGCGG
CAGAAATCACCGCAAATTGCAGAACATGTTCCAAAGCAAAATACAATCGTCA
CCCAGTGCAACAAACCATAGCGGAAACACCAATTCCCGGTTATACTGGGGAA
ATTATCCACATAGATATATTTTCAACTGATCAAAAGCATTTTCTAACTTGTATCG
ACAAATTTTCAAAATTCGCTATAGTCCAACCAATCGATTCAAGAGCAATCGTA
GATATCAAAACTCCGATACTACAACTAATAAATCTGTTCCCTAAAATAAAAAC
AGTTTATTGCGACAATGAAAGGTCTATCAACTCACAAACCATACGAACCATCC
TAGAAAATAGGTATGGTATACAGGTCTCAAATGCGCCCCCCTTGCACAGCAC
GTCTAATGGCCAAGTTGAGAGATTTCATAGCACCCTAGCAGAAATCGCACGG
TGCATCAAGATAGATCAAAACATAACCGAGACGACCGACCTCATTCTATTTGC
AACAATAGAATATAACAGAACTGTCCACTCGGTTACAAATAAAAAGCCTCAT
GAAATAGTTCACGCTATTCCACCAGATTTTGCGGGCACAATAAGAGACAAGA
TCAAAGAGGCTCAAGAGAAAACACTTAGGTACTCAAATGAACACAAAGACA
ATAAGCAGTACCAAGTAGGCGAAAAAGTCTGGTTAAAAACCAACAGACGCC
TGGGTACCAAATTAACGCCACTCTGCTCAGAAGAGGTCATTGAGGCTGATCT
GGGCACGACAGTGCTTATTAAAGGGAGGGTGGTCCATAAGGACAACCTTCGT
TAAGAGGGTGCCCAATTTAACTCTCTATATTTATATTTTTCGCACTTAGGTTA
GGAAACTTGCTACTCCCGTTAGTCCTACTGACAACTCCCTCTTGGACCATAAA
ACTAAACGAATACTCACACGCTAACCACATACCAATAATGGACGGAGATATTA

CTTTATGGGACGAGTACGGTTACATGGGACATACAACGAACGTTACATCGTAC
GAGACTTACGCAGACGAGACGAAACACGCAATGGACTTCTTCGTGAAGGAG
CACATGAGACGGGTACTTGAAACGGACTTGGAACGAATAGAGACTCTTCTGG
ACACACTAAAGGTACGTCACCGACATGCCCGTAGTCTTAATTTCATAGGGACT
GCTTTAAAAGCAATAGCGGGGACACCTGACTCTGACGACTGGGACCAGGTG
AGGCTTAGACAGGGACAGCTAACGGACTCGGTAAATGGACAGATAGAAATA
AACGACAAAATACAATTGCAATTAAACACACTGACCTCATCCATGAACTCTAT
TTTAAAATCGGACAACTTAGACACAGAACATTTGTACGAGACAATTTTGGCA
AAAAACCGTGTTGTAATTCAAGAGCTTGAAAATTTAATACTTGCAATCACCCT
TTCCAAATTAAACGTAATAAGTCCAATAATCCTGAATGACGTTGACGTAAAGG
AGATTGAAAGTAAACATCCCACAAACGTTAGCGTGTCAGATATTTTAGAGGTA
GCTAGCATAAAAACTTTTCAAAATCGAGACCTATTGTACTTTTTAATAAAATTT
CCGAAGCCTTTGTTAACTTGTAGAAAAATAAGAATATTCCCAGTACAGCATGA
AAATAGAATCTTAGATTTCGAGGACGGTAGCACGGTCGCGGATTGCGGTACG
GAAACCTTCGCGGTCAAGGACTGCAATGTATCAGCGGGCACCACCTTCTGCA
GGAGATCGAAAGCGCCAACTTGCGCACAACAACTCATCTCTGGCATGGTCGC
CCATTGCAACACCCAGCCTGGACACTTGGACCCAGTCACCATGATCGACGAG
GGAATGCTCATCACCAACGATGTAACGATAAATATCACCGACGAAAAGGGAA
TAAGCCGGATGATATCAGGAACTTACCTGGTATCATATACCGAAAAAATTAAA
ATAAACGGCACCCTTTACGTCAACAATATCGGAACATCAAAGAAGAAAGCCG
CAGTTTCAGCCATGGCTCAAGTAAACGTTCTGAGGCACCTAGAGCGCCTTAG
TCTGTCATCAATTCACGGAATGAGCGTTAAAAATCTGCAGCACATCAACCAC
CTCCAATCCCGACTGCCATCAGGCAACACCTGGATCTTTTGCTCTGTCTCCTC
CACAGCCTCAATAACCCTGGTCATCATCTTTCTTATCTACCGTCTGAAAGCCA
AACGTCAACAACCATCTAAGACCATTGAATCCGGGGACGACTTCATCTTAAG
ACAAGGAGG

>Gypsy1-LTR_Gypsy#LTR/Gypsy1-LTR_Gypsy

TAGTTAACTAAGTTAACCGGACCGATCGTCCGCCACCAGCACCGGTCAAATT
GCTGACCAAGCATTTGGCCGGAAGCTCGCGCATAGCCGGCAGAAGCTCTGC
GCATTGGCAGAGGCCGCTATGAGTTTTTCCCTTTGTTAGCTTATAGTCAGTTC
GATTTGGATATTCAATAAAGAGCGCATCGCTCGTTAAACCAACTCCGGCAACT
GCCGTTATCATTATAATTTACCATAGCTATCTGAGTCCCTAGGCCGGAGCCAAA
AGGAAGACAGTCATCTTCCCAACGTAATCATCGTAACTCTTCAAGTGGATTTA
CCACCCCCTACCCCCGTACGAGGGACACCCATCGGCCAACCGGCAACTTACC
GGTGACTGCGGCGAGGGATCCCCCCTGCGGCGCCCTCACA

>Gypsy1-I_Gypsy#LTR/Gypsy1-I_Gypsy

CCCAACTCCAGGAACCCACACCACTACCCTGAATCATGTAAGTGGGATTCTC
AACTTAAACACTACTCCAAACTGCGTCTAGAATTTTAAAATATTTAAAATATTT
GTGCGAGTTACATAAATAAATTAAGAAAATCGGCATTTCCACTACAATAAACG
TTTATATGTGTTGCGAATTAAAATTATGTTACTGTTATGAAGTTTAAATATCGAA
TTTTGATTTGTGGTTGACTTTGCAATCCATATTGTGTGCATTTCGTTCCGCCTT

CGCACATCCGCGGGACACTGTCGTTTCATTCGAAATTTAATTCGCTACATTGG
CTTCACAGCCCTTTCAAGCTTTGTTGTTTTTGACCCACTCCACTTCGCTACCC
GATACTGGCGCATGCATTGCTGTGACAATTTTGTGCCTTTTATTTATCTCTTTG
TCTTTGCTGTGGCAATTTTTGTCTTTGTATATTTGTTTGCCTTATTGGAGACCC
GCTCCCCGCAGGCCCTTCACCTTATCGTTACTTAGCTAGGCAGTGGCTCTGCT
CGTTGAGTCTTCGTCCAATGCCTTCAAAGCGGCGACTCAGCCCCCGCGACCC
CCTTGCCGTACTGTTTGGCCCCACGGGCACAACGGCCTGAGTATTCACATAC
ATAGCTACCCATGAGCGAGTCATTCCGACAATATAGGAATTCTAAAAAGTGCG
CTAGCGACTCAGAGTCCGAAAGCGACGATTCGACAGAAAACTCTGTACGTA
AAAACACCCCAACTAACGCATTCACTGCATATAAAATGTCTCTCGAAACGGA
ACAAATTAAAGCTCTCATAAGGGCATTACAAGAGCAAGCCTTAGAGAGTCAA
CGCAGGGAGGCTGACTTGCGTAAAACAATTCAAGATCTGGCCGGCCAGGTC
GCGGCCATACAGATTGCCCCTGCCCGGGCAGAAGCTCCCCCAATCAAGGTTT
ACAGACCAGTAGAAATCACCGGACTGGTCCCTTGTGGGGAAACATTGGATGC
CGTTAAATGTCTTCCAGACTTTATGGGGACACAGGAGACATACGTCTCCTGGC
GGCAAGCGGCAAATGCCGCTTACCATATGTTCAGGAAATATGAGGATAGTTCG
CGGCACTATCAAGCTGTGGTCATCATCAGGAGCAAAGTTAAAGGCCCTGCTG
ATGCAGTTCTGTCGTCCTTTGGGACTATACTGAATTTCGATGCGATCATAAGTC
GCCTCGATTTCACGTATAGTGACAAACGCCCGATACACGTTATCGAGCAGGA
GCTAGGCACCCTCAGACAGGGAAGCCTGACGCTCCTCCAGTATTATGATGAG
GTCGAGAAAAAACTCACCTTACTCACCAATAAGGCGACTATGTCTTATGAAG
CGTCGGCAGCAACGGTGCTGTGTGAGAAGTTCCGAGATGATGCTTTGAGAGT
TTTTGTCTCGGGGCTCAGGCGCAACCTCACAGACGTGCTATTCGCGGCAAAG
CCTAAGGACATGCCGTCAGCGCTCGCCCTGGCGCAAGAAGTAGAGTCCAATC
ATGAGCGGTACACTTTTGCAACTTCATTTGCACGAAGCCAAGAGGATAGGGA
CCACAAGCAATATCCCAAAGTGCAGGAGCGCCAACGGGCCCCCCCACAAGC
CGGCTCGCAGGGAAGTGCTGGGAAGAACCCGCACTTTACTAAGCAGCATAG
AGCACAGGTGCACTCCGCTCCACGTAGCGACCGAATGGCCCGAGAGAACAC
GCCAGAACCCATGGACGTTGACCCGTCGTTGTCCAGGATGCAGCCATCTCAC
GCCCCGGCTTACCCGAAATCGAAGCCGGCCGCGTCTGGCCGTTCGGTCCCAC
CAAAAAGGCAAAGGGTCAACCATGTTGCCCAGGCCTCTGATGATTCGGACA
AGGTTTATAACACCGCAGCCTCCAGTGCAGCAGTTAAAGTCGACGACGATTC
CATCCTAGAGTATGACTCGGATACCATTAATTTTTTAGGGGAAAGTCCCTGCT
ACCCGTCATCAGACGAAGAGTAGCGGGGATCGACATGAAACTACTGGTTGAT
ACGGGCGCGGCAAAAAATTTATCCGACCATTTAAGGGGTTGAAAGGCGTCC
GCCCGGTGCAGTTCCCATTTACAATCCATTCGATTCATGGTGTGACTACAATA
ACTAAGAAATGTTTTGTGTCCATTTTTAATCTTAAAGCTACCTTTTTTTTATTAC
CAGACTTGACCTCCTTTGACGCGATCGTTGGCCTAGACCTGTTAAAACAGGC
CGGCGCGTCGCTTTGCCTAGCTTCCGGCAAGCTCAAATGGGGCTCCGGAGCA
GAGCAAATTGACTTTCATACTTGCCCCGATGTCAATTTCACCAAAGTAGATTG
CTCGGACGCACCGCCCTTAATTAAGGGTGCTTTTTTAAAAATGCTCGGGAATA
GGAAAAAAGCTTTTGCTGATCCTAATGAGGCTCTTCCTTACAATACGTCGGTG
GTAGCCACCATCCGGACGGTTGATGAGGAGCCCATTTATGCCAAGTTATACCC

ATATCCCATGGGAGCAGCTGACTTCGTCAACGGCGAAATTCAGGAACTGCTT
AAAAATGGCATAATCCAAAAGTCAAAGTCCCCCTACAATAACCCAATATGGG
TCGTAGACAAAAAGGGCACTGACGATGCGGGCAATAAAAAAATGCGCTTGG
TGCTGGACTTTCGAAAACTTAACGAAAGGACGGTACCAGACAGATACCCCAT
GCCAAATATCTCTATGATATTGGGGAATCTCGGCAAGGCCAAATACTTCACGA
CCCTCGATCTGAAGTCTGGCTACCACCAAATCACGCTCGCAGAACGCGACCG
TGAAAAGACATCGTTCGCAGTAAACGGAGGGAAGTATGAGTTCCGAAGGCT
GCCATTCGGACTCAGGAATGCTGCAAGCATCTTCCAAAGAACAATTGACGAT
ATTCTGCGAGAGCAGATCGGAAAGTTCTGCTACGTTTACGTCGATGACGTCAT
CATCTTTTCGGAAGATGAAAACGCCCATGTCAAGCATGTAGATTGGGTTCTGA
AGAGCCTGTACGATGCTAACATGAGAATATCGGCAGAAAAGTCACGTTTTTTT
AAGAAAAGCGTGAGCTTCCTGGGGTTCATCGTCACCAACAATGGGGCGGCG
ACTGACCCAGAAAAGGTTAAGGCCATAAAGGAATTTCCGGAACCCAAAAAC
GTATTTGAGGTAAGGTCATTCTTGGGCTTAGCCAGCTATTATCGTTGCTTTATC
AAAGACTTCGCATCAATAGCTAGGCCCATTTCAGACATATTGAAGGGCGAGA
ACGGTAGTGTTAGCCGACACAGGTCCAGGAGTATCCAGGTAGAATTTTCCGA
AGCGCAACAACGTGCCTTCGAAAAGCTACGCAATATCCTGGCGTCTGAGGAC
GTCATCCTGAGATACCCTGATTACAAAAAAGCGTTTGATCTAACGACAGACG
CTTCGGCCTACGGCATTGGCGCAGTGCTGTCCCAAGAGGGACGTCCCATTAC
AATGATCTCAAGGACATTGTCTGACAGAGAGGTTAACTATGCTACCAACGAA
AGGGAGCTGTTAGCCATAGTCTGGGCACTGGCTAAGTTGCGGCACTACCTGT
ATGCGGTTAAAGAGATAAACATCTTTACCGATCACCAACCTCTGACGTTCGCG
GTATCGGAGTCCAATCCGAACGCCAAAATTAAGAGATGGAAAGCACGCATCG
ACGAGTCCGGCGCACGAATTTTTTACAAGCCTGGGAAAAACAACCTCGTTGC
AGATGCCCTCTCGAGACAACAACTCAACGTTGTTGAAGAGCAAGAACCGGA
GTCGTGCGCGGCCACGATTCACAGCGAACTTTCGCTTACGCACACGATCGAG
TCCACGGACAAACCCGTGAATTGCTTCCAGAACCAGATAATTTTGGAAGAGG
CGCGCTCCCATTGGAAACGCACTTTTATATTATTTGGGAATAAGAGGCGGCAC
TCGATCAATTTCTCGTGCAAACAAGCTTTGCTGGAGGAACTCGCCAACATCA
TTATACCTAATGGTGTAAACGCCTTCCACTGTGATCTTCACACGCTGGCGCTA
ATCCAGGACGACATAGTTCGACAATTTCCAGCCACGAAATTCTGGCATTGTAA
GAATAGGGTCACCGACATCTTCGCGATGCAGGAGAGAAAAGAAATCCTCACC
GTCGAGCACAACAGAGCTCACAGGTCGGCCCAAGAAAACGTGAAACAAGT
ACTCTCCGAGTACTACTTCCCGAAAATGACCAAATTGGCGAGCGAAATTGCA
GCCAATTGCAAAACTTGCGCAAAGGCGAAGTATGACAGACATCCGAAGAAG
CAGGAGCTCGGTGAGACACCAGTCCCGACCCACGTAGGAGAAATATTGCAC
ATCGATATTTTCTCAACGGATAAAAAATACTTTCTCACCTGTGTTGACAAGTTT
TCTAAATTCGCCATGGTACAGCCGATTCTGTCTAGAACCATAGAGGATTTGAA
AGCACCCCTTTTACAACTTATGAATGTTTTCCCCAAAGCCAAAACCATCTACT
GCGACAATGAACCATCATTGAAATCGCAGACAATAGTGGCTATGCTGGAAAA
CCATTTTGGCGTCAGCATTTCGAATGCACCGCCCCTACATAGCGTCTCAAACG
GACAGGTGGAACGATTCCACAGCACGTTAATTGAGCTCGCCAGATGCCTAAA
AATCGACAAAGGCATAAGTGACACAGTGGAATTGGTCTTGCTGGCCACAGCC

AGATATAACAAGTCCATCCACTCTGTCATCAATAAAAAACCGGCCGAAGTCAT
GCGGGCAGATCCGGACGATCCACATACCGATGTCCAAGAAAAAATCAAAAA
CGCCCAGATTTTGACAAGAAAACGAGAGAACGCTTCTCGGCAGAACAGAGT
GTTCCAGGTCGGCGACAAAGTCCTAGTAAAGTCAAACAGACGATTAGGCAA
CAAACTTACTCCTTTATGTGAGGAGAAGACCATCGAGGCAGACTTGGGGACC
ACAGTCCTTATTAAAGGGAGGGTGGTCCATAAAGACAACCTCAAGTGACCCA
AGCAGAGCCTAGCCGCGGCTCCCTCGGAGGCACACTTTTATTCTTCCAATTTG
TAGCCACTCGGCATAAGTTTTTCATTGTTTTATAGCCGCTTGGCATAAGTTT
TTTATTTTTAGCCACTTGGCATATTTTTTATATATTTTCGCTATTATTGGTGGTGG
GCAACTCCATTCCGAACAAGTAATAATTTATCACACACGTTACAGGTCGCTCC
CAACCCTTCTTCTTTGTTTCCTGGCCACGACATCGGCCCACATTACTGACTAT
TCCCGAGCGAATTACATTCCCGTCATTGACGGTAAAGTCTTAGTCTGGGAGG
AATTCGCCTATGTCAGACACTCGGCTAACCTCTCCGAGTATAGGCGGGTAATT
GACGAAACCGACAGCATGCTCGATATGTTCCCCCAGTCCCATATGAAGAAGC
TCCTGAGCGTTGATATCGCTCACCTCCGTGACATGCTTGATTCTTTGAGCATC
CATCACAGAGTGGCAAGGAGCCTAGACTTCTTGGGAACTGCGTTAAAGGTTG
TCGCAGGGACACCTGACGCGGAAGACTTCGAGAAAGTCAAGTTCACTGAAG
CGCGGCTTGTTGATGCACACAATAGCCAAATCGAAATAAACACCAAAACACA
AATTCGAATTAACGAACTCACTGATACCATAAATAAACTTTTAAAAATTTCCA
AAAGCGCTCAGATTGATACAGGTCACCTGTATGAAACGCTTTCTACTCGCAA
CAGAATCATTGTAATGGAATTGCAAAACTTAATGCTCACTATAACCCTCGCCA
AAATTAACGTAGTGAGTCCAAACTTCTTGGACCACGCAGATCTGGAGAGTGT
TTGGGGCGAGGAGCCCACCAACACCCCCATAAGGGAGATTTTGTCCGTTGCG
TCTGTAAAAGTCCTACAATCCCTTACCATCTTACACTTTATTATTAAATTCCCC
AAGATTATCATGGCGTGCAACAAAGTCACTATCCTTCCAGTGGTACACCACG
ATACGGTGTTAAGGCTGAAAGATAATGTGGTAGCAGAGTGCAACAGAGAAAT
ACGCACAGTAAAGAATTGCTCCATAACACCAGGGGCAACATTTTGCCAGTTA
TCTTCAGTGAGCTCGTGTGCGCAGGAGCTCCACGCTGGGGTCGTAGCACATT
GCGACGCACAGCAGAGTGATCTACATCCGATCACCTACGTCGACGAAGGAAT
AATCGTCATCAATGACAGACCAGCACTCGTGCGTGTGGACAATGGAACGGCC
ATCCACATTAGAGGCACGCACCTCATAACATTCATTGAGAGTGCCATGGTCAA
CGAGACCGTCTTCTTTAATCATGACATGGTCCAGAATAGGGCGCCGGGAGTG
GCTAATTCCCCAGTCCTTAATATCTCGATGAAACACGAGGTCCTGAGCCTCCC
ATACCTTCACCGTTTAAGTGAAAAGAACTTGGAGCAAATCAGGAACTTCGAG
AAGGACGTCGACGGATACCGACTAAGTCAGATAGCGCTAGTTGCGGGAGCA
ATTTTCTGCGCTCTTATCTGCATCGGTTTAACCTGGCAGCGAACCACTAGGGC
CAAGAAATCTACAGCCCAACTGAAGGAAGTTCTCGCCCAAATAGGGTCAGC
CGAGGGCGGCCTTAATCTTGAGGAGGGAA

>Gypsy-5_el-LTRGypsy#LTR/Gypsy-5_el-LTRGypsy

TGTGGGAGTCGAGCCAAATGGCAACCTTATATGTGCTACCGCACTTATTTGGT
AACGAACCATTTTAGCCAAATTCAAATCCTGGCAAAATTGAATATTGCCCTAT
AAAAATACCACAAAATTCCCGAGTAGGATTATAGCAAGCTACGGTCACATATG

ATTTGCATACTTCCGTTCTCTTTTCGTGAAGGCGTGATCGAGTGCAGTTTAAC
TTTATGCAAAATCCATGATTAAAAATCCACGAGCATCGTGTGGTTTGGATAAT
AGTTTTTCAAGGCAAATACTGCCAAGTGATAGTTTCCTTTAACTGCGTCGCTG
CCGGTCCCACATTTGGAGATAGAAGTCTTAGTGGCTGCGTCGCCAAGGCGGT
CTGTATTGTTTTTCGCCAAACTTTTGACCACGCGTGAAACTTACTGTGGCTTC
ATCGATCGCAGAAAGATTTACAACTGCTTGCATCAGGAGCACCCACCGCGTG
CAGCGGAAAGCTGGGCTTGTCCTCTTCTGCTGCCGACAGATCGCAGGTCAAC
CCCCCCCCCCCCCAGAGTTGGCGTTCTTTTTTTTCTACTCTTCATCGCCGCA
CGCAGGAAGCAGATCGCCGTGCATCCATTTTATGCACAAGTCAACTTGGTGT
CTTCTTCATCCGTGCGCATTCCAAGAATATTTGTGCACAACACGAGCCCCAGG
CTGCTGGCCTTGTCGCTGCGTATGCTGCGAACCTGAAGCAGCCGCAACACGA
ATGATCGTGTCCAGTCTTAAGTATCTTTAGAATAGTTTTTCATATTTGTGTTAAT
TTTACCTAAAAATATTTACTTATAAATTTCTACGATTCTAATTTAGCTTAACTTG
TGGGAGATCTGACAAGTATATATATGTGATTTTCCCGATTTATATTAATACCTAC
AGCAAATCATCAACAGTCATCAGGCCTGCTGACATCGATGGAAAGTGCGTGA
GTACGGAACAAAAATAATAAAAACACACAAGCATATATATAAAAAGTCAGAT
ATTCCTAAATAGTGTGCTTAAAATACCTTGCCTTAATTAATCATATATCTAGATC
TCTTAATTTAAAGAAGTTTAAAGTTTAAATATACATTAATCATACATAATTCTAA
CGTAAAGTAAGCCAAACAAACAAAAGCATTAATGTAAACTTAAATACCAGTA
TTTGCATTCCTATCTTGATTATTTTTCCTTTATTTTATCGTTGATAAGCAAAATTA
CTCAATAAGAGATTTCCTTAACGTAAATAACGTAATTTTTAGTTTAAGGATTAG
GTTTAGGAAAATGTATCGGAATATTATCTTTATCTTCAATTATATGGGGTCCAA
AATGAACTAATCTGATGTCTTGTTGGGCACCAAAGGGTATGTAAAGCAGTGG
TTGTATTAGGGTGGAGTCCGAATGGATTTCACAGGTAGGGTGGGTATAGAATA
AGGCCAATAACATCTGGGCCTCATCTTCAATAGGTACACTTTCTTGTCCGCTT
CTTAACCGTTTGGTTATCGTTTTTTTTTTTGAATCTACCGTATCTCTGTCTACAG
TATTGTAGCTGTGACACTCTTGTTCATCTCAACACAAAACCCACGCCCATTTT
CCCTGAGCATGCCGGTAAAAATAGTAGACAGAGTGTGTTGGGGTGGACAAC
GCTCTATGAGTGTGTTCGTTACA

>Gypsy-5_el-IGypsy#LTR/Gypsy-5_el-IGypsy

GAGTTATAATTGGCGCCCAAACGTGGGACAGCCCGATGCCTGATTAGGCACT
TGTCGTTTGACAACCGAGCTTCCCGTTAATAAACAATCCATCCACTCTTAGTT
TCAGTTAGGTATTTCATATTCCGTCCTGGAATTTATGATATCTGGAGAGTTCCG
GAGCCATACCGGAGGAAATGTTATGCTTTTATATGTTTTTTTTTCTATCAGTTC
GGTAATAATCGAACATTATTGTTAAAAAAAAGAACATTCTAATTTGAGTAATT
GGTTCTTAGGAATTATAGTTCTGATTTAAAGTCGGTTCTATACCAGGATTTCAC
TATTCAAAAATTACCTCCTTATCATTTAGGATCGAGATTTTTCTAGCTGGATAG
ATTCGGCTAGTTACTCTTCTAAGTGGTAAGAGTTCATAAATAAATAAATGACAT
TTATTTTACAACTCTGTATGCACACCCCGTTAGCTTTTCGTACCTATTGAATTAT
ACAACCTACTGCTTTGCTACTCAGGGTAAGACCCGCAGGTATTATTTTTGGTC
GCTTAACTTTTATCTTTTTGTCGCTTAGCGTTGATCGTTGGATCTTAGCGCGTA
CTCATTATCTTTTTTCGCTTATCTTTGATCGTTGGATCTTACCGCCTATTTTTCTT

GTTCTAACGAGAGGAATTGTTAGACTTTATATATTCTTCTAGAAGCTTCGAAA
ATGATGATGCGAAGTAATGAAAATTTAGTTGACGTAGATACGGATGCGTCGGT
GACATGCACCATCTGCAACGGGCCAGTACGTAATCTGGATTTGGTTGAAACC
CGTTGTAAACATTCTTTTCACAAACATTGCCTTGACAATTACCTTAAGAACCA
TGACAAATGCCCCATATGTGGCCAGCCATGTTCTCAATCCGAGTTAGGATCTC
ATTTGCCTTTGTCAACTAAATCTCAAGGTAACAGGATGATGACCCGTTCAGGT
TCTCGGAATACAGCCAAACAACATGAGAAGTCTGATCCAAATTCCCAACAGG
AAATTTGTAGCGGGGATAGTTCAGATTCCAGAGTTGTGACAGAAGACCGTGT
TCAGCAAATAGTCCAAAGTTCAATGCAGGCCTTTCAAGCAAGCATGCTACAA
TCAGTGACAGAACAAATCACTATGGCGTTCACTCAATTGAATAAATCCACATT
TGTAAATAATGGACAGAGTGAGCTGCAGCAAGACATACAAAATAATTTCGGT
CGAGACGCTCCCGAAATAAACTCAGAAAGAAATAACAACTCACATGTTAGGA
CTTCACCAGACTTTCGTAGCGATCGAAGTGATCTTTCTTTGGATAGACCTGAT
AGGATTTCCAATATTATATCAAATTGGCGTATTAAATTCAGCGGTTCAGCCAAC
GACATTGCCATTGAGGATTTTATTTACCGCGTAAATTGTCTCACTTCGCAAAG
CCTGAACGGAAACTTCGAACTTCTTTCCCATTTCGCTAATTTACTGTTTGCAG
GACCGGCCTTAGCATTTTATTGGCGTGTTCACAGATCGGTGGATAACATGAAT
TGGAACGTGTTATGCAGGCGTTTAAAGGAAAGATATCAAGATCAGAGATCTG
ATCGTGAAATTAAAATTGCTATGCGTCGCCGTAAACAGGGTAGCACAGAAAA
TTTTGATGATTTTTTGGATGCAATGCTTTCCATTGCAGATTCCCTTAGTGAACC
AATGCAGGACAGTGAGATTACTGTAGAAGTTCGTCATAATCTCAAACCGGAG
ATCAAGCACGAATTACTGCACATAGACACCCCAAACTTAGCAACATTGCGTA
AGGAATGTCATCGTCACGAAGATTTCTTTCGAAGCACCCGGACAAAGCCAAT
CCAACGTCCGAACACAAGCAAGCGTTTTGTTAACGCAATCCTGCATGAAGAT
GATTCCGAAGATCAGTCTGAGGAAGAACAGGATGTCGAGGACGAGATTTGT
GTCGTTAGGACTTCTGAGAAAATTAAGTGCTGGAACTGCGATGAATCAGGTC
ACGGTTACCAGAATTGCCTTAAGACACGCCGTATTTTTTGTTATGGTTGCGGA
ACACCGGAGGTGTATAAACCCAACTGCGCAAAATGTAAATCGACATCGGAAA
ACTCTCAGCAGGGCATTCGTTATGCGAACAAAACGAATGTCCGCCGTTAGCC
TCGAAAATACAAACTGAACCAAATTCTGTGGCAAATGATGTTCATTCCATTCT
GCCCTCAACTCAACCTGACGTAACATTATCCCAAACTTTTAATCCATATCATTT
GCAAGTGCTGGAGTATGCAAATCGTAAATTGCAAATATTTAGGGAAAATTCAC
TTCATAATAATTTGGATTCGTAAACGATCATTCAACCGTTTTACGATTTCATCA
ATTGTTCAAAATAAGAACGATATTCGACCTTTCACACACATTGACATATTTGGT
CAATCGCATTTAGCTCTCCTAGACAGTGGCGCCAACAAAAGTGTTATAGGTG
GTGAGCTAGCACAACAATTAATATCAAGCAAGCCATTTAATAAATTTAAATCG
GTTGTGCGAACTGCTGATGGCCAAACGCAAAATGTTGCAGGCACTATACAAA
TTCCCTTGACTTACAACTCAGTTGATAATAACTTCGAATTCTTAATAGTCCCTT
CCATAAAGCAAAGCGTAATATGCGGAATGGATTTCTGGTATACGTTTGGAATT
TCTATTAAGCAAACAGTTTTGATCAACGAAATCAACTTTGAACCCGAAGAAG
ACTCTACTCGCGTACGATTGTCTGATTCACAAAAATCAAAATTGCAGAAAGTA
ATAGACTTTTTCCCATCGTTCGAGAACGAGGGCTTAGGATTGACTAACCTGAT
AGAGCACAATATCGATACATCCAATGCGAAACCAATCAAACAACGATTTTATC

CACTTTCGCCTGCTAAAGAAAAACTTTTGTGCATAGAAATCGATCGCATGATT
AAGATGGATGTCATTGAAGAGGCACCTTCATCGCCTTGGTCATCTCCAGTTAC
ATTGCATATCAAACCAGGCAAGGTGCGATTTTGTCTCGATGCAAGAAAATTAA
ATGCAGTCACAGTAAAGGACGCATACCCAATCCCAATTATGGATGGACTGCTA
AGTCGTCTTCCACCGGTACATTGCATTTCCAAAATTGACCTCAAAGATGCCTT
TTGGCAGATCTGTTTAGATCAAGAATCCCGTGCCAAGACTGCTTTCACCGTTC
CAAATAGGCCCCTTTATCAGTTCAAGAGAATGCCTTTTGGATTGTCAAACGCT
CCACAAACCATGTGCCGTTTGATGGACCTCGTTATACCCTATCAATTAAAATC
GCATGTATTAGTCTACCTCGACGATTTGCTGGTTTTGTCAAACAATTTCGAAG
ACCATCTTTTGCATTTGTCCGAAGTAGCCACCCAATTGCGTAAAGCTGGATTG
ACAATTAATGTCCAGAAAAGTCAGTTCTGCCTGAAAACAGTAGACTATCTCG
GTTACCTGGTGGGCGAAGGCACACTACAAGTAAATCCGAACAAAATCGCTGC
TGTGAGAGACTTTCCGGTTCCAAAGACCCAAAAACAACTAAGACGATTTCTT
GGTATGACTGGTTGGTACCAGCGATTCATATCTAATTACTCCACAGTCATTTTC
AATCTTACAGAGTTGTTACGTGGCAAATCCTTCAGTTGGAACGATCTTGCTCA
AGAAGCTTTTGATAATATCAAAGACAAGTTATGCTCTGCTCCTTGTCTCATTC
ACCCCAATTATGACAAACCATTTATCCTGCAGTGTGATGCTTCACTACATGGA
GTGGGTGCAGTTCTAGCTCAATGTGATGATTCCGGTTGTGAACGTCCGATAGC
ATTCATGTCTAAGAAGCTGAATAAAGCCCAACGTAACTATACAGTTACAGAAC
TCGAATGCATGGCTGTAGTTCTGGCAATTAAGAAGTTCAGAATGTACATCGAC
GGTCATAGCTTCAAAGTAGTCACCGACCACTCAAGTCTTCGATGGCTAATGA
ACCAATCAGATTTAAGCGGGAGATTGGCAAGATGGGCTATCAAACTTCAGGG
TTATTCCTTCGAAATCGAGCATCGCAAGGGAACAGAAAATGTGGTTGCGGAC
GCTTTGTCTCGATCGTTCGAGGATGTTGACGATATTGCTGCAATAGACCTTGA
AGTCCATTCCGAAATTGATTTATCTTCCAGTGCATTTCAATCGGAAAAGTACT
CTGCTCTCAGAGATAAATTAGTGTCTCAAAAATTACCTGATTTTCAAGTCAGT
GATGACTATATTTATCATCGGGCAACATTCCCCAATTGTACTGACGTTTCACCA
GACGATTGCTGGAAATTGCTTGTACCTGAGTCGTTGCGTCATAGCGTAATGAG
TTCGGCTCATGATCAACCAACATCTGCACATTGTGGAATGGCTAAATGCTTAG
AGCGTATTAGACGGCGTTTTTACTGGCCAAACATGGTTATCAACGTTCGTGAT
TACATTCGAAATTGCGAGACGTGTCAGACCACTAAATATCCCAATCGTTCTTT
AAAACCACCAATGGGAGCACAAGTGCAAAGCGATACAATTTTTCAAAGACTT
TATCTTGATTTTATCGGCCCATTTCCCAGATCGAAATCCGGCAACATTGGTATA
CTGATTATTCTGGATCACTTTTCCAAATTTACCTTTTTAAAAGCTGTGAAAAA
GTTCAACACTAAAGTGATCATCAGTATATTGCGGGATGAAATATTTTCATGCTT
TGGTGTACCCGAAACAGTCGTGAGTGACAACGGAACTCAATTTAAAAGCCG
TGATTTCTCTGACTTTCTTTCGAAATATGGCGTTCGTCATATGTTTACTGGTGC
ATATGCTCCACAATCCAACGGAGCTGAGAGAGTTAACCGTTCTATAAACGCT
GCTTTAAGAGCTTACATTCGCTCTGACCATCGCGAATGGGACGTGTTCCTCAG
TAGCATCAACTGTTCTCTTCGTAACTCGATTCACCAATCTATTGGTATATCGCC
TTATCAGGTTGTTTTCGGTAAACACATGATATCCCACGGTAATGACTACAAAC
TGTTGCGCAAACTTAATTTACTCACAGAAGGTGACGTAAAATTATCAAGAAC
TGACGAATTTCAAAGAATTCGCTCCAACATTGCCAAACATTTGAATAAGGCTT

ATGAGACAAACCAAAAGTCTTACAATCTCCGAGCACGACCCCGATCTTTTGA
GGTTGGTCAAGAAGTTGTTAAAAGAAATTTCGTTCCAAGTAATGCAGCGAAT
AATTTTAACGCGAAACTCGCTCCTGTCGGTGTTAAGGCCCGAGTCAAAGAAA
GGATTGGTCAATCAATATATCTCTTAGAGGACATGAACGGCAAGGAAATTGGT
AGATTTCATGCCAAGGACATTTGGTAATTGCTTTTGACTCCTTTTTCAGTTTTT
ATTGTTAGTCACAGGGACTAAAAATCAAATCTGTGGT

>GTWIN_LTRGypsy#LTR/GTWIN_LTRGypsy

AGTTAACAACTAAGAGCACACACTATTAAGAGCACACATATAACAAATTAATT
AATATAAACAATGCTGACGCGCCCAAACTGAGTTCAGCGCTCTGCGCCACGA
ACGGTCAGCAACAGCAATCGGACACCCCTTATCCGGGGTACCGAGCTATGTT
GCATAAATGCTGAGTCGGCTTGCCGACCATGGCTTTATGGCGTGATGCATTGG
AGCCACAGTAGATTTACATTTTCATATTCTTTTGTATTCAGTCTTAAGCCAGAG
CTTTAATAAAGAGCAGCTATTCACTCCGGCTCGCAGCCGTAAAATATCATTTT
ATTATTTGAACATTCTCGCTGAGCCAAAAGGCCAGCGATCGACAAAGAGGGC
AAAGTCACGCCCTCCGACATAAACTTCATAAGAAGATATTATCCTATTGTCCC
TGGTAGCAGCCAGCACCAGATCTCCACCGCCTGGAGACCACCGGACACATCC
CGAAGGAGGAACACATTAATT

>GTWIN_IGypsy#LTR/GTWIN_IGypsy

GGCGCCCAACGTGCATTTGAACCTGCACCTAGTTATATCTTAACATAACACTT
AGTTGAGAATACAAGCCATGTAAGTTTGATTAATGAAGTCACTCGGCAACCTT
AAGAATTTAGTGTGGAATATTAAATAAATAAAAAAAGCTAACAGCCATTTCTT
TGCATGCTTTTAGGGAGCATTATTTCCACAGCATACTTTTACCAAGTTACAAT
TCTGTAGCATACCCCAAGTTACCCGCTCGTTTCAATTGCATACTTTTACCAAG
CGTAAATTCTACAGCATACCCTAGGTTACTTTGAACTACTGCCCAAACGAATT
AATAAGAAATAATTATTTCTTCTGCGCATACTTTTTCCTTGTTTGTTTGTAGGG
ATTTCCTTTTCCTTTGTGTCGCCTTCTATTTCTCGCGTGTGCAAATATCGCGCC
TCGTATGGCCGATCAACCGTGTTCGGAAAGAATTAAATTGCGTGGTGTCAAC
CAATAAATGAGTTGGACGGCATATAGAGACATAAAAGTCGAGCGCAATAGTG
ACGGCGAGTTTGATTTAGAGCCCGTAGGTTTTTGTCCGAATACTACAAATAGC
ACTGCAGCCATGGACGCCGCACAGCTTCAAGCTATTATAGCGGGAGCCGTTA
ACCAGGCTTTGACTGAGCAGGAAAACAGACTTAAGAGGGATTTCCAGGCGC
AATTAGATGAGGTAAAACAACAGATGCAACACTTGCGTGTGGAAGCACCAC
AAGTAGAGACATACCAAAAAATAACCGCAGGCCCGGAAGTACGGTGCGACA
TCAAGCTTGACATTGTGAAGACAATGCCAGACTTTTCGGGAGAGCAGGATGA
CTATGTGTCATGGCGGCAGTCAGCCGTGGACGCATACGAGATCTTCAAACGA
TATAATGGTAGCGAGGCGCATTACCAGGCCGTTTCGATTATCAGAAACAAAAT
TAGGGGGCCAGCAAGAGGGCTGCTAGTTGCCCATAACACAGTCCTAAATTTC
GATGCCATTATTGCTCGACTAGACTGCACCTACGCGGACAAAACGTCGCTGC
GTGTACTTAGGCAAGGACTCGACACAGTTAGGCAAGGGGAGCTTAGCCTCAT
GCAATATTACGACGATGTAGAGAAACGGTTGACATTAATAACAAACAAGATA
GTTATGACTCACGAACCGGACAGTGCTATTCTTTTTAATAATGAAGTTAGAGA

GGATGCTCTCCACGCGTTCATTGCCGGTCTAAGGAAGCCCTTGAGGGCCATA
GTCTTGCCGGCGCAACCCAAGGATTTGCCCTCAGCTTTAGCTTTGGCTAGGG
AATCCGAGGCTACCATAGAACGCACTAACTTTGCCGCTACATACGCAAAGGC
TCTAGAGGACAAGGCAATCACCTATGAGCACCGAAAAGATCGGAACCACTC
AAGGGAACCGCATGGGAGATATAGCAGGGCCGAGGACAGCCAAGGGAAAA
ACCCTCATTTTTACAAGAAACAGGGTAGACAAAATAATTCTGCCCAGAATAAT
AACAGCTACCGGAATCAGTCACCTGAGCCTATGGAGTTAGGCTCCACTGTAA
CCCAACATAGGCAACCGACCTCCTTCGGAAGCGGTCAGCCTGCTAGCGTCAA
GGCCGAGGGTGCTCGTGGCCAGAAGAGATTCGGCTCCGCACGCATGACTGG
TCAAAGGCGTCAGAGGGTGCAGAACACCATCCAACAGGCTGACGATAAAAA
CGACAGCGAATATGAGGGCGCGGCCGCAGCGGCCGTTACCGAGATAGATGAC
GAGTCTGATGAAAATGATCAGATCAATTTTTTAGGGAGCGCTCCCGACTGCC
GTTCATTGAACGCCAGTTCCATGGGAGGAAATTGAGAATTTTAATAGACACA
GGGGCGGCGAAAAACTACATCAAGCCCGTAAAGGAGCTAAAAAATGTGGTG
CCGGTCAATTCCCCTTTCACAGTTAGTTCCATCCACGGTTCTAACACAGTCAA
ACAGAAATGTTTAGTTAACATTTTCGGGAAAACAGCGGATTTTTTCCTTTTGG
ACACGTTAACACTTTTCGATGGCATTATTGGATTTGATTTTCTAGCTCAAGTTG
GAGCAAAGCTAGACACAGAAAAAGGCACAATCAACTATGGTTCTGTTGTTG
AAGAGCTACAGCATCATAGTTGCGACGAGGTAAATTTCACTAACGTGAATGA
CGCTACGGTGCCAGAATCTGTAAAAACTGAGTTTACAGCCATGATCGCAAAC
AGATCTAAGGCATTTTCAGCCTCTAACGAGGCACTGCCGTTTAATACTTCTGT
TGTTGCCACAATTCGCACAAGCGATGATAAGCCAGTATATTCCAAATTATACC
CGCACCCCATGGGTGTCTCTGAATTTGTTAAGCGAGAAATCGCAGACTTACT
GCAGAAAGGCATTATTAGAACCTCAAGGTCGCCGTACAACAATCCAACATGG
GTTGTTGACAAAAAAGGCCATGACGAACAGGGGAACAAAAATAAGCGTTTG
GTTATAGACTTTAGGAAACTTAACGAAAAAACCATCGCTGATAAGTACCCAAT
GCCAAACATCCCCATGATACTGGCAAATTTAGGAAAAGCCAAGTACTTCACA
ACATTAGATTTGAAGTCCGGCTACCACCAGATATACTTGGCCGAACATGACCG
TGAAAAGACCTCCTTTTCGGTGAACGGCGGAAAGTATGAATTTTGTCGATTA
CCGTTCGGATTGAAGAATGCGGGAAGCATCTTCCAAAGAGCGATCGACGATA
TCCTACGGGAACAAATTGGCAAGTCATGCTACGTTTATGTAGATGACGTCATT
ATTTTTTCTGAAAATGAAAACGACCATGTTAAGCACATAGATTGGGTCTTAAA
AAGCCTGTGTGATGCCAACATGAAGGTGTCCAACGAGAAGACGCACTTTTTT
AAGCAAAGCGTTGAGTACCTTGGATTTATTGTCACCAATGGAGGTGCAAAAA
CCGACCCAGAAAAGGTAAAGGCCATAAAGGAATACCCAGAGCCTACAAATTT
GTATGAGCTAAGGTCATTTTTGGGTCTGGCCAGTTATTACCGCTGCTTCGTTA
AGGACTTTGCGGCGATCGCTAGACCGTTGACGAGCTTGATGAAAGGAGAAA
ACGGCTCCATCAGCAAGCACATGTCCAGGAAGACTCCCATCGAGTTCGGTGA
TTTGCAGAGAGATGCATTCGAGAGGCTGAGAAATGTCTTGGCTTCTGAAGAT
GTAATTCTCAGATACCCTGATTTCAGGAAGCCATTCGATCTAACGACGGACGC
TTCCGCAAACGGTATCGGTGCAGTCCTATCGCAAGATAAAAGACCCATCACT
ATGATCTCTAGGACCCTCAAAGAAAGCGAGTCACACTACGCCACGAATGAAA
GAGAATTGTTGGCCATAGTGTGGGCCTTAGGCAAGTTGCAACACTACCTGTAT

GGCACTCGCGATATTAACATTTATACGGACCACCAACCGTTGACATTCGCGGT
ATCCGATCGGAATCCAAATCCGAAAATAAAAAGATGGAAAGCGTATATCGATG
ATCATAACGCAAAAATCCACTACAAACCGGGAAAAGACAACCATGTGGCAG
ACGCTCTTTCCAGGCAGAACATTAATGCCTTACAAAGTGAGCCTCCGTCAGA
CGCTGCGACTATTCACAGCGAGCTGTCACTGACCTACACGGTCGAAGCAACA
GACCAGCCAGTGAACTGCTTCAGAAACCAAATTGTTCTAGAAGAAGCACGT
TTCCGACTAAAGCGAAGCTTGGTGTTGTTTCGTAGTAAAACTCGCCACTTAAT
CAATTTCGCCGACAAAAGCACCATTTTGGAAATGCTAAAGGAGGTCGTGAAC
CCCGATGTCGTGAATGCGATACACTGCGATCTACCCACCCTGGCAAGTTTTCA
ACACGACCTAATTGTTCATTTCCCGGCTACGCAATTTCGATACTGTAAAAATAT
TGTAATAGACGTTACCAATCGAAATGAGCAGTTGGAAATTGTCACGACAGAG
CACAATCGTGCACACAGGGCAGCGCAGGAGAATACCAAGCAAATCCTCCGC
GATTACTATTTCCCCAAAATGAGCAGCCTGGCAAAGGAAGTAGTTGCAAATT
GTAAGATATGCACCAAAGCCAAATATGACAGGCACCCTAAGAAACAGGAGCT
CGGGGAAACACCTATTCCTAGCTATACCGGCGAAATGCTGCACATTGATATCT
TTTCGACCAATAAGAAACAATTCTTAACGTGCATTGACAAGTTCTCAAAGTTT
GCAATAGTGCAACCAGTGCTGTCCAGAACAATAGTGGACGTCACAGGGCCC
CTACTTCAACTCGTAAATTTGTTCCCCAAAATCAAGACAATATATTGCGACAA
CGAAGCCGCCTTCAATTCAGAGACTATTACCTCGTTACTTAAGAACAGCTACC
ACATTGACATTGTCAACGCGGCCCCGCTGCATAGCTCGTCAAACGGCCAGGT
GGAACGATTCCACAGCACCTTGTCAGAGATCGCCAGGTGTCTTAAACTAGAC
AAAAAGATTAGCGATACGACAGAATTAATTTTGAGGGCAACGATAGAATATAA
TAAGACCCTACATTCTGTCACTCAGGAGAAACCGGTCGAGATCCTCCACTCG
GGTCCCGATGATCGCTGCCTAGGCATCAAAGACAAGCTGGTAAAGGCCCAGA
AAAATAACATCGAAAGATGCAACCCAGCTAGGCAGAACCGTGTTTTTGAGGT
AGGAGACGAGGTTTTCGTTAAAAACAACAGAAGGTTAGGAAACAAGCTAAC
CCCGTTATGCACAGAACAAAAAGTGCAAGCAGACCTGGGAACGTCTGTTCTT
ATTAAGGGGAGGGTGGTCCACAAGGACAACCTCAAATAAAAAATTCCCACTT
TTATTTTCCTATTCCTAATTGTAAGCCAGATTTAAGTAAATATGCCGTGGTCTC
AATCTACGTTTGGTTTGCAGGTTCGCCCTCGTAACACTCATCATCCTGGCAGT
GGCAAATGCACGGATTACCGACTTTTCCCACGCCAAGTACATTCCCGTCGTAG
ATGGAGATGTACTGGTGTTCGAACATCGTAACTGCCTGAGGCATTCGAGCAA
CCTGTCTGATTATATTTACATGGTAGATGAAACAAAGAAATTGTCCGCTTCTTT
TCCACAGTCGCATATGCGCAAGTTGTTAGACGTTGATACAGATCACCTGGTAA
ACTTGTTGTCCGTTTTAAAAATACACCACCGTATCGCTAGAAGCTTAGACTTC
CTGGGTACAGCTCTTAAGGTAGTTGCGGGAACTCCCGACGCCTCAGATTTTC
TAAAGATCAAAATGACCGAAGCTCAGCTGGTAGATTCCAACTCCAGGCAAAT
AAATATAAATTCCGAAACCCAAATACAAATAAACAAACTTACCGACACCGTTA
ACAGAATTATTAAAGCCCGAAACAACGACTTGGTCGACACCCCGCATCTGTA
CGAGGCATTGCTAGCGAGGAATAGAATGCTGGCAACAGAAATCCAAAATTTA
ATTCTAACAATAACATTGGCTAAAGCTAACATAGTAAATCCCACAATCCTTAAT
CATGCCGATTTGAGCTCATTAATTTAACAAAACACTCCAATAGTTAGCTTATTA
GAAGCCTCTAAAATTAGGGTTCTTCAGTCCGACAGCATTATTCACATACTAAT

AGCCTATCCCAGGGTGAAGGTAAAATGTAAGAAGGTCCTCGTATACCCAGTAT
CACACTATCAGACAATCCTGCGACTCGATGAAGACACCTTAGCGGAGTGTGA
GGAAGACACCTTTTCGGTCACCGAGTGCGTGGAGACCACGCACGACACTTT
CTGCGAGCGGTCGCGACGCGATACCTGCGCCCGCTCACTCCATGCGGGAAAT
ACTGCCCAATGCCACACGCAATCCAACCACCTTAGGGCAGTAATGCCTATAG
ATGACGGCATAGTTATCATCAACGAGGCAACAGCCCGCGTCAGCACGGATGG
AGGCCCAGAGGTGCTCGTCAAGGGGACGCATCTAATTACATTCGAACAATCA
GCTACTATCAACGGTACGGAGTTCGTAAACCTCCGTAAGGCAATAAACAAGC
AGCCTGGCGTAGCAAGATCGCCGCTACTGAACATCGTCGGCCACGACCCGGA
ACTTAGCATGCCCTTGCTTCACCGCATGAACAACGACAACCTGCGCTTTATCC
AAGGATTCAAAGACGAGGTTGACGCCGCGGGTTCCCCCAAACTTTGGTTCGT
GGCTGGAGTAGTCCTCAACGTTGGACTGATTGGTTCGCTTATCCTTTTTCTGG
CATTAAGGAAACGGCGAGCCTCCGCTGAGATTCAACAGACCATCGATAAACT
CAACATAACCGAGGACGGTCATAATCTTAGGGGGGGAGT

>G_Jockey#LINE/G_Jockey

CACTCAACGAGTGCAGACGTGCCTACGGACCGACGGCAAGTTATTTTCGTGC
TCAAAGTCCCGCTACTCTAAAACCGCTACGTAGTGTCGCGAGATTTCTTCGCG
CACCGTGATTGGCTCAGCCGGCGAACCTTACGGTATCGCTACCACTACCAAC
GCACTCGTGCGTGCGTGTTATCGGTATCAACAGTTACATTCGGCTAAAGTTAC
TGCGAACAACTCAGCAGCAGCCACGTGCTGAGGCTGGTACACCAACAAACG
GTTCCTACCGTGCCCTCCTCCCCTCCTTCCCTACTCCGGGACAACATGGACTG
GCAAGCCCCCCCGCGACCCACCAAGCTGACCAAAGTGCCTAGAAAGAAGGC
GCTCAAGGAGAAGGTGAAAAGCAGCTGCTCAAGCGATAGCAGCTCCTCGGA
GTCAGAGCCTGGGGAAGTCAAGCGCAAAGCAGCGAGCAGAGACGCTAATGT
AGCCGCCGACAACGTGCCCAGCACCAGCGCAGCTCTGCGCAAGAAGCTGGA
AAATAACTCCTTCGCCCTCCTGTCCAGCACTGAGGACGAAGACGATGACGAC
GACAACACCGACAACGAGCAGCAAACCCCTGTTGAGGAATCTGCTCCAAAA
ACCATGAAAAAACCCAACCCGACCCCGAAGACCATCAAGCCACCCCCGATC
TACATCCCAGACGTGACCAACATCTCAGCCCTTGTCAGGATGATTACGACTCT
CGTCGGTGCCCACAAGGAATTCTCGTACAAAACTGAGAGAAACAACAATGT
ACGAGTAATGATGCCTGACAAGGAATCCTACTCAGCCCTTCGTCAGCAGCTT
GTGACCCAGAACAAAAGGCACCGCACATTTCAACCTAAGGACGAGAGAGCG
TACAGAGTAGTCATCAAAGGACTGCATCACACAACCGACCAAACGGAGATTA
GAGAGGAACTCTTGAAACATGGCCATACTGTCAGGGACCTGCACAACCCAAT
CGGCAGAAAATCAAAGGAACCCCTGGGGATCTTCTTTGTAAACCTGGAACCT
GCGAGCAACAATACAGACATCTACAAACTCAAGAGAATCTGCAGGTCGGTC
GTCACCGTTGAGCCGCCTCTGAAATTCAACGATGTTCCGCAGTGCTTCAGAT
GTCAAGGGTTCGGACACACCCAGCGCTACTGCTTTTTAGAGTTTCGCTGCGT
CAAGTGTGGTGGCCTCCACGACTCCAGGGCGTGTGAAAAAAAGGAAGACG
AGAAAGCATGCTGCCTACACTGTCAAGCCGACCATCCAGCGTCGTTCAAAGG
GTGCCCCGCGTATAAGAAGGCAAAGGCTCAACAAGCTCCTAAGCCCAAAGC
AAGGAGCATGGAAAGCAACATCAAGCCCTCCTTTGAGCTCCCAAATATTACA

AACGGTATGAGCTATAGAGACGCGCTAAGTGGCACACGCAAGTCCCAAGCA
AGCACTCCCCCACCGACACCCCCAACCCCACCTGAAGCCCCACAACCTAAC
CACATGGAGGCTATGTTCACTCGATTTGAGAGCCTGGTCGAAAGAATGATGG
AGAAGATGTTTGCTCAGGTGACGCAGCTTGTTGCTTCCATCCTCAACAGCAA
GTCATGCAAATAAGTCTCAACATAGTCTTCTGGAACGCGAACGGCTTGCAGA
GAAGCAAAGCCGAAGTTGAGCACACCATCAAAACCGACAACATCGATATTTT
ATTGGTCTCAGAATCCCATTTTTGCCCCAGATCCCACTTCATCATCTCCGGATA
CGACCTCATCACAGCCAACCACCCATCAGGTAGAGCTCGAGGAGGAGCGGC
CATGCTCATCAAAAGCGGCATACAGTTCACTGAACTGCCTGCGATACAGGAG
GATTGGGCACAGTGTGCAGTGGCCAGAGTCAATAGCCTACAGGGAGATATTA
CGGTTGGAGCGGTTTACTTCCCCCCCAGGCACGCGATTACAGAGACTCACCT
GCATGAGTTCTTCGAGTCCCTCGGACCTCGCTTCATTGCAGCCGGAGACTTC
AATGCAAAGCACTCCTGGTGGGGGTCCCGCACAAACAACCCCAAAGGCAAA
ACGCTCCACAAGTACCTGATGCGCAAAAACTTGGACTGCCACTCTACTGGAG
AGCCCACACACTGGCCCTCGGACCCTTCTAAGCCGCCGGATCTGCTGGACAT
CGCGATCTGCAAAGGCATAGGTCGTGCCAAACTCGTCTGCACTACATACGAC
AGGCTCGTATCGGACCACAGCGCCGTCAACCTGCTCCTCAACATCCCTGTCC
TCAGGAAGACGCCGCTCCGTAGACTCACGGGGAATCGCACCAATGCCCCCA
AGTTCACGTTCTGGATGCTCTCCTCCCTAAACCCAGACCCAGACCTCTCCACT
CCAGGCAACATAGACGCGGCCATCGAAAAACTGAACAAGGAGATGCACAAC
GCCGCTGAGTTTGCGAACCCTCCTCCTCCTACGACCCCGAGAACTCCCGCAA
GAGACCTGCATTTGTGGTCCCCAGAAATCGCCGCCCTCGTGGCCGAGAAGA
GACGCCTCAGACGAGTATGGTTCCTCTCGCGTAACCCCAGGGACAAGACAG
CGCTCAATCGCGCCTCCAAGGAACTCAAGGACAAACTAACCACCCTACGCC
AAGACTCGTTTCAACGATTCCTTGAAGACCTGGAACCTGGWGACCCGCAGC
ACAACCTGTGGAACGTCACGCGGCACATCAAAAGACCCGCCAAGAAAATGG
TACCAGTGCGTAAAGCAGACKGCTCCTGGTGTCGGTCTGAGGCAGAAAGAG
CCGAAGCTTTTGCTGACCACCTTCACTCTGCCTTCACTCCGTTTGACCGATGC
ACAGCTGCAGAGCAAGCTGACACCATCAGAGCTGTTGAAAGCCCATGTGCT
CCAGGACCTGCAATTCAGCCCGTCGCACCAGAGGAGATCGCGCAGGAAATT
GCCTCGCTCAGAAACGGCAAGTCTCCCGGCCCTGATCGCATCGACGCTACTG
CGTTAAAAATGTTGCCCACATTCTGCTCACAGCTGCTTGCCAACATTTTTAAC
AGCTGCTTCCGGCTAGGGTATTTCCCAAAACAATGGAAACGCGCCGAAGTGA
TTACCATCCCCAAGCCCGGCAAACCTGAAGCCAATCTTGCCTCCTATCGTCCG
ATAAGTCTGCTGGCAATCCTCTCCAAAATACTCGAAAGAGTATTTCTGCGCAG
AGTGCTGCCAGTACTGGATGAGGCTGGTTTGATCCCCGATCACCAGTTTGGC
TTCAGGCGCTCCCACGGAACACCAGAGCAATGCCACCGGCTTGTAGAGCAA
ATTTTGGAGGCCTTCGAAAGGAAGCAATACTGCTGCGCCGTCATGCTGGATG
TGAAGCAGGCCTTCGACAAAGTCTGGCACCCTGGACTCCACTATAAAATCAA
GACTCACCTTCCCGGATCCCACTTCGCCTTCCTCAAATCATTCACTGAGGGTA
GAGAGTTCCAAGTTCGCTGCGGAACAGCGACCAGCACGCCTAGGCCGATAA
GAGCCGGAGTACCCCAAGGCAGCGTCCTTGGACCAATACTGTACACACTCTA
CACAGCAGACCTTCCTATCACACCCTCCCGGAGCCTAACAGTGGCCACATAT

GCCGATGACACCGCCTTCCTAGCCTCCGCCTCAGACCCCCAAGAAGCATCAG
CCATCATTCAAAGCCAGCTGGATGCCCTCGACCCATGGTTGAAACGATGGAA
CATTGCCGTGAACGCAGACAAATCCTCCCAAACCACTTTCTCCCTGCGCAGA
GGAGACTGCCCCCCAGTCACGCTCAACGGGGAAACTATTCCAACCTCAAGCT
CCCCGAAATACCTTGGATTGACTCTAGACCGGAGGCTCACTTGGCGCCCCCA
CCTAATCACCAAACGACACCAGGCTGACCTGCGCCTCAAGCAACTCCACTGG
CTCATCGGGAAAAGGTCCAAACTTAGGGAAAACCTAAAACTCCTCCTGTACA
AGGCCATCCTGAAGCCAATTTGGACTTATGGGATTCAGCTGTGGGGCACTGC
CAGCGTCTCAAACCGCAACCGCATACAGCGCTTCCAGAACAAGTGCCTGAG
GCTAATCGCTGACGCTCACCCATACCATGAAAACTCCGTTATCCACAAGGAG
CTTGGAATGCCATGGGTAGCAGAGGAGATCTCCCGCTTCAGCGAGAGATACG
CTAAACGACTGGACAACCACCCTAACCATCTGGCTATTAACCTCCTGGACAA
CAGTGAAACCATCAGACGCCTCCAGAGGAAACACCCGCTTGATCTCCCCCAC
CTATAACCCACAACAATGAACCCCCGACCAATCTACAACTTTGTAATCCCTTA
AGTTAATGCCCCCCCCACCCAAACATTTAATTATTGTCCACATGGACAGATTTT
AAATTAATAAATAGATCGCTAAAAAAAAAAAAAAAAAAAAA

>G7_Jockey#LINE/G7_Jockey

TGGTAGCTGGTGCAGAACCGAGAAGCAGAAAGTCAACGCGTTCGCCGACCA
TCTGAAAAACGTCTTTACCCCTTATTGCCGCTGCTCTCCTGCTGATGCAGTCA
TTTATACTCCTGGAGGAACCCCTCGAGCACGTTGAGCCCATACCAACGGTCA
CAGAAGAAGAAACTGCTAAACTAATTGCTGCTGTCAAGTGCCTTGAATTTAG
ATACGGCACCTTGGAAACGTGCCGAGGTAGTAATGATACCTAAGCCTGGAAA
ACCTGAGACCAATCTCGCTTTTTATCGTCCCATAAGCTTGCTGCCGAAAGAGT
ATTTTTAAGTAGAGCATTGCCAGTTATGGACGAAGCTGGCCTGATTCCCGATC
ACCAGTTTGGCTTCAGGCGGCGTCATCAGAAGAAATCTGCCCTAGCCTTGAA
GTACGGTTTCTGAAGACCTTCCTCAAGGGTCGCAAGTTTGCGGTGAAATTTG
GTGAAGCGCGCTCTGATCATAAAGGAATTGGCCCCTGCTATACAATGCTTACA
TGGCAGAACTGACTGTGCTGCAGAGCCGTGGGAGACGCTGCAACCGAGCTG
ATGCAAAGCTGGACCTCCTAAACGCCTGACAGAAAAAGATAACTATAGCGGT
CAACAGCGACCAGTTAACGGCTTCCACATTTTCTCTCCGACCCCACAATCCCT
GAAAATGCCATATAACGGTATCACCCTCGATAGGCGACTTACCTGGAAGCCGC
ATATCCTGAAAAAGATAAACCAAGCCAACCAGCCCCTCAAGAAATATTATTG
GCTTATAGGAAGAAGATCAAAGTTACCAACTTCGTCCAAGGTTCCCATATATA
AAGCGATCAGGCCAATCTGGACGTATGGCATCCAACTATAGCACAGCCACCG
GCGCTCACCTGCTGAATGACAACGTCACCTTGGATTCCCTAGGGTCGGAGAA
GAGATAAAGAAGTGCAGCGATCGGTACATAAAGAGGCTTCATAGGCATCCCA
ACATAACGGCCATCTTCCTACTGGACAATAGTGAACAACGCCGAAGACTGCG
CAGGACTCATCCCCTAGATTTGGCTCGAGAGCCGCTGAATAAATATTCAATTC
ACCTGCTAAGTGTATGTTAAAAAAATTACTTATCCCTGGTGGATATTTTTAAAT
AAATAAAATAAAATATTTGAACTAAAAAAATATATAATAA

>G6_Jockey#LINE/G6_Jockey

CAGTCGCGATCGAACACTCGACGAGTGCAGACGTGCCTGCGGATCGACAGC
AAATTGTTCACAGTTTTAAGTCCCGTTACTTGTGCCCAGCCACTTCGCGTCGC
GTGATCTTGTCGCGCGTTTTGATTGGCCCAGCCAACGTACCTAACGGTAGTTC
GCACTAACACCATCGCACACCCGAGTGTGCGTGTTATCAGCGCAAAAAACCC
AGTGCTCGAAGCAGCGGTATATTTGCAAAGCAGCAGCCACGTGCTGCCTGGC
TCACCGGCTTACGGTGCCCAGCTTCCCCCCCCTCCTCACTCCTTATCAACTTT
GGAGAAGATGGACTGGCAGGCCCCCCCGCGCACCCACAAGCTTGGAACAAC
ACCACGCAAAAAGGCTCTGAGAACACGCAAGAGCAGCTCCAGCAGCGAGG
GAAGCACCTCGCATACAGAGCCGGACGAGATAAAGCGAAAACCGGCAAAGA
AAGCACAGGGAGAGGAGCTGGAAGAGAAGCCAAGCACTAGCGCAGCTCTG
CGCAAGAAGCTCGCCAACAACGCCTTCGCTTTACTCTCGAGCGAAGAAGAC
GAGGACGACCAAGAGAGCTCTGATGACGAACCCGGACCTAAAGACGATTCC
AAGCCCAAGACCCCCGAGAAACCAAAGCCCACCCCGAAGACCATCAAGCCA
CCTCCGATTTTTATCCCCGATGTGACCAACATCTCGGCACTCGTCAAGATGAT
CACGACTCTTGTAGGCCCGAAGAACAATTTTACCTACAAGACCGTGAATGGC
AACAACGTACGTGTCATGATGCCGGACAAAGAGTCCTATACAGCTCTGCGTC
TCCAACTTGTGGCCCAAAACAAGAGGCATCGGACTTTCCAGCCGAAAGATG
AACGTGCATACAAGGTTGTCATCAAAGGACTCCACCACTCCACCGATCGTGA
GGAAATCATTGAAGACCTTCGCAGACAAGGGCACGCTGTTAGAGATCTGCAC
AATCCCATTGGCAGAAGAACTAAAGAACCGCTGGGAATATTCTTCGCCAACC
TGGAGCCTTCCAGCAACAACAAAGACGTCTACCAAGTCAAGCGGATCTGCA
GGTCGGTAGTAACCATTGAACCGCCGCAGAAGTTCAACGACGTGCCTCAATG
CTTCAGGTGCCAAGGATTCGGTCATACACAGCGTTACTGCTTCCTGGAATACC
GATGTGTAAAGTGTGGAGGCCCTCACGAATCGAGGGCATGTGAGAAGAGGG
AGGACGACAAAGCGTGCTGCTTCCACTGCCAGGCGGACCATCCTGCGTCTTT
CAAGGGATGCCCTGCATACAAAAGGGCCAAAGCACTCGCTGCTCCGAAAAC
AAGGCCCGTCGCTAATGCTAACAAGGCGCCGCCCGTGGCATCACCAAACGTC
ACCTCTGGCAGGAGCTACCGAGACGCCCTCAACGGAGTGCACGCAGCACCG
CAGAATCCCACAACCCCAGTCCAAACCCAAACAGAAACCCCACACTCCGGT
CAGATAGAAGCGATGTTCGCTCGCATGGAAGGAATGATGGAAAGGATGATGG
AGCGCATGTTCACCCAGATGACACAGCTGGTGGCCACCATTCTCAACAGCAA
GTCATGCAATTAAAGCTCCACCTAGTCGTCTGGAATGCGAACGGCCTGCAGA
ACAGCAAGGCCATAGTCGAGCACCATCTGAAGACCCACCAGATCGATATCCT
ACTCGTAGCCGAAACCCACTTCTCCCCCAGATCCCACTCATAGGGAACTTGG
AATCCCACAAGTCGCTGATGAAATCTCCAGGCTCAGCGAGAGATACCTGAAA
AGGCTCGAAAACCACCCTAACCACCTCGCCACCAACCTGTTAGACAATAGCC
AAACAAGCAGACGTCTCATGAGGAGACACCCTCTCGATCTTCCACAACAATA
GACAACACATATAAAACCCGCCACAAATACATGTACAATAGTATCCCTTAAGC
TAATGTTCCCCCGCAAAACCATTTAATTATTGTCCACTAGGACAGATTTTAAAT
AAACAAACGCACGCTACAAAAAAAAA

>G5A_Jockey#LINE/G5A_Jockey

CCTCTGCATTTAAAAATGGGCCCCCTTAGGATACCAACTTGGAATTCAATTGG
GGCCTCCTTGAAGACCAATGAGTTCCTCGCTCTTATTGAGCTTCACGACATAG
ACATCCTCCTGTTGTCAGAAGTCCACTTCTCCTCCCGGTCCAACTTCAGGGTC
AATGGCTTTACCCTACATACTGCCAACCATCCGGATGACAGCCAACACGGAG
GCGCGGCGATCCTTGTTGGATCAAATATCTCTCATCAACCATTCGCCACTCTAT
CGCAAAATCATATCCAAGCAGTAGTTATTCAGCTGACTGCAAGCAGAGGAAA
CTTTAATGTTGCCTCAGTATACTGCCCTCCGACTCTTAGATGGACAGATGCTAT
TGTTGGGCAGTTCATCACACAATTTGGCACAAAGTTCCTTGCGGCAGGTGAC
TGGAACGCAAAACACAGATGGTGGGGAAATTACAGGATGCGTACCAGAGGT
AGGGTGCTGTATTCTGCACTTGCGGAAGAAGGAATCGACATCGTTGCAACTG
GAGAAGCCACTTGCTACCCCTATCGTGCAACTGCCTCTCCAAGTGCCATCGAT
TTCGGGATCTCCAAAGGCTTTAGACAGCAACAAATTAAGGTGCAACTACTGT
CATCTGAYCATCTCCCCTTGCTGTTTGAGCTAGATGAAGACGCCCAATCATTT
AGAAGTGTTACAAAAATGCTGTCTCCACAAGCGAACATTCGGATATTCAAAG
AACACATCGAGGCTACTGTAGAACTCAACATTCCCATTGACACCTGCAGTAG
GCTGGAAGCGTACGTCGACTACTTCACACCTGCAATCATTGAAGCTGCAAGG
CAGGCTACACCAACCCCACATCAAGCTCGTCTCACGGCCATAAGGAGGCCTC
CCATTTTAAGCATAGAGGCTAGGGACTTGCTCAGCCACAAAAGACGCCTCAG
AAGACGATACATCGCGACAGGAGATCCTAGCATTTACCAGCAATATTCAAGTA
CTACTAATAGACTGCGCCGTCTGCTAGCCAATAACCGTAAAGCAAACCTGGAT
ACCTTATTGGAGGGTGCTGGTCCAGATAGTAATAGTGGATTCTCAATATGGAA
ACTCACGAGAGGCATCAAGAGACAGCCTTTGTTTCAATCACCAATTCAGGAT
CGAGGTGGCCTCTGGCTTAAGACGGATGATGAAAAAGCAAGTGCTTTTGCTT
CGCACCTGTCCTCCACCTTCATGCCCTTCAATTTAACAGACGACACCAATCGT
GAAGCCATCGCAAATTTTTTGGATACTCCGACTGCTCCGACACGCCCCATAAG
ACATACCTCACCGCAGGAAGTAATGATGCAACTAAAGGCTTTGCAACCCAAA
AAAAACCCTGGTTACGATGGCATAGACAACCGAACTGCGAAATTTCTACCAC
GCAAAGGAGTGCTTGCTCTTGTGAAAATATTTAATGTCATGCTAAGATTAGGT
CATTTCCCCAGGCAATGGAAGCGTGCGCGCATTGTAATGATCCCCAAACCTG
GAAAGTCACCAACACAGATCGACTCATACCGCCCGATAAGCCTGCTACCAAC
TTTCTCCAAAGTTTTTGAGAGAATTTTGCTTACCCGCTTGATGGAACTGCCGC
AGGTAACAGAACACATCCCACAACACCAATTCGGTTTCAGGAAGTCTCATGG
TTGCCCCGAACAAATCCATCGCCTTGTAAAGCACATCACGCATGGCTTTGAG
CACAAGCTTTACACAGTCGGCGTATTTTTGGACGTGAAACAAGCGTTTGATA
AGGTGTGGCATGAAGGTCTGCTATATAAAATGAAAGATCTCCTCCCAGCAGC
CCATTATGCCATCCTCAGCTCCTTCATCGCTGATCGGACCTTCGATGTTGCAGT
ACGAGACTCTCGGTCAAGCATGGAACACATTCATGCAGGGGTTCCACAGGG
AAGTGTGCTTGGACCCTTCCTGTATACCCTGTACACTGCTGACATGCCAACAC
CCGTCAACAACACCGATGAAGCGTCCCCTGCTCAGCTGATCCTGGCCACCTA
CGCTGATGACACCGCCATGCTTGCGTCGCACTCATCCTTGCAATTCGCTTCCA
ATGCAGTTCAGGAATGGCTGCATGCAATCGAGCGATGGACTGCCAAATGGAA
CATAGCCATTAACTGCACTAAGTCGGCCTGTGTCACATTTACCCTGCGACCTC
AAACCTGCCTAGGTCTTCTCTTCGACGGAAATACCATTGACTACGTCTCATCC

CACTGCTACCTCGGAGTTCACCTTGATCGGACGCTGAGTTGGAAAGCCCATA
TCACGGCAGTTAGAGCCAAATCCTCTTGGAAACTGAAAAAGCTGGACTGGC
TTTTCCACTCAAGCAAACTCCATATGGCAACTAAGGCTCTTTTAATAAAAGCC
ATATTAGGTCCAACACTGACCTACGCCATCCAAGTGTGGGGAACAGCCTCTA
AGACTCAGCTCAATAGGCTTAGAGTAATACAATCGCGAGCGGCACGACATGC
ATCTGGGCTACCCTGGTATGTGAGCAACCGAGTTATTGAAAGGGACTTAAAA
GTTGTCCCATTGGGAGACCAAATAAACTTCCACAGCAGCCGATATGCCGACA
GACTTAGCGCCCACCCGAACACGTTGGCGAATGATCTTGTCGACCCTATTTCC
CTCCGACGTCTGAAGAGAATACATCCCCATGAACTCCTTACACGTAAGATAGT
ATAATCTCTACATTTGTATACACTATAATTAAGGAACAGAATCCATAATATCTTT
TAAGCTACACATTAGGTTAAGTTCAGAAGGAACTGAACGATTCCTACTGACT
AATAATAA

>G5_Jockey#LINE/G5_Jockey

ACTTCGTTTCGCAGTTGCGATAAGCCGGACGCGTTTTTGGTGCAGTGACACA
GTTTGGTTTAACAAATTTACGGACAAGTCCACAACTGTGGCACGAAGTGTAG
TGATCGAGAACATAATCAAATTATGTGTTGGCAAGTGAAAGTGTGGCGACTT
TTGCTAGCCAGCGTCGCTTTTCTTTACTTGGTGAGGCTGGCTCAAGACTCACT
GAAAAACTTCACGAGGCGATCAGCTGTGAGTGCTTTAAGCTGTAAGAGCTTT
AAGCCCTGGCTGTTTCGTGTCCACGGTACACTACTACTTTCAAACCGCGATCT
TCAAGATCTATCTGGGTCGTGTCTTAGATACTCTGCCGCTAAAACTTTAAGCT
TCTAGCTGGTTCGAGTCGTCGCTAAGCATTTTTCTTTTTGAAAATGGATAACG
GTCCACAGCAAAAATCTATCGACTACGAGTCGATGATGCTAATTGCTGGCGCG
GGCCCAAAAGAGATACGGGAGCAAATGAAAAAGAGCTGGAAAAGTGAAAC
TCCAGCCTCTGCTACTACCGGTATTTCAAACACCTACTGCAGTTCGACCTTCA
CACTGACCTCCACCAGTTCGACTATCTCGTCTGCGTACATCTTCACCTCCTCC
TTGAGCGGCAACATATGCTCTACTGCTGCCAGTACAATTCACAAAAGTGCATC
TGTCAGCTCAATCGCGAAGCCACAACTTGCCACCGGCTGGCACGACGTCTCC
TTTAAATCAAGAAATGGTGGAATCAAAAAGAGAGCAGGCGCAGTCTTCTCCC
CTAAAATGGCAAAAAAGGTTGCAACTGATATGCCGGCAAAAATCTCTGCCAA
TTGCTTCCAAGTCCTAAGCGACGATGAAGATATGGTCGTGGGAGAGGCATCT
TCGAGTGACGACGACGAGCCATGCTCCTCGAATACCGCCCTCAAACGTGCCG
CAAGGAAAGCAGGACCAAAACAACAGCTGAAAGGTGCTCACCAAACTGTA
CCAGCCGCCCCGAAATCAAGCCGTCACTCTAAGGTGCCTAGAATGATGTTTC
CCAATGTGGTTAACTTCACAGCATTTCGCAGCGAGCTGGACGCCCTTGTGGG
CGACTCCTATACAATTAAGGTCCTAAATTCGGGAGACTGCGCTGTTCAGTGCA
ACTCCCCTGATAGCTACAGGCTGGTGGCCCGGCACTTTCTCGACAAAGGATC
TCTTTTTCATCACCATCAGCTACCGGAGGACCGACCCTACAAAATAGTCATGC
GCAACATTCACCACGGAGTCCCATCGGAGGATATCATCGCAACCCTCCAGAA
CGAAGGACATAACGTTGTACGGATTTATACCCCTCGCAACAAGGCAACCTCT
CTTCCTCTTAATATGAGGTTCATTGACCTCAAAAAGGCGGAGAACAACAACC
AAATAAAGGGCATATCTGTTGTCTGCAGGCACAGGGTAATATGGGAGAAACC
CCGTAAGCAATCGGAGCCGATTCAATGCCACAGGTGCCAAGGCTATGGTCAT

ACCAAGGCATATTGTTCTCGGCACTACATATGCAGAGAATGCGGAGAAAATC
ACCCCACTGCAGAGTGCAAGCTGGAACAGGACGAGGCCAGATTCTGCTTTC
ACTGTGGTGGCCCCCATGCAGCCAACTTTAAAGGTTGCAAAAAGTACCTGCT
AGAGGCTTCTAACCGCAAAAACCAACGGAAGGTCAATGAACCCTCTGGTTC
TGGTCCTGCAAGAGGACCACACCAACCCTGTCCACCAGCCCACATGTCTGG
AAAGCCTTCATTTGCAAATTTTGTCAGAGGAAGTCAGCCCGTCGCCAAGCCT
GCCGTTATTGTCCCCCATGCAAGCGCTAACCTTGAGTCCAAGCTGGAGCAGC
TGTTTATTAGGCTTGACAGGATGATGTCACTAGTTGAAACGCTAATGCAACTG
CTCCTGCAAACTCGCACCTTCCCTTCTGCTGCTCAAAATGGGTCCTCTTAAGG
TAGCAGCTTGGAATGCCAATGGGGCCTCCTCGAAGACCAATGAGATTCTCGC
CTTCATCGAGCTTCACGAAATCGACATCCTCCTGCTGTCGGAAACCCATTTCG
TTTCCCGGTCCACCTTCAGAGTTCCTGGCTTTACCCTCCACACTGCCAACCAC
CCGGATGACAGCAAACGCGGAGGTGCGGCGATCCTTATCAGATCACTCATCT
CCCACCTACCCTTCTCCACCCTGTCGGAAAATCACATCCAGACAGCAGTTATC
CAGCTGACGGCAAGCAGGGGTACTTTTAACATTGCCTCAGTATACTGCCCTCC
TAATCTCAGGTGGACGGAGGCTGACGTCGAGCTGATCATCGCTCAATTTGGC
ACAAAGTTCCTTGCGGCAGGTGACTGGAACGCAAAGCACAGATGGTGGGGA
AACTACAGGATGTGCACTAGAGGCAGGGTGCTGTTCTCTGCTCTGGCGGGCG
AAGGTATCGACATCGTTGCAACTGGAGAAGCTACTTGCTATCCCTTTCGAGCA
AGTGCCACTCCAAGTGCCATCGATTTCGGGATCTCCAAAGGATTCAGACAGC
AAGAAATCAACGTGCAACTACTGACAGAACTGTCATCTGACCATCTCCCCCT
GCTGTTTGAGCTGGATGAAGACGCCCAGCTATTCAAAGGTGTCACAAAAATG
CTGTCACCTACTGCAAATACTGTGGCCTTCAAGGAGCACATTGAGGCCACAG
TAGATCTCAACATCCCCATAGACACCTGCAATAGTCTGGAAGCGTATGTAGAT
TACCTCGCAGCCACAATCGCGGAAGCAGCACGGAGGGCCACACCTCCCCCA
CATCAAGCTCGTCACACGACCGCAAGGAGGGCTCCCATCTTGAGCTTGGAA
GCCAGGGAGCTGCTCTCCCACAAAAGGCGCCTCAGGAGACGGTACATCGCA
ACAGGAGATCCTAGCATTAAGCAGCTATACTCTAGCACCACTAACAAACTGC
ACCGTTTGCTAGCCAGGACGCGACGAGAAAATCTGGATACCCTGCTGGAGG
GTACTGGCCCAGATAACAACAGCCATTTTTCGTTATGGAGGCTCACAAGAGG
TATCAAGAGACAGCCGTTGTTTCAATCTCCTGTCCAGAGTCACAGTGGCCTC
TGGCTTAAGACGGACGATGAAAAAGCGAGGGCATTTGCTTCGCACCTGACCT
CCACCTTCATGCCCTTCAACCTAACAGACGACTCAAATCGCGTAGCCATCATT
AATTTTCTGGACACTCCGACTGCACCGGCTCGCCCCATTAGGCATACCACACC
GCAGGAAGTCATAATGCAGCTGAAGGCGCTGCAAATCAAGAAAACCCCTGG
TTACGATGGCATTGACAACCGTGCTGCGAAGTCTCTGCCACGCAAAGGAGTC
CTAGCCTTAGTAAAAATATTTAATGCCATGCTAAGGCTAGGGCACTTTCCAAG
GCAATGGAAGCGTGCACGTATCATCATGATACCTAAAGCCGGAAAGCCGCCA
ACGAAGATCGATGCATATCGCCCAATCAGCTTGCTATCAACTTTCTTCAAGAT
TTTTGAGAGAATACTCCTAGCCCGCCTGATGGAACTGCCGCAAGTAGTGAAC
CACATACCTCGACACCAATTTGGCTTCAGGAAGTCCCACGGCTGCCCTGAAC
AAATACATCGACTGGTAAACCAGGTGACGCATGGCTTCGAGCACAAACTCTA
CACGGTCGGCGTCTTTCTAGACGTGAAGCAAGCGTTTGACAGGGTGTGGCAT

GAGGGCCTTCTATACAAGATGAAAGCTCTCCTTCCTGCTCCCTACTATGCCAT
CCTGAGATCGTTCATCTCTCATCGGACTTTCGACGTTGCAGTGCGTGATGCTC
GGTCCAGCCTGGAAGAGATTCACGCAGGAGTTCCACAGGGAAGTGTTCTTG
GGCCCTTCCTGTACACCCTGTACACTGCCGACCTGCCATCTCCCGCCAACAA
CACCGAAGTCTCCCCGGATCAGCTGCTCCTGGCCACTTATGCCGATGACACC
GCCATGCTGGCGTCTCATCCTGTACTGCAAACTGCCTCCAACGCAGTCCAGG
AATGGCTGCATGCAGTCGAAAAATGGACTGCCAAATGGAATGTGGCCATTAA
CTCCTCGAAGTCAGCCTGTGTAACTTTTACCCTGCGGCCCGGAACTTGCACA
GATCTGACTTTTGATGGAAACCCCATCAACAATGTCACATCGCACTGCTACCT
AGGAGTTCATCTAGATCGGAGACTGACCTGGAGGGCCCACATCACGTCGGTC
AAATTCAAGTCACTGGCGAAGCTAAAAAAGCTCGACTGGCTCTTCCACTCCA
GTAAACTACAAATGAGCTCTAAGGCTCTTTTAATCAAAGCCATACTTGCTCCA
ACGTGGAGTTATGCCATCCAGGTGTGGGGAACTGCCGCCAAATCCCAGCTCA
ATAGGCTCCGTGTGGTCCAGTCGAGAGCTGCACGTCACGCATCTGGGCTCCC
CTGGTACGTGACGAACCAGGTAATCGAAAGAGATCTGAAAGTTACCCCTCTT
GGGGATCAGATTAATTTTCACAGCAGCCGTTATGCCGACAGGCTTATGGTCCA
CCCGAACCGACTAGCAAATATCCTAGCTAATCCCATCTCCCTCCGAAGGCTGA
AGAGGGTACACCCCACCGATCTCCCTACCCGGAGGATAGTATAAAATATTACA
CATTAAGTAATTAATAACTAGAAAAATTTTTCCTTTTCTACTTAATAATTAAGA
AACCACTTGGTCTCATATACATTTATACACACAATAGGTTAAGTTCAGAGGAA
TTGACTGAACGATTCCTTTTGGATCCAAAATAAATAGAATGATTATTTAAAAA
A

>G4_Jockey#LINE/G4_Jockey

CGAAGTTCAAAGAAAAAAGAATTCCCTGGACAATTCATCAAGCACCAGCGC
AAATAAATTCGCTCTCTTAAGCGACGGATTGCCCGACAAAACAGGAAACAAA
TATAACAAAAACGAAGATCTCGAAATGGTAAATGAAGACAGCGCAACTGATT
CTGCTAAGCCCCCACCTATCATTCTGTCTGACGTCAATGATATAAGTGAAATG
CTAGCCTATCTTAATTCTAAAATTAAAAGAGAACTTTTTTATTATAAGACTCAA
CGTTATGGACATGTAAGAGTAATGGTTAAAAGTATTGAAGAATTCAGAAAATT
AGTCAAAACACTTAATAATGATTGTGTGCAGTACCACACATACCAGCTTAAGG
ATGACAGAGCATTTAGAGTTGTTATTAAGAATTTGCATTTTTCCACAAATTTAG
ATGAAATTAAAAGTGATGAAGAGTCAAAAGGTCATGTTGTTAGAAACATAAG
TAATCTTAAAAGCCGTGCGACCAAAACGCCACTGAACATGTTCTATGTAGATA
TTGAGCCTAACAACAAAAACCGAGACAATGTAAAACACATAGGTAACGCTAT
TGTCAATATTGAGCCTCCCCGCAAAAACAATGAAATCGTTCAATGCTATCGCT
GCCAGGAATTCGGACATACTAAATCATATTGTACCAAAACGTACCGCTGTGTA
AAATCCTCTTCTCGACACCCAAGCAACATCTGCCCGAAAAATACAGAACAAC
CAGCAAAATGTGCCAACTGCTACGAAGAACACGCCGCCAGCTACAAAGGGT
GTAGAATTTATCAGGAACTCTTGTCCAAAAAAATCAGCTACCAATCGAAAATC
CCTGAATAGCAATAAAGACCTGAGTAAAAATGATTTAGGAATCCAGCAAAGT
TTGCTCCGCCTAACAAGCCAACATATACCCAACAAAGTAACGATTATCAATCT
TATGCCCAAATTGCTGCAGGAAATAGCAAAACAAACACATCACTGGAGAGA

ATAGAGCAACTATTAGAAAAACAGTCTGAACTTACAAATAATTTACTTAACAT
GATAATGTTACTTGTAAACAAATTATGCAAGTAAATCTTAACCTTGGAATATGG
AATGGCAATGGGCTKTCCAGCCATGTAAATGAAATATCTTTATAAAAACGAAT
GAAATTGATATTATGTTAATTTCCGAGACACACTTTACCAGCAAACCATATATC
ATGGTTGTTGGATATGATATAATTAGAGCTGACCACCGTTCCTTTTAATTAGAC
CTTTTAATTAGACGGCTAAAATTAGACGGCCTAAAATTTCAAATTATGGACAG
CATTAGAGAAAATGCTATGCAGGCTGCCACGGTCACGATCAAATGTATGCATG
CGGACGTATCTGTAACAGCAATTTATCTTCCCCCAAGATTTGCTCTCAAAGAA
GCAGATTTTAAGAACTTTTTTCAAAAACTTGGACCACAGTTTATATTGAGAGG
CGACTTCAATGATAAGCACCCCTGGTGGGGCTCCAGATTGACAAATCCGAAG
GGAAGCGAACTGTACAAATGCATAGTAAACAATAGCATAACAACATTTTCAA
CCGGCAAGCCTACATACTGGCCTACTAATAGTAGAAAGATACCAAACTTAAA
AGACTTCGTAGCATACTTTGGAATCCCAGAATCTCACATGCGAATAATGGAAA
GTTTTGATCTAAGCTCAGATCATTCTCTAATAATAGTGACATACAGTACAGTAG
CTCATATATTGACAAAACCATACAAAGTAATTTCTGCAAATACAGATATCAATG
CATTTAAAAGTTATCTGGAAACAGATAAGATAGACCATGCTGTGGAGCTACTC
ACAGAACAAGATAAAGTAAGCTATATATGTACGAAGCTACCAGCCAGAAACT
CACAATCAAATCAGCTCTATCTCTCAGCTGAAATCCGACAACAAATACAACA
CAAGAGAAATTTGCGTAAAAGATGGCAAGAAACTCTCTACCCTGCCGACAA
AAGATCGTATAACAAGGCTGCATCTGATCTCAAAAAACTACTGTCAACTTTAA
GAAATGAATCTCTCGCTGAATATCTTAGAAATCTAGATCCACATTCTTGTAACC
ACGAACATAATTTATGGAGAGCAACCAAATATCTCAAGCGACCTGCAAAAAG
AAACACAGTAGTCCGAAACTGTAATGGCGAATGGTGTAGATCTGATGATGAA
CAAGCCAAAGCATTTGCTCAACACCTGCACTCTGTATTTCAGCCAAATGATAT
TGATAACCCGCAAACAGAAAGAGAAGTAGATAACTTTCTCGAGTCACCCTGC
CAAATGAGCTTACCCATTCGTAAAATCAGTATTAATGAAGTTTCATCAGAAAT
TAAATGGCTGAATAGTAAAAAGGCTCCAGGTTCGGACAAAATAGATGGCATA
ACCTTGAAAATTCTACCACCAAAGTGTGTACGATTTCTAACGTTTATATTTAAT
GCGATGTTAAGAGTTGACCACTTCCCAAGCCAATGGAAATGTGCAGAAATTA
TAATGATCCTTAAACCAAACAAGGCAGAAAATGAAGTGACATCGTACCGTCC
CATTAGTTTGTTGTCAATATTTTCTAAAGTATTTGAAAAAATACTTTTAAAGAG
AATGTTGCCAATCTTGGACGAATTCGCTATCATACCCGAACACCAGTTTGGAT
TCAGAAGAGGCCACGGAACCCCTGAGCAATGTCACAGGATTATAAATGAAAT
TTTGTCAGCATTTGAGAGCAAAAAATACTGCACTGCAACATTTCTTGACGTTC
AACAAGCGTTTGATCGAGTCTGGCATGACGGCTTATTATATAAAATCAAAAAG
TGGTTACCTGCACCATATTTTTTATTATTAAAGTCATACTTAACCAATAGACAC
TTTTATGTGCAACAAAAAAATGAATACTCGCCCTTGCATTTTATAAAAGCTGG
AGTCCCACAAGGAAGCGTCTTAGGACCTGTCTTATACACCCTGTACACGGCA
GATATGCCGGTAACAAATACCTGCACTGTGGCAACATACGCGGATGATACAGC
TATATTAGCTACCAGCTCATCTAAAGAGGAAGCCTCACAACTCCTGCAAGCA
GAGCTACGCCTTATTGAAAGCTGGTTTCTTCTTTGGAAAATTAAAGTCAACGC
CCTGAAATCTGCGCAAATAACTTTTGCATTAAGAAGAGGTGACTGCCCAGAA
GTGTCATTTAATGGATCAGCAATCCCACAAAGTAATTGCATAAAGTACCTTGG

CTTGCACCTCGATCGCAGACTAACGTGGAAAAACCACATAAAAGCAAAGCG
CCAGCAACTAAATCAAAAAAGTTTGAAGATGACCTGGTTGCTTGGCCGAAA
ATCTGCAACCACTCTGGAAAATAAAGTCCGTTTATACAAAGCTATACTAAAGC
CCGTGTGGACTTATGGCATACAGCTCTGGGGTACTGCCAGCAACTCAAATATT
GAGATTCTACAACGCTACCAATCAAAAATATTAAGACAAATTGTTAATGCTCC
ATTTTATATTTCAAATGCAAGTATCCATAAAGACTTAGGAATCCCTTATGTTAA
AGAAGAAATAGCAAAACATAGTAAAAAATATATAGACAGACTAAGAACACAT
GAAAATAACTTAGCCTTAAGTTTGGTAAATAATAATAACAACGTCAGAAGACT
TAAAAGATTTCACGTGCTAGATCTCCCCGACAGGTATTAAGTATTAAAACATG
TTATGCATAGAGTATGTAGTAGGGATTAATGGATAACTACTGCCTCTGTTAATT
TAAGATTAATTATAAAATTTACTTATTGTCATACTTTTTTAAGACAGATTGTAAA
TAAAAA

>G3_Jockey#LINE/G3_Jockey

AACTGATAAGAGACTCCAATTTAGCTGCTGTAAAGTACCAATTTAAGATGTCA
GCAGAAGACGAAAGCAATATTTTTGCGTGGCAATAAGTGCCCACAAACCCAC
GTTCCATTAAGCGAAAAAATGTAAACTCCCCAACGGTGGCGGGGAAAGTTAT
GACTATCGACCATGGACCTTCCACTTCTAGCCATGATAACTACTTTGCTGTGC
TTGCTGAAAAGGACATGTACAAATCCAACAAAAGATGATGAAATTAAAAGCG
ATACACCATCATCTGAAAAACCACCCCCAATTTTTATATCTGATGTCAATGATA
TCAACGGATGGCTTCGTTACCTTAACTTACACATGGACAGCACTGAATTCACG
TACAAGTCACAACGGGACGGACAAACTCGCATCATGGTAAAAAATGTTGAA
GAATTCAGGGAATTAGTCAAAAAATTAAACACTGACCAAGTGAAGTACCACA
CATTCCAGCTTAAGCAAGTTAGGGCATTTAGAGTTTTAGCCAAAAACATCCAC
CACTCCACCGATATTGAAAGCATAAAGAAGTCAATAGAAATGCAAGGCCACG
TAGTTAGAGGAATTCACAACATTAAAAGCCGCATTACCAAGGAGCCATTACG
AATGTTTTTTATAGACCTAGAACCACATAACAACAACTCCGAGATCTACAACG
TAAAACATATCAATAATGCTATAATAACAATTGAACCACCAAAGAAAGTTAGT
GACATGGTGAAATGTTACCGATCCCAAGAATTTGGGCACATCAAATCATACTG
CAATAAAAAATTCAGATGTGTTAAATGTGCTGAGAATCACTCAAGCATCTCCT
GTCCAAAAGAAAAGACTGAACCAGCCACTTGTGCCAACTGCCATGAAAACC
ATGCCGCTAGCTACAAAGGCTGCAGAATCTACCAAGAAATTTCCAAAAAAAG
GTCCAATGTACCCAACAGCAGGCCAGCACTAACATCTCTTTATGACATACCGA
GAGCAACATCATTCATTCCAACGAAACCAAACCATGAAAAAAGTTATGCGAA
TCAAGCTAATTATGCACAGGTAGTACGAGATGAGACTGAACAAGAAAAATCA
ACATTGGATAGAATTGAAAAGCTATTAATGAAGCAGTCTGAGCTTACATCGAA
TTTACTAAACATGATAATGATGTTAGTAAACAAACTATGCAAGTAGATCTTCGT
ATCCATCGTATATCCATATGGAATGCTAACGGCTTTCCAACCATCGAAATAAAA
TAACTTATTTATTAAAACCAGAAATATTGATATTCTACTAATTTCGGAAACGCA
CTTTACAAAGAACAATTTTATAGCCATTAAGGGATATAACGTCATAAATGCTAA
TCATCCATCTGGGTGGAAGTGCAGTTATTGTCAAAAATACTTTCCAGTACAAA
CAAATAGACAACATCTGCTCAAATATAATGCAAGTGGCATCGATTGAAATAAA
ATGTATGCATTCAAATATCTCTATAGCTGCAGTATACATCCCACCAAGACACTC

TTGGAAGCTCGATGAAATCAACTCTCTCTTTCGAGGGCTAGGAAGACACTTT
ATTGCTGGAGGCGACTATAACGCAAAGCACTCATGGTGGGGGTCAAGATTAG
TAAATCCAAAAGGCGCAGAACTATACAAATGCATAACTAACAATAAATATTCA
ACCTTATCCACCGGAAAACCGACGTACTGGCCAACAGATCTTAGTAAAATTC
CAGATCTGCTGGATTTTATTGTATTCTCTGGACTACCAGCAAGCAATTTCCAA
ATAGAGGAGAATTTTGATTTGAGCTCTGATCACACACCGATTATTGTAACCTAT
GGTACTACAGTTTGTTTCAAACAGAAGCCATACAGAATAATTAACGCAAAAA
CCAATCTAGGTGCTTTCAAAAACTGGCTCGAAGAACGAATTTGTCTAAATGT
ATCGTTAAAATCAGGGGAAGAAATTGAGTCTGCAATTGAAGCTTTGACAAAT
TCGATTCATGCAGCTGGGTACATGTTTACTCCACCGCCAAATGAAAGTACAAG
CAGGCAAACTTTTCTACCACTAGAACTAAGAGCGCAAATAGCTGAAAAAAA
AAAGCTTCGAAAAATATGGCAAACCACGCGAAGCCCAATAGACAAAAGAAA
ATTCAACAAAGCTGTCAATAACCTGAATAGCAGACTGTGGGAAATTAAAAAT
GAAGCGACAGCCGAATACCTTCGAAATCTTGATCCAAACACAAGTGACCTTT
ATTATAGTCTGTGGAAAGCAACTAAATATCTAAAGAGGCCAACCAGAAGGGA
TACCACAATTGTTAATAGCACGAGACATCGCTGCACAAGCAACGCTGAGAAA
ACTGAAGCTTTTGCTGAACACTTAAGCTCAGTCATTCAGCCAAATTTGGAAA
ATGATCCTGAAACAGTAGCTGAAGTACATAACTTTTTAGAGGCTCCTTGTCAG
ATGAGCCTGCCTATTAACCAAACTACTATCTCAAAAGTAGAAGAAGAAATAA
AAGAGCTAAACAGGAAAAAATATCCAGGATATGACAAAGTCTGTGCAATTAC
AATCAGAAATCTCCCTCCAAAATGCATCCGATTGCTGACACACATATACAATG
CCATGCTCAGACTGGAATACTTCCCCAGTCAATGGAAATGCGCGGAAATTATA
ATGATTGCTAAGCCAAACAAACCGGAAAACTGCACGTCTTCATACCGTCCCA
TTAGTTTGTTGGGGACCTTCTCTAAAGTATTTGAACGAATACTTTTAAGAAGA
ATGTTACCTGCACTGGACGAACTTAACATCATATCCGAACACCAGTTTGGGTT
TAGAAGAGGGCACGGAACGATTGAGCAATGTCACCGCATCACTCATGCAATA
GTGAACGCATTAGAAAACAAAAATTACTGCACGGGAGTATTCCTGGATGTCA
AACAGGCATTTGACACAGTCTGGCATGAAGGTCTTCTGTACAAAATAAAGAA
GTGGCTACCCGCACCATACTTCCAAATTATATCGTCCTACCTTAAGAATAGAAT
ATTCTATGTTCGCGAACATGACGAATTTTCAAGTATCTACACTGCTGAGGCTG
GAGTTCCACAAGGGAGTGCTTTAGGTCCAGTGCTGTACACAACTTCCACAGC
TGACTTACCGACTACAAATGCTGTTGAGATAGCAACATATGCTGATGATACTG
CTATTATTGCCACGAGTAATGACCCGCGCGTCGCATCCAACTTAATTCAAGAA
GAACTAGGGTTAATTGAAGCATGGCTTAAAAAATGGCGAATAGCAGTAAACG
CACAAAAATCAGTGCAAGTCACATTCTCGTTGAGACCCGGGAGTTGCCCGCC
AGTTACACTAAACGGCTCCACCATACCAGTTAGTGAAACGGTGAAATATCTTG
GAGTACATCTGGACAGGCGTCTCACTTGGAAGCAGCATATAAAAGCGAAGCA
AACTCAGTTAAAGAAAAAACCAAAAAAAATGTACTGGCTCTTAGGACCCCG
CTCTCAATTAAGCCTAGAGAACAAATTACGGCTGTATAAAGCAGTCCTAAAG
CCAGTATGGACCTACGCGATGCAACTGTGGGGAACATCTAGGAACTCTAATAT
TGAAATATTGCAGAGATACCAGTCTATGACGCTAAGAACAATCACAGGTGCT
CCATGGTATATGACTAATCATGACATCCACAAAGACATGGAACTTCCCCTTGT
TAAAGACGAAATAAAGTCAAATGCAGAGCGATACATAAGTAAACTACACGAC

CATGATAATGTCCTAGCACTAGGCCTACTAGATAACACCAGCAACCGAAGGC
GACTAAAAAGATTTCATGTACTGGACCTGCCATATAGATTTACAAATAGATAA
AAATATGTAAATATGTATATATATGGATTGAGCTCACTGGAGCTCAATTCATCC
GGTGATACAATTATTTAACGCTACAGATTGCAAATACAAAAAAAAAA

>G2_Jockey#LINE/G2_Jockey

CGAAGACCGGCCACCCCTTTCAGCAAACCCTGCACCATCAAGCTCCCAAGTA
TCTCCAATATGAGCTACAGAGATGCGCTCATGTCTACCCAAGTGCCTCAGCAA
ACCAATCCCAACCAAACCCGTCAAACCCCACCTGAATCTCCGCAACCCAGTA
ACATGGAACAAATGTTTGCTCGCTTCGAAGGAATGGTCGAAAGGATGATGGA
GAAGATGTTCTCGCAAATGACGCAGCTTGTTGCTTCCATCCTCAACGCAAAA
GTATGCAAAGCCTAAAAATAGTTATTTGGAATGCTAATGGCCTGCAGAGGAGC
AGAGCCGAGGTGGAACACCTCCTAAAAACAGATGCCATCGATGTACTACTGG
TATCAGAAACCCACTTTTGTCCCAGATCCCATTTTAGCATTAGCGGCTACGAT
GTCATCCCTGCCAACCATCCATCTGGCAGAGCGCGGAGGAGCAGCCATGC
TCATCAGGAGTGGCATCCAGTACACGGAGCTACCTGCGTTTCAGGAGGAATG
GGCCCAGTGTGCCCTGGCCAGAATCTCCAGCCTACATGGAGACATCACCGTT
GGAGCGGTCTACTTCCCACCTAGACACTCGATCACCGAGACTCACCTACAAG
ATTTTTTTGAGTCCTTTGGACCTCGCTTTATAGCAGCTGGTGACTTCAACGCC
AAACACTCCTGGTGGGGGTCCCGCTCCATCAACCCCAAAGGCAGAACGCTC
CACAGATTCCTGCAGAGCAGAAGACTGGATTGCCACTCCTCTGGTGAGCCTA
CCCACTGGCCTTCAAACCCTTCCTTGCTGCCCGACCTCCTGGATTTTGCTATC
TCCAAAGGCATAGGGCAAGACAGACTAACTTGCTCCAACTATAACAAGCTGA
TATCCGACCACAGCGCTATAAAAATGCTTCTCAACATCCCCGTCCTCAAAAAA
GAGTTGCCCAGAAGACTCACCGGGAAGCACACTGATGCCTCCAAGTTCACA
CTCTGGATGCTGTCCTCCCTGCACCCGGATCCTCCACTCTCCACTCCAAGCAA
CATCGATGCGGCAATCAAAACTCTCACGGATGAGATGTACAATGCTGCCGAG
TTTGCCAATCCTCCTCCTTCAACGTCCCCAAGAACTCCCGGTAGAGACCTTC
ACCTCTGGTCTCCAGAAATTGCGGCCTTCGTAGCGGAAAAAAGGCGCCTCAG
GAGAATCTGGTTCTTCTCGCGCAACCCAAGGGACAAGGCAGCACTCAACAG
AGCCATCAAGGAACTCAAGGACAAGCTGTGTACCCTACGGCAAGACTCCTTC
GACAGATTCCTTGAGGAGCTGGAACCTGGTGACCCGCAACATAATCTGTGGC
AAGTCACGCGTCATATCAGGAGGCCTGCAAAGAAGGCGTCGCCGGTGCGCA
AAGCGGACGGCTCTTGGTGCCGCTCTGAAGCCGAAAGAGCTGAAGCGTTTG
CAGATCTCCAGAATGCATTCACACCATTTGACAGATGCACTGGCGAAGAGCG
TGCTGCAACCACCAGGTTCCTAGAGAGTCCATGTCCTCCTAGCCTGCCCATAG
AGCCCGTCACCCCAGAAGAGGTTGCGCAAGAGATCGCCTCCCTAAAGGCTA
GCAAATCCCCAGGACTGGATCGCATCGACGCCACATCCCTTAAAATGCTGCC
ACCTCCCTGTTCCCAGTTGTTGGCCAACATATACAACAGATGCTTCTCACTAG
GGTACTTCCCGAGATCATGGAAACGTGCAGAAGTCATTCTCATCCTCAAACC
TGGAAAACCTGAAGCCAATCTTGCCTCATATAGACCGATTAGTCTGCTGACAA
TCCTCTCCAAAATACTCGAAAGAGTATTTCTGCGCAGAGTCATGCCAGTACTG
GACGAGGCTGGACTGATCCCTGATCACCAGTTTGGCTTCAGGCGATCCCACG

GAACACCCGAGCAATGCCACCGGCTCGTAGCACGCATCCTAGATGCATTCGA
GAACAAACGATACTGTTCGGCCGTATTCCTGGATGTCAAGCAGGCGTTCGAC
AGAGTGTGGCATCCTGGACTCCTCTACAAACTCAAGTCCCACCTTCCCAGTT
CCCACTATGCCCTACTCAAATCGTATACTGAAGGAAGAGAGTTCCAAGTGCG
ATGCGGTTCCTCAACAAGCACGACAAGGCCTATACGAGCCGGAGTACCTCAA
GGCAGCGTCCTTGGTCCCATCCTCTACACCCTGTTTACAGCAGACCTCCCTAT
CATACCCTCCCGTTACCTCACAGCAGCCACCTATGCAGATGACACGGCGTTCC
TTGCCACCGCAACAAACCCTCAATTAGCATCAGCCATCATCCAGAGGCAACT
GGATGCACTGGATCCATGGCTGAAACGCTGGAACATCATGATCAACGCTGAT
AAATCCTCCCACACCACCTTCTCTCTGCGCAGAGGAGAATGCCCCCCGGTCT
CACTCGACGGCGACACAATCCCTACCTCCAGCACCCCCAAATATTTAGGGCT
GACCCTAGACAGAAGGCTGACTTGGGGCCCCCACATCAACAATAAGCGTATC
CAGGCCAACATACGCCTAAAGCAACTCCACTGGCTCATCGGTAAAAAGTCCA
AGCTGCGAGAGAAACTAAAGATTCTCGTCTACAAGACTATTCTCAAGCCAAT
CTGGACGTACGGAATTCAGCTGTGGGGCACTGCTTGCACATCACATAGAAGG
AAGATCCAGCGATTTCAAAACAGATGTTTGAGAATAGTCTCCAACGCCCATC
CCTACCACGAAAATTCCGCCATCCACGAGGAGCTCGGGATTCCATGGGTAGA
CGACGAAATCTACAGACACAGCGTGAGATATGCTAGCAGACTGGAGAACCA
CCACAACCACCTGGCCGTCAACCTTCTAGACCACAGCCAATCCCTAAGACGC
CTGCAGAGAACGCACCCGCTTGACCTTACTCAACATACTTAATCATACTTAAC
CCCTACCCAAGTACACTCGATGTACTCCCCTTAAGTTAATGTTTCCCTCCAAA
AAATTTAATTATTGTCCACTAGGACAGATTTTAAATAATAAATAAAAGGCTACA
AAAAAAAAAAAAAAAAAAAAAAAA

>FW3_Jockey#LINE/FW3_Jockey

AGAGAAAATCTCGAATACTTTTGTAAACGAAATAATTGAACTTGTTCGAAAAT
ACAACTTCCGCATAATACCCCAAAAAAGGGACAGCATCCACGAAACTAAGGT
CCAAGTAAAGACCGAAGAGAGCTTTAGGACGCTTACAAAATACACTACAAA
AATCTACTACACCTATCAACTGAAAAGTAGCAATGGCCTTCAAGTCGTTCTAA
AGAATATAGACCTGAAGTAACACCTTCTGAGATAACGCAGGCATTTAATGATA
AGGATTTTAAAGTAAAAACCGTCTTCAACATTCTGAACAAAGATAGAAAACC
ACAACCACTCTTCAAAGTTGAGCTGGAACCTGACACTAAAGCCATCCGGAA
AAAAGATGTGCACCCAATCTACAAGCTAAAGTTTCTTTTGCATCGTAGAATTA
CCGTAGAAGAACACGTAGAACACACTAGCGCAAAGGGTCAGTCCAATGCAG
GAACTGTCAAGAATATGTCCAAACAAGAGGATACTGCACATTGCGTCCAGTT
TGTGTCATCTGTGGAGAGCTTCATGACTCTGCTCATTGCACGACAAATAAAA
AGTGCGGAAGCTGTGGCGGTAACCACRCAGCAAACTATAGAGGCTGTCCAAT
ATACAAAGACCTCAAAAGCCGGCTTCACCCCCGAGTAACGCCTGTACGCCTC
CATCACGCACACAATGCACTTAAACCAAAGAACTCTCAGAAATAAACCCAGA
CGTATTTTTCTCAACCGCAAAGAGATCCTCATTTGGCCCGGGGATCACCTCAC
AAAATGTGACTTTCGCAAACGTCCTGAAATGGGGACTTACTAAACCTAGCAC
TACGACAACCACTCTACATACTGCCCAAATTGATCCGACACCGAATCCTCGAT
TACCGACGCAACAACAAAGCAATATCGAAACCATGATGCATACTTTGCAGCA

GCATATGATTGAATTCATGTCCTTCATGCGGACTACCATGCAAGACCTCATGC
GTAACCAAAATCTCCTCATGGAGATGCTAATATCACAACGTTCCAAATAATCA
ATGGTTGCCCTACGAATATCATTGTAGAACGCCAACGGCGTTTCACGGCTTTA
ACTTTAAATAGCTCAATTTCATCATGACAATCAAATTGGCGTCATGCTACTGTC
GGAAACACATCTCACTGCTAAATATAACTTCCAAATAAGCGGCTTTTTGTTTT
ACGGAACAAATCATCCAGACGAAAAAGCACATGGCGGAACCGGTATCCTGAT
TAGAAATCGCATCAAACACCACCATTATAGTTTGCAGCTATTTACATGCAGGC
CACATCTATAAACGTACTACAAGGAAGCGGCAATATTACCCTCGCAGCGGTAT
ACTTTCCCCCACGTTATATTGTATCCGCAACAATTTCTGGACTTCTTCAACTCG
CTAGGGGATCATGTCATTGCTGCAGGTGACTACAACGCCAAACACACCAACT
GGGGATCTTGCTCGTCACCCCTAAAGGAAACAGTTGTATAACGCCATCATAA
ATGTACATACTGGCCTACCGACCCGAACAAAATTCCTGATTTGATCGATTTTG
CAATCACCAAAAACATCCCTCGAAACCTGACCACTCGCCAGTCCTCTTAATA
CTGAACGCCCTCCAATGTCCTGAGACTACAAAGCAGACTAGCAGATTAACAT
CAAAAAGAACAAACTGGATCAAGTACAGAAACTATATAAGCTCACATATCGC
GCTGAATCCAAGGCTTAACTCTGACGCCGATATTGAATCCGCAACAAATACTC
TCGAGAGTGTCTTAGCTGCAGCTGCCCGCATCTCTACACCAAAAATTGGGAG
TGCACCTCGTAGCCGCTCGATGGCAAGGCTTGTCATCGAGCGGCTCGTTCTT
CAAAAACGACGGATGCGACGGGAGTGGCAAGTCCATAGATCGCAATTCGCA
AAGCAAAGACTAATAAATGCTTCACGTAAACTCTCCAATGCACTCTGGCAAG
AAGAGAAAAACGCCCAACGTCGCTATATAGAACAACTATCAACATCCAGCAG
TAAAATCTCACTATGGAAAGCCCATCCCAGTTTAACTACCCCGGCAGAAACT
ATTTCACCTATAAGAACCATCGCAGGTGGATGGGCCCGAAGCGATAAAGAGA
GAGCTACCACATTCGCAACGCACCTTCAACATGTGTTCCAGCCGAACCCTGC
TACAAGTTCGTTTGTATTACCGACGCTAACAGACGACAACCAAACACCACAT
GAGCCAATCGAATTTCGAGCCAACGAAATATCAAAAATTATAAAGGATCAAC
TATATCCGAAAAAGTCCCCAGGATGTGATCTAACAACTCCAAAGATTATTATT
GAGCTTCCATACTGTGCTATATGCACTATCTCCCAGCTCTTTAACGCTATCACA
AACCTCGGGTACTTTCCAGAGAGATGGAAAAAGTCGATCATAGGAATGATAC
CGAGGGCAGGAAAGGACCTCTCAGTTTCCTCATCATACAGGCCTATAAGCCT
ATTGTCTCGTCTTTCGAAACTCTTCGAAAAATGTCTGATGACCCGGATCACCC
CCTATCTGATGACACGTAACCTTATTCCAGCGCATCAGTTCGGCTTTCGAAAG
AAACATGGAACCATTGAACAAGTCAATCGAATAACATCAGAAATACGAACTG
CTTTCGAAAAACGCGAATGCACAGGAATATTTCTGGATGTCTCCCAGGCATTT
GACAAGGTCTGGCTAGACGGTTTAATGTTTAACATCAAAGCAATGCTTCCCC
AAAACACCCACAAACTCTTGAAGTCGTATCTCTACAACAGAGCCTTTGCTGT
GAGGTGTAACTCTGATATGTCCAATGACCACGCTATAAACGCTGGAGTACTTG
GACCTACGCTATACGTCCTTTATAGATCAGATATACCCACAAGCAGACTGTTA
ACGACTTCTACATTTGCCGACGACACAGCGATTTTAAGTCGATCCAAATGTCC
TCTACAAGCAACAACTCAACTCTCTCGTCACTTATTGGCTGTGGAGAGGTGG
CTATCTGATTGGCGAATCAAAATAAACGAACAAAAATGTAAGCAAGTAACAT
TCACACTCAACCGGCAAGACTGCCCTTCGATCACATCTGAA

>FW2_Jockey#LINE/FW2_Jockey

ATCGACCACACAGACTGCTATAGATCGATACATACAAATAAAGCGAAAACTA
AGCCCGCAAAATTATTCAATGGGTAATAAATCAAAAATAAATTGTGACATAAC
AAGACAGAAAACTAACATGCCCAACAATGATAACCGATTTGCCATTTTGGCC
GATTTAGACGATCTGCCAAATTCTGACAAAGACATTAAGAAGACCCCTAAGC
CGCCTCCAATCTACATCAGAGAAAAAAGTTCCAGCGCCCTCGTAAACAAAAA
AATGGACCTTATTGAGAAAGACAGTTTCCACATAATTCCCCTGACCAAGGGC
AACATTCATGAAACAAAAGTGCAAGTCAAAACGGAAGTAAACTTCAGAGCG
CTTACCAAATACCTCAACGATGCCAAGAAAAACTTTTATACGTACCAGCCAA
AAAGCAGCAAGGGACTACAAAGTAGTATTAAAGGCCTTAGGCCTGAAATAAC
CCCTGAGGAAATATCAGGAGCCCAAATAGAACAAGGTTTTAAGCCGAAATCA
GTTATCAACATATTTAACAAAGATAAAAAGCCACAACCTCTTTTCAAGGTCGA
ACTTGAACCAGACTCTTGGGCATTGAAGAAAAACGAGAGTCACCCCATTTAC
AAGCTGCAATATCTCTTACACCGTAGAATCTCTGTTGAAGAGCCGCATAAGCG
CAAAGGACCTGTACAGTGTGCAAATTGCCAAGAATATGGTCATACAAAAACT
TATTGTACCCTTCGCACTGTATGTGTAGCTTGTGGTGAGCTTCACAGCTCTGT
TAACTGCCCATCAAACAAAGCTGACCCCGGTATGAAAAAGTGTAGTAACTGC
GGAGGTAACCACACAGCAAACTACAGAGGTTGCCCTGTATATAAGGATCTAA
AAAATCGGCTACACCAAAAAGTGACTATGATGCGTGGGCAAAGCACGCCGA
GCACAATTATACCCTCAAAACATACACCTGATGTCCACTTAAACAGTCTGACC
AACAGGAACGTAACATTTGCAAGTGCGCTTAAATCAGGTCTTGCATCTACAA
ATCCTACAACTCCATTCCCACTCGCTGAACAAAACAAGGTAAATGCTGATCA
GCCAACAGGACAGCCACAAGGCAATATTGAAACAATGATCTTTAACTTGCAA
CAAAGCATGACCGAATTCATGACATTTATGAGAACAACCATGGAAAATCTTAT
GCGTAATCAGGATCTATTGATACAGATGCTGGTATCGCAGCAGTCAAAATAAT
CAATGGCTCCCTTACGTATAACTCTATGGAATGCCAACGGCGTTTCGCGACAC
AAGCTTGAAATGGCTCAATTTCTCCAAGACAAACATATCGATGTAATGCTCCT
TTCTGAAACACATCTTACAAACAAGTACAAGTTTCAAATAAATGGTTATATATT
CTACGGCACCAACCACCCTGATGGTAAAGCACATGGAGGCACTGGAGTCCTG
ATTAGAAACCGCATAAAACATACTTTCCGCAACGACTTTGGGACAAACTACT
TACAGGCAACATCTTTAAATATACAGCTGGGTAACGGACAAATAACACTTGCT
GCTGTATACTGGCCACCTCGCTTTACGATATCCGAAAACCAATTTTTGGATTTT
TTCAACACACTAGGGGATCGCTTCATAGCTGCTGGTGACTACAATGCTAAGC
ATACGCACTGGGGATCTCGTCTTGTAACCGCTAAGGGAAAACTATTGTACAA
AACAATTATTAAAGTAAGCAACAAGCTTAGTTGCGCCTCCCCGGGCACACCT
ACATATTGGCCAACGGACCCCAGAAAGATACCAGACTTGATTGACTTTGCTG
TTACAAGAAACATCTCTCATAATCATATAAACGCCGAATCTCTCCCAGACCTAT
CTTCTCTGGCCGGGCTCATTAATTATCTTCAATATGCGGATCTGAAAAATTCAC
CCTGTCGAGTGACATCCAATAGAACTAACTGGCTAAAATACAAGAAATTCGT
AAGTTCGCACATTCAATGCCTGAACCCAAGAAAAAAACAAACTGTACAGTAT
ATGCAACGTAAGCCAAATGTTCAAATTGAGCAGCTTGTCTTGAAAAAGCGCC
GCCTACGACGAGAATGGCAATCTTATAGATCGCCATTTGCAAAACAAGGGCT
CAATAACGCAACTCATACAATAAGAAAGGAGGTAATCAACGAGCAGGAATGC
GCACAACGCCCCTACATAGCGCAATTATCACCCTTTAGCACAAAATACCCATT

ATGGGGACTACACCCCTCTGTAAGCCCACCGGTAGAATCTGTTGTGCCAATA
AGAAATTCTGCAGGTGAATGGGTCCGCAGCGATAAACATAGAGCTTCTACTT
TCGCTGAGCACCTTCAAAACGTATTTCAACCAAAGCCGGCAACGAATAATTT
CGTATTACAAGAGCAAACAGTTGTAAGCCAAACGACACAGAATACAATAGAA
TTTCGACCAACTGAAATTGCTAAAATCATTAAGGAATTAAAGCCTAATAAGAG
CCCCGGCAGCGATCTAATAAACCCTAAGATGATCATCGAACTTCCATTTTGTG
CCGTCCAAACTATTTGCCAGCTCTTCAATGCAATAAATAGAATTGGCCACTTC
CCTTCGTTATGGAAAAAGTCGATCATCATTATGATACCGAAACCGGGAAAGG
ATCATACAGTTCCATCATCATACAGACCTGTAAGTTTACTACCATGTATGTCAA
AACTCTTCGAAAAATGTCTGCTGACCTTTATTATACCCTATCTGCGAACTTTTA
ACAAAATACCGGAGCATCAATTCGGTTTTCAGGAGAAACATGGAACAATCGA
ASAAGTCAATCGAATCACAACTGAAATTCGAACTGCATTCGAAAAGAGAGA
GTACTGCACTGCCATTTTTCTCGACGTGGCACAAGCATTTGACAGAGTCTGG
TTAGATGGCCTAATGCATAAAATTATGTCAACACTCCCTGAATGCACCCATAA
GCTTTTAAAGTCGTACCTGCACAATAGAGTGTTCTCAGTTAGATGCGGCACTG
TTACGTCTGAGGACCACATCATAGAAGCTGGGGTTCCTCAAGGCAGCGTACT
TGGCCCAACACTATATCTACTATATACAGCCGATATACCAACATCGAGGCAGCT
AACAATATCTACATTTGCCGATGACACTGCAATCCTGAGCCGCTCTAAATGTC
CACGGCAAGCTACTGCACAACTTGCTCTCTACCTGGCACATATTGAGAAATG
GCTTTCTGACTGGCGAATAAAAGTCAATGAACAGAAATGTAAACACGTAACT
TTTACCCTCAACAGACAAGATTGCCCTTCGCTCATACTAAATAACACTGTTATT
CCAAAATCAAATGAAGTTACGTATCTAGGTATTCATCTTGACAGACGACTCAC
TTGGCGTAGACACATCGAGGCAAAAAGAACACACCTCAAACTAAAAGCCAA
TAGTCTACATTGGCTTCTTAGTATACGCTCTCCCCTAAGACTAGAATATAAGGT
CTTACTCTACAACTCCGTCCTAAAACCGACCTGGACCTATGGCTCCCAGCTAT
GGGGGAACGCCAGTAACAGCAGCGTAGAAATCATCCAACGAGCCCAATCGA
AAATCCTGAGAACCATCACCGGCGCACCGTGGTACGTTCGAAGTGATAACTT
ACATAGGGATTTATTCATACTTCCGGTCAGAGATGAAATAGCCAAACAAATGG
AAAAATATCGCAACAAACTACGTGCCCATCCGAACAGGCTTGCGAGAGACTT
AACACGGCTATCTAGCCGAACCCGACTTCGTCGTAATGATATGCCAACCCAAC
GATAATTATTAGGGCCACACAAACTCATATCAGTTGCAATAGTAACTGTTAGTT
AAGTACTTTTTAAGATTTGTAAACTTATTGTTAGTCTCATAATGAGAAGCTTCA
ATAAACAAACCAACGCATAAAAA

>FWJockey#LINE/FWJockey

TTCGATCGCCGACGTGTGAAGACGTTTTTATCGTGCTCCGCACAAAATCGGTT
GTTTTGAGTGAAGTGAACGCCAAATAAAATAAACTAAATAAAAAATCTGAAA
GCGAAAGAGACGCTCTATGCGATGCAAGATCGCTTAAATACATAGTGAATTGT
TATCTTAAATAATAAAACTATGAGTCAGAATGACACTCGCGCCCAGCGTCAGC
GCGAGCATGACGAACGCCGACTCTCAATTCAGCGCAACAACGCGTACTTCTC
CTACGTCTCACCGACAATCCCAAACGCAGACATCGAGCGGTCAATAACCCAT
AGCCCAGGAAACCTTCTTCTACCAACAAATCAAGAAAGAGCGCGCTCCTGCT
CTCCCGCTCTATTGGCTCCGACAGAAGCCCCGCTACCTCCAACAACAACAGC

TGGAGAGGGACCGGCAGCCCGCTCTGCCTCGTCATCGGCTGCACCCGCTCAC
GGTCTGACTAAGTCAGCGAAAGCAAAACCGCTAGCAATAAACGGTACTGCT
GCACTGCCAGCAAAACAAAACGAAAACGTAAACAAAAAAGCTGGGTCGAC
CTGGCAGACTGGAATGGACCGCTACATTACAATAAAGCGAAAGCTCAGCCCG
GAAAATTCAGATTTGGGAAACAAGCCGAAAAATACACGCGACAACTCTACCT
TGATCAAAAATGTAGCCCCTGCAAATACCAACAGATTTGCCTTGCTGGTAGAT
ACCGCTGAGGACGTGCCGCTGGGATCCGTTGATATCGAACCGAAGAAAACA
AAGCCTCCGCCAATATACATCCGCGAGAAGAGCACAAGCCGTCTTGTAAATA
CTTTGATTGGCCTTATTGGGAAAGATAGCTTTCATATAATTCCCCTCGTAAGAG
GTACTATCAACGAAATCAAACTTCAGACGAAAACGGAGGACGACTACAGAA
AAGTCACAAACTATTTACCGCACAAAAAATAGGCTTCTACACCTACCAGCTT
AAAAGCAGCAAGGGCCTGCAAGTAGTCCTGAAGGGCATTGAGTCTGATGTT
ACGCCCGAAGAGATAACTGAGGCGCTAAAGGAAAAGGGATTTTACGCCAAA
AACGTGTTCAATATCAAAAACAGAAACAGGCAGCCCCAACCACTCTTCAAG
ATTGAGCTTGAACCAGAAAACAAGCCTCCTAGAAAAAACGAGGTTCACCCA
ATTTACAAACTCCAGCTCCTTTTGCACCGTAGGATCACGGTAGAAGAGCCGC
ACAAACGCAACGCTCCTGTACAATGTACAAACTGCCAAGAGTATGGCCACAC
GAGGTCATATTGTACACTTCGCCCGGTGTGCGTAGTCTGTGGAGATCTCCACG
ACTCCAAACAGTGTCAAATTAACAAAGAAAATGCATGCGAGAAAAAATGCA
ATAACTGCGGGGGCAATCACACAGCAAACTACAGAGGCTGTCCAATCTACAA
AGAGCTGAAAATCCGTCTTCACAAAAGAATGAACACGGCGCGGGCACACCA
AGGATCAGCTACCCTGATACCATCAGAGACAAATCCTGAAGTAATTTTCTCGA
AAGCAGCTAGTTTCGCTCCCTGGCCTACATTCAACACTAACAAGACAACATT
TGCTAACGTTTTAAAATCAGGTATGACGCCTCCAACCCAAAACTCCCGAACT
CCACATGAAGTGCACACAAAATTAGACACACAACAAAACTATCACCCAGCTG
CGCAGCAGGAAACAAAAACTGAAGCTATGATGCAAGCCTTACAACAGAGCA
TGATGGAATTTATGACATTTATGAAGACCACCATTCAAGACATGATGCGTAAT
CAAAACCTTTTGATACAAATGCTTGTAGCCCAACAATCAAATAAATAATGGCT
ACCTTACGCATAGCTACGTGGAACGCCAATGGCGTTTCACAGCGCAAACTTG
AGCTAGCTCAATTCCTACATGAGAAGCATATCGACGTAATGCTTCTTTCGGAA
ACTCATCTCACAAGCAAATACAATTTTCAAATAAGAGACTACCATTTCTACGG
TACAAATCATCCCGACGGAAAAGCACACGGTGGCACCGCCATACTCATAAGG
AACCGTATGAAGCACCACTTTTACAAAGAATTTGCGGAAAATCATCTTCAGG
CCACATCTATCAACATTCAGCTGGATGACAACACTCTCCTTACACTAGCGGCC
GTATACTGCCCCCCCCGTTTCACAGTATTAGAAGCTCAATTCCTGGATTTCTTC
CAAGCACTAGGGCCACACTTCATTGCAGCAGGCGACTACAACGCTAAACATA
CTCACTGGGGATCGCGACTTGTGAACCCAAAAGGAAAACAGCTTTATAAGAC
GATAATAAAAGCCACTAATAAACTTGACCATGTTTCCCCCGGGAGTCCTACAT
ACTGGCCATCAGACCTCAATAAGCTGCCAGACCTGATCGACTTCGCAGTTAC
GAAAAATATTTCCCGCAGTTTGGTTAAAGCTGAATGTCTGCCGGATCTCTCAT
CTGATCACTCGCCTGTACTAATTCACCTCCGCCGATACGCAGAAAACGTGAA
ACCACCAACCAGATTGACCTCTAGCAAAACAAACTGGCTCAGGTATAAAAAA
TATATAAGTTCACATATTGAGCTAAGCCCAAAACTCAATACTGAATCTGATATA

GAGAGCTGCACGTGTGCATTGCAATCCATCCTTACTGCAGCAGCTCTTACTGC
AACACCCAAAATAACAAATAATACAATTAATTCAAAAAAGACCAACGTACAA
ATCGAGCAACTCGTCCACGTAAAACGTCGCTTACGCAGAGAATGGCAATCTT
CCAGATCCCCAACTGCAAAACAAAAGCTAAAAGTAGCCACACGGAAACTGG
CCAACGCTCTGAAACAAGAAGAGGACGACGATCAGCGCCGATACATAGAGC
AACTCACACCAACAGGCACAAAACAAAAGTCACTGTGGCGAGCCCACTCAA
CTCTTCGCCCACCGACTGAAACCGTTTTGCCGATAAGGAATTCATCAGGTGG
CTGGGCCCGTAGTGATGAAGACAGAGCCAACACATTTGCCGCTCACCTACAA
AATGTGTTCACGCCAAACCAGGCTACTAGCACATTCGCGCTACCGTCCTATCC
CGTAAACCGCCATCAGCAACACACCCCAATTGTGTTTCGTCCTAAAGAAATA
ACTAAAATAATCAAAGACAATCTCAGCCCGAAAAAATCCCCCGGCTACGACC
TTATAACACCGGAAATGATCATCCAGCTGCCACATTCTGCAGTTCGCTACATA
ACCAAGCTCTTTAATGCCATCACCAAACTTGGTTACTTTCCACAACGATGGAA
GATGATGAAGATCATAATGATTCCAAAGCCTGGTAAGAACCACACAGTCGCT
TCATCTTACAGACCAATAAGTCTACTCTCATGCATTTCGAAACTATTCGAAAA
ATGCCTGCTGATCCGACTTAATCAACATCTGATATACCACAATATAATCCCAGC
CCACCAATTTGGATTTCGCGAAAGCCACGGAACCATTGAACAGGTGAATCGT
ATTACAACGGAAATAAGAACTGCATTTGAATATCGCGAATACTGTACAGCAGT
ATTTTTAGACGTATCCCAAGCATTCGACAAAGTCTGGCTCGACGGCCTAATGT
TTAAAATTAAAACATCCCTACCCGAAAGCACACACAAACTTCTAAAGTCTTA
CCTCTATGACAGAAAGTTTGCAGTGCGGTGCAACACTGCCACTTCCACTGTT
CATACAATTGAGGCTGGAGTCCCCCAAGGCAGCGTTCTTGGGCCAACCTTAT
ACCTCATCTATACAGCCGACATCCCTACAAATAGTCGCTTAACGGTATCCACAT
TTGCCGACGATACAGCTATCCTTAGCCGTTCAAGGTCCCCTATCCAAGCTACA
GCACAGTTGGCACTGTACCTCATCGACATTGAGAAGTGGCTCTCTGACTGGC
GAATAAAAGTAAACGAGCAAAAATGCAAGCACGTGACGTTTACGCTAAACA
GACAAGACTGTCCTCCGCTCTTGTTGAACAGCATACCACTCCCGAAAGCAGA
CGAGGTAACGTACCTAGGAGTACACCTAGACAGAAGACTCACATGGCGCAG
GCACATTGAAGCCAAAAAAACCCAACTTAAACTCAAAGCCAACAACTTACA
CTGGCTCATCAACTCTGGTTCTCCGCTCAGCCTAGATCACAAGGTCTTGCTCT
ACAATTCTATATTGAAACCAATCTGGACCTATGGCTCACAGTTATGGGGCAAT
GCCAGCAACAGCAATATTGACATCATTCAGCGAGCACAATCAAAGATTCTGA
GAACCATCACTGGGGCACCGTGGTACGTTCGGAGTGAAAACATCCAAAGAG
ACTTAAATATCCCATCAGTTACCAACGCAATCACGGAACTTAAGGAAAAATAC
CATAGCAAGCTTCACACGCACCCCAACCACCTAGCGCGAGGTCTAATCCAGC
TCAGCAGCCGTTCCCGTCTCCGGCGAAAGGACCTACCAACCCAGCGAATAA
ATTATTAGGGCCGTTTAAACATAGAACAGTTGGAAAAATAATACAACTGTTCA
AAAAATACTTGTTATAGTTAAGATTTTTAAACTTATTGTTAGTTCTTATACAAG
AAGATTCAATAAATAAAAGCAAAGTAAAATAAAAAAAAAAAAAAAA

>FUSHI_FUSHI#Unknown/FUSHI_FUSHI

TGCCCACTGTTGGCAGGTGCTGCATTGGCCCATTTCTAATAGGCTTTTGAAGT
TTCAAATATTAATCGTCTGCAGGCAGCAGGATCACCAATCGATAGGCCAATAA
AAACAATACCCTGTGTCATCTGTGTATACAAGGCAAGTGCGGTATTTTGAT

>FTZ_FTZ#Unknown/FTZ_FTZ

AGGGCTCTCTGATTTTGCTATATATGCAGGATCTGCCGCAGGACCAGCTCATT
CGCAAACTCACCAGCGTTG

>FROGGER_LTRCopia#LTR/FROGGER_LTRCopia

TGTTGGAAATATAAGATTGCATATCATCTGTCTGGCAACTTTATTACTCTCTCA
GAGAGCAAGCTTCTCTTGCTCTCATTTGCCACTCAAAGTAGTGAAGCATGTAT
ACGAAGTTATGTACACAGTTCAGTTCTAATAAAGCATTCACCCTAATCACATC
TCATTGTCTCAATCATTTTACCGCGGCGGCACTCTTATGCAAACA

>FROGGER_ICopia#LTR/FROGGER_ICopia

TTTGGTTATGGGCCCAGGCCCTACGCGTAAAGAAATGTATTGATTCGTTTACA
AAGTATTCGTGAAGTGATCAAAGTGAAGTAACCAAAAATAAAACAAAGTTAA
GAGAAAAGAACTCGCGTCTATCTTTGTTTAAGCCAATGCGGGATAAAGAGAC
AGTTAAGTGTCTCGTATACCCCTCAGCAACACGGAATTGCTGAACGTGCAAA
CCGCACAATTGTGGAAATGGCCAAAAGTCTTTTAATACATGCTGGTTTGGATG
AATTTTTATGGGCTGAAACGTCACTTGCTAGCTGAAGCCAGCAGTACTGACG
ATGAAGAAGCCGTTTACGACAGCGATGAGGGCAGGACGTTTGTCAGTGCCA
ATGACGACTCTGATGACCAATCTGCAGATGACACCCTAATGGCACAACAGCG
AAGGCCAGGCCGACCAAAGTTCATACGAACTGGCCAACCAGGAAGGCCGAA
AAAGGTGTATCAAACTCTTCAATATTTGAATTACGAGGAAGATGAAGAAACA
CCGCAGTCGTTGGAGCAAGCACTCAAGGGCCAATTGTAAATTGGCAGGATTC
AATGCGAGCGAAGTATGAAGCGTTAATAGCCAACAATACATGGTCACTGGTA
GACCTGCCCAAAAGCAATTGGATCAAAATGGGTATTTCGCATCAAGAAAGAC
AAGGATGGCGAAATAGAGAGATTCAAATCACGACTTGTTGCCAAAGGTTGTG
GACAACAATTTGGAGTGAACTACTGGGAAACATATTCCCCGGTAATCAGGTA
CGAGACCATCAGGATGCTGCTTGCAGTTGCTGCAGAAAAAGACCTTCACATG
CATCAAATTGATATATCAAACGCATACCTAAACAGCGACCTTGAGGAGGATGT
TTATTTGAAACAACCAAAAAATTACGTTGACAAGGAAAATCCTGGAAAAGTC
CTTAAGTTGCAAAAAGCAATCTATGGGCTTAAGCAGTCTGAACGACTGTGGA
ACGATGCCTTAAATGAAGTTCTTCAAAATATGGGCTTTAAAAGAAGCAAGAA
CGAGGCATGTTTATACTACAAGAAGCAACAAAACGGTTTTAGCTATATAGCTG
TCTACGTTGACGATTTGATCATCATATCTCCCAAAGAAAGCGACATAGAAGAC
ATCAAAGGGAGCATAGCAACAAAGTTTGATATGAAGGATGGCGGCCAACTGA
GATATTTCCTTGGGATGGAAATAAGCCGAAAGGGACAAACAGGTCCTATAAA
GCTATGTCAAAAGCGCTACATTGAAAATCTACTAAGACGGTATGGCATGCAAA
GCTGTCGACTAGTGGGAACCCCATTTGACCCTGGTTATGAATCTGGTTGCACA
AATGAGAAATGTGCAAAGGTAAATTTAACCCATTTTCAATCACTAATTGGTTC
GCTTATGTATTTAGCAGTAGTAAGTCGACCAGACATATTGCACTCTGTCAGCA

AGTTATCACAGAGAAATACAGATCCTCACCACGAAGACGAAGCAGCAGCAA
AGCATGTGCTACGATATTTGTGTGGGACAATCAACCTGAGCATTATCTACATG
AAGACCGGCGAGCTGGTCAAGGAATTCGCAGACGCAGATTGGGCAAACGAC
AAAGTCGACCGAAAGTCGTATTCAGGATATGCATTCCTTATGGCTGGAAGTGC
ATTTTCATGGGGCTCATCAAAGCAGAGCGTCATCGCCCAAAGCAGCACCGAG
GCTGAATACATCGCCTTATCAACAGCAGCAAAAGAAGCAGTGTTTTAAGAA
GACTCTTACAAGAGATGGGATGGTTCGACAAGGGGCCATTGAAACTACTATG
CGACAATCTGAGCGCCTCGAGTATTGCCAAGAATCCAATTAATCACAAGCGT
ACAAAACACATTGATGTCAGATATCATTTTATAAGGGACAAAGTTAACAAAA
ATGAAATAATTGTTGAATATGTTAACACTCAGAATAATGTCGCAGATATACTAA
CCAAAGCAAAAGCATTGTGGCTTTTTGAAATCACTTGGATTTGTTTAAATTTG
TAATTGCAAAACAGATGATGTTGATCTTAAGCAAAAGTG

>FB4_Mariner-Tc1#DNA/FB4_Mariner-Tc1

AGCTCAAAGAAGCTGGGGTCGGAAAAATCGAATTTTTGAAATTTGAAAGCT
GGAATCGTTTGCCCATTTTTTGCCCATGTTTGCCCACCAATTAGTTTTTTTGC
CCACGTCCAGTTTTTGAGATATGGATTTTCGAAAAAGTTCGAAAATGTTCGAA
AATCAAAAATTTCGCTTTTTTCAAATTTTTTTTTTTTAAATCGCAATAACATC
GTTTGCCCACGTTTGCCCACCCTTTAGAATTTTGAAAAAATTTATACTTTAGA
AAATATAAGGCTTTTAAGTTTACCTCGGTCTAATCAGAGAGTAAATCGTTTGC
CCATCTCTTAAAACCAAATATTATCAACAAAAAACGTTTGCCCAACCATTATT
ATTAGTTTTTATCGTTTGCCCACCCTTTAAAAAACCTTTAACAAAATTTTTTT
TCGATTGCCCACACTTGAAATACAACCAATTTCGTTAGCCCACCTCTTCAAAA
TAAATATTTCCAATAAAAAACGTTTTCCCACCATTTAAAAATAAATAATTTCGA
TTGCCCATCCTTCAAAATTCATTTTAACGTTTGCCCACCCTTTAAAATTTGTTT
TTTTCGTTTGCCCACTCTTAAAACTAAATAATTTCGATTGCCCACCTTTTAAAA
CTAAATAATTTCGTTTGCCCATCCTTTAAAATTCATTTTTAACGTTTGCCCACC
CTTTAAAAATAAATTATTTCGTTTGCCCACCCTTTAAAATTTGTTTTTTTCGTTT
GCCCACTCTTAAAACTAAATAATTTCGATTGCCCACCTTTTAAAACTAAATAAT
TTCGTTTGCCCATCCTTTAAAATTCATTTTAACGTTTGCCCACCCTTTAAAAAT
AAATTATTTCGTTTGCCCACCCTTTAAAAGTTTTTTTTTTTCGTTTGCCCACTC
TTAAAACTAAATAATTTCGATTGCCCACCTTTTAAAACTAAATAATTTCGTTTG
CCCATCCTTTAAAATTCATTTTTAACGTTTGCCCACCCTTTAAAATTTGTTTTG
TAAGATGTGGCGCCAATTCAGATATTTTAGGATCGGCGGATAGAAGCACTTAC
TTATATGATGATGATGAACATACATAGACATAATATGTACAGCTGTGTTCAGAA
AAATAGCAGTGCGAAGGAAACTAAGTAATACAAAGGTATTTTTCCATGTCCCT
TTTCGGAATCGACTTTTTATTCCTCTTATTTTTGTTAAATGGAATGTGTAGATAG
GGAAAAAAAGAAAATCCGGTCAGTTTTTCTTGTTATCCTTTTTTTATTTACATT
CTTGAGCAAAATCACAATTTTTAGGCTGTTCATAAGAATAGCAGTGTCTGGTT
CTGACCAACGTAAAGTCCCGAAATGATCAATATTTTCTAAAAAGTGAGTTTGG
TTAAGTTAATTCGTATATTTAAAAGGACAATAAATTAAAAAAATTAAAAAAAT
TTTATTTTAGTGGGTAGAGGACAGCACTACTCCCAGGGGAAAAGAATGTTAA
TTCTTAAGCTTAGAAAGGAAGGAAAAACATATAAGGACATTCAAAAAACCCT

TAAATGTTCTGCCAAAATGGTATCCAATGCCATTAAATATAAATGGAAGCCCG
AAAACCGTGGTACCAAACATAAAACCACAGATATAGAGGATCGACGCATTGT
TTCTTACAGCAAAGTCTATCGTTTTGCATCCTTTAGGGACATAAAGTCTGAGC
TGAACTTGGGAATCAGCGACGTTACTATTCGTAGACGACTACTGAATCAAAAT
TTCAGTGCGAGGAGTCCACGAAAGGTTCCCCTACCTAGCCCAAGGCATATTA
AGGCAAGGTTAAGCTTAGCTAAAACCTACCTAAACTGGCCAGTCTCCAAATG
GCGTAATATCCTTTGGACTGATGGGTCAAAAATCATGCTATTTGGTGGAACTG
GTTCACTACAGTATATCTGACGACCTCCAAACACGGAGTATCACCCAAAACA
CCCAGTGAAGACTTTCAATCACGGTGGACCTAAAATCATGGTATGGGCTTGTT
TTTTTTATAATGGTATGAGTCATGCTATGGATTATGATTTATGGTATTATAGACC
AAAACGCATATGTAAATATACTTAGTGATGTCTTATTGTCATATTCTGAATAAA
ATATACCCTTAAAATGGACATTCCAACAGGATAATGATCAGAAACGCAGATGT
AAATCGGCTAAGAATAGGTTCACCCAAAATAGAATAGATGCAATGCCGTGGC
AAGCACCACCTTCCCATTTAAACCCGATTGAAAACCTGTATGGGGACATTAA
ACAGTTTGTGTCGAAGAAGTCCCCGACGTCTAAGACTCAGATTTGGCAAGTT
GTGCAGGATACATGGGCAAAAATTCCTCCCAAACCTTGCTAGGACTTGGTGG
ACTTCATGCCGCGTGGGTGTAAGGCTGTGCTGGCTAACAAAGGCTATCCAGC
CAAGTATTAGGCCCGAATTAACATATTAAAAAGAAAAACTAAGTTCGTTCTAG
GTCAAGTTAAATTTTGTTACTATTTTTTCATAGCACTGCTATTTTATTGAACACC
AGAATTTCTGCCTATTTATTGTTTTAATCTATATTTTCGAAACTATTGAAGAAAT
AAAAGTGAAACATTTGTTAAATTGTTTGAAATGAAATACCTAATGATATTATTA
AAAAAAAATTCCCATTAAAACTGTAAATCATAGGAATTTTTTATCTTAAACTCT
GAAGTCCAAAGCACTGCTATTATTCTGAACACAGCTGTACATAACGAAATAAT
TTATTTTTAAAGGGTGGGCAAACGTTAAAAATGAATTTTAAAGGATGGGCAAT
CGAAATTATTTAGTTTTAAGAGTGGGCAAACGAAAAAAACAAATTTTAAAGG
GTGGGCAAACGAAATACTTTATTTTTAAAGGGTGGGCAAACGTTAAAAATGA
ATTTTAAAGGATGGGCAAACGAAATTATTTAGTTTTAAAAGGTGGGCAATCGA
AATTATTTAGTTTTAAGAGTGGGCAAACGAAAAAAACAAATTTTAAAGGGTG
GGCAAACGAAATAATTTATTTTTAAAGGGTGGGCAAACGTTAAAAATGAATTT
TAAAGGATGGGCAAACGAAATTATTTAGTTTTAAAAGGTGGGCAATCGAAAT
TATTTAGTTTTAAGAGTGGGCAAACGAAAAAAACAAATTTTAAAGGGTGGGC
AAACGAAATACTTTATTTTTAAAGGGTGGGCAAACGTTAAAAATGAATTTTAA
AGGATGGGCAAACGAAATTATTTAGTTTTAAAAGGTGGGCAATCGAAATTATT
TAGTTTTAAGAGTGGGCAAACGAAAAAAAAAAACTTTTAAAGGGTGGGCAA
ACGAAATAATTTATTTTTAAAGGGTGGGCAAACGTTAAAATGAATTTTAAAGG
ATGGGCAAACGAAATTATTTAGTTTTAAAAGGTGGGCAATCGAAATTATTTAG
TTTTAAGAGTAGGCAAACGAAAAAAACAAATTTTAAAGGGTGGGCAAACGA
AATAATTTATTTTTAAAGGGTGGGCAAACGTTAAAAATGAATTTTAAAGGATG
GGCAAACGAAATTATTTAGTTTTAAATGGTGGGCAAACGTTTTTTTTGGAAA
TTTTTATTTTAAAGAGGTGGGCTAACGAAATTGGGTGTATTTTAAATGTGGGC
AATCGAAAAAAAATTTTTGTTAAAGGTTTTTTAAAGGGTGGGCAAACGATAA
AAACTAATAATAATGGTTGGGCGTTTTTTGTTGATAATATTTGGTTTTAAGAGA
TGGGCAAACGATTTACTCTCTGATTAGACCGAGGTAAACTTAAAAGCCTTATA

TTTTCTAAAGTATAAATTTTTTCAAAATTCTAAAGGGTGGGCAAACGTGGGCA
AACGATGTTATTGCGATTTAAAAAAAAAAAAATTTTGAAAAAAGCGAAATTTT
TGATTTTCGAAAATTTTCGAACTTTTTCGAAAATTCATATCTCAGAAACTGGA
CGTGGGCAAAAAAAACTAATTGGTGGGCAAACATGGGCAAAAAATGGGCAT
ACGATTCCAGCTTTCAAATTTCAAAAATTCGATTTTTCCGACCCCAGCTTCTT
TGAGCT

>DOC6_Jockey#LINE/DOC6_Jockey

CAGTCTTCGACTAGCTCTTGTCGAACGCGGATGTAAAATCTTCTATTCTCTCT
GAGAACATTTTGGTGTACATTAAATATTTTCTGTTTTCTTCTTTAATTGTGATAT
TCGCAAAAAACTACAATCAAACGTTCAAGTGATGCAATTGGCGATGCTGAAG
CATCTGCCGTTGAAAAAATGCGTACTAAAAGGAATAAACCACTAGTGGAGCG
CCAGAACGAACGGGGCTCCAAGCAATTTACAAATTATTTTCGTCCACTACAA
ACTTTAGACGATGATAATGATGATAAACCTATTGAAACGGACACACTTGATGA
AATAATAATCAAACCACCTCCTATAACATTAATTAAACAAAATACAAAATTTGT
ACACGAGCTAATGGCTAAAATTAAAACTGATGATTACTTTATTAAAACCATCT
CAATTGGAATTAAAATATTTTTACCAAATATGGACTGTTTCAATGCAGCTTGTA
ACCAACTCAAGGAACATAACTGTGAGTTCTACACGTACGATACTAGATCAAA
CAAGCCATACAAAGCCATTTTATCCGGGCTGGATAAACTACCAATTGAAACTG
TCAAATCCATGCTACAAAACCTAGGATTACAATGCACCGAAGTCAAAATTGT
GGAAAAGAAAACTAAGTCTGATCATGAATTATTACTGTACATTGTTTATTTCGT
AAACAAAAGTATCACAGTAAAGGAACTGCGCAAAAACTTTATGTATGTCAAC
CACACTAAAGTACGCTGGGAGTATAAAGTGAAATTGCAGAATAAAATTACTC
AATGCTACAATTGCCAACTATTTGGACATGGATCAAATAATTGTTCAGTTAAA
ACATCATGTGCCCATTGTGCTGGATCACACCAGACTGCCGTATGTATGGACGT
AAACAACAAAAAATGCGCTAACTGTAAAGGCGACCATCCATCGACAGAACT
GACATGCCCATGCCGAATTTCTTATCTCAATTTACTTTCCAAACGCTCATCACA
ACGCATTGGAAGAAATAACTTGGAAGCTCATGGAATGAGGAATTCTAAGCAA
CACATCGCTCCATCTGCTCTCTTTGGAAATCAACCCCAGATCTCATTGCCTAC
TCAAACTAAGAGAGTGCAACAACTCAACTTTGGTAGCTATAGTAATGCACTC
ACCAATATTCATACTTTGCCTAATCCCAAAGCAAATATCACAAATGAAACTAA
TCTCTTTTCATGTGAAGAGATAAATTCTCTTTTGTCTGAATTAATAACAAGATT
TAATGAATGCTCCAATAAGGGAGAGCAATTTCAAGTAATTTCTCAGTTAGCGA
TTAAGTATGTTTACACAAACAAATAATTTTATTGATAATTTGAATATTATGGCAT
GGAATGCTCGTGCCGTTAGAAACAAACGTATAGAACTGATCAAGTTCTTAGA
AAATAATCACATTCACATAGCACTTATAAATGAAACGTGGCTGACTCATAGTG
ACCGTTTTAATATACCCGAATACACTATATACAGAAATGACAGGAAGGAAAGT
AGGGGAGGCGGCGTTGCCATTGCAGTTAGTAACACATTGATACATGAACAAA
TACCCTGCATTAAAAGTCATGTTATTGAAAATGTAGGTATACAAATCAACACT
GACTCTAATTCCAGTCTAAAAATTTACTCTATTTACTTTGCTGGAAATACTTCA
AAATGTATTCCTAGTTCATGTGATTTATCTTGGGACCATAATCATTTAAAAAGT
TTGTATAGATCTGATCTCCTTAAGATCTCACAAATAAATGGAAATTTTCTAATT
TGCGGGGACTTCAACTCGCGCCACAGAGCATGGAAGTGTACCCGAGCCAAT

GGTTGGGGCAAAATTCTCAATGAACTTTCAGATCTGGGAAAATTCAGTATTTT
GTACCCTACGCAACCCACATATATTCCGCATAACCATAAAGCAAAAGCATCAA
CATTGGACCTATGCCTCACTAATATCCCTAACCAGCTGGCTAATCCAGCAGTA
ATGCAAGAGCTTTCCTCTGATCACCTACCAGTGGTTATTAAATACTCTACAAAT
TTTATGCGCAATGTAAAAACATATCCGATTTTGGGAAGGGCAAATTGGGCGCT
TTTTAAGAGAATTATAAATGAAAGGCTGATGGTTGAAGAGGTGGTTGTCAAC
TGCTCACAGATATCGACTTTGGATATTGATAATTTAATCGGCTCTTTCACTAGA
GTAATCAATTATGCCTTTCTTAAAGCAGTCCCTCATAAACCTATACATCACTCC
AAGCTTACACTCCCTGTACATATAACTGAATTATTTAAAACAAGAAATATTATC
CAAAGACAATGGTTTAGGTCACGTCATCCATTATTAAAATCAAGATGTGATCA
TTTAAATTACATCATACGAAAAGAGCTTTTCATTTATAATAATGCAAAATGGAA
CAATAAACTTCAAAAGCTGGATAAATGCAGCAAACCCTTTTGGAATATAACC
AAAGCAATTAAAAAAAGAAAACAAATTGTTCCGTGTCTAAGTAAAGCTGATG
AAATATACGCTTCTGGAAAAGAAAAAGCGAATATTCTTGCTGATGTCTTTCAA
CTGAAGCATAATCTGACACATTCATATTCAAACCAAAATACAATTGACCAAGT
CAAAATATCTTTACAATCTATAGCTGAAACCCCAAACAACATCGCAGAAATTT
GTAGAGTCACTTATCCAGAGGTTTTTCAATTGTAAAAGCTCTCCATACTAGA
AAATCTCCTGGAATTGATGGAATACCTAATATATCCTTGAAAAACCTTCCTACA
AGCGCCATTAATCATATTGTTAACATTGCCAATCACTGTCTTCAGGCAGGCTAT
TTCCCACGGGATTGGAAGATAGCAAAAATAGTTCCAATTCTCAAACCAGGAA
AACCTCCTGATAACCCTGAAAGCTACCGACCAATAAGCCTATTAAGCGGATTG
TCCAAGATTCTGGAAAAATTAATTAAATTGAGATTGGTGAAATTTTTGGACAT
ACACAACACACTACCAACGGTACAGTATGGTTTAGAAATGGTCTAAATACC
ATACTGCCGACCTTAAAACTAAGAAATTATATTAAAGAAAGTATCCAATCCAA
ACAATCTGTAGGGCTGGTTACACTTGATATTGAGGCTGCATTTGATACTGTATG
GCATGACGGACTGTTGCACAAACTAAAGATGATTGGAACTCCCATATACCTCA
TTAAAATAGTTCAAAATTTTTTGACACATCGATACTTTAGCGTTAATTTGAACA
GTAGTCAATCTGCTAGACGAATTTTAAATGCAGGTGTGCCACAGGGTTCTGTC
TTAGGACCTACATTGTTTAATATTTTTACACACGATATTCCACAAGCTACAAAC
TGTGTACTTTCACTGTTTGCTGACGACGCCGCTGTCTACAGTGCTGGTTTCTC
GTACAGCGAGATAAATCAATCCATGCAGAGTTACTTAAATGAATTAGACATAT
ATTACAAAAAATGGAAAATTAAAATTAACCCTAATAAAACGAATGCTATATTT
TTTACAAAAAGAAGGAAACCTCGTTATCTTCCTGATAGACAGTTGCGAATACT
AGACTCTCCAATACAATGGGTTGACAACATTCGATATTTGGGTATAATTTTTGA
TAAAAAACTAACATTTAAATACCACATCAACAACACAATTATGAAAGTTAATA
AAATTATTTGCACACTGTACCCTCTAATAAATCGTAAATCTAAATTATCAATATC
CAATAAGATCATAATTTTTAAAACAATTTTTAATCCAATATTAATGTATGGGTCA
CCTGTTTGGGGTAGATGTGCTCAAACTCATATCAAAAAACTTCAAATATGCCA
AAACAAGCTGTTAAAATTAATAATGAACTTACCTTATTACACAAACACTAAGT
ACCTACATATAAAAGCAGGTGTACAAAAAGTTCAACAAAAAATACAATATTA
ACAATCTTTTGTTTCAAGTCTTAGTTTTAAGTTAGTTGTAAGTCAATTAAGGT
TTTTCTTCTCCCTTGTTTTCCTTAATAAATAATAAATATTGTTATACAAAAAACT
AAAAACAGGTCAACTAGACATAATTAATAAATATTTCAGATGAAAGCCTTAGC

TAAACACTGTAAATACCTAATTATACCATTAGCTTTAATGAAATGTACATATATA
TATAAGGGATAAAGCACAAATAAA

>DOC5_Jockey#LINE/DOC5_Jockey

CATCTGAACTTCAACCCCGACGGACGTGTTTTTAACAGAGGTCCAGTTAATAT
CGAAGTTTAAGTGCAAAATTCAAGCCCAAGTGCAACCATCATATAAAAGCGA
AAAGACGCTAACACATATTTATATGTGTTAAAAACCTTATGTCTCTCTGTGAAC
AAGATAAACAAAAAAAAAAAAACAAGTGAAAACAATAAACAAAACAAAAA
TATGAGCGCATCCGACGCTCAAAATCAGAAGCGAGAGCTTCAAGCTAAACA
AAACAAAAACAACTCCTACCTCTCATTCACCCACATATCAAACTACACAAAC
AATTATCAAAGCTTACACCCTCAAAGTGATGGAAGAGCGCGATCTTGCTCTC
CAGCATTGGAGAGACCCAACAATTACTCTCAGCACAGGAGCGCAGAATGCG
CGCCAAATTCATCTGCACGTACACCAACAACAACTTCCGCTATACCAATAAAC
ACACACTCAACTCTCCCGCTCACAGCAGTTACGCTCTCAGCTCTCCCACTCT
GGACTCTTCCGCTCTCAGCAACTTCAGAAACCAACACCACAACTGTAGCAAC
TATAGCAACAACAACTACAGTTTTCTATGTTTCAATGCCGATTTGCTCTTCTTC
AGCGAATATTAAAACGCCCAGCTCAATACCCGCCTCAACAAAAATAAACAAA
ACTCAATCCTCTCTCCCAAACCAACAAAACTTAAAACTTTACCGAGACAATC
AGTCTCCAAACAGTTCCAAAGATCTCTTAAATAACAACAAGAAACAGAGGA
ATAAAGCTCAAACCGCTACCCAAAAAACACTCAAAAAGTACTGGCTAGCCG
AGCCACACTCAAGCAACCGTTTTGAGCTATTAGCACAAGATGATGAAGACGA
AAACCAAACTGACAATGGGACAAATGCAACTATAAATACGCAAATGCAAATT
GAACACAAAAGTGCAAAACCGCCCCCTATATATGTTCAAAATGTTGAAAACA
TATACGCACTAACTACTGCTTTAAATTCATTGGAAGAAACACGATATGAACTA
AAAGCACTATCAAGTAATGAAATAAAAATACAGCCACTAGAAACTACTCACT
ATCACAACATATTAAGACTATTAAAAGATAAATCGACCAAATACTACACCTTC
AGACCAAAAGACCAGAGAGGGTTTAAGGTAATTCTTCGTAACGTCCATCATG
CTACAGACAAAGACGATATTATCAATGAACTAGCTCAGCAAGGGCATGAAGT
TATAAATTTACACAACATCCAGCGCTACGACTCCAAACAACCATTGCCTCTAT
TCTCTATTGAACTCAAAATGAAAGACAATAATAAAGACATTTATAAAATTGAG
CACCTACTTCATTGCAAAGTAATCTTTGAGCCACCGCGACCAAAGCGAACAC
TTCCTCAATGTACAAACTGCCAAAAATATGGACATACAAAAAATTACTGCACC
AAAGACCCTACTTGCGTAAAGTGCGCTGGAAAACACAGCTCATCATCCTGTA
CTATTAACACAAGATCGGACAGAACAAGCATTAAATGTGCACTATGCAGTGA
AAACCACACTGCAAACTATAAAGGTTGTATGGTGTACAAAGCACTGCAAATT
CAAAAATACCCAACTCTTCGGAAAAAAGAAATACCTGTAACAGAAACAACA
CATCAAACGACACAACACAGTACAACAAATAACGTAGTAAGCTTAAAGACG
CCAGCTTTATCCTATGCCGAAGTCTTAAATAAAAACCAAGTAACTAGCCATCA
AACCAACTCAACACCTCAACCTACTATTGTACCTGATATACAGCCTGCTTCAA
CTCAATTAAGCCAAACTGCACAAAATGATTTTGACGAACTTAAACAAATGAT
GAAACAATTAATAGCCCAAATGACAAATATGATGAACATTTTCACGCTTTTATT
ATTCAAACTTGACAAATAAACACCTAACAATCGCCATCTGGAATGCTAACGG
ACTTTCACGCCATTTACATGAACTAAAAACATTCTTAAATGAAAAGCAAATTG

AGGTCATGCTCATTTCTAAAACACACTTAACTGAGAAAACCTATTATATAAAC
ATACCTAACTATAATATTTCTTCTACTTATCACCCGGACGGTAAGGCCCATGGT
GGTACTGCAGTAATAATTAAAAAAAGCATCAAGTGCATAGAGCTCGATGGATT
CAAAAAGGACTACATACAGGCTACTACTAAATCGACCTCAGACACAACGGGT
CCAATAAATATATCTGCAGTTTATTGCCCACCTAAATTTAATAATACAAAAGAC
CAATACCTCGATTTCCTAAAATCACTGGGAAATCGGTACTTGGCAGGAGGTG
ACTATAATGCCAAACACACAACTTGGGGGGTCCAGACTTACCACTGCAAAGG
GGACGTCAACTGTATGAAGCAATAAGAAACAATAATAGCCGTGCTCTAAGCA
CAGGAGAGCCGACTTACTGGCCAACTGACACAAATAAACTACTGGATTTAAT
TGACTTCTGCATTACAAGAAATATAACATGCGAGAACCTAACAATAAAATCAT
GCCTAGACCTCTTTCCCGACCATTCCCCAATATTACTTACATTACATGGTGGGC
TAGAAGAAAACAGCAACGACTCTCCTTTATTTAAAGACAAAACAAATTGGAA
CATGTTTCGCGACATTTTGGAACAGAATAGGAATATAAATATCTCTCTAAAGA
CAAACAACGCACTAGACTCCGGAGTAGCATATTTAAATGAGAACATCATAGAT
GCAGCAACGCAATCGACACCATCTATAAAAATGAAATGAGAAAAATCAGGCA
AAAGCGTACACTTAGGAGGATACGGCAAAGGACTAGGCATCCAGAAGATAA
AAACAAACTAAATAGAGCAACAGACGAGCTCAAGAGAACTCTCAGGGAAG
ACAAAGATAACCGACTTCAATACTACCTTAGCAAACTTGAGACTACCTTATCT
ACAAATTATTCCCTGTGGAAAGCGACCAAAAAATTTACGAACTCAACTATAC
ACAAGCCACCTATAAAAAGAAACAACAACACATGGGCAAGATCAAACAAAG
AAAAAGCAGACACCATTTACAACGACAATGCTGAGCTTCCAAGACAAATCA
CCAACTTAAATTGTGCAATTAAAACTATAACAAAGGCAGAAATTAAATCTCAC
ATAAAACAAATTAAAAACAAGAAAGCCCCCGGATATGATTTAATCGGAGGAA
AAATTCTTAAAGAACTACCGGACGCTATAATCACCTATATTAGAAACTTGTTTA
ACGGAATCCTTCGATTGCAGTACTTTCCCATAACTTGGAAGGTAGGCCAAATT
AAAGCAATTCCAAAACCAAACAAAAACGCCAATACTGTAAGCTCGTACAGA
CCAATCAGCTTATTACCTGTCCTCTCAAAAGTATTTGAAAAAGTCCTATTTGA
AAAACTCCTATTTAATAGAATCGAGCCAATCCTAAAACAAAAAAATATAATAC
CTCCTCACCAATTCGGCTTTCGGAAGCAGCACTCGACTGTCCAACAGGTCCA
TCGAGTAATTAACAAAATAACAGACGATTTTGACAAAAAGAAATTTTGCTATG
CTGTGTACTTGGACATTGCTAAAGCATTTGACAAAGTATGGCACAAAGGACT
GCTGCATAAGCTAAGAAATATACTACCATATAACCTATATACCATTCTTCAAAG
CTATATAACGGACAGATATTTCTATATTAAATACGACAATGAATGCTCAGATATT
ATGCCAGTGTCAGCGGGTGTTCCCCAAGGTAGTGTGCTTGGGCCCATATTATA
CCTGATTTATACCTCCGATATTCCAGCGCCAACTACCCCTGGATCAATGATTGC
GACGTTCGCAGACGATACTGTGTTAATGTCGTCAAGCGCGTGCGAAAAAACA
GCTGCAAGTACACTACAACAAATCATGACACAAACAGTATCCTGGTTTGAGA
GATGGGGTATCGAGATAAATAAAGACAAAACTCAACAAGTAATATACACAAA
ACGACAACCCAAAAACTTCGCAATTAAAATCAATGACAATCAAATATTATTAC
ACCCATATGCCAAGTACCTTGGACTAACTATTGACTCAAAACTAACATGGAAA
CAGCACATTCTAAATAAACGAAATGAAATAAAAAACAAATTTCGACAACTTA
ACTGGCTAATAGGAAAACACTCAAAACTAGAATTACATAATAAAATAGCAATA
TACAAGGCGATAATAAAACCAATATGGACGTATGGAATAGAACTCTGGGGAA

CTGCCAGTAAATCCCACTTACAGCTAATTCAGCGAACGCAATCAAAAATGCT
CAGAATAATCACAAAAGCCGAATGGTACATTAGAAACGAAGATATACATAATG
ATTTAAACATAGAAACAGTCAACGAAACTATTAAAAAAAAGCAGCAAAAAA
CACACAAATCAACTACTTAGCCACCAAAACTCAAACTTGAGACAAATACCGA
TGACAGAGTTGGAACCAAGAAGATTAAAACGCAGAACTCCAACAGAATTAA
ACTATTAAATTATTAATGTATTAGCGTATCCTTGCACTGGGAAGGGCCGCTTCT
TTTCTTTTTCACATCTAGTCAACCAATTACTTATTGTTATTTTTTAAAAAACAG
ATTGTAATTTAAAGAAAAA

>DOC4_Jockey#LINE/DOC4_Jockey

CAAAACGAAATTCGTGAGCAACGTCAACGTGAACAAGACGAGCGTCGGCTC
TCTTCAATCAAAAACAACGCGTATTTCTCGTTCGTTTCTACGGAAACGCTCAA
GCCCTCCCAAGACCAGAGAGCGCACTCTTGCTCTCCGTCGTTGCCAAGCACA
GTTCCAAAATCTTGGAGCGAAGAGTGCGCTTCCCCAATGCCGCAACCAACA
ACAAATTACACACAAACTCCGTTAACCATTGTAGCCAAGCCTCCTCTCTTAAG
CCTAGCTGCAGTAACTACCAGCACGGTTGCCTCTCTCACAACTGCAATCGCAT
CGACAACAACATTAACCGCAACAGTCTCTTCAACTCAAACGATTCAGTCAAC
GACCTCACGGGCTTTTGCATTGCAAGCAAAACAATTAACAGCTACAATTACC
GATTTACGTGAGTCAAAGAGCAATCCAAGTGGCCGCCCGAATTCGGTCATGC
AGACTGGTATGGATCGCTACGTTTTTGTAAAACGTAAACGGAGTCCGCAACG
CACAACAGGTAATAGAGCCAAAATAAATTGTGGTAGCGAAACAAAAACTACT
ACTACCAACAATAAAAACATGTTTGCACTGCTAGCAGAAAACGCAAGCGAG
GAAGCTAATAAAACGACAGATAGTGTATCTCAAAAACCAAAGCCTCCACCAA
TATACATCCAAGAGATTACTACGAACGCACTAGCAAATAAAATAGTTCAGCTT
ATCGCAAACAACAATTTTCACGTTATACCTCTAAGAAGAGGAAAAATTCAAG
AAACTAAGCTTCAGGTTAAAACCGAAGACCAGTTTCATGCGGTTTCGAAGTT
TTTAAACGAAAACGGCAAAAAATATTACACGTACCAACTCAAAAGCAGTAAA
GGGCTGCAAGTGGTACTAAAAGGCATTGAGCCTGATGTCACACCCAATGAAG
TTGCGAATGCACTTCACGAAAAGGGCTTTAACGTTAAATCAGTTATTAATATT
CTTAATAAAGATAAGAAGCCCCAACCGCTCTTCAAGGTCGAGCTAGACCCAA
CTAGCCAAACACTAAAGAGAAATGAAGTGCACCCAATTTACAATCTCCAATT
CTTATTACACCGTAAAATTACTGTAGAAGAACCACATAAGCGTAATGGTCCAG
TACAATGCTCCAACTGCCAAGAATATGGCCACACCAAGACATATTGTACCCTT
CCTTCCGTTTGCGTCGCCTGCGGGGATCTGCACGAATCTGGCACTTGCCCGG
CTAATAAACTTGATCCAAATTCAAAAAAATGTGGAAATTGTGGTGAAAACCA
CACAGCTAATTACCGGGGATGCCCTGTCTACAAAGATCTTAAAAGTCGCATG
AACAAACGAATTGCTTCGGCTCGTAGTACTAATAATACCCAAAAAATATGGTA
CCCCAAAAATGTATGGTACCCTCCCAATCGTTTCCTGACATTTTCTTCTCCAA
CGCCAAATTGTCATCCTCTTCCTCCAGTAAGCACCATACCGGGTGTTTCATTT
GCTAGTGCCCTAAAATCGGGAACGGAAGGTCCTGCGTCCACAACAGAGTTCT
CAAAAGCGCCGCAACCTCGAGCCGTGCCAGACGACCTGCAACAACCCACAA
GTGCCATCGAAAAAATTATGTTATCATTCCAAGCAACGATGACGGAATTTATG
TCGTTCATGAGAACAACAATGCAGGATCTTACGTGTGCCTTAGTACAAGTACT

TGCATCTCAACATTCAAAATAAAAAAATGTCCTCCCTTCGTATATGTTTGTGG
AATGCGAATGGCGTCTCCCGCCACAAACTTGATCTCGCTAGGTTCTTGAAAG
ATAAAGAAGTTGATATTATGTTGCTCTCAGAGACCCATCTAACCAGCAGTCGC
ATTAAACATAACTTCCTTGATCGTTTTGAGAAAAACTACCTACAAGCTACATC
TGTGGTGGTCYATTCTAGTGGCGGTAATATAACTCTGGCCGCCGTCTACTGTC
CGCCCCGTTTCTCAATTACTGAGAATCAATTTATGGAATTCTTTTGCTTTCCTT
TAGAGCTTCTTAACGMTGTACAAHDTGAADACTTCTCACTGTAAGGTGCAAT
GCGAATACCTCCGAGGACATGCGAAGGGGAGTCCCTCAGGGTAGCGTTYTTG
GTCCGACACTGTATCTCCTATACACAGCTGACATCCCTACAAGTGATCGAACA
ACACTATCAACTTTTGCTGATGACACCGCAATTCTCAGCCGATCGAAATGCCC
TCTGCAAGCATCAGCGCACCTGGCTGGTCACCTAACTGTTGTGGAGAATTGG
CTGGCTAACTGGCGTATTGCAATCAACGAGCAGAAATGCAAACATGTCACAT
TTACGCTTAATAGGCGTAGCTGTCCCCCATTAACTCTAAATAACGTCCAAATTC
CACGAAGTGATTCAGCCATTTACCTCGGAGTTCATCTAGACAGAAGATTAACT
TGGCGTAAGCACATTGAAGCCAAGAAAACGCATTTGAAGCTGAAAGCCAGT
AGGTTTCACTGGCTTATCAACTYACGCTCTCCACTAAGTTTGGACTACAAGGT
GCTGTTATACAACACAGTTCTAAAACCAATATGGACATATGGATGTCAACTAT
GGGGCAATGCATGCAACAGCAACATTGAAATAATACAACGAGCACAATCAAA
AATCCTGCGAACAATAACTGGTGCGCCTTGGTACATCCGGAACGCGAACATC
CACCAGGACCTACGGATTCCAGTTGTAAAAACTGAAATTGTCCAGCAGAAGG
CTAAATACCTAAAGC

>DOC3_Jockey#LINE/DOC3_Jockey

TGGCATTTCAAGTGAACAGACGTGTTTTCATCGAGCTCCGTATAAAATCACTT
GATTTTAGTGAAGTAAATAGTTAATAACATAAAATAAATAAATAAATCGAAAG
CGAAAGAGACGCTATTGGCAATGTTAAATTGCTATACTACAAAATTTTCTGTG
TGTGGAATAATAAAGTAAAACTAAAAAACAAATAGACAACGAACGAATCGAT
CGTTCTAAAATGAGCCAACGCGAATTGCGCGCTCAGCGGCAAAAAGAGCAA
GACGAGCGCCGACTCTCTTCGCAGCGGAACAATGCATACTTCACCGTCGTGT
CTGACGCCCGCGAGTGCCAAATCATCCACCCGGTCAATGACCTAGCACCTGC
AGAATGCAAACGATCGCGGTCTTGCTCACCAATGCTTATGACTGCACGAAAT
GAACCCGAACGCACTGAGAGCGTCTCTCTCAAGCTCTCCCCATTCCAAACGG
TGTCAAGTTCTTTAGCTACCAGTACAGCAATCACGATATCTACTGCAACTGCA
CCTTTAGCAACAACAACTGCAACAACAACTGCAACAGCATCAACATCAACTC
TATGCTCAATACCGACAGCGGTGTACAATTCCGCTGTACCAACAACAACGAA
AGCAAGAGCACTCACACCGACTCTTGAAAGCGGAAGCGGACAAGAACCAA
AACAAAACAAAAAACTTTGTCAAACAGGGCTGGATCGCTACATCCAAATAA
AGCGAAAGCGAAGCCCACAACACAAGCAGGCTGGTAATCAGCCGAAAATAA
ATCGCGCCAACGCCTCCAATGACTCAAGTTCAAATAGGTTTGCACTATTGGAT
GACTGCGAAGTGCAGGAGCAGGAGCAGAAGAAGATAAAGCCGCCACCAATA
TATATACGTGAGAAAACCTCAAGTGCGCTGGTTAACAAACTAGCTGCAATTAT
CGGAGAAAACAAGTTCCATGTTATACCTCTTACTAAGGGCAACATACAAGAA
ACGAAAGTGCAGACTCAGGATGAGTCTAGCCACCGATCAGTGACCAAGTAC

CTGGACGAAGCTGGGAAGAGTTACTATACCTACCAGCTGAAAAGTGCCAAA
GGATTGCAGGTTGTCATTAAAGGAATAGAGTCATCAGTGACCTCATCCGAAAT
TATCGCGGCACTAAAGGAGAAGAACTTCAACGCCAAAACGGTGATCAACATT
CTCAACAGGAACAAAGAGCCGCAGCCACTCTTTAAAGTCGAACTGGAGCCA
TCGAGTCAGAAAAACAACAAAAATGAAGTACATCCTATATATAAGCTACAGT
ATTTACTGCACCGAAGGATAACCGTAGAAGAGCCACACAAGCGCAGGCAAC
CTGTGCAGTGCACCAACTGCCAAGAATATGGACACACCAAAGCGTACTGCAC
CCTAAAATCCATCTGCGTCGTTTGCAGCGATGCCCACAGTACTGTAAACTGTC
CTAAGAATAAAAACGACAGTTCAATTAAGTTATGCACCAACTGTGGCGAAAA
ACACACAGCCAACTGGCGAGGATGCATTGTTTACAAGGAACTCAAGAGTCG
CCTAAACAAACGAGTGGATTCAGCTCGAGATCGTATCACACCAACAATTCTC
AAAGCAATGGAACCGACTACGACTAACATCCCTTCGTCTGCCAGCCACCGAA
CAATGCCAAATTCTTCCTTTGCAAGTGTACTAAAATCAGGGATCCAAGCACC
AACAGCAGTCACCTCCAGCGTCCAGCATAAAACTCACCAAATAAACACAAAT
AAACAAATAAACACACAGAACATGCAACAGCAGCCAATTGGCGTTGAAGCG
ATTATGCTTTCGATGCAGCAAAGTATGAAAGACTTCATGACCTTCATGCAAGA
AACTATGCAAGAGCTTATGAGGAACCAAAACATCCTTATTCAAATGCTCGTTT
CTTCCAAAATAGGATAATGACTCCCTTAACAATAGCTACCTGGAACGCTAATG
GCGTTTCGCGGCACAAGCTAGAACTTGCTCAGTTCTTAGCCGACAGAAACAT
TGACGTTATGCTACTCTCAGAGACTCACCTTACCGAAAAGTACAACTTCCAA
ATACCAGGATATAAATTTTACTTTACGAATCACCCGGATGGCAAGGCCCATGG
AGGCACTGGCATACTAATACGATTGCGAATTAAGCACCACTTTCTTGGTAATT
GGCAAGAAGACTGCTTACAATGTACCTCTATAAACCTCCAATGCCATAACGGT
GCTCTCACTCTGGCTGCTGTCTATTGCCCACCTCGCTTCACAATCTCTGAGAA
AAAATTCACAACACTTTTTGACTCGTTTGGTGAAAGGTTTGTTGCAGCTGGC
GATTGGAATGCCAAGCACATGTACTGGGGATCTCGGATAGCGAACCCTAAAG
GAAAACAGCTGTACAACGCAATCATCAAACCGCAACACAAACTTAACTATGT
TTCCCCGGGAGCGCCTACATACTGGCCAGCAGATCCCATGAAGCTTCCAGAT
TTGATTGACTTTGCCATCACAAAAAGGATATCCCAATCAATGATTACAGCTGA
GGCACTTCCAGAACTCTCATCTGATCACTGTCCGGTAATCTTTCATCTTTTGC
ACCAACTACAACACATCGAGCGACCGTGTAGGCTTACAAGCAATAGGACCAA
CTGGGCGAGGTACAAAAAATATGTTTGTTCCCATACCGGCTTTTCTAGCCCCC
TGGAAACCGAGCAAGACATTGATCAGTTTGCTGGTGATATAGAATCAATACTT
GTCGCAGCAGCAAAAGCGTCAACTCCACAAGACAACCATGTATGCAGCACA
AAGTTTAACAAGACAAGTAGAGATATTGAACTGCTTGTGCTTGAAAAACGAC
GACATCGAAGGGAGTGGCAGGAGCACAGATCACCTGGCGCAAAGAGTAAAT
TAAAAGCAGCTTCTCGCAGACTTACCAAAGCGCTTAAGAAAGAAGAAGCGG
ACGCACAACTAAGATACATCGAGAATCTCACGCCCACAGGCACAAAGAACT
CGTTGTGGAAAGACCACAGGAACCTACAGGCACCAACAGAAACGGTTGTAC
CTCTCCGTAACTCTACCGGAAACTGGTCTCGCAGTGACACGGACAGAGCAA
GAGTCTTCGCTGATCATCTTAAAAAAGTATTTCAACCAAATCCAGCCACTAAC
TCATTTACTCTCCCGCCTTTAACTGCATCTGACTTAGCACCACAAGATCCTGT
AGAATTTCGGCCAAGCGAGATAACGAAAGTCATTAAAGAGCAACTGAAAAC

TGGAAAATCGCCTGGCTACGACTTAATAACCCCGAAAATGATCATCGAGCTCC
CAATGTGTGCAGTTCTGCGAATCTGCTTGCTCTTCAACGCAATTACTAAAATT
GGATACTTCCCTCAAAAGTGGAAGAAATCAATTATAATCATGATCCCTAAGCC
TGGGAAAGACAAAACACAGCCATCATCATACAGGCCAATAAGCTTACTTACT
TGTCTATCCAAGTTATTTGAAAAAGTGCTGCTGCTACGCATCAGCCCCCACCT
CAAAACACACAACACACTCCCATCGCACCAATTTGGGTTTCGGGCAAAACAT
GGAGCCATTGAACAGGTTAACCGCATCACAACAGAAATTCGCACTGCCTTTG
AACACCGTGAATACTGCACAGCTCTCTTCTTAGACGTTGCACAAGCATTCGAT
AGAGTATGGCTGGATGGACTTATGTTTAAAATAATCAAACTGCTGCCTCAAAA
CATACATAAGCTTCTAAAGTCTTACCTATATAAAAGAGTGTTCGCGGTCAGAT
GCAGTTCAAGCACTTCAAGCGATTGTACTATAGAAGCCGGAGTGCCTCAAGG
AAGTGTTCTAGGCCCAATTCTATACACCCTATACACGGCGGACATCCCAACAA
ACTACCAGCTAACGATATCCACATTCGCTGATGACACTGCAATACTAAGTCGA
TCCAGATGCCCAGTAAAAGCTACGATGCAACTAGCACGCCATCTAACTTGTG
TAGAACATTGGCTTGCAAATTGGCGCATACGAGTAAACGAAGGAAAGTGCAA
ACAGGTAACATTTACCCTAAACAAACAAACCTGCCCGCCCTTGGTAATGAAC
CACACGCGCATCCCACAAGCCGATAACGTAACATACTTAGGTATTCATCTGGA
CAAGCGGCTCACCTGGCGGAAACACATAGAGGCCAAGACGACGCACCTCAG
ACTAAAAGCAAGGGATCTACACTGGCTTATAAACGTTCGCTCTCCCCTAAGCT
TGGAGTACAAAGTCCTCTTATATAACTCCGTTTTAAAACCCATATGGACCTATG
GCTCCGAGCTATGGGGCAACGCATCGAGAAGCAACATCGACATAGTACAGCG
AGCACAGTCTAGGATTCTGAGAATCATTACTGGAGCTCCATGGTATCTTCGGA
ACGAGAATATACACAGAGACTTACAAATAAAACTTGTCATTGAGACAATAGC
AGAGAAGAAAGTAAAATACAATGAAAAACTAGCCTCACATCCAAATCCTCTT
GCAAGAAAACTTATTCGAGTATGCAGCCAAAGCCGACTGCACCGGAACGAC
CAACCAGCCCAGCGTTAAATTTGTTAGGCCACACAGCACGAATCATTTCATAA
AATGATATTTAGTTAGTTTAGAATAAGATTTGAAAACTTATTGTTAGTCTCTTA
AGTAAAAGGGAAGATTCAATAAATAAGCAAAACATGAAAAAAA

>DOC2_Jockey#LINE/DOC2_Jockey

GTCGCTGACGTGTGAAGACGTGTTTTCATCGAGCTCCGTATAAAATCGGTTGT
TTTTAGTGAAGTGAATGGTTATTAATATAATACAAACAAATAAATCGAAAGCG
AAAGAGACGCTCTGTGCGATGCACCATCGCTCGAATACATATTCTGTGTTTGA
AATAGTATAAGCAAGTAGTTTTACACCCAAAAAAAAAAAAAAAAAAAAAAA
AAACACTATGAGCCAGAACGACACTCGCGTTCAGCGACAACGCGAGCAAGA
CGATCGCCGGCTCTCATTGCAACGAAACAACGCGTACTTCTCTTACGTCTCCG
CGACTTCTGCAGACAGCGAGCGGTCAATCACCCATAACCCGACAAGTTTACC
ATTGCCAACAACTCAAGAAAGAGCGCGTTCTTGCTCTCCCTCACTACTGTCA
CAAAACCCCCAATCTCCAAACACTTGCGAAATTTCTTTACGCTTACCTACGGT
GACTAGTACTGTGACAACAACAACCACTGCAACTGCAATCACAACAACAAC
TGGAACGGTATCGTCAGCTCGCTCAATTTCGTCGACGCAAGCACTTGCTCCT
ACTGAGCCAGCGATCAAAACAAAAACACAATTTAACGGTTCTGCTGCGCTAT
CAGCAAGCCAAAACGTAAACAAGAACGCCGGGTCCACCATACAGACTGGAA

TGGATCGCTACATAACAATAAAAAGAAAACTTAGCCCTCAAAATTACAAAAG
TCAACAAACAGAAAACAAATCAAAAATTACACGCGGCAATGCTAACATGCTC
ACTAACAAAACGCCAGAAAACACCAACAGATTTGAGCTGCTGGCAAATTCT
ACTGATGAAAGTATACAACCGGCAAAGTCACCTAAGAAGGTCAAGCCTCCTC
CAATATACATTCGCGAAAAAAGCTCAAGTGCATTAGTGAACAAAATAGCCAC
ACTTATAGGAGAAAACTCATTTTACGTAATACCCTTAAGAAAAGGGAACATAA
ATGAAACAAAGGTTCAAACTGAAACCGAGGATAGCCATCGTCTACTAACCAA
ATTCTTGGATGAAGCAGGAAAAAACTTTTATACATACCAACTAAAAAGCGCA
AGGGGCCTACAGGTTGTTCTCAAAGGGATTGAGGCAACTGTTTCGACCACTG
AAATCGTAGAAGCGCTCAAGGAAAAGAACTTTAGTGCAAAAATGGTCATGA
ACATACTAAACAAAAATAAGGAACCACAACCAATGTTCAAAATCGAGTTGGA
GCCAGAGCGCCAGACACTGAAAAAAAAATGAAGTTCAACCAATCTACAAGT
TACAGCTCCTACTGCATCGGCGTATCACTGTGGAAGAGCCACACAAGCGCAA
TCGCCCAGTGCAGTGCACTAACTGCCAAGAATATGGCCACACTAAGGCGTAC
TGTACACTGAAATCTATTTGCGTAATCTGCAGTGAAGCTCATAGCACAGCGAA
CTGCCATAAGAACAAGGAAGACATCTCTGTCAAAAAGAGCAGCAACTGCGG
TGAAAGTCACACAGCGAACTGGCGTGGCTGTGTAGTATACAAGGAACTAAA
GAACCGCCTTAACATATGCGGAGAATCAATACGCGCTCAGACCACCCACATC
GCTCACACTCCGCCACAATCGGGCCCGTCCTACACTGCACCCCCCCCTACAC
ATCGTACCAGTCCTAGCGTCTCCTTCGCTAGTGCCTTAAAATCGGGAATTAAA
TCTGTGAATCCGATAACACAAAAGTCGACTTCTACTCGGGAAGAACAGAAA
ATAACTACCTTGAACGAACAAACGCAGCATATATCAACTGGCATTGAAACGAT
GATGATCTCACTCCAGCGAACCCTAAAAGAGTTTATGACATTCATGCAAACC
ACAATGCAAGAGCTTACAGCTTCTTCTGTCATCCAAATCAATATAATGGGTCC
ACTTCAAATATCTATGTGGAACGCGAATGGCGTTTCACGGCATAAAAACGAA
CTTGCCCAGTTCTTACTCGAAAAAAATATTGACGTCATGCTCCTCTCCGAGAC
ACATCTAACAGACAAGAACAACTTCCATATCCCAGGATACTTATTCTACGCCA
CAAACCATCCGGACGGCAAAGCCCATGGGGGCACTGGTATACTTATCAGAAG
TCGCATCAAACACCACCTTTATAACAGAACTGAAATGGACTACCTGCAATCC
ACTTCCATAAGCATGCAATCCAGCAGCGGCCCCGTCACTCTGGCCGCAGTCT
ACTGCCCACCTCGTTTTGTAGTTTCTGAGGACCAATTCTCGGAATTTTTCAAC
TCACTCGGTGAGCGATTCATAGCGGCCGGTGACTACAATGCCAAGCATACGC
ACTGGGGATCACGTCTTTTGACCCCAAAGGGCAAACAACTTWACAACGCGC
TCATTAAGCCGCGAAACAAGCTCGATCATGTGACTCCGGGTAGGCCTACATA
CTGGCCAGCAGATCCAAATAAGCTACCGGATCTCATTGACTTCGCAATAACAA
GAAAGATTCCAAGAAATAATATTACGGCCGAGTCACTTGCAGAACTATCATCT
GATCACTCTCCAGTTCTACTTACTCTCTGGCACCGACCACATATAACTGAGCG
GCCCTACAGGCTGACAGGCAACAGGACCAACTGGGCAAGGTACGCGAAATA
TGTTTGTACTCACATGGAACCAACCCAAACAGTATTCACCGACGAGGATGTT
GATCGCCTGGTCAAATCGATAGAGGAAACACTTGTTGCTGCAGCCAAGGCCT
CTACACCTCCAGASACACACAAAATGACAAATCATAGCAAGACAAATCGTGA
AATCGAACAGCTAGTACTTGAAAAACGAAGACTAAGAAGAGAATGGCAGAA
TCATAGATCCCCAACGGCTAAAGAACATCTAAAAATAGCAACACGTAAACTA

ACCAGGGCACTTAAGCTTGAGGAAGCCAACGCCCAGCTTCTATATATCAAAC
AACTATCGCCCACCAGCAAAAGGAACCCTTTGTGGAAAGCACACAAAAGCA
TACAGCCACCGGCAGAAACAGTCGTTCCACTGCGAGGTCCCTCTGGAAGCT
GGATACGCAGCGACGAAGATAGAGCTAATGCGTTCGCCGATCACCTTCAGAA
AGTATTCCAACCAAATCCTGCTAGCAACTCATTTGTCCTCCCTGTAGTAACTA
AAAGCTTGCCCCCTATAAATCCAGTAACATTCAGCCAAAGTGAGATAGCATCT
ATCATAAAAGATCTTAAGCCCAAAAAATCACCTGGACATGACTTGGTGACAC
CAAAAATGATCATAGAACTCCCACCGTGCGCGGTTCTGACCTTATGCCATCTC
TTCAACGCTATTACGAAACTTGGATACTATCCCCAAAGGTGGAAAAAGGCGG
TTATAGTAATGATTCCCAAGCCAGGCAAAGACAAAACGCAACCCTCATCCTA
CAGACCAATCAGTCTCCTAACGTGTCTCTCGAAACTGTTTGAAAAGGCATTT
TTAAAACAACTAACTCCCTACTTAAAAAGGCGAAATACAATACCATCGCATCA
GTTTGGCTTTCGCAAAAATCACGGAACGATCGAACAGGTCAATCGCATCACA
AACGAAATTCGTACCGCTTTTGAGCACCGGGAATACTGTTCAGCTATATTCTT
AGATGTTGCACAAGCATTTGACCGAGTCTGGCTGGAAGGACTAATGTACAAG
ATAACAAAACTGCTTCCCCAGAACACGCACAAATTGTTCGAATCTTATCTCTT
CAAAAGAGTGTTCTCAACCAGGTGTAACAGTTCGACTTCACACGACCGCGTT
ATCAATGCTGGAGTCCCCCAAGGTAGTGTACTGGGCCCCGTTCTTTACACCCT
ATTCACAGCAGACATGCCTACAAACTATCAGCTCACAACCTCTACGTTTGCTG
ACGATACCGCAATACTTAGCCGATCTAGATGCCCAGCAAAGGCAACAGAGCA
ACTTGCCTGCCATCTTAAAATAGTGGAAAGATGGTTTGCGGACTGGCGCATTA
AGATAAACGAGACCAAAAGCAGACATGTAACCTTCACACTGAACAGACAAA
CCTGCCCACCCTGTACCCTGAACAATACATTTATCCCACAAGCAGACGTAGTA
ACATATCTCGGCGTTCACCTAGATAGACGCCTCACCTGGCGGCGACATATAGA
ATCTAAGAGGACTCATATGAAGCTTAAAGCAGCCAATCTTCACTGGCTTATTA
ATTACAACTCTCCCCTTAGTCTGGAATACAAAGTACTCCTCTACAATACCGTG
CTGAAACCCATCTGGACATACGGCTGTGAACTATGGGGAAACGCTTCAAAAA
CCAACATTGAAATCATACAGCGAGCTCAGTCCACGATTCTGCGAACTATCACT
GGGGCACCATGGTACCTACGCAGCGAAAGCATCCATAGAGACCTCCACATAA
ATCTTGTCAATGAGGAAATTCAGATGAAAAAAAGTAAACATCAAGCAAAGCT
CGCCGCACACGAAAATCCTCTCGCGAAGTCGCTTACGCGAGTCTACAGTCAG
AGCCGACTGAAGCGCAAAGATTCTCCAGCCCAGCAAAGAAATCCTAGGGCC
GTCTCTAACTAATACAATTACTTCATATTGTAATTAATATAAGTTATATAATAAG
ATTTGAATAATTATTGTTAGTCTCACAAAAAGAGAAGATCCAATAAATAACGC
AATAAGTTTAAAAAAAAAAAAAAAAAAAA

>DOCJockey#LINE/DOCJockey

GACATTCGGCATTCCACAGTCTTCGGGTGGAGACGTGTTTCTTTCAAGCTAC
GAATAGCAAGTTCTAAAAACTACAACAGTATAGTGAAAGTTAAACACAAAGT
GTAAAGTGCAGTTTGCACAACTAACAATTATTGACTATAGTAATTATTTACTAA
AATAAATAATTATTCCATATTGTTCTGGTAATTGTTATATGTGGACTTAGAACAA
TGAATCAAAACGACATACGTTCTCAGCGACAATGTGAACAAGACGAGCGCC
GGCTCTCTTTACAACGCAACAATGCATACTTTTCTTTCGTCTCACCGCAAATC

GGTGATCGAGCACCCTCACCTTCAACTAACTCGAAACTTTTGCCCTCAGCGA
ACGACAGACCGCGTTCTTGCTCTCCCTCTCTGCCTGCTTCGGCTCACAAGTC
GTGGAGCGAAGAGACCGCCTCTCCTACCCCGCTCCTCTCGCAGCGCCAAAC
GACCGTCCCGGGTAACTGTAACACTGCAATAACGAGTGCAGTGACCTCACTG
GCAACTGCCACAACATCAACTTCGTCAGCGGCCCAACTAATTATCGCTGTGC
CAGCTGTAAATAATTCAGCAGCACTGACCGTTTGCAACAACAATAATGCACG
TAAAGAAGAATCAAAACAAAAGCAGAAGTCGATTTCGACTGTGCAGACTGG
CATGGATCGCTACATCCAAATCAAGAGAAAGCTCAGCCCTCAAAACAATAAG
GCAGGTAATCAACCCAAAATCAATCGAACCAACAACGGCAATGAAAACTCT
GCAGTAAATAATTCAAACCGATATGCTATCTTGGCTGATTCTGCGACCGAACA
ACCCAACGAAAAAACGGTAGGGGAACCAAAAAAGACCAGGCCTCCACCAA
TTTTCATACGAGAACAAAGTACAAATGCACTTGTAAATAAACTCGTTGCTTTG
ATTGGTGACAGCAAGTTCCACATTATCCCACTTAAAAAAGGAAATATTCATGA
AATAAAACTACAGATCCAAACAGAAGCAGACCACCGTATAGTGACTAAATAC
CTAAATGATGCTGGTAAAAACTACTACACATACCAATTAAAAAGTTGCAAAG
GGCTACAGGTAGTACTTAAGGGCATTGAAGCAACAGTGACACCAGCTGAGAT
AATTGAGGCTCTGAAGGCCAAAAACTTTTCTGCAAAGACAGCTATTAATATTT
TAAACAAAGACAAAGTTCCGCAGCCACTATTCAAAATAGAACTCGAACCAG
AGCTCCAGGCACTAAAGAAAAACGAAGTGCACCCAATATACAATTTACAGTA
CTTGCTACATCGGAGGATCACCGTGGAGGAGCCGCACAAACGTATCAATCCA
GTTCAATGTACTAATTGCCAAGAATACGGCCACACCAAGGCATACTGCACCCT
TAAGTCCGTATGTGTTGTCTGTAGCGAACCTCATACTACCGCAAACTGCCCCA
AAAACAAGGACGATAAGTCTGTGAAGAAATGCAGTAACTGCGGGGAAAAAC
ATACTGCAAACTACAGAGGCTGTGTGGTGTACAAAGAATTGAAGAGCCGCCT
AAACAAACGTATTGCCACAGCACATACATACAACAAAGTCAATTTCTACTCTC
CGCAACCGATTTTTCAACCACCCCTAACTGTCCCAAGCACTACTCCAACAATT
TCTTTCGCTAGCGCCCTAAAATCCGGACTAGAAGTGCCCGCCCCACCGACAA
GAACTGCTCATTCCGAACATACACCGACAAACATCCAACAAACACAACAAA
GTGGCATCGAAGCTATGATGCTATCCCTACAGCAAAGCATGAAAGACTTCATG
ACGTTCATGCAAAATACTTTGCAAGAGCTCATGAAAAACCAAAATATCCTGAT
TCAACTTCTTGTATCTTCAAAATCCCCATAATGGCTTCCCTACGGATATCTCTG
TGGAACGCAAATGGCGTTTCACGGCATACACAAGAGCTCACACAGTTCATTT
ACGAAAAAAACATCGACGTAATGCTACTATCAGAAACGCACCTCACAAATAA
AAACAATTTTCATATACCAGGATACTTGTTCTATGGTACAAATCATCCAGATGG
TAAAGCTCATGGAGGCACTGGAATACTCATCAGAAATCGCATAAAACACCAC
CACTTAAACAATTTTGACAAAAACTACTTACAATCTACGTCCATAGCCTTACA
ACTCAACAATGGTTCAACGACTCTAGCCGCAGTCTACTGCCCACCGCGCTTT
CCAATCTCTGAGGATCAATTCATGGAATTCTTTAACACACTAGGTGACAGGTT
CATCGCAGCGGGTGACTATAACGCCAAGCACACCCATTGGGGATCTCGACTT
GTGTCGCCAAAGGGTAAGCAATTGTACAATGCGCTTACGAAGCCAGAAAAC
AAGCTAGACTATGTATCCCCGGGTAAGCCTACATACTGGCCAGCAGACCCAA
GAAAAATCCCAGACCTGATCGATTTTGCAATTACTAAACATGTCCCCCGCAAC
ATGGTCACCGCCGAAGCACTAGCAGATTTATCATCAGATCACTCACCTGTTTT

TCTAAATATGCTAACTCGCCCCCACATCGTCGACCCACCGTATAGACTCACAA
ATTTTAGAACAAACTGGCCAAGGTATCAAAAGTATGTCTGTTCACACATAGA
ACTAACGACGGCATTATCTACAAAGGAGGATATAGACAAGTCAACGGAAACT
CTTGAAAACATTTTAGTTTCGGCTGCAAAGGCTTCAACCCCGCCAGTGACGT
ATGCAAAACCAAACTACATCAAAACTAATCGCGAAATCGAGCGGCTGGTATT
AGATAAACGACGCCTACGAAGGGATTGGCAGTCTAATAGATCACCAATTACTA
AGCACATGCTTAAGATAGCCACACGCAGGCTTACCAATGCTCTCAAACAAGA
GGAAAAAAACAGCCAACGTTCATATATCGAGCAACTCTCTCCCACCAGCACT
AAGTACCCTCTTTGGAGAGCTCACAGAAACCTAAAGACTCCAATAGCGCCAA
TTATGCCACTCCGAAGTCCCTCTGGCACCTGGTTTCGAAGTGATGAAGAAAG
AGCCAGTGCTTTCGCTGACCATTTACAAAATGTATTCCGACCAAATCCCTCTA
CCAACACATTTATTCTCCCTCCTTTAATAGCAGCCAATCTAGATCCTCAAGAA
CCCTTTGAATTCCGACCATGTGAACTAGCAAAGGTTATCAAAGAGCAACTGA
ACCCAAGAAAATCGCCTGGCTACGACCTAATAACTCCAAGAATGCTCATTGA
ACTCCCAAAGTGTGCTATTCTTCACATCTGCCTGTTGTTCAACGCAATCGCCA
AGCTTGGATACTTCCCTCAAAAATGGAAAAAGTCGACCATAGTAATGATTCCA
AAGCCAGGAAAAGATAAAACGCAGCCATCATCATATAGACCGATAAGCTTAC
TAACATGTCTTTCAAAGCTGTTTGAAAAAATGCTACTCCTTCGGATTAGCCCT
CATCTTAGAATAAACAACACACTTCCAACACATCAATTTGGCTTTAGAGAAA
AACATGGAACCATCGAACAGGTCAACCGAATCACGTCAGAAATTCGTACTGC
TTTTGAACATCGAGAATACTGCACAGCCATTTTTCTAGACGTCGCGCAGGCAT
TTGACAGAGTGTGGCTCGATGGACTTTTGTTTAAAATAATCAAGCTGTTGCCC
CAAAACACACATAAGCTACTGAAGTCATACCTATATAACAGAGTGTTTGCAAT
AAGATGCGATACAAGCACTTCACGCGATTGCGCAATCGAAGCTGGAGTGCCG
CAAGGCAGTGTACTGGGTCCAATCTTATACACCCTGTATACGGCGGATTTCCC
CATAGACTACAATCTAACAACCTCCACGTTCGCTGATGATACCGCGATACTCA
GTCGCTCGAAATGCCCAATAAAAGCCACGGCACTCCTATCCCGACACTTAAC
ATCTGTAGAACGATGGCTTGCCGACTGGAGAATTTCAATAAATGTTCAAAAAT
GCAAGCAGGTTACCTTTACCTTAAACAAACAAACATGCCCACCACTGGTCTT
GAATAACATATGCATTCCACAAGCCGACGAGGTAACATATCTGGGAGTTCATC
TGGACAGGCGGCTCACTTGGCGCAAACATATAGAAGCCAAATCGAAACATCT
TAAACTTAAAGCAAGGAACCTCCACTGGCTCATAAATGCTCGCTCTCCACTTA
GTCTGGAGTTCAAAGCTCTTCTATACAACTCCGTCTTAAAACCTATCTGGACT
TATGGCTCCGAGCTGTGGGGCAACGCATCCAGAAGTAACATAGACATTATTCA
GCGAGCACAGTCAAGAATTCTGAGAATTATCACTGGAGCGCCGTGGTACCTT
CGAAACGAAAACATACACAGAGACCTAAAAATCAAATTAGTAATCGAAGTAA
TAGCTGAGAAAAAAACGAAGTATAACGAAAAGCTGACCACCCATACAAATC
CCCTCGCAAGAAAACTAATCCGAGTATGCAGTCAAAGCCGGCTGCACCGCA
ACGACCTCCCAGCCCAGCAATAAACTTATTAGGGCATTAATGAAAAAAAAAA
ACTATCACTAAGTGAAAGTTAATTAAGTTAGATTAAGATTTGAACACTTATTGT
TAGTCTCTTAACACAAAGGGAAGATTCAATAAATAATAAAAATTAAAAAAAA
AAAAAAAAAAAAAAA

>DNAREP1_Helitron#DNA/DNAREP1_Helitron

TTATACCCGTTACTCGTAGAGTAAAAGGGTATACTAGATTCGTTGAAAAGTAT
GTAACAGGCAGAAGGAAGCGTTTCCGACCATATAAAGTATATTTATATATATAT
ATATATTCTTGATCAGGATCAATAGCCGAGTCGATCTGGCCATGTCCGTCTGTC
CGTATGAACGTCGAGATCTCAGGAACTATAAAAGCTAGAAGGTTGAGATTAA
GCATACAGATTCTAGAGACATAGACGCAGCGCAAGTTTGTTGACCCATGTTG
CCACGCCCACTCTAACGCCCACAAACCGCCCAAAACTGCCACGCCCACACTT
TTGAAAAAATTTTTGATATTTTTCATATTTTTATTAGTCTTGTAAATTTCTATCG
ATTTGCCAAAAAACTTTTTGCCACGCCCACTCTAACGCCCTAAAGCCGCCCA
AAACTGTCACGCCCACACTTTTGAAAAATTTTTAAATTTTTTCTCATTTTATTC
CCCAATATCTATCGATATCCCAGAAAAATTATGAAATTTCGCGTTCGCATTCAC
ACTAGCTGAGTAACGGGTATCTGATAGTCGGGGAACTCGACTATAGCATTCTC
TCTTGTTT

>TOM1_LTRGypsy#LTR/TOM1_LTRGypsy

AGTAACGTAATTATGCCTTCCTCATAATTCACTTATACAAAAGACCGACCGCG
GTGGTCCGCCGTATYGATTCCTCGTTAAATAAACAAACATTTCTCGATATTGC
GCATCCACTCAAGCCGTAATCAAAACCCAGCCACTAGGCAGGCACATAGCCA
CGTAGCACATAAGAATTCTTTTTCATATGTATGCACATAAGCATAAGCAAGAA
GCGCTCTCGCGCAGATCAAGTAAACAACCCACTAACCCAGTACATCTAAGAT
TTTCTCACACCAATTGTTTCAGTCTTAAGCTGATATTCAATCAATAAAAACGC
AAAGCGGATTCATTCGGAGTTTTATTTATTTCGGCGTTGATCTTAGTTCAAAT
ACGGATCATTTATTCGACTCAAAACAAAAACTATTTTACT

>RT1CR1#Non-LTR/RT1CR1

GGGGACGCGCGCGAGTAGCCGTGTGAATCGAGTTGGCGCTCCTGGAAAACC
CCAAAATTTTCCGTGTCGCGGCGGCTTGATGGAGACAGGAGTGGCTGTCTCT
AACACACAGTGCCGATATGAGTACTCACCAGCAGTGGGCAAGCTCCAAGTAT
CCATGGATCGCCCGCCGGCGCCGATCCTCAGGGCCATGGAGCAGGTCAGGGT
GAGAGAAGCAGCGAACTTTCTCTGCACTACTTTCTCTTTGTCGCTGTGTTGTT
TCTTTTGGCGTCTTGGCTGCCACCAGACTTGCCGGTCACGCCAGCTGATCTC
GCAGTCAGCTTGGAAAGCAGTGCTGGTAAACGGCTAAAGCACCGCGGTCTG
GCAGCGCTGGTCGGATGCACGAGTCCCCTAGCACAGGTGAGCCGTTTTCTCG
GTGAAAGCACTCACTGACAAAAGCTAGCAGCTACGGAAATCGCCTAACGATC
GGTTCACCGGTCGTAGCATACATTTTTGGGCGGCTGACGGTTATCCTTGGCAA
CATCCCATGGAAACCCGGAGACAGATCCATAATCGAGCAGGAGAGGCAGAA
CAAGAGTGTAAAAAGTCAAGGAGCAGACCAATAGCCAACCCCCCCGCTTAG
GCGGCACACTTTAAATCCGGGGCTCTGGCGACCAAGCTGGAGCGGTATATCT
CCCACCGTCGCAACTTTCCCCTGCTCACCCACCCCATAAGTCCACGGCGCGT
CTGACCCAAAGTGGGCGGGTACGTGGCCTGCGCCTGTTCTCTTTACCAGCCG
GGCAGCAGCAAAATAAAAACCCATTCAGCGTCTGAGCCAAGTTGGCCGGCG
GAATGGTCTGCGTCTGCGCCGCATCGGCCGGCAGAATATCCAAAACCTGGCG
AAAGGTATAAAGCCGCAAACCTGGCAGGAGGTATAAAATGCAACACCTAACT
GGAATGTCTGTAAACAACTTTATTACTCCAGGTGGCAGCCACACATGTGGAA

ATCCACCCTCGGCTAATCCCGAGGCTAGGGCACGGATTCTGGAGGCCCTACC
AAACATTCTCCCCAAACCCGCCCCGACGGCGGCACACTTGGCCGTCGGACTA
AGCAATGCAACAAGTGCACCCACAACGGACGAAGAAAGCTTGGTGTTTGGG
AAAAGGTCAAAGGTCCTAAGGACCCCACCCCAGAACCCGAGCGATGGCACC
CCCAAGAGGCCATTAGAAGCGACATCCCCCCTACCAGAGCCCAACCAGCAA
AGCGGGTAAAGACCCCCCAGCTAGAAATCGAGGAGATGGGAGCAATCCTGG
ACGACCTTCTGACGAAGGTCAACCACAACGGGGTGAGGAGCGTCAATCAGG
CAATGAAAAACTCATTCGCCAGATTGAAGGAGCTCCAACTAAAGCTGCGCAC
AAGGCTGCCGGAGGCGGAGAATTCGCATGTCGGACGCACCGCAAGAGCCGA
TGCTAGCCAACAGACCACCCCTAAGCGCCCCTCCAGCCACGAAGAACCGAG
TAAGGGTCCGCGAAGAAAGCCCAGCGATCCGACCACCAGAAGGCAGCGGG
ACCCAAGCGCCTACCAAGGGCTCCGCCCCAAGCCACTGCAGCGGGAAACAA
GACGCAACCAAGATTGCAGCATCCGCCCGGACCGCGCAGGAAGAGACCGCC
AAGGGAGAGGCCAGATGCGCTGGTTATCACCCCCTCAGCTGGCTTACCGTAC
AGCGAAGTGCTGTCGCTGGTCACAAGAGGGCAGGACGCCAGGCTCAGGGCC
ATCGGGGAGAACGTATCAAGGGTTAAGAGGACGGCCAAAGGCGAACTGCTC
CTCGAGCTACGTGCCTCTGCCCAAGACTTGACGCAGAAGCTCAAGATGGAC
ATGGGAGCGGTGCTAGGAGACCGCGCCAGCCTTCGCGCGTTAACTCAATCCA
AAGTATTTTTGATTCGCGACCTCGACGAGCTTACTACTGAAGACGAGCTGAG
GAGGGTCCTGGAGTCCCGGCATAGATTCCAGCAGCAGTGGTGGCTATCAAGA
GCCTCCGTCAAACGCAGTATGGAGGGAAGTCTGCTATAATAGCAGTTCCAGC
CAATCTGGCGGACCCGCTGATCAAGCGTGGCAAGCTGAGGGTAGGATGGTCC
CAATGTCTGATCAAGGAACTGGAGCCACGCCAAAGATGCTTCAAATGTCTGG
AGGAAGGCCACATAGCGGCCCATTGTAGAAGCGCCGTCGACAGAAGCCAGT
GCTGCTTCAGATGCGGGTCCGCGGGACACAAGGCCGCAGAGTGTCCCAACG
AGGCTAAGTGCTTTTTGTGCGCAAGCAGAGGAAGCCAAGCGACCAACCACC
AAGCAGGCACCCGGAAGTGCCCATTGGCGGGCAAAGGAGCACCAAAGGCA
CCACAATGATGCGTTTGATTCAGCTAAACCTGAATCACTGCACGGCAGCCCA
AGACCTGCTAGTGCAGACGGTGCGCGAACGCAGAGTGGAGCTTGCGTTACT
TAGCGAGCCCTACCGGACGGCGGACAGCCCAGACTGGGCTTTCGACCGCGC
CAAGAAAGCAGCAATCTGGAGGTGCAGCAGAGAAGCCCAACAATTAACCGA
TGTTTTTTCGGACATCGGGTTTGTTAGGGCAAAGGTGGGCAGATGGTGGGTG
TACAGCCGGATGCTAGAGGCCGCACCCAGGTTCTCATAGCTGGCGACTTCAA
CGCATGGTCAGAGAGCTGGGGCAGTTCAACCACCAACGCGAGAGGCAGGAT
GGTGCTCGAGGCATTCGCGACGCTGGACCTGGCTCTATTAAACCAAGGGAAC
CGGCACACGTTCAGGCGTGCCGGACTGGGCTCTGTGGTGGACCTCACCTTCA
CTAGCGGCTCGTCGTTCAGGCTAACGAGGTGGAGACTCAGCGAGGAATACA
CTGGCAGTGACCACTTGGCCATCATTTGTGATCTGGGATGCCCTTCCTCGACC
CAAGCCCAGCTAGCAGCCCAAGCCAGGATAAAATACAAAACGGACACCCTG
GACACGCAGTTATTCCGAGAGCAGTTCCTACCCTCGGTGAGTGGAGAAGGA
GCTGAGCTGACGGCAGTGGCGCTGATGAGGCAGCTGAAGACCGCGTGCGAC
GACAGCATGCAAACAAGCAGGACACATAGCCAACAAAGAGCCCCTGTCTAC
TGGTGGAACCAGGAGATAGAGACGGCTCGCCGAGAATGCCTCTCCGCCAGA

CGTCGCTATCAACGCGCTAGAGGTGCGGAGTCCTTTGCCGAACGCCAATCCG
AGTATAGAGCCCGCAGGAAAGCACTCAAGCTAGCCATACGGGAGAGCAAGC
GGAAATGCTTCCTCGACCTATGCGATTCTGCTGACAGCGACCCATGGGGAAG
TGCCTACAAGGTGGTGGTCAAGCAGGCATATACGAGGACTCCCAAGCTACTG
GACCCAGCGATGCTCCGCAGTGTAGCGGAACATCTGTTTCCTTTGATGGACA
GGTTACGCCCCGCCGACCCAGCCACAGGGGACCACGTCGAAGCCGACGCCA
CGGTCAGCAGTGAGGAGATCCTGGAGCTGGCGAAACTGCTGAAGGACGGCA
AGGCCCCCGGGCCCGACGGCATTCCGATCAGGGCGCTTCGGCTCTCTCTACC
TCCAGCCAACTCGTTTGCGAAGGCATTCACCAAGTGCCTGACGGAAGGAGT
CTTCCCAAGTTGCTGGAAGGTACAAAAGTGTTGCTCCTCCCAAAACCAGGG
AAGCCACCCGAGGAGCCTATATCGTTCCGGCCGATATGCCTCATCGATGGAAC
TGGCAAGCTCCTGGAGAAACTGGTGTGCATTCGGCTAGAGAGGGCTATCGCA
GACGCGGGTGACCTCTCACGGTCCCAGTTTGGCTTCAGGAAAGCGCGGTCC
ACCGTCGACGCCGTCAACAGAGTGGTCGAAGTAGCGGCCCAAGCAATCGAG
GGCACCAGATGGAAGGGGGGTAGCAAAGAGTACTGCCTCATGGTCACACTA
GACATCAGGAACGCCTTCAACACAGCGAGGTGGGACCGGATCCTAGAAGCA
TTAATTGGCTTCGGCGTCCCAGCCTACCTAGTCAGGATCATTCGCAGCTACTT
CTCGGATCGGGTCCTACTGTGCGAGTCGTCGGAAGGAGTCTTCAGGCACCAG
GTCACCGGTGGAGTCCCGCAAGGCTCGGTTCTCGGCCCGCTTCTGTGGAACA
CCATGTATGACGGGATACTTCGCCTACCGCTGGTTGGGAGGAGCGAGATCGT
AGGCTTCGCGGACGACGTGGCTTTGCTCGTTGTTGACAAGCATCCCGGCAAG
GCTGAGGAAAAGTGTAACCAAAACATCAGGGCCATTGAACAATGGCTCAGC
TCAATGGGGCTAGAGCTGGCGCCGGAGAAGACGGAGGCTGTCCTGATTAGC
TCCAGGAAGGCAGTGGAGACGGCCACTGTCGAAGTAGGCAGTACCTCGGTG
TCCTCCTCTCGGGCAATTAAGTACCTGGGAGTCATGATAGACACCAGGCTTTC
GTTCCGTGAACACCTAGCCTACGCTAGCTCGAAAGCAGCAGGAGTAAATCGG
GCGTTATCAACCATAATGTTGAACACCCGAGGCCCAAAACAGGCGAGTAGAC
GGCTGCTGACGAGCGTCACCCGCGCGACCATGCTATATGCCGCCCCGGTGTG
GGCAAAAGCGCTTGAAACAGAAAGCTACGCCCAGGGCCTAAATGCTACCCA
TAGACTGTCGGCACTACGTATCTGCTGCGCCTTCAGAACAGTGTCCAACGAG
GCGGCATTGGTGATCGCAGGCATACCGCCTCTAGACCTATTAGCGCAGGAAA
ACAAAGTGGTCTTCAACCAAACCCACGGTAGAAGCCTCTCCCCCTGCGCCA
AGAGGTCAATACGAGCAGAAGCAAGGCAGCACACCCTGGAGACATGGCAGC
ACAGGTGGAACAACGAGCTTAAGGGTCGCTGGACGCATCAGCTGATTCCTAA
TCTCAAGCCGTGGATAGAACGGAAGCATGGCGAAATAACGTTCCATCTAACG
CAGCTGGTCAGTGGGCACGGTTGCTTTCGCAGCTATCTGAAGCGCTTTGGAC
ATGAGGAGGCGGATGATTGCCCATGGTGCGGAAGTGGACGGAGCGAAACCG
CAGAGCACGTTCTCTTCTCGTGCGATAAATATGCAAGGGAACGGAGCTCTCT
GGAAACCGTACTTGGCAGCAGACTGAATGCGGATAACCTAGTCCCATTCATG
CTGCAGGGTGAGGCGGAATGGCAAGCGGTCAACAGCTTCGCCGCAGCGATA
ACTACCGAGCTCAGAAGAGCCGAGAGAGCCAGGAGGGTCCACGAGTAAGC
AAATCTTGATCGTCCTCGCGAAGCAATGCTTCACGGCCATACCGCGGGGGGT

TCTGGATTTGCCCTCCTTACTGTTACATTGTTATATTAATTAATCTTAATCTTAA
ATGTCTAATAAACGAATTAAAAAAAAA

>RT1BR1#Non-LTR/RT1BR1

ATAAAGCAGCTGATAGCGCTGCCAACTTGGCGCAGCGGGCGATCTGGAGAG
GGAGGCGTTGCCAGATCAACCGTGTGATCAGTGTTGTGTTGCGACAGTAGAG
CGCGAAATTCAAAATTAAACTTAAAATTAAACCGTTGAATTTTAATTTAATTTT
AATTTTGATTCTGGTTACTGGGAGCGCACAAGTCTTTAAGAAAGGGGAAAAG
GCACAAAGGTCGAGGACAGACCAAATATCATCCTCTCACCGTGCTGGTGCCT
AAGCAGAGGCTCTGGTGTCCGAGTCGAGGCGGTACAAACCTCCATCCGTAA
AAGCCAGGGGGAAATCCGTGGCCTACGTGGGAACCCCTTTCTAAGGCTGGT
GCTTCGCCTAGGTAACCTTAGGCCATCCTTCAGTTGATGGACAGCGCAGTGG
GGGGCAGTCCCCCTGATCGGAACGACCCGTTCAGGAGGAGCCCCCGACTTT
CGAGATCCCCCACCAGAGGAGTCGGTAACCCGACCCATGGTGACGGAATCTC
GGTCCCGTCGGTGGCAAGGGGTGAGCACGCAGGCCCCCAGCCGCCGTTGAC
TAGCGACTGCAGCGATGCTGTAGAGGTGGAGAACTTGGCTCCTACAGCATCT
ACGACCGCGACAAGTCAGAAGACGATGGACCACATCGCCGTGCCATCGCAA
AAGACGGGAGGGAAGTCCCCAGCTGGTTCCCCTCATCGGTCTTCGGACCTG
ACCGCGGCTCTCCAGAATGAGGACCTGAGGGGCATACTTACCAGGATGAATG
GCAAGATCAGCACCTTGCTATCCGCATTCGAGACCCAACGGCACGTGACACG
AGAGACAAAGGGCATAGCTTCCGAACTCTCAGCCCTTAACAACAGGGCGTTA
GAGCTGTACAAAGGTACTACTAGCGAGCACCGCTTGAAGAGCAGTGCTACCC
AGACGGAGGTACAAAAGCCTACCAAAAAGGCTCCTCAAAATGCGCAGGTAC
CCAAGGTGCAAGTGCCAAAAGTCCAGGCGCCCAAGCCGAGCAACCCTACGT
TGGGTGGCAACGGTGAAGAAAAGGCGACATCCATACCAAACGAGTCCAAAC
CAGGCAGCTACGCATCTGTCGCCAAGAATGCTAGCACGGGTGCAGAGTGGA
CCAAGGTGAAGCCTACGCGCCTGCGTAAAAAACCGGAGGCACTCATCGTAA
AAAAGACAGGAGAGGCTTCGTACGCAGAGATGCTTCGGAAGCTAAGATCGG
ACCCGAGCCTTAGCGAACTGGGCAGCCACGTGCGAAAAATCCGGAGAACGC
AGAAAGGTGAGCTGTTGCTCGAGGTAGAGGGGAAAGCTTCGGAAAGCGTTC
CCAAGTTTAAGAGCGACCTGGAAGCGGCGCTCAATGACTTGGCCTCTGTGCG
CACAGGAGCGCAAAGAATAGCTCTATCTTGCAGCGGATTGGACGAGGCTACG
ACAGCAGAGGAGCTCCACAGCTGCTTGGTCGCCCAATTCCAGGGCCTGCAG
ATAAATCCTGAAGATATCAGGGGCCTTCGCAGAATGCGGGATGGCACGCAAA
TAGCCTCAGTGCTGCTGAACGCGAACGTTGCGATACCAGTCCTTAAACAGGG
CACCATAACCGTTGGATGGTCAAGATGTCGTATCACCCAGGACGTCCGACCC
ACGAGATGCTACAGGTGTCTCGGCTATGGGCATCGATCAGCAACCTGCAAGA
ACACTGACAGGGCAGACTGCTGTCTTAGATGCGGTGAGCGTGGGCACAAGG
CAAAGGGGTGCGCTGCAGCACCAAAATGCCTGATCTGCAGCAGCGAGGTGG
ACAGAAACCACTCGACGGGTAGCTTTGCGTGCCCGACCTACAGAGCGACCC
TAAAAGAAGCCAAGAGCCACCTTAATGCACACTCATATTAGCGTAGTACAGC
TCAATGTCAATCATTGCGCAGCAGCTCAGAGCCTCCTGGCCCAGACTGCGGT
TGAGCGCAATGTAGACATCATGCTCCTAAGCGAACCCTACGTCTCTGGTAGC

GGACAATCGTCCATGATCCTTGACGAGACAGGTAAAGCAGCTATCAAATGCT
GCAGCTCTCTCCACGTCGAGGAACTGGCTGCTTTACCTATGCGGGGTATCGCT
TATGCGAAGTTAAAACACGTGCACTTGTACAGCTGCTACGCTCCGCCGAGCG
ACACCCCCGATCAGTTCGAGGAGTTTCTGGAGGCGCTCGTGGACCATGCGAG
AGGGCGAAGCCCGAAGGTCATTGCCGGCGACTTTAATGCCTGGGCAGTGGA
ATGGGGCAGCAGGACATCCAACACCAGAGGCCGAGCTGTGATTGACGCCAT
GGGAATGCTGGACCTTATACTGCTGAACGACGGACGGAAGCCGACGTTTAAC
AACGATAGGGGTACGTCCTTTATTGACGTTACCTTTGTCAGCAGAGGGCTAGT
AGACAACAACAACTGGATGGTCCATGACGTCATGACGCTGAGCGACCACGC
CCTGATCTCCTTCAGTCTCTCCCCGGAGGGCATGCCCAGGAGACGGCAGAGT
AGAGCAGTCGGGAAAGCATGGGACACCAGGAAGATCGATGAGGCCATGCTG
GCCTATCAGATCAATTCCCTGGAAATCCCAAGTGGGGACGCAGAGAGTATGG
CGGCAGGCCTCATGAATATGCTGGGAAGAATCTGCGACGCAATCATGCCAAG
GAAAAATAAGGCACAGCGCAAACCACCCGTTTACTGGTGGAGCGCCTCCCTA
AGCCAACTACGGTCTGATTGCCTCAGGGCTAGGAGAATGGCGCAACGAGCC
AGAGGCAGTACCCACCACGCGGAACTCTTGGAGGCTTTCAGAAGGAAACGT
CTAGAGTTCAAGCACGGCATCGCGGCTGCCAAAGCGCGGTCGTTTAAGGAG
CTGCAGGATGGCGTAGACAGCGATACCTGGGGCCTCGCCTACAAGCTTGTTA
CCAAAAAGCTAAGGAGGAGAGCGGCAACCCCATCCGACCCGGGGGTCCTGG
CTAACATAGTAGGGGAGCTATTCCCAAAGCAGACCACACTATGGAGGCCAAC
AGAGGCAGCCCCTGCCCCAGATTTTCCGTGCGTCACAGAACTTGAAGTCGCC
GAGGCAGCCAAGCGCATCAAACCCAACAAAGCCCCTGGACTAGATGGTATTC
CTGGAGCTGTTATAAAAGCAGTGGCGCTGGGTAGACCTGAAATCTTCAGGGC
CACCTTCCAGCAATGCCTTCTGGACGGAATCTTCCCAACAAGGTGGAAAAGC
CAGAAGCTAGTCCTGTTGCCGAAAGGCAAGGGACCAGCACATGCTGCAAAC
AGCTACCGCCCTCTATGCCTACTGGATATAGTAGGAAAACTGTTCGAACGTAT
CCTGTATACCAGAATAGAGGCAATCACCGAGAGCATCAACGGCCTGGGAAGC
CATCAATATGGCTTCCGGAAAGGTAAGAGCACTCTGGACGCTCTTTCGGCCG
TTTGTAACATCGCCAAGACCGCTATTTCTGGTGATAGATGGTTAGGGGGCAGG
AAGGAATACTGCGCAATTGTGACTCTGGACGTAAGGAACGCTTTCAACACCG
CCAGATGGCCCGTAATCCTCGCGGCCATGTACCGTATGGGGATCCCGGAGTAC
CTAAGGATAGTCGTTGGCAGCTACTTTAGGGACCGGGTCCTATGGTACGATAC
GGAAGATGGCCCAAAAAGATACCGAGTTTCGGCAGGTGTTCCCCAAGGATC
GGTACTTGGACCAATCCTATGGAACATTATGTACGATGGGATCTTGGGCATCA
ACAGGCCCGTAGGAGTAGAGCTGCATTGTTTTGCTGACGATGTGGCAATCAC
AGCTGTCTCGAAAACAATCGCAGGGTTGGAAGACAAATGCAACTCTACGATC
GGTGCTGCCATCCGCTGGCTCGAGAAAGCCGGGCTAGCAATAGCGGCTCACA
AGACCGAAGCAGTCCTACTAAGCAGCAGGAAAAAGGTGGAGAACATGCTGG
TCTCCGTCAAGGGTACACAGGTGACCTCTCAAGAGTCCCTAAAGTACCTGGG
GGTAATGATAGATCGCAGACTATCGTTCAAGGACCACGCGAGCCACGCCAGC
AAGAAGGCAGCAATCACAGCCTCTTCGTTGGCGAGGCTTATGCCCAACGTCG
GAGGCCCAAGACACCCGGCCAGGAAACTGCTGGTGTCAGTAGCAAAGGCTT
CGCTACTATACGCTGCACCAGTCTGGAGCAATGCCACTGGCAGGGTCTCATA

CCTGAAAGGAGCTCGTTCGGTGCTACGGTCAATGTCTCTGAGGCTCATTAGA
GGTTTCAGGACCATATCCGAAGACGCGGCGCTAGCGCTGGCAGGCCTGCCGC
CGATTGATCTGGAGATCAAGGCTCTCAGCCTAATGCGGAGTGGCGCTTCCAG
GCAAGAGGCACACGAGTGGCTATTAGGTGAATGGCAGAGCAGATGGCAAAC
GTCGCGACGGGGGAGGTGGACTTATCAGCTCATCCCAGAGATGACGGTTTGG
GCAGAGTGCCAACACAAATGCTTGGACTACCACCTAACCCAGTTCCTCACGG
ACCATGGCTGCTTCCGGGCCTATCTACTCCGGTTCCGTCACGTAGAGTCAGCC
CAATGCTTGTTCTGCGTCGACGGTGAAGAAACAGCAGAACATGTGCTAATGC
ACTGCTCCAGGTTCACGGCGGAGAGAGAGCAGCTAAAGACGCTGTCAGGTT
CCCCGTTCAGCCCTAGTGGCTTGTTCGCGGCTATGCTGGCGAACAGGGGGGC
TTGGGAGCGGGGACACAGCATTATCATTAATATGATGAAGCGTGTCCGATCAG
ACGAGATGGCCAACAGAGTGGATGTCTAAGCCCAAACTGGTGTCCTGGGTG
ACGGCGGGCGAAGAATTCATCCTCAGCGTCCCCGGCTCGTCGTAAAAGGCG
ACTAAAGGGTGGAAGGAGGAGCCCCCATGGACTACACTGAAGGAAGGGAGT
GCGACCTGGCCTCACATCCTGCTCACCGAAGTCATACCTTGACTGGCAGTCC
CGGTGAGCGAGCAAGGACTGTAGAGCACGCGGAGGTTTTTGTTTTAGTACGT
AGGCATAATTCCAATAGGGCTTATGAATCGTGCATGCCACCTACGGACGGTAG
GTGGTATCTTTAGAAGATTTTAATTTTCCTACCGTAAGTCAAATAATAAAAAA
AAAAAA

>RT1AR1#Non-LTR/RT1AR1

TCGAGTTCCCGCGTATTTCGCACGTCTTTTTGCTGCGCTGCGCGAATTTCGTC
ATATACGAGTCCGGTCAGCCGTAAACTGCAACATAAACCTGCAAATCCACTCT
GGGGTTTGCGCCGCGTTTCTGAGCGTCACGGTGTCGTTCCGGAATTCACGAG
TGATCCAGTAAACTGGTAATAAATCAGATCAGCTGCATCAGACAATACAGCTG
ATAGCATTGCCAACCTTTCGCAGTCGACGAGCTGGTAAGACTGGCGTTGCCA
GATCAGCGGTGCGATCGTCATTCGGCCGCGCGCTCGTTGGGCGCGAAATTCG
AATTCAAATTCAAATTTGAACTAATTAAGTTGAACAAAAATTTGAAATATAAC
CTAAGAACTGAAGCTAGTTCTATTTCCCACGGAGCGCTGGGGACCAACAAGC
CAAAGTCCCCCTTTTATATCTGATGGACAGAACAGGGGGGGGCAGTGCCCCC
GACCCCAACGACCCGTTTAGGAGGAGTGGTAGGCTATCGAGATCCCCTATAA
GAGGAGTCGGAACCCCAATCCAGGGAGKGGGAGGTGAGCAAGCAGGCCCC
CCGCCGCCGAAAGCTAGCGACTGCAGTGCAGCTGTGGAGGTGGAGACTCTG
GCCACTACAGTCTCCACGACAGCCACAAGCCTGAAGACAATGGATTTCATCA
GCGTGCCGGCCCAAAGGGCGGGAGCGAAGTCCCCATCAGGATCGCCGCATC
GCTCGCCAGAACTGACCACGACGCTCCAGAATGAGGACCTGCAGGGCATCC
TGGACATGATGAAAGCCAAGATCACCGCCATTCTATCCTCGTTCGAGACTAG
GCGTCACGTAACCAGCGAGGATAGGGGCGTGCTAGTGGACCTATCGGCGCTC
AATAACAGAGCGATTGAGTTACAGGAGGGTATCAACAAGAAGCCCCCACCA
AGGAGTACTGCCACACAGACTGAGGCAGAAAAAACRAAGCGTAGCCAGGT
GGCGCCACCCAGACAGGCATTACCGAATGTGCAGGTCCGCAAAACGGACAA
CCATCGATCTGCCGCGAAATCCACCGGTAAGGCGTTACCCACGACGGCTGAC
TCCAAACCGGAAAGTTATGCGTCTGTCGCCAAGGACGCTAACAAGGACGAG

GAATGGGCTAAGGTGAAGCCCAAGCGCCTGCGCAAAAAACCTGAGGCGCTA
ATCCTGAAAAAAACGGGTGAGGTTACGTACTCGGATATGCTTCGGAAGATGA
AAGCAGAACCGAGTCTGACCGAATTCGGCAAGCACGTGCGTAAAATAAGGA
GGACGCAACAGGGAGAACTACTTCTTGAATTAGAAGGTAAAGCCTCGGAGG
TCATCCCCAGCTTTAAAAATGAGCTAGAAGCGACGCTCAAAGAGATTGCTTC
GGTTCGCACGGGCGCGCATAGGACTGCGCTAATCTGCAGCGGACTAGACGAG
ACAACGACGGCTCAGGACCTTCATAATTCCCTGGTCTCCCAATTTCAGGGCAT
CCGCCTGGAACCAGAGGATGTAAGAGGCCTTCGCAGGATGCGTGACGGGAC
CCAGATAGCCTCTGTGCTAATGTGCGCGAACGATGCCATTGCGGTCATCAACC
GGGGCGTTGTAACTGTGGGATGGTCGCGTTGCCGCATAGCCCAAGACGTCCG
CCCAATAAGATGCTTCAGATGCCTCGAATTCGGCCACCGAGCTCCCTACTGCA
AGTCAGTCGATCGCTCTGACTGCTGCCTACGGTGCGGCGAGCATGGGCATAA
GGCAAAGGGCTGCGTAGCCCCACCAAGATGCCTGATCTGCAGCAGTGACGT
GGACAAGAACCACGCGACGGGTGGTTTTGCATGCCCCACCTACAAAGCCAA
CACCAAAGGAGCTAATAGCCGTCAAAATGATGCCAGAAGAAATTAACATCAT
CCAGCTCAACGTTAACCATTGCGCAGCAGCACAGAACCTCCTGACTCAAACA
GCGAAGGAGCGCCATGCGGACGTAGTGCTTTTGAGTGAACCATACCTACCTG
GTGTGGGTAACTCAGGAGTGCTACTCGATGAGACAGGCAAGGCGGCCATTAA
ATGTACATCCAGGCTATTGGTAGAGGAGTGGGATACCGTACCAATGCGCGGC
ATAGCATACGCCAAAATTAGAGGAATCCACTTCTACAGCTGCTATGCTCCACC
TAGCGACAGTCCCGARCAATTCGAGGACATGCTCGAAAAGCTGGTTAACCAT
GCAAGTGGGCGCAGACCAACAGTCATCGGAGGTGACCTCAATGCCTGGGCT
ACAGAATGGGGCAGTCGAATCTCTAACACAAGAGGACGAGCAGTGATCGAT
GCCATGAACCTGCTAGATCTCGTATTGCTAAACGACGGGTTCAAACCGACGT
TCAATAATGACAGAGGTACATCTTTCATTGATGTCACCTTTGTTAGCAGAGTT
CTAGTGGCTAGCTCGAACTGGATGGTCCATGAGGACATAACGCTGAGCGACC
ACAACCTAATCACGTTCGGTGCCCGAACAATGAGGCCGTCACCCAAACAGC
GAAGATGTGCGCTAGGTCCGGTATGGGACATTAGGAAACTGGATGAAGACAT
GCTGGCATACCAGATCGAGGGCATGGAGTCCATAATTGGACACGCGGAGACC
ATGGTGACAGCGCTCATGGATAGGCTTAGAGCAATGTGTGATGCGGTGATGC
CGAGAAAAAGGAACACGAAACGAAAGCCCCCTGTCTACTGGTGGAGTGACT
CATTGCACCAGCTTCGGACGGAATGCATCAAGGCGAGGAGGCAAGCGCAGC
GATCTAGAGGGCAACCGCACCACTCCCAGTGCATTGAGGTATATAAAGCGAA
GCGAACTGAGCTTAAGAACGGCATATCAGCAGCAAAGGCGAATGCATTTAAG
GACCTGATTGATAGCGTAGACGACGACCCCTGGGGTCTCGCCTACAAAGTCG
TAAGGAAAAAACTCAATTCCGCTGGTGCAGGATCTCCTCAGGACCCAGCCGC
CCTGGCCAACATCGTGTCGGAACTATTTCCAAGCCAACACACGTTATGGCAA
CCCGCTGTTGACCCCCCCGCCTCTGACTTCCCGTGCATCACGTCATGCGAAG
TCGTCGAAGCAGCAAAGAGGATCAGACCGAACAAAGCTCCCGGCATTGATG
GTATCCCGGGCGTGATCGTCAAAGCAGCCGCCACCGCAAGACCGGAGGTCTT
CAGGGATACATTCCAACAGTGTCTGCTGGACGGAGTCTTCCCCAAGCGCTGG
AAAAAGATGAAGCTGGTCCTTCTGCCGAAAGGCAAGGGGCCCGCAAATGTT
CCACGCAGTTACCGACCGTTGTGTCTGTTGGACATTGTTGGCAAGCTCTTTG

AGCGCATACTGTACGCGCGCATTGAGCTAATCACTGAAAGTCCTACAGGCCT
TCAGGGCCAACAGTATGGCTTCCGAAAGGGTAAAAGTACGCTCGACGCTCTC
AAATCCGTAACAGACGCTGCCAGGAAAGCACTCGACGGTAACCGATGGTTA
GGCGGCAGTAAGAAGTACTGTGCCATCATCACGCTAGACGTCAAGAACGCAT
TCAATACAGCGAGATGGCCCATTATCCTCGGGGCTATGCGCAACTTGGGCGTT
CCCGACTACATACGAGGCGTAATTGGCAACTACTTCAGGGACCGTGTGTTATG
GTACGTAACAGAAGATGGTCCAAGAAGCCACCAAGTCTCTGCAGGCGTTCC
CCAAGGGTCGGTACTAGGACCGATCTTGTGGAACATTATGTATGATGGAATAC
TGAGCATTAGCAAGCCCAGAGGTGTGGAGCTGCACTGCTTCGCCGACGACG
TTGCGATAACAGCGGTCGCCAAGACAATACCGGAGCTCCAGGACATAAGTAA
CGTGGCAATCACGGCGGCCATAGAATGGCTCGAGAAAGTCGGACTTAAAATA
GCTGCGCATAAGACCGAAGTAGTCCTGCTGAGCAGCAGAAAGTCCGTTGAG
TGCATGCGTGTGGAAGTCAAAGGAGTTGAAATCGCCTCAGCAGAAACGTTG
AAATACCTTGGTGTTCTAATAGACCGAAGGCTCTCGTTCAAGGCTCACGCAA
GGTATGCCAGCAAAAAAGCGGCAATGACAGCAGCAGCCTTGGCGAGAATCA
TGCCCAACGTGGGAGGACCCAGACTGCCGGCTAGGAGACTGTTAGTGGCGG
TTTCAAAGGCAACGCTGCTTTATGCTGCGCCTATCTGGAGCTGCGTTTCCACA
AAGAAAACCTATCTAGATAGTGCCCGCGCAGTATCACGGACAATGGCTCTCA
GGCTAATCAGAGGCTTTAGAACCATATCGGACGACGCAGCGCACGCTCTGTC
AGGCATTACACCCATTGACCTGGACATAAAGGGCAAATACCTTGCGAGTGAG
GGATACACTCAATTAGAGATCAAAGAGTGGATTCGAGGAGTATGGCAGACCA
GGTGGCAAGAGTCACAACGGGGACGCTGGACTTACAAACTCATTCCGCAAC
TAACGGAGTGGGCTGATTGCGAGCACAAAACGGTGGACTACCACATGACCC
AGTTCCTCACGGACCATGGCTGTTTTCGAGGGTACCTATGTAGGTTCCGCCAC
GTGGATACAGCCCAGTGCCTTTATTGCACAGACGCAGTGGAAACCGCAGAGC
ACATCCTACTGCACTGCTCCAGGTTCGCCGAGGAGAGGGCGCAACTCGTGGC
GCTCGCTGGGTCACCTCTCAGCCCGAGAGGCTTGGTTGCTGCTATGATGGCG
GACAAAATCGTTTGGGATGGGGCTCACGTGATCATCGTCACCATGATGAAGC
GTGTCCGTAAGGACGAGATGGCCAATCGGAACTATAGATAAGGAGTACCCCC
GTATGTTGGCGGGGCAAGAACTCTACGACTGGTACGCTCAGTTGGTCGTAAA
AAGGCGCTGCTGTGCACCGCAAAAGAAGATGGTGTGCAACTTGGCACCACA
TCCTGCTCACCGATGAAATACCTTGACTGGCAGTCCCGGTGAGCTTGACAAG
GACAGGAGAGAGAGCGGAGGTTTTTGTTTAGTACGTAGGCATAAGCCCTCAG
CTGAGGGTTATGAATCGTGCATGCCATCCAAGGACATTAGATGGTATCTTTAG
AAGATTTCATTTTCCTGCCGTATATAATAATAAAAAAAAAAAAAAAAAAAAA
A

>LTR5Gypsy#LTR/LTR5Gypsy

AGTTATTGCCCTGCAATTGATTCTCTAACATCTTGTGGTTCCACATAGTCTCCG
CTGCCATCAACGCCAACGAACGGTTAAGCGCGACATCGACACTTCTGCGCTG
CGCCGCGGCCGACGCCTGCTGCGCCACTGCCGACGACTTCACTTGATTGCTA
GGGACTTAGGGAAACATTTTGTACGCTAGATTTAGTTTCGAATGATAAATTGC

AATAAACGGTCGCTTGCGATCTTCAAAATCAAATCGATAACTGTAATTATTAACT

>HMR2HMR2#Unknown/HMR2HMR2

AAGCTTAGATATAGAGCCACCAACCGCTCTGAAATACTGGAAAGAAGCATTTCCCCTCACCAAGGATGCTTCTTAACGCTCATCTGCTCAGTTGATTCAAATTTAAATCCTAACATTAGCGCCATGACCACTGTGGAGAATTTACCAGCAGCCAGCACACTTAGCCGACCTGCTGCAAAGCGGGATTTATTTACGAGCACCCTATGAGTTTTTCGGAAGTGGTAGCTGGAGCAGGTCCAGTTTCTGTGGCACCCTCTAACCCGGCACCACTGACGAAAACTCCAGGCAAGCGGACAAATGAAGATCTGGATTGCTCAAATTTTAAGACGCCAATAAAAAATGATGCGCGACATCCAATTTTGTAACTCCCTGCATTTTTCCCCCGCTCATCACACCCGTTTTCAAAAGCAAGGCAGCTCAATCTGTTTATAAGGAATCAAAGCCAGAAATGGACCCCCCACCAGCCGTTACCTGTAGCAACAATGTCTCTGCTTGCAGCGAGGCGCACCCGGGATTGCCCCCCTACCCCCTCAAAATACAGATATAGCTGCCACCATGGAAAATCGTACCCAAGAGCCGTAGAGCACCCCCTATTCTAGCTAACGACGTGAAGGAAATTGTCCCGCTGCTGGAAAAGCTGAACTACACAGCAGGTTTATCCAGCTACACCACCAGGGCAATAGATGGGAATGGGGTCAGGATCCAGGCCAAGGATATGACTGCCTTCAACAAAATTAAAGAAGTCCTGGTGGCAAACGGCTTTCCATTATTCACCACATCAGCCCAAGAATGAGAGAGGCTTCCGAGTCGTCATCAGACACTCATCATTCACACTATGCTCGGGGATAGTCGAGGAGCTGCTGAAACTCGGATCTCCAAGCGACGCTGCGTCAGAAATATGACGAATCAGCTACAGGTGGCCCCATGCGAATGTATAAAGTTGAGATCGTCATGGCCAAGGATGGTAGTCATGAAAAAATTATCTCACTCAAACAAATCGTGGGCAAAGGTGGATATTGAAAGGAAAAACAGGACACGGAGCCAAAAACTCTTGCATGAAGCCACCAAGATGCATGAAATGCGCTGGCGATCACCTATCATCCTGTTGCACCAAGCCAAGATCCACCCCTGCCACATGCATCAACTGCTCTGGAAATGAGCATTAGTGCTTACAAAGGATGCCTCGTTTATAGGGCCGAAAAACAAAAGCTT

>HMR1HMR1#Unknown/HMR1HMR1

TCACGTCATCCCTAGAAGAACATTGAAGCGCTTGACTTCGGAATGCCGGAGCGAGGAGGAGGACTAGCTGGCCGAGGATCCGATATTCTTTGGTGAGGACTTGGGCGATTTGAGTGGCATCAAGGCCTTCTGGTGCAACCGAAAGCATAGGGACTGCAAGTGCAAGCGCTTTGTTTAGAAAACAACCAGGACTATATTCGGAGAATTACTTACCGTGGGTCGTCCAGGGGCGGTCTCAAGTTCGGCAGCTGCGTTAAGAAGATGTCCGGGAAGCTGGGCCCGAACATGGCTGTGTATCAGTTTTAGGATGGAAGTAGTTTTTACAGCGTGGGCAGTAGATCTTCACGGTTGACTTGCCCCACACATCGCTGAGGCCCACTGGCAGGGTGTTCTGCCTATTACAGGAGATATTSGGACACGATTCAAAGTCTCCTCTCATATACTTTCGGTGCATATCATTCACGCCACGCTCCGACCTGATGTATCGGGCGTGAATCATGCCGTACCACTTTTTCTCATCGYCGTAGAACAAGCCCGAGGAACTGTCGAACACCGGCTTCAGGATCACGTCCAGTGCTGGCTGAAGTACTCCARGCCCATCTGGTTGAACGTGTCCTGAAAGTAGTCGGTGGGCACGCGGCAGAGGAACTCGTTGCCCTTGATTCCGAGGAACCAATY

GATCCAGCTGCTGTTGTTGTTGTTGTTCTGGCTACAAGATTTACTGTGACTT
GCTATAATTTCTATAGGAAAAACCTAGTTACTTACGAGCTCGACATGTTGCCA
GTTCACTTGTTCACAGAAATATGACTTGTAGACTTCAAAGTCATCGTCTTCTT
CTTTGAGTTTTTGACACGTGCTAAAAGGCCTGCCAGGACCAAAGACACTARA
ATAAYAAGATGCATAACGCCATACGATTTTTTCGYCGTGGCTCNAGNGGTGGC
TCCAGGCTCTCTCGAATTTTTGT

>CR1ACR1#Non-LTR/CR1ACR1

TTGGCTTTCAAAGCGAACGGTCGTTTTTTTTTGCGCCCCGATTTTTATTTTGTG
TTTGCCAAACCAGCCGTGGTTTTTAATAATCTTCTTTAATTATCATATATTTTAA
ACATAATTATTAAAGATCCGTTCGCTTCGCGAGTGATTCTTTGTGATTTGTGCA
GTAGTTTAAACAAATATACAAAGTATTTTGCATTTTTCGTCTTTGTGTTTTGTT
TACTCTCCCTTTTGTGCGTTGTTCGCAGTGCTGTTTGGTGTTGTTTTTTGCATG
CACATAAACTGCACTTTTCGCTCTCACTCTCTCGCTCTTTCGCTCTCCCACAC
AAAATCTAAATTTCTCAGCGTGCAGACTGCTCTCGTGCGGTTCTTTTAACAGC
TCGCTTGAAAGTTTTGATCTTGTGTTTTCGTGCACAGTGGTCTGATTTTAGAT
AAACATGGAAACCGTAAATGTTGTCTGCTTTTATAAAAATTGTTGCCAGGTTA
TAAAACCTGAGCAGCCAAAAATGACTTGTTGGCTATGTGATAGAATGGTGCA
TACTAAGTGCGCTGGTTTTAATGGCCGTACAAGTGATGACCTAGCAAAGGGT
AAAAATCTAAATTACTGTTGTGATGCTTGCCTTGTGGTTGCGAACGAGATGAA
ATCGTTTATGCGCCAAACTAAAGGTGGCTTGAAAGAACTGATCAACAGTTTT
GGCGTAGCTAGAGATAGTTTTCGTCGAGCCGATGATCTTCTTTCCGCGCTTAA
TTCACAGTTTAATGGCCTTAAGCTATTAGATGAATCTCCAAAGCGCAAAAAAG
CCGCTGGTGGTAGGCTGCCTAAGGCCCCGCAACCACGGGCAAATGATCAAC
AGGACTCCACAACTGATCAGCTGTCAATCGCAGCAAATCCGCATATGTTGCTA
AGATCGGCAGCTAAAAAAGATTCGGAGCAATCCGATCAATCCGCTGTGGAGG
GAGCCCACCCTGTGATTGCTAAAGATTCCGAATTGTCTGCACTGGTTTCTCAA
CCAGTGATTCCACCTGTCTCGATTAGTTTACCACCACAAGGGAACATTGAGTC
CGGACTACCGGCACAAGTTGGCACTTCAGAAATACAATCTGCAGGGCCAAA
ACCCCTCGCGGTGGTTCCACTAAAGAAACAAATTTTCGTTTCTCGGCTTTCCC
CTGATCAAACATCGTCTGATGTATTGTCTTATATACAAGACAAAACAAAAGCC
GATAATATAAAAGTGGAAAAATTTAACTTCTCTTACGCTAGGGACATATCATCT
TTCAAGATAACTGTCCCAAACGAGCTTTTTTCGACCATATGTTCGGGTGATTT
TTGGCCGGATAGTATGGTGGTAAAAGAGTTCGAAGCTAAGATCAAAAATAGG
AAAAAGGGGCCCGTGGGAATTAAACTTCCCTCACGTGGCCAGACCACTGCA
CCATCCGCCTCTTCATCTTCTTCCTCTTCAAAAAACTAACAACGAATCTTACT
ATCTCCTATCAAAATGTTAGAGGCTTGCGTAGCAAATTAACCAAATTGTATTGT
GATAGTCTTTCATTTGCATCCCATATAATAGTGTTCACAGAGACTTGGTTAAAG
CCGGAGATACTTAACTCCGAAGTCTTCCCAGGTATGTACACTATATATAGGTAT
GACCGTCCCTCCAGACGGGGAGGTGGAGTCTTAATTGCGGTTAGATCTACCC
TCGCGTCGGAGGAGTTACTTTTGGACGAATCCCGTAACTCGGAATTCTTATGC
GTAAAGCTGTCTTTTTCCGACAGATCAGTTTATATTACGTGCTCGTATATTCCG
CCATCTTCTGAATTCCCAGAATATCAGAATCATCTGTCCGCTATCCAGTCCATC

TTGAATAAACTGTCTGACAGGGACCAATTGGTAGTTCTAGGTGATTTTAATAT
ACCTGGCACAATGTGGTCCCCAGAAAAACAATCGAATATCCTTCTCCCTTTAG
CACAACATGACTTTATTGACGGCTTGCTCGACTTATCGTTGTCGCAAGTTAAT
TATATTCCAAACTCTCTTGGTCGACTGTTGGATCTATGTTTTGTAACAAGTCCT
GAAAGTGTGTTTCTGTCCAGGGTAGCACCTCTTACTCAACCTGAAGATCCATA
TCATCCTACTTTCGAGGTGGCAATCGACATAGGTACGGTATTAAAAGTAAATT
CAGAGAAGTCAACAAAGCGAATTCCCTGTTTTCGCAAGGCAAATTTTCGGAG
GCTAAACAATTTTATAGCTGGCTTTAATTGGTCCGATCTTTACTCCTGCAACAT
AATGGCGGATGCGATTAATATGTTTTATACCGCAATTAAATCATTTTTTGACTCT
TGTGTTCCGATGTACTATCCGTCAATCTCTAAACCTCCTTGGTTTAATAAAGAG
TTGACACACCTAAGAAATGTAAAGTCCAGACTCTATATAAAATTTAAAAATAC
CGGTTCTCAGTCCATCCTTTGTAAATATTTAAGCGCTCGGTTAAACTTTACCGT
GCTTAATGCTCAGTGCTACAAGAATTATTTAAACCGTTGTAAGTTCCAGTTTG
CACAGGATCCGAAACAGTTTTATAATTTCGTTAGCACTAAGCGAAAAACAAG
TTCTTACCCTTCCTCGCTATTTTTTGAAAACACTACGGTAACATCGGATCAGG
CAATAGCCGATCTATTCGCCAAGTTTTTTCAAACAACATATTCAACTTTACCTC
ATTCCGAACAGCCATACTCTTATGCGGTATCTAAGTCGAATCTAATATTTTGCC
CCACTATAAACGAAAGCTCACTGCTTAACGATCTTCAGCGTGTTAAACCGGT
CTATTCGCCAGGTCCCGATGGAATCCCTGGCTGTGTGCTCAGATTCTGTGCGG
AAGCCTTGTGCAAGCCTCTACTGAAACTGTTTACCTTATCTCTGGAATCTTCA
CAATTCCCTCATATATGGAAGGAGTCCTTTGTGATTCCTCTTCATAAAAAAGG
TAGCAAACTGGATGCAAGCAATTACAGAGGAATCTCTAAATTGTCGGCTATCC
CAAAACTTTTTGAAAATGTTATCACTCCTCATTTGCAGCACCTTTGTAGATCA
ATCATATCACCGTGTCAACACGGTTTTATGAAACGCAGATCAACAACCACTAA
CCTCTTGGAGCTAACTTCTTTCGTAATACAGGGTTTCAAAAATAATCTTCAAA
CAGATGTCATCTACACTGATTTTAGTAAAGCATTTGACTCTGTTAATCATTACC
TTCTAATAAGAAAACTTGATCTTTTAGGTTTCCCTGTTGATCTTCTAAATTGGA
TTTCAAGCTATCTGAATGGCAGGACACAACAAGTCCTCTTTAAAAATTCTTTA
TCTTGTATTCTCCGAGTCACATCCGGTGTCCCACAAGGGAGCCATCTTGGTCC
GCTTCTTTTTACCTTATTCATTAACGACCTCCCCTTAATAATAAAACATTCGCG
TGTACTTATGTACGCAGACGATGTTAAATTATGCCTCCAGCACAAGGACACTT
CGTGCCATTTGGACTTACAATCCGATCTAGACCGATTTCAAATATGGTGCCGT
GATAACGTATTAGACTTAAATGGCTCCAAGTGTAAAGTTATGACCTTTTGTCG
TGCCAACCCAATACGCACGACTTACACTCTAAGTGGGTGCTCTTTGGACAGA
ATAACACGAGTTGATGATCTTGGTGTTCTTCTGGACCCTAAACTAAAATTTTC
TGACCATATTTCGTCTATTGTCAATAAGGCCAGGGGTGTGCTTGGTTTTATAAA
AAGGTGGTCTAAGGAATTTGATGATCCTTACTTGACTAAAACCTTATTTATTTC
GTTAGTCCGTCCGATTCTCGAGTACGGATCACCTGTTTGGAGTCCACAATACG
CAGTCCACTCGGACCGCATTGAATCGGTCCAAAAAAACTTCTTACTTTTTGCC
TTGCGGCGCCTAAATTGGGATGCAAACCGTATATTACCTCCTTATTCCAGTAG
ACTACTTTTAATTAATTTACCGTCCCTAGCTAACCGTAGAACTATGCTTGGAAC
AGTCTTTATTTGTAAGCTTATTCGTGGGGATGTTGAGAGTCCCGACTTGATTA
GTCGGCTTAACTTCTCGGTTCCAAGTAGATTCACTAGAAACTATATACCCCTT

ATCTTAAATCATTGTAGATCTAACTATGAGTTGCATGACCCTTACAGAGTTTTA
TGTTCTGACTATAATAGACTTTATCCTATTATCTGTAATCTTGACTCTCTGCCGC
TCTTAAAGCAATCGATTTTAACTTTTTATTACATAATTAGATCCTACTAACACT
AATATTTAATATAGTTATTTTACATTATATTCATTTCCTCGTTCCTGTTCTCTTTT
TTATATCGCGTCTATATCTTCTCGCGAATCGAGCCGTACGATACACGGCAGCGC
CCCTCGGTCGGTTGGGCGGGAGGTGTGGCCGTGGGACCCGTGCGAAAAAAA
A

>412B_LTRGypsy#LTR/412B_LTRGypsy

TGTAGTATGTGCCTATGCAATATTAAGAACAATTAAATAAAATAGCATATTAAC
TTATGGCAGCACTTTGTTGCTATGTTTATGTTTATGTTTATGCACGCAGTTAGG
CCAGGGCGGATGTAACATGATCGCCCACTCGAAGGCAAAAAGTATAAGTGCA
TGGTCAGCATTCACACGCCGACCAAATACATATTACATACGTACATACATATCT
CGCTCTCCCGATAAGCCTAGATATATAAGATATACATAAGAACGCCGCTCCGCT
GCTGGCGTACCCGGCAGCGCAGCTACGCGGATTAGCCTAAGTCCAAATATATT
AAAAAACTGTAAAATCAGAGAGACTCTGTAGACGTTGAGCTGACAGAACCA
TTTCTGCCTACTCTAAAATCAAAAGAAGAAATTGAATAAATATATGTCAGCCC
GACGGCTGCCTTAAACTTAAAACGGACTTGTGTTCTTAATTGGAGTTCATCAT
TACACAAGTGTAAATGTTAACGAATCTAGAGCATAGCATAGCTTAGCATAAAC
TATGACGACTAGTTATCGTGACAAGCAGTTCATCATTACA

>412_LTRGypsy#LTR/412_LTRGypsy

TGTAGTATGTGCCTATGCAATATTAAGAACAATTAAATAAAATAGCATATTAAC
TTATGGCAGCACTTTGTTGCTATGTTTATGTTTATGTTTATGCACGCAGTTAGG
CCAGGGCGGATGTAACATGATCACCCACTCGAAGGCAAAAAGTATAAGTGCA
TGGTCAGCATTCACACGCCGACCAAATACATATTACATACGTACATACATATCT
CGCTCTCCCGATAAGCCTAGATATATAAGATATACATAAGAACGCCGCTCCGCT
GCTGGCGTACCCGGCAGCGCAGCTACGCGGATTAGCCTAAGTCCAAATATATT
AAAAACTGTAAAATCGGAGAGACTCTGTAGACGTTGAGCGGACAGAACCAT
TTCTGCCTACTCTAAAATCAAAAGAAGAAATTGAATAAATATATGTCAGCCCG
ACGGCTGCCTTCAACTTAAAACGGACTTGTGTTCTGAATTGGAGTTCATCATT
ACA

>412_IGypsy#LTR/412_IGypsy

TGGCGACCGTGACAGNNTCCAACGCTGGACGAATTGACCAAAGCTGGTGAA
AACAAAGGAACAAAGGAACACTGGACTGGAAGAAGACTGGACTAATTAAAT
GGAACTGCAAAAACCAAGGAAAAATCTGAGTGAGTAGAGTTCTATTGAGTAT
GGGCAAACACCGTGGCGGTTTGAAAACTAAGCTGAATAAACGTATAGCCCAC
GTAAGGTGGCTAATATACGGTCAGCAAACGCCACCGGTTTGGTCGAAAGCTC
TAAAGCTACATGCAGAGCTAGACCACTTGTTGCAATATCAGCAAGAATTAAA
GACCCATAAGCTCGAGAAAACTCACTCAGATAATATTAAAAATATACCCACAA
TTAATGAAGTTCCAAAATACCAGGCATGTCCAGCACCAGCACCAGCATTAAC
AAAACCAAAGAAGTCCTGCCCCCCTGGCTGCGAAGGAATCTGGAGTCCCCA

CTGCCTGGGGACTTGTGAGCGACCATCGACGTCTTCAGCGGCGAAGAAATAG
ACAGCAGCGAGGGAGTGTCAGCGTGCCACCCCGGCGACGCCCAGCTGACAC
CTGATAGAGCATCATCAACAGCAGAATATAATAATAAATATATATAAATATAAA
GTAAATATAAAATATATATAGATAAGAAAAATTGTAAGAAATATTGTAAAACGG
AGCATATACTATTATGCCCTGTTAACCCAATATGGCCCGTGAAGCCATAGCTAG
AATCAGGCAGGCAACAATGTAAAATACAATTTTTTTTTACTCTTGCGAACAT
TGAAAGATTTTATAAATAGATAATTCCAAACATAAATGTCTATAGAGACAAATG
AAATAAGTAAAACTGAAAATAAAAGTATATACAAAGGAAATTTTCTATTCTAT
TCTCCAAAATATAAAATTAGTATACCCAAAATGGGTCTAATAGACACTAAAAC
TGTGGACTCTACAGCCAATGTAATAAATAAAGTAGAAGTCCAAAATGCAGAC
TTGTTCTGGATAACCATAATACTAATTGTAATTGCATTAATTATGGTATCCAATG
CATTAATAAAAATATACAAACTGCATAACAAGTGTCTTAAGAAACGATACCGT
AGCACTGCTAACGGTATAGATAATATTTAAGGAAGATCTTTAATAAAGTCAATT
ATGAATGAAAATATGAGAAAAATTATATGAAAAAAAAAAAATAATAAATAAAA
AAAAAATATAAAACGTAATATTGAATTTATCTACATTAAAAAAAAAATATATAC
AAATGAATAAATTTGAAGTTATGAGTATACCACAGCATGGACTGGGAAAAGC
TTGTTGATCAGATAAAAGATCAAAATGAAAATTTCAGAAAATCCTATAAGTGC
TTAACGCAAAACAGATCAACACAAGCTGTAACAATCAATAGGAATGCCCAAG
TCTTGGTAAATAGTTATAATGAAATCAGAGAGTTGATCCAACAAAATAGAAAG
AATTTGGAACGCAAACAGTGTGCTAAGGCTTTGAACCTACTGGTGACATTAA
GAGAAAAATTAATATTTATAAAAAATAAATTCAGTCTCCAGATAGAAATTCCA
ACCATAGTAAACACCCCACTAAGAATAAATTTGAATGAAGACAGCACTAACT
CTGACGAGGAAGATAGGACTATAGTCAAGGAAGACATTAAAGAGGAAGATC
TTCACGATCTAACTATACCAGCAAAATTAATGCTGAAGAACGACGATAAAAC
AAATAACGCAGCCGACTCCGAAAATAACTTAACCATGGCAGAAGAAGCAGC
TGCCATTAGGTCTTACATTAGGGAAGTCGCCTGCACAGTGCCAGAATTTGATG
GGCAAAAGATCCATTTACAAAGATTCATTAAGGCAATCAAATTGGTAGACCTA
GCTAAGGGACCATTTGAAGACATTGCAGTTGAGGTCATTAAGTCAAAAATAG
TTGGCACAATTTTGAACTCAGTTGACAATGAAACGACAATTCCAGCAATTATA
AACAAATTGCAGAAAGTAGTTGTCGGTGAGACATCCAGTAATGTCAAAGCAA
AGCTAGCAACAGTTCAGCAGAGAGGTAAAACTGCAACGCAATTTACCGCTG
AAGTTGATAGCCTGAGAAAACTTTTAGAAGCTTCCTATATCGATGAGGGTATA
CCTCTAGAACATGCCACTGGTCTAAGCACCAAAGAGGCAATTGAAACCATGA
TACATCGTGCTGAGCACGAAAGTATCAAAACAGTACTGGAAGCAGGGACTTG
CACCACTATGGATGCAGCGATAAGCGCATACATAAGAACGAGTACAAGAGTT
ACCGGTGACATCAATAAAGTGATGTACTTTAGAGGTAACAGACCCAATAGAG
GATACGGAAATGCCAATAGAGGTAGTAACCGCCGTAGAGGCTTTAATAACAA
TAGTATTAGAGGCAACTACCATAACGGTTACCAAAATAACGGTTACCAAAATA
ACGGTTACCAGAATAACGGTTATCAAAACCGCTATAATGGAAATAATAACCGT
TATAATGGCTATAACAGAGGCCGTTATAATGGAAACAGAGGCCGTAACAACA
GTCAGAACAACTACAACAGAAACAATGCCAATGTACGAGTAATCCAAGAAC
AGGGCAACTCGCAACAGCCTTTAGGTACTCAGTAGAAGAAGATCGTAGAGTA
TACACCATCAATTATAATCTCAACATATTTCTACATTCATTCATGCCAAAACA

GGCGTAAAACTAGTTTTTCTACTTGATACAGGTGCAGATATCTCTATTCTCAAA
GAGAACTCTGACAAATTTTCTAATATTCAAATAACCAATAAAATAAACATTCA
AGGCATAGGCCAACAGAAAATTCAGTCTCGAGGACAGACTTTTATTGAGATA
CAGACAGGTAAATACGTTATCCCACACGATTTTCATTTAGTAGATAAAAACTT
TCCAATACCGTGTGATGGAATAATCGGAATAGATTTCATAAAAAAATATAATTG
CCAAATCGATTTAAACCAAGAAGAAGATTGGTTTATAATTAGACCAAACAATT
TGAAATTTCCAATATATATTCCCATAGCATACAGCTCTGGTATTAACACAACGT
TATTACCAGCAAGATCCCAAGTTGTCCGAAGATTAATAGTATCATCAAAAGAT
GATAACATTTTAATTCCAAACCAGGAAATTCAAACTGGTATTTATGTTGCAAA
TACAATCGCAACATCAAGTAATACATTTGTCCGAATTTTAAATACAACCGATTC
CGACCAATTAGTCAATATGGACACTCTAAAATATGAGCCACTTTCGAACTACA
ATGTAGTTCAGGCAAATAGTGAACACAGAAATAAAACTGTCTTATCTCAATTA
AAGAAAAATTTCCCCGAATTGTTTAAATCACAATTAGAAAATATATGCAGCGA
ATATATAGATATATTTGCATTAGAATCAGAACCTATAACAGTTAATAATTTGTAT
AAACAACAGTTGAGATTAAAAGATGATGAGCCAGTATACACGAAAAATTATA
GAAGTCCTCATAGTCAAGTGGAAGAAATACAAGCCCAAGTTCAGAAATTAAT
AAAAGATAAAATAGTTGAACCATCAGTTTCACAGTACAATAGCCCTTTGCTAT
TAGTACCCAAAAAGTCAAGCCCGAATTCTGATAAAAAGAAATGGAGATTAGT
AATAGACTATCGCCAAATTAATAAGAAACTTTTAGCTGACAAATTTCCACTAC
CGAGAATAGATGATATTTTGGACCAACTTGGTCGAGCAAAATATTTCTCCTGC
CTTGATTTAATGTCAGGTTTTCATCAAATCGAACTGGATGAAGGCTCGAGAGA
TATAACATCTTTCTCAACCAGCAATGGCTCATATCGTTTCACGCGATTGCCATT
TGGCTTAAAAATAGCGCCTAATTCATTCCAAAGAATGATGACTATAGCATTCTC
CGGAATAGAACCGTCTCAAGCATTCCTTTATATGGATGACTTAATAGTCATAGG
TTGTTCCGAAAAACATATGCTTAAAAACCTCACTGAAGTTTTTGGTAAATGCA
GGGAATACAACCTAAAGTTACATCCTGAAAAATGTTCATTTTTCATGCATGAA
GTCACATTTTTGGGACACAAATGCACAGACAAAGGAATTTTGCCGGATGACA
AAAAATATGATGTCATTCAGAACTACCCAGTTCCACATGATGCGGACAGCGCT
AGACGTTTTGTAGCATTTTGCAATTACTACAGACGTTTTATCAAAAATTTCGC
CGACTATTCGCGGCACATAACAAGATTATGTAAAAAGAATGTTCCATTCGAGT
GGACAGATGAATGTCAAAAAGCATTCATACATTTAAAATCTCAGCTAATTAAC
CCAACACTCTTGCAGTACCCAGACTTCAGCAAAGAATTTTGCATAACAACAG
ATGCAAGCAAGCAAGCGTGTGGCGCAGTTTTAACTCAAAACCATAATGGCCA
CCAACTCCCAGTTGCTTATGCATCCAGAGCTTTTACGAAAGGTGAAAGCAAT
AAGAGTACAACAGAACAAGAGTTAGCAGCAATTCATTGGGCAATAATACATT
TCAGACCATACATTTACGGAAAACATTTCACTGTGAAAACAGACCATAGACC
ATTGACATATTTATTCTCGATGGTGAACCCCAGCTCTAAATTAACTAGAATAAG
GCTTGAACTAGAGGAATATAATTTTACAGTAGAGTATCTAAAGGGCAAGGAC
AATCATGTAGCAGATGCGTTATCAAGAATAACCATCAAAGAGCTAAAAGATAT
AACTGGAAATATATTAAAAGTCACTACAAGATTTCAAAGTAGACAAAAATCCT
GCGCAGGAAAAGAACAATTGGATTTGCAAAAGCAAACCAAAGAAATAGCTT
CAGAGCCCAACGTATACGAAGTCATAACAAATGACGAGGTACGAAAAGTAGT
GACATTGCAATTGAATGACTCGATATGTTTATTTAAACATGGAAAGAAAATTAT

TGCAAGATATGATGTTGGTGATCTTTATACTAATGGAATTCTTGATTTAGATCA
ATTTCTCCAAAGGCTTGAATTGCAGGCCGGTATATATGATATCAGCCAAATCA
AAATGGCACCGTGGAAAAAAATCTTTGAACACGTTTCAATAGATAAATTTAA
AAATATGGGCAATAAAATATTAAAGAATTTAAAAGTAGCGCTACTTAACCCGG
TGACCCAAATAAATAATGAAAAAGAAAAAGAAGCTATATTGTCTACATTACAT
GATGATCCAATACAAGGAGGGCATACAGGCATTACAAAAACCTTGGCCAAGG
TCAAAAGACATTATTACTGGAAAAATATGAGTAAATACATAAAAGAGTACGTA
AGAAAATGTCAAAAATGCCAAAAAGCAAAAACAACAAAGCACACAAAGAC
TCCAATGACGATAACTGAAACACCAGAACATGCTTTCGATAGAGTTGTTGTG
GACACAATTGGTCCACTACCCAAGTCAGAAAATGGTAACGAGTACGCAGTCA
CTCTCATATGTGATTTAACCAAGTACTTAGTTGCCATACCAATAGCAAATAAAA
GCGCAAAAACAGTCGCAAAAGCTATATTTGAATCTTTTATTCTAAAGTACGGT
CCAATGAAGACGTTCATAACGGACATGGGAACAGAGTATAAGAATTCAATAA
TTACTGACCTGTGTAAATATTTGAAAATAAAAAATATAACATCAACAGCTCAT
CACCACCAGACAGTTGGAGTAGTAGAAAGAAGTCATAGAACTTTAAACGAG
TATATACGATCCTACATATCGACGGACAAAACCGATTGGGACGTATGGCTTCA
ATATTTCGTATACTGCTTCAACACGACCCAATCTATGGTACATAATTATTGTCC
ATATGAATTAGTTTTCGGTAGAACAAGTAATTTACCAAAACATTTTAATAAACT
ACATAGCATAGAACCAATATATAACATAGATGATTACGCTAAGGAGAGTAAAT
ATAGGTTAGAGGTAGCATATGCTCGAGCAAGAAAACTTCTTGAAGCACACAA
AGAAAAAAATAAAGAAAATTATGACTTAAAAGTAAAAGACATAGAATTAGAA
GTAGGAGATAAAGTTTTACTAAGAAATGAGGTAGGTCATAAATTAGACTTTAA
ATATACGGGGCCCTATAAGATAGAAAGCATAGGAGATAATAACAATATTACGCT
ACTTACTAATAAAAACAAAAAACAAATAGTTCATAAAGATAGATTAAAGAAA
TTTCATTCATGATTGAATTTAAACTTATATTTTCCTTAATCATTTATACAAATTTT
CCATACACTACGTATATTTTTATCTTTGCATTATAAAATCAACTATTGTTGTTCA
AACAAAAACACAAACAAAATAAAAATAAAAATAAAATAATTTGCATTTAATA
ATCAAAATAACTTCACTAGGTTACGTTATTTTTCAAAAGGAGGGAGA

>297_LTRGypsy#LTR/297_LTRGypsy

AGTGACGTATTTGGGTGGTCCAAACCAGCCACTTCCATTATTTCAAAGAAATC
AGTAATGCACTCTAGTAATTTTCCATAACTGTATCCCAGCTGCGCAGACTCGT
TTATCTTTTGCAGCGCAGCGTTCTTTGTAAACATCCTAAAGACCTGCCTAAGC
AGATTTGACTGCCCTCTTTCAACGCTACCTAATCTTAAGAACCCAAGAGCGA
GGCTCTCCCGAAATACAAATATTGTTCAAATACTGAGGCTTCTCCTCAATCCA
ATTTGCATTTGATTTTTAGTCTTAAGCTGAGATCCAAAGAATAAAGTCGTGAA
ACTATTTCTCCTAAAAACTATTTTTTATTTCTTGGCGTTGTCCTTAGTCAACTG
ACGGGACATTAGTTCGACTCATAAATAAAACAACAATTTTACT

>297_IGypsy#LTR/297_IGypsy

GGCGCAGTCGGTAGGATACAAAAGTATCCGAAAAAAAAGAACCTTCGAATG
GAAAATAAGTTAAATTTTATAGTCCTGTGCTCGAAACATCTCCCAAAATAAAT
TCGTGAAAACTCTTCAACTTCAATTATAATTCCAATTCGGTTATCCAATAATAA

GTGGAAGTGAAATACGAAACAAAAATATTAAGTCCAAAGGCAACTAAGTTTT
AAAACCAACATATAAAAATAAAAAATTAAAACAATATAGAATTTTAATAATAC
AACACAAAAATTTACAAAACAAAAAAACAAACAAGTGAAACTAGAAAGCTT
AAAAATAATAATAACATTGAATCCGAAACAAAACAAAAAAATAAAACACAAA
AGTTAAAAATTTTACAATAAAAATGTCACAACCAATTATTGCGCTGAGCGACA
TAAACCTTGCCGAAGCCCGTCGGCAGCTTAAAGACATTATGCCATTCAAGGG
TGATCCAGAAACCCTTCACACCTTTATCAGCAGAGTGGATTACGTAATTTCGC
TCTACCAAACAAATGATGTCCGACAACAGAGGATTCTACTGGGAGCCATCGA
AAGGAACTTGGACGGACAAATTACACGATCTTTGGGACTTCCGAACGTCGAA
GATTGGCCTACCCTTAAAGCAAGACTCATCGCGGAATTTAAAATTCAAACAC
CAAACTACAAACTTCTGGAGAACTTCAGGGAGACACCATACAGAGGAAGCC
TAAGAGCATTCTGCGAAGAAGCGGAGAGACGACGTCAATTACTAATTTCGAA
ACTACACCTGGAAGGTAACCAATCGGATTTTCTTATTTATATTCAGGGTATTAA
AGAATCTATTAAGATACTGATAAGGAAACTACCAATACAATTATTCACTATTTT
AGCCCATCACGATATTACAGACTTAAGATCCTTAATTACCATTGCACAAAATG
AGGGAATTTATGAAGAACACATTAATTTTGAATTTTATGAAAAACCAGAATAT
CGTAATAAAAATTCAAATTCTAACCAGAATTCGAAAACACAAAAATTCAATAC
AAATGTTCAAACTCAAAATCGACCAAGTTACTCACAATATTCCCAACCCTTCC
AACCTAATTTTAATCAATACATTCAACCATTTAGACCTAGCTATACACAGCAGA
TAACTAACAACCCACCCATGTGGCACGCACCTAATTATTTCAGACCCAACCA
ATACATAAACCCACAACCCATTATTCAAAAAAATCATTTCCAACAATATCCCA
ACAAAGCCCAATTTCCCCAAACAACGCATTTTAGAGGAAATACATACCCTCG
ACTACAACAACCCTCTACATATAAAAATACTAACTTCCCGATTACTAAACGAC
TAAGACCATCGGACAGTGAACAAACTAAAATGTCTATTGACGAAATTAGATT
CCAAGACGCGCATGAATTCGAACAAGTCCAACCTAATTATTACGAGCAACAG
TATTTTAACCAAAATCAATACAATCCGTATCAAAATCATAGCTTCATTAATGAA
GGGCAACAACAAGTTCAATTTGTACAAATTAATAACAAACAAAACCAAAATA
ATTCTGAACTAAACGAAAATTTTCGGTTAACAGTTCCGGAAAATACGAATAC
ATAAAAATAGTATACAAAGGGCGTTCATACAAATGCCTTCTAGACACAGGATC
AACAATTAATATGATCAATGAAAATATATTTTGTCTTCCCATTCAAAATAGTAG
ATGTGAAGTTTTAACATCAAATGGCCCTATTACCTTGAACGACTTGATTATGTT
ACCCAGAAATAGTATTTTCAAAAAAACCGAACCATTTTATGTGCACAGATTTT
CTAATAATTACGATATGCTAATTGGCAGAAAATTGTTGAAAAATGCTCAATCA
GTTATTAATTACAAAAATGATACAGTTACCCTTTTTGATCAAACATACAAATTA
ATTACTTCAGAATCCGAAAGAAACCAAAATTTGTATATCCAAAGGACACCAG
AATCAATTGCAAGCTCAGATCAGGAATCAATAAAAAAATTAGATTTTTCACAG
TTTCGATTAGATCACCTAAATCAGGAGGAAACTTTTAAGTTAAAAGGCTTGTT
AAATAAATTTAGAAATCTTGAATATAAGGAGGGAGAGAAATTAACATTTACAA
ATACAATTAAACACGTACTAAATACAACACATAACTCCCCAATTTATTCGAAA
CAATACCCACTTGCGCAAACACACGAAATCGAAGTAGAAAACCAAGTACAG
GAAATGCTGAATCAGGGATTAATTAGGGAAAGTAATTCTCCATACAATAGTCC
TACTTGGGTCGTACCAAAGAAACCGGATGCTTCTGGTGCAAATAAGTACAGG
GTAGTAATTGATTATAGAAAGCTAAATGAAATAACCATACCTGACAGATATCC

AATTCCAAATATGGACGAAATTCTTGGCAAACTGGGTAAATGCCAATATTTTA
CAACGATCGATCTGGCAAAGGGATTTCATCAAATAGAAATGGACGAAGAATC
AATTTCTAAAACTGCATTCTCCACAAAAAGCGGTCATTACGAATACCTTCGAA
TGCCATTTGGCCTTAGGAATGCACCCGCTACTTTTCAAAGGTGCATGAATAAT
ATCCTTCGACCGTTGCTTAACAAACACTGTTTGGTGTATCTGGATGATATTATA
ATTTTTTCAACATCCCTTACAGAACATTTAAATTCAATACAATTAGTTTTTACA
AAGCTTGCAGATGCAAATTTAAAATTGCAACTAGACAAATGTGAGTTCTTAA
AAAAGGAAGCTAACTTTCTTGGTCACATAGTTACCCCTGATGGTATTAAACCA
AATCCTATTAAAGTTAAAGCCATAGTTTCATACCCAATTCCGACAAAAGATAA
AGAGATAAGAGCTTTCCTTGGATTAACAGGTTATTATCGCAAATTTATTCCAA
ATTACGCAGACATAGCAAAACCCATGACCAGCTGCTTAAAAAAAAGGACAA
AGATAGATACACAAAAACTTGAGTACATAGAGGCATTCGAAAAACTTAAGGC
TTTGATAATTCGTGACCCAATTTTACAATTACCTGATTTTGAAAAGAAATTTGT
TTTAACCACAGATGCAAGTAACTTGGCCCTCGGGGCTGTCCTTTCTCAAAAC
GGTCATCCTATATCTTTTATTAGTAGAACACTTAACGATCACGAATTAAATTAC
AGTGCTATCGAAAAAGAATTACTTGCCATAGTTTGGGCCACAAAAACTTTTC
GACATTATTTACTAGGACGACAATTTCTCATTGCCAGTGACCATCAACCTCTT
AGATGGCTTCATAACTTAAAGGAACCAGGTGCTAAGTTAGAAAGATGGAGAG
TTAGATTAAGCGAATACCAATTTAAAATAGATTATATTAAAGGGAAAGAAAAT
TCAGTTGCCGATGCATTATCAAGAATTAAAATTGAAGAAAATCATCATAGTGA
AGCTACTCAACATAGTGCAGAAGAGGACAATAGCAACCTTATTCATTTAACA
GAAAAACCAATAAATTATTTCAAAAAACAAATAATCTTTATTAAATCCGATAA
AAATAAAGTAGAGCATTCAAAAATATTCGGTAACTCCATTACCACAATTCAAT
ATGACGTAATGACACTTGAAAAGGCCAAACAAATTTTACTCGATCACTTTATC
CATAGAAACATTACCATTTATATTGAGAGCGATGTAGATTTTGAAATCGTTCAA
AGAGCACACATAGAAATTGTTAATACCACCTACACAAAAGTAATTCGCAGTCT
TTTCCTATTAAAGAACGTTGGTTCATACGCCGAATTCAAAGAAATCATACTTC
AATCACATGAAAAACTTTTACACCCTGGTATACAGAAAATGACAAAATTATTT
AAAGAAAATCACTTCTTTCCAAATAGCCAACTATTAATTCAGAATATAATAAA
CGAATGCAACATATGCAATTTGGCCAAAACAGAACATAGAAACACCAAAATG
CCTTTAAAAATCACACCCAACCCGGAACATTGCCGAGAAAAATTTGTAGTAG
ATATTTATTCATCTGAGGGAAAACATTACATCAGTTGCATTGATATTTATTCTAA
ATTCGCTACACTTGAGCAAATTAAAACTAAGGATTGGATAGAATGCAGAAAC
GCATTAATGCGCATTTTTAATCAACTAGGAAAACCCAAATTATTAAAGGCAGA
CAGAGACGGAGCTTTCTCCAGTTTAGCTTTAAAGCGATGGCTTGAAGAAGAA
GAAGTCGAATTACAGCTCAATACAGCAAAAAACGGAGTAGCAGACGTCGAA
AGATTACACAAAACAATAAATGAAAAAATTCGTATAATCAATTCATCTGATGA
TGAAGAAGTAAAATTAAGCAAGATAGAAACAATCCTCTACACATACAACCAA
AAAATTAAACATGACACTACTGGACAGAGACCTGCTCAAATTTTCTTATACGC
TGGGCATCCCATATTAGACACTCAAAAAATTAAAGAGAAGAAAATAGAGAAA
ATAAATGAAGACAGACGGGAATTTAATATTGACACTAATTACAGAAAAGGTC
CACTACAGAAAGGCAAATTAGAAAACCCATTTAAACCAACCAAAAATGTAGA
ACAGACAGACCCTGACCATTACAAAATCACTAATAGAAATAGAGTTACGCAC

TACTACAAAACACAATTCAAAAAACAAAAGAAAAATAATAAACTCTCAATTT
CACAGGCACCTGGTACCCGATAACACTATTGTTTATACTGATCACAGCTGTTC
ATGGACAACAAATTCAAATTAATAATATTGACACCAACCACGGATATCTCCTT
TTTTCTGATAAGCCAGTACAGATACCATCCTCCTTTGAACATCACTCCTTAAA
AATCAATTTAACTGAAATAGACATCGTGGTTGACTATTTTGAGCAAAGACTAC
GAACCGATTACCATGCACCCCAGATCAATTTTTTATACAATAAAATAAAAAGA
GAACTAGCCAGAATAACCCTGAAACATAGAAACAAACGGGGTTTTATTAACA
TTGTGGGTTCAGGTTTTAAATACCTATTTGGAACACTAGATGAAAATGATCGA
GTCGAAATACAGAAAAAACTTGAAATCAACGTCCATAACTCAGTAAAATTAC
ATGAACTCAACGACGCCATACGATTGATAAATGACGGAATGCAAAAAATACA
GAATTATGAAAATAACCACACCATCATTGACAGTCTTTTGTTCGAACTAATGC
AGTTTACGGAATACATAGAAGATTTGGAAATGGCTATGCAGCTTTCCAGACTT
GGACTGTTTAACCCCAAATTACTAAACTACGACAAACTTGAAAATGTGAACA
GCCAAAACATTTTGAACATTAAAACATCCACTTGGATTAACTACAATGATAAC
CAAGTATTAATCATATCCCACATACCCATTTACCTTTCACTAATAAGCACAATT
AAAATAATTCCTTACCCAGACTCCAACGGCTATCAGCTAGATTACACAGACAC
ACAATCATATTTTGAAAAAGAAAATAAAGTTTATAATACCGAAAATAAAGAAG
TAAAAAATGAATGTGTCACCAATATTATTAAACACTTAAATCCAATTTGTAATT
TTAAGCCAGTACACACGAACGAAATAATAAAATACATAGAACCAAACACAAT
TGTAACTTGGAACTTAACCCAAACAATTCTTAACCAAAATTGCCAAAATTCA
ATTAATAAAATAAAAATAGAAGGAAACAAAATGATAAGAGTAACGCAATGCA
AAATAGAAATCAATAATATAAATTTTAGTGAAACTCTGTTAGAACCAGAAATA
GATTTGACACCACTATACACACCACTTAATATAACAAAAATAAAAATTGTAAA
ACACAACGACATTATTGAGATGATTTCAGAGAACAATATTACACTTTACATAC
AAATGATCATTGTAATAATCGCACTAATTTTGTTGTACTCATATTTAAGATATGT
ATCATTTAAACCATTTATGATGTTGTATGCAAAACTTAAAATAAGAAAAAATC
AAAATCAAAACACACCACAACAAACAGAAATAGAAGAAATTCCATTTCCCA
CACTATATCCATCAATCCCAGCCCAAGTATAGGCTTCTCTTTAAGGGAAGGGG

>176-LTRGypsy#LTR/176-LTRGypsy

AGTGACATATTCACATACAAAACCACATAACGTAGAGTAAACATATTGAAAAG
CCGCATACGTAAACAATAAGTGACCACCATGCTAATGTGGATCAAATAACAA
AAATATCCACTCTGCATTTTGACACCCCCATACTGTATGCCATCTGCGCAGTAT
GCATTCTAATAAACAAATTCTTTGACAGCGGCACTTAGCCATTCTTGTAAACA
AATCTTAAAGTCTGCCTGCTCTCTCTGAGGCTTCTCCTCCACTTAAGAATCCA
AGAGCAATGCTCTCCCAAAAACACTAACATATTCTTTAAGCAAGCACAGAGG
CTTCTCCTCATTTTCACTTTCATTTGATTTTCAGTCTTAAGCTGAACGTTAATC
AATAAACAACACAATCGATCCCGAAATTTTGATTCGTTTTATTTTGGCAAAAC
TCAATTTTCAGCGTTGGTCTTAGTTCATATTCGGAACGGTCCATTTAATAGACT
CAAAACTATTTATTGCAACCATTTATTTGCAATT

>176-IGypsy#LTR/176-IGypsy

GGCGCAGTCGATGTGATCAGTGTTAAAGTTCCTTGATGCGGTAACCAGATTTG
CCAATTCCTGTGTTCTTTTTGTTCTCTGACAAAAGTACCACGCTAACGGGCAC
CCACGTGACGGTTAATATCGCTTTAAGTTTTTAATTAAACCTCGACAATAAAG
TGAAACCGAAAAATCACAATTTGCCTAAACAAACCTGAATTTATTATCAGGA
AGACGCTATTGAATTTGTGAGAGGCTGTAAATCCAATTGGTTACCTCAAAGAC
CCACGAAAAAGCTATAGTGCAACCCTTGTGAAAATCAAAACCTATCTTAAAA
AAAAAAAAAAAAATATAAATAATAAATTAATAAGCGAAAATTAAAACGTATTA
AAAGTAAGAATAATAAATAAATAAGTGAAAATTCTATATGATAAAAATTAAAA
ATAAGAATAATAAATAAAAAGACAACATTTTAAATTAAACAATATTAAAAAAA
TATAAAAATATTAAAAACTATATTAAAAAAAAAAAAAAAAAAAAACAAAAAA
ACAAAAAAAAAAAATACACGATAATAATAATATAATAACTAAATAATAATAATAA
TAAAAAAAAAAAAATAAATAAAAAAAAATAAATAAATAATCCAAAAATCAAAAA
TGGCTCAAGAACCAGCAATTGTGCCACCACTATCAGACAGCAACATGACCCA
GGTTGCCTACCAGATTGGCAATGTGGAGAAATTCAACGGTGATCCAGGCTCA
CTATACACCTTTGTGAGTCGAATTGATTACATACTGGCTCTTTATGCTACCGGA
GATGAACGCCAACAGCAGATCATATTTGGGCATATTGAACGCAGCATCAGCG
GAGAAGTTATGCGCTGCATTGGAGCCTATGACATGTACACCTGGCAGCAGCT
TAGAAGACAATTGGTACTCAACTATAAACCCCAGACCCCTAACCACGTTCTTT
TAGAAGAGTTTCGAAAGACCCCATTTCGAGGCAATGTACGAGCATTCCTGGA
AGAAGCAGAAAGCCGCAGACAAACACTTACTAGTAAGCTTGAATTAGAGCA
AGATCTTGAAGAAAAGACTTTTTATTTGAAATTAATAAAATCCAGTATAGAAT
CACTAATTGAAAAATTACCTACACACATTTATTTAAGAATAAATAACCACAAC
ATACCAGATTTGCGATCACTTATAAACCTTTTACAAGAGAAGGGCATGTACGA
ACAAATAAATCATACAAGTACACATGTCCAAAAACAAAATTTCTCTGATAAGC
CACAAAAGTCCTTTAATCAAAATACTAATCAGTCTAACAATATCAGAAAATAT
CCAACACCTTTCCTACATTATAATTCACCAATACCATATCAAGCTCCACAAATT
TATCAAACACCACCAACTAATAACCCACTTTATCGTCATCCAATACCCTACCA
CCCTAATCCAAACAATGTTTTTCAACCAAGCCAACAAAACAATGTTTTCCAA
CCAAGCCAACAAAACAATGCTTTTCAACCAAATCAACGTACAAACTTTACAT
CTCGACCAATTTTTAACACCAATCGAAACAATGCATTCGATCAGAATAGGTTC
GGACAACAACCCCAATATCAAAATCAACAATCAACACAAAATTCAAGTTCCT
ATGTACCCAATCGACCAATAAAACGATTAAGACCAGCTAATAGTGGACAGAC
TGGGATGAGTGTTGACGAAACATTATATCAAGAGGACGCTTTTTATCAGCAGT
GTGTTCCATATGACTATTTTTATTATCCAACTTACGACCATTCAGACTATTATCC
AGAAAATCAATATCAAATTGACGAAAACAACCAAAATTTACAAAGAACACA
ACAGTTACAGCAGATTAATACAGACGAGACAAACAATGACAACCAAGAACC
CAATGTTGAACAGGCCGAAAATTTTCAGCCACAAGCCTTGGAAAACCCCAAT
ATATAACAATTAAATACAAAGAAAATAATTTGAAATGCCTTATTGATACCGGAT
CAACAGTTAACATGACATCTAAAAATATATTTGATTTACCAATCCAGAATACTA
GTACTTTTATTCATACCAGCAATGGACCGCTCATTGTCAACAAAAGTATAATC
ATACCTTCAAAGATTTTGTTCCCAACAACAAATGAATTTTTATTGCACCCTTTC
TCTGAGAATTACGATCTTTTATTAGGAAGAAAACTTTTAGCAGAAGCAAAAG
CAACAATAAGTTACCGCGATCAAGAGGTAACTCTTTACAACAACAAATACAA

ATTAATAGAAGGAATAGCAACACATGAACAGAGTCATTTTCAAAATGTAAATA
TGATACCTGACACCATGCTCAGACAGCCAAATAAAATTTCACCCATTTTAGAA
TCAGACCTATACAGATTGGAACATTTAAATAACGAAGAAAAACAAAGATTGT
GCGCACTCCTGCAGAAATACCATGACATACAGTACCATGAAGGTGATAAGTT
GACATTTACTAATCAAACCAAACATACTATCAATACAAAGCACAATCTACCAC
TTTACTCTAAATACAGTTACCCACAGGCTTATGAACAGGAGGTCGAAAGCCA
AATACAAGATATGCTAAATCAAGGTATTATACGTACCAGTAATTCACCTTACAA
TAGCCCCATCTGGGTGGTTCCAAAGAAACAAGATGCATCAGGCAAACAGAA
ATTTAGAATTGTAATAGACTACCGAAAATTAAATGAAATAACAGTAGGAGACA
GACACCCAATCCCAAACATGGACGAAATCTTGGGAAAATTGGGCAGATGTAA
TTACTTCACAACTATAGACTTGGCAAAGGGTTTCCACCAGATCGAAATGGATC
CAGAATCAGTTTCAAAGACAGCCTTTTCTACCAAGCACGGTCATTATGAATAT
TTGCGCATGCCATTCGGATTAAAAAACGCGCCAGCCACCTTTCAACGGTGCA
TGAATGATATTTTAAGACCACTCTTAAACAAACACTGTCTTGTGTATTTGGAC
GACATAATTGTATTCTCGACATCCCTTGATGAACACCTGCAATCGCTCGGACT
AGTTTTCGAAAAATTAGCAAAAGCCAACCTTAAATTACAGCTTGACAAATGT
GAGTTTCTCAAGCAAGAAACCACATTTTTAGGACATGTTCTAACACCAGATG
GAATAAAACCAAACCCTGAAAAAATTGAAGCCATTCAAAAATATCCAATTCC
CACTAAACCAAAAGAAATAAAAGCTTTTCTTGGACTGACAGGATATTATCGTA
AATTTATTCCAAACTTTGCAGACATAGCCAAACCCATGACTAAGTGTTTAAAA
AAGAACATGAAAATTGACACTACCAACCCAGAATATGACTCTGCATTTAAAA
AATTAAAATATCTAATATCAGAAGACCCAATTCTTAAAGTACCCGACTTTACA
AAGAAATTCACTTTAACCACAGACGCAAGTGATGTCGCTTTGGGGGCAGTAC
TGTCACAAGATGGACACCCACTTAGCTACATTAGCCGAACACTTAATGAACA
CGAAATAAATTACAGCACAATTGAAAAAGAACTCTTAGCAATTGTATGGGCG
ACAAAGACTTTTCGACACTACCTACTTGGAAGACACTTTGAAATATCCAGTG
ACCATCAACCATTGAGCTGGTTGTACCGTATGAAAGACCCAAATTCAAAACT
GACCCGATGGAGAGTAAAATTATCCGAATTCGATTTTGATATAAAATATATAAA
AGGAAAAGAAAATTGCGTGGCGGATGCTCTGTCCAGAATAAAACTTGAGGA
GACATATTTGAGCGAACAAACCCAACATAGTGCAGAAGAGGACAATAGTGAT
TTAATTTTTATTACAGAAAGACCTCTAAATACATTTAACAGACAAGTTATATTT
TCAAAAGGACCACCAGACATTAAAGTTACGAAATATTTCAAAAAACACATCA
CCCAAATATTTTACGACATTATGACCAGGGAAAAAGCCGAACAATATTTGATA
GACCATTTTTGTGGTAAGAAAAGTGCGTTGTATATTGAGAGTGACGCTGATTT
CGAAGTCATTCAAGCCGCACATAAATTAGCCATAAACACCAAATATACAAAA
ATCCTGCGTAGCACGATTTTGTTAAAAAACATAACCACTTATGCGGAATTTAA
GGAATTGATCTTGACTGCTCATGAAAAACTTCTACACCCAGGCATACAGAAA
ACTACTAAACTTTTCGGAGAAACTTACTATTTCCCTAATAGCCAGCTACTTATT
CAGAATATAATAAATGAGTGCAGTATTTGCAATCTGGCAAAAACAGAGCACC
GAAATACAGACATGCCAATGAAAACCACACCCAAACCAGAACATTGCCGCG
AAAAATTCATGATAGACATTTACTCATCCGAAGGCAAACATTACGTTAGTTGC
ATAGACATTTATTCGAAATTTGCCACATTAGAAGAAATAAAAACAAAAGACT
GGATAGAATGCAAAAACGCGCTTATGCGCATATTCAACCAGCTTGGCAAGCC

AAAGTTACTAAAGGCGGACAGAGACGGCGCATTTTCCAGTTTAGCCCTCAAG
AGATGGCTGGAGAGTGAGGAAGTCGAATTGCAGCTTAACACAACAAAAACT
GGTGTGGCGGACATAGAAAGACTACATAAAACAATTAATGAAAAGATTCGCA
TAATCAAAACATCCGATGACGAAGAAACCAAATTGAGCAAAATGGAAACAG
TACTTAACATATACAATCATAAAACCAAACACGACACCACTGGACAGACCCC
TGCACACATATTTCTCTACGCTGGACAACCAATATTAGATACCCAACAAAACA
AAGAAAACAAAATAAACAAAATAAATAATGACAGAGTGGAGTACGAAGTCG
ACACAAGATACAGAAAAGGTCCACTACAGAAAGGCAAATTAGAAAATCCTTT
TAAGCCAACAAAAAATGTGGAGCAGACTGACTCTGATCATTATAAAATTACTA
ATAGAAATAGAATTACTCACTACTACAAAACACAATTCAAAAAACGAAAGAA
AAATAATCAGCTCTCAATTTCACAGGCACCTGGCACTTGATAACATTGCTGCT
GATGCTGATCACAACAGTTCATGGACAACAAATTGAAATTAATAATATTGACA
CAAACCACGGATATCTCCTTTTTTCTGATAAACCAGTCCAGATACCATCATCCT
TTGAACATCATTGCTTGAGAATCAATTTAACTGAAATAGACACCATAGCTGAT
TATTTTGAGCAAAGACTACGTACCGACTACCATGCACCCCAGGTCAAATTTTT
ATACAACAAAATGAGAAGAGAACTAGCTGGAATAGCCTTGCGACATAGAAAT
AAACGGGGACTTATTAACATTGTAGGTTCAGTTTTTAAATACCTATTTGGCAC
ACTTGACGAAAATGATCGAGTGGATATACAGAGGAAACTTGAAACAAACGC
CCATAACTCGGTAAATTTACATGAACTCAATGACGCTATTCAATTAATAAATGA
CGGAATGCAAAAGATACAGAATTATGAAAACAACAGCAACATCATTAACAGT
CTTTTATATGAACTCATGCAGTTTACAGAATACATAGAAGATGTGGAAATGGG
AATGCAGCTTTCCAGACTCGGTCTATTTAATCCCAAACTACTAAACTACGATA
AACTTGAGAATGTAAACAGCCAAAATATTTTAAACATTAAAACATCCACTTGG
ATTAATTACAATGATAACCAATTATTAATCATATCTCACATACCTATTAACTTTT
CATTAATAAATACAGTAAAAATAATCCCTTACCCAGACTCGAACGGCTATCAG
CTAGAATACACAGACACACAATCATATTTTGAAAGAGAAAATAAAGTTTACA
ATAACGAAAATAAAGAAATAAACAATGAGTGTGTCACCAACATTATTAAACAT
TTAAAACCAATTTGTAATTTTGAGTCAATCCACACAGATGAAATAATAAAATA
CATAGAACCAAACACAATTGTAACCTGGAATTTAACCCAAACAAGTCTCAAA
CAAAATTGTCAAAATTCATTTAATAATATAAAAATAAAAGGAAACAAAATGAT
AAAAGTAACCCAATGTAAAATAGAAATCAATAGCATAATTCTAAGTGAAAATC
TCTTTAAACCAGAAATAGATTTGACACCATTATACACACCACTTAACATAACA
AAAATAAAAACTGTTAAACACAACGACATTAATGAAATGATTTCACAAAACA
ATATTACACTTTACATATTTATGACTACTGTCATCATTATACTTATTTTATTGTAC
TTATATTTAAGATACGTATCATTTAACCCATTCATGATGCTGTATGCAAAACTAA
AATTAAGAAAAAATCAAAATCAAAACACAGCACAACAAATAGAAATGGAAG
ACGTTCCATTACCCCTACTATATCCATCAATCCCAGCCCAAGTATAGGCTTCTC
TTTAAGGGAAGGGA

>1731_LTRCopia#LTR/1731_LTRCopia

TGTTGAATATAGGCAATGCCCACATGTGTGTTGAATATAGGCAATTTCCACATG
TGCATATGTAATTTTGTATGAGAACATACATACATACACATGAACTGTATGTAT
GTATATATATTAGCAAATAAGCAGCCGCATGAAGGTGGCATTTTTATGTGTATC

AGTTTCAGTTTCAAATAAAACTTCTTCGTGTTCGGACACGCGGCTCAAGACT
TTTTATTTCGCGTTTACTCTTTCAGCCTTTGCTCTCAATTCGCTGAGTTTGGGT
GAAGATTAGGATCTTCCCATTATGATTGTCAGTGTTCCACACTTGGAGCACCT
TTTCAATAAACAACA

>1731_ICopia#LTR/1731_ICopia

GGTTAATGGGCCCAGCGCCCTAGGAGCTGCCTAAAGGAGAAACGTGTAGTG
AAACTCAGGAGTTAGATTTTGGAGTCTACTCAAGATTGCCGGAATGAGTAAC
CTGTATCAGATCGATAAGCTGGAGGATGGATCCTATGAAACGTGGAGCATCCA
GATGCGTTCAGTGTTGGTGCACGCATGTTTGTGGAAGGTGGTTTCAGGAGAG
TCCGTGAAACCTGAGGTTGATACTGGAGGTGCTTGGCAATCCCAAGATGAAA
AAGCATTGGCCACGATCATCTTGAGTGTGAAGTCTTCGCAACTTGGTTATGTA
AAAGGGTGTCTCACTGCGGCTGAGGCATGGAAAGTTTTACAGGATGTCCACC
AGCCGAAAGGGCCGTTACGAACGGTCATGCTGTATAAGAAGTTGCTGAGCAA
ACGTCTGTTGGAAGGGCAGAGTATATCGTCACATATTAAAGAATTTAAGGAAA
TCTTTGATGCCCTTGATGCGGTGGAAATTGGTATCACCGAGAAATTGCGCAGT
GTTGTTTTGCTGTCGAGCCTTCCAGAGAGTTTCGAGAATTTCGTTGTCGCCAT
TGAGACGCGCGACGACGTGCCGCTTTTCGATGCTCTATGTATAAAGCTGATCG
AGGAAGACACGAGAAGGGGAGGAGCGGAGCAGCAGAGAGAAAAACAAAC
GGAGAGCGCAAAGGCATTTACTGCAGTACATAAGCCACAGGCGCCGGCGAG
AGAAGCTCGGCCGAGCGCAAAGAAGAGGAAAGACGTAGTTTGTTATAACTG
TGGAGAGCGTAGGCATTTTAAAGCGAACTGTCGTCGCGAGAAAGTAAACAA
AGAGAGCGCGACACAAGAACAATGCAGTTTGTTAAATGCGCTGGATAGTGGT
GGTTTTTGGCAAAACACAGTGGTGTCTCGATAGCGGGGCTACCAGTCACATG
TGCTGTGACAGAAGTGTTTTTACTGAGTTTGAAGAGCACACTGAAAAAATTA
GTCTTGCTGGAAATGGATTCCTACTAGCAAAGGGCATAGGAACAGTGAAGCT
GAAGACTGATTTATGTACTCTGGTATTGAATAACGTACTCTTCGTCCCAGATTT
GAACGGCAACTTTATGTCAGTCAGCCGTGCAGCTCAGTATAAATGTTTTGTCA
ATTTTGGACCACATTACGCTGACGTCATTCAGGAAGGCGAGCGAATACTGCG
TGTAATGAGAGCTGGTAATTTATATATGTTTCAAGGGAAACATAACAGTTGTTT
TGCGGCCGTTGATGCTGATGGTTCACTATGGCATAAAAGGAATGGCCATTTGA
ATACAAGCAGCCTACAGGAGATGGTGAGGAAGAAGATGGTGTACGGTGTTG
AAAAGGTCGTTTTCAAACCAGACGCAGTATGCAAGACGTGCATGCTGGCAA
AAATCCATGTGCAACCATTTCCGAAGACAACGAGGAGCAGAGCTGAGGAGC
TGTTGGATATGATCCATTCAGACCTGTGCGGGCCATTTAGCACACCGTCACTT
GCTGGATCAAAGTACTTTCTCACTTTCATAGACGACAAGTCCAGGCGGATTTT
TGTATATTTCTTGCGGAAGAAGGACGAAGTCTTCACTAAGTTTGTCGAGTTTA
AGAAACTGGTCGAGCGACAAACAGGTAGAAAGATAAAATGTATCCGGAGCG
ATAATGGTGGTGAGTTCGTCAATAATGTTTTTGATGACTATTTAAAGGCACATG
GGATCGCTAGACAGCTGACTATTCCACACACTCCCCAACAAAATGGAGTTGC
AGAACGAGCCAACCGCACGCTAGTAGAAATGGCTAGGTGCATGTTGCTGCAA
TCGGAGTTGGGTGAGGCTCTATGGGCTGAGGCGATAAACACTGCGGTGTATC
TGAGGAACCGATCAACGAGCAGAGCATTACAAAGCAAAACCCCTATGGAAG

AGTGGACCGGAAAAATACCAGCAGTGAGCCACTTGAGGGTTTTTGGTGCCAT
AGCAGTGGCATTGGACAAAGGAGTCCATAAAGGCAAATTCGAATCCAAAGG
AAAGGAATATCGTATGATTGGATATTCAATAGCTGCTAAGGGGTACCGTCTGT
TTGACAAAGAGAAGCGGTGTGTGATCGAGAAGCAAGATGTCCTTTTTGATGA
GTCTGGTAGTTTGGTAAATCATGGAAATACCATTGAGTTCCAGTTTCCCGCAA
CTGATGACCCGGAGCCGCAGAGTGATTCGAATGCACGGGAAGGTGACGATA
CAGAACCCGTGGGCAGCAGCGACGACTATGAGAGTGCAGCTGAGGCAGAAG
AAGCTGAAGTACATGTGGGGCCTGGACGGCCAAAGATTGTTCGGACGGGCA
GACCAGGGCGCCCGAAGAAGCAATACAATGTACTTGGCGTGTTGATGGCTAG
CGACGTCGAAATTCCCAAGTCCTATGAGGAGGCCATCAATTCGCAGTATTCTG
CAAAGTGGGAAGAGGCAATGGGCCTGGAGTACAAGGCGCTACTTGCAAATG
AGACATGGAAGCTGGCTGACTTACCAAGAAATCGCCGGTGTGTGGCTTGCAA
GTGGGTGTATTCCCTGAAACGAGACGTCTCTGGTAGAATTGAGCGCTTCAAG
GCACGACTAGTAGCAAAGGGGTGTTCGCAGAAGTTCGGAGTGGACTACTTC
GAGACTTTTTCACCCGTGTGCAGGCTCGAGAGTGTGAGGCTCATTTTGGCAT
TGGCAGCAGAGATGCAATTGTACTTGCATCACATGGACGTATGCACGGCGTA
CTTAAATAGCGAGCTAAAGGATACTGTGTACATGAAGCAGCCCCAAGGGTTC
ACAGATGCTGCTAATCCCGACCAGGTGTTATTGCTGAGGAAGGCAATATACG
GCTTGAAGCAGTCAGGCAGAGAGTGGAACTCCAAGCTCGACGGTGTTCTAA
AAGACTTGGGATTTAAGGCCTGTAATCATGAACCATGTCTTTATCAGCAAAGT
GGTCAAGGTAATCTGATGCTCATCTTAGTATATGTTGATGATTTAATTCTAGCG
TGCCAGTCAAGAGAAGATATGGAGGATCTGAAAGCCAAGATTTCAGAGTCTT
TCGAGTGCACGGACAAGGGTCCACTGCATTTGTTCTTAGGCATGGAGGTGCA
ACGAGATGGCGACCTTGGAGAAATCACTTTGGGCCATTCGCAATATATCAAG
GAACTATTGCGGGATTATGGCAGCGAGAACTGTAGACCAGCGACGACACCTT
TGGATGCAGGGCATCAAGTTTTGTGCGCGGGTGAGCAGTGCCAGAAGGTCG
ACGCAGGGCAGTATCAGTCTACAATTGGTGAGCTAATGTGGCTTGGGCTTACT
ACCAGACCAGACATGCTACATTCGGTGGCGAAGTTGGCTCAGAGGAATCAG
GACCCGCATTCTGAGCACATGGTGGCTGTGAAGCACATCCTCCGGTACTTGG
CGTCAACTGTGGACGTCAAGCTGCATTATCAAAAGTGCGGTCAGGCATTTAC
CGGCTTTGTGGATGCAGATTGGGGAGGCGACCGTTTGGACCGAAAGTCATAC
ACAGGGTATGTGTTTTTCCTGTCTGGCGGACCAGTATCATGGAGGTCCGAGA
AGCAGCAGAGCGTGGCGTTGAGCAGTACTGAAGCCGAGTATATGGCTCTGAC
CACGGCTTGCAAGGAAGCTATAGCTTTACGAAGGCTAATAGTGGAGATCGTAT
GCGGTGATCTGAAGACCCCGACGGTTATGCATGGCGACAACCTGAAGTGCGC
AGCACAGTTAGCGAAGAACCCGGTTCATCACTCTAGGACGAAGCACATCGA
CATTCGATATCATTAGAGAAGTCATGAAAGAGGGTCACGTTGTGTTAGAGTAC
ACTTCTACGAATGAGATGATAGCAGACATTATGACAAAGAATCTTTCAAAGG
GAAAGCATAATGGGTTTATGAAAATGTTAAATTTGTTTTAATTTTTGTAAACAT
GTTGGCATTGAGGAAGGCT

>DIVER_LTRBEL#LTR/DIVER_LTRBEL

TGTACGGATTATGAGCGGAGTCACCCGGTGCTTCAGCTGTCTTAACAGCGGA
TTAACATTTTATATATCGATGCTAATTGAACTGAACTGTATTCTTTCTTTCGCTC
TTGTAATTTTGCGGTCATAGCGATCAGACGTGATTTTTGTATATGAAGAAATAA
ATGAAGTTAAATAGTGTATATATGATTCTTATTTGCGAACCCCATTACAATGAT
GTCAAAGCA

>DIVER_IBEL#LTR/DIVER_IBEL

TTGGTGGCCCATGAGGGGAACCTTGCTCATAAGAATCATAATTAATAATTGCT
AAAAGCTGTTTGGCAATTTTATCTTCGTGGTCGGCTTCATCCAGATAAATCAC
CGTGCCTACGTTTTCTATCGCGCTCGCTCTCATCACGACAGAGCAGTATACGC
AAACATTCGCGCTCGCTCTCATCACGACAGAGCTAAGTCCCGTTGCCCTTCG
ATTCCGTTAGAGTTGCCGGAATCTCTGCCAACTCAAAAAAACAAGACGTCAA
AGAAGAACACCGATAACATACGAGCATTTGTGCATATTACATACAGTGGAGCA
ACGTCAGTATAAACAATGTCGAAATTTGATCAACTGGTGCGAGTTCAAACTG
ACCGCATTGAGTCTTTGAAGCGCTTGTTCAGTAATGTGAAAAAAGACTCGAG
TGCGCGGAAAACGGAAATATATTTCGAAAAACGTTTGAATCAAATCGACGAG
TTTAACAAAGAATTTCATCAGGCGCATCACACACTTATATGCATGGCAGACTA
CGAAGAAAGTGTGTATAAGCAACAAAATACAATCAGCCAATTCGAAGACCTG
GGCATGGAAGTTTACTGTTTCGTGGCCGAAGAAAAGAAACGAATTTATCCAG
GCACAGTGACGCACAACGAATCAGCTAACTCTACAATAACAACACATGTAGA
GGAAGTACGGATACCACTGCCAAAATTACCAGTTCCGAAATTCTCTGGCAAC
TGCGCGGACTGGCCAAGTTTTCATGATGCATTTTTACGCTTAATTCACAATAAT
GAGCGTCTGGATAAGATTCAAAGGTTCCATTTTTTAAAGGAAGCATTACCAGT
AGGCCTGGACAACGACATTCGCCAAATCGCTTTAACTGAAGCAAACTATGAA
GTCGCTTGGACCACACTTCTACAACGATATAACAACCCACGAATTGTGTTCGC
CAGCCACATGAACATGCTCTACAACTTACCGAATCTTTCGAAAGAAAAATCT
GCTGACATACGGTCTATGGTTAGTACAGTCAACGTCTGCATTGCCGCTTGCAA
TACCGTCAAGGCGCCACTACAGGGAGGAGATTTTTGGTTGACTCATTATCTAA
CAACCAAACTACCCAAAGACACTCACACAGCTTGGGAGCATCATCTGGGCA
GCAAGATTGACGTTCCTTCATACAAAGATCTGCAACAGTTCCTCAATGATCGA
CTTGTTACGTTAGACGCTATTGAAAGCCGTAACGCGTGCAGCGGCATGAAAC
AGTCAAATGAAACTTCAGACGGTACTAAACGCGTGCGTGTGCACAGCGCCC
ACACCAGGTCGGGTGCTTCCGCGTCCGCTTGCTATCATTGTGGCAACTTACAC
ATACTTCGAAGATGTCCGCAATTTCTTTCAATGGATTGCTACCAACGGAAGGA
AGTGGCCAGCAAGGCAAAATTGTGTCTCAATTGCCTGGGAAAATCGCACAC
ACAAGCAAGCTGCCCCAGCAACAAGAATTGTCTTCATTGTGGTCAACGTCAT
CACACGATGTTACATTTTCCAGCAACACAGCCAACGCTGATACCCTCCTCGA
CATCATGCCAAGGTTCAGCAGCCAGTTCAGATGCCAAGCCGACTCTACAGTG
CATGTCAACTACAACTTCATCTAAGACTCATCGAAAGGTACTACTAGCAACA
GCTCGGGTTGTACTCAGTAACACACAGACAGGATGCCAGGCCACGGTAAAC
GCGCTTCTTGACCAAGGATCGGAAGCAACTATCATTTCGGAGCATGCTGTAC
AGTCTCTCCAGCTCTCCCGAAGCACAACTCGCACTTCAATCACCGGAGTCGG
TCAAGATTCAGGACGACGCTGCAAATTCATCGTAAGTTGTTCGGTGCAAACA

GCAACTAACCCAAATTTCTCGTTAAAGGTCGACGATGCTTATGTTTTGAACAC
GTTGACATCACACATGCCAAGTCAAAGTTTTCCAGCAGGAAACTGGAGTCAT
ATCCATGGTCTCATGCTCGCAGACCCCTATTATTATAGATCCAAGCGAATCGAT
ATCATTTTTGGAGCCGACCTTATGGCACAACTCTTGTTACCTGGAACTAAGAT
TGGCTTGCCAAACGAACCCATAGCGCAGAACACTCAACTCGGATGGGTTCTG
TTAGGCAATGTTGGCAACACGCATATTACACGCCACATTCGATGTAATCATGC
CATAATAAACTCCGAAGAGCTGCTTAAGGTATTTTGCGAGGTAGAATCAGTTC
CAGAACGCCCAAAGCTCTCCAAAGAAGATCAATGGTGCGAGTCTTTCTTTAA
GCAGACCCATCAACGTCAACCAGACGGCAGTTATCAAGTCCGTTTGCCATTC
AAGAGGAACTTTGACCCCAGTATGACGCTCGGAAAATCTCATCAGATCGCCC
TGAACAGATATCTTCAACTCGAAAGGCGCCTTCAAAGGGACCCAGACAAATG
GATCAGATACTGCAAAGGAATTGAAGAATATTTTCAACTAGGTCAAATCACGT
TGGCAGAGACGAGCGAAAACTCAACCATAACCACGGATTCCTACGGTCGGC
ATGTTGCATCATGCGTGCTACCACATCATGCAGTTTTCAAAGAAGAAAGCCTC
ACCACAAAACAACGTATCGTTTTTGACGCATCGGCCCGGACCTCAAATGGCA
GATCGTTAAACGATGTACTATGTGTAGGTCCCACGCTGCAAAATGATCTGCCA
GCCGTTCTCTTGAATTGGAGACAATATCAGTTTGTTTTCACTGCCGATATACA
ACGAATGTATCGCTGTATCAATGTCCACCCTGATGACACCCATTACCAGAGGA
TTTTATGGCGGCCGGCGGATGGAGTCATCAAACAGCATTGCTTAACTACCGTC
ACGTTTGGAACAGCGTCTGCACCTTATACAGCTATAAGAGTCATCCATCAAAT
AGCTGAAGACACACAGACAAAATATCCTATGGCATCCAACGTTCTCAAAAAT
GAGATATATGTCGACGACATTCTCTCAGGCGATCATTCACAAGAGGCAGCAAT
ACGGAAAAGTTTGCAAACTATGCTAGCTTTAAAATCCTCTGGCATGGAGCTA
CGAAAATGGGCTAGTAATGACCAGGATCTGATGGCAACGATACCTCTCGAGC
ATCGATGCAAGCAGACATCCCTCAGTTGGGACAATGCGGACACCATCAAAAC
ACTGGGTATGTACTGGTTGCCCAAGCAAGATTGCTTCACTTATAAATTACTAG
CAAATACTCCAGCCGGTATAACAAAACGAGAAATCCTATCGACCATAGCACG
TTTATTTGATCCTCTTGGATTAATCGCTCCAGTTGTAATTTCAGCAAAGATTAT
ATTGAAAGAAATCACACTAGCGAAGCAATATCGCGAGGACGGATCGAGTACC
TCACTGGATTGGGATGAGCCAGTTCCCAACACCATTGCCGTCAAATGGCAAC
AATTTCGACAGCAACTAATGAAGGTTAAGACGATCAAAATACCACGCAGCGT
CAAATTTACGCCACTATTTAGTAGTGAGATACAACTGCACACCTTTTGCGATG
GATCCTCCAGTGCTTACGCAGCAGCAGTGTATGCACGCACCCAACAGTCTGA
TGGGACTTTCTATACAACGCTCATCGTCGCAAAATCAAAAATTTCGCCAACCA
AGCCGTTAACAATACCGCGCACTGAGCTATGCGGTGCAGTATTAGCTACCAA
ACTCCTCAAATGGGTGCTGGAGAATAACCGATGGACCAATGCACATATATCTA
CCTTTTACTGCACCGATGCTACCATTGTTCTGCACTGGATTAAAGGAGACATT
ACTAGGTGGAAAACGTTTGTAGCCAACCGAGTGTCTTACATTCTCGACCACA
CCTCAGCGGCTCAGTGGCACCACATAGACACATCGGAAAACCCAGCAGATTG
CGCAACCAGAGGCTTACCACCGAGCCAAATACCTGATATTTGGTGGCATGGC
CCATCCTGGCTATGCAAACCACACAATATTTGGCCAAACACGCAATCACAATT
GCTCAATCCAGAAGAACGGGATCTGGAGGCCAAATCCATAAAAATTAGAGCC
TTCACTACCTTGTCAGACACAAAGGATTCTATTATTGACCGATTCTCGTCGTAT

ACAAAACTGCTACGTGTCACGGCATACATGTTACGATTCTGCCACAATGCTCA
TGCTCGAGCACAACGAAGTCACGGATCACTCTCTCCTGACGAGCTGGATGAG
GCCCTGTGCTGCATAGCTCGCCTTGCACAATCCGATACATTTCACGCCGACAT
CCAAGCGCTTAAAAGGAACAAGCCGTTACCACCCCGTAGCACACTTTCAAAT
CTTACACCGTTTCTTGACAACAATATCTTGAGGATTCGTGGCCGTCTCAAGCA
TTCAAATCTCTCTTTTTCTCGAAAACATCCAATTATATTACCTCATTGTCACCT
ATTTACAGATTTGGTAATTCAACACTCTCATCAGCTCACTCTACATGGCGGCG
CTCAATTAACACTGGCTCACATACGCTACAAATTCTGGATTCCCAGAGGCAGA
CAAGCAGTCAGGCGAATCATCCGGAAATGCGTCACATGTTTTAAGGTAGCTC
CAGTCGTAGCGAAGCAATTGATGGGTGACTTGCCATTACATCGAGTCAACCC
CCCAACTCGTCCGTTCATTACAACAGGAGTTGACTACACTGGTGCGATTGAA
CTTCAAGCCGCGCGTGTACGAGGATCAACCACCTACAAAGGCTATGTAGCCA
TTTTTATATGCTTAGCAACCAAGGCGGTCCACTTGGAAGCCGTCACTGGACTT
TCAACAGAGCACTTCCTGCAAGCATTTACGCGATTCACCGGACGTCGAGGAC
AAGTTCAACATATGTATAGTGATAACGGCACAAATTTTGTTGGCGCGAGTACA
TCGCTTAACCAGCCCATCACTTGCAAGGCAGCCCTAAACGAATCGACATGTC
AACATGGGACAAGGTGGCATTTCACACCTCCATATTCTCCCAATTTTGGTGGC
ATCTGGGAGGCAAACGTGAAAGCAATGAAGCATCATCTTAAACGGATCGTCG
GCAGCCACAAGCAGACGTATGAGGAGCTTACTACAGTTTTGATCAGGATTGA
AGCATGTTTGAACTCACGTCCACTTTGCCCGCTAACCGCCGACCCTGACGAT
TTAGAAGTACTAACTCCAGCACATTTCTTAATTGGTGACGCACTACTGGCACC
GCCACAAGGTCGGCCGAATAATAAGCCTTTGCGTGAACTATTTCTCGCACAA
CAACACATGACGCGACAATTCTGGTCTCAATGGTCTCGTGATTGGTTATCACA
CTTGCAAACTCGGCCAAAGTGGTGCCAAATCAAAGATAATCTTAGCATCAAC
GACTTAGTCATTATAAAGGATGATAATCTACCACCAGCTAAATGGACTATAGG
CCGAGTCGTCGAACTGCACCCTGGATCGGACTCGCTAGTCAGAGTGGTTACA
TTAAAGACGAAATCTGGCATTCAAAAGAGGTCAATCACAAAGCTTTGTCCAC
TTCCGATTTCAACATAATTATGATCAACACACGGATCAAGAATCACAAGGAGC
CTTCATGCTGGACCACGCATTGGCGGGCGGCA

>DIVER2_LTRBEL#LTR/DIVER2_LTRBEL

TGTTGGGTCATGCAGCCGCCAAAACTGTGCAGTAGACTAATCTCATACTCTTT
AATATTGTAAGCCGAAGTAGTCAGTGGTAGCAGTTGCGATGCCCATAGTGCA
AACCGCTGTTCTCGCACGCATTTATCTGTACGGCGCTCATTCCTTGTTCTTTTT
GTTATCTACCTACGTTAAGCTTGGGCGCTGCATTTCGGCTGTCTGTCCCGCCA
ATTGCCACTTCTTCGTCTGTCGGAAGAGACTAAACTTGTGCATTCGATATAGC
TCTTTGTCGGCCCTAGCTGCTGTAAACAATTCGCAAAATAAACAGTATCGTAA
AATAAACAAACTTTCTTCGGCTATTTATTATTCGCAACAGCTATTGAAAAAGC
TCAATAGCTAAACA

>DIVER2_IBEL#LTR/DIVER2_IBEL

TTGGTGACCCCGACGTGATAGTGTTCATTTGCATGCATTAATTAATGCACTTAT
CCCGTTAATAAATAAATATAAATACATTAAATCGCAATCGGTTGGACAAGTGA

GTGGTGATTTCGGTGTTAGTGGCTGTGTTTGAGCGAGCTAGCATTACATATGT
ACATACATACATTGCTACATATATCGTTACGTGCATTGACTCCGCTGGCATTTT
CGGCATATATTTTGTGCTCATCTTCCCGCTGAACCGAATTAAAGGCGATTTGTT
GCCATGCCCGCTGAAGGTAACAAAAACAGGGTGACCTTTTTAAAGCGCAAA
GCCAATGCGCTGTTCAGCAGGTTGCAGAGGCTACAAACGTCGCTGGCTAATG
AGACGCTAAGCGGATACGATGAACCCACTCTTACGGTGAGGCTGGAGCACTT
TGCAAAACTGCAAAGTTTGGCGGAGGCTACGCAAACTGGGCGGATTTTTACT
CCGTGTTCACGAGCATAATCGACAGCCATCCGGACCTCTCCAACATTGAGAA
ATTTTAACATCTGCGGTCATGTTTGAGGGATTCGGCGCTGGAAACTATCCGAT
CATTGGATATTTCAAACAGCAATTACGAAGCGGCTTTAGAGTTGTTTCAAAAA
AGGTTTGATGAATCGGCGTCTCGTTTTTCAGGCGCACATCACCGAGATTCTGG
GTTCAAAGGTAGTACCGGAATGGTTCAGTGGCATCGCTTCGGGAATTGTCGG
ACAAGTTTAACGCTCACATTCGTGCGTTGAAGGGTTTGGGCACCACTGAGCA
AATCGCTGGCTGCATCATAGTGCAAGTGCTGCTGCAAAAGCTGGATGCGGCG
AGCCAAGCTAAGTGGGAGGAGAGCTTGGAGGATCCGATCTTTGCCAACCTTA
TTCCGTCGTGGAAATCGATGGCTGCATTCCTGGAGCAGCGATGTAGGACTATG
GAGGCCGCGGATTGCGCCATGGCAACCTATGCGCCAGGCGTTCAGGTGGGCA
GAAATCGTTCGACGCTAGTTGCTACCACCCAAAATTCTCTTGGTTGCATGCTT
TGCCATAGTGCAGAGCATGCCATATATTATTGCCCGCAATTTACAGACTTAGCG
CCAGTAGATCGTCTGCGCGAGGCAAACAGACTATCACTTTGCCTAAATTGCCT
AAAGGCAGGTCATCAGCTACGGCAATGCAGCTCGAGCCGCCAACGCACCTG
TGGAATCAGGCATCATACGCTGCTCCATCTAGGTGGTCCGCCTTCCTCGCAGC
CACATGTTCCGGTGTCTTCAAGCTCCCACACTGAGCCTTCTGCCCCTCTTTCG
ACTTCTTCTACTCTAATTGCCCAGGATCTCGGTAGTGACCTTGTGCTGCTAGC
CATTGCAACCGTTCTAGTGCAGAATCGGTCGGGACTGTTCGTTCCCTGCAGG
GCCTTGTTAGATTCTGGCTCTCAACTGCACTTGGTCACCTCTCGGTTTGCAAA
TCAACTGCAACTTAAGAGGTCAAGGTCGTCCGGCTCCGTCACTGGAATCGGG
GATTCCAATTTCGCGACTGTTTCGGTCGGTCACTTCGGATTTCTCAACGAGCA
TAACAGCAGTTATCGCTCCCAATATCACGGGATCGCCAGACAAGTTTTAATGT
GGACATTGGGGACTGGAAGATTCCAGAAAACCTGCAGCTCGCCGACCCGGA
ATTTCATATAGCTCAGCGTGTTGACCTGTTAATAGGAGCTAGCTTGTTTTATGA
ACTGCTGTGCCGTAGGTCAGATAAGGTTGTTGCCCGGACTGTCCCTGCTTCA
AAAAACTCGTCTGGGCTGGGTTGTGTCTGGAGGCTGCGCGCGCCCTTGCGG
GTAGCGCCTTAATAGCTTCACGCGTTCCTTCTTCAGCCAGCAAGGAAAACAG
CATTTGATGTTGAACTTGATTCACTTCTGCAGCGCTCTTGGGAGGTAGAAAA
GTGTCCCGGCCCAATAGTTCAAGCCACTAAGGAGGAGTTAGATTGCGAGGCC
CACTTTGGTTATAAATTACACCCGACTGCCAGCTGGCGATTACTCGGTACGGT
TGCCGCTAAAACTCCATTTGGAGTCTTTAGGAGATTCCTATCCTCAGGCTTTG
CGGAGATTCTGGTCGCTGGAAAGGAAGCTTACAAAGCACCCTGGCTTGAGG
GTTAAATATTCGGCGTTCATGAAGGAATATCGTGATCCGGAACATATGTCGCC
TGTGCCTGCCTCCGAGGTCAGCTCGTCCCGATATTTTTTTACCACATCACTGC
GTCATGAAGGAGGATAGCACTACCACCAAGCTTCGCGTTGTCTTTGACGGAT
CAGCTGCCACTTCCACTGCTTACTCGCTTAACGATGTGTTAATGGCTAGCCCG

GTTATCCAGCCCAAGCTATTTCACATCCTAATTCGATTTCCGCTCACACCCAGT
TGCCATTCGGGAGCTGACAGATGTCACGGCTTGGCGTTATGTTCCAACAGCG
TTAAATCCGGCTGACATCTTATCCAGAGGATCCCTGCCGTCTGAGCTTAGCGA
ATCGTCACTCACCCGCCTATCTTACGGAGCCTGAAAGAGATTGGCCAAAAGC
TGTTTGTCCTGACAAACCGGTGCTTGAGCTTCGTCGAAGTGTGTTCGTCGTA
AAGTCGCCGTACGTGGATGTAATTGCTAGCTCCAAATTTGCAAATTCTTACCC
CTCACTGCAACGAGTGTTTGCATACATTTACAAATTTTGTAATGGAATTCGTC
ATCCTGGGCTCACCGTCGCACACATTCAAGAGGGTACTCATATGATGCTGCGG
TTGGTGCAGCGCGCGCAGTTATGGGAGGACCCTGCAGTCCCTAAAAACGCTG
GGAAGAGTTTCCTCGTCTAGTCCCATATCCTCGCTCTCGCCGTTCCTGGATCA
ATTTGGACTTCTTAGAGTAGGCGGTCGCCTCTGGAATTCGTCATTGGACTTTT
ATGGCCGCCACCCGAAAATCCTTCCAAGGTCCCATTCGGTGACTCTGGCAAT
TATTTCGCATTACCATGAAATCAATCTTCATGCCGGACCTCGAGCTCTTCTGG
GTGCAATTCGATCACAATATTGGCCTATTGGGGGGAGGAAGACGGTTACCAA
GGCCGTGAACAGGTGCATCAGATGTTTTCGGATTATGCCGCGGCTGATAGAG
CACATAATGGCGGACCTTCCCAAGGAGCGCTTGGAAGGATCTCACGCTTTCG
AGGTTGCTGGTATAGACTTCTGTGGACCCTTCTTTCACAAGTCGGATACTCGC
AACAAGCCCGCGGTTAAATGCTACGTCTGCGTATTTATATGCTTTGCAACCAA
GGCAGTGCACCTGGAGCTGATCAAGGATCTCTCGACAGTTGCGTTTCTGTGC
GGACTCAAGAGGTTCATATGCACCCGGCGGAAGCCGAAGCAAATTTGGTCA
GACAACGCCACCAACTTTGTCGGCGCCAAAAATAAACTTCTGGAACTTCGAA
GGTTATTTCTCAGCAGCGAGCATCAAGGCTCCGTTCAGAATTTTTGCCTTTCG
GAGACGATTGACTGGCGGTTCATCCCTCCACGGTCGCCCCATTTCGGTGGAC
TTTGGGAAGCAGCGGTGAAAACGGCCAAACATCATTTCTACCGCGCTGTGGG
TACGGCGGTTCTGACCTTCGACGAGCTGAGGACGCTGGTGTGCCACATCTCG
GCAGTTATTAACTCCAGACCTTTAGTTCCCATTTCAGAGAACCCTGCCGATCT
GGACGTCCTCACTCCGGCGCATTTTCTCAATGGTGGTCCGCCTTCGTCGTTTG
ACGAGCCAGATATAACGGGCCTAAACTATAATCGGCTTGACTCTTGGCAGCG
CATCTCCTTTCTTCAGCAAATATTTTGGTCACGATGGAAGGAAGAGTACTTGA
CGTTGCTCCAGCAGCGCTCCAAGTGGCGCACCCCAAAGCCTGGCGTAGCCG
TGGACAACGTCGTTCTTGTTAAGGACGAGAATCTACCCCCAATGAGATGGCC
TTTGGCGAGAGTCATGCAGTTGATTCCTGGCAGAGACGGCGTCGCTCGAGTT
GCAGAATTGAGGACCGCGTCTGGAGTAATAAGGCGGGCAGTGAACAAGCTG
TGTCTGCTTCCCCTTGAGGACTCTGTTGGAAGCCAAGCTTCCAACGGGGGA
GGA

>COPIA__LTRCopia#LTR/COPIA__LTRCopia

TGTTGGAATATACTATTCAACCTACAAAAATAACGTTAAACAACACTACTTTAT
ATTTGATATGAATGGCCACACCTTTTATGCCATAAAACATATTGTAAGAGAATA
CCACTCTTTTTATTCCTTCTTTCCTTCTTGTACGTTTTTTGCTGTGAGTAGGTC
GTGGTGCTGGTGTTGCAGTTGAAATAACTTAAAATATAAATCATAAAACTCAA
ACATAAACTTGACTATTTATTTATTTATTAAGAAAGGAAATATAAATTATAAATT
ACAACA

>COPIA__ICopia#LTR/COPIA__ICopia

GGTTATGGGCCCAGTCCATGCCTAATAAACAATTAAATTGTGAATTAAAGATT
GTGAAAATAAATTGTGAAATAGCATTTTTTCACATTCTTGTGAAATAGCTTTTT
TTTTCACATTCTTGTGAAATTATTTCCTTCTCAGAATTTGAGTGAAAAATGGA
CAAGGCTAAACGTAATATTAAGCCGTTTGATGGCGAGAAGTACGCGATTTGG
AAATTTAGAATTAGGGCTCTTTTAGCCGAGCAAGATGTGCTTAAAGTAGTTGA
TGGTTTAATGCCTAACGAGGTAGATGACTCCTGGAAAAAGGCAGAGCGTTGT
GCAAAAAGTACAATAATAGAGTACCTAAGCGACTCGTTTTTAAATTTCGCAAC
AAGCGACATTACGGCGCGTCAGATTCTTGAGAATTTGGACGCCGTTTATGAA
CGAAAAAGTTTGGCGTCGCAACTGGCGCTGCGAAAACGTTTGCTTTCTCTGA
AGCTATCGAGTGAGATGTCACTATTAAGCCATTTTCATATTTTTGACGAACTTA
TAAGTGAATTGTTGGCAGCTGGTGCAAAAATAGAAGAGATGGATAAAATTTC
TCATCTACTGATCACATTGCCTTCGTGTTACGATGGAATTATTACAGCGATAGA
GACATTATCTGAAGAAAATTTGACATTGGCGTTTGTGAAAAATAGATTGCTGG
ATCAAGAAATTAAAATTAAAAATGACCACAACGATACAAGCAAGAAAGTTAT
GAACGCGATCGTGCACAACAATAATAACACTTATAAAAATAATTTGTTTAAAA
ATCGGGTAACTAAACCAAAGAAAATATTCAAGGGAAATTCAAAGTATAAAGT
CAAGTGTCACCACTGTGGCAGAGAAGGCCACATTAAAAAAGATTGTTTCCAT
TATAAAAGAATATTAAATAATAAAAATAAAGAAAATGAAAAACAAGTTCAAA
CTGCAACATCACACGGCATTGCGTTTATGGTAAAAGAAGTGAATAATACTTCA
GTGATGGACAACTGCGGGTTTGTCCTTGATTCTGGTGCTAGTGACCATCTTAT
AAATGATGAGTCGCTGTATACCGACAGTGTGGAGGTTGTGCCTCCACTTAAG
ATTGCAGTGGCCAAGCAAGGCGAATTTATTTATGCCACTAAGCGTGGTATTGT
CCGACTACGGAATGACCATGAGATTACACTGGAGGATGTACTCTTTTGTAAGG
AAGCTGCTGGTAATTTGATGTCCGTAAAGCGTCTCCAAGAGGCAGGAATGTC
GATCGAATTTGACAAAAGCGGTGTAACCATTTCGAAAAATGGGTTAATGGTT
GTCAAAAATTCAGGTATGTTAAACAATGTACCTGTGATCAATTTTCAAGCATA
TTCTATAAATGCTAAGCATAAAAATAATTTTCGTTTATGGCATGAGAGGTTTGG
CCATATAAGCGATGGCAAATTATTAGAAATAAAACGAAAGAATATGTTTAGTG
ATCAAAGTCTTCTAAACAACTTAGAGTTATCATGTGAAATTTGTGAACCCTGT
TTAAATGGTAAACAGGCAAGACTTCCTTTTAAACAATTGAAAGATAAGACCC
ATATTAAAAGACCACTTTTTGTAGTACACTCAGATGTCTGTGGGCCTATTACTC
CAGTTACTTTAGATGATAAAAATTATTTTGTGATCTTTGTTGATCAGTTTACAC
ATTATTGTGTAACTTATTTAATTAAATATAAATCTGATGTGTTTAGCATGTTTCA
AGATTTTGTAGCCAAGAGTGAAGCTCATTTTAATTTAAAGGTTGTGTACTTAT
ACATTGACAATGGTAGAGAATACTTGTCAAATGAGATGAGACAATTTTGTGTT
AAGAAAGGAATTTCTTATCACTTAACAGTGCCACATACACCTCAGTTAAATGG
TGTTTCTGAGAGAATGATAAGAACCATTACGGAAAAAGCTCGAACCATGGTT
AGTGGTGCAAAGCTAGATAAAAGCTTTTGGGGCGAAGCAGTATTAACTGCTA
CTTATTTAATCAACAGAATTCCTAGTAGAGCACTTGTTGATAGTTCAAAGACC
CCATATGAGATGTGGCACAATAAGAAGCCATACTTAAAACATTTGAGAGTGTT
TGGTGCAACTGTTTATGTGCATATTAAAAACAAACAAGGAAAGTTTGATGATA
AATCATTTAAAAGTATTTTTGTGGGCTATGAACCCAATGGTTTTAAGTTGTGG

GATGCTGTAAATGAAAAATTTATTGTCGCAAGAGATGTTGTTGTCGATGAAAC
CAATATGGTTAATTCTAGAGCTGTTAAATTTGAAACAGTGTTCCTGAAAGATA
GTAAGGAAAGTGAAAATAAAAATTTTCCGAATGACAGTAGGAAAATAATACA
AACAGAATTCCCGAATGAGAGTAAGGAATGCGACAACATACAATTCCTGAAA
GATAGTAAGGAAAGTGAAAATAAAAATTTTCCGAATGACAGTAGGAAAATAA
TACAAACAGAATTCCCGAATGAGAGTAAGGAATGCGACAACATACAATTCCT
GAAAGATAGTAAGGAAAGTAATAAATATTTTCTGAATGAGAGTAAGAAAAGA
AAGCGAGATGATCACCTGAATGAAAGTAAGGGATCAGGCAACCCGAATGAG
AGTAGGGAAAGTGAAACAGCAGAGCACTTAAAAGAAATTGGAATTGATAAT
CCAACTAAAAATGATGGCATAGAAATTATTAATAGAAGAAGTGAGAGATTAA
AGACTAAGCCTCAGATATCCTATAATGAAGAGGATAATAGTCTAAATAAAGTT
GTTCTAAATGCTCACACTATATTTAACGATGTCCCAAATTCATTTGATGAAATT
CAATATAGGGATGATAAATCTTCTTGGGAAGAAGCCATCAATACAGAGTTAAA
TGCTCATAAAATTAATAATACTTGGACAATTACAAAAAGGCCTGAAAACAAA
AATATTGTAGATAGCAGATGGGTATTTTCTGTTAAATATAATGAACTTGGAAAT
CCAATTAGATACAAAGCTAGATTGGTTGCACGAGGATTCACTCAAAAATACC
AAATAGACTATGAAGAGACATTTGCTCCTGTAGCTAGAATTTCAAGTTTCCGA
TTTATATTGTCATTAGTAATACAGTATAACTTGAAAGTCCATCAAATGGATGTA
AAAACAGCTTTCTTAAATGGCACGTTAAAAGAGGAAATTTATATGAGACTTCC
TCAAGGTATATCGTGTAATAGTGACAATGTGTGTAAATTGAATAAGGCAATTT
ACGGACTCAAGCAAGCGGCTAGATGCTGGTTTGAAGTATTTGAGCAAGCATT
GAAAGAGTGTGAGTTTGTAAACTCTTCAGTTGATCGCTGTATATATATTTTAGA
CAAAGGTAACATCAATGAAAACATATATGTATTATTATATGTAGATGATGTGGT
TATAGCTACAGGAGATATGACAAGAATGAATAACTTCAAAAGGTATTTAATGG
AAAAGTTTAGGATGACTGACCTAAATGAAATAAAACATTTTATTGGAATTAGG
ATAGAGATGCAGGAAGATAAAATCTATTTAAGCCAATCTGCATATGTTAAAAA
AATTTTAAGTAAATTTAACATGGAAAATTGTAATGCAGTTAGTACTCCTTTACC
TAGTAAAATAAATTATGAATTACTTAATTCAGATGAAGACTGCAATACCCCATG
CCGTAGCCTCATAGGATGTTTAATGTACATAATGCTTTGTACACGCCCAGATTT
AACTACTGCAGTAAATATCTTGAGCAGATATAGTAGCAAAAATAACTCCGAAT
TATGGCAGAACTTAAAAAGAGTTCTTAGATATTTGAAGGGCACTATCGATATG
AAATTGATTTTTAAAAAGAACTTGGCATTTGAAAATAAAATTATTGGTTATGT
GGATTCTGATTGGGCTGGTAGTGAAATTGATAGAAAAAGTACAACAGGGTAT
TTATTCAAAATGTTTGATTTTAATCTCATTTGTTGGAATACAAAGAGACAGAA
CTCAGTAGCAGCCTCATCAACTGAAGCTGAGTATATGGCCCTATTTGAAGCCG
TGAGAGAAGCTCTATGGCTTAAATTTTTATTAACTAGTATTAACATTAAACTAG
AAAACCCCATTAAAATTTACGAAGACAATCAAGGCTGTATTAGCATAGCAAA
CAACCCCTCATGTCATAAACGAGCTAAACATATTGATATTAAATATCATTTTGC
CAGAGAGCAAGTTCAGAATAATGTGATTTGTCTTGAGTATATTCCTACAGAGA
ATCAACTGGCTGACATATTTACAAAACCGTTGCCTGCTGCGAGATTTGTGGAG
TTACGAGACAAATTGGGTTTGCTGCAAGACGACCAATCGAATGCTGAATGAA
ATTTTTATATATATTTTTCAAATTTAAATTCCTGTAAACATATTTTGTTACAATGA
TCTGATCGGGTTTTTCTGGGTTTTCCCCGTATCCTCGCAGCAAATGCTGGATC

AGTTAACACTTCCCAGAATGCACACCACCCACATTTGATAGTTACTAATGAAT
ATTATTGTTATGTTTTTAATTATAGACGTTATTTTTGAGGGGGCGT

>COPIA2LTR_Copia#LTR/COPIA2LTR_Copia

TGTTGAAAATATGTATGTAATATGTATGTATATATAATAATATGTAATAAAGTCA
ATGCACTGTGTCTCCCTCTTTTGGTCGCGGTAACCAAAAGCTTTTTTCTCTTAT
TGTGTTATCCTCTTTAGCGTGTAATTTGGCTGCCTGCGTGCAGTAACATTGTAC
TCTAGATCAGTCACAATAAATACTTGAAACGAATA

>COPIA2I_Copia#LTR/COPIA2I_Copia

GGTTATGGGCCCAGCCCACGCGGACATAAAAGTGAATAGTTCTAAACGAATT
AGAATATTCGGCATCTTTAAATATTGTGATTCAGGGATTTAATACGAAAAACTT
ATCGAAGTGAAAATGAGTTCATCAATAAACCAAATAGAAAAACTTGATGATG
AAAACTACAGTGCGTGGGCTGTACAAATGAAGAGTGTCTTAATTCATGCGGA
ATTATGGGGGGTGGTGTGTGGACGTGTGGTTAAAAACGAAAGTGATAGTGCT
GAGCTAAAGGCTTTGTTCGACGCGAAGGACGAAAAAGCATTAGCAAGTATA
ATGCTATGCATTAAGACATCCCAAATTAACCATATAAAAAACTGTGAGACTGC
TGTAGAGGCATGGCAAAGACTTAGTGAAATCCACACTCCCTCAGGACCGGCA
CGACGTATATGTTCGCTAAAGCAGTTGTTGCACATGAGAATGTCTGAAACAG
AAGTTGTTTCGAGTCATGTGAACAATTTTTGTGCGGTTGTTGAAAAACTAAA
AGAAATTCAATTGGTGATCCAAGAAGAAGTCCTGAGTATTCTCCTGTTGTCCA
GTTTGCCGGAATCGTTTGAAAGTTTCGTAGTTGCAATAGAAACGCGCGATGA
GTTGCCCACATTGAAAATGTTAAAAATAAAATTACAAGAGGAAGGGCAAAGG
CGTATGGCAAATGAAGAACATTCTGCAAAAAGCGAACAAAGTGCATTTGGA
ATTCGGTCTGCGAAACAAAAAGCGAAAAGTGACATTAAGAAGAGTGTGCAA
GTAAATAATCAGCAACTGATTAACGGGAAAAGAACAGTTAATTGTTGGAACT
GTGGTCGCAGCGGCCACAAAGCTGTAAACTGTATGTATAGGCAAAGGAAAG
GAGAACATAGAGAGAGCGAAAACTTAAATACCGAGAAATCATTCTCTGTGCT
CTGTTCAACTGTACAGCTTGGCGAATTGCCAAAAAATATGTGGTGCCTTGATT
CAGGAGCAACGGCTCATTTGTGCGGTGAGAGATCGATGTTTAAAAGCTTCAG
AGAGCATAAAGAGCGCATTCTTTTGGCTGGCAATAAGTATATTATGGCTGACG
GTCGTGGTACAGTGAAAATAGCCTGGCGCAACTCATCATTTGAATTGATCGAC
GTACTCTTTGTAAAGAATTTACAATGCAACTTTATGTCGGTGTCGAAGGCAAT
TGAAAACGGATTTCGTGTGTCGTTCGAAAACAGGCGCGGAATTTTAAAAAAT
GATAAGAATAAAGTTGTTTTGATTGCTGAACTGCAAAGTGATTTGTTTATGTT
CCAAAATGAAAGAAAAGTTGAAAGTGCATGTTTCGTTGCACAGACAAATTTA
ATGAGAAAGTGGCACCAGAGATTCGGTCATTTGAACTTTGCAAGCTTGAACA
AAATGATCAAACAAGAAATGGTACTCGGACTAAAGAAAAAATATTTAAGTGG
CGCAAAAGAAAATGACTGTTTGAGCTGCGCAAAAAGCAAGATATGTGTGAA
GAGTTTCCCAAAAGCCTCTGGAAATCGCACAAATGAAGTACTGGAGCTAGTG
CATAGTGACATCTGTGGTCCAATGCAAACGACGTCTGTTGGAGGTGCACGCT
ATTTTGTCACCTTCGTAGATGACAAATCGAGATACATGTTTGTGTATTTCATAA
AGACGAGAGATGAAATACTGGCCAAATTCAAGGAGTTTAAAGCATTTGCCGA

AAACCAAAGCGGCAAGAAGTTGAAAGCAATAAGGAGCGACAACGGGCGTG
AGTATTTAAGTAAAGCGTTTCAATCTATCTTGACAGAAAACGGCATAAAGAG
ACAATTGACGGTACCGCACACCCCACAACAAAACGGTGTAGCGGAGCGCGC
TAACCGAACCCTGGTCGAGATGGCAAGGACTATGATGATTCACGCAGGCGTC
GGCGACTCACTATGGGCTGAAGCTATCAATACTGCTGCTTATCTACGCAATCG
CGCTGAGACGTCAGCATTATCTGATGTAACACCGTTCGAGTCTTGGACTGGA
AGAAAGCCATATGTGTCCCACCTGAAGATATTTGGTTCGAAGGCTATCGTGCT
TAACAAATCCACAAAAAGGAAGTTTTCTGCGAAGGGCGAAGAAAATATTTTG
GTTGGATACTCTGATGCTTCTAAGGGATATCGCCTGTTCAATCCTATCAGGAA
GAATATCTGTGTTGCTCGTGACGTAATTGTATTTGAGAACGACCAGGATGATG
GTATGACGGCAGGGCAAGCAGTACCTTCTCCCGATGTTCAACTTGTGGAGAT
GCAGCACCTTCCAGTCTGTGTTGCTGAGGGTAAGAAACAAGATGATGTTTTC
CAGGTGTCAGATAAACACGTTGACGACGTTGCGTCTGTCGAGCAACACCGAT
TCAGAGGGAGAAGTCGTCATCCACTGAAGACAATGAAGCCACCGAAGTCAG
AGGACCTGGACGGCCAAAGAAGATTTATACTGGTAAACCTGGAAGACTACG
AAAACAATACAACATGGTCAACGGCATGAGTACGGATGAGGTTCCAATTCCA
ACAAGGCGTCAAGGAGGCGTTAACCATAAGAAACTTTGAAGAATGGAGAAC
CTCAATGCAGAAGGAGTTGGAGAGCCTACGTGCAAACAAAACCTGGTCATT
GGTGGATTTGCCGGCAGGTGAGAAGGCCATTGGGTCAAAATGGGTCTTTGCA
GTGAAGCGTAACAAGATGGGCAAAGTCGAGAGGTTTAAGTCTCGACTTGTC
GCAAAAGGGTGCGCTCAGCGATACGGCGTTAACTTCACGGATACGTTCTCAC
CAGTGGCACGCTACTCATCAATCAAATTAGTAATTGCTTTGGCGGTGGAAAAC
GGTCTCTACATGCACCAAATGGACGTCTCGTCTGCATACTTGAATGGTGATCT
TCACGAAACGGTATATATGAGGCAGCCAGAGGGATTCATCGATGAACGATATC
CAAAGAAGGTGCTGAAGTTACACAAATCTATATATGGACTGAAGCAGAGCGG
CCGAGAATGGAATAAACTACTGAACGAAGTACTGCAAAAGATTGGGTTTTCT
TCATGCCCTAGCGAGCCATGTGTTTACACCCGTAATTCTGGCAAAAGTAAGA
ACCTTGTTGTAGTGTACGTAGACGACCTTATCATAGCCAGTTCAAGCAAGGA
GGAACTGTGCGATATTAAAGCATCAATTTCAAAAGAATTCGATGTCGTAGACG
GGGGCGAGCTAAGACATTTCCTGGGTATCGAAATTGAACGTGACGGAGAAA
CTGGTGGAATCAGGATTGGCCACAAGCAGTATATTGAGAGCCTTCTCAATGAT
TATTCGATGCAGGATTGTAAGCCGAATCTCATCCCACTGGAAGCAGGCTTTGA
AGTAAAATGTGACAAGGCCGATTGTCAGAAGGTGAATCAAGTCAGCTATCAA
TCGCTGATTGGCTCATTAATGTATCTGGCAATCACAACTCGTCCTGATATTATG
CATTCGGTCATTAAACTATCACAACGGAACTCTGACCCACATAAAGAGCACG
AAGCCGGTGCAAAGCGAGTTCTGCGATACTTGAGGGGCACAGCTGATTTGCA
GTTACACTACGAGCGTACTGGTGTGCCTATACATTGCTATGTTGACGCGGATT
GGGCTGGTGACACCACGGACCGAAAATCCTTTACTGGATGGGCATGTATTGC
TGCAGGAGCTGCCTTTACGTGGGACTCAAAGAAACAATCAGTGGTTTCCCTA
AGCAGCACAGAGTCAGAGTATGTGGCACTTTCCATGGCAGCGAAGGAAGTG
GCGTATGTTCGGAAATTGGTCAACGAAATGGGTTTTGGTGAAACACAGGCAA
CTAAGGTTTATAGCGACAACCAGAGTTCCCAGTGCTTGGTGAAAAATGACAC
TTTCCATGCACGTAGTAAGCATATAGATATAAAATATCATTATATAAGAGAACT

GTATAAGAACAATATAATAGAAGTTAATTATGTACCCACAGAAAACATGATGG
CAGATGTTCTAACCAAGAATTTGAATAGATTTAAACATGAAAAGTGTATCCAA
GGGATGGGTTTAAACTAACAAAAATGTATTTTGATTTTGGAATTAAGATAAAA
ATATTATCCGTAAGGGTTGACATATTGCATTGAGAGGGAG

>Copia1-LTR_Copia#LTR/Copia1-LTR_Copia

TGTTGAAATTACGTTATAATTTAAATAAGCTTTATATGTTTTGATCTGGCAACG
CTCAATTATAGCTTAAGAAGTCATTGTGAAATTCCCATTACTCAGTTTCAATTA
AACCGTCAACTACACCACTACGTTCCTGTGTTTATTTGCTCGGTCGGTCTAGA
AGCACATAACGGTTGTCCTGATCGTACGGTAAATCACTAACA

>Copia1-I_Copia#LTR/Copia1-I_Copia

GTTGGTTATGGGCCTTCAGCAAGCTAAGTTTTCGGTTTGAATAATTCAAAGTA
ATTAAATGGACTAACACGCGTCCGGTTGAAACTCGAAATAAATAAAAAAACT
AATTTGCGGAATTCGACAAAATGAGTGTGTCAATTCAAATAGAAAAATTAGG
CGACGACAATTACGACGTATGGAGCATTTTAATGCGTAGTGTGCTTATAACAG
CAGATCTATGGAAAGTCGTTTGTGGTCAATATGTAAAGCCAGAAGATGGCTCT
GAAGATTCCGAAAGATGGAATATGCTCGATCAAAAGGCCCTGGCAAGTTTGT
TTTTAAATTTAAAAGAAACGCAGTTAATGCATATAAAGGCATGCACAACATCA
GCAGACGCATGGAAAAAATTGTGTGATGTACATTTGCCAGGTGGCCCGATCA
GAAAAGTGCAGTTATATCAAAAACTATCAAGGCTGAGAATGCTTGAAGGGGA
TAACGTTGTTCAATATGTCAATAAATTTGCTGAAACAGTCAACAAATTAGCTG
AGATGGACATAACAATTAATGATGAATTAAAAAGCATCATGTTGTTATCCAGT
TTACCAAATTCGTGGGAAAATTTCGTTGTAGCGATCGAAACACGCGACACGC
TGCCGACGTTCGAAACGGTAAAAGTGAAGCTTCTGGAAGAAGGAGCGCGCA
AGCAAGAGAGGGACGATCGCGAAGGTTCTGTCCAAGCCGTGTATGTACATAC
AAAATTACATGGAACTGCAAAGCAAAAACAACGCGAAGAAAATAACCTTGA
AAAACATAGTTTCAAGGGCAAATGCTACATTTGCGAGAAATTCGGTCACCGA
GCACGAGAATGCCCGAACAAAGGGAGGCATAAAAAACTAGGGGAAAGCAA
GGAGAAATCAAACTGCTTGATGCATATTAGTGCTACCACTCAAAGGAAAAAC
ATATGGTGTGTTGATAGTGGTGCCACGTCGCATATGTGTTGTGACAAGGGATT
GTTTACATCTTTCATTAATAAGGAAACATCGATTATGCTAGCAGCTGATAAGTT
CGTGAAATCGAGCGGCATCGGGACTGTTATGCTTAAATCACAAAATGTAAAC
ATTGAATTACGAGATGTAATTTACGTTCCATCATTGCATATGAATTTTTTATCAG
TGAGCAAGTCCGCGGAGTACGAAAACATAACTACTTTTGATAAAAAGGCGGC
GGTGATAAAAAACAAACAAGGTGAAGTGATGATGAGAGCAATGCAAGAAGA
CAATTTGTATTTATTTACAAGTAGCTCTAAAAACGGTGCGGTTCATTTGTTGAA
TGATTCTTCTCGCATGGCAACATGGCACAATCGGTTTGGTCATTTAAATTTTCA
ATGTTTAAAAGAAATCAAAGAAAAAGAGCTAGTAATTGGCATGGACTTTAAA
AATATGTCGGTGAATATTAATTGTGACACATGCAATATGGCAAAAATACACGT
ATTGCCATTCCCACAGAATAGCGAGAGAGCTACGCAAAGTGTACTGGAACTA
GTTCACAGTGACGTGTGCGGGCCAATGAATGTTAGTTCTTTAGGCGGCAACA
AGTATTTCGTCACGTTCATAGACGACTATTCAAGGAAAATATTTATATATTTTAT

GCATGCGAAGAACGAAGTGTTTGATAAATTCAAATTGTTCAAATCATATGTCG
AGTGCCAAACGGGCAAGAAAATAAAAGCTTTGCGAAGTGACAACGGGACA
GAATATGTAAACCGGCAGTTTACAGAGTATTTGAACACCTGCGGCATCAAAA
GGCAATTAACGGTTCCGTATACACCACAACAAAACGGCGTCGCGGAACGCGC
TAATCGAACGATCGTCGAGATGGCCAAAAGTATGCTGATTCATGCGAAATTAG
AGGAGTTTTTGTGGGCCGAGGCCGTGTCCACAGCTTCATATTTGCGTAACAG
GTGCCCTTCCAAAGCACTCATGGGCGCTACACCCTTCGAAATATGGCAAAAT
AGGAAGCCATCCGTTAAACATTTGCGAGTATTCGGATCTCGCGCGTTTGCACT
GGATAAGACAAGAAAAAGCAAGTTCCAGGCAAAAGGAAAAGAGTATATATT
TGTAGGATACTCTTCAACAGCTAAAGCTTACCGTTTATACGATCGGGAAAAAC
GTATCATTGTTGCACGTCGTGATGTCAAATTTGTAGAGGGTGAGTTTGAATCT
AAAAAATGCACTATTTCGGAACAAAATAATGACTTCGCTACAAACATCATTCA
CCTTGAGTCAAACATCCAAGTACCAGAGCAGCAGCGGCCGGCTATGCCCATG
GTGGTCGAGCAGAGCAGTAATTCTTACGACAGCTGTGACTCCGGTGAAGAG
GAGGAGGAGTTTGTAAGCGCAAGCGATGAAAAGCAAATTCTCACTGAGGAG
GAGATCGTACCAGACGTACAAGAGCAAGAAAGCAACGCACCTCGACGTGGA
CCCGGCAGACCAAAAATTATTCGCAGTGGTAAGTCCGGTAGACCCAGAAAAC
AATACAACAGCCTCAATTACATTGAAGACATTGAGACGCCACAGTGCGTTGG
AGACGCTTTACGGGGCGAGCATGCCCAAGACTGGAAGACGTCAATGCAGAA
AGAATATGATGCTCTCGTTTCCAATAACACATGGACATTGTGTGATTTACCTCC
AGGACAGAAGGCCATAGGATCTAAGTGGGTCTTTCGCGTCAAAAGGGATAAA
GAGGGAAATATACAAAAGTTTAAATCAAGACTTGTTGCTCAAGGTTGTGGGC
AAAAGATGGGCGTCAACTATTCGGAAACGTTTTCTCCAGTAATCCGCTACGA
AACCATCAGAATGCTATTCGCTATAGCAGCAGAAAAACAGCTATGCATGCACC
AGGTTGACATATCAAACGCATATTTAAACGGCAGACTTCAAGAAGAAGTTTAT
ATGAGGCAACCACAAAACTTCATTGATGAGAAGCATCCAAACAAAGTATTAA
AGCTTCAAAAGGCGATCTACGGACTTAAACAGTCTGGGCGTGTCTGGAATGA
CACATTGGACGAAGTTTTAAAGAGCATTGGTTTTAAGCGCAGCAAAAATGAG
GCGTGCCTGTACGCAAAGCAGCAGCAGCAACAACACAGCTACATTGCAGTAT
ATGTCGACGATCTAATCATCATCAGCTACGACGAGAACGAGATCAGCGCGATT
AAGAGGAAGATCGCAAATAAATTCGACATACATGATGGCGGCCAACTAAATT
ATTTCCTTGGCATGGAAATCCAACGAGAAAGTACAAGAGGGTCGATTTCGCT
ATGCCAGAAGCAGTTCATCATAAACCTATTGGATAAATATGGCATGCAAAATT
GTCGTCCCGCAGCTACGCCGCTAGACCCTGGTTATAAGATGGGTTGTAGAAAT
GAGAATTGTGTCAAGGTAAATATAACACAATTTCAATCATTAATCGGTTCCCTT
ATGTATTTAGCAGTCTTGAGCCGACCAGACATTTTGCATACAGTGAGCAAACT
GTCGCAAAGAAATACTGATCCACACAGTGAAGACGAGACTGCAGCGAAACA
TGTTTTGAGATACCTTTCAGCCACGGTTGATCTCAAAATCACATACAGCAAGT
CCGGCGAACAGGTAATGGGCTTCGCCGACGCAGACTGGGCAAACGACCTGT
CAGATCGCAAATCATACAGTGGGTATGCATTCTTCCTTGGAGGCAGTGCGTTT
TCATGGACTTCAGCTAAGCAGAGCGTCGTCGCCATGAGCAGCACAGAAGCA
GAGTACGTTGCTTTATCCACGGCTGCAAAAGAAGCGGTGTATCTTCGCCGTTT
GCTTTTAGAGATTGGATGGTCATTAGATGGACCAATAACCATATGTGGCGACA

ACATAAGTTCTCATCATATTGCGAAGAATCCAGTACACCATAAACGCACTAAG
CACATAGATATTAAGTATCATTTTGTTCGTGAAAAAGTAGAATGTAATGAAATA
ATTCTTGAATATGTACCCACTGATAAAAATGTTGCAGATGTACTAACGAAAGG
CTTGTGTAAGCAAAAGCAACAGAATTTTACTAAGTTGCTTGGATTAAATTAAT
TTTCGTTTTACATCACATATATATAGCTTAAGAGAAAG

>Chouto_LTRGypsy#LTR/Chouto_LTRGypsy

AGTTAACAAGTAACATCAGGAAAAGCTGCGTGTGACGACGAACGACACACG
AAGCTATACTTCGGATGCTGGTCGGCGGCCGTGATCTGTGCGAGATAGGGTA
ATCTTAAGCTTTCTTTCTTTGTATTACTTAGTTCTGAACTGACAGCCGAAATAA
AGTGATGTTATCGTAAGATACCTGCCGTGAATATCAAGTCGGAGCTACGACCC
TGCTACCGGCTTATTCCAGTTGGTCACTTCAGCTATAGTCACCGATCGTGGAC
ACCACCTGATCCCGAGGTGATCCGCACTCTGCACCACACCGCCGCTGCAGAG
GCGACAACGAGGTGACTGTGTTGTGAGCAGCAACGTGTCGCAACACACATT
CAGCAACCACTTGGTCACTTTAGCTATAGTCACCGATCGTGGCCACTACCTGG
TCCTGATCCACACTCTGTACCACACCACAGCTACAGAGGCGACAACGAGGTG
ACTGTGTTGTGGGCAGCAACTCGCCGCAACACACTTCAGCTACCACGTGAGC
AGCAACGTGCCACGTTGACATCCCCTTGCAACAACGGTGCAGGCGGAGGAG
TTCGCGGGCAACTACCTTGCCGCGTCCATCTTTAAATCATATCT

>Chouto_IGypsy#LTR/Chouto_IGypsy

GGCGCCCAACCAAGTCGACGACCCCGACAAGATCACATAATCTGGAAGGCA
CGAGCTAAATAAAACCCATTTGAGTTGGCAATCTAGGACGAACTGTCCTGGG
CAGAGGAGAAGGAGACAAACGCAACAACAAAACCCGAAAAGGAAGAAGG
AAAACTATCATAGATTCATGTGAGTGAAACCCCTTTTTATGAATTGCCAAAAG
AAATTAAAAGGAAATGTAACAAAATAATGCAATTAAATGTTAAATCTCAGTTA
AGACATTTATTTTGGAAAGAGTGTTGGCACTAATTTAAAGTAATTGCAAATTG
AGTAGTTTCTACTTTGCATAGAAGGTACAGTTTTCAGCGGACGACTAGTTGCA
TGCTTATCTCTCGGTCCCGGTGTTTTTTTTCCTAATAGTAAAATCTGAGACACG
TGCAAGAATACGGTGAATAGATAAGCGCTTAGCTTAGCGCCTAGCTTGGCGA
GCGATCAATTTTTTCTTACCTTTCCCTTGTATTCTGCACTTGTTTGCATTATCTG
TGCGGGACTGCCAAATACCTTGACGGCGGTTGTATGAGCGAGACAGAGTCTG
AAGAGTTGGTTGATCAACTGAGTAAACTCGCTTTAACCGACGAGGCCGAAC
GTATAAGAAAGAAGACATACAATATGGATCCACAACAACTACAGGCAGTCAT
AAATGTAGCGGTATCGGCTGCATTAAATGTGCAGAGAGAAGATTTTGAAAAG
AAGTTGGAGGAAGTCAAAAATAAGTTTACCGCTTCAACAATATCTGCTCCTG
AGATTGCAGTGTACGTGCCTATTGCTGTAACCGGCAATGTGAGTTGTGATGAA
GGCCTCGATGCGGTAAAATCATTGCCAGAGTTTGATGGGTCTCAGGGAAAAT
ATGTTTCTTGGCGACAAGCAGCAGCAGCTGCATATGAATTATTCAGAGAGTAT
AGCGGTAGTTCTAGGCATTACCAGGCTGTTCTCATAATCCGTAACAAAATTAG
GGGATCTGCTGACTCTGTTCTTTCTTCATTTAATACAGTCCTTAATTTTGACGC
CATTATTAGCCGCCTTGACTTTACCTATTCCGATAAACGCCCATTACACTTGCT
CGAGCAAGAGCTTACAACACTCAGGCAGGGGCAGATGTCTATTACACAGTTT

TATGAGGAGGTTGAGAAGAAACTCTCACTCCTTACAAACAAGACAACTATGA
CATATGAGACGGACCATGCCCGATATATGAATGATAAGCATAGGTCTGATGCC
CTGCGTGTCTTTATTTCTGGGCTAAAGAGAAACTTAACTGATGTTCTATTTGCT
GCTCAACCTGCAGACCTGCCTTCAGCCCTAGCAATGGCTCAGGAAATAGAGG
CAAATCACGAGAGGCATGCCTTTGCGACACAATATGCCAGGTCCATAGAAGA
AAAATATCTCAGGCAGGCTCAAAATCGTCAATTACAAAGGACAACAGAATCC
AACCATGAACCGACGTTTAAAAAGTGGGTAAAAAATCCATTTTTTACAAGGG
ACAATGGTGGACGACCCGTTGTTGACAATAGACCGTTAGAGAAGATGGACGT
AGATCCAACATTGTCGAGGTTTCGTCAGCTGACAAACTTTCAGCGCCCTCAG
GGTTTTTCCATGCCACAGGCGCTTGACGCACAACAGCAGAGTGCACAAAATA
GACCGACTAGCTCTCAAAGAATGTCTGATTTCAAGCGTCAAAGGGTTAACCA
TCTGTTTGAAGAAGATAGGAATGATCAAAAGGAGGATCATTATAATCAGATTG
CTAACGATGCGGTGGATGACATAGACGGTGAAGAGGAAACAGACTATGAAA
GCGATTCCGTACATTTTTTAGGGGTTCATCCCTGCTTCCGTTCGTCCAGCGAA
CAGTAGCGGGGGAGCAACGAAAATTTCTAGTGGATACGGGAGCCTCTAAAA
GCTACATCAAGAAAGAGATAGGATTGCCGACTTCAGTTCCCGTCGACTCACC
TTTTATTGTTAAGTCACTTCATGGCTTTGAAAAAATAGAACGCAAATGTGAGC
TTGATCTGTTTGGTGTAGAGAGCCATTTCTTCTTATTACCCGGACTTAAAACAT
TTGACGGMATAGTTGGCCTTGACCTTTTAGCTCAGGTAGGTGCGACAATTGA
CCTCAAGGGCAATTCTGTCAAGTTTAATGGAGGACGTGAAGATCTACACTTT
CAAAAATGCGCTCAAGTTAACTTTACGAAGATTGAGGATATTGTCGTGCCCCC
AAAAGTTCAGGAAGATTTTCAGCAGATGATGTGTAAAAACTCGAGAGCCTTT
GATGATCCCAATGAGAAGTTACCATTCAACACAAATGTCGTAGCGACCATTCG
AACAGAGGACAATGAGCCAGTTTATTCAAAATTGTACCCGTTTCCTATGGGTG
CTTCCGATTTCGTGAATCGGGAGATTAAAGATCTACTCAGAAATGGCATTATT
CGGCCATCCAGGTCTCCCTACAATAACCCCATATGGGTTGTAGGTAAAAAAG
GCACTGACGCTGCGGGATCTCCCAATAAACGCTTAGTAGTCGATTTCAGGAA
GTTAAATTTAAAAACTATTGCCGATAGATACCCGATGCCGAGTATAAATATGAT
ACTGTCGAATCTCGGGAGATCAAAATTTTTCACCACTCTCGACCTTAAGTCCG
GATACCACCAGATTGAACTTGCAGAATTGGACCGAGAAAAGACATCATTTTC
CGTCAACGGAGGCAAATATGAGTTCTGCCGCCTACCGTTCGGGCTGAAGAAT
GCCTCAAGCATCTTTCAGAGGGCCATAGATGATGTACTACGGGATAAGATAGG
AAAAATCTGTTACGTTTATGTGGATGACGTTATTGTTTTTTCAGAAACTGAAG
AGTCCCACATTGTTCATATCGGACAGGTTTTAGAGAGCCTCCATAAGGCCAAT
ATGCGAGTGTCTAGGCAAAAGTCAAAATTTTTTAAAGAAAGTGTTGACTTCC
TTGGTTTTGTTGTCACAAAGGAGGGTATTAGGACAAGCCAGGACAAGGTCCA
GGCCATCAAGGATTTTAAACAGCCCAGCAATTTGTTTGAGCTGAGATCGTTCT
TGGGCTTGGCAAGTTATTATCGATGCTTTATAAAGGATTTCGCATCAATAGCTA
GACCTTTGACGAGTATTCTGAAAGGTGAAAGTGGCAGGGTCAGCAAATACC
GTTCAAAAGGCATTCCGGTCAGTTTTGATAAGTCTCAGCTTAGAGCATTTGAC
AAGCTTAGAAGTATCCTTGCATCAGAGGACGTTATTCTTCATTATCCTGATTTT
ACGCGACCATTTGATTTGACGACTGATGCGTCATCCTTTGGCATAGGTGCAGT
ACTGTCTCAGGGAAACAGGCCAATAACAATGATATCCCGAACGCTAAAAGAA

AGGGAATTAAATTATGCGACAAATGAAAGGGAGTTATTGGCAATCATTTGGGC
CTTGGACTGTCTCCGACACTACCTCTATGGCAAAAACGACATAAACATATTTA
CGGACCATCAGCCCCTTATATTTGCGGTCTCTGACAAGAACCCAAATTCAAA
AATTATGCGTTGGAAAGCCCGAATCGAAGAGAGCGGTGCAAAGGTCATGTAT
AAACCGGGGAAAGAAAATTGCGTTGCGGACGCTCTGTCGCGACAAAATATTA
ACGCTTTGCAGAGCTCATCAGAGTCAGATCGAGCCACTATTCACAGTGAAGA
ATCCTCAACATTTGTCATTGAGGCCACGGAAAAGCCATTGAACTGTTTCCAG
AATCAAATCGTATTGGAGGAAGCATCCGTGCCGAATAAGCGACTATTTATAGT
ATTTGGTACTAAGACACGTCATCAAATTGATTTTACTGATTGGGAGACATTAC
TGGACTTAGTAAAAGACGTAGTAACACCCAACGTAGTGAATGCCATACACTG
TGAGCTTCCAGTCCTGGCCCGTATTCAAGATAGTCTGGTTCGGCTCTTTCCTG
CGACCAAGTTTTGGTTTTGTAAGTCTTGGGTGGCGGACGTTATCAAGCCTGAT
GAGCAAAAAGAAATTTTAGTTATTGAACATAACAGAGCTCATAGGGCTGCTC
AAGAGAACGTKAAGCAAGTGCTACTTGACTACTATTTTCCAAAAATGGGTAA
AATTGCAGCCGAGATCGTTTTAAACTGCAGGGTTTGCCAAAAGGCAAAATAC
AACAGGCATCCAGTAAAACAGGAATTAGGTATAACCCCAATACCAACGCAGG
TTGGGGAGATGTTACATATAGACATCTTCGCAACAGGGGGAAAGTACTTCCTC
ACCGCTGTTGATAAATTTAGCAAGTTTGCGGTGGTTCAGCCAATTGCATCGCG
TTCAATATTGGATGTAAAGACCCCAATCCTTCAGCTGGTCAACCTTTTTCCTA
ATGTTAAATCGATATATTGCGATAACGAGGCATCGCTTAACTCCGAAACAATA
CGATCCATGCTAAAAAATCAATACGATATTGAAATAGAAAATGCCCCACCTCT
ACACAGTACGTCCAATGGTCAGGTGGAAAGGTTCCACAGTACTCTTATAGAG
ATAGCCAGGTGTCTTAAATTGGAAAGGAAAATAGAAGATTTTACTGAATTATT
CCTTCAGGCGACTATAGAGTATAACAGGTCATTTCACTCTGTCACGAATAGAA
AACCTGTCGAGATCATCCATGCTAATGCCGACATTGCCCCTGATATAATCATGA
GCCTAAAGAAGGCACAGCAGGAGCAGTTAGATCGGAATAATCCGACTAGGC
AGAATAGAGTATTTGAGAGGGGTGAGAAGGTGTTAGTTAAAACCAACAAGA
GACTGGGGGATAAACTTACGCCCCTGTATGTCGAGAATAGGGTCGATGCTGAT
ATAGGGACAACGGTCCTAATTCAAGGGAGGGTGGTCCACAAGGACAATATTC
GGTGAGGCCACTTACAAGATTGATTTATAGGCTAAAAAGTTGTGGCAGATACT
CTGGCGTGTTACCCAAAAATTCAGGCTCGTATAAATGCTCACGCAACCATCGC
AGTAAAACATTAAATCTAATTTTCTTTGTAAATTCAAATTTATTTAAATTAAAA
AAATACATATATTGAAAGTAATTTAATTGCCCTCACTCTTGCCAAAATAAATAT
TTTGCATTCTTTGAAGTTGCACTGTTGACCCAAAGGCGATTATAAAAAAGGTT
TCACAACTGTTAACCTAAAATACTAGGAAATACTATGTTTATTATTGAATCTTG
TACAATTAATTTTAGTTTGTATGGTAGATAATTTGAAAAGCGCACACCGACCG
GATATCACACCTCGATGAAAACAAATGAATGCAAGGTCGAACTAGAAGCGTT
GCTGTCAGCTCATGGAATGGGGTACAATTAACCGTTTGCAGCCTGTTGGTCG
GCTCATCTCATCGACCTAGCACGCATATCAAGGATCGGCCAACTACTCTTCGT
CAACTTGCACCTGCAGCCTTAATCCTGCCCTTTCTTCACTAGTCAAACGAAG
AAATGCTGCAGAATATAACTGGTTACAAATGCTTACAATCAGATGCCACCTTC
ACCAACATCAATCCGCTTTGCATTCTACGTAATGTTAAATATCATGATTTATCA

AGCGGCAAAGTGGAGCCTCGATGTCGACAACGCAAATCCTTGATAACATAGA
CATGGACGAGGACGCCCATTTTTAATGTGATGGGGAGT

>Chimpo_LTRGypsy#LTR/Chimpo_LTRGypsy

TGTGGCGCCCAGACTGAAGCCCGGTCTGGCATCACGGCGAAAACAGGTACT
AACGCCAAGACCTTTCCTGGAGGACGATATCTTCTGATATCGTCAGACTGGA
CACACTGGAACTTCAGCTCCGCCGCGCAACAAGCTTACGGCATTAAAACAAC
AAGATATTGTACTTAGTAATTAAATAAAGCGGTAACTACATAAGTTACCATA

>Chimpo_IGypsy#LTR/Chimpo_IGypsy

TTGGTGACCCCGCAATAAGCAAAATGCAGAACACCGGAGATTGTTTGCCTGC
ACCGCCCACTTCGCCTGACTCTGGTGAAATTCAACGCGGCAACAGCGCAGG
CACCGTTATTCCTGTCGTCGAGTCGGTTGCCACCATCAAGCTACCGCCATTTT
GGAAGCAAAATGTGCGGTTATGGTTTGTGCAGATCGAAGCGCAATTTCAATG
CAGCCGCATTTCTTCAGATAACACGCGCTACTACCATTTAATCAGTGCTTTGG
ATGCTGATATAATGACTGAAGTATCTGATGTTCTGGCGCAACCCTCGGAAGTA
AACAAATACGAGCACCTGAAGGAGATTATTATCAAGCGATTTACGGAATCGC
CGGACAGACAGTTGCAGCGCGCTTTACTGGAGCTTGACCTTGGGGACAAGA
AGCCCTCGCAGCTCCTTCGGCAAATGACAACACTTGCTGATGGTCGCGCGTC
TGCAGACGCCTTGCGAGTCCGCTGGCTCTCTCAGCTACCGCAGCACGTACAA
CGGGGCTTGAAGCTCCTACGCTCAGCAACTTTGGAAGAGCAAACTGCACTC
GCTGACGATCTCATGGAAGAGGAATCGGGTTCTTTTGTCATGGCTGCTGGGT
CCTCCTCGGCACCACAATTCAGAGGTAACACCCGCGAATACGCGCCGGCTGC
TGGCACAAGTGTCGTGGACGACTTGAAACGCGACATCGCTGCCATCAAGGC
CTCTTTAACTCTTCTGCAGAACATGCAGCCTCCCAGCCATTCTCGCTCAGCCG
ATCGCAACCAGCAGCAACTCCGAGCACCACGTGTCTGCTTTTATCATGCCAA
ATTCGGACCTAATGCACGAAAATGCTCCTCGCCCTGTGCCTGGGTGCCACAG
CAGGGAAACTAAAGAAGCTGTCACCTGTTCAGGCGACTGGTGACAGCAGAC
CTCCAAGCACTCGCCTCTCAAAGGAGTACCGACTTCACATAATCGATCGTAAT
ACCCACATAAAATTCCTTATCGACTCCGGATCAGTCATCTCACTTATACCTGTT
TCGATAATCAAGGGATTGCATCGTCGCAAAGACGACTTCAAACTCTACGCGG
CCAACACGTCTATAATTGATACCTACGGCACGCTAAATAATCTAACTTTTGGTC
TCAACTTACGGCGTAACTTCACCTGGTCGTTCGTCGTTGCGAACGTCCAATC
AGCAATCATCGGAGCAGATTTCCTCACGCACCACGGCCTCATCCTCGACCTC
AAGGGTAAACGCCTTATGGATCCCTCAAAAGCTGTTTCGACCAAAGGAGAA
GTTGCAGAAGCAAAACTCTACAACGTTTCTACAATCAACGTGCAAAGTTCCA
GTGGAATAGATCGCCCGTACTCCAATCTTCTTAGTCGCTTTGTGCAGATCACA
CGTCCTAATGTGCCTTTAGCAACCCGCATTCATAACGAGGTTGCGCATTGCAT
TCAAACAACTGGCCCACCTGTTTTCGACAAGCCAAGACGGCTTACAGGCGA
AAAATTAAAAGCGGCACGCCGAGATTTCGATCTCTTACTCCAGCAAGGAGTG
ATTCGTCCCTCCAGCAGTCAATGGCCAGTCCTATTCATTTGGTCCCCAAGGG
GAGCAACGAATGGCGTACTACTGGGGATTATCGCAAACTTAATGCGTCCACTA
TTCCAGATCGTTATCCGATTCCCCATGGGGAAGACATTCTTCAACGTCTGCAT

GGATGCTCGGTTTTCTCCACAATCGATTTGGTGCGTGCCTACCACCAGATCCC
AGTCGCTCCAGATGATATTCCGAAAACAGCCGTCACTACACCGTTCGGCCTTT
TCGAGTTTGTCGGCATGCCACCCGGACTCCGCAATGCAGCGCAAACCTTCCA
GCGCCACATGGACAACCTGCTTCGTGGCATTCCATTCGTAGGATGCTATATGG
ACGACATCATCGTGTTCTCCACAACGCATGAGGAGCATTTGAAGCACCTCCA
GACAATCTTTGAGATTTTGCAGAGGAACCACCTCCAAATTAACCCTAAAAAG
TGCCAATTTGGTAAAAGTGAGGTTATTTTCTTAGGGTTCCAAGTCAACGCGA
GCGGATTCAAGCCCCCTGTTGAGCGCACGCAAGCAATCAACAATTTTCCCAA
GCCTCAAACTATCATGGAATTGCGCCGGTTTTTGGGAATGGTCAACTATTACC
GACACCTCATCCCAAACGCAGCTCAGGCTCAAATCCCGCTCACAGACTACCT
AAAAGGCGCAACCAAAAATGACAAGCGCGAAATAGCCTGGACCGGCCCTGC
GGAGAGTGCATTCCAGGCCTGCAAGCAAGGAATTTTACGTGCCACGTGCTCT
TCGTTCCTTTCCCCATCGGCTCACCTCGCCCTTAAAACGGACGCCTCCGATAC
AGCGATCGGTGCTGCTCTCGAACAACTAGTCGGCAACGTTTGGCGTCCCATC
GGGTTTTTCTCGCGGAAACTATCTCCGGCAGAGCAAGGCTACAGCACGTATG
ATCGCGAGCTCCTGGCTATTTTCGCTGGTATCATCCATTTCAAGCACATTCTAG
AGGGTCGTCCATTCATCATCCAAACCGACCATAAACCATTACAATACGCTTTC
AGGCAGAAAGCAAGCAAAGCATCGCCTCGACAACTCCGCCAATTGGATTTTA
TCTCCCAATTCTCAACGGAGATAGTGTACAATCCCGGGGAAAACAACGCAGT
GGCAGACGCCCTCTCACGCATCAACACCATAAGCATGCCGTCTAGCTTCAGT
GCTCAAGCGATATTCGAGGCCCAACAAACCGACGATGAAATGGCTGAGCTCT
CTCAAACCACGTCCCTGGGTTTACAAGTTAGGAACATCGACAACCTAGACAT
CTGGTGTGACATCTCGGGTGGACGCGAGCGGCCATATTTACCCAAAAGTCTG
AGACGCCAAGCTTTTGATGCAGTACATGGATTGGCGCATCCCAGCGGCCGTA
CAACTGCAAAATTACTTAAACAGCGGTTTGTCTGGCCAGCAATTAACCGTGA
TGCCACCCTCTGGTCTCGACAATGCGTTCCTTGCCAGCGGGCCAAAATCCAT
CGCCACACACGCAGCCCGGTGGAGAAGATCGATGTTCCTGACAATCGTTTCG
AGCACATTCACGTCGATCTGATTGAACTTCCCACAATCCGTAACTTACGCTAC
TGTCTTACCATCATCGACCGCTTCAGCAGATGGCCTGCAGCTATTCCACTATC
CAATATCACCGCGGAGACTGTCGCGACAGCTCTGTATACACAGTGGATCTGC
ATGTTTGGCTGCCCACTCACTATAACAACGGACCAGGGAACTCAATTTGAAT
CCTCATTGTTTGCTGAGCTGGCTAAACTAATTGGAGCCGAAAGGATTCGTACA
ACCGCATATCACCCTCAGTCAAATGGCATCGTGGAGAGATGGCACCGAACCC
TCAAGGCCGCCCTGATGTGTAAGTCTCACATAGCATGGCCGGATCTCCTGCCT
ACAGTTCTGTTGGGGCTGCGCTCGGCGTTTAAGGCGGACCTCCAGTCATCGC
CAGCCGAAATGCTTTTTGGCACCACTATTCGTATCCCCAACGAATTTTTTGAA
TCGCACACTCCAACCGCCTTCAGCAAGGATCACCGACTAACCGAGCAACTAC
GTCAACATATCAGGGCAGCCCGACCTACTCCTGCAGCGCATCATGCTAAACC
ACCTGTTTTCATTTGTAAGGACCTGAGAACTTGTACGCACGTCTTCAAGCGA
GTGGCATCTGTTAAGAAACCACTCGAGCCGCCTTACACGGGACCTCATCGGG
TAATACGCCGGGTTGATGACAAGTTCTACATCATCAGCATTCATGGACAAGAG
AAGGCCATATCAGTCGATCTCCTCAAGCCTGCCTTCTTAGCTGAAGCTGACGC
TTGCGAAGACCACCACGAGGATCCACCGCCTGACCAACAGATTTCGCAGCC

ACCTACACCGCCGAGCCCTGCACCTGCAGCTCCAGAATTTATACCGTTACCTT
CACCACCTGGCGAAGTCACTGGAGGGGGAGTAGA

>BURDOCK_LTRGypsy#LTR/BURDOCK_LTRGypsy

AGTTAACACAATCACAAAACACCCGAAATATAGTCGTAAGCCTCAAGTGCTT
TTCCCATCTATAGATCGAGCTTTACCTATAAGAAACTGTAACTTGTTAAGCTTT
AGAGATAAGAACTCTTGCTATACTTAAGTCAGTCGATTTTGGAAGATTAGAAG
CGTCGGTCATCGCCACGTACTTACTATTCGTCTCATTAAGTGCAGACCGCGCA
AGCCTATTGTAATTAATAAACTTACGCTAATAAATATATGGAAAATCTACTAAA
ATGATAATT

>BURDOCK_IGypsy#LTR/BURDOCK_IGypsy

GGCGCCCAAACGGATATAAAAACCTACGATAACTGAATAATTATAAATAAATA
ACAAAAGGAGGATCCGGAGACAAAACCAGCGGCTTTGGCTAATTAACTCTA
ACCTAAGAAATAAAAATTTGCTGATTACATAAAATATAATATTAATTACTAAGA
CCATCTACCTTAAAATTGTTTGTTAATCACTATTATTATATTGTAAGTATAACGC
TTATTGAACGAATTAAAAATATTATTATTATTATTATATTATAACCTATGCAAAGA
GTATTGATAATAAAAATACATGAGTGACAGTGATAACCTTTTAGACAACCTAG
TGTCAAGCTTAAATAAATGGTCAGCGCACCAGGCAAGTAGGCAAAACAGTG
CAGAAAAAAATAATAAGTCATCAGATAATTGGTGGTCAAAAACAAAGACAAC
TAGCGAAATGGAATTTGAAGCTCAGTTAAAAGCGATCGTAGAGAGTGCTGTT
GCCGGTGCGCTCGCAGTCCAAAAACAATCATTTGAAAAGCAATTGCAGGAG
ATGAATGAGCGAATCGGGAAATTAACAGTGAACACCCCAGAGGTGGAAACT
TATGTAGATGCTGAAATTAGACCAGGTGTTGTCTGTAGCGAGCCTCTAGATAT
ACTTAAATCTCTGCCAGATTTTGATGGCAAAAGTGAAACATATGTGTCGTGGA
GAAAAGCGGCTCATGTCGCTTTTAAAGTTTTCAAAGATTACGAGGGAAGTTC
AACATTTTACCAAGCTCTTGGTATTATGCGAAATAAAATAAAAGGTCCAGCGA
ATACAGTATTGGCTTCTTTTAATACTCCGTTACATTTCAAAGCAATGATCAGCC
GTCTTGATTTCACATATTCTGACAAAAGGCCGATCTATCTAATCGAACAAGAG
CTATCAACTTTGCGACAGGGAGACATGACTCTTACTGAATTCTACGATGAAGT
CGAGAAAAAACTGACCCTACTTACCAACAAGACAATAATGACATTTGATAGT
GCCTTGGCGATGTCACTGAATGAAAAGTACAGGACGGACGCGTTACGTGTAT
TTGTAACCGGAGCTAAGAAATCGTTGAGCGACATTCTTTTTGCAAAAGGTCC
AAAAGATTTACCAACTGCTCTCGCTTTAGCGCAAGAGGTCGAGTCGAACCAT
GAGCGTTACCAATTCGCCCTTATTTATTCTAAAAATATTGGAGACAGGGGTCA
GAAAATCGAACAAAGGCACAGCGATAAGGATAGAAACTCAATCATGCCCATG
CAAACTAAAAACCCATATTTTAGCAAGCGTCAGGTGCATACTTATGATAACCA
GGAAAGACAAGATCCAGTCCAGTTAACAAATCCTGATGTATCCATGCGATCTA
GAAGAACTGGAAATTTTGGACAAACTCCATTTCCGACTCAGGGAAATATTTG
GCCATCCCAACAGCAAAATTCTTGGCCATCTCAACAACAATATTCTTGGCCAT
CCCAACAACAAAATTCATTTCGAACACAAAATCAATTCGCATCGCAACCCCA
ACAGCAAAACACAAGTCAGGCTCAGGGACATTTTGGGTATGCGCAAGCATC
AAAAAGACCAACGAGTGGCAGTGCAAGGTTTACAGGGCCAAAACAGCAGA

GGATCAACTACTTACCTCATGAGAAAGGTCAATGTGAGGAAGATACAGACGG
TTATCAAAAGGAGGCAGAAGCGGAGGTTGATGATTATGAGGACGAACTAGTG
AATTACGATCATGTTCATTTTTTAGCCACAAATCCCTGCTACCGTACATAGAAA
GAGAGATAGCAGGGAGAACCATAAAACTTTTGATTGACACCGGGGCTTCGA
AAAATTACATACAGCCCCTCCCTGAATTAAAAAACATAATGCCGGTACAAAAT
AAATTCACGGTAAAATCGCTTCATGGTTGCAACACCGTCAAACAGAAATGCT
TTATTAAGCTATTTAACACATCTGTTCAATTCTTTATTCTTCCAAGTCTCTCTAG
TTTTGACGCAATAATAGGACTTGACCTTTTGAAACAGGGAAATGCAACGTTA
GATTTTAAGAACAAAACGTTGAATATCAACAATGAAGTGGAATCTATTCAGTT
TTTGAGATGTGACAGCGTAAATTTCGCCAACATAGAGAATATTGTGGTTCCAA
ATCAGATATCTAATAAATTCCATACAATGCTTCGAAACCGATTGGCCGTCTTTG
CGGAACCGGAAGAAGCACTGCCGTATAATACCAACATTGTTGCCACAATACG
TACTGAGGACGACCAACCCATTTACTCAAAACTCTATCCGTACCCCATGGGCG
TATCGGATTTTGTGAATAAGGAGACACATGCTTTGTTAAAGGACGGAATTATC
AGGCCCTCGTCGTCACCTTACAACAATCCGGTTTGGGTAGTCGATAAAAAAG
GTACAGATGAAGAGGGAAATACTAAGAAAAGGTTGGTTATAGATTTTAGAAA
ACTAAATTTAAAAACAATCGACGACAAGTACCCTATACCAAACGTAGTATGG
ATCTTGTCAAATTTGGGAAAAGCCAGATTCTTTACAACCCTTGACCTTAAATC
GGCGTTTCACCAAATTCTGCTCGCAGAAAAGGATAGAGCGAAAACTGCCTTT
TCAGTAGGAAATGGAAAATACGAGTTTTGCCGTTTGCCGTTTGGCTTGAAAA
ATGCCCCAAGTATTTTTCAACGTGCTATTGATGATGTTGTTAGGGACCGTATAG
GAAAGTCATGTTACGTTTACGTTGACGACGTAATAATATTTTCAAACGGAATT
GAGGACCACGTAAACGACGTTGCTTGGGTACTAGACAGACTGTCTGGGGCA
AACATGAGGGTTTCTAAAGAGAAATCGTTTTTCTTCAAGGAAAGCGTCGAGT
ATCTCGGATTCATGGTGTCAAGTGGAGGTATCACAACCAGTCCTAGCAAAGT
AGAGGCTATTCAGAAATATAATCAACCTACTAATCTGTTTAGTGTTCGATCGTT
TTTAGGGCTAGCAAGTTATTACCGCTGCTTTATTAAGGACTTCGCCTCTATTGC
TAGACCACTCACTGACATTCTGAAGGGTGAAAACGGAAAGGTTTCCGCAAG
CCAGTCTAAAAAGATACCAATTTCTTTCGATGAAAGACAATGTTCTGCTTTTG
AGAAGCTTAAAAATGTTCTTGTCTCCGAAAATGTAATGTTATTGTATCCCGATT
ATAGAAAAGCCTTTGACTTAACAACAGACGCTTCGGCTTTTGGCCTGGGGGC
AGTCTTATCACAGGATGGCAAGCCTGTTACAATGATTTCGAGAACTTTACAGG
ATAGAGAACTTAATTTCGCAACAAATGAACGAGAACTTTTGGCCATCGTTTG
GGCTTTAAAGTCTCTTAGGAACTATCTATATGGTGTCAAAAACTTAAACATTTT
TACAGATCACCAGCCGTTAACATACGCCGTGTCAGATAGGAATCCAAATGCA
AAAATCAAGAGATGGAAGGCGTTTATAGACGAACATAATGCTAAAATTTTCTA
TAAACCTGGCAAGGAGACCTATGTTGCCGATGCACTATCCAGGCAGGCTATTC
ATGTCCTAGAGGACGAACCCCAGTCAGACATTGCAACAATACATAGCGAAAT
TTCATTGACTTTTACAATCGAAACTATCGACAAGCCGGTTAACTGTTTTAGAA
ACCAAATTGTGATAGATGAGGGCACCGCAGACTCAACTCGAACTTTTGTTATT
TTCGGAAGCAAGACAAGGCATCTAATACAGTTTCTAGACAAAGAGACCTTAA
TCGGAAGAATTCGTGATGTGGTTAAGCCGGATGTAGTGAATGCGATACACTG
CGAATTACCTGTACTAGCTTTCATTCAAAACAGTCTTGTAAATGACTTTCCAG

CAACAACCTTCCGACACACTATGAAAATGGTCAGCGACATTTTAATCAAAC
TGAGCAACGGGAAATAGTGTCTTTGGAGCACAACAGAGCGCATAGGGCAGC
ACAGGAGAATGTAAAACAAATTCTTCAATACTACTTTTTCCCTAAAATGTCAC
AAATAGCCGCTACCTTTGTTTCTAACTGCTTGGTTTGTCAAAAAGCCAAATAC
GACCGCCATCCGCAAAAGCAAATCCTCGGGAGAACACCTATTCCGTCACATG
TAGGCGAGACATTGCATATTGATATATTTTCTACGGGCAGGAATTACTTTTTGA
CATGTATTGACAAATTTTCCAAATTCGCTATTGTGCAACCAATCGGCTCTCGA
ACGATAACTGATTTAGAACCTGCAATTATGCAACTAATGAACTTTTTTCCCCAT
TCAAAGACAATATTTTGTGACAATGAACCGTCCATAAATTCCGAGTCAATCAA
GTCACTTTTGAAAAATCGTTTTAATGTTGACATAGCGAACGCACCTCCACTTC
ATAGTACCTCAAACGGACAGGTTGAAAGGTTTCACAGCACGCTTTTAGAAAT
AGCTCGATGCCTGAAACTTGACAGTGGAATGAATGATACAGTCAACCTTATTC
TTCAGGCAACAATAGAATACAATAAGACGGTGCACTCAGTCACCAATAGAAG
ACCGATCGACATTATTCATTCAACTCCTCCCGAATTGGCTAACGAGATAGTAG
AAATGGTTAACGAAGCTCAGGAAAAACAGCTAAGAAGAGAAAATGTAACAA
GACGAGACAGAACCTTTGAGGTGGGAGAAACCGTCATGGTAAAACAAAACA
ATCGCTTGGGAAATAAACTAACCCCACGGTATAGGGAAGAACTAATCGAAGC
AGACCTCGGGACAACGGTCCTCATAAAAGGGAGGGTCGTTCATAAAGATAAT
CTACGCTAGGTTTAGTATTTCTTTTCCTTTTGTGACCATCGCCAAGTTAGCAAA
ATACAAACGTGAAATCTGAACACTAGTAAAAGAGTTTGCAAACATTTTTCAA
TTAAATATTTGTCAAATCCTTCTTATTTAATCTTTAAACATTTTGTATTATTTCCG
CTTCATCCTCTTTAGAAAATTTTAAAGGTATGTGATGAAATGCTAGACCCGAA
TGATTTGAAAACTTAAAGTCCACGCAACCACAAATATTTCCTGAAACTACCAT
AGAAAATAAATGCATTACCAAAACGGCATAATAACAGTATAGCGCACTCACTC
TAATTAGATTTCAAATTCCCGATTAAAAAAAAAATAAAACACTAATGTTATCA
ATACCCTTTCCTGATTCTGTTCAACTAAAATAGGAAAATCAATACTTGCAATC
AATAAGCGTTTTACTACATACTTTAATATCAAAATATCTGAATGAACTTTATTAT
AAAATTATAATTGTTATACTTAATTATTGTCAAAACTTTAGTATTAAAACTGTAA
CTACCTCTTAAGTAGATGAGAAGAGTAGAAGAGGGAATTAAGATCTATCAAC
GTAGTATCTGCTAAAGACGTAAAGATGCGGCAACTATTTCTGCGCCTGGGTAC
TGAAACGACGAACTGAATAATATCTGCCATCAGACGCCAACCAGAGTGCGTT
CAACACATACGTTTTGATGGTCAACTAGTTCAACCAACATCAGCATCATCGTC
GTCAACAAGTCGACGGTTACAATAAAGATTTTTTCCAAGTTCGCTACGATCAT
CTCCAGAACCTTGTTGCGAACCCATGACATGGAGAATCAGCAGCATTTACGA
ACTTCTCGGATCATCCAGACACGCAGAGCTGCCTTCCCTTCGATGGTTTAACG
CAGTACCAGGTTGGCAGTATGGGAACTTAGTGCACAACCAATGTTACCCGTA
AGATCCGCTTTCAAATAGATTTGCCAATTGTAAAAAGTCTGTGGACAGCCTTC
GTCTTAGAAGGGGAG

>BS4_Jockey#LINE/BS4_Jockey

TCATATCTGGATGGACGGAAGCTTATGGTCAGGTACACGGATACCTACTCCGC
TCCCTGCCAAATGTTAGCTGGAGTTCCGCAGGGCAGCGTCCTTGGCCCGCTG
CTCTATTCTTTATATACTGCTGACCTACCTAGACCTACCTACGAGAATGCGCAA

TACCCCTCTAAGGCTATCATTGCGACTTACGCCGATGATATTGCGGTTCTTTAC
AGATCCAAATGCCGTATTGAAGCTGCAAATGGGTTGCAAGGATACCTTCAAA
CTTTATCGGCGTGGAGTAGAAGGTGGAACATGAAGGTCAATCCTTTAAAAAC
ATTCAATCCATGTTTCACACTTAAAAGGCTTGCTACACCAGCAATACAATTTG
AAGGTGTGACATTGGAACAGCCATCTCAAGCGAAATACCTCGGCATAACCTT
AGATAAGCGCCTTACTTTTGGGCCACACATAAAAACAATAACCAAACGATGT
GGCCAAAGAATGCAGCACCTAAGATGGCTGATAAATAAAAGGAGCACAATGT
CGCTAAGAGCCAAAAGAGCAGTATACGTGGATTGTATAGCCCCAACTGACTT
AAAAGACTGGGATTCAGCAAGACGATTCGTTAATCGTCCAGCCCTCCCAAAA
CCTGCTAGTAATCCTCGTGAGAGGTTTGGTTCATTAAGATTTGTTTTTATATGT
GTTGTTAAGATGCTAATCAGAATGCATGGTTTCAACGCTTAATAAAATAATATA
TTAAAAAAAAAAAAAA

>BS3_Jockey#LINE/BS3_Jockey

CGTCTAGGGGTGCACTTCAGCGCTCKACGGATGCTAGCAGMAGCATTGAAG
CCATCCTGCAGACGCTWAATGAAAATATWAWTTCTTTGCGCTCGATTCAAGA
GAAGCAGATGGAATTAATGATGATGATGATGAAGAASAGCAACAACAGTCAC
AGCAGCAGGGGCAGATWATCAATCTGCTCACWGCTCTMCAAGCGCGTCAAK
CGCCATAATGATGCCGCTSCGCATCTTAGTGKGGAACGAAGSTGCCCGAAGT
GGAATTTTGTGCGGCGCTACGAGATCGATGTCCTGCTGCTCAGCGAGACTCA
TTGTAAGGAGGCACAGGCACCAAAAATATTTGGATTTGAGTCCTACACTGCC
AACGACCCGAGCGACGGTAACGCCAAAGGTGTGCAGCGATCCTGGTCAAAT
CCAGCCTAGTCCACTTCCCTCTTACACCAATAGCCACTGACAAGGTGCAACT
CGCGTCTGCAGGCATCGAGACGGCCATGGCCCTGTCAATTTTGGCGCGATATA
CTGCCCTCCGAGGTTCATATGGAATACGGGCGAATTTAAATGCATTCTGGATC
AACAATGTTCCAAASTCCTCGTCGCGGGCGACTGGAATGCATCCCACTGGCT
ATGGGGAGCGGGGAGATGCAATCGAAGAGGGCGAGCGCTAGCGAATCTGGT
CCTAAGCTCAGATATGAGCTCCCTAGCAACCGGAGGTCCTACAAGATTCCCG
TACGGCAGTAGAGGCTCCCCAGGCTATATAGATTTTGCTGTCAGCAGGGGCAT
ACTGGACATGCATGCTGAGATACGGTCCGTTGCTGAGCTCAGCTCCGACCAC
CTACCGCTTATAATCACACTAGATGCAACAGCTACAGCTTATCCCAAGATGGA
GCGACTAATCGGTAGGCACACTGACCTGGAACGATTCCATGAGCATCTTGAG
TCCTCTCTGAACCTGAGTACGGCGATAAACTCAGAAAGGGACCTAGAAGAA
GCTGTTGACACTCTCACCCATAATATCAAGGCTGCAGCCAGACTGGCAACTC
CTAGCCCGGTGCAGAGGCCCACAGCAGCTAAAATCCCGATCACCAGAGAGA
TTCTGCTCCTTATAGCTGAGAAGAGGCGTTTACGCAAAAGATGGATGAGGTC
TCGCCACCCGCTGGACAAAGCGGATTGGAACAGAGCAAAGTGTGTTTTACAT
CGCGCCTGGTCGAACATAAATCAGCTTGGTTCGATGACAGGCTTGCAAACAC
TGGTATCGAATGTGATGCGACGCACTCGCTATGGAATGCTACGCGCGCTATCA
AAAGGCGATGTGCGCGGAAAGCGCCATTGGCCGATAACAGTGGKGCATGGT
GCCGGACTTTTATAGAGCAAGCCGAAGTGTTTGCTGTTCACCTCGCGGAGCG
ATTCCAGCCTTTGAACCTCGCCAGTCCCCAGGATGTTGACGCGACTCACGAC
CAGCTGTCCCAAGCGCTCCAAATGGACTTGCCGATGCAACCGATCACTCCCA

GCGAGATTGCTGACGTCATCGCCAAACAAAACCCCAAAAAAGCTCCAGGGC
ATGATACTACCTGCAACTCCACCCTAAGGACACTACCGAGATGTGCGATCCTC
TACATTACGTTGTTATTCAACGCTATGGTGAGGCTGCAATACTTCCCTCCACA
GTGGAAGCTCGGTATTATCTCCATGATTCACAAACCTGGAAAGCCTGAAAAG
AACCCTGGGTCCTACCGGCCAATCAGTCTCCTCCCTTCGATCTCGAAGGTGTT
TGAGAGACTGATTGCTGCCCGGATGGTCAGGATTATGGAAGCGAAGGGTATC
CTGCCCGAGCATCAGTTTGGTTTTCGTGCTGGACACTGTACGGTAGAACAAC
TACACCGAGTGGTCGAGCAAATCCTATCGGCTTTCGAAAACAAGGAGTACTG
CAATGCACTTTTCCTGGATGTACGTGAGGCGTTCGATCGGGTGTGGCACTCC
GGTATCCTGCTCAAAATTAAGAATACGCTGCCTGCACCATACTTCGGCCTCCT
GAGGTCGTATCTCGAAAAGAGAAGATTTGCGGTACGATTCCACTCGGCTCTG
TCAAATGAGCATAATGTGGCAGCCGGAGTACCACAGGGAAGTGTACTTGGTC
CGCTGCTTTACTGCCTGTACAGCTACGACATGCCACGGCCAGACGTTAGCCT
ACCCGGGACGTCAATGTTGGCCACATTTGCTGATGACGTGTGTGTCACCTAC
AGGTCCTGCTGCGAACACGATGCTGCTGACGACATCCAGGACTTCGCATCAA
CATTTGCGGAATGGGCAAAACGTTGGAACATTGGCATCAATGGTGACAAATC
AGCGAATGTGTGCTACACGCTGAAAAGGAAAACACCACCGGCTGTGCTCAT
CGATGGCACCCCTGTCCCCCAGTCCAATTCAGCCAAATATCTTGGTGTAATCT
TGGATCGGAGACTAAACTTCTCGAAGCAAGTGAGTGCGATGAGAGTGCGTAT
ACGTGCAGCAGCGTCAAAGCACTTCTGGCTAATTAATTCGCGAAGTAAATTG
TCACTCTCCAACAAGGTGACAATTTACAAGCAAATTATAGCGCCAATATGGAG
GTATGGTTGTCAGATTTGGGGCTTGGCTTGCGACAGCCAAATTCGTCGCATTC
AGGCTGCCCAAAACAAAATCGCCAGGATGATTACCGGCTGCGAATGGTACGT
AAGGAACACAACCCTGCACAAAGACCTCAAGCTAGCCACTGTCTTTGAGGC
AATAAACATGCACTCCAGCCGGTACCACGACAGGCTAGAGCGCCACAGAAAT
CGCCTAGCCAAGGCACTGTCCAGAGCTCGCCCACCAAGAAGGCTCCACAGG
AGACAGCCGAAGGACCTCATCATACGGTCCCCCTTGACGAGAGCCAGGAGA
TGATGATGATCTCATAATTGTTATATTGTTAATTGTTATATTTGTATTATTGTTTT
GTTACAGCTAACTTTTGTTAGCCGGCGCGCCTGAAAGGGCTGAATTAATAGC
GACCAAGGTGACAAAGGGGTTTTCAGCTACCCCGTATGCCTTAATAAAGTATA
AAAAAAAAAAA

>BS2Jockey#LINE/BS2Jockey

AAGGTTCGTGTCTAACAATACGCACCTGACAAAGTGGATTAAGTGAAATTAG
TTTTCGCGGTAATAAACTTATGGACAAGACCAGAATACTGGCACACATAGCA
AATAGTGACCCCCCAAGTCACTAACAGTGAAATAATAGTGAAACGAAAACAT
TTTCATTCAAAAATACAAAGTTAAGTTTCTCGAACTGGGGCTCCGCTGCCCA
GCTGCCACGCGATCGCACAAACAGCTGTTTGCGAGCTTAAAGCTTTCTATCC
CAGGGTTCAAGTTTTGGCTAGAACCCTGGTGATTTGGTGCACACTTCAATATG
AACACTTCAAATGAAACCGCTGCGGCTGATGAATCGTTGGATACTGCGTTTCT
CTCGAGCCCCCAATGTGCTGCCCCGCAGCGCTTTCAAAAAATAAAGCGAAAG
TCTCGTGCTTCTCCGGAGACTGAAAGGAAAAAACCCAAATCAACCATCGGC
AAACAAGGGGAAAACCCTTCGGCTACAGAACCTAGATATGGCGGCAATTCAA

ACCGATTTGGTTTACTTGCGCATCTCACAGCTGACAAACAAGTAGGCAATGA
AATTGGCGATCTGTATGACCAGCCCAGTACCAGTCATCAAGCTGCAATTGCTG
CCGCTAAGCGGGATGCAGCCTCCGCTGGTACCACTAGCTCAGCCAAAAAGGC
GCAGTCCAAACCACCTCCTATAGTAATGGAGGGAGTGGACGACGTATACCTG
ATGATGCAGAGCATCGAAAATATAGTGGACCTAGAAAAGATTGAGGCTAGGG
CGTCAATGAGCGGTGTCCTAAGGCTTTACGCGGCTGACGCTAATACATTTCGC
ACCATAGTGAACTGGCTCGAGATCGAAGAGTATGAGTTCCACTGCTACCAGC
TTAAAGAGGACAGGCCTTACAGGGTATGCGTGAAAGGCCTGCACCACAGTA
CGCTACATCACCAAATCAAGGATGAGCTGGAAAAGATCGGGCACAAGGTTCT
CGATATTCACACACCGCTTAGGCGAAACGAACCGGGTACCTCAAAAGCGTCG
CCAGTCAATATGTTCTTCCTAAATATTGCTGCTGCGGCAAACAATAAGGAGAT
CCTGGCGGTAAAGGCACTATGCCATATGAGAGTAGTTATTGAGCCTCTCCGCA
AGCGTAACGCTATTGTCCAGTGCCATCGTTGTCAGCAGTTTGGCCACACAGC
CAAATACTGCCGTAAGGCCCACATTTGTGTGAAATGTGCCGGCGAACACCCA
GCCAAGGACTGTACCAGGCCACGCATCGAGCTGTGCACTTGCTACAACTGTG
GCGGCCAGCATCCTGCAAACTATAAAGGTTGCAGCAAGCTACAAGCGTTCCT
GCAGCGATCCAGACCCAGAAGTGGAGTGGCTGGAAGAACAGAAGTAAGCG
AGCGACCAACTCCACGGGGCTTAGCTGGAGGTAAGGAGATCCCCTCTTCTCG
AGGCGGAATATCTTATGCAGATGTGGCTAGAGGGTCCATTCACCACAAGCAA
CCAATGAGCCTGACGCACCAGCAACAGAAGCAACAGCAACAGCCCTATGAT
GGAAGCCCCAGTCGTCAAAGGAGCCGCAGCCGGACAAGGGCGTCTAGGGGT
ACACTCCAGCGCTCGACGGATGCTAGCAGCAGCATTGAAGCCATCCTGCAGA
CGCTTAATGAGAACATTAATTCTTTGCGCTCGATTCAAGAGAAGCAAATGGA
ATTAATGATGATGATGATGAAGCAACAGCAACAACAGTCACATCAGCAGGGG
CAGATTATCAATCTGCTCACTGCTCTCCAAGCGCGTCAAGCGCCATAATGATG
CCGCTGCGCATCCTAGTGTGGAACGCCGACGGCGTATCCACGAAGTTGCCTG
AAGTAGAGTGCTTCGTGCGACGTCACGAAATCGATGTATTACTGCTCAGCGA
GACACACTGCAAGGGGGCAGAGACGCCTAAGCTATTCGGATTTGTAGCCTAC
ACTGCCAATGATCCGAGTGGTGGCAACGCCAAAGGCGGAGCAGCTATCTTAA
TCAAAAATAGCCTTGCCCACTTTCCGCTAACACCAATAGCCACTGCCAAGGT
GCAACTTGCGCCGGCGGTTATTGAAACGGCACTTGGTCCTATAAGCTTTGGA
GCGGTCTACTGCCCACCGAGATTTGCATGGACTACGGACGAGTTTAAGGACA
TTTTGGAAGAGTTCCAGACGAAGTTCATTGTTGCAGGCGATTGGAACGCGTC
CCACTGGCTCTGGGGTGCGGGAAGGAGCAACCAAAGAGGCATTGCATTAGC
GAATCTCGTCCTAAATTCGGAGGTGGACTCGCTAGCAACAGGAGGACCAAC
AAGATACCCGTACGGCTGTAGAGGCTCACCAGGGTACATCGATTTTGCACTG
ACAAAGGGTGTGCTGGGCATCCACGCTAACATAAGTGCGGTTGTTGAGCTTA
GCTCCGACCACCTGCCTCTGGTAATTACGCTGGATGCGGGGGCAATATCCTAC
CCTAAGATGGAGCGGCTTATCACTAGGCGTACTAACCTGGAGGTATTCCAATC
GCAACTGGAGTCCACACTGCCCCTCAACACTGCCATAAACTCTGGACAGGAC
GTTGATGATGCTATCGAACTGCTCACCAACAATATCAAGTCAGCAGCTAGATT
GGCAACTCGCAGCATATCTCGGCAGCCCGCGGCAGATCGAATCCCAATACCC
AGGGAGATCCTGCTGCTTATAGCTGAGAAGAGGCGCTTACGCACTAGGTGGA

TGAGGTCTCGGCACCCGTCGGACAAAACGGAATGGAACCGAGCTCTGAGTA
GGCTCCGATGCGCGTTGGTGCTGCACAAAGCCGCATGGTTCGACGAAAGGC
TTGCCAATACCGGAGTCGAAAGCGAAGCGACGCATTCGCTGTGGAAGGCCA
CGCGCGCAATCAAAAGGCGTTGCACGAGGAAGGCGCCTCTAGTCGATAGCA
ACGGGACATGGTGTCGGACCGACTTGGGACAAGCGGAGGTATTCGCTGCGC
ACCTCGCCGAGCGATTTCAACCATTCAAGCTTGCCAGCCTGCAACAGGTTGA
AGAAACTCAGGACCAGCTGAACCAAGCGCTTCAAATGGATATGCCAATCACG
CCGTTTGAACCCTGCGAGGTAGCCGAAGTCATTGTGCGCCAGAGTAACAACA
AAGCACCTGGACATGACGTCATCTGCAACGCCACATTGAAGGCCCTGCCCAG
ACAAGCGATCCTCTACATAACGTTGGTTTTCAACGCTATTGTGAGGTTGCAAT
ACTTCCCTTATCAGTGGAAGCTCGGGATAATCTCCATGATCCACAAACCTGGC
AAGCCGGAAAGGGAGCCCGCCTCCTACCGGCCGATCAGTCTCCTCCCTTCAA
TTTCGAAGGTGTTTGAGAGACTGATTGCTGTCCGGATTGTAAGGATTATGGAA
GCCCAGGGGATTACCCCTGAGCACCAGTTCGGTTTCCGTGCTGGCCACTGTA
CTGTCGAGCAGCTCCATCGAGTCGTCGAGCAAATTCTGACTGCCTACGACAG
TAAGGAATATTGTAACAGCCTCTTCTTGGACATTCGAGAAGCGTTTGATCGAG
TGTGGCACATTGGACTCCAACTGAAAATCAAGCAGACGCTGCCTGCTCCATA
TTTTGGGTTGCTGAAATCGTACCTGGAAGGAAGGAGGTTCGCTGTGCGCTTT
CATTCAGCAATTTCCACCGAGCACAACGTGGCAGCTGGTGTTCCACAAGGTA
GTGTCCTCGGCCCCCTGCTCTACTGCCTGTATAGCCACGACATGCCGCAGCCA
GATGTAAGCCTTTACGGGAAATCTATGTTGGCCACATTTGCCGATGACGTGTG
CGTCACCTACAGGTCCCGATGCGAGCACGACGCAGCCGATGGTATCCAGGAC
TTTGCATACCGGTTCTCGGAATGGGCAAGACGATGGAATATTGGCATCAATAG
CAGTAAATCCAACAACGTCTGCTTCACTTTAAAGCGGAGAACGCCACCGCCC
GTCTACATCGAGGAAGTCCCCGTACCACAGCCGAACGCAGCAAAGTACCTTG
GAGTGCTTCTGGATCGCAGACTCACATTTTCCAAGCATGTGACCGACATCAG
AACGCGCCTACGTGCTAAGGTGGCGAAGCACTACTGGCTACTTTCTTCGCGC
AGTAAATTGTCGCTATCCAACAAGCTGACAATTTACAAACAGATCCTAGCACC
AAACTGGAAGTATGGGTGCCAAATCTGGGGCTTAGCCTGCGACAGCCACATC
AAAAGGATCCAGGCTATTCAAAATAAGGTAGCAAGACTCATCACCGGCTGCG
AGTGGTTTGTTCGAAACACCACCCTGCACAGAGACCTGAAACTCGCAACGG
TATTTGACGAAATAAACAAGCACTCGAGCAGATACCATGACAGGCTGGAGCG
CCACAGAAATCGGCTGGCCAGCGCTTTAAACAGATCTCGCCCACCAAGGAG
GCTCAATAGAAGGCAACCGAGGGATCTCATTACCCGATCTCCTTTGACAAGG
GTCCGCAGAAGCTGACGCTTATCTTAAATCCTATTTGTTATATGTGATTGTTAT
GTAATTGTAGTTAAATTACTGTAAATTTGAAAAAGCTAACTATAGTTAGCCGG
CGAGCCCAAATGGGCTGAATTAATAGATAAGAAGGACACAAAGGGGCTTCA
AGACTTCCCCGTATGCCTTAATAAATAAATTAAAAAAAAAAAAAAA

>BSJockey#LINE/BSJockey

CTGAAGTCGCAAATCTGCATTCATAGAGATCGGTTGTGTCGCGCGTATGCAAA
AGTGATTATTTTGCTTTATTATTGCAATTTCTTGGGTGCTTAAAATAGCACTCA
CCAGTACATTCGGGCGCTGCTTCGTGCGGTGTCGGCATTTGGCAACAACAAA

AAGCGTTAATCGAAGTGCGGTGTAGCTACGATACCTGCCCTTCGGGCTTATTC
CCCTCACCCCGCGCAAAGCCGCTGAAGGGGGCAATAAAATCTATGCTTATCA
GCAAAACTGATCCGTATTTGATCTGTTTTGTGGTCAGTTAAGCAAGCTATTTT
GTAAATATTAAGAATTATTATTAAGACAATGGATGAGAACAATTCTGATGACA
CCCAGCTTTTAAATAAGCAGAGTAACCATAGAACAATGTTCTCAATAGCTGGC
AAATTACCTCACGAGATTAGAAACGAGTGCCGATCAGCAATTCAACGCTTTA
CAAGCAGCGTAACTCAAAGCAGTAGCGTCACCACAACAACGGTGACATTTA
CTAGTGCCAATAACAGCACCATATATACAATGGCAAATGCCGCAATAAGCAGC
CCGTGCCTTGGAACAAGATCCACTCACCAGGAAAGTTCCACATTGATAAACT
CCGGAATCGTAGAAGATAATCTCAGCGATGCTGCCAGAAGGTTATTAAATGAC
CAAAATCAGAGAGCGGGTAAAAGGAAAAATGGAAAGCCCTTGTCCCCCATC
TCCAACCCGAAAAGAGGGAGTAGCAGCCAAGTTTTACACTCGCCCCCTACG
ACTAGCCTGAAGATAAGCACTAATAATAGGTTTGCCATTCTGGACACGGATAT
TTCTACTAACGAAGAAAGCGTGGAAGGCATGATGATAGAGGGTGCTGATATT
GACAGTGCCCATATGGATGATTCTCAACTCGATGGTTCCAATACTGGTCGAAA
CTTGCAGGAAACACACAATACAGCCAATCAACTTAATGATCACAAAAAACCA
CCACAAATTGTTGTAAATATCAGAAACTTGAATGATCTGTTTGAGCTTATAAA
AGAAAAGACAAGCTTAGATAACGTTGTCGTTAAAGCTAATCAAGGGGAAAC
GGTTAGAATATTTCCAAAAGACAGCGACACTTACAGGAAAATAGTGAGCCAT
ATGGATGACATTGGTATTCAGTTTCACACTTACCAAATGCTGACAGATAAGCC
ACACAGAATTGTAGTAAGGGACTTACATCACAGTACATCAAACAAAGACATA
ACCGCCGATCTGAAATGTTTAGGCTACGAAGTGCTCCACATTCACAACCCTA
GTTCTAGGACTAATAAGGACGAAAAACTAAACATCTTTTTCATTAATATAAAG
CCCTGTGCAAAAATTAATGAAATTTACCATGTCAAGACCCTTTGCCGACAGA
AAATACGGATTGAAAGGATGAGAAAGTCTTCTGAAATTGCGCAATGTCGTCG
TTGTCAGGAGTACGGCCATACAGCTAAATACTGCCGCAGACACCCAAATTGT
GCCAGATGTGGCGAAAATCACCAAACCATGCAATGCACCCGACCGATAGACG
CACTGCCCACATGTTACCATTGCTCTGAAAATCATACGGCTAGCTTCAAAGGT
TGCCTAAAGTATCAGGAGCTTCTTCGCAGATCTATGGGGCCTGCAAGAAATG
GAAACAGGTTAAATAAGAACACCCATCATCACTCTCCTAGAGACCGGCAAGA
GCTTCCTGCCTTGCAGCCCAATTACCGCAAGAACAACACCCAATCAACAGTA
CAGCAGTTATCGACACAACCACAGCTTAATTTTGCCCAAAGCCAACCATCTAT
AGGCACTGGTGGAAACAGAGCAGTATCATATGCTACAATAGTAAAAGGATAC
CCAAAAATAGCGCCCTCCAAGGACGGACCAGCCCAGCGTCTACGCTTAAAC
AACCCACAAACGAAACAAATCTGCAGCAACACCAGATCGAATACACAGCAG
AATAACTCATCTGATGTGCAAGTATTCTTACAACAGCAACAACAACAGTTTCT
GGAATGGCAACAGCAGATCCAACAACAACAACACCAACAGTTTCTTATGTGG
TTGCAACAGCAGCAGCAAGAACAACTACAGTATAAAAGCCAAACCAATCAA
CGACTGGAAAAGCTTGAAAAAATGGTTCTTGAACTAGCGAATATGTTAAAAG
AATGGGCTGGGAGTGAACTTAACCCCCAGCTTTTTAACAACGTCTCAGCCTC
CCTATGAATCCACTAAAGATTCTTATTTGGAATGCTAACGGCATTTCAAGAAA
AGCCAAAGATGTTGAGCTGTTCGCGCACAACAAAAAGGTAGACATCCTTCTT
GTGACTGAACTAAGACTCAAAAGAGGGGAAACTGTAAAGATATATGGATATG

CGTACTATCAGCCATATAGGCCATCCCTTAATAATAATAGTGTTGGCCGGAGTA
GCGGTGTTCGTGAGGACAACTCTTCGCCACTTTCCACAAAGGGTCATTGAGA
CACGCCACATACAATTGTCATCAGTAAAAGTAGCCACAGGACTGGGGGACCT
GCAGTTTAGCGCTATTTACTGCTCCCCAAGTACTAGAATCGAGGAAAGACATT
TTACTGACATAATACGCGCCTGCGGCCAAAGGTACTTGGTAGGTGGCGACTG
GAATGCCCGCCACTGGCTTTGGGGCGACACTTGCAATTCACCTCGCGGGCGG
GAACTAGCAGAAGCCTTGTCCGTGACTGGAGCTAAGATCCTCGCAACTGGCT
CTCCGACAAGGTATCCGTATGTGCCCAGCCATACGCCCTCATGCATAGATTTC
GCAGTGTATCATGGTATACCAGACCACCTAGCAACTATAACACAAAGCTGGG
ACTTGGATTCTGATCACTTGCCTCTTATCATTAGCATTGAGACAGACAGTACT
CATGTCAATCCAAGTCCCAGGCTAGTCACCAAACACACTGACCTCCTTGCCT
TTAGCCGACAATTGGAGAGCCTTATTTCGCTGAACACCACGCTTAATTCTGGT
GAGGAAATTGAAATGGCTGTTGACAACCTAACTGAAAGCATTCATAGGGCCG
CGGCTGTCTCTACTTCTCCCGTCCCTCGGATAGGCACCACATATGGGATAGTC
TTGACAAGAGAGGCTAGAGAGCTTCTGACACAGAAAAGAAGACTCCGAAG
GCGAGCAATCCGATCTCAAGACCCCTGGGACCGACTTTTATGGAACCGTGCT
GCAAAGCAACTACGAAACGTCCTCAGAGAACTTCGAAGCAACTTTTTTGAG
CAGAAACTAGCTAGTATGGACTACACAGTGGATGCTGGATACTCGCTATGGA
AATGCACCAAGTCCCTTAAAAGACAGCCGTTTAGACAGGTTCCTATAAGGTG
TCCGGGAGGCGAACTTGCTAAAAATGAAGAGGAGCAGGCTAATTGTTTTGCA
AATCATCTGGAGACAAGGTTCACCCACTTCCAATTCGCTACAACGGAGCAGT
ATCAAGAGACGCTTGATAGCCTAGAGACACCTCTGCAAATGTCACTACCCATT
AAGCCCATCAGGGTTGAGGAAATTGTCGAAGCTATCAAATCTCTCCCGTTAA
AGAAGTCTCCTGGCATCGACAACGTTTGCAATGCCACACTAAAAGCACTACC
TGTTCGAACAATTCTCTACTTGGCGCTGATATACAATGCCATACTCAGGGTGC
AGTTTTTCCCAAAGCAGTGGAAAATGGCAGCAATCCTAATGATACATAAGCCT
GGTAAACCTGAAGAGAGCCCTGAATCGTACCGACCCATAAGTCTTTTATCTTC
GCTATCCAAGCTATGGGAACGACTGATTGCCAACAGATTAAATGACATCATGA
CCGAGCGTCGTATCCTGCCGGATCATCAGTTTGGCTTTCGTCAGGGACACAG
TACTGTGGAGCAGGTACACAGACTGACAAAACATATCCTTCAGGCCTTTGAT
GATAAGGAATACTGCAATGCTGTGTTCATTGACATGCAACAGGCATTCGATAG
GGTCTGGCATGACGGCCTTATCAGCAAAGTTAAAAAGTTATTCCCAGCACCAT
ACTATGGAGTCCTAAAATCATACTTGGAAGATCGGAGATTCATGGTCAGGGTC
AGAAACTCCTACTCGATTCCCCGCGTTATGAGAGCTGGAGTTCCGCAGGGCA
GCGTACTGGGACCGTTGCTCTACTCAGTATTTACTGCAGATCTGCCCTGCCCA
AACGCCTATCATATGGCAGATCCCAGGAAGGCCCTTCTTGCTACGTACGCTGA
CGATATTGCCCTGCTGTACAGCTCTAATTGTTGCAACGAGGCAGCAAGGGGT
CTCCAAGAGTACCTCACCACTCTGGCTGCATGGTGCAAAAGATGGAATTTAA
AGGTCAATCCGCAAAAGACCATCAATCCCTGCTTCACCTTGAAGACCTTAAG
TCCCGTCACCGCACCCATAGAGCTGGAAGGTGTAATCCTAGATCAACCTTCA
CAGGCTAAGTACCTCGGGATTACCCTTGATAAACGGTTGACTTTCGGCCCGC
ACCTGAAAGCTACGACTCGGAGATGTTATCAAAGGATGCAACAACTTCGATG
GCTGTTAAACAGAAAAAGCACCATGACACTGAGAGCCAAAAGAGCAGTCTA

CGTCCACTGCGTAGCCCCGATCTGGCTGTACGGAATACAGATCTGGGGTATCG
CAGCAAAATCCAACTACAACCGCATTCAGGTATTGCAAAATCGTGCCATGCG
TGCAATTACAGACTGCCCATACTATGTACGTGGCACTACCCTTCACCGTGATC
TGAATCTTCATACAGTGGAAGAGCAGATCTCCAAGCACACCAGCAGATATAG
TGATAGACTAAGACGACACCACAGTATACTTGCTAGACGCTTACTCCCTGCTA
GGCCTCTAAGGAGATTAAAAAGGAAGGGTTTCGCCAAAACACTTGGACAAC
CCTAAAGACCCCCTCGAAATATGAGACATAGTTGTAAGTCCTCACATGATTAG
TGAGAGGTTTGGTTTTATCTTTTATATGTTAATTGCGCTGTTATGTTACTGTTAT
TGCATTGTATTGATTCATCGCTTCTAAATAAATAAATATAAAAAAAAAAAAA

>BLOOD_LTRGypsy#LTR/BLOOD_LTRGypsy

TGTAGTATGTGCATATATCGAGGGTACACTGTACCTATAAGTACACAGCAACA
CTTAGTTGCATTGCATAAATAAATGTCTCAAGTGAGCGTGATATAAGATCACC
CATTTATGCTTTAAGCTAAGTCAGCATCCCCACGCTGGCCGCTGGCCATATATG
CGCATAAGCTCTCTCTCTCTCTCTCTTATACATATATATATACGCTGCTCTTCTG
CCGCTGTCGACGGCGGCGCAGTCGCAGTATTTAGGTAAGATTAGACACTCTG
TAGAGGTTAAGCGGGCAGAACCGTTTCTGCTACTCGAAGAGATAAGAAGAA
ATAAAAAGGTGGCCTGACGGCTGCACCCAACTGCAAGGAAAACACGTGTTC
TCAATTGGTGGCATATATTGGTTTATTACA

>BLOOD_IGypsy#LTR/BLOOD_IGypsy

TGGCGACCGTGAGGCAGGAGCCTGCGATCTGAGGACTACTGAGGAAATGCT
GCTAATATTGCCGATTTGATTTGGGAATTCTAAACAGCGACAACAGGTGTGAG
AGGCAGGCCGCCCCTTACACCAGTGCGGGAGACCTAGAGACGGGACACTGA
TGAAAAAAAAAAGAAACAAAAATACTGAGTGAGTAGAGTGTGGTAATGGGC
AAACGCGGATGTCAGGAAATCAAAAATAAAGGTATAGCACATATTAAGTGGC
TATGATATACAAATAAAACACCGCCCCCATGGGCAACGGCACAGAAATTAAC
TGCCGAATTAGACTTTCTGAAAGAAAACCTCCAGCAAAGAAAGCCGAATAC
CACAACTCACTCAGCAAAAATAGAAATAATCAATGAAGAAATAACTGAAAAT
TCAACATCACCCAAGCCGAAAAGACCCGACGTCTGCATGAACGACTGCCCT
CGACCATTGTAAGCCGCAACAGCAATTAGCACGGCATCCTGCGAGGGTAGGA
TTAGGATAAAGGATAAAGGATTCCACCGGCGCGCCGCACATGACAACAGCGA
ATGTCTACCAAGCAGACGTTCGAACACCCTGCTCCTGTCGAGCAAAGGGATC
TGCCAAGTATCAAAGAGGTAATAGAGGTAGATCCGTCCGCGGGACCAAAGCC
CTTGACCATACAAGAGTACAAGGCACGGACTGCAGCGAGGGAGCAGCCACC
TAAAAAGAAGAGGGGTGGCCGCCGGATTAAGTTGCTCAGCGCCCGGAGGCT
CAACATCGAACTACTGAAGACGGCAACTAATGAGGAAGACCGGCAGCGCTA
CAAAGAGCGCCTTGCAGCCATCAATCAACAACTTCGTGGTGCGAAGTAAAG
CGGCGGGCTGCGTTATACGCCATAGCCTCAACCGCCCAAATATTATATTAATGT
TGTCGATGCGGTTTCCGCTGCAACAAAATTACTAACTTATCAGGGACCCATTT
CATAACTAACACATTATACTCAGTCCTAAACTTAAAATAAGTAATAATATTGTA
AAATTGCAAATTGCAACCGATGTAAACTGAGTATAATGAATTCATCTATCAAG
TAAAAATATGTTTAACAACAGTTTAGACCTATTAAAATTTCGAGCTATATTTAT

ATCTGATCGAGATAACAATAATTGACCAATTCTCAAAGTTAAAATTCTATTTGT
ACTTTTGATATACAAATAAAGACTAATTTTCCCCATATCAAAATGGGACATAAG
TCGTGGATACAACCCCACAGTTAAATTCAATGTACTTACTATTTTTGATTTTAG
TTATCCTATCAGCCTTTTAACCTGGCCTTAAAACTTTATCAGTTTCACAAAAGA
TCGTTGAAAAGACTTACATGAGTCGAGCCAATGATTTAGACAAAATCTAATAG
AAACTACACCAAAAAGGTACAAGTGCGATTACATCGCTAAAAGGTACATACA
TGGAATGGCTAAACTTAACCATATCCATAAACAATATTAGAGATGCTTTTGATA
AATCCTATAAATGTATTAATAAAACCGCGCTGATCAAAACTCAGACGCTTATTT
TTCACATAAAGGTATTGATAACACAATACAACACATTACAAAACCTAATAGTA
ACAAACAAAAGCAAACTCACTGAAGAACATAAAGTCCAATGCTTCAAAGTT
CTCAGTTCATTTGGTAAAAGACTACATAATACCAGCGTTAGACACAGTATTAT
AATAGAAGTCCCAACAGAACTAACCAAAATAGCAGAATTCGACGAAAGCCA
GTTAAGAGACTTGGACGAGTCGCAGCCGTTAGAAGATTTAGATATCGAAAGC
GATATCGAATCAATAGAAGAATTAAAATTTAATACCGTACAACCAAATACAAG
AAACATGGCCAACGCATTAGAAGCTCAGAGAGCATACGTTAAACAGGTATCT
GCCACAGTACCTGAATTCGATGGTAAGAAACTCCATTTAAACAGGTTTGTGA
CAGCACTTAAGTTGACGGATCTAACTAAAGGAGATCAAGAAACTTTAGCAGT
AGAGGTCATAAAGACCAAAATTATTGGCCCATTAAACTATAAAGTAGAACATG
CGACAACGATACAGGCAATAATTACCATATTGCAGGCAAACGTAAAAGGCGA
ATCGCCTGACGTTATAAAGGCCAAATTAATAAATGCCCAACAAAGAGGCAAG
ACCGCGTCTCAGTATGTTACAGAAATAGACAGTATGCGTAAGCAGCTCGAGG
CAGCTTACATAGACGGCGGATTAGACGCCGATAATGCTGACAAATTCGCGAC
TAAAGAGTCGATATCAGCAATGACCAAAAACTGTGCCAACGAGGCACTTAAA
ATGATCTTAACTGCAGGTACATTTAGTACATTCAACGACGCAATGGAAAAATA
CCTACATTGCAGTACAGAAATAACCGGCAATTCAAATACAGTCTTATTCTATA
ATGGGAATAATAGACGTGGTAATTATAATGCCTACTATCGTGGTAGAGGCAGA
AATAATTATAACCATAATTATAACCAGAATTATAACCAAGGTTATAATAATAACA
ACAGAGGTCGCGGAGGCTACCGCGGCCACGGTAATAACAGAGACGGAGGTA
ACCGAAGGGGTAACCAAAGTCAGAATAATAATAACAACCGAAATGTGCGTAA
CGTACAATCGGAAAACAGCCAGACCCCCTTAAGCGATCAACAGTAAAAGTGT
TTAAAGTAAACCTAAATCTGAGTATTTTCATTAAGACAAAAAACCATGAAACA
AACACAGTTCTTACATTACTAATAGACACAGGTGCAGAAATTTCATTGCTAAA
AGCCAAAGCAAAGGAATATAATAATATAAATTTCAGTAATATATCAAATATTAC
AGGTATTGGGCAAGGAACCATACAGTCTATAGGTACAGTAGATCTTGACATAC
GCATTCAGGATGTTCTAGTGCCACATGAATTTCATGTAGTACCTGAGAATTTTC
CGATACCATGCGATGGCATAATCGGAATAGATTTTATCAAGAAATACAATTGCG
TATTAGAGTTTCAAAATAACAAAGACTGGTTCACAATAAGACCCAATAACTTC
AGTAGACAGATTAGTGTACCAATTACACATAACTTAGACTCCAACACACTCTT
ATTGCCAGCTAGATGCGAAGTAATCAGACAAGTCAAATTACTCACTAACGAA
AAAACGGTGGTAGTACCAAATCAGGAGCTGCAACCAGGTATAATAGTAGCAA
GCACCATTGCCGATAGCAAAAACGCATTGATTCGCATTATAAATACAAATAAT
AAAGACGCCATAATAGATAGCGCGAAGATCAAATGCGAATCAATGAAAGACT
ATGACATTTTTACAACACCAGTAGAAAAGGAAAATAGAACTGAAGAAATTTT

AAAACAATTAAGATTCCCTAAACAATTCAATAATGAACTAACTAAGTTATGCA
CCGAGTTTAGCGATATTTTTGGTCTAGAAACAGAACCAATATCGGCTAACAAT
TTCTACAAACAAAAACTCAGATTAGGGGAAAAAACACCGGTCTATATAAAAA
ACTATCGCATGGCAGATAGCCAAAAACCAGAAATCGCCAGACAGGTAAAAA
AATTAATAGATGATGGAATAGTTGAACCATCAATGTCTGAATATAATAGTCCAT
TACTTTTGGTTCCAAAGAAACCACTTCCGAATTCCACGGAAAAAAGATGGCG
ATTAGCAGTTGACTATCGTCAAATAAATAAGAAACTATTATCAGACAAATTTC
CACTTCCAAGAATAGAAGATATTCTTGATCAATTAGGAAGAGCAAAGTATTTT
TCATGTCTCGACCTAATGTCTGGATTCCACCAGATAGAACTAGAAAAAAGGT
ATAGAGATATAACGTCATTTTCAACAGCCAATGGCTCATATCGCTTCACGCGAT
TACCATACGGACTGAAAGTAGCACCAAACTCCTTCCAACGTATGATGACACT
TGCATTTTCTGGTCTTGAACCATCGCAAGCATTTCTATATATGGATGACTTAGT
AGTAATAGGTTGTTCAGAAAAACATATGCTCAAAAATTTGACTAACGTATTCG
AGCTATGTAGACGACATAATTTGAAACTACATCCAGGGAAATGTTCTTTCTTT
ATGAAAGAAGTAACATATTTGGGTCACAAATGTACCGATAAAGGTATACTCCC
AGATGACACCAAATATGAAGTTATAGAAAAATATCCTATACCAACAGATGCCG
ACAGTGCTAGGCGTTTCGTAGCCTTCTGTAATTATTACAGACGTTTCATTAAA
AATTTTTCTGATCATTCACGCCACTTAACGAGGCTTTGTAAAAAGAATGTTCA
ATTCGAATGGACAGCAGAATGCAATGATGCATTCGAATACCTTAAAACAGAAT
TAATGAAACCAACATTACTACAGTACCCAGATTTCGGTAAAGAATTTTGCATA
ACAACCGATGCTAGTAAACAGGCATGCGGAGCGGTACTTACACAAGATCACA
ATGGTCAACAACTTCCAGTGGCATACGCTTCAAGAATGTTCACTCAAGGTGA
AAGTAATAAGTCCACTACAGAACAAGAATTAACGGCCATTCATTGGGCCATA
AATCATTTTCGACCATACATATATGGCAAGCATTTCATGGTAAAAAGCGATCAT
AGACCATTGTCATACCTATTCTCTATGAAAAATCCAAGTTCAAAACTCACTCG
TATGAGGCTGGATTTAGAAGAGTATGACTTTACTGTAGAATATCTTAAGGGGA
AAGATAACCATATTGCGGACGCCTTGTCTCGCATAACAATAAAAGATCTGAAA
ACAATCAACAGAGAAATATTAAAAGTTACCACCAGATCAAAAGCTAAACAGG
AAAATTCCTGTAAGGACGAAGCAATAGTCAAAATACAAGAGGAAAAAGAGC
AAACAATAGAAAAGCCCAAAGTCTATGAAGTTGTCAATAATAATGACACAAA
GAAATATGTTTTAATCAAAATAGATAAACACAAGTGTTTATTAAAACGAGGAA
AAACAATTGTTTCACGCTTTGATGTTGATGACTTGTATTCTAATGAAACATTTG
ATCTAAATCAATTCTTTCAAAGGCTTATTTCAAAAGCCGGAATGCATAAAATA
ACAAAAATGCGAATATCACCAAGCGAACAGATGTTCCAATTTGTATCACTAAA
TGAATTTAAAATAAAGGGCAACCGAGTACTCGAAAAAGTAGAACTAGCTATT
CTACAAAAGGTGATAATTATAGACAAAAATGACGAAGCTCAGATTAAAGAAA
TTTTGACAAAATTCCATGATGATCCTATAGAAGGAGGCCACACTGGTATTTCG
CGAACCCAGTCAAAAATCAAAAGATTTTATTATTGGCCCCAGATGACCAAGA
CAATCTCAAAGTATGTAAAGACTTGTTTGAAATGTCAACAAGCCAAAATTAC
AACACATACGAAAACTCCATTAACATTGATGCCAACGCCAGCAACAGCATTT
GATACTGTTTTAATTGATACCATTGGTCCACTACCGAAATCGGAAGACGGAAA
TGAGTATGCAGTTACAATCATATGCGATCTAACCAAGTTTTTAGTAACTATTCC
AACACCAAATAAAAGTGCTAAAACAGTTGCAAAGGCTATATTTGAATTATTTG

TACTGAAGTACGGTCCAATGAAGACGTTCATTACAGATCAAGGTACGGAATA
CAAAAATTCACTTATGAATGAATTATGCAAATATATGCATATAGAAAATCTAAC
ATCTAGCGCTCACCATCATCAAACTTTAGGAACAATAGAAAGAAGCCACCGA
ACTTTTAATGAATATATACGTTCATACATATCGGTTAACAAAAGTGATTGGGAC
ATTTGGTTACCATATTTCACTTATTGCTTCAATACAACACCCTCAATAGTCCAT
GACTATTGCCCATACGAACTAGTATTTGGCAGACTACCCAGACAATTCAAAGA
TTTCAGTAAGATAAACAAAATAGACCCAATATACAACTTAGACGACTACTCTA
AAGAGCTTAAATGCAGACTAGAATTGTCGTACAACAGAGCAAGAAGAATGTT
AGAAAAAGCAAAAGCGGATAGAAAATTAAGATATGATAGGAATACAAATAAT
TTCGAATTAAAAATAGGAGATAAAGTATTACTTAGAAAAGAAACAGGTCATA
AGTTAGATAAAAGATATGAAGGTCCTTATGACGTAGTAGATATAGGAATAAAT
GACAATATAACCATTAAAACAGGAAGTAAGAAACAACAAATAGTACATAAAG
ATAGGCTAAAAAAGCACAAATAGAATGAAAAAAAAAAAGGGCAATCAATGC
CAAACCTTTCATAATAAAACTTAAATAACGGCCTGATCAGCCAAAACAATATA
ACAAAGACATAGATATAATCGAATTTTTATTAATTCAAAATACATACATATTTTT
TCTTTATTCATTTAAAAATTCTATATCATAAATAATGTTAATTCATTAAAAATAAT
ATTTAAGTAATTTTTATTTTATAATGGTAATATAGTTGATAGAAAATAACTTCAT
TTCTTTACGTTATTTTAAAAAAGAGGGGAGG

>BLASTOPIA_IGypsy#LTR/BLASTOPIA_IGypsy

TGGGGGCTCGTCCAGTCCTAAATCGGTTATATGAAGGTGCAGTTGTTTAAAGA
AAAAAGACATTGTTGTGTGCGTGGGTATAGTCTTTAAAACGTTGTAAAGTTGT
GGCTATATCTATTGCATTTAAAGTTGGAAAAATCAGTTGTACAGATTTTGTTTG
AACACAAGTCGGTAAAAGTCGGGAAAGCTGCTAGAGAGAACTGATAAAGTT
GAAATTGTCGTGTGCGTGGATTTAGTCTTTAAAGTTGTAAAGTTATGGCTACG
TCTACTGCATTGAAAGTTGAAAAAATCGATTGAACTCATACAGACTCAAGTC
GTTTTGCTGTTGTGGAATTTAAAACAATTAAATTGCAAAGGTGGTGAAATTCG
TTTCTAACGAAAATCAAAATTTGTCTTTTAACCGGTGGCGCCGTCTGCAAAAT
CGACTACCGTCGCGCCGTTAGAACATTGTCGTTGTTTGCTGGTGTTAGTGCCT
TGTCGCGGAATGTTCACACGTACACCACCTACAAATAAAAAACTTAACACCG
ACCAAATACAAGCAATTCTAGAGAACGAAAGCGAGGACGAAAGCAGAAAA
GAAAAAATGAACGAAGAAGATCAAAAGTTGGCGCCTGTAGGAGAAGCAGA
GGCAAAGAAGCAGAATAAAGACGCTAGTGCTAAAGTCGAAGAGAAATTTGA
ACAAATGATGAATACTCTAACCCAGAGCATGTTGGCAAAATCTAAACAAGAG
GGGCAAGTAATTATCGCTGCAGAAAAATTTGAAAAAGTTGTAAGTGACTGTG
ATGGCAAATCAATTCCTATTAAAAAATGGTTTGAAATTTTTGAGAAAAATGCC
GAGGCATATGAACTTTCGGAGAAACAAAAATATGTTCAAGCCAGAAGTAAGA
TGATTGGATCAGCAGAACTTTTCTTAGAATCTGAATGTGTCAGTGGATACACT
GAACTCAAAGAGTTACTAATTGAAGAATTTTCAGGCAGCTATAATAGCGCCGT
TATTCACAAAAAGTTGCAAGACAGGAAGAAGAAGAGGGAGGAAACTCTACA
CGACTATTTGTTACAAATGAAGAAAATAGCAGCCTTAGGTGAAGTTGAAACA
GTTGCTTTGATAACTCATATCGTAAACGGCCTCGACATTAAAAAGGAGTATAA
GGGTGCTATGCTCCGTTGTAAAACTCTTAAGGAATTAAAGCAAGAATTCGAA

ATCTACGAGAGTCTGAATATTGTTGACAAGCCGAATATTCAACCAAAACCAA
AGCAAATTACACAAGGTGTAAAAGCAGATCACTGCTTCAACTGTGGTTCGAG
GGAACACAAACGAAAGGATTGTACACTTCCTACCAAATGTTTCAGCTGTAAT
CAAGAGGGCCATATCTCAAGCAAGTGTCCGGAAAAAGTAAACAGCATGCGC
ATTCACGTTGATAGTGCACGAACAAAGCCAGTAATCATAAATGGGATTATCAT
CAACTGTCTGGTGGACACAGGATCAGATGTGACCATAATTAAAGAAGCTATAT
TCAAGAAGATGAAAGATGTTGATTTAAACCGCACTGCAACAGTATTGCGAGG
TTTGGGAAATGCCTCAACACAGCCGATTGGATGCTTCAGAGCATTAATCAAG
ACCGACCAGGTGGAAGCAAGCCACAACGTTTTAGTCGTCCACGATTCTAAAT
TCAGTTGCGATGGAATAGTGGGACACGATTTTATCAGCAAGTTTCGTCTTATC
TGTAGTGCAGAAGGCTATACTTTTCTTGACCTGGAAGCAGATAAAAAACAAG
CGGTTGAGTATTCCCAAATGTTTAATATTTGTGAAGAATCTTCTTTTACAGTTG
CACCACAATACCGAGAAGACGTTGAACGCATGATAGAGAGAACATACGAAA
CACCACCCAAGCAGATAAAGCAATGTCCAGTCGAACTCAAAATTATTCCTGA
TGGCGTGATTAAACCCTTTCGCCATGGACACACCCGACTATCTGAAGAAGAA
GCTATAGCTGTAAAGAAGCAGGTAGAGGAATGGGTCGAGCAGTCAATCGTCC
GTAAATCTACATCAAATGTTGCCAGTCGCATAGTCGTTGTCAGGAAAAAGGAT
GGTACCCTACGCGTTTGCGTGGACTATAGAAAATTGAACACCATGGTTCTGAT
GGATTGTTTTCCGGTACCCATAATGGAGGAGGTGCTTGAAAAACTGCAGAGT
GCCAAATGGTTTACAACCATGGACTTACAGAACGGATTTTTTCATGTGGCCGT
AGAAGAAGCCAGCAAGCCGTACACAGCATTTGTTACCCGAGAAGGCTTATTC
GAGTTTAACAAAGCGCCCTTTGGTTTTAAGAATTCCCCAGCAGCGTTTATACG
GTTCGTTCAATTTATTTTTCAAGAACTAATCAATTCCAATATAATGCAGCTATAT
ATGGATGACATAATTGTATATGCCGCTACCCCAGAAGAATGCATGGAAAAGAC
GGAAATGGTACTTAAGAGAGCTGCAGAATTTGGTCTAAAAATAAAATGGAAG
AAGTGCAACTTTATGCAGAGGCGAATTCATTTCCTGGGACATATTATCGAAGG
TGGACAAATATGCCCTGGAAAAGAGAAAACATCAGCAGTGAATTCCTTTGGA
ACACCTCAGAATGTAAAAGCCGTTCAAGGATTTCTGGGTCTCACAGGATTCT
TCAGAAAATTCATACCTGGATACGCCCAAATTGCGAGACCACTGACGGACCT
ATTAAAAAAAGATGCCATTTTCAACATTGGACCAGTAGAGCAGCAGTCGGTG
AATAAGCTGAAAGAGATTCTGGTAAACGAACCAGTATTGAGGATCTACTCAC
GAGAAGCAGAAACCGAACTTCATACAGATGCCTCTAAGGACGGGTTAGGAG
CCGTTTTATTGCAGAAGTTCGAAGGCAGTTTTCACCCAGTCTGCTTTTGGAGC
AGAAAAACTACAAAAGCCGAATCAAATCGTCATAGTTATTACCTTGAAGTAA
AAGCCGCATACTTAGCTCTGAAAAAGTTCAGACACTATTTATTGGGAGTCCCT
TTCAAGCTCGTCACGGACTGTGTCGCATTTAAACAGACAACAAAAAAAGCA
GATGTCCCAAGAGAAGTTGGCCCATGGATTCTCTATATGCAGGATTTTAATTTT
CAACCCGAACATCGTGCAGGAGAAAGAATGAGACACGTTGATTTTTAAGCC
GCCATCCCCAAGCATGCATGATGATAACATCCGAGTTGACAGCACGTATTAAA
AAGTCGCAGCAGAACGATGATTCAATTAGAGCAATCCTGGAAATTCTAAAAG
ATCGTCTATTCCAACCCTACAAGCTAAAAGGTGGCCTGTTGTATAGTATGGTC
AATGGCAATGAACTACTGGTTGTCCCTGCACTAATGGAGAGGGAGGTGATTC
AAAGCGCACATGAAGTTGGCCATTTGTCGTTGCAAAAGACGATGCATAGCAT

ACAGCAGCAATTTTTTTATTTCCTCATTTGGGAATACAAGGTAAAAAAGCTAA
TTTCTAACTGTATAAAATGTATCATCCACAGCAAAAAGTTGGGAAAGCAGGA
GGGATATCTAAATTGCATAGATAAAGGAGACGCACCGTTGCACACACTACAC
ATCGATCATTTGGGGCCAATGGATTCATCGGCCAAACAGTATAAATACATTCT
GGCAACAGTCGATGCGTTTTCAAAGTTTGTCTGGTTATTCCCAACCAAATCAA
CCGGACAGGAAGAAGTGGTCAAGAGGCTGACCGACTGGTCAAACATTTTTG
GTTTCCCTAAGCGAATTGTTAGCGACAAAGGAACGGCCTTTACGAGTGGTGC
GTTCGAACAATTTATGAGCAGCCATAACGTGGAACACGTCTGCACAACTACT
GGAGTGGCCAGAGGCAACGGCCAGATAGAACGAGTAAATCGTTTAATTTTGG
CAATAATATCAAAGCTGTCTTCAGACGAACCGTCGAAGTGGTACAAATATGTG
CCTGAGGTACAAAAGGCGATCAACTGTCACGTGCATTCATCACTGAAGCTGT
CACCATTTGAGGTCATGTTTGGCACCAAGATGTACACCCGAGTTGAGGATCG
GTTACTGGAACTGCTCCAAGAAGAAGTGGTCTGTCAATTCAACGAGGACCG
CTATGAGATGAGACAGCTGGTAAAACGCAACATCGAGCAGGCGCAGAAGGA
CTACAAGCGCAATTACGACAAAAAGCGCCGAGCTGAATACAAATACAAAGC
AGGTGATCTGGTTGCAATTAAAAGGACCCAATTTGTAGCTGGCCGCAAGATG
GCAAGCGGGTATTTAGGTCCATACGAAGTCACAGGGGTCAAAGACAATGGCA
GATATGACGTTAAAAAAGCAGCAAACGTCGAAGGACCCAATGTCACATCCAC
CAGCTGTGACAACATGAAGTTGTGGAAGTACATAGCCGAAAATGCAGACCTA
TTGTCATCCGGGTCGGATGATGATGATCAGGAGGGCCGAA

>Bica_LTRGypsy#LTR/Bica_LTRGypsy

TGTCAAGAGGAAGAGCATGAGAGAGGGGGAGTTGCTTGCGAGATGCCAGGG
AGTGCGGACGTGTGCTCGCTGCGTGAACGATGAAGGCAGATGTATACTGAAG
ACATATAACTATTGTAACACATTAATAAAAGAAAAGAATAAACATGAAGATCA
ACCTGACA

>Bica_IGypsy#LTR/Bica_IGypsy

ATTCAGAAGTGGGATACAAATTCATTTCTAAGTGGAAAATGTGAATTAAAGA
AAACGAGTTAAATAGTAAACGATTGGATTAGAGACGTACAACAAAACGGCAC
GGAATAGCAAAACGGTTGTACAAGAGCAAAGAGAGATTCGGAAAAACGGCA
CGATATAGCAAACGTCGAATAAAAAAAAAAAAGGGAAAAGAGGAATAGTAA
ACGATTGGATTAGAGACGTACAACAAAACGGCACGGAATAGCAAAACAGTT
GTACAAGAGCAAAGAGAGAAACCAAGGAAGATCGACAAACGGCGAGGAAT
AGCAAAAGGCAAAGAATCGCAAACGGTTGATCGAAAGGAAAAAGCAAGAA
ACAACGGTAACGGTTAAAAAAAAAAAGGGTATACATACGAAGCCACAAAGG
GTACAGGTATAGAAGGAAAATAGAGAGAGCAACGTAGACGGACTGAGCGAC
AAACTTGGCGGGAGAGCGAATCAGAACGGCAACGAGTGCAGACGTGCAGA
AGTAAAGGCTCTCGAAAATGCGTGTAAAGTTAAGGGATTTAAAGATTGGGGA
TTTGGACGTGATCTTGAGGGCTCATGATCTCCAAGTCAAAGGAAACAAAACG
GCAAAGATCAATGAGTTGATGACGAAACTGGAAAGTGATGAAATTGAGACA
GACGACTACGATATAATGGAACAAAACGCAAGTACAAGTATGCAAGCCCAAA
TCAACGAACTTAAGGAGATGGTGGCAAACTTGGCAACGATGGTCCAAACGG

CATGCTTGCAGCAACAAAGTCAAGCACAAGGGACTGTGGAACAAGAAACG
ACACGCTTACGTCAGACGCTGGATGGATCGAGACACACACATGCGCCAAAC
GGGATGCAACCGGAAAATGTGCTCTCATCGCCACAGCTAGAAGCTCAAGGTC
AAGGTTTTACAAGCATCAAAGAAATGATAGGAATTCTGCCGGATTTTGATCCA
ATCAAGGGATCTATCACATCGGAGCAATTCATCGCAAAGGTAGAGCAACTAC
AAAGCGTATACATGTGGACAAGTGACGCTGTGCTGTTTGCAGTGCAGCATAA
GATGCGTGGAGTAGCAAAAACATGGCTGGATGCACAGCAGGTATATCGATCA
TGGGATCAATTCAAATCGGCATTCGCTCTTGATTTTCCATGTTTAGTGAGTACG
GCCGATGTGCACAGAGAATTGATGCGTCGCAAGCGACGCAATAGCGAGTCAT
TAATTGAATACTTTTATGCGATGGTAGCTATTGGACGGCGTGCGAGTGTCGAT
GAACCATCAATAAATTCGTATATCATCAACGGGCTTAATTCAAAAGAACTCAC
GAAATCATTATTAGCGATGAACATACGCACTTGTTCAGAGCTGCTAAAGTCGT
TGGAAAATTTGAGATTTTCACAAGAGATACATCAACAACAATACAACGCGTC
AACGATCAATGAAAAGCAATACAACGATGCTGATGGCAAGATGAAAGCAGTT
AAATGTTATAATTGCAATAATTTCGGGCACTTTGCAGCAAAATGCACAGTGCC
ACAGCGAAAGGAAAGATGTTCCAAATGCTCTAAGATTGGGCATAATGAAAAG
GATTGCAAGGTGAAAACATCGTCAACGGTGGCTCAAGTGAATGAGTCGCATC
ACAAGGAGTATCCGCCAATAATGAAAACAGTTCTCATTGGAGGCCAACAGTT
TGAAGCATTCATCGACACGGGCAGTGATAACACCCTAATGAAAGAAGCAGCA
GTGCCAGATGGAGCAATACGGCAGCCGAGAGTCAAACGTCTGAAAGGCTTT
GGAGGATCTGTGGTCGAATCGAAGGAGTGCATTCTTTCAGAGTTTGCATATG
GCAAACTGAGACTCTTCACGCAAATTCAGGTGGTACCAAACGAAGTCCTACC
ATATGATGTCCTCGTGGGCAGAGATATCATCTGCCACGATCAGGAAATGCTGG
TTGCCGAAGATTCAGGATCAACGTTGACAGCAGCGGAGCAGGTCGGGTTTA
ATGTTAACACGGACATTGACCCCGACCACCAGGAACAGGTGAGCGATCTATT
AAAGAGCTATAAAGAGTGTTTTGCCGAAGATTTGTCAAATATTGGCAGGTGC
AAAACGACGAAGATGGATATAGAGGTATCCACTACGAAAGCCATCTTGGGGC
GGCGATATCAAGTGCCGTTTGCCCAAAGGGAGATGATGACTACGATAATAGG
TGACTTACTGAAGTACGGGATAATTGAAAGGAGCAAGTCAACGCATGCAGCA
TCAGCAATATTGGTGCCAAAGGCAAATGGAGAACGGAGGCTATGCGTGGACT
ATCGGGCTCTAAACGCAGTCACTATAAAGAAGCGATATCCGATGCCGATTGTA
GAGGAGCAATTGGCAAAGCTATCCGGAAATGTATACTTCACGACGCTGGATAT
GACATCCGGTTATTACCAGGTTCCGATGGACAAGAAAAGCAAAAATTTGACG
GCATTTATGGCACCAGATGGACTGTACGAATTTAATGTCATGCCCTTTGGTCT
GGTGAACGCACCGATGGTCTTCCAAGAAGTCATTACTGAGATCATACGAGAG
CTGAAACACCAACGGAATATTATCAATTATGTGGACGAAGTCATAATTGCATC
CAAGACGGTCGAAGAAGGACTCATTATATTAAAGGAATTCCTGGATGCTGTTA
AGTTGGCGGGCTTAACGCTGCGCCCATCCAAATGCGCTTTTATGAAAACGAA
GGTGACCTTTTTGGGTCATGTGATCACGGGCAACGGGATTCAGCCAGGCAAT
GAGAAGACTGACTGCATCAATGAATATCAAAGGCCATGTAACGAAACAGAA
GTACGCAGATTCCTGGGAGTTACAGGATTCTTCAGAAAATTTGTCAAGGAGT
ACAGTATGATTGCGTATCCATTGAGCAAATTATTGAAAAAGGATGTGGACTTT
ATATGGGGAGAAGATCAGGAACAGGCATTTCAACGACTAAAGGATGCTTTAA

CATCGAAGCCGGTGTTGACTTTATACGACCCCACAAAATATCATGAAATACAC
ACTGATGCTAGTAAGCTGGGAATAGCAGGAATTATGTTTCAAAGAGAAGAAG
ACGGAACGAAGCCTGTATTTTATTACAGCCGGCTATGCAGCGACGCAGAAAG
CAGATACTCAAGCTATGCTCTAGAAATATTGGCAATTACGGAGACACTGGTTA
GATTCCGGATATATTTACTAGGATCTCCATTTCTGGTGGTGACAGATTGCAACG
CGGTGGCGACGCTGAAGGAATCAACAGAGCTACAACCACCAATAGCAAGAT
GGTGGCTCAAACTACAAGAGTTTAATTTCACATGCAAACATCGACCAGGAGC
GGACATGCCACACGTGGATGGCATGAGCAGACAACCAGTGCTGCCACACGG
AGAAACTTCGCACACAGTAGCAGACAATGTATTAGCGATTGTGCCTACCGAC
GAAGATTGGGTGTATGCAATGCAAAGGCAAGACCCAATACTGGCGCAGATAT
TTGAAGTATTTAGGACTAAGGAGAAGAGTTCACAATGGAGCCAAGTAAAAG
CAGACTACGTTGTTGAAGCGAGCAGACTTCTACGCAAAACGGCCAACGGCA
ACAAATTAGTTGTACCCCAAGCAATCCGCTGGCGTATAACAAAAGCAAACCA
CGATGACATAGGACATTACGGCACGGATAAGACAATTCAACGGATTCAACGG
AACTTTTGGTTCCCACGCATGAGGAGATATGTGAAGTCGTACATATCTTCCTG
CCCAGAGTGCTGCATTAACAAAGTAAAAGGAGGCAAACCTGAAGGGTCACT
GCACGTATACGACACGTTACCTATACCATTCCGTACAATAAATATGGATCACAT
AGGCCCATTCCCAAAGAGCAAAAGTGGAAATCTTCACATTTTGGTTATTGTAT
GCGCATTCACCAAATACACATTTTTGGCAGCAACACGAACGACGAAGACAAC
ACCAGTAATTAAAGCACTTCAGCAGTTGTTTGCAGTATTTGGTCAGCCGAGTC
GCATAATTTCTGATCGCGGAACAGCCTTTACGTCGAAGGAATTTGAAAATTTT
ACAAAAGAGCAGGGTATTCAACATATCAAAACGGCAGTCAGAACACCCAGA
GCTAACGGGCAAGCTGAAAGAATGAATAAAACTTTATTAAATGCTCTAAAGA
CCAGCACAGAAAAGACTAAAGAATGGGACCAGCAGTTATTCAAGATACAGT
GGGGCATTAATTCACACATAAATGCCACCACTAAATTTTCTCCAAACGATTTA
GTCTTCAACTTTAACCCAAGAGACGTGACGAACAACCGTCTAATACAGGCAC
TATCAGACGATTCCATTTCAGAATCGGATATTGAAATGAAAAGACAGCAAGC
AGCAGCAAATATTACAAACGAGCAGAAGAAATGGAAAATTAGGTTCGACGC
AAAACACGCACAACCAACAAAATACGAAGTTAACGATTTAGTCGTCATTACC
TTCGTACCATCGGCTACAGGAGAGTCACATAAATTGGATGCTGCTTATAAAGG
ACCTTATATAGTAACAAAGGTCCTCAACAACGATCGGTATATGGTGGAAGATC
TGCCAGACCACACTGTTACTCAGCGACGCTATTGTAACGTGATGTCAAGCGA
CCATATGAGGCCAATGTGTGCCTTAATTCCAAACCTGGATATAGATGAGCCGA
TATATGAATATAACGACGACGCAGGAATGTCAGGAGAGGCCGGACA

>BEL_LTRBEL#LTR/BEL_LTRBEL

TGTTTGGGAACGAGACACCCTGTATACGCGAACAAGTCACCCTTTATCTTTAT
TTACATTCTTATTTGTCTGCAGCTTCATCGGAGCTTATCAGCGGAATCAATGTA
AGCATCGCACCGCTGTAATTGTCCGCGAGCTTGCCCAGTACTTTTCCAAACTT
CTAACTCCCTTCTAACTGTAACTTGTTTACGTCTTATGCTAGACTAATCGTATG
GCGTGATTACAGCCAAAGCTGAAGTCAGTCACAATTTTGATCTGCGAGAAAA
CGTACGCATCGGTGTCGAAATAATTAATATTAAGTGTCTGAACTTAACCAATA
AATGAAAATTAACAGTAACACTGGCGGTTTTATTTATAAACA

>BEL_IBEL#LTR/BEL_IBEL

TAAAAATTGGTCCTTCGAGCCGGATAACCGGAAGTGCGTTTCGTTCGGGCAT
TTGATTTTGATTATTGGCCTTTTGGCAAACGATAATCTATAGATTCCTACATCGT
GTAGAATCGTTCCCTTCTTTCGACCACCATGCGGAGTGTGATTCAACAACGG
GGCTTCTGCAAAAGCCAAATTACTCGTGCGCATAATAATGCCTTAAAATTTGT
TGATGACATTCACTCAGTGCAAACAATAGTTGTCCGCCTGGCGCAACTACAG
GAAAATTATTTGCGGTTCGTACGGCTCTCGGAAGAGCTGTATGCATTTCAATC
GGAAGCCGATTGGGAGAACCCTGACGAGGATTTTGACGCATATGAGGACAA
ACATTATGCTACACACGCTATTCTCAGCAATACTTTGGAGGAGTTGAGACGGG
ATGTCACCTCAAACAGTATTGATGCCACAGTTCAAGCGCAGGCACACCCCAG
AGAAGTCATGTCGATTTTCAGTTCGAGAGAATTAAACTTCCGACTTTTTCTGG
AAATTATGAGGACTGGAAACATTTTTCGGACATGTTTATTGGATCGATTGCTT
CCAATTCGAGCCTGACGGATTGCCAACGATTTCATTATTTAAAATCGTACCTT
GCCGGAGACGCGCTTGCATTAGTTAAACATATTCCAGTTACTAATGACAACTA
TCGGGAAGCATGGGAGCGGCTGGAACAGCGATATAACAAACAATCGCTAATT
ATTCGATCGTTCTTAAACAGTTTCATGAGCCTTCCGAGTGCTATAAATTCAAAT
ATCGGCACAGTGCGGAAAATTGCCGATGGTGCAGACGAAGTTATTCGTGGTC
TACGAGCTCTTAATTGCGAAGAGAGGGATCCCTGGCTAATTTTCATTCTACTT
TCAAAATTAGATAGCGATACCCGCCAAGCCTGGGCTCAGTGCGCAGAATCCG
AGGAAAAAGGTGTGACCATCAACCGATTCTTGAAATTTCTCACATCACGCTG
CGATACGTTGGAGGCTTTTGAATTAACTCGATCAACCCAAGCTCGACGCGCA
GCTACCACGCACCACGCAGACACGCATCCAAGACGGGAAGAGCCGAAGTGC
ACATCGTGCCAGCAGAATCACCAACTGTTTAAGTGTCCTCAATTCATCGCACT
CGACATTGCATCTCGCCGAGACTTCCTCAAATCAAGAAAGCTCTGTTTCAATT
GCCTCAGCCCGGCTCATATGGTGGGCAACTGTACATCGAGGCATACTTGTCGG
ATCTGCCGCCGCAAGCATCATACTTTGGTTCATGGCTCGTCGCAGCCAATTCA
AAATGGCAACAACATTGACACAGCAAGTGTTGACAGCCGCGATCGACCAGC
AGTCTCACATGCGGGATCTACAATTGGCCACAATCAACCGCTAGCTCGAGAA
GGTCATCGCTTGGGAAGCGAGACTCCCGCGGAAAACAACTTTACGCATCATA
CTCTGGAGAATATTCCGGCGGCTGGTTCTCAGACTCTGTTGCCAACCATCCTT
GCTGACGTCATCGACGCCTGGGGAAACACTACAACCTGCAGGCTGCTCCTGG
ACACTGGATCTACAATAACCTTGGCATCGGAATCATTTGTTCAGCGAATAGGC
GTGCGTCGAACGCACGCACGGATTTCTATTCTCGGTCTCGCCGCCAACAGCG
CGGGCGTTACCCGAGGACGCGCACATATCAAGCTGCGCTCTCGTCATTCGGG
CCAAACTGTCGAATTGGTCTCGTTCATTCTCACCTCGCTGACGTCATCACTTC
CTGCCCAAGTTATTGACACCTCATCCTCTACGTGGAGGCAAATCTGCGAGCTT
CCTTTGGCAGACCCAACGTTCTGCACACCTGGAGCAATCGATGTCATTGTTG
GATCGGATCAACTTTGGTCTCTATACACAGGAGATCGGAAACACTTTGGTAAC
GACTTTCCTATCGCTCTCAATACTGTATTTGGTTGGATTCTTGCAGGCTCTTAC
TCTGCATTCGATGATCACCCTACTTCTGCGGTTACTCATCACGCGGACCTAGA
CACGATGGTTCGTTCATTCATGGAGATGGACAGCATTCAGCCTAACCAGGCTC
TCCTGGACGCCAGCGATCCCACAGAGCGTCATTTTGCTGCCACACACAAGCG
CTCGACGGACGGGGTGTACGTCGTCGAGTATCCCTTCAAGGAAAAGGCACC

GCCTATTGATTCGACCTTGCCACAGGCCATCAATCGCTTCTTCTCGCTGGAAC
GCAAATTTCGTCGGTATCCAGAATTGAAGCAGCAGTACGAAGCTTTCCTGGA
CGACTACTTGCAACGTGGACATATGGAAAAACTGACCTCGGCTCAGGTTGAA
GAGTCCCCAGACACCTGCTTCTATTTGCCGCACCACGCTGTCATCAAACTGG
ACAGTCTGACTACCAAATGTCGTGTAGTTTTTGATGGATCAGGAAAAGACAG
CTCTGGAGTATCGCTCAATGACAGACTACATATTGGTCCACCGATTCAACGCG
ATCTTTTTGGCGTTTGTCTACGCTTCCGGCAGCACCAATATGTTTTATGTGCAG
ATGTCGAAAAGATGTTTCGAGGCATTAAAGTCTTTAAGCCACACACCAATTTT
CAGCGCATTGTTTGGCGCACGACTGAGAATGAACCTCTGCTTCATTTTCGCCT
GCTGACGGTTACCTACGGATTGGCACCGTCACCATTTCTGGCTGTTCGAGTTC
TAAAGCAACTTGCCGACGATCATGGCCATGAATACCCTGCAGCAGCTCACGC
TCTTCTGCACGATGCCTATGTGGACGATATCCCGACAGGCGCCAACACATTCG
AGGAGCTTATGATTCTCAAGGACGAGCTTATAGCCCTCTTGGATAAGGGAAA
ATTCAAGCTACGCAAATGGAGTTCTAATAGTTGGCGTCTTCTGAAATCATTAC
CAGAGGAAGATAGATGTTTTGAACCTATCCAGCTCCTCAACAAATCAGCTGC
GGATTCACCTGTCAAAGTTCTTGGTATCCAATGGAACCCTGGGAAGGACGTC
CTGTATCTCAACCTAAAGGGATGCGATGCGACCATTTCTCCGACGAAAAGAG
AACTCTTGTCTCAGCTATCAAGAATTTATGATCCGCTTGGACTGGTAGCGCCG
GTCACAGTTCTACTCAAGCTAATCTTCCAAGAAAGCTGGACAAGTGTCCTGC
AGTGGGACGACCCCATACCTGAAAGTCTACGTACGCGCTGGAGAGCCTTAGT
AGAGGATTTGCCAGCACTTACGCAATGCCAAGTACCACGGTATATTGCGTCAC
CATTTCGAGATGTTCAACTACACGGATTCGCCGACGCATCCTCGCACGCCTAC
GGTGCGGTAGTTTACGCTCGAGTTGCAGTTGGATGCAGCTTTCAAGTAACTC
TGGTTGCCGCCAAAACACGGGTGGCCCCGATCAAGCCCGTATCAATTCCACG
TTTGGAGCTAAACGCTGCGTTACTTCTATCTCGATTGCTTTCTATTGTCAAAAC
ATCACTAACAATTCCTCTTTTCAGCACGAGCTGCTGGACAGATTCAGAAATTG
TGCTACACTGGCTTTCAGCTCCCCCTCGACGGTGGAACACCTACGTCTGCAA
CCGAACTTCTGAGATATTGAGCGACTTTCCCCGTAGCTGCTGGAACCATGTTC
GCACGGAAGACAATCCTGCAGATTGTGCTTCCCGAGGACTTCATCCGTCAAA
GCTTCTGGAGCATCGACTGTGGTGGAAAGGTCCGTCTTGGCTGGCCACACCC
ACCTCTGAGTGGCCACCTTCTACAAGCAAGTTCAGCGTATCTTCAAGTTTCG
ATGTCAACACCGAAGAACGAGCCATAAAGCCCACGACTCTACATAACTTTCC
TGATGAAAGTATACACGAGTTACTCATCCACAAATTCTCAACCTGGACGCGTC
TTATAAGGGTATCTAGCTACTGTCATCGCTTTATTCACACTCTTCGATCCCATC
ATAGGAATTCGGCACCATTCCTTACGTCTGAAGAGTTGCTGGACGCACAGCG
CCGACTTATTCGACATGTGCAACAAAAATCCTTTGCCAGAGAATATGAGCAG
CTAGAGAATCGACGCCAGCTTAACGCTAAATCGCATCTTATCCGGTTTTCTCC
GTTTCTGGATGATTATGGAGTAATGCGAGTCGGTGGGAGAATCGAGCAATCTA
CACTCAACTATAACGCCAAGCACCCGATTCTGATACCTAAAGATACACCACTA
GCTGGACTCCTGGTTCGACATTTTCATGTCTCCTATCTGCACACTGGAGTTGA
TGCAACGTTCACCAATCTTCGTCAGCAGTACTGGATTCTGGGAGCCCGCAAT
CTCGTCAGAAAGGCAGTCTTCCAATGCAAATCCTGTTTTCTTCAACGAAAGG
GCACAAGCAACCAGATCATGGGAGAGCTACCAATTCCTCGAGTTCAAGCTAG

CCGCTGCTTTCAACACACAGGGCTGGACTACGCTGGACCGATCGCAATCAAG
GAATCAAAGGGAAGAACTCCACGCATCGGAAAGGCATGGTTTTCTATTTTCG
TGTGTCTCACTACAAAGGCACTTCACATCGAGGTTGTTAGTGAGCTAACTAC
ACAGGCTTTCATCGCAGCCTTTCAACGATTCATTGCCCGCCGAGCGAAGCCT
ACTGACCTGTATTCGGATAATGGAACAACATTTCATGGAGGCAAGAAAACTT
TGGATGACATGAGACGTCTGGCCATTCAACAAGCCAAAGATGAGGAACTAGC
AGGATTCTTTGCCAATGAAGGGATTTCTTGGCACTTTATACCCCCGTCTGCTC
CACATTTTGGAGGGATGTGGGAAGCTGGAGTTCGCTCAATTAAACTCCATATG
AAACGAATACTTGGATCAAAGGCTTTAACGTTTGAGGAGCTCTCTACTGTCCT
GACCCAAATTGAAGCTATCCTGAATTCACGCCCGCTGTGCCCAACTGGGGAT
AATTCTTTGGATCCACTGACGCCTGCTCATTTTTTGACTGGATCTCCGTATACT
GCATTGCCTGAACCCTGTCGTCTGGATATGCAAGTCAATCGATTGGAGAGGT
GGAATCAGCTGCAAGCCATGGTTCAAGGCTTTTGGAAAAGGTGGCATATGGA
ATACCTGACATCTCTTCATGAGCGGACAAAGTGGCATCTGGAAACCGAGAAT
CTGAAGATCGACACACTGGTAGTACTCAAGGAGCCCAATCTACCGCCCTCTA
AATGGATTCTTGGCCGCATCACAGCAGTGCACGCAGGAATCGACAACAAGGT
CCGAGTCGTTACAGTGAAGACTGCTCACGGATTATACAAACGCCCAATTGCC
AAAATCGCTGTACTGCCTCTCTGCTGAACAACCGTTCAGGGGGGCCGGTA

>BATUMI_LTRBEL#LTR/BATUMI_LTRBEL

TGTTCAAGTTACGCTCACCCGCTGTCACCCGCTGTCACCCGCTCTCCGCTCCC
TCTTACGCTCTCCCGCTCTTCACCTCAGAGTCTCCAAGGAGTCCTCGGGCTTG
GGATAGCCTAACTAATTAGAATAAGCATCAGTGTAAAAACTAACCACGCTGA
ATAAACATACGCCCGGTCGCCGCGCAATTACGAAAAGTCTAGTGTTTGCTTTC
CTTCGAGTGTTTCTTTTCAGCATATTTGAATTCAGGACAGCCATCCCCCTACAT
CCCAACA

>BATUMI_IBEL#LTR/BATUMI_IBEL

TTTTGGTCCTTCGAGCCGGATCACCTGGATTTTCAAGTTTGTCCACCAGCGAA
CAAATTATAAGATAAGTACGAAATTTCCATTCCTTTTAATTGCCGGTCTGCAGC
AAAAGGTTCGAAAATCCAATTTCGTTCAATTTGCTGTAAGATTTATTGTCAAA
TCTAACGGATTTCTCCGACAAAAGGCAATTAAAGAAAAGTACTTATCCAATCT
CACGGGCGCCGCATATTACTCGCCGTTCTCCGTTCTCCTTTCACCCTCATTCGT
GAAAATTTCTAAAGTCCAAATGGGCGAATATATTTAAATATTAATCCAGTGCG
ATAATGCAAAATTCCAAATGTGAAAAAGTGAATAATTTGTGCCAAGTTCAGT
GAAACTTTCTAAGTCCAAAGCTCTGCCAAAATTGGCAAAAATTCTGTTCTCG
TTTCACTGTGTCAAAGCGAAGCCAAACTTCTTTTCGCAACACATTTTTGCTTT
AACTCCGCAGTCCACTTAATACCATTTGCTTTGCTATCGAAGAATACCACAAC
GAAACAAACAACACCATACCCTCTGGCCATTCAAATAACATATTAATTAACAT
TTCCGCAGTTCCATATCTCTTACATCAACATATACCTACTCCATACTCTTGCATA
TATTCACATCTTACACAATATATCCTCACCATATATTACATATATTACATCAACAT
ATAATATCCACATATATTACCGACATACATTGCGCATATTATCAGCATTCCTTTA
ACGTATACCAAAGTTTAAATTCGATCCCGTCGGCAAATCCAACCACAAATAAA

ATTTATTCCAAGTGCCGACGCGGAAAGGCGTTTTCTTTTCCATCAATTTTTTCC
GTAAATTTCCAAATTAATTTCCGAGCAATAAATTAAAAGCGGTTTTTTCTTTTT
TTTTAACAAATAACTTATTGTTGAAAACATTTATTAAATTATTAAAAATTATATA
AATAATACGACCGCCAAATACAAGTCGTTCACCCGACAAATATTTTTTCCTGT
ATTGCTTGGATATTAATTTGTGTTTGTTTTAGAAGTACTTACAACGCGGAGAA
AAGACTCCAAATCCACCATTCCATTTTCTCCGTTTCCAGTTATAAACAAAAAA
ATAAATAAAATTTTCTTCCTTCTAATAAACATTTTATTTTACCGTGTTCACATTC
CAAGTGTTCCAACCGTAAATAAGGTGGACCTAATTACCATAAATCACAGGTCA
TTTATACAATTCGCTGTTCACTCCGAGTCACCTGTCCAATTAGTCTAAACTACG
GCGTTTCCACTTCGCAAATTCAACACCACTTTCTCACCCATTACATCCTATACG
GTCCTTTTCCGCTGCTTTATACCGTTCACGGCAGGAAGCTTAAATTTATTAAGT
GGAATCTGTCTACTTTTTCAAAAGTGTGACCGGGCTCCAAAAACGCTTCCCT
TCCATTTCGTATTTCTTCGATTATGCCCATCGGGGACGATAAGAAGAAATTGTC
CGCTGACAAACCCAGGTCTATTTTTTCACCACAAGGGCCCAAGAGTCCAAGA
ATCCCAAGCATTTCGGTGAAAACGCCTGCGCAGATTTCCGACGACTGTGCCA
CTCCATCCAAAGCCACAGTACAGCGCACAGCTAAAAATATGGCTGCTTCCGA
TCTAGCGCTAGCCAAATTCATTTCGGTTTCTGACGCTCAAGCGAATTTGAGGC
TCAGATCAACACTCCGGAATCCGCAGCTCCAACCGTCACGATGCTTAGCGTC
CGTCGCGACCAAGTCCGAAGCCTATGGGACAAGGTTGAAAAGAATTCGACC
TCTGCTCAGAGTGCCTTGTGTCAGCAGGCGAGCGGCAGCAAGCGGCATGCC
TATTCTCAGGGCTAGTTACAGTTATTGCTATTCAGTCTATGAAAGGTGTGTTGC
CCAGCTCGTTGATAAAATCGAGCAGGGGCACTTCTCAGTCCATCCCAAGCGA
ACGCTGCGGCCCAGGCCTACATTTCCTCTGGCTGTCGGTTGCCTCCATGCGAT
ACAGGAGTTTTCGCAGGTGACTATCTTCGCTGGCCGCTTTCCGGATCTTTTAC
ACGCCATTTATATTAATAATCCACGGCTGACTCCGTTCGAAAAGTTATTCCACT
TAAATGCCAAAACAAGTGGCGACGCGCATGCCATAGTTTCGATTTCGCCTCTC
ACCAAACGAGGGTTTTCCTCTGCGTGGGAAAACCTAATAGAGCGTTTCGAAA
ATAAACGATTGTTGGTAAACAGTCAATTGAAAATACTGTTTAATGTGCAGTCG
ATACCACAGGAATCTGGGGCGGCCTTGAAGGTAATGCAAAGTACTGTTCAAG
GTTGCTTGACTGCCTTAGAACTGTCAGGCATCAACACTGAGAACTGGGACTG
CCTGCTGGAATATCTGTGTTCATCCAAGCTCCCGAAGATAACTCTCTCCTTATG
GGAGCAGTCTCTACATAAGAAAGCCGACATCCCGACATGGGGAGAACTGAA
CACCTTCCTCACAGAACGTCATCGAACCCTAGAGGCCATCGATGATGTGAGA
CCGTCCGTACCAAGTCAGTCGCACTCCAAAGCGATGAACTCAAGTGGGCCCT
CTAGAGATGGCAAGCTGGCGTCCGACTTGTGCAACAAGGAAAACCATCCTGT
CCGTGTATGTCGCGTTTTCTCCAAATGGTCGGTTGACGACCGGTCAGCCTACA
TTAAACGGAAGCAGTTATGCTTAAACTACTTTGCAAAGGGACATCAGCTTCG
TGAGTGCAAAGATCGACAAAGTTTTACTTGGTGGCCGGCATCACACGTTGTT
GCACCGAAACAACCTCTTTTCCAGCAATTCAAGCCCTTCAAATCCTGCAAGC
CCAATTTCCGCTACTCAGGCCAATTTCGTTCCAAACGAGCAAGCCGGTGTTC
AAAATTATTTTGCCACGGCTCAAGAGCTATCCTTCTTGGCAGTGCCATAATCA
ATAGTTCCCATCTTGGCACTAACTTTAAGGCACGCGCCCTGATCGACTCCGGA
TCAGAGGCGACATTCATAACCGAGCCACTGTTCAATCTAATTAGATTGCCATT

CCAGGTGGTTCAAGCCCAAGTCTCGGGCTTAAACCAAACAGTAGCTGCTCA
GTTCAAGAACGCTGCAGTTTCACCATCCGATCTCCGACTAGGCCGCGTTGCA
GTTGGAGACGACGGCCTATGTCCTCCCTCAACTAGCCGGAAATCTGCCTTCC
TACCCAATTCCGCAAAATTTCTTCGGGATCTTCCCGATTTTCCACTGGCGGAT
CCAAAATTCTATGAGAGCGCCCCAATAGATGTACTTATCCGGAGCCCACATCC
TGCTTCGGTGCTTCTGAGTGGAGCAAAAACCAACATCTGTGGCTCTCTCTTG
GGGCAAGAGACCATTTTCCGCTGGGTACTAACTGGGCCAGTGTCAGCCTCAG
CCCAAAGCAGGATTCCTCTTTTTCGACACAGATCTCCCACGCGTACGATAATT
CACTGGACAAACTCCTCACAAAATTTGGGGAGGTGGAGGATATACCAACAA
AGTTGCAAAAGAATCCGATTCCATGTGCGAGAACGGGTTGGTAAATGCTTAC
GACGACACCAGTGCGGCAAATATGTCGTTACTCTGCCTTTTCGCGACCCAGA
ACATATCGGTTGCGGGCTAGGGCATTCTAGGTCTTGGGCGTTGGCTCAGTTCT
TGAAGAATGAGCAGCGTCTAAAAAAAGATGAGGCCTTGAAAGCGAGATACG
ATTCGGTGATCCAGGAATATCTCGACTTAAAGCACATGCGACAAGTTCTGCCT
ACCCATGATTGCAACGCCTATTATATGCCACATCACGCCGTCTTAAAACCGGA
GAGTGTAACTACTAAACTCCGTGTAGTATTCAATGCCTCCAGCCCTTCATCGA
ATGGTACCAGTTTAAATGATATCCTTCATGCTGGCCCTGTCTTGCAGTCCGACT
TGACAGTGCAAATTCTGAAGTGGCGCGATTTCCGATACGTGTTCAGTGCCGA
TATTCAAAAAATGTATCGGCAGATCTGGGTAGATCCGAAACACACTCCATTCC
AGCGAATACTTTTCCGTAACAATAGAGGGGAAATCAGAGATTTCGAATTGAA
AACAGTAACCTTTGGAGTCAATTGCGCGCCCTTGCTGGCGATCCGAGTACTG
CAGCAGCTAGCAGCTGACGAAGAACTCAGCCATCCAAAAGCTAGCAATGTC
ATTCGAAATTTCATGTATGTGGATGATGTTTTAGCCGGAGCGGACTCTACGGA
AGAAGCTCAGCTCATGGTGCACGAGCTCCGAGACGCTCTGAATTCTTCTTCG
TCCCGCCAGAGATGGCTATCGAAACGTCCTTTACAACGCCAAGTCCTGTCCC
AAATTGCCAAATTGTTCGACCCTGCAGGCTGGTTAGCACCGTTTATCGTTCGA
GCTAAAATTTTCATGCAGGAGATTTGGCTACAGGAGCTTGGGTGGGACGAAA
ACGTTCCAAATGACCTTTTTCAGCGATGGCTTAATTTTCTCCAAAGTTATTCG
GTTTTCGAGCAGATACGCATTCCACGCTGGCTATCGTTTCATCCAGATTTCAA
GGTCGAGCATCATGGCTTTTGCGATGCATCGCAAAAGGCTTATGGCGCCGCA
ATATATGTCCGCGGAGAAGTGGGCAGCGCCATTATGGTGCAACTCCTAACCGC
CAAAACCCGGGTAGCACCAGTCAAAACGGTTTCGCTCCCAAGACTCGAGCT
CTGCGGAGCGTTATTGCTTTCCGAAATGGCTGCAGCCATCATTCCGCAGATGC
CTACGATTAACTCCAAACTTTACTGTTGGACGGACTCCACCATAGTGCTTGCA
TGGTTAAGCAAGCCAGCATGCCAGTGGACCACATTTGTAGCCAATAGGGAGA
CGAAGATCGCCCAGGCCACAAAAACAGAGAATTGGTCTCATGTTCAATCTGA
GCATAATCCAGCAGACCTGGCAAGTAGAGGAGTTTCCCTCCAAGATCTAGCC
GATAGCCAGTTATGGTGGCACGGACCGACTTGGTTGCAAAATCCACGCAACC
AATGGCCTACTCAGGTCAACGCTCCGGTGACCGACCTGGAGAAGCGTGCTCT
AAAAGTCCATCTCGCGAAAGCTCCTTCTGAAGAGTTGTTGGCACGTTTCTCC
AAGCTAGAGAAAGCTCTACGAGTCCTTGCCTATGTTTATCGCTTCATTCAGCG
GTGCAGGAAGCAGACATCTCCATCTGATGTTCATCTACTGGCCACTGAAATCG
CCGCCGCCGAGCGGTTCCTAATTTCGAACACTCAACGCAGAGAATTCCCTGT

GGAATATCACTGCCTAAGTGAAAAGCGTCCAGTGCCAAGTTCAAGTGCCATC
CTAAGCATGAACCCGTTTCTAGATCCGCAAGGACTGATCAGGGCATGCGGCC
GTGTGGCGGCTTCCGAAAGCCCTCAATACAATGAACGCCATCCAGTGATTCT
TCCGTATAACTGCCTGCTTTCTCGCCTCCTTGCGAAGTTCACGCATCGCACAA
CTCTCCATGGTGGTAACCAGTTAATGGTGCGCCTCATCCGGTCGAAATACTGG
ATTCCGAGAATCAAGAACCTGATGAAAGCAGTGGTAAATTCGTGCAAAGTAT
GTGTGATCCACAAAAGGCGGTTGCAAAGCCAACTGATGGGTGTCCTGCCCA
AAGAAAGAGCATCGTTCTCCCGACCATTCACGGTATCGGCATGGATTACGCC
GGTCCGCGATATAAAGAACTATACGGGAAGAGCATGTGTTATTACAAAGGGG
TATGTGTTAGTTTTTGTTTGTTTCTCCACCAAGGCCATCCACTTAGAGCCTACA
TCTGACTTAACGACCGAGAAGTTTCTTGCCGCTTTCTCTCGTTTTGTATCCAG
GAGAGGGTGTCCACGTCAAGTCCAGTCAGACAATGGCAAAACCTTTGTTGG
CGCTGCCACCCTGCTTTCCCGCGATTTCCTTCAAGCCGTAAAAGAGTCGGTG
ACGAATGCCTATATTCATCAAGAGATGCAATGGCAATTATTCTCCGGGGGCAC
CCAATATGGGAGGCCTTTGGGAAGCAGGCGTAAAAAGCTTCAAGACGCTATT
TTACAAATGCACGGCCACACGAAAATACACGTTCGAAGAACTCTCCACGCTC
TTGGCAAAAATAGAAGCGTGCCTTAACTCCAGGCCGCTCTCTCCTATGTCTGA
AGATCCGACAGACTTGCTGGCTCTGACGCCAGGGCATTTCCTTGTCGGGGGA
CCCCTTATGTCCACGGTGGAACCCGAAGTAAAGGGGGAAACGAAATCCCTTC
TTAATCGGTGGCAGCATTTGAAGGCTCTCCATCAGCAGTTCCGTGTGCGATGG
AAAGAAGAGTACCTCAAAGAACTCCACAAGCGTTCTAAATGGCAGGTCCCG
TGAACTTCGAGCTAAAATACTCGTGCATGTGGAGCAGCGTGTGGTGGGATCG
GTCGCACTTCTTGCAACGATCACCGCTTCGGCAGTCTCCCGTGGAATGCTCG
TGAGCGAGGCAATTGGCGCTAGTATGTTGGTAATGAGGACTGCTCGCAAACG
CTTTTCAGCGCTGAGCTTTAGGAACCTCGCGCACGTCCGAAGAGGATGGATT
ACCGCGGCAGACTCGGCATCGGTAGGATTTAATACCTCGGGTACGTCTGCTCT
CCACGGCACGACCTGCGTGCGTTTTGTTGACGAGGAGCCATGTGCGCGTAGT
CGAATGTCGAAAGGAGATCGAAAACGAAATGAAAAATAACGGATGATTAGT
GATAGTGAACTACAACTAAGGACGAGAGGAGAGACCTATTATTGTGGAGATT
CGGAACTCCGTCGGCAAAAGCACCTTTTTTGCCACTGGACGTTTAATAACTC
CACGTGCAGTACGGATGTTTACTACACGACGTTGCCGTCAGCTCCTGGGAAA
ACAGACTCAATTCTGCCGAGCCGCCACTCATTAGAGGGCAAGTTGTCGTCCT
TGATGACGACTGTTGAGTTACGGGGTTTGGACTGTCACCGTCTCCGCTCCCT
CTTACGTTCTCCACTCCCTCTTACGCTCTCCCGCTCTTCACCACAGAGTCTCC
GAGGAGTCTCTGGCGCTTGGGAGAACCCAACGCATTAGAATAAGTTTTAGTG
TAAAACTAACCACGATCAATAAAACATACGCCCGGTGCCCGCGCTAATTCTAC
AAGTCTTCGAGTGTTTTTTCGAGTGGTCTTTTTTTCAGCAAACTAGGAACTTT
CCAGGACCAGCACCCCCCATCACCCCAACAACGACCATGTCATCGATGGCGT
CGCCAACCGGCGCCAACCCTGCAAGCAGCTTCGGCCTCGTCCCAACGTTCCC
GCCGGCACAATCCGCCAACTCAGCGTAGGAGTTCACCGCCACGACGACCGG
AATCGACGACGCCAGGCCCATCACTCTCGTCGCCGCTGCAACGCCACAGCGT
GAACATCCTTCCCACAGCGCTGGTCAAGATGGAGACCGGGACGAAGACCTT
CAGACCGCAGCACTCATCGATCCGTGCAGCCCCATGAGCTGCATCGACGCTT

CGTTGGCGTCAGCCTTTAAGCTTTCGATGACCAATGTTGGCGACGAGAAGGT
CTGCACGACGACGATTCGCTCCAGGATCGACGCGAACACGAAGCTCGAGGT
CGTGCTCAAGATCGAGCCCAGGGTGCGGATCCGTACACCTGTCCGGGCATTG
AGCGACACCGTAGTGTCCAAGTACAGGGACATCATGCTGGCGGATGACGGGT
TCCATCGGCCTGCTACCGTATCCATGGTCTTAGGAGCAGACATTTATCCTAAG
GTTATCCAATCCGGATTCCTGACCTTCGACGAGGGAATGCCGGTCGCTCAAA
AGACCGTGTTTGGGTGGATCGTGTCCGGTGCCTGCAGCTTGCCTAGATGGCT
ATGTTGCAACCCCAGTGATTGCAAGGGGGGCGGAA

>BARI_Mariner-Tc1#DNA/BARI_Mariner-Tc1

CAGTCATGGTCAAAATTATTTCACAAAGTGCATTTTTGTGCATGGGTCACAA
ACAGTTGCTTGTGCAGCAAGTGGGGGGAGGTGAAATGCAAAAAAACTTTTG
CTTTTGCAAATTCAAACCTATGCAGAGTCAGATGAAAGAAGAATTGAAAAAA
TAACTGTTCCTATGCGCAAGGAAGAGGCAAATGAAGAGATCTTTATCAGTTG
TCAGAAGTATTTGCACACGGTTTCGTCGCATCACAATTATTTTCACAACGCAA
TTTCTTCTTCAGTGATTGGTTTAGAGTGACAAGTGCCGGTTTGTTTGCTTAAA
TACATTTAAATTATTGAATAAAAATTAGATTTAATCATTTTCCTATTACAGTTAT
TAAATAAAATGCCCAAAACAAAAGAGTTAACAGTTGAGGCCCGGGCTGGTAT
TGTTGCTAGGTTTAAAGCCGGTACACCTGCGGCCAAAATAGCTGAAATATATC
AAATTTCGCGTAGAACTGTCTACTACTTAATAAAAAAGTTTGATACAGTTGGC
ACATTAAAAAATAAAAAAAGATCAGGCCGAAAACCTGTGCTGGACCAAAGG
CAATGCAGGCAAATACTTGGAGTTGTGGCGAAGAATCCTAGTGCCAGTCCGG
TAAAAATTGCCTTAGAATCAAAAAATACAATTGGCAAACAAGTTAGTAGTTCT
ACAATTCGTCGCAGGCTAAAAGAAGCTGATTTAAGACATACGTTGTTCGCA
AAACGATTGAGATCACACCAACCAACAAAACAAAACGTCTTCGATTTGCGTT
GGAATATGTTAAGAAGCCTCTTGACTTTTGGTTTAATATTTTATGGACTGATGA
GTCTGCATTTCAGTACCAGGGGTCATACAGCAAGCATTTTATGCATTTGAAAA
ATAATCAAAAGCATTTGGCAGCCCAGCCAACCAATAGATTTGGTGGGGGCAC
AGTCATGTTTTGGGGATGTCTTTCCTATTATGGATTCGGAGACTTGGTACCGAT
AGAAGGAACTTTAAATCAGAACGGATACCTTCTTATCTTAAACAACCATGCTT
TTACGTCTGGAAATAGACTTTTTCCAACTACTGAATGGATTCTTCAGCAGGAC
AATGCTCCATGCCATAAGGGTAGGATACCAACAAAATTTTTAAACGACCTTAA
TCTGGCGGTTCTTCCGTGGCCCCCCCAAAGCCCAGACCTTAATATCATTGAAA
ACGTTTGGGCTTTTATTAAAAACCAACGAACTATTGATAAAAATAGAAAACG
AGAGGGAGCCATCATTGAAATAGCGGAGATTTGGTCCAAATTGACATTAGAA
TTTGCACAAACTTTGGTAAGGTCAATACCAAAAAGACTTCAAGCAGTTATTG
ATGCCAAAGGTGGTGTTACAAAATATTAGTATTGTATTTATATAAAATAAAGAA
ATTCTTATGTTGAAATTAGATGTTAAGCTGAAATTTACTAAATTAAGTTGAGTG
AAAATACTTTTGAAGCGCAATAAACATGTGAAAATACTATTGACAACTTGCAT
GCATATTTTCTTTTGCTTTAAGCTTTGTACTATGAACCGTTATCTTTCGTATTTC
TTTTCGACTACCTTCTGCATAGATCAAGCTAAGCGATAAGAACTATTTCAGGC
AAATCGGACAACAACAAGAAGAAATATAACAAAAAGAAGTTGAAGTTTGCA
AATATTGTGCGTTGTGAAAATACTTTTGACCACCTCTG

>BAGGINS1Loa#Non-LTR/BAGGINS1Loa

GGCCGCCACGCCAGTAGGTATCACAAGGGGATCAACGCGCCACCAACGGTG
GTACGCGCCGTAATTGTGACACACTACTGGAAGTGCAACTACACTCTCCACG
CGAGGGCGGCTGGAAACAGGCTCTTAGTTAGGTCATGTCTCTGCTAAGAGTG
CTGGCTAATCGTAGTTGGGCTGGAGCTGGTAACTCTCAAAAATTTCCACATCT
CCGGGCCGGACTAAGGTGTCATTCGACCCGTGCCGAGAGTCTGCCAGGCTG
GGGGCCCGGGAAACAACTAGCCCAGTCGCTAACGGAGAGTCCGGGCAAGTG
GCGTCCGCCCGGTCTGGTTAGCCTTACCCGGGTACTGCGGATCTCTGCCCGG
GTGGACCGTTTATTTCTCTGCAACTCGTGGGAACCATGAACAATAGACCCAA
AAATATTAAAATACCGCAGGCCTTGACTGCGGGAAATGTCGCAAGACAGTTT
GAGGACCGTCTCCTTGACGGAGAGCGGCCTACTTCGGTGGTAACACCAAAA
AATGCTCCTAATCGGAGTGTGCCAACGGCACAAGCACGCCCAGTTGCGGGA
GGGAAGAATCGGGCAGCCACACCGAGTCACTCGACTGTGGTCCAAGCCGGC
CAAGCGGGACGGGCTAAAAACCCATTACCGCAACTTGGTCAGCCTGGGGCC
CGGGATGACCCTAACCGTAAGGGGCTGAGCGGGGCCAGAACGAAGTGGTAT
CTTCGTTACCTTCAGCAGGGCAAGTCACCCGCAGAAGCTTTGTCTCTGGCCA
AGACACCGCAGCCGCTAGAGCAAAGGCCCAACTCTGCTTCGAAGCGGGCAA
ACTCCACCCTGACGCCTCCGACGGAAACGCCTAAGAGGCAGAAGGTGGAGA
AGAGCAGGCCACTGACTGCTCCTTTCGATGGCCCAAGTACCTCAAGGGCTGC
AAAGGCAACTGAGGTACGCAAGCCATCATATGCGGCCGTAACTGGGGCCATT
AAAGTTGCGGTCGTACCAGAGGGGTACCCCAAAGTCGTCCTCAGCAGTGAG
AACCTCTCACAGCTCGAGGACGCACTACTGGAAGAGATAGTCCTCTCCGGTT
GGGATTCTCCAATTAAGTTTGGAGGAATCCACTTCAGGGTGGGTCACCTGAT
AGTGGATTGCCGCAACGCAACCACTGCTGAGTGGCTGCAATTAGCAGTCCCC
AGCCTCAGTAAGTGGAGCGGTGTCTCCCTGGAGGTAAAGATGGGCGACGAC
TTGCCATCGTCACACAACATCACCATCTTCTGCCCCAGGACAGGAGACAAGA
CAACAAAGTGGATCATGGAATTGATCAAAAAACAAAATGACTTGGACACTG
AGAACTGGCGTCTTATATCAAGGAAGAATGAGGGTGGTGGCTCGCTCCTCAG
CCTTGGCATTGATGACAACTCATGTGCCAAGATTATTTCTAGCGACCACAAGC
TGAGCTTCAGGTTTGGAGACATCTCAGTTTGTGGTCTAAAGAAGGCCAAGGC
GATCAAAGCCGGCACAAGACGGGTCGAGACTCCTCGAAATCCACCAGTCTC
CGCGGAGAAGGAGAAGCCCAATAAGCTCTCCGAATCCAGCGAGGATCTGGC
GGATGATGAGGATCTCGATGCGACCGTCATCGAGATGGGGGATCAGACCCCG
ATATCCTCTCAGGAGCTAGGCATGGAACTAGACCTGCTGAGTAAGGAAGAAG
CGGTACCGGCGGATGGTGATCTGGGCATCTCCAGATCAGCAACGCCGACGAT
GGAGCCGATCATCTAATGGTCGGGAGTACAGGGATAGCTCAGGTGAACATCC
ATCGCGCCAAGGCGGCCTCGGCCGTGCTGGCAAGGATGTTCACCAAGCAAC
ATCTTGGGCTAGCCCTGGTACAGGAACCCTGGTATAACCAGGGAATAAAAGG
ACTGTACGTGAAGAACGCCAAGGTAATTTGGGATCAACGGGCTTCTAGCCCT
AGAGCCTGTATCCTGGCATGTAGAAGTATTAATTATTATATACTTACAGAATTT
CTCACGCGGGACTGTGTACCGATCGTGGTAGAAGATGTGGGAGCAGCCAAG
AAGACTGTGGTGGCATCGGCATACTTTGCCGGTGACGAGGCGTGCCCACCGC
CCGAAATCACTGCACTGGCTGAACACTGTAAGAGGATGCACTCACCCATCAT

CATTGGATGTGATGCAAATGCACATCACGTGATATGGGGCAGCAGCGACGTG
AACCAAAGAGGTGAGTCACTCTTAGAATTTATCTTGAATAACAATCTAGAAAT
CCTAAATGTCGGCAATGTTCCGACATTTGTTACTAGGGTAAGATCTGAGGTTC
TGGACATTACACTTTCCAGCAGATCAGTGGCTTCGCATATAAGCGACTGGCAC
GTATCTCCTGAGGAGTCAATGTCAGATCACAGAATTATTCGTTTTAACATAGG
GCTAAATCTAGAATGTAGCGAGCGTAAGCGCAATCCCAGGAATACGAACTGG
GAAGGGTTCAATGCCTCCTTACTGCGAGATCCAGTTACTAGGGTAACCGGGA
AACTCCGTACCACTCTAGAACTGGAGGCCGCCGTAGAAGATATTAACTCGTG
TCTTACTAACGCGTTCAGAGAAAACTGCTCCCTTGGCCGAGCCAAGAGGGAT
AAAGATGCTCCATGGTGGAACGACCAATTGGAGAAGTTACGGAAAGCGACC
AGGCGTCTCTTTAACAAAGCAAAAAGAGAAGGGAACTGGGATGCCTACCGG
AATAAACTGACCATGTATAACCATGAGATCAGGAAAGCCAAACGAAGGAATT
ATAGAACCTTCTGTGAGGCAATAGTAAACACATGTGAAGGCGCTAGACTTCA
CAGAGCAATCTCCAAAGGAGTGCCAGAATCAAATCAGGCACTACGAAAGGA
GGATAACTCCTATACTATAGGAAGTAAGGAGAAACTTGAGCTACTACTGGCA
ACTCACTTTCCGGGAAGCACGCTTCAAACGGATGGGAATACCCCGGTAACGG
CACAAAGTAGACCGCGTGCTACTGATTGGGCCAAAGCAAAATCAATAGTAAC
AACGGAGAGATTAAGATGGGCAATTGGTACATTCCAGCCCTACAAATCTCCT
GGTCCAGATGGCATTCAGCCAATCCTGCTTCAACGAGCGCTAAGTCAGCTCG
CAGTACCGATCAGGAAAATACTGATCAGCAGCCTAGCTTTAGGACACATACC
TACTGCCTGGAGTATAGCAAAAGTGGTCTTCATTCCGAAAGCTGGAAAGAAA
GACATCACTGATCCGAAGTCGTTCAGACCCATCAGCTTGACATCGTTTTTGCT
AAAAACGTTGGAAAAGCTAGTGGATGTCAGCATTAGAAGCACTCTGCTTGTG
GAGCATCCGCTCCAACGGACGCAGCATGCCTACAGGGCGGGCAGATCAACT
GATACTGCCCTATATCACCTGAAAAGCCTTATTGAGGATTCACTGACTCATAA
GGAAGTGGCGTTATGTGCCTTCTTAGACATACAGGGTGCTTTCGACAATACCT
CCCATGAGGCTGTCAACGCATCCCTGGCAAGAAGAGGACTAGATGCGACAA
CCAGCAGATGGATCAAATCACTACTGGCATCTAGGCGAGCCATGGCCACTATA
GGAGGACAGTCGTCCACAGTATCTACCACAAGGGGTTGCCCCCAAGGGGGT
GTCCTCTCGCCTCTCCTGTGGAGCTTGCTGGTAGACGAGTTGCTGGACAGAC
TGACCCGCAGGGGTATACTTTGCCAAGGCTATGCTGACGATATTGTCATTATA
GCTAGAGGTAAATATGAGGAAACACTCTGCGATATCATTCAACTGGGCCTCA
ATATGACCAGCGAATGGTGCAAGGAAGTAGGCCTGAGCCTAAATCCTAGCAA
AACTGTTATTGTTCCTTTCACAAATAGGTACAAGCTACAGAGAATGAAGGCA
ATAACCCTGTCAGAATGTCGCATAGAGGTCAGCAAAGAAGTTAAGTATCTTG
GGATAACCCTTGACTCCAAACTTAGCTTCAAAACTCATGTCGATAACACAATT
GATAAGTGCACCAGAGCACTTTTCACGTGTAGAAACATTGCTGGTAAGTCGT
GGGGAACCTCACCACGCATAATAAGATGGCTGTACCTCATGGTAGTCAGACC
CATACTAACCTATGGGGCAATAGCCTGGGGTGATAGAGCACGCCTTAGCACC
ACTAAGGTGAAACTCCATAAGCTGCAGAGAATGGCCTGCGTATGCATGACAG
GAGCAATGCGTACCTGTCCCACTGCAGCCCTAGAAGTACTAATGGAGGTGAC
GCCGCTTCATATCGTCATTGAAATGAAACGGAAAGCCACCCTAATAAGAATTG
AGGGAGCAGGAAATGACTGCAACCTCACAAGTAAGGATGCTGAAAGCCTAA

AAAGGGATATCCCTTTGCTAATGCAACCAAGGGATGAGATGCCAGCTGAGCA
TAGGTTCGCTCAGAACTTCAGCACTCATCTCAGTAATAAAAACAGTTGGACA
TCCCTGGGGAAAGTACACCCTACGAAACCACAAACAATAAAGTGGTATACAG
ACGGATCCCTCACCGACGAGGGAAGTGGGCTGGGGGTTGTAGGCCCCAGGT
TGAAATACCACGAATCAATGGGCAGATACACCAGCATTTTTCAAGCTGAAGT
CTGTGCTATTGGACGCTGTGCGGAGTTTAATCTGCAAAGGAACTATCGTGGC
AAGGACATTGCTATACTGTCTGATAGTCAAGCAGCCATAAAGGCGCTCAGCA
AAGCTAAGATAACATCTAAGCTAGTAAATGAAGTGAGGACAGCCCTAGACAA
ACTAGGAGCTGTCAACAAACTCACAATAAGGTGGGTCCCGGGACACAACAA
CATCCCGGGAAATGAGCTAGCGGACAACCTAGCCAGGAAAGGGGCAGAGAA
CCCTCTAATTGGGCCCGAACCCTTCTGTGGTGTTGGTCACCACAGAGTACTG
GGCTTACTTAAGTCAATAGAGGAAGAAAAACGTCTGTCCTTCTGGGAACACC
TACCAGGACTTAGGCAGTCTAAGATTCTCCTCCGTGAATATAACCATAAAAGG
TTCAAGACCTTAATGACACACGGGAAAAACACCGTGCGCATTTTGACTGGCC
TTCTTACGGGGCATTGCCGACTTCACAGCCATTTGCACAAGATTGGCATTGCA
GACAGTGAACTCTGTCGCTTCTGCTGCATGGAGGAGGAGAGCTCTGCACATA
TTATATGTGACTGCATGGCGCTTTCCATCAGGAGGAACAGACTCCTGGGCATG
TATGTAGTCCCACGGGAAACAATTGCAGCCCTGAACCCCAATAAAATTCTGG
CATTTATACAGTGCATTGGACTTCAGGGGGATCTTTGAGTCATGAAGGGGTAG
TACAATAGATCCGACTGGGTCGCAGTACACATACAAACCCACTTAATAATAAT

>ALA_ALAD#Unknown/ALA_ALAD

ACAGTCGCCACAGCAGCAGCAACAGCCGCCGCCCTATGAATCGCCCAAGGA
C

>ACCORD_LTRGypsy#LTR/ACCORD_LTRGypsy

AGTTACCATGCCCAGCATTAACCCCCCTCAACAACCACCTCCGCCTATGAAGC
CCGCCCGGTAGGCGACATCAGCAAAGTGCCAACGCTGTATATATATATATTGAT
CACGAGCTACCATGCCAGCATAGCCTCGTCCCCCGCTACCTGAAACTCTGTTG
CACCCGATGATGAAATCGGCATGAACACACACACACACACATATTCACACAT
ACTGCGTGGTGGCGACGCTCTCGACGTTGAAATTAACATGAACCTACACACA
CACATACACACTGCGTAATCGCGACGTCCTCGTCTAGACTCGCTATCTAGAAC
TAACGGGATCAAAAGCACTGCTGCTTGCCCGTGCGTATACATTAAGAATAAA
GCTTTCATCATTCTTGATCTTGACACCAAACCGAGCAGTTGATTTATTTAAAG
TGGCAAATATATATAACCTACATATATCATAAGTACACAATAAAGTCATTATTGA
CTCCCATCACATCAGCCTGGGCAGCAACTAACTAAGAGTAGAGAAAGGAGG
ACCCCCGATCCAACGGAGGCACCCGTAACT

>ACCORD2_LTRGypsy#LTR/ACCORD2_LTRGypsy

AGTTAACATACCCCCTCTCTCGGTCTACCCAGTACTGCAACTGAGAGCCGAG
CGAAAGTTCAACGAAGATCGCCAATACGAACTCTGACGAAGATATTTGGGAG
ATACATTATACATATTTGTAATAAAGAATTCATTATCATTCTCATTTCACACCTC

AAACTACTCAGTCGTTTTTTTGGAAGCTTTCCTCCCTCGGCCACGGGATTGTA
ACT

>ACCORD2_IGypsy#LTR/ACCORD2_IGypsy

GGCGCAGCCGGTCAACGGACAAGGGATTAACAATGCCCACTACGCTTAAAG
AGGTATTGAACATGTGACGGCAGTGGTTGTGGGGATTTTATTAGTGAGTGGTG
AAATAGAAAGGAATTGGATAAAGCGTATTGAAACATAATAACGTGCAAGTGC
AAGTAAAACAAAGGAAACAAACGAAATCAAGTTCAAATTGCTTCACAGGCG
ACCGTATATGTGTACATCAAACGCTGATAAGGCACCGGGATCGCTTGTGCGGG
ACAATTTACAACAATTATACCAACAAGTTACTGTGTGTGATGACCGTATATGT
GTACATCAATCGCTGATAAGGCACCGGGATCATCCATACACCCTAACTCATAA
AAATGGTAAATTAATCAACAAAAAAAAAAAATAAATAAATAAAAAAAAAAA
AAATAAAAGAGGAAAAATTTTTTCTGACATGTTAAACTACTGTGTATGGTTAC
CATTTGTGTGTCGGAGAGCGCTTGGTGTGTGCGAGAGTATTGGTTGCTCTTTG
GTTACTTCATAAGAATGGTAATTTCATCACCATGGTAATATTACATCAGCCTATA
TTCGCACTGCGAACGGTTGTTGTGTCTCTATAGTTCTGAGAACAACCGTTTGG
CACGGCGACAGAGCGACGTCGCTGCCGCTGAGTGTTGGTCGTTGTGCACAA
CAGAAAAGAGAATGGTAAAGAACGAGATCAGTCGAATATTAGACTACGTTGT
GACCAGAACTCGCTCCGCAAAGAATAAATTTTGGACACCGATCGCCTCCGAG
ACAACGGGACCTCCTACACAAGTGTTTTTAAAAAAAAAAAAGAAATAAATAT
AATAATAAATAAATAAATGTAAGTTGTGTGATTAACGACCGAATATGTGCACAT
CAATCGCTGATAAGGCACCGGGATCGTTTTCACTTAAAAATTTAAATGAACAC
AAATTTTTTTTAACAAATACAGAACTCGCTGTGCAGGAGGTGAATTTGAACAT
CAGTCGCCTCCAAGGCAACGAGATCTAATTCACAGTGGGTTATAAAAATAAA
TGTGAGTTGTGTGATTAACGACCGAATATGTGTACAACAATCGCTGTTAAGGC
ACCGGTATCGTTTTCACTTATCCATTTACATGAAAAAAAAATAATAATAATAAC
AATCATAATAATACTAATAAATAAAAAAAAATAGACAAAAAAAAGAAAAAAA
GGAAGAATAAAAAAAAAAAATTTGCACAGTGATACAGTTTCCTTCGGCAAAAT
CAAGTAAGCATGTATGTGTTTGTGTTGCGCCGCTTGTATAACCCTAGCGACGT
CGACGCATCACTGCACATTGGCTGCCGCCAAAAGCTGATGTCGCTCCAGAGT
GCGAAAGTTAAAAGTATATAACAGAAAGCATTTCAGACCCTGTAACGTATGTC
CCTTTGTACCCCTGTCCGGCCGTATGAACCTCAATTAGATTTGCAGACATTATG
ACTCCTACGCTTCGCCAGTATATACAGTATATACACTACAAAAAAAAACACTC
AATTAAAAAAAAACCCACTCAAAAAAAAAAAATGCATATATGTATAGAAAAAA
AAAGAAAAAAAAAAAACCAACTTAACGCCTACTGTTGTCACATGTTCACAGA
CCTATAAAAAATACACTCTATTCAATAGCTATTTAAATCATGTATTACAGCTAA
AGTCGAAAACACGAAAAAAAAAACTACAAAGGTGGATAGAAGCACAGCAG
CACTACAAGAACGACATATACTAAGCACCGGGAAACAAGGATAAGAAGAAA
CTGCGTAACGGACACACACATACAGGTTTTCTTTTATTTTGAAAAGAATACAC
ACATATAATACACATTCAAACAGATACATATACATAGCATCACAGAATATAATA
CAATATATTACTACATTAATATACAAACGAAAACAAAACAAGCCTAACACCCA
AAATTTTCCCTAAACAAAAAGATATTATTAATATACCTATTTTTACATATTATTA
CACATATCATAACTATACCCCAATATATAATTATTCACTATTATTTGCAAAAAAA

AAATACAGATTTCTTCCCAATACGTGTAAATGTTAGGAAAAAAAGCCAACATT
AGTAATACAGTTAGGGGCAGACTAGGGCTAAGAAGCGAATCTGGTCTATCTG
AGATAAGGGAAGCGACAGAACCTAAAGAAAAAGACCAGTCGGTTCAATCCG
AAGACAATAATCCCACCAACACTATGGATTCCGGTAACGACACAGCGGCAAT
AAGTCCTATCGTTAATTCATCAAGAAACACAAACCTGAGCCTACAACAATTAC
TAACCCTTGTACATCAACTACCAACCTACGAAGGCCCACCTGACAATCTTGAT
AGATTCATAGACAGAGTAGATCAGCTCCTTTTGTTAGCCTCATCAGTGGACCA
AACAACAGGCGGAAAGTACTTACTTGGAACTATCCGTGACAAAATTAAAGGC
AGGGCAGATGAAGCACTGAATGTCTGCGATGTACTCCTTACTTGGGATGACA
TCAAAATAAACCTCAAGCGACTATACTCTAGCAAGAAAACAGAAGAAATGCT
TGTTAGAGAGCTACACAACCTTCCGGACGGACTCAGTATGGGAAAATTGTAT
TACTCAGCCGCTAAAATAAGAAGCGATTTAATGTCACTCGCTAGAGAAGCCG
ACCCTAGTGCACACTCTCTCGCGGTCAAACGCGACCAATACGATAGATTCTG
CCTAAATACATTCCTAACTGGATTAAAGGACCCACTTAGTTCAGCCATTAGGA
ATCAAAGGCCAGAGACAATCGAGAAAGCATACGAGTACGGTCAGATTGAATT
AAATTTCCATAGAAGCCTGAACAAACACCAAGATAACCGACGACGCGACAA
CCCATTTCACAGGAATCACCCATACGCAAAAGGACAACCAACCAATAGCGAC
TACAACAATCGCTACATACCACGACAACAGAACTATAATAATGGCAACAACG
CTCGTCATGTACCACGATATGATAACAACAGCAACAACAATGATAGCCGCTTT
ATACAGAGACAGCAAAATTTTAACAACAATAATAATAGACGCTTCACACGTC
AACATAATTCCCATAACGATAGTAATGGTCGACAAGACAGAGAAACCCTGGG
CAGGAACCCCTTCCACAATAATGAACAACCAGATCAGACCCTTAGTCACCAG
AACAGGAACAAGCATAACCCAAGCGCTCCCCTATGTAACATTAACGATGATG
CGAATTTTCTACTAGAAGCCTCGGGAAGCCAGTCGGCCACATAAGCAAGAAG
ACTCACGGATCTTCCTTGCCATTTGTTTTCTTATCACCAAACTCCATGGTGTCA
AACCCTCTCAAATTCCTAATAGACACTGGATCTACTCACTCTTTCATAAATCCA
GCACTTATTAACGAAGAGCACGACAAAAAAATTAACCTCGGATATCAATATCC
ACACTGTTCTTAATAAATTCAAAATTCGGGAATATACCGATAAAATAAATTTCA
AACAATTCGAAAAAATACCCAATTTTAAGTTCTTATTATTTTGATTTCCACTCC
CACTTCAATGGCCTCCTAGGCATGGATATCCTATCTAGCCTAAATGCAAAAATA
AACCTTGCCGAATCCATACTAGAAACACCAGAAACCGCCATCCCAATCCTCA
CAAGAGCAAACCCTGTAGACACAGTATATAACCTGCCACAAAATACCAAAAT
GCTACTTCCGCTCCCAGTAAACTTAATGGAGGGAGATTTTATATATGAAACCA
CCAATTTGGATAATCAAGTATCCATAACAGGCGGTTTAAACACCGCAAACGC
AGGATCAGCTTATTTCGAGGTCTGCAATAACTCAGACCAGATCCAAACACTTT
ACCTCGAAGAACCGTTACAGGCAGAAGAGTTCTCTTCGCAAACCCACCAATA
CTTAAACTGCATGACCGCGGCAACAGATACAGAACGACAAGACGACCAACA
ATTAAACATTCTGACGGAACACCTCAACAGCGAAGAAAAACAATACATACTT
AAACTGTGCAAGTCATTCAAAAGACTTTTCCACAACGAAAACAACCAACTA
ACGTTCTCTAACGCCATCAAACACTCTATACCAACAACAGATAATATACCGAT
TCACACAAAATCATACCGTTACCCATTCGTACATAAAGACGAAGTCCGAAAG
CAAATTTCAAAAATGCTCGAACAAAACATCATAAAAAACAGCCATTCCCCCT
GGAGTGCACCCGTTTGGGTCGTACCTAAGAAAAGTGACAACATAGGCGAAA

AAAAGTGGCGCTTGGTAATCGATTTTCGGAAATTAAACGAGAAGACTGTCTC
TGATAGATACCCTATCCCAAACATTGCAGATATACTAGACAGCATAGGAAAGA
CCATGTATTTCACAACAATCGACTTAGCAAGTGGCTTTCACCAAATTCAAATG
AACCCACGCGACGCGAGCAAGACGGCATTCACCGTCGAAAATGGGCATTAC
GAGTTCACGAGAATGCCCTTCGGTCTCAAGAACGCCCCTGCCACGTTCCAAC
GCGTTATGGACAATGTTTTGGGTGACCTAGTCGGTAACGTCTGTCTCGTCTAC
CTCGATGACATAATAGTATTCTCCCCCTCACTCCAAAAACATATAGCAGATATT
AAATCTGTTTTCACAAAACTCCAAAACGCAAACCTAAAAATCCAACCAAGCA
AGTGTAGCTTCTTAAGGAAAGAAATCGACTTCCTGGGACATATCGTAACCCA
GGAAGGAGTCAAACCAAACCCACTTAAGATTCAAACCATCTAGGACTTCCCA
TGTCCAAAGACAACCAGGGAAATTAAATCATTTTTAGGACTATTAGGGTATTA
CAGGAAATTCATAAAGGACTTTGCGAAAATAACTAAACCCATAACGAGACAG
TTAAAAGGGAAGAAATTTATAATAATAGACGATGAATTTAGACAAGCATTCGA
AACGTCGAAAGATCTCCTTTGTAATGATCCTATACTCATATACCCTGATTTCAA
TAAACCGTTCACGCTGACCACAGACGCAAGTAATTATGCGATAGGCTCTGTG
CTGTCACAAGGTCCAGATACTAATGACAGACCCATATCTTTCTCTAGCAGGAC
ACTATCGAACACGGAAATCCGCTATTCTACGATAGAGAAAGAGATGCTAGCTA
TCATTTGGTCTGTACGTCACTTCAGACTACACCTCTTTGGGCGTAGATTCAAA
ATAGTCACAGACCATAAGCCATTAATCTGGCTAGAAAATCTGAAAGGTCAGA
ATCCAAAACTACTTCGATGGAAAACTATCCTGGCCGCTTACGACTACGAAGT
CGTATACCAGAAAGGGTCGCAAAACGTAGTTGCTGACGCACTCAGCAGAATT
GAGCCAAACTTAAACATAAACGAAGACCCACGAATTATACCAATCGTTTCGC
AACCACTAAATCACTTCAATTGCCAAATAATATTCTGTACCGGATCAACAACG
CTAAGAGAAGTAGAAACACCATTTATCCATAAAGTAAGACACATAATCACAG
AACCCGTGCATACTTTCGACTCCGTTACAAACACTCTTCAAGCAATCATGAA
ACCGAATAAAACAGTCGCTATATTTGCACCGGACGATATTTTCAAAATAATAG
AAGAGTCCTATAATAACTATTTTTTTGAAAATGATTTATACAAAATTGTCCGCT
GCAATATATGGCTAACCGAAATTACGAATCCTAACGATCAGGAAAACGAAATT
CGTGCGTACCACTTAAATACCAATCATAGGGGGATAGACGAAACGTATAACCA
TTTGAAAAGGGACATTTACTTCCCGCATTTAAAAGACACTATCACAAAAATTA
TAAAAAATTGTGATACCTGCCTAACCCTAAAATACGATAGACATCCCCATAGA
CCATTGATGCAAGACCCAACCGCCCCAGAGGGTCCATTGGCAGTCGTACATA
TAGACATATATTTCATTAATATACTTGTAACCTCACAATTATCGATAAATTCACC
AAGCTAGCGCAAGCCTACCCCCTAAGCAATAGAAATTCAATAAAGGTCGCCG
ATGCCCTATTACAATTCATAAGTCACTATGGCGCACCAAAGAAAATAATTTTC
GACCAGGGCGCGGAATTTTCGGGCAGTCTATTCAACGACCTCTTAACTCAAT
TCCAAATAACAGCTCATACAACTTCATTTCAACAATCAACGGGCAATGCTTCT
GTCGAGGGACTGCACTCCACCTTAACCGAACTATACAGGATAATCTTGAACA
AAAGAAAGGAAGCACGCCTAGAACGCGAGCACGTCCAAATACTCGCAGAAG
CAGTAGCAACATATAACAATGCAATACACTCCAGCACTTCCTATACCCCTTTC
GAGCTCTTCCACGGAAGAACTCACATATTCAACAAAACAATAATTATCGACA
ATCACCACGATTAAATTAAAAAATTAAACGAATTTAGAAAAGACATATACCCA
AAAGGACAGGAACATTTAAGCGCGACCGCAGAAAGAAGATTGGCTAAATTA

AACAAGGACAGAGAGAAACCAGTACAAGTCGAGCCTAATGATACAATATTTA
GAAAAGAAAACCGCAGAAACAAGATAACACCGAAATTTTCACTACACAAAG
TAGATAGGGACAAAGGCGTCACTTTCCTAACAACTAAAGGTCAAAAGTTACA
CAAACAAAAAATTAGAAAAAGAGTTAAGAAAACAGTGGCAGGAACATAAGA
AAGACAAGCACTATTTAATAAATATATTATTATCAAACTAAGTAACATAAAAAC
CCACATTAACAAGAAAGAACACTTATTTATTATAAAGTAGGAATAGCTTCAAT
TTAGACACTTTAATTACGTAAATAAAGTAGTCGATAGCCATTAGCCAGTAAAC
TAAACATTATCCCTAAACTGTTATATCATAAGAAACAAATAATAATATAAGATA
GAAATTAAGTAAACAAAATGTAACTTTAGCATTAGATGTAACGTTTATATACAC
AACACTCATGTTTAACATTAAGATACCCATTAAAAAAAAAAAAACTGTTAAAT
TCAAAATACAGTCGGAGTTCGTGGCATATACCAACAACACGCCACCTAATCA
CCTTGCTCCGATCCGGCCATTTCCCATTACGGCCTTCTCTTCGGACTGGGGGG
GGGGGGGGGGGAGG

>45S#rDNA/45S

AGTGTGAGCTTCCTTGGGTTTATAGTCACTTGTAACGGTGCTACAACAGACCC
AGAAAAGGTTAAGGCTATAAAAGAATTTCCGGAACCCAAAAGTGTTTTTGAG
ATACGGTCATTTCTAGGCCTAGCCAGCTACTACAGATGTTTTATTAAGGACTTT
GCGGCAATAGCAGGCCTATATCAGACATCCTAAAAGGGGAAAACGGGAACA
GTTAGTAGACACAGGTCACGAAACATTTCCGGTTTCAGTTTCTCGGAGACGC
AGCAACAAGCTTTCCAGAAACTACGAAACATCTTGGCATCTGACGACGTGAT
GCTAAGGTACCCCGACTACAAAAAGGATTTGATCTAACGACAGATGCCTCGG
CCCATGGCATTGGCGCAGTATTGTCCCAAGGACATTGAAGGAAAGGGAGGTT
AACTACGCCACCAATGAGAGGGAACTCTTAGCCATAGTCTGGGCTTTGGCCA
AGCTACGGCATTACCTGTATGCGGTTAAAGACATCAACATCTTTACCGACCAC
CAGCCGCTAACCTTTGCAGTATCGGAATCCAATCCGAATGCAAAGATTAAAA
GGTGGAAAGCGCGCATTGATGAATCAGGAGCGCGTATTTTCTACAAACCCGG
CAAAGAAAACTTGGTCGCCGATGCATTGTCACGGCAACAAATTAACGTCATG
GAGGAGCAAGAACTCAATCGTGCGTGGCCACTGTTCACAGCGAACTCTCCTT
GACGCACACTATCGCAACGACGGATAAGCCCCAAACTGCTTTCAGAACCAG
ATAACTCTGGAGGAGGTACGCTTTCCGTTAAAGCGCAGCTTCGTCCTCTTTGG
AAACAAGAGGCGGCATGCGATTAACTTCCCCTGCAAAGAGTCATCGATTGAT
GAACTCGCAGATGTAATCGTTTCGAAAGGGCGCAAACGCTATACATTGTGAC
CTGCACACGCTGGCACTAATACAGGCGAGTGGTCGGTGTTCCAGCCACCAAT
TTGGCACTGCAAAACGTGTAACGGTATTTTGCAGTCCCGAGAGACGGGAAAT
TCTTACCGTAGAACACAATAGGGCCCACAGGTCGGCCCAAGAAAACGTTAA
GCAGGTACTCTCCGAGTACTACTTTTCCGAAGATGACCAAGTTGGCTACCGA
AATCGTAGCAAACTGCAAAACATGCGCAAAAGCCAAATACGATAGGCACCCA
AAACAACATGAGCTAGGCGAGACTCCAATCCCCTCTCATGTGGGAGAACTAC
TGCACATCGACATTTTTTCTACGGATAAAAAGTATTTTCTCACTTGCATTGACA
AGTTTTCGAAGTTCGCAATTGAGCAACATGTCCACTCTAGGACAATCGAAGA
CCTTAAACCGGCCATACTACAGATTATGAATTTTTTCCCTAGGGCTAGAGTAG
TTTACTGCGATAATGAACCTTCATTAAATTCGCATACTATCTCGACCATGCTTG

ACAATCACTTTGGCGTTAGCATTGCCAATGCACCGCCACTCCATAGCGTGTCA
AATGGGCAAGTGGAGCGCTTCCATAGCACCCTTTTGGAGCTTGCTCGTTGCC
TAAAAATCGACAAGGGCATAACCGATACTGTGGAAATTATTTTGTTGGCAACC
GCCAAGTATAATGAGTCAATTCACTCGTTGACAAACGACCAGTTGACATCGT
GCAGGAGCACCCAGACCCACAGACGGAAGTCCGGAACAGAATCATTAAGGC
ACAAAACACGCTCAGGACTAGGGAAAACGCCTCCCGACAACACAGAGTATT
CGAAGTCGGCGAGAAGGTATTGGTTAAATCCAATAGAAGGCTGGGAAATAAA
CTCACACCATTGTACGAGGAGAGAGCCGTAGAAGCGGACTTGGAACCACGG
TCCTCATCAAGGGGAGGGTGGTCCACAAGGACAATCTTCAATGATATGACCA
ACAATATCCTTTATGGTTTTTTTTATTATTCATTTTTTATATTATATTTTTTATCAC
ACCTTCAAGCCATCTGGCGCACTTTATTTTTTATATTCATTAGTTATTACACCTT
CAAGCCGCTTGGCGCACTTTGTTTTTTACTATAGGTTCGTAGCCACTGGCATA
GTTTGTTTTAAGTTCATTTTATCGGTGGTGGGAAAACCCTCTATAAGCATAAA
AATAGCTAAAGCTTAATTTCACAGGATCGGACCAACATTTTGCATACTCCTGC
CCTTGGCGTCGGCCCACGTTACCGATTATTCACAGGCTAGGTACATCCCCGTT
ATAGATGGCGAAATCCTAGTATGGGAGGAGTTTGCTTACGTCACGCACACAG
CAAACCTCTCAGAATATGGGCGTGTAATAGAGGAGACAACCAACATGATAGA
CATGTTTCCGCTATCCCATATGAAGAAGCTTCTGAGCGTGGATACCGCCCACC
TCCAAGACTTGTTAGAGTCGTTGGGCGTTCATCACAGAGTAGCTAGGAGCTT
GGATTTCTTAGGACCTATGCTAAAGGTTGTAGCAGGGACACCAGATTCCAGC
GACTTAGAAAAAATCAGGTTTACCGAAATGAGGTTAGTCGAGTCTAGCAATA
GACAGATCCAAATTAACACCAAAACCGAAAATCAAATTAATCAACTTACCTC
TAACCGTCAATTCAATTTTGAGATCAGCCAAACCTCACAAATAGACACTGGA
CACTTATATGAAACACTGCTTGCTAGAAACCGCATGTTTATGGCGGAATTACA
AAATTTAATGCTTGCAATAACCCTAGCCAAGATTAACATTGTTAGTTCGAACA
TTTTAGATCACGAAGACTTAGAAACAGTTTGGCTTGAGGAACCCACCGACAC
ACCTATAGGAGATCTTTTGTCCGTCTCGTCTGTAAAGGTTTTACAGTCCCGTA
ACATTTTACACTTTATCATTAAATTCCCCAAAATTAAATTAGCCTGCAAGAAGA
TCACTATTTTCCCAGTTGCCCATGGTGAAACGATGTTGCAGATCATAGACAAT
GTCATAGCCGAATGCAGCGGAGAAGTTTACGCCATCAAAAACTGCTCCGAAT
CACCGAGAGCCACATTCTGCCGCCTAGCTTCAGAGAGTTCGTGCGCCAAAGA
CCTGCACGCCGGAGGGGTAGCACACTGCCGAGTACAAGAGAGTGACCTGCA
TCCGATAACCTACGTAGACGAAGGAATTATTATCATCAACGATAGGTCGGCCA
AAGTGCGAGTGGACAACGGCTACTAGAAATCTTGGACTCATGGCACACACCT
CATAACCTTTGACAAACAGGCCACCATAAATGACACACCTCCTTCATCAACC
ACAATAACACCCAGAAGAGAGCTCTAGGGACAGCAAGTCTTCCCTTGTTGA
ACATCACCGCCACCCAAGATGTCCTCAGCCTCCCGTATCTTCACCGTCTGAGT
GAACGAAACTTGGAGTTCATTAAGGAGTTCAGAGAAGAGATTGAGAATCAA
CGAACACGTCTCGTAGCAATTATGCAGGAGCAATATGCTGCGCACTCATCTGC
ATCGGGCTTATTTTTAGGCGTTTCACTGAGGCAAGAAAACCTGCAGGCCAAG
TTAGGCAGATTATCGCTGAACTACAGACGCCGAGGGCGGCCTTAATTCTGAG
GGGGAGTAGTTAACTAAGTTAACCGGGCGACGGCTACGACCGATCGTCCGAA
GACTCGCTCCGGCCAAATTGCTGACACAGCGTCTGGCCGGAAGCCCGTGCAT

AGCCGGCAAACACTACCCGTTGGAGTGGCCGCTATGAGTTTCATATTTTGTTA
GCCTTAGGTTCAGTTTGATTTTATATTTAATAAAGGACCGCATCGCGCCTTGAA
TCGACTCCAGCTGTTGCTGTCATTATTAAATTGGTTGATTGGTTGACCAGCCT
TAAGGGCACTCAACAACGGAGAGACGTTCTCCCACCGTATCTTGCCTAATTC
TGAGGAGAAGAGGTCTCGCCGGCAACCGCCCTGCCTCCCGGATATCCGCAAT
CCACGCCCGAGAACCGCGGCGAGGGTATCTACACCAACATAATTAATTTAATT
TACACACCGCCGTCGCTACTACCGATTGAATTATTTAGTGAGGTCTCCGGACG
TGATCACTGTGCGCCTTGCGTGTTACGGTTGTTTCGCAAAAGTTGACCGAAC
TTGATTATTTAGAGGAAGTAAAAGTCGTAACAAGGTTCCGTAGGTGAACCTG
CGGAAGGATCACTATTGTATAATATCCTTACCGTTAATAAACATTTGTAATAATA
CAAATAAAAACAATTTACCAAAATAAAAATATAACAAAATGGTTCCACGGAAT
CAAAAGTTAAAATCAAAATAAAACGAATATGGGTTTTATTTATATAGTGTGGG
GCTTGGCAACCTCATAAAAAGATTTTAACATTCCTAATGTATGTTGTGCGTATT
TGTGGCGAGTACTTACAACAACGGCGTTTCCTATAAAATAATGTTTCGAACAT
AAAAATCGAAGAAACAAAATTCGAAAGTGGAAGTCGAATCAAAATAAAATA
ATTTCGAATGTGGTAATCATCGAAATAAGTGTTAATATAATTGGTAGATATTAA
CTAATTTTTAAAATTTGTGTGTATTTATTACTATACACGCGTTGCGAATATGTAT
TGTTCATCTTAGTTATGGGCATACGTTGGCTAATGCAACAACCTGAAATAAAC
AATGTTGTACCTGCATCCATCAGGTTAATGTTTTATATAAATTGCAGTATGTGT
CACCCAAAATAGCAAACCCCATAACCAACCAGATTATTATGATACATAATGTC
TTATAAGAAACATTTTCGCAACATTTATTTACCTATATAAATTCATTTATTGAAG
GAATTGATATATGCCAGTAAATGGTGTATTTTAATTCTTCAATAAAACATAATT
GACATTATATAAAATTATAAACTCATGCGGTGGATCACTCGGCTCATGGGTCG
ATGAAGAACGCAGCAAACTGTGCGTCATCGTGTGAACAGCAGGACACATGA
ACATCGACATTTTTACGCATATCGCAGTCCATGCTGTTATGTACTTTAATTAATT
TTATAAAACTAAATAATTAAAAATGGATAAATAAAAACTTTGCATACAAGAAT
TAATAAAAATGTTATAACGAATTTAATTAAATGTTTTATCATTATATATAAAGAA
TTTATGGCAAGATAAAGTTATATACAACCTCAACTCTATATGGGACTACCCCTG
AATTTAAGCATATTAATTGGGGAGGAAAAGAAACTAACAAGGATTTTCTTAGT
AGCGGCGAGCGAAAAGAAAACAGTTCAGCACTAAGTCACTTTGTCTTAGTC
AGCGTAATTAACTTGACTAATAATGGGATTAGTTTTTAGCTATTTATAGCTAATT
AACACAATCCCGGGGCGCTCTATATACTTATGTATAATGTATATTATTATTATTTA
TGCCTCTAATTGGAACGTACCTTGAGCATATATGCGGTGACCCGAAAGATGGT
GAACTATACTTGATCAGGTTGAAGTCAGGGGAAACCCTGATGGAAGACCGA
AACAGTTCTGACGTGCAAATCGATTGTCAGAATTGAGTATAGGGGCGAAAGA
CCAAGCGAACCATCTAGTAGCTGGTTCCTCCCGAAGTTTCCCTCAGGATAGCT
GGTGCATTTTAATATTATATAAAATAATCTTATCTGGTAAAGCGAATGATTAGA
GGCCTTAGGGTCGAAACGATCTTAACCTCTTCTCAAACTTTAAACGGGTAAG
AACCTTAACGGGCTTGATATGAAGTTCAAGGTTATGATATAATGCGCCCAGTG
GGCCACTTTTGGTAAGCAGAACTGGCGCTGTGGGATGAACCAAACGTAATGT
TACGGTGCCCAAATTAACAACTCATGCAGATACCATGAAAGGCGTTGGTTGC
TTAAAACAGCAGGACGGTGATTATGAAGTCGAAATCCGCTAAGGAGTGTGTA
ACTAACTCACCTGCCGAAGCAACTAGCCCTTAAAATGGATGGCGCTTAAGTT

GTATACCTATACATTACCGCTAAAGTAGATGATTTATATTACTTGTGATATAAAT
TTTGAAACTTTAGTGAGTAGGAAGGTACAATGGTATGCTAGAAGTGTTGTCGT
AAGCCTGCATGGAGCTGCCATTGGTACAGATCTTGGTGGTAGTAGCAAATAAT
CGAATGAGACCTTGGAGGACTGAAGTGGAGAAGGGTTTCGTGTGAAAAGTG
GTTGATCACGAGTTAGTCGGTCCTAAGTTCAAGGCGAAAGCCGAAAATTTTC
AAGTAAAACAAAAATGCCTAACTATATAAACAAAGCGAATTATAATACACTCG
AATAATTTTGAACAAAGGGAATACGGTTCCAATTCCGTAACCTGTTGAGTATC
CGTTTGTTATTAAATATGGGCCTCGTGTTCATCCTGGCAACAGGAACGACCAT
AAAGAAGCCGTCGAGAGATATCGGAAGAGTTTTCTTTTCTGTTTTATAGCCGT
ACTACCATGGAAGTCTTTCGCAGAGAGATATGGTAGATGGGCTAGAAGAGCA
TGACATATACTGTTGAGTCGATATATTCTCTCCGGACCTTGAAAACTTATGGTG
GGGACACGCAAACTTCTCAACAGGCCGTACCAATATCCGCAGCTGGCCTCCA
AGGTGAAAAGTCTCTAGTCGATAGAATAATGTAGGCGAGGGAAGTCGGCAA
ATTAGATCCGTAACTTCGGGACTTCGGGATAAGGATTGGCTCTGAAGATTGAG
ATAGTCGGGCTTGATTGGGAAACAATAACATGGTTTATGTGCTCGTTCTGGGT
AAATAGAGTTTCTAGCATTTATGTTAGTTACTTGTTCTCCGGATAGTTTAGTTA
CGTAGCCAATTGTGGAACTTTCTTGCTAAAATTTTTAAGAATACTATTTGGGTT
AAACCAATTACTTCTTATTAATTATAACGATTATCAATTAACAATCAATTCAGA
ACTGGCACGAACTTGGGAAATCCGACTGTCTAATTAAAACAAAGCATTGTGA
TGGCCCTAGCGGGTGTTGACACAATGTGATTTCTGCCCAGTGCTCTGAATGTC
AAAGTGAAGAAATTCAAGTAAGCACTGGTCAACGGCGGGAGTAACTATGAC
TGTCTTAAGGGGAGTCATGGGGTATTTGAGAGCAGAGGGGGAGTATTCTTCT
GTAATTCGTAAGTCATATCATATGATGTGCGGAAGGGGAATTTTACTCTGTAAC
TCACAAGTCTCTCCTTTACTCAAGTCGAC

>5S#rDNA/5S

GCCAACGACCATACCACGCTGAATACATCGGTTCTCGTCCGATCACCGAAATT
AAGCAGCGTCGGGCGCGGTTAGTACTTAGATGGGGGACCGCTTGGGAACAC
CGCGTGTTGTTGGCCTCGTCCACAACTTTTT

>TRANSPAC_LTRLTR#LTR/TRANSPAC_LTRLTR

AGTCATGTATACCCTTACATACCCTCTTATTAGATTAAGTTATGTTAGAATACAC
ATGGCGCTAAGCTTTTTGTCTGAGCTTAGCTTTATTTTTAGTCAGTAGATAAGA
AGACCTCGATCTGTACACATCATTGAATAAACCCTCAATTTGTTAAGCGAAAC
CTTTCGTTTTGTTTTAACCCCTCTTTAATCGACAAAGAAGTCGATGGGTGCTT
AACTGAGGAGTTCAACCTTCAACCTACAGACGACAACGAAGTCGTTTCTGG
AGGCAACGATCTTTTGGACACAACGGTCTCTGCTCGGAAGGCCCTATCAACC
TTCCACATAACT

>TIRANT_LTRLTR#LTR/TIRANT_LTRLTR

AGTTACCACCCCACCCCCTAAACCCCCACGCCTCTAAACAAATCATCGGACA
CTCAACCGGGAAGACGGCAACTGGAACACCGCATCCGGCCGAATGCTGACA
TTCCGGCCGAATGCTGACATTACACAAAAGTCGCACTGCAACATTGTCCCCA

GCTAGCCAGCCACATGCCGAGTCGGCATGTTCATTATGCTTACAATTAAGAAC
CTATGTACTTATGTATAAGATGAAAACGGAGGACTCGAGTAGCCACTCTCTGA
CAATAAACTTCTTACTGATTTTGAACTTCAAGAAAGTCAGTCGTATTCTTTATT
GGAAATCTTCACACTACAACTATCTGCTGAAACTTAAAAACCTTCATACATTT
ACACATCATATCTTCACAAAAGGCTCCACCCTCGATCACGGACTTAACT

>R1_Non-LTR#Non-LTR/R1_Non-LTR

CGGACGTGTTTTCGTTGCGCTCGTGGACATAGTGCGAAGAACTTTGTTTTCC
GTATTTGGAAGTATACGGAATAAATAATTTAGTGTTCCGTGGAAGTGGTGCGC
AAATTTTCGCGAATTAAAAACAAGCGGTTTGGAAGTAATTGACAATAAATTAT
TGGAAATTTTCCACTCCGCACGTGTTGAGCGGCGGAGCTTGCGGGTGAGCTT
TTCGAACAGCTGAGAGAAGCTTATTGGTGGTAGTCACCGCTAAGGATTGTGT
CTTGGGACAGCTTAGTGCACTCTACCAATAGGTGGAGCTATCACCATAGCAA
CTAGCCCGTGTCAGCGAGCATACGATTGCTGGACTCGTCAAAAGGAGGAGCC
ACCATCGGAGCAACGCCGATGGAGAGCGACAGCAGTGTGAGTGCCTTGAGC
GGAAGCAGTGCCTCAAAGGTGTCAAGACGAGGCAGGCGTAGGAGCCATCTG
GCCTCCAAGAGCTCGGCGCCAACGCAGGCGAAACTGGTTGCCCTGGCCTCG
AATGGAGTGCCGGAACCCGTTGGTGTGCTGGAGGAGGCGTTTTCGTCGCTG
GAGGATGCCCGGGCGGCTACGTCAAACGCTGCCAACGATGCTGCCCCCCCCG
CTGCTGCCCCCGCTGTTGATCACACTGTTGCCCCTGATGTTTCCACTGCTGCT
AAAATCGCTGCCACCACTGCCACCGCTGCCACCGCTGCCGCCCGTGCTGGGC
AAGCAGCCATGATGGCAGAGCTGTCGGCCACCCAGCGCATGGTGCGAAACA
GTTTCCGCAGCCTAGGAGGCGTAGACACGGAAGAGCTCTCGTGTGCCATCAG
CCGCTATGATGAGCTGGTGATGGCATTAATGCTCCGGTGTGGAGAACTGGAG
ACGCGGCTCGCTATGCCACCACCGCCGCCGCCGCCGTCCAAGGCGAACACTA
CTGCCGCCAATGCTCCCCAGATGCCTCAGGTTGCACCCATCGCTGCCCCGCG
GACAACCAAGGTTCGTGAGACGTGGTCAGCGGTGGTGAAGTGCGACGACCC
TGCGCTATCGGGGAAAGCCATAGCCGAAAAGGTGCGGACGATGGTTGCACC
CTCCCTCGGAGTCAGAGTACACGAGGTACGTGAGCTGCCGTCGAGGTGGTG
GTGCGATCATTCGTACTCTTCGGTTGGAGAGCTGCAGAAGGTGATGGCATCG
AAAAGATTCGCAGAACTTGGACTGAATGTGGCACGGAACGCGGCCGAGAAG
CCGAAGGTCATAGTCTATGACGTCGACACAGCCATCGGCCCAGAAGAGTTCA
TGCAGGAGCTTCACGAGAACAACTTCGACAGTGAAATGACTCTGGCCCAGT
TCAAAAAGTCGGTGCACCTGGTGACCAAGGCGTGGTCGGCTACTGACGGTG
CCACCGTAAACGTGACGCTAGAGGTAGACGACCGGGCGATGGCGAAACTTG
ATGTAGGACGTGTCTACATTAAGTGGTTTTCGTTCCGATGCCGATCGCAAGTC
CGCACCTATGCCTGCCACAGATGTGTGGGTTTCGACCACAAGGTTAGTGAAT
GCAGGCAGAAGGAGAGTGTTTGCCGCCAGTGCGGGCAACAAGGCCACACC
GCGGCAAAGTGCCAAAACCCGGTGGACTGCCGGAACTGCCGTCACAGAGG
GCAACCTTCGGGGCATTATATGCTCTCGAATGCTTGCCCGATATACGGAGCGT
TGTTAGCGAGGGTGCAAGCTAGACACTAATGTTTAGCTTCATCCAAGCGAAC
TGTGGCCGAGGCAGAGCTGCGACCATCGAGCTCGGAGTCCGACTCAGGAGA
TCGGAGTCAATGTTTGCTCTGGTGCAGGAGCCGTATCTTGGCGGGGATGAAA

TGGATGTGCTGCCTGAAGGAATGAGGGTTTTCACCGACCGGCGAGGGAAGG
CAGCCATCCTAGTGGATCATCAGGAAGCCATCTGCATGCCAGTGGAAACTCT
CACCACAGATTATGGCGTATGTCTGGTCGTTAAAGGGAGTTTTGGCTCAATCT
TCCTTTGCGCCGCATACTGCCAGTTCGATGCACCTCTGGAACCGTACCTCCGG
TACATGGATGCGGTCCTGCTGCAGGCCAGCAGAACCCCCGCAATCCTGGGCC
TCGACGCGAATGCAGTGTCCCCCATGTGGCTTAGCAAACTCTCTCGTCATGCC
GAGGGGCAAGCTAACTACAGACGGGGTGAGCTGCTGTCTGAGTGGATGCTG
GAGGCAAGAGTCGCCGCCCTAAACCAGTCAACAGAGGTGTACACGTTCGAT
AATCACAGAGCGACTAGTGATATCGACGTGACAATCGTCAATGAAGCAGCAT
CTATGTGGGCCACATATGAGTGGAGAGTGGACGAGTGGGAATTGAGTGACCA
CAACATCATTACTGTTGTGGCCGAACCAACTACCGCGCGCTCAGTTGAGAGC
ATAGCTCCTGTGCCGTCCTGGAACTTCTCCAATGCACGTTGGCGATTGTTCAA
GGAGGAAATGGTGAGTAGAATAGCCGAACTTCCGGAAAACTTTTCAGAGTC
GCCGTTGGACCAGCAAGTTTCGACCCTGCGCAGTATAGTACATAGTGTATGTG
ATACTGCGCTAGGAAGGAAGTTGACTCGATCGCCCAGCAGGAGAGCACGTT
GGTGGACTGCCGACCTCTGCGCTGCAAGGCGCGAAGTCCGAAGACTTCGTC
GCCTGCTCCAAGATGGAAGGCGTCGAGATGACGATGCCGCTGTAGAGCTTGT
AGTGGTCGAGCTGAGGCGTGCCTCAGCCTACTACAAGAAGCTCATTGGAAG
GGCGAAGATGGATGACTGGAAACGCTTCGTGGGAGATCATGCCGACGACCC
ATGGGGGCGCGTCTACAAGATTTGCCGAGGTCGCAGGAAGTGCACGGAGAT
TGGGTGCCTCCGCGTGAATGGCGAGCTGATCACTGATTGGGGTGACTGCGCA
CGAGTGCTCCTCCGCAATTTTTTCCCAGTTGCGGAGTCCGAAGCACCGACTG
CCATCGCGGAGGAAGTCCCACCGGCCCTCGAAGTATTCGAGGTTGATACATG
TGTTGCCCGGCTGAAGAGCAGGCGCTCTCCCGGGTTGGACGGCATCAATGGC
ACTATCTGCAAGGCAGTCTGGCGCGCCATACCCGAGCACCTAGCATCATTGTT
TTCCCGATGCATCCGATTGGGATACTTTCCAGCCGAGTGGAAGTGCCCACGA
GTTGTCTCGTTGCTCAAAGGGCCAGATAAGGACAAGTGTGAGCCCTCCTCAT
ACAGAGGAATATGCTTGCTACCAGTCTTTGGAAAGGTGCTCGAGGCCATCAT
GGTGAATCGTGTGAGAGAAGTTCTTCCGGAAGGCTGCAGATGGCAATTCGG
ATTTCGCCAAGGACGATGTGTGGAGGATGCTTGGAGGCACGTGAAGAGCAG
TGTTGGCGCCAGCGCGGCGCAATACGTGCTCGGCACATTCGTGGACTTCAAA
GGAGCATTCGACAACGTCGAATGGAGTGCTGCACTCAGCCGACTAGCCGACT
TGGGATGCCGGGAAATGGGCTTGTGGCAGAGCTTTTTCTCCGGCCGAAGAGC
AGTGATCCGAAGCAGTTCCGGTACTGTGGAGGTACCGGTAACTAGAGGCTGC
CCGCAGGGATCAATCAGCGGCCCATTTATCTGGGACATACTGATGGATGTACT
GCTTCAGCGTCTCCAGCCGTATTGCCAGCTGAGTGCATACGCGGATGACTTG
CTGCTTCTCGTCGAGGGAAATTCCCGAGCTGTGCTAGAGGAAAAAGGAGCG
CAACTAATGTCCATCGTAGAAACGTGGGGAGCGGAAGTTGGCGATTGCCTCT
CGACCAGCAAGACGGTAATCATGCTGCTGAAAGGTGCCTTGAGACGTGCGC
CTACGGTGAGGTTTGCTGGACGGAACCTTCCGTATGTGCGTAGCTGTCGGTA
CCTTGGCATCACGGTCAGTGAAGGAATGAAATTCCTCACGCACATAGCTTCG
CTTCGCCAGCGGATGACAGGAGTCGTTGGAGCATTGGCGCGTGTGCTTCGAG
CCGACTGGGGCTTCAGTCCTCGAGCCAGGCGGACCATATATGACGGACTCAT

GGCACCTTGTGTGCTGTTTGGTGCCCCGGTATGGTATGACACCGCGGAACAA
GTAGCTGCCCAGAGGCGACTAGCCTCCTGCCAGAGGCTAATCCTGCTTGGAT
GCCTTTCGGTATGCCGAACAGTATCCACAGTGGCACTGCAGGTACTTGGTGG
AGCTCCCCCGCTTGATCTGGCTGCTAAGTTATTAGCGATCAAATACAAGCTAA
AACGTGGATTCCCGCTGGAGGAGAACGACTGGCTTTACGGCGAGGACATTG
CGTGTCTTAGCTGGGAGCAGAGGAAGACTCGCCTAGAGGAGTGTTTAATCCA
GAGTTGGCAGAACAGATGGGACGATGACAGCGAACCAGGACGGGTGACGC
ATAGGTTTATCCCATACGTCACTCTTGCCTATCGGGATCCAAGTTTTGGATTCT
CGATGAGGACGTCTTTCCTGCTTACAGGGCACGGGTCGTTCAATGCATTTTTG
CACGGGAGAGCCCTCAGCGATACCACTGCTTGCGCATGTGGAGATCCATATG
AGGACTGGATGCATATCTTGTGCGCTTGCCCCCTATATGCAGATCTGCGGGAC
CTAGATGGACTTGGAGTGCAGCGCCTTGGCGAAAACTGGATCTTCGAGGGA
ATCCTGGATGATCAAGAGAAGACTCAACGGCTGGCAATGTTTGCGGAAGAA
GTGTTCCTGAGGAGGAGGGCCCTTTAGCTCAACATCTCTGCCGTGTGGTTAG
CGGGCGAGAATACTACCACAGTCCGCTGTTGCTTGTCGTAAGAGACGACTAA
TACAGCGATAGGATTCCTCTAACCCTGCTTGTCGGAGCAAAAGGGGGAGGCC
CACCGAGCCTCTTTTCGGTACCACGGGTTGAGCAGCTATCCAAGACTGCTCA
TTGAGGTAGGCCCCCTGGTGGGAGTATCGTGGTGGCTGTGGTTGGTACCCAT
ATCGCGGGTAGAGCCTTCATGCTCGACGTTTGAGTTACGGTGCTAGTTGCGC
AAAACTCGGGTGCTGTGACCCAGAGATCAGTAGAGATTTTAGGTAGATCTCG
CTCCTCAGCAAGGGGGAGTGCTTGCCCGGCAAGCAAGTACTCGAATTGCTAC
CGGGGTGGTCGCTATGTACATAGCTATAGCTTCTAGTCCGGGACGCTTGTCTG
GCGTATCCAGACACATGCACCATATGCTCACTTGTGGGCGTATAGGGTGCCGT
GGTTGTAATCCCTTCAGTGTGGAACACGCCACGTAAAATAAGTTCGGAGGGA
TCCGAAAAGCATACAT

>R1-2_Non-LTR#Non-LTR/R1-2_Non-LTR

CTTGCTCGGCGGTAGTGATGTGCGACGACCCGAGCATACCTGGGAAGCAGA
GTGCTGAGCGGGTCCGCAAGGAGGTCGCCCCTGCCCTCGGTGTCCGTGTACA
CGAGGTGCGTGAGTTGAAGTGCGGTGGAGCCGTCATCCGCACACCATCGGT
GAGCGAGATGAAGAAGGTCGTCGCGAACAAGAAGCTTGCCGAGGTTGGTCT
CAAAGTTCAGCCGAAGAAGAGCCAACGACCCAAAGTCCAGGTGTTCGACGT
CGACAGTGGAATACAGCCTGATGGAGGAATTGTACGTCAACAATTCAAGGAA
GAGTTCTCCCCAGCCGCTTTCATGAAAGAAATGCACCTCAACACGAAACCGT
GGTCAGTCACCGATGGCGAAAGAGCTAATCTGACGCTTGAGGTTGACGAAA
AGGCGCTGAACGTCCTTGAGCAGACCGGACGGGTCTATATCAAGTGGTTCAG
TTACCGTTGCCGGTCGCTCGTCCACACCTATGCTTGCCATAGGTGCCTTGGAT
TTGACCACAAAGTTTCCCAGTGCAGGGTCAAGGAAACCATTTGTCCACAATG
TGGACAAGCTGGCCACACAGCACCCAGATGCACGAATCCGGTGGACTGCCG
GAATTGTCGTTTTAAGGGCCACCCTTCGAGGCATTCAATGCTGTCCTTCACGT
GCCCCATCTACGGTGCGGTGCTTGCGAGGGTGAACGCTAGACATTAATGTTTA
GCTTCCTTCAGGCAAATTGTGGCCGTGGCCGAGCTCCTACCATCGAGCTCGS
AGTCCGCTTGCGCGACTCTGGCCACTTGTTCGCATTGGTGCAGGAGCCTTAT

GTGGACCTCGCTGGACGAATCACGGGAGTCCCATCCGGGATGCGTGTGTTTT
CGGACAGGCGTGGAAAGGCTGCCGTCTACGTGGACAGCACGGATTCCATCT
GCATGCCGATAGAGCCGCTTGTCACCGAGTTTGGAGTATGCGTGAGCGTTAC
TGGAAGTTTTGGCTCAATCTTCCTATGCTCCGTGTACTGCCAATTCAACACCG
GACTCGAACAGTACCTCGGGTACTTGGATGCGGTGCTGCTGCTAGCCAGCCG
CACGCCTGTCATCCTTGGTCTCGACGCGAACGCAGTATCCCCCATGTGGTTTA
GTAAACTCCCTGAGCGTGCTCAGGGATCAGCTAACTACTTACGGGGTGAGCT
GCTGTCTGACTGGATTCAAGGATGTCGAGCCGGAGTGCTGAATATGCCGTGT
GACGCGTTCACATTCGAGACTCCCTACGCACGTAGTGATATCGATGTGACACT
CACCAACGATGCAGCGTCTACGTGCGCTACGTACGACTGGAGAGTGGATGAA
TGGGATCTTAGTGATCACAACATTATCAACGTTGTGGTTACGCGAGACCCACC
AAACACAGTTGAGAGCTTTGCTCCTGTGCCATCCTGGAACTTCTCCGCTGCA
CGCTGGCGCCCATTTGAGGATGAGGTGACAAGACTGGCTTCGGAACTCCCG
GACGAATTCGCCGACACGCCGTTGGATGACCAGGTCTCTGCAGTGCGCTCGC
TCGTGCACTCTGTGTGCGATCAAGTGCTGGGACGCAGACGACCCAAGACCG
CAAGGAAGATAGTTTGGTGGACTGCCGAACTTTCTTCTAAACGCCAAGAGGT
CAGGAGACTGAGGCGGCGGCTTCAGACCGCTCGTGCGCGCGCAAGCCACGA
TGCCGAGCAACTTGTCTCTCAGTTGAGGGAAATCTCAGATCAGTACAAGGAG
CTCATTCTGAAGTACAAAGAGGATAACTGGAGGCGCTTAGTGGGAGAGAAC
AAGGACGATCCATGGGGGCAAGTCTTTAGGATTTGCCGTGGCCGCAAAAGG
ACAACCGAACTCGGTTGTCTTCGCTNAGGTGGCAGGCAGTACGTAACCTGGC
ACGACTGCGCGGGTGTTCTTCTCCGGACCTTTTTTCCATTCTCCAGTTTTCGG
CACCCACTGTCATTCCTGAAGAGATTCCACCACCGCTTGTCCTCTGAGGTAG
ACGCTTGTGTCGCAAGGTTGAAGAGCAGACGCTCTCCCGGCATGGACGGCA
TCACCGCGGTGATATTCAAGGCGGTGTGGCGTGCCATTCCCGAGCACATCAC
AGCGTTTTACTCCCGCTGTATCAGAAGTGGATACCTTCCCTCTAAGTGGAAGC
GCACAATTGCAGTGGCACTACTCAAAGGGTCGGATAAGGACAGGAGTGATC
CTGCTTCTTACCGCGGTATCTGTCCGTTGTTGTTTGTTTTCGGCAAAGTGCTA
GAGGGGATCATGGTGAACCGATTGAAGGACGTTCTACCCGATGGAAGCAGAT
GGCAATTTGGATTCCGAGAAGGACGCTGCGTTGAGGATGCTTGGAGACACGT
CGTGAGCACTGTTGCTGCCAACCAGGCACAATATATGCTCGGAATCTTCGTG
GACTTCAAAGGAGCCTTCGACCACGTGGAGTGGGATGTCGTGATGAGACGA
CTCATCGACTCCGGCTGCCGAGAAGCCAGCTTGTGGAGAAGCTTCTTCTCTG
GCAGGAGTGCAAGTTTAGTCAGCAGGTATGGTGAAGTGACTGTTCCGGTAAC
ACGAGGTTGCCCTCAGGGATCCATCAGCGGTCCATTTATTTGGAACCTTATGA
TGGACTCACTTCTCCAGCGTCTGGAGCCACTGCGTGGTTTCAGCGCGCTTGC
AGACGACTTGTTGCTTTTCGTTGAGGGTACCCGAATCGTACTGGAGTCGAAA
GGCGAACAGCTCATGTCCGTTGTTGGAGCATGGGAAGTCGGCGTTGCCGTCC
TCACCAGCAAGACGGCGCTGCTGCTGGGGCATTTTGCCCAGAGTAGGCACA
CTACAGTACGGTTTGCAGGAGCAAGCCTGCCGTATGTTGATAAATACCGGTAC
CTTGGCGTTACAGTTGTCGAGCGGTTAAATGTTCTTCCGCATATCAAGTCGCT
ACGTGATCGGCTGACTGGAGTTGTGCAGGCATTGGCACGCGTTCTTTGTGTG
GACTGGGGCCTCAGTCCACGCGCCAGGCGGACAATATATGCCGGACTCATGG

TGCCATGTGCCTTGTTTGGTGCATCGGTCTGGTATGATGGACCCAGCTCTGCC
ATGAGGCATCTGGTCTCCTGCCAGAGGCGAATCCTGCTTGGATGCCTACCGG
TATGCCGTACAGTGTCCACTGTGGCAATGCAGGTGCTCGSTGGCGC

>MICROPIA_LTRLTR#LTR/MICROPIA_LTRLTR

TGTCGTGGCGAAAATAATGAGTATGCGTGTAGTCGCTGTTTACTTCTTCTCCA
TGTTCCCTTTGCTATTATGCGTGTTCCTATTTATGAACACGTGGCGAAAATAAT
AAATGCGTGTAGTCGCCGTTTACTTCTTCTCCATGTTCCCTTTGCTATTATGCG
TGTTCCTATTTATTGTCAATGTGTGAGGATGAATAGATGAATTATCTATGAACG
GGATTTTGCAAAAACGACTTGCGCTGCTTGGTTAGAAAGGGAAAACTATATA
ATGAAAAGGGAATGCCAAAAATTGAGAAGAGACAAAGCAGGCTGCACGAA
GCTGGAGTGAGGGCATTAATCGTGGAGAAGCCAAAGCAGACGCAAGTGGAC
TCGTTGACTGCGCACAGCTGCATAAAATTATATAGTAAAAAGAGATTTGAGCG
ACGCTGATATGGACGGACGGACGGACGCGAGGCCCCTGATATTCTTAACCCG
ACA

>MICROPIA_ILTR#LTR/MICROPIA_ILTR

TCAGAAGTGGGATCTGTGCCACACCCTGCATTTTCTGAGGATCAGTGGCGTG
CAGTAGTGGAAATGCAAAATCGGAATTTGGCTGAACTTGTAAAAATCATGCA
AGTGACGCCGGCACGTGAGCAGCAACCGGTTATGATGTTAAGCTACCCAAAT
TTAACCCTGATGCTGCATGCGTAGAGGCAGCAAAGTGGTGTTCAACAACCGA
TATAATTCTAACTGAGCACCCCCTTAAAGGAAGTAAATTGATCATGGCACTAA
GTAACTGCATGGAGGGAACTGCATCTCAGTGGCTAACACAAATCTCGTACCA
GGGTATGACTTGGCAAGAGTTCCAGGAATTATTTCTGCAGCGCTTTGAAACC
GAAGAGACGCCGGCCGCTACGTTTTTAAATTTACTCAACAGCCGCCCGACTG
CCGCCGAATGTTACGCGGTGTATGCGAGTCGGCTGGTGACGACGCTGACTAC
AAAGTGGCGGAATATGGAAATAGAAGAAATTGCCGTTACAACTGTTCTTGCG
CATATGGCAAACATTGACAGTCGTTTGCAGCGCGTCCTCTTCACATCCAATGT
GCGTACCAGAAGTAAGCTACAGGCGGAGTTAAAAGCGTTTACGTTCGACAA
GAAGCGACATGCTCGAGATGACAACCTTGGACCTGACCAGAAGAACCGTAA
GGCATCGCCAGTTGTATGCCACTTCTGTTCAAAGCCGGGACATCGAATTGCTG
AATGCCGAAGTAAAATGCGACAAGATAGACAGGCGAAACCGCAGCGTGAAA
AATCAAATGTTACGTGCTATCGGTGCGGCCAACCGGGACATTTCTCCAACCA
GTGCCCGAAAAACGGAAGTGCAGCCAAACAAGATGTGACTCAACAGAAGA
CTGTTAACCAATGTTGTGTGACTGAGCCAAAGGGAAGCTTGCATCAACGAGG
TGAGATCTATCCAATTTGTTTCGATTCCGGTGCAGAGTGCTCCCTTATTAAAG
ACGACATTAGCAGTAAGTTATCTGGTAAACGTATAAACAATACTGTAATGATA
AAAGGCATTGGTGGTGGCAGTGTGTGCAGTACATTGCAAATCTTGAGTGAAG
TCACTATAAACGAAAATATTATGGAAATATTATTTCATGTAGTCCCGAACGAGC
AAATGAGGAATGATATTCTGATAGGGCGAGAAATACTTAAACAAGGCTTTTAT
GTAATTTTGACATCCGATAATTTTAAAGTTGTAAAATCAAAAACTGTTAATAAT
TGTTCCGTTACTGAGCGATCGTTTACTTTGTCCGATATTGACACCGAATTAGTC
GACAATGAGAAAGCTCAATTAATTGAGTTACTTGAAAAGCACTCGACTTCAT

TTACCAACGGGATACCTCATACTCGAGTAAATACAGGCGAAATGAAAATCCG
TTTGATTGATCCAACTAAAACTGTTCAGCGCCGACCTTACAGACTTAGCCCCG
AAGAGAGAGAAGTAGTGCGAATGCAGGTGAGCGAATTGATAAGATGTAATAT
TGTTCGCCCAAGTTGCTCTCCCTTTGCTAGCCCCATGTTGCTCGTCAAAAAGA
AGAACGGAACCGACCGTCTATGTGTTGATTTTAGAGAGCTAAACTCGAACAC
GATTTCGGATAAATACCCCTTGCCGCTTATCAGCGATCAAATTGCTAGACTTC
GCGGAGCAAATTATTTCACATGCCTGGATATGGCAAGTGGTTTCCACCAAATC
CCGATTCACCCTGAATCCGTGGAATATACTGCATTTGTGACCCCAGACGGCCA
ATTTGAATTTCTTACAATGCCTTTTGGCCTCAAAAATGCGCCATCTGTTTTCCA
GCGCGCAGTCATAAATGCACTTGGTGACCTTGCTAACTCTTTTGTAATCGTTT
ACATGGACGACATAATGGTAGTATCGCCAACCAAGGAATTGGCTTTGGAAAG
GTTAAAAACTGTTTTGAATGTTCTTACAAAGGCTGGTTTTACCTTTAACCTTG
CTAAATGCAGTTTTCTCAAAACAACGGTTCAGTATTTGGGCTATGAAGTGCGA
GCGGGAGAAATTCGTCCGAATGTGCGAAAGATAGCTTCATTAAGCTCCTTGC
CTCCTCCTCAAACTGTCTCCGGCGTTAGACAATTCATTGGCTTGGCCTCTTAC
TTTCGCAAATTCGTGCCTGGATTCTCCCAACTTATGAAACCATTGTATTCACTT
TCGTCTGGTAGCGGCAAGATTACATGGAGCGCTGAGCTGGAAGAGATCAGAC
TTAAAGTTGTGACGATCCTCACAAATGAGCCTGTTCTGGTAATCTTCGACCCG
CAATATCCTATTGAGTTGCACACTGATGCAAGTGCCTGTGGATATGGAGCGAT
ACTTTTGCACCGTATAGAAAGTAAGCCCCATGTAATCGAATACTTCAGCAAAA
CAACTACCTCTGTTGAATCTAGATATCACTCCTACGAGCTGGAAACCTTGGCA
GTGGTAAAAGCCGTTAAACATTTTCGCCATTACCTAGTTGGCCGTGAGTTCGT
TGTCTATACAGACTGCAATTCATTAAAAGCTTCTCGCACAAAAATAGATTTAA
CCCCCAGAGTTCACCGCTGGTGGGCCTACTTACAATCGTTTAATTTCGAAATT
CAGTATAGAGAGGGTAAGCGTATGGCTCATGTGGATTTCCTATCAAGAAATCC
TTTATCACCCGAACACATTTTGTCAATAAACAAGATTCCCGAAAAACGAGTA
AATCTGTCTGAAATTTCAAGTACTTGGCTTCTTGCTGAGCAACGGTTAGACCC
TGAGATAATAGAAATTGTTAACAAATTGGAGTCAGATGAATTGGCCGAAAAC
TTGGCCAAAACGTATGATTTGCGAAAAGGTGTATTATATCGCAAGGTCCAAAG
ACGAGGTAGAACAAGTTATTTACCAGTTGTACCCAGAGCTTTCAAATGGTCA
GTAATTAACCAGGTACACGAGTCGATAATGCATTTAGGGTGGCAAAAGACAC
TTGATAAAGTGTACCAGTATTATTGGTTCGCTAAAATGAACAAGTATGTTCGA
AAATTTGTTTCAAACTGCATAACTTGTAGATCAGTGAAATCATCTTCCGGGAA
GGTTCAGGCGGAACTTCATTCCATTCCGAAGACAAGTATACCGTGGCACACC
ATCCACATAGATATAACGGGGAAATTAAGTGGCAAGAGCGATTTGAAGGAGT
ATGTCATTGTTCAGATCGATGCCTATACAAAGTTTGTTTATCTGTATCACACCT
TAAAGATAGATGCCGAAAGCTGTGTTAATGCTATGAAATCTTCCATATCCTTAT
TTGGAGTACCAGATCGCATCATCGCCGACCAGGGCAGATGTTTTACTAGCTCT
AAGTTTTCAGAGTTTTGCGTATCGCAGAAAGTTGAACTTCACTTGATTGCTAC
GGGAATGAGCCGTGCAAATGGGCAAGTGGAACGGGTGATGGAAACACTGAA
AAATTTGTTGTCAGTGGTAGAATCAAGTCAACGATCGTGGCAGGACGCACTT
GGCGAAGTCCAACTTGCACTGAATTGTACAATTTCTCGTGCCACTGAGGCAA
GTCCGTTAGAAATGTTAATTGGTAAACAGGCTCGACCCCTTGGATTAGTTCCC

CCATGTGAGACCGAATGTGAAATAGATTTGGCAACTGTTAGAGCTCATGCGA
CAGAAAATATGAATTCCTTAGCGTCTTACGACAAATCCCGATTTGATAGCAGT
AGGGCAGCCGTTGACAAACACCACGTAGGTGACTATGTGCTATTGAGGAATG
AAGAAAGACACCAAACTAAGTTAGATCCGAAATTCAGAGGACCGTTTTTGGT
AACTGAAGTATTAGAGGGTGACAGGTATACACTAAAGTCGTTGACGAGTAAC
CGATCGTTCAAGTATTGCCATGAAGATATACGTAAAATGCCGGATACAGAAAT
CCCGAATGAGTTAAACGAGAATGTAGAGCAATAGCTGAAATATAGAAACAGT
TGAATGAAAAGAAAAGCCCGCCAATGAGTTCTTTTGTGAACGAGAGACCGC
GGGGCGGGGGTATCCGTCTAGGTGAGACGATGAATTGTGAGTTATCCGTCTA
GGTGAGACGATGAATTGTGAGTTATCCGTCAGGTGAGACGATGAATTGTGAG
TTATCCGTCCAGGAGAGACGATGAGTTTGGATTGAATTAATAATCAAGTGTGT
GTGAACTGGCGGAAGATCGATATATAGAAATCGATAAATGATAATGTTAAGAT
AAGTTGTGAGCTGATGTATTACTGATCAATGGAACTGAATATGAAAATAGAAT
AAGTTATCCCAGCAACAGTGAAATAAGAGCTGTTTTGTTTCTTCACAGAATTA
AGATTTAAGAAATACACCTAATAAAGTCAAACTAATGAAATTAAATGTTATTG
AATAGTGATGAAAGTATGTGATCTTGATATCTTGGTATCTCGGTATCAAAAGCT
TACACGAGGACGTGTAAATGTCAGAATGGCCG

>LTRMDG3_LTR#LTR/LTRMDG3_LTR

TGTAGTAGGCTGCACCTTCTACCCTCTTCCTTTACTCTTAGTCATACATACCTA
ATTATACATAGCCAATCTAGTCATAAGCTTATACACTCATACACCCATCCTTAA
CATACAAATATTATCGAGAAACTTATCGACTAATCGACTCGCCACTCTGCAGA
GAGCGCGGCAGTCAGTCGCTGTTGAACCAAGCTAAAGGACAGATCAAAAAT
AAAAGAGACACGTGAAATTGTATTAGAATATTAACTTCTGTAAACGGCGGCTA
AA

>BLASTOPIA_LTRLTR#Unknown/BLASTOPIA_LTRLTR

TGTAACATGAGTAAGGCTGAAGGCTGGCAACAACCCGGTTGGCAGCGCTGT
TGAGCAGCAACATGATTGTCGGAAATCCAAGTTATCGACAATCAGTCATCGA
AGGACGATCGCAGGCAGCAGTAGAGGCGAGTGGAAGTCAGCGTTGCAGTCA
GTCGAGTTCTCAGCAGCAGTCGTTCGGTCCACAAACTAAGAAATACTTTATAT
AATTACCGCATTTAGAATTAAACTAATAATTAAATTAATAATAAACAATAATAAT
AAACAATCTTACA

>ACCORD_ILTR#LTR/ACCORD_ILTR

GGCGCAGCCGGACTAGAACTAAAAGGATTAACAACGCCTTTCACGTTCTTCG
TTCCACTTCTGTAAACGGGCAAGTGAGTGAACAAGTGGCAGAAAAAAAAAA
ACACAAGTTTGAGGAGCCAAGTGTAAATATGTGGTTCTCAAAAAAAAGAGA
AGAAAAGGAAAAAACTTCACACAAACAAGTTCAACAGTGACATACAGTATA
ATTACAAACTATTGTGACCGAATATGTGCACATCAATCGCTGAAAGGGCACCG
GGATCACACATAAAAAAAATGTCTTTACATTCAAAGTGACCGAATATGTGCAC
ATCAATCGCTGAAAGGGCACCGGGATCACTTATGACATACATAAAAAGTTAA
CATCATTCAGTGCTATGGCCAAATATGTGTACATCAATCGCTGATGAGACACC

GGGACCATACATAAATAAGCAAAACAAATAAATCTAGTTCAATGTTGTGGCCG
AATATGTGTACATCAATCGCTGAAAAGGCACCGGGACCACAACATTGAAAGA
AATTAAACAATAAATCAGTAATAAAATCACTATGTGTGTCAGGTTGAACCTTT
CCTTGAAAGCCATATACTTGTTCGCGAATGCTCCCAATTGAGAAACATTTGTC
AAATTACAAATACACTCAACTTACTACTTCGGATTGTGTTACTGCCATGTGTTC
ATTTACTTATACAAACTGAATATAAATTTTTTATACTTACTAATGTACATAATATA
TTACAGTCAAACATTGGAGCCAACACTTACTTAGAAAGAAACAAACGGATTC
ATATTATAAATACACACTACATTTTAAGATTATCTATATATAAGATTACAATTACT
TAATTACACCTTATTATTTAAGCTAAAACATTACACTAAACATATACACAACAT
ATAGGTACACGTATACAAAAGTATATAAGCACAATAACACATATACCATATTTT
TAAGTACAACACACAGACTACACAAATCAAAAACACCTACATGCCAAAGAG
AATTCCGTTTAATACCCAAAGTAAAAGAAGACTTGGACTTAGAAGTGGAGGC
AGCCTTCCTCAAATTAGGGAACAAGTAGAACAATCCACCAACAGCAACAAC
ATGGACTCGGGAAACGCATCAGCCCCTTTAAGTCCGACTACTTCCGCTACTTC
CACAGTTATTAACACCAACCTTAACCCAACCGACATTTTGGCTTTTATCGAAC
AACTCCCCACTTTTGAAGGTCATCCTAGCAATCTCGACAAGTTCATAACTAGC
GCTGAGGAACTGCTGTTCCTCATTAGATCAGTAGACAAGACCCCTTATGGAC
AACTACTTCTAAGGGCCGTCCGCAACAAAATCGTAGGAAAAGCAGACGATG
CCTTGACCCTTTGCGACACTCGATTGAACTGGGACGACATCAAGACTAATCT
CAAACGACTCTACACCAGCAAGAGAACCGAAGCGATGATACTTAGGGAAAT
ACAGACCCTCCCAAATGACCTTACAATGGGAAAACCTTTTTACAGTATAATTA
AAATGAGAAACGAACTTATAACAATAGCTAAAGACATGGATACCACGGGTAA
TGCACTCGCAACTAAACGTACCCTCTACGATGATATATGTCTCAACGCATTTAT
CATCGGATTAAAGGATCCACTAAGAACAATCATAAGAATTAGGAACCCGGAC
ACTATCGAAAAAGCTTACGAATACGGACAAATGGAACAAAGTTTTTTCTTCC
AAAACTCAAACAGACAAGTAGAGGGCCGTCGCCGTGACAATCCAACGGACA
GAGGACGCCCATACCCAAAAAATAACCAACAAGAACCGAAACTATCAAATA
ATAATGGTCGCAACATTTCGCACCAACAAACCAGTCATCAGCAAGGGGGACA
ATCGATGAGAAATCAAAACACATCCCACAACTACGAAAAACCAACTTCAACA
CACCAACAATACAGAATCAATACTAACACAAATGTAAATTTGTGCAACATAAA
TGACGACACAAATTTTCCGCAAAGAGCCTCGGAAGACCAGTCGGATTCATAA
CTAAAAAAGCCCACGGGTCATCATTACCTTATGTAATGCTATCCCCAACATTT
GTCCCTTCACAACCACTTAAATTCCTAATAGACACTGGGTCCACTTATTCATTT
ATAAACCCGGCCCTCATTACAGAAGACAATATAAAAAAACTTAATTCAAGCAT
AGCCATTCATACAGTACTTAATACATTCCAAATTACGGAATTCACAGACACTAT
TAAATTTAAACAATTCAAAGAATTGTCAAATCAGAAGTTTCTTTTGTTCAATT
TTCATCCACACTTCAATGGCCTCTTAGGCATGGATCTTCTATCCACTCTGAATG
CAAAAATAAACATCGCGGATTCCATTTTGGAAACACCTGAAACCGCAGTTCC
AATTTTGACACGACCAAACCCTGTAGACATTTTGCATACTGTACCATCAAATA
GCAAGGTAAGGCTTCCACTACCAGTAAACTGCATGCAAGGAGACTTTATATA
CGGGACCACAGATTTTGACAACCAACTATCAATAACAGGTGGACTGTATACT
GCAACCGCAGGTATAGCCTATTTCGAAGTCTGTAACAGTTCAGACTACAACC
AAACACTTTATTTAGAAGAACCATTAATAGCAGAAGAACTCTCTTTACAACAC

TATGGACTTTTACACTGCATGTCTACAATGGCGGATACTACTCAGACGGACAC
CCAACAAGAACTTAATATTAAGACAGAACATTTAAACCAAGAAGAAAAATTT
AATCTAATCAATCTATGCAAAACCTTTAGGAAACTTTTCCATTATGAAGAGAG
CCATTTGACCTTTTCTAATGTCGTTAAACACTCCATACCAACAGTAGACAATG
TTCCGATATTCACTAAATCATACCGTTACCCATATGTACACAAAGAAGAAGTT
CGTAAACAAATATCAGAAATGCTTCGACAAAATATTATTAAAAACAGTCACTC
TCCTTGGAGTGCACCCGTAGGGATCGTACCCAAAAAAGCCGACACTACAGGC
AAAGAAAAATGGCGGTTGGTAATAGATTTTCGAAAATTAAACGAAAAAACCA
TCTCCGACCGTTATCCGATACCTAACATAGCAGACATACTGGACCGAATAGGT
GGAACAAAATACTTCACAACAATAGACTTGGCAAGTGGATTCCATCAAATTG
AGATGAATCCACAAGACGCGAACAAAATAGCTTTCACAGTCGAAAATGGGC
ATTACGAATTCACAAGAATGCCCTTCGGTCTGAAGAACGCACCAGCCACTTT
TCAACGTGTGATGGACAACGTTCTAGGAGACCTTATAGGTAATGTTTGTCTGG
TTTACCTAGACGACATTATTATATTCTCACCCTCACTGCAGAAACACATTAGCG
ACATTAAACTTGTTTTCTCTAAGCTACTGAATGCGAATCTAAAAATCCAACCT
GCTAAGTGTAACTTCTTAAGGAAAGAAATTGACTTTTTGGGACACATTGTTAC
TCAGGAGGGAGTCAAGCCCAACCCAAACAAAATACAAGCTATTAAAGACTTT
CCCTGCCCCAAAACCATCAGGGAAATCAAGTCATTTTTGGGATTACTTGGGTA
CTATAGGAAGTTTATCAAAGATTTCGCAAAAATAACAAAACCTATAACAAGAC
AATTAAAAGGAAAAAAATCAATAGTAATAGACGACGAATTTAGGAAAGCAGT
CGAAATTTCAAAGAATTTATTATGCAACGACCCAATACTTATATTCCCCGACTT
TACAAAACCATTCACGTTGACGACCGACGCAAGCAACTACGCCATAGGGTCA
GTGCTATCACAAGGCCCCGATACCAACGACAGACCCATATCCTTCGCTAGTAG
AACGCTATCAGACACAGAGGTTCGATATTCCACCATAGAAAAAGAAATGCTA
GCTATTATTTGGTCAGTAAGTCATTTTAGGCCATACCTTTTTGGCCAGAAATTT
AAAATAGTCACAGATCACAAACCATTAGTCTGGTTAGAAAGCTTTAATGGTC
AAAACCCTAAACTACTTCGGTGGAAAACTACCCTAGCCGCATACGATTACGA
AGTAGTGTATAAAAAAGGCAAACAAAACGTGGTCGCTGATGCGCTTAGTCGA
ATAGAACCAAATTTAAACATAAACGAAGACCCACAAAAAATCCCAGTTGTCC
AACAACCATTAAATCATTTCAATACACAAATTGTGTTCCACATTGGGGAAAAG
TCTTCAGTACAAATCACAACACCATTTACGTACAAAACAAGACAAGTCATTT
CAGAACCTACATATACCTTTGACACTATTTCTAACATCTTGCAATCAATTCTTA
AACCAAACAAAATGACTGCAATATTTGCCCCAGATCAAATCTTTTTGCTCATA
GAGGAAGCGTATAACAACTACTTCTCGGTGAACGACTCATATAAAATCTCCCG
TTGCAAACTGTTCTTACCCGAAATAACCGAAACGGAAGACCAAAAAACAAG
AATACTTACATACCACTTAAAGAACAACCACAGAGGTATCGACGAAACCTTT
CAACACCTAAAAAGAGATATTTATTTCCCCCGCATGAAAGATATTATTACGCA
AGTAATAAAAGACTGTGACATTTGCCTTACACTTAAATACGACAGGCAACCC
CATAAACCAGCTATGCAATCACCACCTGCCCCACCAGGACCTTTAGAGGTGA
TCCACATAGATATTTACTTCGTCAATGGTACTTATAATCTAACTGTCATTGACA
AATTTTCAAAGTTCGCACAAGCATACCCGCTTGATAACAGAAACTCCATAAA
AATCATTGCCGCCTTGTCTCAGTTCATGAGCAACTTTGGCATACCCAAAAAAC
TTGTTTTCGATCAGGGAACAGAATTTTCCGGAAACCTGTTTAACGATTTCCTT

GCTCAATACGATATACTCCAACATACTACTTCATTCCAACAATCGACTGGTAAT
TCACCCGTCGAAAGACTTCACTCTACGTTAACAGAATTGTACAGAATAGTTAT
GAACCGTCGAAAAGAATTACACCTACAGTGCGAACATACCGAAATACTTAGC
GAAGTATTAACAACCTACAATAACGCGATACATTCTAGCACAAATCACACCCC
TTTCGAACTCTTTCACGGCAGGACACACATATTTGGAAAAACCATAACTTATG
ACAATCATCATGACTACCTCTCAAAACTAAATCAATTCAGACAAACACTATAC
CCCCAAATACAACAACACCTACAAAACACCACCGATAAGAGATTGGCTAAGT
TAAATAAGGACAGGGAACCGCCAATTCTAGTTGAGGAAAATAATACTATATAT
AGGAAGGAAAACAGAAGAAACAAAATAACACCTAGATTTTCTCTACACAAG
GTAGAAAGAGACAGAGGGATAACACTAATAACTACCAGAGGTCAGAAATTAC
ATAAACAAAAAATTAGGAAACACATTAAAAGAACTGTGAACACCCAGTCCA
AATCAAACACTAACACTTAATGAATTCAACTAAACATGAAAATGTAACAATTA
AAAGACAAATTTGATAAACAGACAGGGACATTATTATCAGGATCATATATAAA
ATAGAACACGTAAATAACAAACAAGTTTCGATATAGAAATTTTTCGAAAATTA
CCAGAACCAAATTTTTAAAGAGCAATAACGTGGCCGAAAGCTTACTTGCTAG
TAAGTTAGGCCTCATTTTCAAATTTATGTTTAATACACAACAATTAGAAGACAT
ACGAATTTTATTTAATAAGCAAAATATAACCATAGAATTGGATGAACACATTAA
CAACTTTTGTAACCATCCTGTTAAACTCTTACTTGCAACACCAAACTATTATTT
AAGGTTAAAAATCCATTTTTCAACTAGCAAATGTATCATTTCATTCAAACAATT
CCCTTACCTATACATTGTTCATACTTCATTGTCTCCCACGACAAACTTACCATC
GAGATACCACCAATGGAACGAATAGAAAATAACAAAACATTGAAAGTCCTAT
CACTCGAAAAACTGCACCTGGAGGCATTGGCAACAACCGGGAAAATGGACT
ACATAACTCAACAATACTTTACCGCTACGTACCTTGCATTGAGCATAGTACTA
ATACTTGTGATCTGTCTCCGAACCTGTAAAAGGCAGAAAACGATTTTTACCCA
GCAACCCACCCACACACCTCACGTTGTCGAGTCGGCTATCCCTTCGTTATGGC
CATCTCTCCGCACTAGGGGGGGAGG

>U1#snDNA/U1

ATACTTACCTGGCGTAGAGGTTAACCGTGATCACGAAGGCGGTTCCTCCGGA
GTGAGGCTTGGCCATTGCACCTCGGCTGAGTTGACCTCTGCGATTATTCCTAA
TGTGAATAACTCGTGCGTGTAATTTTTGGTAGCCGGGAATGGCGTTCGCGCCG
TCCCGA

>U2#snDNA/U2

TATCGCTTCTCGGCCTTATGGCTAAGATCAAAGTGTAGTATCTGTTCTTATCAG
CTTAACATCTGATAGTTCCTCCATTGGAGGACAACAAATGTTAAACTGATTTT
TGGAATCAGACGGAGTGCTAGGGGCTTGCTCCACCTCTGTCACGGGTTGGCC
CGGTATTGCAGTACCGCCGGGATTTCGGCCCAAC

>U4#snDNA/U4

AGCTTAGCGCAGTGGCAATACCGTAACCAATGAAGCCTCCCTGAGGTGCGGT
TATTGCTAGTTGAAAACTTTAACCAACCCACGCCATGGGACGTGAAATACCG
TCCACTACGGCAATTTTTGGAAGCCCTTACGAGGGCTAA

>U5#snDNA/U5

ATACTCTGGTTTCTCTTCAATGTCGAATAAATCTTTCGCCTTTTACTAAAGATT
TCCGTGGAGAGGAACACTCTAAGAGTCTAAAACTAATTTTTAGTCAGTCTT
GTCGCAAGACTGGGGCCA

>U6#snDNA/U6

NGTTCTTGCTTCGGCAGAACATATACTAAAATTGGAACGATACAGAGAAGATT
AGCATGGCCCCAGCGCAAGGATGACACGCAAAATCGTGAAGCGTTCCACATT
TTT

>H3#Histone/H3

ATTGTGTTTTCAAACGTGAAGTAGTGAACGTGAACTTTAGTGAAACCCAAAT
CGGAGATGGCTCGTACCAAGCAAACTGCTCGCAAATCGACTGGTGGAAAGG
CGCCACGCAAACAACTGGCTACTAAGGCCGCTCGCAAGAGTGCTCCAGCCA
CCGGAGGTGTGAAGAAGCCCCACCGCTATCGCCCTGGAACCGTGGCCTTGC
GTGAAATTCGTCGCTACCAAAAGAGCACCGAGCTTCTAATCCGCAAGCTGCC
TTTCCAGCGTCTGGTGCGTGAAATCGCTCAGGACTTTAAGACGGACTTGCGA
TTCCAGAGCTCGGCGGTTATGGCTCTGCAGGAAGCTAGCGAAGCCTACCTGG
TTGGTCTCTTCGAAGATACCAACTTGTGTGCCATTCATGCCAAGCGTGTCACC
ATAATGCCCAAAGACATCCAGTTAGCGCGACGCATTCGCGGCGAGCGTGCTT
AAGCTGACACGGCATTAACTTGCAGATAAAGCGCTAGCGTACTCTATAATCGG
TCCTTTTCAG

>H4#Histone/H4

TTAGTTCTTTAGTGACTTTCGTGCTGTGCGTGTATAATAGTATAGAACAGTGA
AAAATGACTGGTCGTGGTAAAGGAGGCAAAGGCTTGGGAAAGGGTGGCGCC
AAGCGTCATCGCAAAGTGCTGCGTGATAACATCCAAGGTATCACGAAGCCTG
CTATCCGCCGTTTGGCCCGTCGAGGCGGTGTGAAGCGCATATCTGGACTCATA
TACGAGGAAACGCGTGGCGTTCTGAAGGTTTTCTTGGAGAACGTAATTCGTG
ATGCCGTGACCTACACGGAACACGCCAAGAGGAAGACAGTTACAGCCCTGG
ATGTTGTGTACGCTCTGAAGAGGCAAGGCCGCACCCTCTACGGATTTGGCGG
TTAAAAAGTGTACATTCTGTGTACCCCTATTAAGCAATCGGTCCTTTTC

>H1#Histone/H1

AAAAGTTTACACTTCAAGCAAACTTCGACATAGTAAATGACTGATGTCAGTA
GCATTGTTAAAGTGCTCTCCTCCTCGATTCTCATCAGAGCAAAGGAGGTTGGT
AGGCAGCGCGCGAGCCATTTTTAACAGAAAAAAAGTGTTCTCAGTGAAAAA
AAGATGTCTGATTCTGCAGTTGCAACGTCCGCTTCCCCAGTGGCTGCCCCAC
CAGCGACAGTTGAGAAGAAAGTGGTCCAAAAAAAGGCATCTGGATCTGCTG
GCACAAAGGCAAAGAAAGCCTCTGCGACGCCGTCACATCCGCCAACTCAGC
AAATGGTGGACGCTTCCATTAAAAATTTAAAGGAACGTGGCGGTTCATCACT
TCTGGCAATCAAAAAATATATCACTGCCACTTATAAATGCGACGCCCAAAAGT
TAGCGCCATTCATCAAGAAGTACTTAAAATCGGCCGTGGTCAATGGAAAGCT

TATTCAAACTAAGGGAAAGGGTGCATCTGGATCTTTCAAACTGTCGGCCTCT
GCCAAGAAGGAAAAGGATCCGAAGGCAAAGTCGAAGGTTTTGTCTGCTGAG
AAAAAAGTTCAAAGCAAGAAGGTAGCCTCTAAGAAGATTGGTGTCTCCTCC
AAAAAAACTGCCGTTGGGGCTGCTGACAAAAAGCCCAAAGCTAAGAAGGCT
GTGGCTACCAAAAAGACTGCCGAAAATAAGAAAACTGAGAAGGCAAAAGC
CAAGGATGCCAAGAAAACTGGAATCATAAAGTCGAAGCCCGCCGCAACAAA
GGCGAAAGTGACTGCAGCGAAGCCAAAGGCTGTAGTAGCGAAAGCGTCAA
AGGCAAAGCCAGCGGTGTCTGCAAAACCCAAAAAGACGGTGAAGAAAGCA
TCGGTTTCTGCTACCGCCAAGAAGCCGAAAGCGAAGACTACGGCTGCCAAA
AAGTAAATTGTGAAAAAGTGCAGTATTTGGTACATGTTCGCAATTAAAATTTT
AGATTTATGATTTATAGATCTGAAATTTGTTTAAACAAGTCCTTTT

>H2A#Histone/H2A

GCATTCGTGTTCCGTGTGTAAAGTGAACTAAGTGAAATAAACGCAAAGCAAA
ATGTCTGGACGTGGAAAAGGTGGCAAAGTGAAGGGAAAGGCAAAGTCCCG
CTCAGACCGTGCCGGTCTTCAATTCCCTGTGGGCCGTATTCACCGTTTGCTCC
GGAAGGGAAACTACGCAGAGCGTGTTGGTGCAGGCGCTCCAGTTTACCTAG
CTGCCGTAATGGAATATCTGGCCGCTGAGGTTCTCGAGTTGGCTGGCAATGCT
GCTCGTGACAACAAGAAGACTAGAATTATTCCGCGTCATCTGCAACTGGCCA
TCCGCAACGACGAGGAGTTAAACAAGCTGCTCTCCGGCGTCACAATTGCAC
AAGGTGGCGTGTTGCCTAATATACAGGCTGTTCTGTTGCCCAAGAAGACCGA
GAAGAAGGCCTAAACGTTTCAAAGGCTAAGCTAAAAACCTACATGTACATAA
AATCGTCAATCAAACCGTCCTTTTCAG

>H2B#Histone/H2B

TTACGATAAGTGAATTGTGTTTGTGAAAATATAAGTAACGTGAACAATGCCTC
CGAAAACTAGTGGAAAGGCAGCCAAGAAGGCTGGCAAGGCTCAGAAGAAC
ATCACCAAGACCGACAAGAAAAAGAAGCGCAAAAGGAAGGAGAGCTATGC
CATCTACATTTACAAGGTTCTCAAGCAGGTCCATCCTGACACCGGAATTTCGT
CGAAGGCGATGAGCATAATGAACAGCTTTGTAAATGATATTTTCGAGCGAATT
GCTGCCGAAGCGTCTCGTCTAGCTCACTACAACAAGCGCTCGACCATCACCA
GTCGGGAGATCCAAACGGCTGTTCGCCTGCTTTTGCCTGGAGAGTTGGCCAA
GCATGCTGTCAGTGAGGGAACCAAGGCTGTCACCAAGTACACCAGCTCTAA
ATAATTTTCTCCTGAGAATGCGGACAATAATCCAAAACGGCCCTTTTCAGGGC
CACAATGTGTTATACCAAAGAAATGCATTTTTCAACCACCAATCATTGAATAT
GAATTTACAAATAAAACTTATTTACCCGCAATATGGGTAATTGGGTAATGTTCA
AATAAATACAAAAAAAATCGGG

>Sat_1688_357

TTDTTTGATGACCGAAATTTGGAAAAACGGATTTTGCCAAAAAGTTGATATTT
ACAAACGGAATTTTTGTCATAACTTGGCTAAAAATGGTCATATAGATGCAAGA
ATAACTGTTTTGAGAAGCTAATTACCAGCGCTAACGATCCCTATHACTTTTTG
AAGGATTTAGGGAAATCAATTTTTGGGTCAATTTTCGTATTTTTTGTAATGACC

CGCCATAAAAATTTGVAAAGTATGGCAAAAAAACATAATTTCCATTTTTGAAC
ACATTTTAATTGTAAATTTAATTACGAGCTCAGCGAGGTATGACATTCCATATT
CAGACAATTATTTTTAAAGTTGTGGCAAAAAAACTGA

>Sat_1688_179

AAATTTCAAATCAAACTGTGTTCAAAAATGGAAATTAAATTTTTTGGCCATAT
TTTGCAAATTTTGATGACCCCCCTCCTTACAAAAAATGCGAAAATTGATCCAA
AAGTTAATTTCCTAAATCCTTCAAAAAGTAATAGTCTGAATATGGAATGTCATA
CCTCACTGATCTCTTAATA

>Prodsat

AACATAGAAT

>AAGAT

AAGAT

>Responder

ACACAAAAACAATAAATTGACTAAGTTATGTCATTTTAAGCGGTCAAAATGG
GTGAATTTCCGATTTCAAGTAATAGACGAACTGAAGATACCTTCTACAGATTA
TTTAAACCTGGT

>Dodeca-11

ACCAGTACGGG

>Dodeca-12

ACCGAGTACGGG

>Sat_307

TGCATTACTCTCGCATACGGGATCATTATTCACTCTATAACATGAGAGAGAGTG
TGAGAGGCAATCTAAATATCTCTCTTGCTCTCTCTCTCTCTCTGTCCTGCAT
TGTTATTTTGGGTTTGCGGTAATATAAGACAGAGAATGAGAGGCAGTCATTAT
ATATTCAAGCCTTTTTTCAGCGGTAATTTTTTTCTGCATTTAACGGGGCAATGA
TTTTTTTCTGATTAGCAGCAATTTGAATATATTATCATCTCGTTCGTCCGTCGCC
ATTTTGTTTGCCAAAAAAACGAAAAGAACAATATGTT

>AAGAG

AAGAG

>AATAT

AATAT

>Sat_597

TTTAATATTTTAATATAACTTTATTTTAACTAGTTCCCTTTGCAAGCAGGCCACC
GAATGACACCTCGAAAGTCATTTGTGCGAATGGGCCGTAACAGATTCCATCG
TATATACTGAACGTACATGACAGAAAGGGATGACAATGTTGTGCTTAATTCTA
ACGGGGATTTGTGCTTATTACTCCATTGCTTTTTATTCTACTTTACTGTGGGTCT
CATTCGTATCAATTATGTCATGTACAATATATTGGGACTCATATACATATTTCTTA
CTACAATAGTAAGATGGAAATAAACACAAAAATGTTTATAAGTTTACATAAGA
ATTGTAGATATTGCAAGAAGAATATTCATTATGAAAATAAAAATTTAATAATAG
AATAATAAATAAAATAATAATAATATTTAAATATTTATTATTTAGCTCGGAGTAC
CTCAAACTAAACTTTATTAAATTACGGCATGCATTGTGAAACTAAAAAAAATC
AACGTTCAACGGCGGTGTTTAACTTTCCTTTTTCATTCCATTGCGGCAGCTGT
TCTTGCAAATTTGTAATGATCACTTCTGATGTTGCACAAGACGAGGACACACT
CTTGC

>Sat_228

CCCAACTTCTTTAGTAACTTCTGTAACGGGTAGTGGGGATAAATCTCCTCTCC
ATCCTGAGGAAAAACCAAAGTCGCCTGAGAAGAAAGACGAAAAGGTCCTAG
CTAAACCAGACGACAGTTCCAAGTCGGTCGTTGAGACTGATAAGCCCAGTCC
TAAAGAATATTCGGACGATGAAACTGATGACGAAATAGAAATTCCCAAGCCA
CTAGACAAACCTATTAGCCA

>SAT-2_Dmel

CAACAGAAGAGAGCAGTTCATTTCAAGAATCCAGTGCAGAAGAAAATCAAA
GTTCAAAAACAAAGAATATATTTTCGAGTCAGAGTTTAAATGAAGACAACAT
TTCTCAAGAAGATACAAGGACACTACCAATATCTGTCCCACAATCAAGTACA
ACAGCAAATAGATTACTTACAGGTTCGAGTGCAGAAGAGCAAACAGCTCAA
GAGGAGACATCGGAACATTCAAAATCCTTACCTCAATTAA
