# Supplementary material for: Comparative genomic analysis and epigenetic profiling of the Drosophila melanogaster B chromosome
Source: Genetics. 2026 May 5;233(4):iyag113. doi: 10.1093/genetics/iyag113 (PMC13439713; doi:10.1093/genetics/iyag113)
Supplement: Supplementary file 2 [file iyag113_supplementary_data.zip › Supplementary_Table_1_GENETICS-2026-309266.pdf]

Supplementary Table 1. Oligos used for probes production for each of the repetitive sequences mapped by FISH.

Repetitive sequence	Oligo	3' marker
Sat_1688	5'TTTTCCAAATTTCGGTCATCAAATAATCAT	Cy3
Prodsat	5' TTCTATGTTATTCTATGTTATTCTATGTTA	Biotin
AAGAT	5' AAGATAAGATAAGATAAGAT	Cy3 or ATTO 590
Responder	5'GTCAAAATGGGTGATTTTTCGATTTCAAGT	Cy3
dodeca-11	5'ACCAGTACGGGACCAGTACGGGACCAGTACGGG	Biotin
dodeca-12	5'ACCGAGTACGGGACCGAGTACGGGACCGAGTACGGG	Cy3
Dodeca-12	5'CCCGTACTCGGT CCCGTACTCGGT CCCGTACTCGGT	Cy5
Sat-307	5'TAGCAGCAATTTGAATATATTATCATCTCGTTCGTCCGTCG CCATTTTGTTTGCCAAAAAAACGAAAAGAACAATATGTT	Cy3
AAGAG	5' AAGAGAAGAGAAGAGAAGAG	Biotin
AATAT	5' AATATAATATAATATAATATAATAT	Biotin
Sat-597	5'TTCATTCCATTGCGGCAGCTGTTCTTGCAAATTTGTAATGA TCACTTCTGATGTTGCACAAGACGAGGACACACTCTTGC	Cy3
Sat-228	5'CCCAGTCCTAAAGAATATTCGGACGATGAAACTGATGACG AAATAGAAATTCCCAAGCCACTAGACAAACCTATTAGCCA	Cy3
SAT-2_Dmel	5'GAGTCAGAGTTTAAATGAAGACAACATTTCTCAAGAAGAT ACAAGGACACTACCAATATCTGTCCCACAATCAAGTACAA	Cy3
AAGAGAAGAT	5'AAGAGAAGATAAGAGAAGAT	AlexaFluor488
AAGAG	5'AAGAGAAGAGAAGAGAAGAGAAGAGAAGAG	Unlabeled
AAGAT	5'AAGATAAGATAAGATAAGATAAGATAAGAT	Unlabeled
AAGAGAAGAT	5'AAGAGAAGATAAGAGAAGATAAGAGAAGAT	Unlabeled
