# Supplementary material for: Comparative genomic analysis and epigenetic profiling of the Drosophila melanogaster B chromosome
Source: Genetics. 2026 May 5;233(4):iyag113. doi: 10.1093/genetics/iyag113 (PMC13439713; doi:10.1093/genetics/iyag113)
Supplement: Supplementary file 2 [file iyag113_supplementary_data.zip › Supplementary_Table_2_GENETICS-2026-309266.pdf]

Supplementary Table 2. Antibodies used in immunostaining analyses, including their type, host species, catalog number, and producer.

**Antibody**	**Type**	**From**	**Cat-number**	**Producer**
H4K5ac	Primary	Rabbit	07-327	Sigma-Aldrich
H3K9me2	Primary	Rabbit	07-441	Sigma-Aldrich
H3K9me1	Primary	Rabbit	SAB5600008	Sigma-Aldrich
Anti-RNA pol total	Primary	Mouse	05-623	Sigma-Aldrich
Anti-RNA pol S2p	Primary	Rabbit	ab5095	Abcam - USA
Anti-RNA pol S5p	Primary	Rabbit	ab5131	Abcam - USA
Anti-Mouse - Alexa Fluor 488	Secondary	Goat	SAB4600388	Sigma-Aldrich
Anti-Rabbit - Alexa Fluor 488	Secondary	Goat	ab150081	Abcam - USA
