# Supplementary material for: Comparative genomic analysis and epigenetic profiling of the Drosophila melanogaster B chromosome
Source: Genetics. 2026 May 5;233(4):iyag113. doi: 10.1093/genetics/iyag113 (PMC13439713; doi:10.1093/genetics/iyag113)
Supplement: Supplementary file 2 [file iyag113_supplementary_data.zip › Supplementary_Table_3_GENETICS-2026-309266.pdf]

Supplementary Table 3. Estimation of abundance based in K-mer analysis (Wei et al. 2014; 2018) for low-complexity satDNAs in all libraries studied (0B-WT, 0B-TM3 and +B) and the sequenced pulsed field electrophoresis B (PFGE-B). Note that the ratio between B+ libraries and 0B libraries falls in the same pattern observed when the abundance is estimated through RepeatMasker.

**satDNA family**	**0B-WT Genomic abundance (%)**	**0B-TM3 Genomic abundance (%)**	**B+ Genomic abundance (%)**	**PFGE-B Genomic abundance (%)**	**B+/0B-WT**	**B+/0B-TM3**
AAGAG	0.0783	0.0706	0.5968	0.8409	7.62	8.46
AAGAT	0.0177	0.0163	0.2692	10.7288	15.21	16.52
AATAT	0.0133	0.0111	0.0331	3.6939	2.49	2.98
AAGAGAAGAT	0.0003	0.0005	0.0103	0.3446	29.73	22.67
Prodsat	0.8593	0.5339	0.7230	0.2019	0.84	1.35
dodeca-11	0.0031	0.0046	0.0377	0.0093	12.20	8.14
dodeca-12	0.0006	0.0009	0.0072	0.0019	11.87	7.91
