# Supplementary material for: Comparative genomic analysis and epigenetic profiling of the Drosophila melanogaster B chromosome
Source: Genetics. 2026 May 5;233(4):iyag113. doi: 10.1093/genetics/iyag113 (PMC13439713; doi:10.1093/genetics/iyag113)
Supplement: Supplementary file 2 [file iyag113_supplementary_data.zip › Supplementary_Table_4_GENETICS-2026-309266.pdf]

Supplementary Table 4. The table displays the average genome proportion (%) and divergence (K2P %) of TE superfamilies across the three genomes (0B-WT, 0B-TM3 and +B) of *Drosophila melanogaster* and the sequenced pulsed field electrophoresis B (PFGE -B). The last two lines provide a summary of the total abundance and average divergence for all TEs examined.

TE Class/Superfamily	0B -WT Genome proportion (%)	Divergence (%)	0B-TM3 Genome proportion (%)	Divergence (%)	B+ Genome proportion (%)	Divergence (%)	PFGE-B Genome proportion (%)	Divergence (%)	B+/0B-WT	B+/0B-TM3
DNA/BARI_Mariner-Tc1	0.0749	0.99	0.0656	1.36	0.0771	0.92	0.0137	0.90	1.03	1.18
DNA/DNAREP1_Helitron	0.5458	13.91	0.5409	13.88	0.5069	13.77	0.1091	13.56	0.93	0.94
DNA/FB4_Mariner-Tc1	0.0729	9.03	0.0750	8.91	0.0833	7.71	0.0219	7.57	1.14	1.11
DNA/HELITRON1_Helitron	0.0002	0.02	0.0002	0.00	0.0001	0.00	0.0001	0.00	0.62	0.61
DNA/HOBOhAT	0.0233	12.31	0.0367	9.85	0.0607	3.87	0.0158	4.91	2.61	1.65
DNA/LOOPER1_piggyBac	0.0060	4.89	0.0064	5.37	0.0052	4.68	0.0012	3.50	0.85	0.81
DNA/M4Transib	0.0372	5.58	0.0404	5.31	0.0371	5.30	0.0093	4.11	1.00	0.92
DNA/MARINER2_Mariner-Tc1	0.0141	6.16	0.0142	6.06	0.0139	5.96	0.0029	5.70	0.98	0.97
DNA/PLACW_P-element	0.0009	1.14	0.0009	1.33	0.0008	1.80	0.0001	1.01	0.87	0.85
DNA/POGOMariner-Tc1	0.0256	0.25	0.0361	0.25	0.0223	0.26	0.0064	0.25	0.87	0.62
DNA/POGON1Mariner-Tc1	0.0016	0.18	0.0016	0.30	0.0022	0.50	0.0004	0.09	1.32	1.36
DNA/RP1DNA	0.0076	11.81	0.0075	11.62	0.0068	11.60	0.0021	11.35	0.89	0.91
DNA/S_Mariner-Tc1	0.0932	8.82	0.0854	8.90	0.0795	8.92	0.0178	8.71	0.85	0.93
DNA/S2_Mariner-Tc1	0.0159	5.55	0.0153	5.43	0.0124	5.31	0.0032	5.84	0.78	0.81
DNA/TC1_Mariner-Tc1	0.0300	4.50	0.0296	4.45	0.0295	4.50	0.0057	4.29	0.98	1.00
DNA/TC1-2_Mariner-Tc1	0.0268	5.32	0.0262	5.32	0.0200	5.29	0.0046	4.66	0.75	0.76
DNA/Transib-N1_Transib	0.0045	12.65	0.0044	12.93	0.0036	13.23	0.0009	13.50	0.81	0.83
DNA/Transib1Transib	0.0100	4.10	0.0106	4.06	0.0084	3.95	0.0017	4.28	0.84	0.80
DNA/TRANSIB2Transib	0.0497	4.62	0.0506	4.52	0.0448	4.47	0.0090	4.59	0.90	0.89
DNA/TRANSIB3Transib	0.0182	5.19	0.0181	5.22	0.0164	5.11	0.0033	5.15	0.90	0.91
DNA/Transib4Transib	0.0096	6.05	0.0105	6.00	0.0088	6.19	0.0012	6.31	0.91	0.83
DNA/Transib5Transib	0.0225	6.26	0.0218	6.35	0.0189	6.24	0.0034	6.33	0.84	0.87
DNA/TRANSPAC_IGypsy	0.0891	0.48	0.0937	0.51	0.0723	0.53	0.0159	0.34	0.81	0.77
LINE/BAGGINS1_LINE_Loa	0.1918	4.61	0.2226	4.69	0.2105	4.60	0.0437	4.75	1.10	0.95
LINE/BS2Jockey	0.1098	6.45	0.1187	6.16	0.1226	6.05	0.0213	6.41	1.12	1.03
LINE/BS3_Jockey	0.0453	7.00	0.0448	6.94	0.0480	6.95	0.0078	6.67	1.06	1.07
LINE/BS4_Jockey	0.0009	2.74	0.0010	2.97	0.0011	3.92	0.0002	-0.15	1.14	1.03
LINE/BSJockey	0.0564	6.98	0.0585	6.56	0.0628	6.21	0.0116	6.20	1.11	1.07

LINE/CR1ACR1_LINE_R1	0.5338	6.65	0.5341	6.61	0.5139	6.51	0.0939	6.73	0.96	0.96
LINE/DOC2_Jockey	0.0866	2.25	0.0861	2.27	0.1177	2.28	0.6862	3.13	1.36	1.37
LINE/DOC3_Jockey	0.1283	4.25	0.1355	4.27	0.1418	4.21	0.0257	4.23	1.11	1.05
LINE/DOC4_Jockey	0.0235	6.38	0.0235	6.34	0.0237	6.33	0.0035	6.41	1.01	1.01
LINE/DOC5_Jockey	0.0602	4.99	0.0575	4.98	0.0584	5.02	0.0122	5.35	0.97	1.02
LINE/DOC6_Jockey	0.0822	0.21	0.0738	0.23	0.0538	0.18	0.0112	0.20	0.65	0.73
LINE/DOCJockey	0.3547	1.11	0.3142	1.27	0.4091	1.03	0.8153	0.81	1.15	1.30
LINE/FW2_Jockey	0.0195	7.15	0.0194	7.37	0.0188	7.31	0.0028	8.22	0.97	0.97
LINE/FW3_Jockey	0.0132	9.48	0.0125	9.85	0.0125	9.20	0.0030	7.93	0.94	1.00
LINE/FWJockey	0.4693	2.29	0.4826	2.39	0.5572	2.14	0.1203	2.10	1.19	1.15
LINE/G_Jockey	0.0546	7.93	0.0498	13.52	0.0669	10.76	0.3005	18.25	1.22	1.34
LINE/G2_Jockey	0.0628	4.34	0.0654	6.22	0.1371	3.73	2.0496	3.57	2.18	2.10
LINE/G3_Jockey	0.0104	4.77	0.0094	4.80	0.0094	5.66	0.0020	5.35	0.91	1.00
LINE/G4_Jockey	0.0400	4.07	0.0422	4.18	0.0375	4.00	0.0072	3.90	0.94	0.89
LINE/G5_Jockey	0.0486	7.21	0.0528	7.05	0.0567	7.01	0.0322	11.47	1.17	1.07
LINE/G5A_Jockey	0.0297	7.18	0.0317	7.05	0.0320	6.79	0.0049	6.76	1.08	1.01
LINE/G6_Jockey	0.0179	7.15	0.0283	4.50	0.0329	8.73	0.1152	26.92	1.84	1.16
LINE/G7_Jockey	0.0020	9.18	0.0019	9.19	0.0024	8.10	0.0006	6.25	1.20	1.23
LINE/HELENA_RTJockey	0.0101	6.53	0.0091	6.26	0.0094	6.41	0.0019	7.14	0.93	1.04
LINE/HeT-A_Jockey	0.2353	12.44	0.3284	12.07	0.3041	10.77	3.6341	8.71	1.29	0.93
LINE/I_I	0.1555	4.46	0.1636	4.39	0.1991	3.78	0.0570	2.91	1.28	1.22
LINE/IVK_Jockey	0.0926	3.73	0.0919	3.70	0.1028	3.47	0.0212	3.11	1.11	1.12
LINE/JOCKEY2Jockey	0.0185	10.17	0.0196	10.35	0.0168	10.41	0.0054	10.42	0.91	0.86
LINE/JockeyJockey	0.1276	2.52	0.1426	2.37	0.1518	2.40	0.0311	2.06	1.19	1.06
LINE/R1_Non-LTR_LINE_R1	0.6705	2.61	0.8029	2.49	0.6897	2.45	0.2092	2.49	1.03	0.86
LINE/R1-2_Non-LTR_LINE_R1	0.0101	7.10	0.0112	7.11	0.0125	7.32	0.0019	6.96	1.24	1.12
LINE/R2_R2	0.1125	0.72	0.1239	0.58	0.1010	0.66	0.0202	0.61	0.90	0.82
LINE/RT1AR1_LINE_R1	0.1354	3.90	0.1370	3.93	0.1486	3.81	0.0211	4.35	1.10	1.08
LINE/RT1BR1_LINE_R1	0.3118	4.19	0.3272	4.09	0.3397	4.23	0.3290	2.15	1.09	1.04
LINE/RT1CR1_LINE_R1	0.0457	9.39	0.0487	9.30	0.0545	9.15	0.0078	9.03	1.19	1.12
LINE/TAHRE_Jockey	0.1529	13.29	0.2320	11.98	0.1734	11.77	1.0054	15.73	1.13	0.75
LINE/TART_AJockey	0.1709	7.22	0.2076	6.33	0.1904	8.30	0.0861	14.03	1.11	0.92
LINE/TART_BJockey	0.0229	8.30	0.0404	5.39	0.0345	10.89	0.0528	18.91	1.50	0.85
LTR/1731_ICopia	0.0965	3.10	0.1092	2.87	0.1198	2.61	0.0219	2.93	1.24	1.10

LTR/1731_LTRCopia	0.0179	3.75	0.0191	3.74	0.0188	3.63	0.0037	3.55	1.05	0.98
LTR/176-IGypsy	0.2413	2.48	0.2434	2.44	0.2323	2.13	0.0507	2.11	0.96	0.95
LTR/176-LTRGypsy	0.0396	2.48	0.0379	2.44	0.0356	2.19	0.0077	2.05	0.90	0.94
LTR/297_IGypsy	0.2634	7.40	0.3133	6.31	0.2673	6.08	0.0637	5.20	1.01	0.85
LTR/297_LTRGypsy	0.0322	5.27	0.0387	4.34	0.0347	4.04	0.0091	3.30	1.08	0.90
LTR/412_IGypsy	0.2424	3.21	0.2490	3.21	0.2177	2.64	0.0598	1.92	0.90	0.87
LTR/412_LTRGypsy	0.0223	1.99	0.0212	1.87	0.0199	1.64	0.0053	1.34	0.89	0.94
LTR/412B_LTRGypsy	0.0158	3.44	0.0129	3.91	0.0136	3.06	0.0036	2.86	0.86	1.05
LTR/ACCORD_ILTR	0.0637	2.30	0.0680	2.35	0.0674	2.32	0.0121	1.85	1.06	0.99
LTR/ACCORD_LTRGypsy	0.0096	0.32	0.0110	0.38	0.0122	0.25	0.0023	0.27	1.26	1.11
LTR/ACCORD2_IGypsy	0.0714	5.66	0.0714	5.85	0.0690	5.59	0.0136	5.75	0.97	0.97
LTR/ACCORD2_LTRGypsy	0.0059	7.05	0.0056	7.57	0.0067	7.08	0.0016	5.32	1.15	1.20
LTR/BATUMI_IBEL	0.3403	4.02	0.3336	4.04	0.3631	3.86	0.0453	3.89	1.07	1.09
LTR/BATUMI_LTRBEL	0.0077	4.74	0.0075	4.92	0.0080	4.98	0.0013	4.41	1.04	1.07
LTR/BEL_IBEL	0.1898	1.50	0.1449	1.86	0.1005	2.71	0.0267	2.05	0.53	0.69
LTR/BEL_LTRBEL	0.0236	1.24	0.0164	1.64	0.0102	2.23	0.0028	1.70	0.43	0.62
LTR/Bica_IGypsy	0.0571	3.03	0.0562	3.32	0.0608	3.20	0.0102	2.73	1.07	1.08
LTR/Bica_LTRGypsy	0.0034	1.26	0.0032	1.37	0.0033	1.21	0.0005	1.52	0.97	1.03
LTR/BLASTOPIA_IGypsy	0.0809	2.88	0.0884	2.63	0.1296	1.87	0.7757	0.23	1.60	1.47
LTR/BLASTOPIA_LTR_Gypsy	0.0080	1.32	0.0082	1.31	0.0104	1.55	0.0737	1.74	1.30	1.26
LTR/BLOOD_IGypsy	0.1651	1.50	0.2561	1.28	0.1784	1.15	0.0462	0.96	1.08	0.70
LTR/BLOOD_LTRGypsy	0.0132	0.95	0.0183	0.94	0.0208	0.70	0.0045	1.00	1.58	1.14
LTR/BURDOCK_IGypsy	0.0575	6.12	0.0475	7.20	0.0820	3.77	0.0192	2.70	1.42	1.72
LTR/BURDOCK_LTRGypsy	0.0050	3.81	0.0042	4.37	0.0083	2.22	0.0026	1.58	1.66	2.00
LTR/Chimpo_IGypsy	0.0426	0.78	0.0493	0.81	0.0515	0.72	0.0065	0.78	1.21	1.04
LTR/Chimpo_LTRGypsy	0.0054	1.25	0.0057	1.18	0.0056	1.13	0.0007	1.26	1.03	0.98
LTR/Chouto_IGypsy	0.0375	4.90	0.0423	4.32	0.0416	4.46	0.0055	4.69	1.11	0.98
LTR/Chouto_LTRGypsy	0.0093	2.64	0.0107	2.63	0.0113	2.53	0.0022	2.67	1.22	1.06
LTR/COPIA__ICopia	0.3418	1.65	0.3306	1.79	0.1931	2.43	0.0389	2.06	0.56	0.58
LTR/COPIA__LTRCopia	0.0297	0.36	0.0287	0.40	0.0158	0.44	0.0042	0.52	0.53	0.55
LTR/Copia1-I_Copia	0.0650	3.88	0.0628	4.04	0.0591	3.67	0.0122	3.03	0.91	0.94
LTR/Copia1-LTR_Copia	0.0054	0.85	0.0044	0.88	0.0043	0.82	0.0013	0.69	0.79	0.98
LTR/COPIA2I_Copia	0.0862	6.48	0.0972	6.01	0.0904	6.13	0.0165	6.26	1.05	0.93
LTR/COPIA2LTR_Copia	0.0080	1.28	0.0094	0.98	0.0078	1.03	0.0026	1.09	0.98	0.84

LTR/DIVER_IBEL	0.0817	2.04	0.0903	1.82	0.1099	1.69	0.0185	1.37	1.34	1.22
LTR/DIVER_LTRBEL	0.0071	7.17	0.0078	6.40	0.0078	6.22	0.0016	4.80	1.10	1.00
LTR/DIVER2_IBEL	0.0777	8.69	0.0856	8.12	0.0919	7.67	0.0112	8.21	1.18	1.07
LTR/DIVER2_LTRBEL	0.0190	2.87	0.0202	2.82	0.0206	2.77	0.0025	2.65	1.08	1.02
LTR/FROGGER_ICopia	0.0094	15.25	0.0094	14.33	0.0085	12.17	0.0014	13.28	0.91	0.91
LTR/FROGGER_LTRCopia	0.0014	1.39	0.0015	1.36	0.0012	1.24	0.0001	1.28	0.86	0.80
LTR/GTWIN_IGypsy	0.0563	14.81	0.1490	5.73	0.0558	14.33	0.0094	13.37	0.99	0.37
LTR/GTWIN_LTRGypsy	0.0101	11.39	0.0286	5.30	0.0082	13.06	0.0019	10.86	0.81	0.29
LTR/GYPSY_IGypsy	0.2278	5.73	0.2215	6.20	0.1404	10.57	0.0213	10.26	0.62	0.63
LTR/GYPSY_LTRGypsy	0.0420	7.59	0.0419	8.38	0.0243	13.56	0.0049	14.97	0.58	0.58
LTR/Gypsy-5_el-IGypsy	0.1625	1.92	0.1388	2.26	0.1355	2.06	0.0293	1.78	0.83	0.98
LTR/Gypsy-5_el-LTRGypsy	0.0651	3.62	0.0653	3.94	0.0556	3.91	0.0145	3.44	0.85	0.85
LTR/Gypsy1-I_Gypsy	0.2046	4.85	0.1677	5.92	0.1081	9.01	0.0208	8.88	0.53	0.64
LTR/Gypsy1-LTR_Gypsy	0.0152	5.87	0.0131	6.84	0.0095	8.91	0.0020	8.94	0.63	0.72
LTR/GYPSY10_IGypsy	0.0600	9.08	0.0584	8.45	0.0585	9.17	0.0131	6.36	0.98	1.00
LTR/GYPSY10_LTRGypsy	0.0052	6.57	0.0055	5.53	0.0058	5.87	0.0008	4.31	1.11	1.04
LTR/GYPSY11_IGypsy	0.0166	10.22	0.0149	10.70	0.0109	10.07	0.0029	9.51	0.66	0.73
LTR/GYPSY11_LTRGypsy	0.0044	9.56	0.0045	9.60	0.0036	9.84	0.0010	11.08	0.83	0.81
LTR/GYPSY12_IGypsy	0.2187	9.95	0.2245	10.01	0.2293	9.79	0.0276	10.42	1.05	1.02
LTR/GYPSY12_LTRGypsy	0.1038	15.36	0.1033	15.26	0.0997	15.50	0.0147	13.51	0.96	0.97
LTR/GYPSY12A_LTRGypsy	0.0432	14.41	0.0442	14.33	0.0415	14.33	0.0083	14.28	0.96	0.94
LTR/GYPSY2_IGypsy	0.1317	13.70	0.1313	13.48	0.1468	12.64	0.0237	11.82	1.11	1.12
LTR/GYPSY2_LTRGypsy	0.0078	9.29	0.0078	9.52	0.0071	9.62	0.0014	7.45	0.91	0.91
LTR/Gypsy2-I_Gypsy	0.0933	8.47	0.0973	8.10	0.1034	7.54	0.0175	6.56	1.11	1.06
LTR/Gypsy2-LTR_Gypsy	0.0442	13.88	0.0361	13.37	0.0388	13.31	0.0075	12.74	0.88	1.07
LTR/GYPSY3_IGypsy	0.0494	12.69	0.0579	11.62	0.0581	12.21	0.0105	11.31	1.18	1.00
LTR/GYPSY3_LTRGypsy	0.0025	9.11	0.0027	9.85	0.0027	8.89	0.0006	12.68	1.08	1.00
LTR/GYPSY4_IGypsy	0.1385	6.66	0.1461	6.30	0.1305	6.69	0.0218	7.24	0.94	0.89
LTR/GYPSY4_LTRGypsy	0.0098	2.70	0.0107	2.93	0.0100	2.67	0.0022	3.46	1.02	0.93
LTR/GYPSY5_IGypsy	0.0780	17.38	0.0817	17.06	0.0775	17.57	0.0153	16.03	0.99	0.95
LTR/GYPSY5_LTRGypsy	0.0055	13.63	0.0058	12.71	0.0059	12.74	0.0013	14.03	1.07	1.03
LTR/GYPSY6_IGypsy	0.0886	11.79	0.0766	12.63	0.0901	11.73	0.0183	9.74	1.02	1.18
LTR/GYPSY6_LTRGypsy	0.0143	7.68	0.0110	7.79	0.0070	6.77	0.0013	6.07	0.49	0.64
LTR/GYPSY6A_LTRGypsy	0.0235	6.74	0.0248	6.67	0.0228	6.71	0.0059	6.43	0.97	0.92

LTR/GYPSY7_IGypsy	0.0480	7.28	0.0475	7.50	0.0459	7.30	0.0105	6.94	0.96	0.97
LTR/GYPSY7_LTRGypsy	0.0030	8.04	0.0029	8.00	0.0026	8.00	0.0007	7.59	0.85	0.89
LTR/GYPSY8_IGypsy	0.0368	12.15	0.0376	12.63	0.0485	12.27	0.0057	12.24	1.32	1.29
LTR/GYPSY8_LTRGypsy	0.0123	15.13	0.0142	14.86	0.0177	15.44	0.0026	14.82	1.43	1.25
LTR/GYPSY9_IGypsy	0.0207	10.21	0.0190	10.00	0.0194	10.04	0.0031	8.79	0.94	1.02
LTR/GYPSY9_LTRGypsy	0.0024	9.27	0.0023	8.66	0.0022	9.37	0.0006	7.68	0.89	0.95
LTR/HMSBEAGLE_IGypsy	0.1613	5.24	0.1454	5.83	0.1527	5.58	0.0268	4.83	0.95	1.05
LTR/IDEFIX_IGypsy	0.3214	7.62	0.3230	7.50	0.3235	6.66	0.0611	6.51	1.01	1.00
LTR/IDEFIX_LTRGypsy	0.0457	4.55	0.0468	4.38	0.0467	3.69	0.0095	3.70	1.02	1.00
LTR/INVADER1_IGypsy	0.0762	10.55	0.0654	11.89	0.0729	10.44	0.0150	7.18	0.96	1.12
LTR/INVADER1_LTRGypsy	0.0403	8.11	0.0378	7.83	0.0369	7.76	0.0079	6.74	0.92	0.98
LTR/INVADER2_IGypsy	0.1532	2.66	0.1581	2.71	0.1291	2.72	0.0243	3.08	0.84	0.82
LTR/INVADER2_LTRGypsy	0.0168	3.77	0.0166	3.64	0.0154	4.02	0.0031	3.66	0.92	0.93
LTR/INVADER3_IGypsy	0.0774	6.05	0.0724	6.44	0.0757	5.74	0.0144	6.06	0.98	1.05
LTR/INVADER3_LTRGypsy	0.0094	4.89	0.0090	5.08	0.0076	4.58	0.0017	5.51	0.81	0.84
LTR/INVADER4_IGypsy	0.0374	6.49	0.0444	5.98	0.0439	6.29	0.0063	5.35	1.17	0.99
LTR/INVADER4_LTRGypsy	0.0128	3.06	0.0164	3.06	0.0123	2.93	0.0027	3.39	0.96	0.75
LTR/INVADER5_IGypsy	0.0117	20.61	0.0123	20.82	0.0119	21.03	0.0023	18.70	1.01	0.96
LTR/INVADER5_LTRGypsy	0.0018	6.68	0.0019	6.94	0.0017	6.96	0.0003	7.31	0.98	0.91
LTR/INVADER6_IGypsy	0.0422	21.31	0.0473	19.37	0.0430	19.40	0.0074	17.13	1.02	0.91
LTR/INVADER6_LTRGypsy	0.0028	2.27	0.0029	2.21	0.0028	2.03	0.0004	2.38	1.00	0.96
LTR/LTR5Gypsy	0.0131	5.78	0.0128	5.57	0.0124	5.42	0.0025	5.41	0.95	0.97
LTR/LTRMDG3_Gypsy	0.0100	2.49	0.0101	2.38	0.0152	1.68	0.0030	1.81	1.52	1.51
LTR/MAX_IBEL	0.2095	4.87	0.2540	4.14	0.2543	4.15	0.0371	4.50	1.21	1.00
LTR/MAX_LTRBEL	0.0114	5.53	0.0137	5.01	0.0151	5.07	0.0017	7.83	1.32	1.10
LTR/MDG1_IGypsy	0.2475	3.19	0.2381	3.34	0.1827	3.23	0.0455	3.01	0.74	0.77
LTR/MDG1_LTRGypsy	0.0231	5.28	0.0219	5.26	0.0189	5.54	0.0058	4.79	0.82	0.87
LTR/MDG3_Gypsy	0.1074	2.25	0.1188	2.03	0.1565	1.38	0.0347	1.42	1.46	1.32
LTR/MICROPIA_ILTR	0.1405	12.63	0.1377	12.92	0.1310	12.47	0.0187	11.94	0.93	0.95
LTR/MICROPIA_LTRLTR	0.0159	2.77	0.0156	2.81	0.0165	2.70	0.0027	2.28	1.04	1.06
LTR/NOMAD_IGypsy	0.2197	2.41	0.2173	2.21	0.2320	2.33	0.0406	2.25	1.06	1.07
LTR/NOMAD_LTRGypsy	0.0252	0.61	0.0297	0.65	0.0330	0.50	0.0052	0.60	1.31	1.11
LTR/QUASIMODO_IGypsy	0.3017	5.12	0.3564	4.69	0.2743	4.54	0.0529	5.03	0.91	0.77
LTR/QUASIMODO_LTRGypsy	0.0557	3.26	0.0615	3.33	0.0616	2.70	0.0113	3.58	1.11	1.00

LTR/QUASIMODO2-I_Gypsy	0.0784	3.75	0.0647	4.41	0.0659	3.98	0.0105	4.62	0.84	1.02
LTR/QUASIMODO2-LTR_Gypsy	0.0150	1.45	0.0130	2.22	0.0118	1.80	0.0024	3.48	0.78	0.90
LTR/ROO_IBEL	0.9484	1.59	1.1071	1.40	0.9324	1.85	2.9398	0.25	0.98	0.84
LTR/ROO_LTRBEL	0.1018	0.72	0.1139	0.70	0.0991	0.67	0.2164	0.22	0.97	0.87
LTR/ROOA_IBEL	0.0866	4.00	0.0904	3.99	0.0849	3.99	0.0136	4.04	0.98	0.94
LTR/ROOA_LTRBEL	0.0142	3.78	0.0148	3.73	0.0119	3.53	0.0028	3.46	0.84	0.80
LTR/ROVER_Gypsy	0.1777	10.40	0.1645	10.72	0.2090	7.73	1.4284	0.37	1.18	1.27
LTR/STALKER2_IGypsy	0.1333	4.99	0.1374	5.66	0.0935	6.76	0.0209	5.68	0.70	0.68
LTR/STALKER2_LTRGypsy	0.0124	2.66	0.0116	3.24	0.0087	2.99	0.0020	4.22	0.70	0.75
LTR/STALKER3_LTRGypsy	0.0080	7.83	0.0105	6.19	0.0080	7.03	0.0018	7.78	1.01	0.77
LTR/STALKER4_IGypsy	0.4086	3.62	0.4048	3.96	0.3406	3.65	0.0820	3.70	0.83	0.84
LTR/STALKER4_LTRGypsy	0.0501	4.02	0.0469	4.07	0.0394	4.28	0.0114	3.49	0.79	0.84
LTR/TABOR_IGypsy	0.0824	3.74	0.0960	3.84	0.0793	3.05	0.0196	2.93	0.96	0.83
LTR/TABOR_LTRGypsy	0.0092	1.47	0.0113	1.29	0.0090	1.36	0.0020	0.80	0.98	0.80
LTR/TIRANT_Gypsy	0.0032	15.20	0.0063	13.31	0.0066	11.29	0.0015	16.25	2.03	1.04
LTR/TIRANT_IGypsy	0.0315	19.84	0.0406	16.25	0.0394	15.60	0.0087	16.03	1.25	0.97
LTR/TOM1_LTRGypsy	0.0073	8.99	0.0072	9.15	0.0062	8.05	0.0008	10.12	0.84	0.85
LTR/TRANSPAC_Gypsy	0.0119	0.55	0.0125	0.48	0.0111	0.43	0.0030	0.52	0.93	0.89
LTR/ZAM_IGypsy	0.0484	8.71	0.0591	7.33	0.0567	7.66	0.0096	6.68	1.17	0.96
LTR/ZAM_LTRGypsy	0.0054	9.78	0.0066	8.45	0.0068	9.20	0.0014	7.98	1.25	1.02
**Total abundance**	**15.4655**		**16.3853**		**15.5206**		**17.0422**		**1.00**	**0.95**
**Divergence average**		**6.01**		**5.86**		**5.86**		**5.86**		
