# Supplementary material for: Comparative genomic analysis and epigenetic profiling of the Drosophila melanogaster B chromosome
Source: Genetics. 2026 May 5;233(4):iyag113. doi: 10.1093/genetics/iyag113 (PMC13439713; doi:10.1093/genetics/iyag113)
Supplement: Supplementary file 2 [file iyag113_supplementary_data.zip › Supplementary_Table_5_GENETICS-2026-309266.pdf]

Supplementary Table 5. Genome proportion (%) and divergence (K2P %) of each multigene family in the three genomes analyzed (0B-WT, 0B-TM3 and +B) of *Drosophila melanogaster* and the sequenced pulsed field electrophoresis B (PFGE -B).

Multigene family	0B-WT Genome proportion (%)	Divergence (%)	0B-TM3 Genome proportion (%)	Divergence (%)	B+ Genome proportion (%)	Divergence (%)	PFGE-B Genome proportion (%)	Divergence (%)	B+/0B-WT	B+/0B-TM3
**Ribosomal DNA**										
45S	0.003814	2.27	0.004299	2.22	0.003632	2.24	0.186069	10.1	1.0	0.8
5S	0.000121	0.46	0.000139	0.47	0.000132	0.43	0.005683	0.62	1.1	0.9
**Total (2)**	**0.003935**		**0.004438**		**0.003764**		**0.191753**		**1.0**	**0.8**
**Spliceosomal DNA**										
U1	0.000008	0.56	0.00001	0.5	0.000008	0.33	0.000155	0.6	0.9	0.8
U2	0.000012	1.59	0.00001	1.61	0.00001	1.98	0.000188	7.13	0.9	1.0
U4	0.000004	8.84	0.000003	9.07	0.000003	9.28	0.000049	25.1	0.9	1.0
U5	0.000006	2.43	0.000006	2.6	0.000004	2.39	0.000119	3.55	0.7	0.7
U6	0.000003	1.01	0.000002	1.1	0.000002	0.96	0.000433	11.37	0.8	1.4
**Total (5)**	**0.000032**		**0.000031**		**0.000028**		**0.000944**		**0.9**	**0.9**
**Histones genes**										
H1	0.000825	0.3	0.000779	0.44	0.00071	0.25	0.012737	0.3	0.9	0.9
H2A	0.000397	0.44	0.000362	0.43	0.000377	0.39	0.008884	0.84	0.9	1.0
H2B	0.000562	0.49	0.000522	0.55	0.000472	0.4	0.011241	0.75	0.8	0.9
H3	0.00041	0.33	0.000371	0.44	0.000396	0.3	0.007414	1.29	1.0	1.1
H4	0.000378	0.77	0.000361	0.76	0.000315	0.67	0.005954	1.52	0.8	0.9
**Total (5)**	**0.002572**		**0.002394**		**0.00227**		**0.046231**		**0.9**	**0.9**
